# FAM134B-mediated ER-phagy degrades APP and suppresses Alzheimer’s disease pathology

**DOI:** 10.1038/s44318-026-00818-9

**Published:** 2026-05-26

**Authors:** Yuting Zhang, Jun Sun, Yang Cai, Ziyan Xu, Xiang Li, Wei Wei, Pai Liu, Qiming Sun, Zhi-Hao Wang, Yixian Cui

**Affiliations:** 1 Department of Neurology, Department of Urology, Medical Research Institute, Frontier Science Center for Immunology and Metabolism, Zhongnan Hospital of Wuhan University, Wuhan University, Wuhan, China; 2 Department of Neurosurgery, Zhongnan Hospital of Wuhan University, Wuhan, China; 3 Department of Pathology and Laboratory Medicine, Emory University School of Medicine, Atlanta, GA USA; 4 Department of Respiratory and Critical Care Medicine, Center for Metabolism Research, Fourth Affiliated Hospital, Zhejiang University School of Medicine and International School of Medicine, International Institutes of Medicine, Zhejiang University, Yiwu, China; 5 Departments of Biochemistry and Cardiology, Second Affiliated Hospital, Zhejiang University School of Medicine, Hangzhou, China; 6 Department of Neurology, Renmin Hospital of Wuhan University, Wuhan, China; 7 Center for Neurodegenerative Disease Research, Renmin Hospital of Wuhan University, Wuhan, China

**Keywords:** Autophagy & Cell Death, Molecular Biology of Disease, Post-translational Modifications & Proteolysis

## Abstract

Endoplasmic reticulum autophagy (ER-phagy) is a selective autophagy pathway in which receptor proteins target ER membranes and proteins for degradation, yet its role in Alzheimer’s disease (AD) remains unclear. Here, we identify FAM134B/RETREG1 as a specific ER-phagy receptor mediating amyloid precursor protein (APP) degradation. FAM134B directly interacts with ER-localized wild-type and familial mutant APP via their C-terminal domains and recruits LC3 through its LC3-interacting region (LIR) to promote APP delivery to phagophores for lysosomal degradation. In AD, epigenetic silencing at the *FAM134B* promoter suppresses its transcription by limiting TFEB/TFE3 binding despite their nuclear enrichment. This transcriptional suppression impairs ER-phagy, leading to APP accumulation and exacerbated AD pathology. AAV-mediated hippocampal expression of wild-type, but not LIR-mutant, FAM134B in 5XFAD mice restores ER-phagy, enhances APP clearance, reduces Aβ deposition, preserves synaptic and myelin integrity, and improves cognitive performance. These findings establish FAM134B downregulation as an upstream pathogenic event in AD, suggesting ER-phagy enhancement as a promising strategy to suppress Aβ generation at its source.

## Synopsis

Reduced clearance of amyloid precursor protein (APP) is implicated in Alzheimer’s disease (AD), yet whether endoplasmic reticulum autophagy (ER-phagy) contributes to this process remains unclear. This study shows that epigenetic suppression of the ER-phagy receptor FAM134B reduces APP clearance and that restoring FAM134B reduces Aβ pathology and cognitive deficits in vivo.

- FAM134B directly binds ER-localized wild-type and familial mutant APP and recruits LC3 to promote ER-phagy–mediated degradation.
- Epigenetic repression of FAM134B limits TFEB/TFE3 binding, thereby suppressing ER-phagy under AD conditions.
- Impaired ER-phagy leads to APP accumulation, which in turn suppresses ER-phagy, forming a feed-forward loop that amplifies Aβ production.
- AAV-mediated FAM134B expression restores ER-phagy, reduces Aβ burden, and improves cognition in 5XFAD mice.

## Abstract (web summary)

FAM134B directly binds ER-localized amyloid precursor protein and promotes its degradation by ER-phagy.

## Graphical abstract

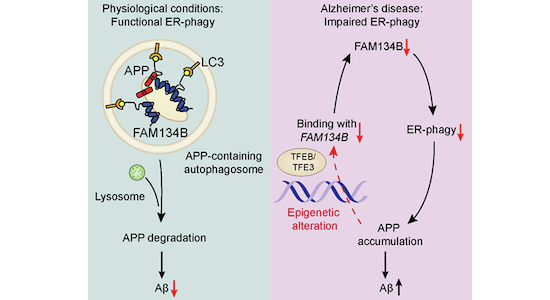

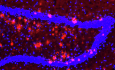

## Introduction

Macroautophagy (hereafter autophagy) is an evolutionarily conserved process in which cytosolic substrates are engulfed by a double-membrane phagophore, forming autophagosomes that fuse with vacuoles or lysosomes for degradation (Klionsky et al, 2021; Nakatogawa et al, 2009; Zhu et al, 2024). Autophagy operates either non-selectively or selectively. The latter depends on receptor proteins that link specific cargo to ATG8 family proteins (LC3s/GABARAPs) for targeted degradation (Lamark and Johansen, 2021). Selective autophagy is classified into distinct forms based on cargo type, such as aggrephagy for protein aggregates, mitophagy for mitochondria, and ER-phagy for the endoplasmic reticulum (ER) (Lamark and Johansen, 2021).

The ER is essential for the synthesis and folding of more than one-third of proteins. ER-phagy functions at a basal level to maintain ER homeostasis and is further activated in response to nutrient stress or the accumulation of misfolded or aggregated proteins within the ER (He et al, 2021; Khaminets et al, 2015; Qian et al, 2023). ER-phagy regulates neurogenesis (Hoyer et al, 2024), and its dysfunction is implicated in neuropathy. Loss-of-function mutations in the ER-phagy receptor FAM134B cause hereditary sensory and autonomic neuropathy (HSAN) type II (Davidson et al, 2012; Khaminets et al, 2015; Kurth et al, 2009; Wakil et al, 2018). Mutations in ATL3, an ER-shaping GTPase functioning as an ER-phagy receptor, have been linked to HSAN type I (Behrendt et al, 2019; Chen et al, 2019; Fischer et al, 2014; Kornak et al, 2014), while RTN3L, a receptor enriched in neuronal tissues, has been implicated in Alzheimer’s disease (AD) (Grumati et al, 2017a; Zou et al, 2018). Additionally, mutations in ARL6IP1, an ER-shaping protein that interacts with FAM134B, also manifest in HSAN (Foronda et al, 2023). Despite these findings, the mechanistic contribution of ER-phagy to neurodegenerative disease remains largely unexplored.

AD, the most prevalent form of neurodegenerative disease, is characterized pathologically by intracellular neurofibrillary tangles and extracellular amyloid-beta (Aβ) plaques (Alzheimer’s, 2024; DeTure and Dickson, 2019). Aβ is generated via sequential cleavage of amyloid precursor protein (APP), a type I transmembrane protein synthesized in the ER (Seeman and Seeman, 2011). Genetic mutations in APP, PSEN1, and PSEN2 increase Aβ production and are causative for familial AD (Price and Sisodia, 1998; Seeman and Seeman, 2011). Notably, APP gene duplication is also sufficient to cause early-onset AD (Rovelet-Lecrux et al, 2006), and virtually all individuals with Down syndrome—who harbor an extra APP copy—develop Aβ pathology by midlife (Wiseman et al, 2015). These genetic findings provide compelling evidence supporting a causal role of APP dosage in AD pathogenesis and highlight APP clearance as a potential therapeutic target.

Autophagy dysfunction and ER abnormalities are consistently observed under AD conditions (Ajoolabady et al, 2022; Nixon and Yang, 2011; Peric and Annaert, 2015; Shin et al, 2014). While most studies investigating autophagy in AD have focused on non-selective autophagy or mitophagy in relation to Tau and Aβ clearance, whether ER-phagy directly contributes to AD pathogenesis remains unknown. Given that APP overexpression is causally linked to AD, and that newly synthesized APP traffics through and can accumulate in the ER, we hypothesized that ER-phagy regulates APP turnover and that its impairment may promote APP accumulation and contribute to AD progression.

Here, we identify FAM134B/RETREG1 as a specific ER-phagy receptor responsible for APP degradation. FAM134B directly binds ER-localized wild-type and familial mutant APP via its C-terminal domain, promoting their targeting to phagophores for lysosomal degradation. Familial APP mutants behave similarly to wild-type APP. Under AD conditions, epigenetic silencing at the *FAM134B* promoter—characterized by decreased active histone modifications and chromatin accessibility—impairs TFEB/TFE3 binding and downregulates *FAM134B* transcription. This defect compromises ER-phagy, leading to APP accumulation and exacerbation of AD pathology. Hippocampal overexpression of FAM134B in the 5XFAD mouse model restores ER-phagy, reduces APP levels and Aβ deposition, and improves cognitive performance. Together, these findings establish ER-phagy impairment as an upstream pathogenic event in AD and highlight FAM134B as a potential therapeutic target to suppress Aβ production at its source.

## Results

### FAM134B expression is reduced and ER turnover is compromised in AD patients and the 5XFAD mouse model

**Figure EV1:**
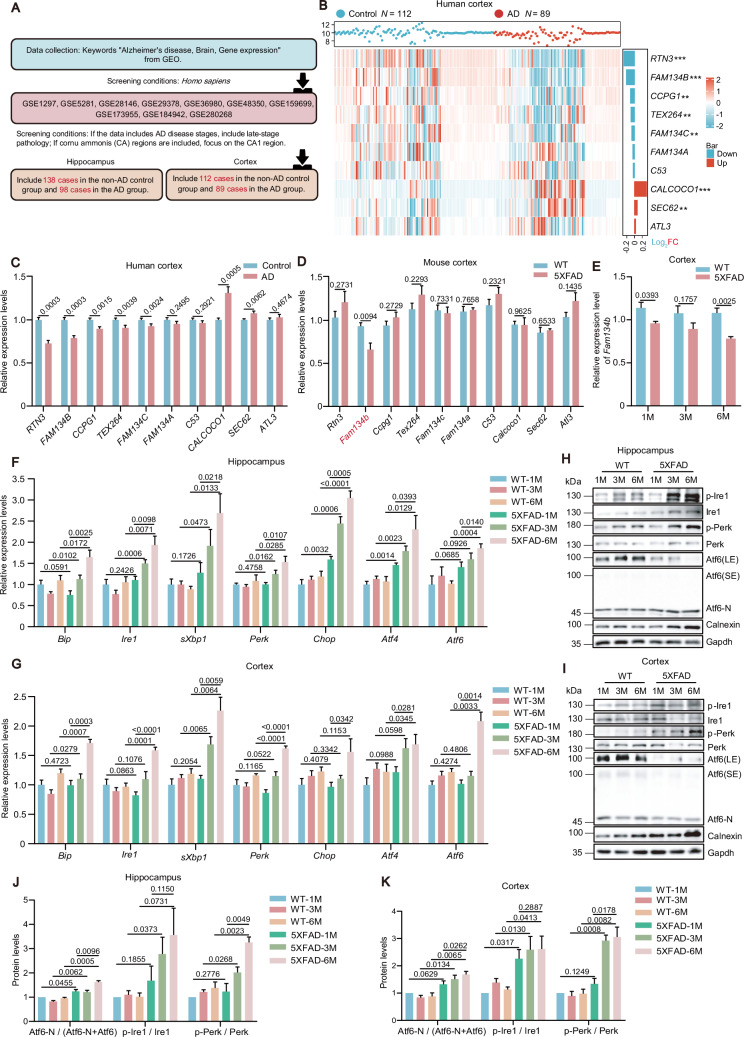
ER-phagy receptors are downregulated and UPR is upregulated in AD, related to Fig. 1. (**A**) Cross-database normalized microarray and RNA-seq analysis workflow. (**B**, **C**) Cross-database normalized microarray and RNA-seq analysis of ER-phagy receptor mRNA levels in the cortex of AD patients (*N* = 89) and non-AD controls (*N* = 112). *P* values were adjusted according to the Benjamini–Hochberg false discovery rate (FDR) correction. (**D**) qRT-PCR analysis of ER-phagy receptor mRNA levels in the cortex of 6-month-old 5XFAD mice and WT littermates (*N* = 3 per group). (**E**) qRT-PCR analysis of *Fam134b* mRNA levels in the cortex of 1-, 3-, and 6-month-old 5XFAD female mice and WT littermates (*N* = 3 per group). (**F**, **G**) qRT-PCR analysis of mRNA levels of UPR-related genes in the hippocampus and cortex of 1-, 3-, and 6-month-old 5XFAD female mice and WT littermates (*N* = 4 per group). (**H**, **I**) Immunoblotting of UPR-related protein levels in the hippocampus and cortex of 1-, 3-, and 6-month-old 5XFAD female mice and WT littermates (*N* = 3 per group). p-Ire1 and p-Perk represent phosphorylated Ire1 and Perk, indicative of activated Ire1 and Perk; Atf6-N represents the cleaved, transcriptionally active N-terminal fragment of Atf6. LE long exposure, SE short exposure. (**J**, **K**) Quantification of protein levels in (**H**, **I**) (*N* = 3 per group). Error bars represent SEM; ns, no significance, *P* > 0.05, *P < 0.05, **P < 0.01, ***P < 0.001, ****P < 0.0001; (**C**–**E**) were analyzed by unpaired Student’s *t* test; (**F**, **G**, **J**, **K**) were analyzed by two-way ANOVA.

**Figure 1:**
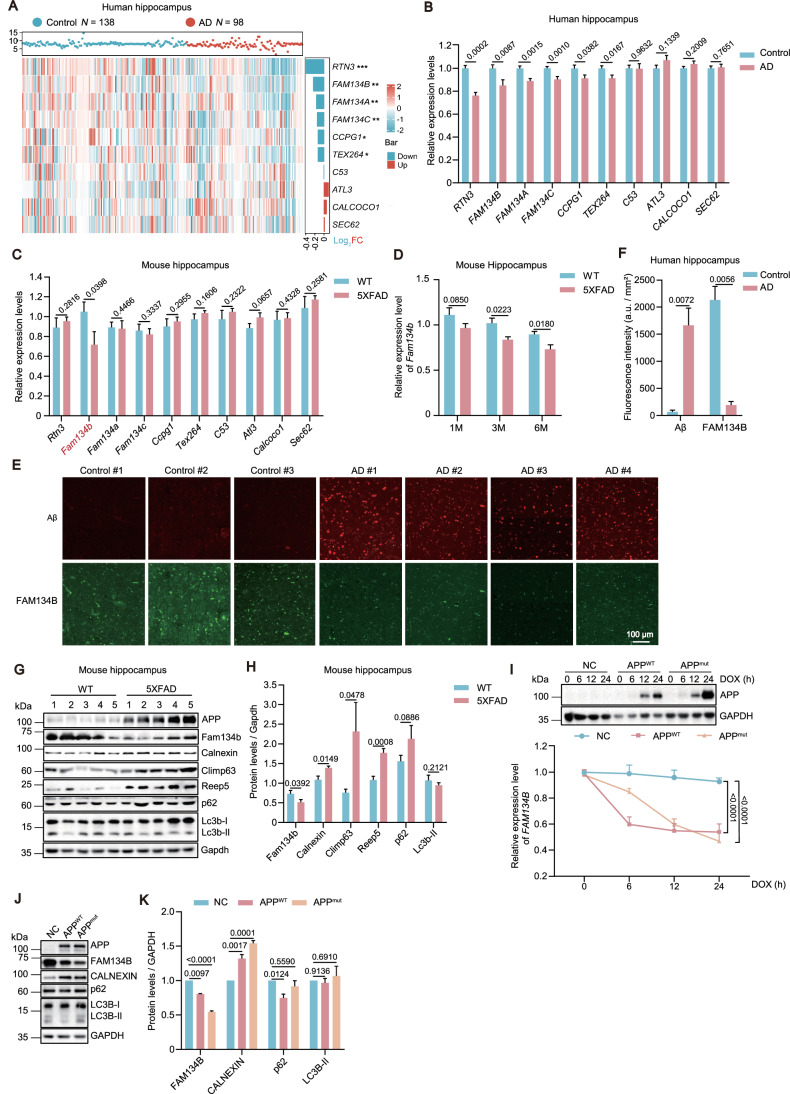
ER-phagy receptors are downregulated and ER-phagy is impaired in AD. (**A**, **B**) Cross-database normalized microarray and RNA-seq analysis of ER-phagy receptor mRNA levels in hippocampus from AD patients (*N* = 98) and non-AD controls (*N* = *138*). *P* values were adjusted according to the Benjamini–Hochberg false discovery rate (FDR) correction. (**C**) qRT-PCR analysis of ER-phagy receptor mRNA levels in the hippocampus of 6-month-old 5XFAD female mice and WT littermates (*N* = 5 per group). (**D**) qRT-PCR analysis of *Fam134b* mRNA levels in the hippocampus of 1-, 3-, and 6-month-old 5XFAD female mice and WT littermates (*N* = 4 per group). (**E**) Immunostaining of FAM134B and Aβ in hippocampal sections from four AD patients and three non-AD controls. (**F**) Quantification of FAM134B and Aβ immunofluorescence intensity in (**E**). Quantification for each independent sample was performed across 40 random 0.25 mm² areas, with a single representative area displayed in (**E**). (**G**) Immunoblotting of protein levels in the hippocampus of 6-month-old 5XFAD mice and WT littermates (*N* = 5 per group). Calnexin: ER-resident protein, present in both sheet and tubular ER; Climp63: sheet ER marker; Reep5: tubular ER marker; p62: non-selective autophagy substrate; Lc3b-II: lipidated Lc3b, a marker of autophagy; Gapdh, loading control. (**H**) Quantification of protein levels in (**G**). (**I**) Top: Immunoblotting of APP^WT/mut^ protein levels following doxycycline (DOX) treatment to induce expression in U2OS cells integrated with pLVX-TetOne-Puro-APP^WT^ or pLVX-TetOne-Puro-APP^mut^. Bottom: qRT-PCR analysis of *FAM134B* mRNA levels in matched cell samples. *n* = 3. (**J**) Immunoblotting of protein levels in control and U2OS cells expressing DOX-inducible APP^WT/mut^ after 24 h of induction. (**K**) Quantification of protein levels in (**J**). *n* = 3. Error bars represent SEM; ns, no significance, *P* > 0.05, *P < 0.05, **P < 0.01, ***P < 0.001, ****P < 0.0001; (**B**–**D**, **F**), and (**H**) were analyzed by unpaired Student’s *t* test; (**I**, **K**) were analyzed by one-way ANOVA. Source data are available online for this figure.

To investigate the role of ER-phagy in AD pathogenesis, we analyzed the transcript levels of ten known ER-phagy receptors (FAM134B (Khaminets et al, 2015), SEC62 (Fumagalli et al, 2016), RTN3L (Grumati et al, 2017b), CCPG1 (Smith et al, 2018), ATL3 (Chen et al, 2019), TEX264 (An et al, 2019; Chino et al, 2019), CALCOCO1 (Nthiga et al, 2020; Stefely et al, 2020), C53 (Stephani et al, 2020), FAM134A (Reggio et al, 2021), and FAM134C (Reggio et al, 2021)) across ten hippocampal and cortical microarray/RNA-seq datasets from the Gene Expression Omnibus (GEO), comparing AD patients and non-AD controls (Fig. EV1A). This analysis revealed a significant reduction in the mRNA levels of several ER-phagy receptors, including RTN3, FAM134B, FAM134C, CCPG1, and TEX264, in both the hippocampus and cortex of AD patients compared to non-AD controls (Figs. 1A,B and EV1B,C).

To validate the transcriptional downregulation of ER-phagy receptors, we analyzed their mRNA levels in the widely used 5XFAD mouse model. The 5XFAD model expresses human APP695 with the Swedish (K670N/M671L), Florida (I716V), and London (V717I) mutations (APP770 numbering; corresponding to K595N/M596L, I641V, and V642I in APP695) and PSEN1 M146L/L286V (Oakley et al, 2006). 5XFAD mice exhibit hallmark features of AD, including amyloid plaque deposition, progressive neuronal loss, and cognitive deficits (Chishti et al, 2001; Jawhar et al, 2012; Oakley et al, 2006). qRT-PCR analysis revealed a significant decrease in *Fam134b* mRNA levels in both the hippocampus and cortex of 6-month-old 5XFAD mice, while the mRNA levels of other ER-phagy receptors remained largely unchanged (Figs. 1C and EV1D). We further examined younger 5XFAD mice and found that *Fam134b* transcription was already downregulated in the hippocampus and cortex at 1 month of age, with a progressive decline in mRNA levels as the mice aged (Figs. 1D and EV1E). Notably, this reduction precedes intraneuronal Aβ42 accumulation (~1.5 months) and plaque deposition (~2 months) (Oakley et al, 2006). These results demonstrate a common transcriptional downregulation of *FAM134B* in the hippocampus and cortex of both AD patients and 5XFAD mice.

Immunofluorescence staining and quantification revealed a marked reduction in FAM134B protein levels in the hippocampus of AD patients with abundant Aβ plaques, compared to non-AD controls exhibiting undetectable Aβ levels and relatively high FAM134B protein levels (Fig. 1E,F). Immunoblotting and quantification of hippocampal samples from 6-month-old 5XFAD mice revealed significantly increased levels of APP, accompanied by a marked decrease in Fam134b protein levels compared to age-matched control mice (Fig. 1G,H). Notably, we also detected significantly increased levels of ER marker proteins, including Calnexin, Climp63, and Reep5, in 5XFAD mice (Fig. 1G,H), consistent with impaired ER turnover. By contrast, levels of non-selective autophagy markers—including lipidated LC3B (LC3B-II)—showed no significant changes, while SQSTM1/p62 levels exhibited a non-significant upward trend (Fig. 1G,H). Because impaired ER turnover is associated with activation of the unfolded protein response (UPR) (Liang et al, 2020), we examined UPR markers. We observed that UPR-related genes, across all three major UPR pathways, were upregulated at both the transcriptional and protein levels in 5XFAD mice, with an increasing trend as the mice aged (Fig. EV1F–K). These findings collectively demonstrate that Fam134b expression as well as ER turnover and homeostasis are compromised in the 5XFAD mouse model of AD.

### APP accumulation impairs FAM134B receptor function and ER-phagy in AD cellular models

**Figure EV2:**
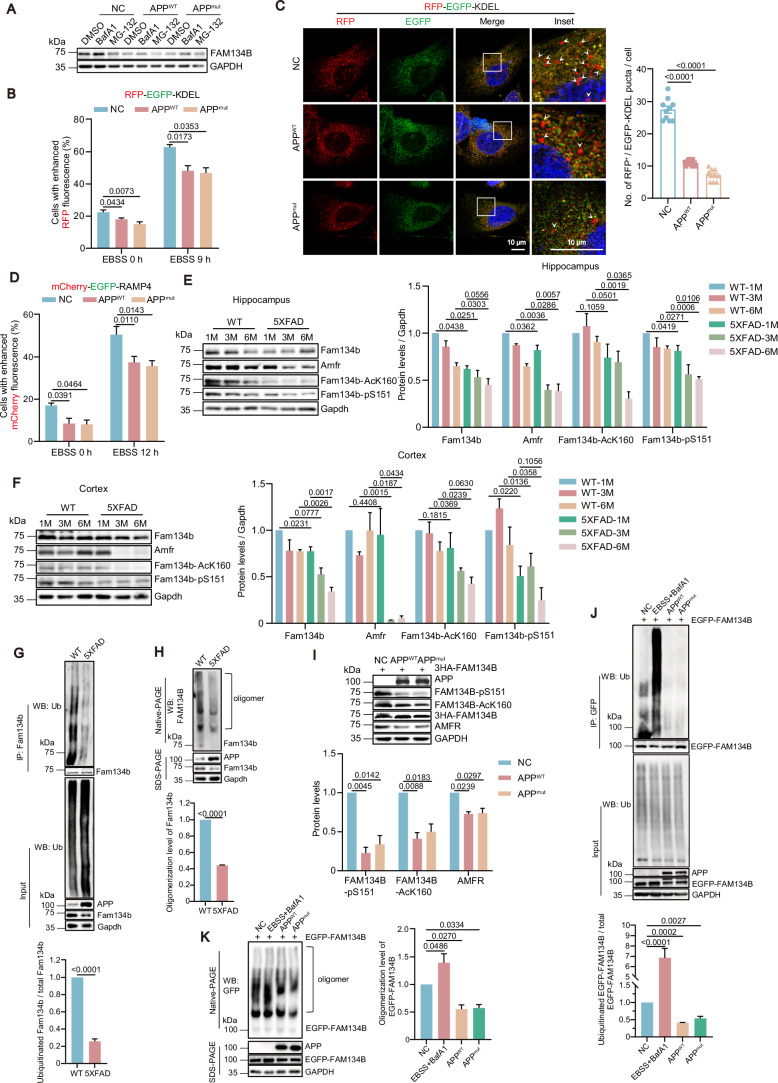
Impaired ubiquitination and oligomerization of the ER-phagy receptor FAM134B in AD, related to Fig. 1. (**A**) Immunoblotting of protein levels in control and HEK293T cells expressing DOX-inducible APP^WT/mut^. Cells were either untreated or treated with BafA1 or MG-132 for 6 h. (**B**) Quantification of cells with an increased RFP/EGFP (-KDEL) ratio by flow cytometry to assess ER-phagy flux. U2OS cells stably expressing RFP-EGFP-KDEL reporter and DOX-inducible APP^WT/mut^ were treated with DOX for 24 h to induce APP expression, followed by EBSS treatment or left untreated. *n* = 3. (**C**) Confocal images of U2OS cells stably expressing RFP-EGFP-KDEL reporter and DOX-inducible APP^WT/mut^. Cells were induced with DOX for 24 h, followed by EBSS treatment for 6 h. Arrowheads indicate RFP^+^/EGFP^-^ puncta. Right: Quantification of RFP^+^/EGFP^-^ puncta. *N* = 10 cells. (**D**) Quantification of cells with an increased mCherry/EGFP (-RAMP4) ratio by flow cytometry to assess ER-phagy flux. U2OS cells stably expressing mCherry-EGFP-RAMP4 reporter and DOX-inducible APP^WT/mut^ were treated with DOX for 24 h to induce APP expression, followed by EBSS treatment or left untreated. *n* = 3. (**E**, **F**) Immunoblotting of protein levels in the hippocampus (**E**) and cortex (**F**) of 1-, 3-, and 6-month-old 5XFAD female mice and WT littermates (*N* = 3 per group). Fam134b-pS151 and Fam134b-AcK160 indicate Fam134b phosphorylated at Ser151 and acetylated at Lys160, respectively. (**G**) Fam134b ubiquitination levels in the hippocampus of 6-month-old WT and 5XFAD female mice (*N* = 3 per group). Fam134b was immunoprecipitated with anti-Fam134b antibodies and analyzed by immunoblotting with anti-ubiquitin antibodies. Bottom: Quantification of ubiquitinated Fam134b normalized to total Fam134b. (**H**) Fam134b oligomerization levels in the hippocampus of 6-month-old WT and 5XFAD female mice (*N* = 3 per group). Top: Native PAGE immunoblotting of Fam134b using anti-Fam134b antibodies. Input protein levels were assessed by SDS-PAGE and immunoblotting. Bottom: Quantification of oligomerized Fam134b normalized to total Fam134b. (**I**) Immunoblotting of protein levels in control and HEK293T cells expressing DOX-inducible APP^WT/mut^. Cells were cultured in nutrient-rich medium and transiently transfected with 3HA–FAM134B. Bottom: Quantification of protein levels shown above. FAM134B-pS151 and FAM134B-AcK160 were normalized to total 3HA–FAM134B; AMFR was normalized to GAPDH. *n* = 3. (**J**) Effect of APP^WT/mut^ overexpression on FAM134B ubiquitination. Top: EGFP-FAM134B was immunoprecipitated with anti-GFP antibodies and analyzed by immunoblotting with anti-ubiquitin antibodies. Lysates were prepared from untreated U2OS cells (NC), EBSS + BafA1-treated U2OS cells (6 h; positive control), and DOX-induced APPWT/mut-expressing U2OS cells cultured in nutrient-rich medium. All groups were transiently transfected with EGFP-FAM134B. Bottom: Quantification of ubiquitinated EGFP-FAM134B normalized to total EGFP-FAM134B. *n* = 3. (**K**) Effect of APP^WT/mut^ overexpression on FAM134B oligomerization. Left: Native PAGE immunoblotting of EGFP-FAM134B using anti-GFP antibodies. Lysates were prepared from NC, EBSS + BafA1, and DOX-induced APP^WT/mut^ U2OS cells. Input protein levels were assessed by SDS-PAGE and immunoblotting. Right: Quantification of oligomerized EGFP-FAM134B normalized to total EGFP-FAM134B. *n* = 3. Error bars represent SEM; ns, no significance, *P* > 0.05, *P < 0.05, **P < 0.01, ***P < 0.001, ****P < 0.0001; (**B**–**D**, **I**–**K**) were analyzed by one-way ANOVA; (**E**, **F**) were analyzed by two-way ANOVA; (**G**, **H**) were analyzed by unpaired Student’s *t* test.

To extend our findings from the 5XFAD mouse model, we generated stable cell lines expressing human wild-type APP695 (APP^WT^) or familial mutant APP (APP^mut^) under a doxycycline-inducible promoter to avoid toxicity from constitutive overexpression. Upon doxycycline induction, APP^WT/mut^ protein levels gradually increased, while *FAM134B* mRNA levels progressively declined (Fig. 1I). Immunoblotting revealed significantly decreased FAM134B protein levels in APP^WT/mut^-expressing cells (Fig. 1J,K). This decrease is not due to increased degradation, as treatment with Bafilomycin A1 (BafA1), an inhibitor of the V-type ATPase that impairs autophagy, or MG-132 (a proteasome inhibitor) did not restore FAM134B protein levels (Fig. EV2A). We also observed increased levels of CALNEXIN in APP^WT/mut^-expressing cells, whereas LC3B-II and p62 levels showed no significant change (Fig. 1J,K). These results demonstrate that APP accumulation suppresses both FAM134B expression and ER turnover in AD cellular models, mirroring observations in 5XFAD mice.

To determine whether APP accumulation impairs ER turnover by inhibiting ER-phagy, we employed RFP-EGFP-KDEL as an ER-phagy reporter. Since lysosomal acidity quenches EGFP but not RFP, increased ER-phagy is indicated by an elevated RFP/EGFP ratio or a higher number of RFP^+^/EGFP^−^ puncta. Flow cytometry revealed a significantly reduced RFP/EGFP ratio in APP^WT/mut^-expressing cells under nutrient-rich conditions (Fig. EV2B). A similar reduction was observed under starvation (EBSS) conditions, which are known to induce ER-phagy (Fig. EV2B). Confocal imaging and quantification also showed markedly fewer RFP^+^/EGFP^−^ puncta in APP^WT/mut^-expressing cells than in controls under starvation conditions (Fig. EV2C). Similar results were obtained using mCherry-EGFP-RAMP4 as an independent ER-phagy reporter (Fig. EV2D). These data demonstrate that APP accumulation impairs ER turnover by inhibiting ER-phagy.

We then investigated whether impaired ER-phagy in AD models is due solely to decreased FAM134B protein levels or also results from reduced receptor activity. Phosphorylation of FAM134B at Ser151 promotes acetylation at Lys160, enhancing its oligomerization (Jiang et al, 2020; Wang et al, 2023). AMFR-mediated ubiquitination also facilitates oligomerization, which promotes ER fragmentation and ER-phagy (Gonzalez et al, 2023). Immunoblotting revealed that both Ser151 phosphorylation and Lys160 acetylation were significantly reduced in 5XFAD mouse brains, accompanied by decreased Amfr levels and reduced Fam134b ubiquitination, indicating impaired post-translational regulation (Fig. EV2E–G). Consistently, native PAGE immunoblotting revealed a marked decrease in FAM134B oligomerization in 5XFAD mouse brains (Fig. EV2H). Together, these data indicate that Fam134b receptor activity is compromised in 5XFAD mice.

While FAM134B protein levels are still reduced in 5XFAD mice (Figs. 1G and EV2E,F), these defects could be a secondary consequence of decreased protein abundance. To address this, we expressed FAM134B in APP-overexpressing cells to equalize FAM134B abundance. Despite similar expression levels of FAM134B, post-translational modifications and oligomerization remained reduced in both wild-type and mutant APP-expressing cells (Fig. EV2I–K). This suggests that the observed effects are not solely due to reduced FAM134B levels but may involve an independent regulatory mechanism. Collectively, these findings suggest that the decreased expression and reduced receptor activity of FAM134B contribute to compromised ER-phagy and ER homeostasis in AD.

### ER-localized APP is degraded by ER-phagy

**Figure EV3:**
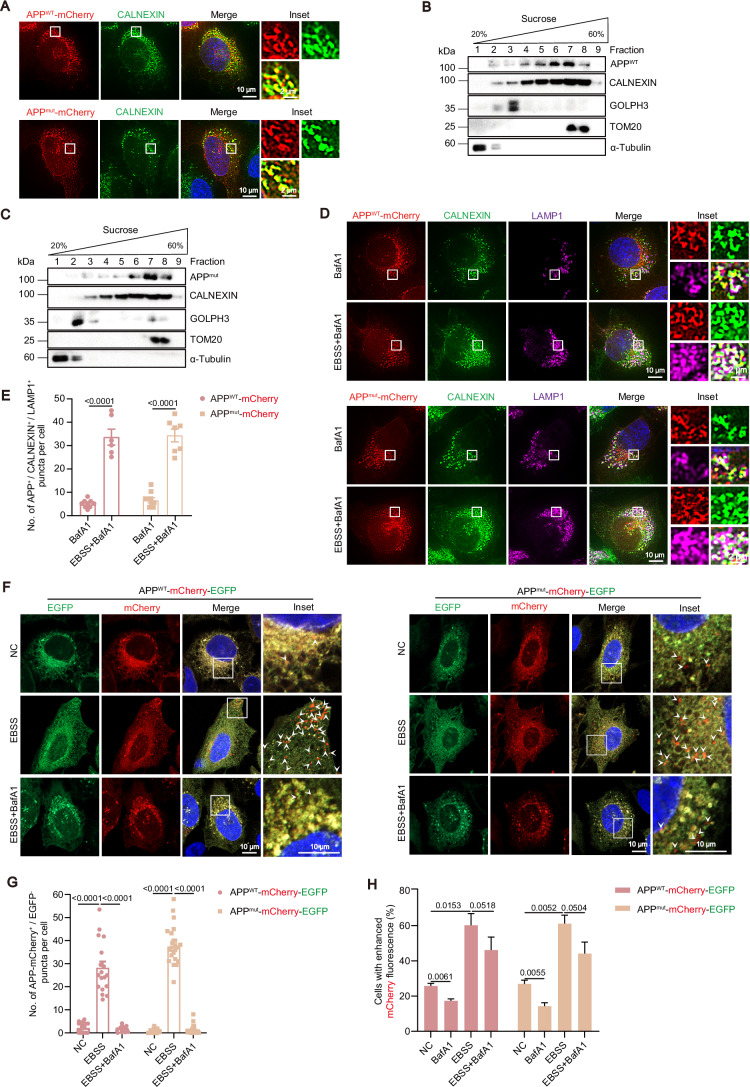
ER-localized APP is degraded through the autophagosome-lysosome pathway, related to Fig. 2. (**A**) Multi-SIM images showing colocalization of APP^WT/mut^-mCherry with CALNEXIN. U2OS cells expressing DOX-inducible APP^WT/mut^-mCherry were cultured in nutrient-rich medium. CALNEXIN was detected by immunostaining. (**B**, **C**) Subcellular fractionation of U2OS cells expressing DOX-inducible APP^WT^ (**B**) or APP^mut^ (**C**). Cell lysates were fractionated on a sucrose gradient. CALNEXIN, ER marker; GOLPH3, Golgi marker; TOM20, mitochondrial marker; α-Tubulin, cytosolic marker. (**D**) Multi-SIM images showing colocalization of APP with CALNEXIN and LAMP1. U2OS cells expressing DOX-inducible APP^WT/mut^-mCherry were treated with BafA1 or EBSS + BafA1 for 6 h. CALNEXIN and LAMP1 were detected by immunostaining. (**E**) Quantification of APP^+^/CALNEXIN^+^/LAMP1^+^ puncta in (**D**). *N* = 10 cells (each BafA1 group), *N* = 6 cells (APP^WT^-mCherry, EBSS + BafA1), *N* = 7 cells (APP^mut^-mCherry, EBSS + BafA1). (**F**) Live-cell confocal images of U2OS cells expressing DOX-inducible APP^WT/mut^-mCherry-EGFP, either untreated or treated with EBSS, or EBSS + BafA1 for 6 h. Arrowheads indicate mCherry^+^/EGFP^-^ puncta. (**G**) Quantification of mCherry^+^/EGFP^-^ puncta in (**F**). *N* = 17 cells (APP^WT^-mCherry-EGFP, NC); *N* = 25 cells (APP^mut^-mCherry-EGFP, NC); *N* = 18 cells (APP^WT^-mCherry-EGFP, EBSS); *N* = 25 cells (APP^mut^-mCherry-EGFP, EBSS); *N* = 20 cells (APP^WT^-mCherry-EGFP, EBSS + BafA1); *N* = 30 cells (APP^mut^-mCherry-EGFP, EBSS + BafA1). (**H**) Quantification of cells with an increased mCherry/EGFP ( − APP) ratio by flow cytometry to assess lysosomal delivery of APP. U2OS cells expressing DOX-inducible APP^WT/mut^-mCherry-EGFP were either untreated or treated with BafA1, EBSS, or EBSS + BafA1 for 6 h. *n* = 3. Error bars represent SEM; *P < 0.05, ****P < 0.0001; (**E**) was analyzed by unpaired Student’s *t* test; (**G**) was analyzed by one-way ANOVA; (**H**) was analyzed by two-way ANOVA.

**Figure 2:**
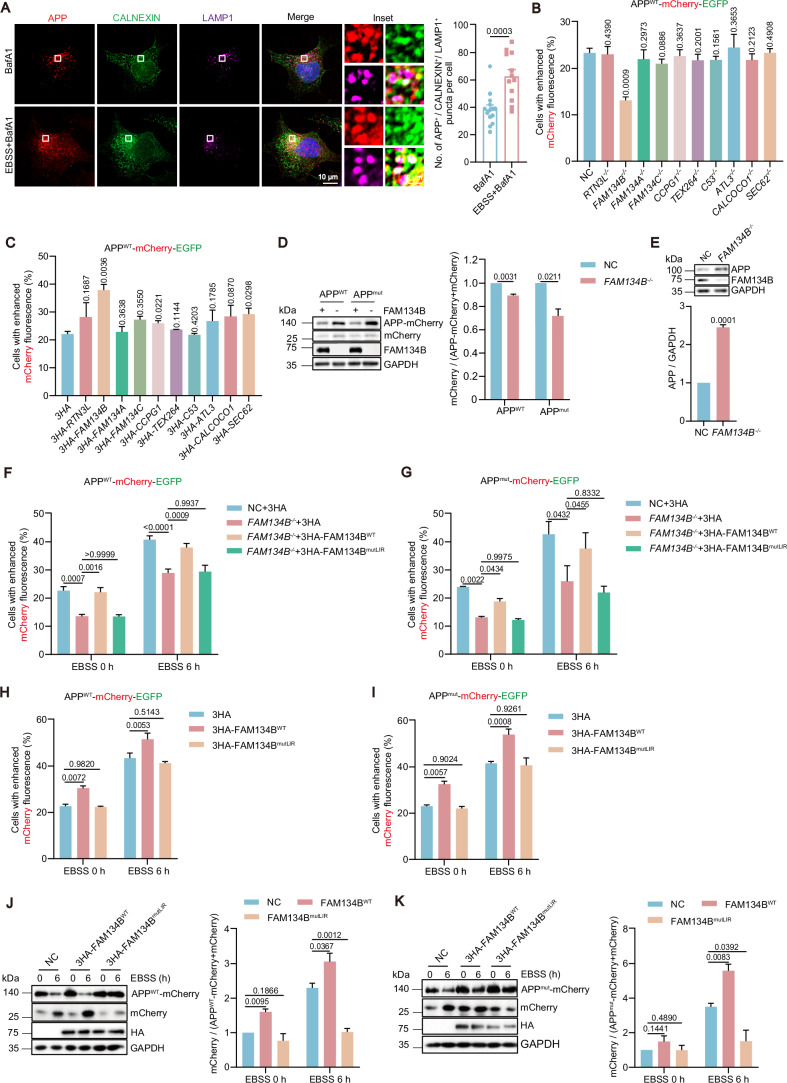
FAM134B selectively promotes APP degradation through ER-phagy. (**A**) Confocal images showing colocalization of APP with CALNEXIN and LAMP1. SH-SY5Y cells were treated with BafA1, or EBSS + BafA1 for 6 h. CALNEXIN, APP, and LAMP1 were detected by immunostaining. Right: Quantification of APP^+^/CALNEXIN^+^/LAMP1^+^ puncta. *N* = 14 cells (BafA1); *N* = 12 cells (EBSS + BafA1). (**B**) Quantification of cells exhibiting an increased mCherry/EGFP ( − APP^WT^) ratio by flow cytometry to assess lysosomal delivery of APP^WT^ in control and ER-phagy receptor–knockout U2OS cells cultured in nutrient-rich medium. U2OS cells stably expressing DOX-inducible APP^WT^-mCherry-EGFP were subjected to CRISPR-mediated knockout of the indicated receptor. *n* = 3. (**C**) Quantification of lysosomal delivery of APP^WT^-mCherry-EGFP in control and ER-phagy receptor–overexpressing U2OS cells cultured in nutrient-rich medium. U2OS cells stably expressing DOX-inducible APP^WT^-mCherry-EGFP were transiently transfected with plasmids encoding the indicated receptors. *n* = 3 for all conditions except 3HA, 3HA–RTN3L, and 3HA–CALCOCO1 (*n* = 5). (**D**) Immunoblotting of APP^WT/mut^-mCherry cleavage into free mCherry in WT and *FAM134B*-knockout U2OS cells cultured in nutrient-rich medium. *FAM134B* was knocked out in U2OS cells expressing DOX-inducible APP^WT/mut^-mCherry. Right: Quantification of the ratio of free mCherry to APP^WT/mut^-mCherry. *n* = 3. (**E**) Immunoblotting of protein levels in WT and *FAM134B*-knockout SH-SY5Y cells cultured in nutrient-rich medium. Bottom: Quantification of protein levels shown above. *n* = 4. (**F**, **G**) Quantification of lysosomal delivery of APP in U2OS cells expressing DOX-inducible APP^WT^-mCherry-EGFP (**F**, *n* = 4) or APP^mut^-mCherry-EGFP (**G**, *n* = 3). *FAM134B*-knockout cells were transiently transfected with empty vector (3HA), WT FAM134B (3HA-FAM134B^WT^), or LIR-mutant FAM134B (3HA-FAM134B^mutLIR^) to evaluate rescue effect. WT cells transfected with sgRNA control and empty vector (NC + 3HA) served as control. (**H**, **I**) Quantification of lysosomal delivery of APP in U2OS cells expressing DOX-inducible APP^WT^-mCherry-EGFP (**H**) or APP^mut^-mCherry-EGFP (**I**). Wild-type cells were transfected with empty vector, 3HA-FAM134B^WT^, or 3HA-FAM134B^mutLIR^ to evaluate the overexpression effect. *n* = 3. (**J**, **K**) Immunoblotting of APP^WT/mut^-mCherry cleavage into free mCherry. HEK293T cells expressing DOX-inducible APP^WT^-mCherry (**J**) or APP^mut^-mCherry (**K**) were transiently transfected with empty vector, 3HA-FAM134B^WT^, or 3HA-FAM134B^mutLIR^ to evaluate the overexpression effect. *n* = 3. Error bars represent SEM; ns, no significance, *P* > 0.05, *P < 0.05, **P < 0.01, ***P < 0.001, ****P < 0.0001; (**A**–**E**) were analyzed by unpaired Student’s *t* test; (**F**, **G**) were analyzed by two-way ANOVA; (**H**–**K**) were analyzed by one-way ANOVA. Source data are available online for this figure.

Given that APP undergoes ER-to-endosome trafficking upon synthesis (Placido et al, 2014), it is prone to accumulation in the ER, raising the possibility that ER-phagy may contribute to its clearance. To investigate this, we first examined the subcellular localization of APP. Multi-modal structured illumination microscopy (multi-SIM) imaging showed that overexpressed APP^WT^ and APP^mut^ colocalized with the ER marker CALNEXIN (Fig. EV3A). Subcellular fractionation showed that both APP^WT^ and APP^mut^ were enriched in the ER fraction (Fig. EV3B,C). Immunofluorescence imaging of SH-SY5Y cells showed the colocalization of endogenous APP with CALNEXIN (Fig. 2A). Under nutrient-rich conditions, limited CALNEXIN-APP puncta colocalized with the lysosomal marker LAMP1, whereas starvation markedly enhanced this overlap (Fig. 2A). Similar results were observed in APP^WT/mut^-overexpressing cells (Fig. EV3D,E), suggesting that ER-localized APP undergoes basal lysosomal delivery, which is markedly enhanced upon starvation.

To visualize and quantify lysosomal degradation of APP, we constructed cells expressing APP^WT/mut^-mCherry-EGFP. Live-cell confocal imaging showed that under nutrient-rich conditions APP^WT/mut^-mCherry-EGFP exhibited few mCherry^+^/EGFP^−^ puncta (Fig. EV3F). Starvation markedly increased the number of mCherry^+^/EGFP^−^ puncta, and this effect was abolished by BafA1 (Fig. EV3F,G), indicating autophagy-dependent lysosomal degradation. Flow cytometry analysis confirmed a significant increase in the mCherry/EGFP ratio under starvation conditions, which was inhibited by BafA1 (Fig. EV3H). These results demonstrate that APP accumulates in the ER and is targeted for lysosomal degradation through ER-phagy.

### FAM134B specifically promotes ER-phagy-mediated degradation of APP in an LIR-dependent manner

**Figure EV4:**
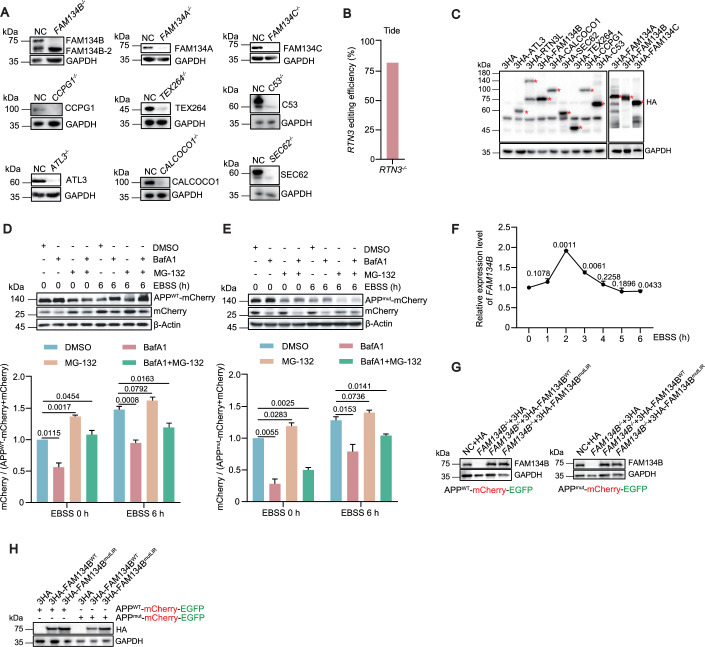
APP is degraded through the lysosomal pathway, related to Fig. 2. (**A**) Immunoblotting to validate the CRISPR-Cas9 knockout efficiency of ER-phagy receptors. (**B**) TIDE (Tracking of Indels by DEcomposition) analysis showing an 82.3% total editing efficiency for *RTN3* knockout. TIDE was used in place of immunoblotting because an RTN3L-specific antibody is unavailable. (**C**) Immunoblotting to validate the overexpression efficiency of HA-tagged ER-phagy receptors. The asterisks mark the correct band position of the target protein. (**D**, **E**) Immunoblotting of APP^WT/mut^-mCherry cleavage into free mCherry. HEK293T cells expressing DOX-inducible APP^WT^-mCherry (**D**) or APP^mut^-mCherry (**E**) were treated as indicated. *n* = 4. (**F**) qRT-PCR analysis of *FAM134B* mRNA levels in U2OS cells, either untreated or treated with EBSS for the indicated times. *n* = 3. (**G**, **H**) Immunoblotting to validate the expression levels of 3HA-tagged FAM134B^WT^ and FAM134B^mutLIR^. Error bars represent SEM; ns, no significance, *P* > 0.05, *P < 0.05, **P < 0.01, ***P < 0.001; (**D**, **E**) were analyzed by two-way ANOVA; (**F**) was analyzed by unpaired Student’s *t* test.

To identify which ER-phagy receptors are required for APP degradation, we generated individual knockout cell lines of known ER-phagy receptors in cells expressing APP^WT^-mCherry-EGFP (Fig. EV4A,B). Flow cytometry revealed that *FAM134B* knockout cells showed a significant decrease in the mCherry/EGFP ratio compared to control cells, whereas knockout of other receptors had no effect on the mCherry/EGFP ratio (Fig. 2B). By contrast, when these receptors were overexpressed in APP^WT^-mCherry-EGFP cells, only *FAM134B* overexpression led to a significant increase in the mCherry/EGFP ratio compared to control cells (Figs. 2C and EV4C). The sgRNA used for *FAM134B* knockout specifically targets the longest FAM134B isoform, and immunoblotting confirmed that the N-terminally truncated isoform FAM134B-2 was not affected (Fig. EV4A). Similarly, overexpression experiments were performed using the longest FAM134B isoform (Fig. EV4C). These results suggest that among the ER-phagy receptors tested, FAM134B is specifically required for the delivery of APP to the lysosome.

To validate FAM134B’s role in APP degradation, we conducted an APP^WT/mut^-mCherry cleavage assay. Since mCherry is resistant to lysosomal proteases, the delivery of APP^WT/mut^-mCherry to the lysosome leads to the release of free mCherry. Immunoblotting showed an increase in free mCherry when APP^WT/mut^-mCherry cells were shifted from nutrient-rich to starvation conditions (Fig. EV4D,E). This cleavage was inhibited by BafA1, but not by the proteasome inhibitor MG-132 (Fig. EV4D,E), supporting a lysosome-dependent degradation pathway. Notably, MG-132 has been reported to stimulate autophagy (Lan et al, 2015); consistent with this, under nutrient-rich conditions MG-132 alone promoted lysosomal cleavage of APP^WT/mut^-mCherry rather than blocking it (Fig. EV4D,E). Further, immunoblotting and quantification showed that cleavage of APP^WT/mut^-mCherry to free mCherry was significantly decreased in *FAM134B*-knockout cells compared to control cells (Fig. 2D). To extend our findings from overexpressed APP to the endogenous context, we knocked out *FAM134B* in SH-SY5Y cells and observed a marked accumulation of endogenous APP (Fig. 2E), indicating that FAM134B is required for endogenous APP degradation. These data collectively suggest that FAM134B is required for APP degradation via ER-phagy.

To determine whether starvation activates FAM134B-mediated ER-phagy, we examined *FAM134B* transcription following EBSS treatment. qRT-PCR analysis showed that EBSS significantly increased *FAM134B* mRNA levels (Fig. EV4F), indicating transcriptional activation. In contrast, *FAM134B* transcription was suppressed in APP-overexpressing cells (Fig. 1I), suggesting that starvation counteracts this inhibition, consistent with enhanced FAM134B-mediated ER-phagy of APP under starvation conditions.

Receptors direct specific substrates to phagophores by binding to LC3 proteins through their LC3-interacting regions (LIRs) (Lamark and Johansen, 2021). We then tested whether FAM134B’s LIR is required for targeting APP to the phagophore. Flow cytometry showed that re-expression of wild-type FAM134B (FAM134B^WT^) rescued the decreased mCherry/EGFP ratio in *FAM134B*-knockout APP^WT/mut^-mCherry-EGFP-expressing cells, restoring it to levels similar to those in control cells. By contrast, LIR-mutant FAM134B (Khaminets et al, 2015) (FAM134B^mutLIR^) failed to rescue the mCherry/EGFP ratio (Figs. 2F,G and EV4G). Flow cytometry further showed that when FAM134B^WT^ was overexpressed in APP^WT/mut^-mCherry-EGFP cells, it significantly increased the mCherry/EGFP ratio, indicating enhanced delivery of APP to the lysosome. By contrast, overexpression of FAM134B^mutLIR^ failed to do so (Figs. 2H,I and EV4H). Immunoblotting analysis of the APP^WT/mut^-mCherry cleavage also obtained similar results (Fig. 2J,K). These results collectively suggest that FAM134B promotes ER-phagy degradation of APP through an LIR-dependent mechanism.

### FAM134B targets ER-localized APP to phagophores via C-terminal interaction

**Figure 3:**
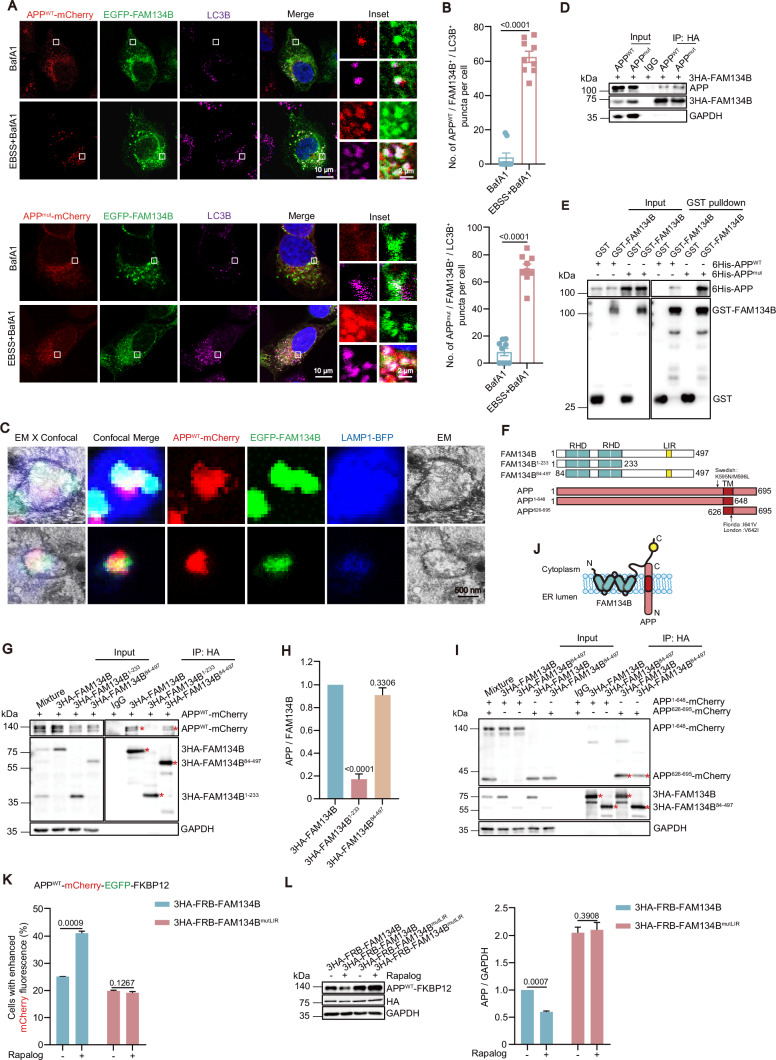
C-terminal interaction with FAM134B promotes APP degradation via ER-phagy. (**A**) Confocal images showing colocalization of APP^WT/mut^-mCherry with EGFP-FAM134B and LC3B. U2OS cells expressing DOX-inducible APP^WT/mut^-mCherry were transiently transfected with EGFP-FAM134B, then treated with BafA1 or EBSS + BafA1 for 6 h. LC3B was detected by immunostaining. (**B**) Quantification of APP^WT/mut^-mCherry^+^/EGFP-FAM134B^+^/LC3B^+^ puncta in (**A**). *N* = 9 cells. (**C**) Correlative light and electron microscopy (CLEM) images showing colocalization of EGFP-FAM134B, APP^WT^-mCherry, and LAMP1-BFP. U2OS cells expressing DOX-inducible APP^WT^-mCherry were transiently transfected with EGFP-FAM134B and LAMP1-BFP, then treated with EBSS + BafA1 for 6 h. Confocal and EM overlay images reveal triple-positive puncta colocalizing with autolysosomal structures. (**D**) Co-IP of APP^WT/mut^ with FAM134B. HEK293T cells expressing DOX-inducible APP^WT/mut^ were transiently transfected with 3HA-FAM134B; cell lysates were subjected to IP using anti-HA antibodies. IgG immunoprecipitation of mixed APP^WT^ and APP^mut^ lysates served as a negative control. (**E**) Pulldown assay showing in vitro binding of GST-FAM134B with 6His-APP^WT/mut^. Recombinant 6His-APP^WT/mut^ purified from *E. coli* was incubated with GST-FAM134B or GST (negative control) immobilized on GST-affinity beads. (**F**) Schematic of full-length and truncated FAM134B and APP constructs. Domains shown include RHD (reticulon homology domain), LIR (LC3-interacting region), and TM (transmembrane domain); APP695 with the Swedish (K670N/M671L), Florida (I716V), and London (V717I) mutations (APP770 numbering; corresponding to APP695 K595N/M596L, I641V, and V642I). (**G**) Co-IP of APP^WT^ with full-length and truncated 3HA-tagged FAM134B. HEK293T cells were transiently co-transfected with APP^WT^-mCherry and different FAM134B constructs, followed by immunoprecipitation with anti-HA antibodies. IgG pulldown of mixed FAM134B lysates served as a negative control. (**H**) Quantification of APP^WT^-mCherry immunoprecipitated by FAM134B in (**G**). *n* = 4. (**I**) Co-IP of full-length and truncated APP with full-length and truncated 3HA-tagged FAM134B. HEK293T cells were transiently co-transfected with the indicated APP and FAM134B constructs, followed by immunoprecipitation with anti-HA antibodies. (**J**) Schematic of FAM134B and APP binding via their C termini on the ER membrane. (**K**) Quantification of cells with an increased mCherry/EGFP ( − APP) ratio by flow cytometry to assess lysosomal delivery of APP. U2OS cells expressing DOX-inducible APP^WT^-mCherry-EGFP-FKBP were transiently transfected with FRB-FAM134B or FRB-FAM134B^mutLIR^, either untreated or treated with Rapalog. *n* = 3. (**L**) Immunoblotting of APP-FKBP protein levels. U2OS cells expressing DOX-inducible APP^WT^-FKBP were transiently transfected with FRB-FAM134B or FRB-FAM134B^mutLIR^, either untreated or treated with Rapalog. *n* = 3. Error bars represent SEM; ns, no significance, *P* > 0.05, ***P < 0.001, ****P < 0.0001; (**B**, **K**, **L**) were analyzed by unpaired Student’s *t* test; (**H**) was analyzed by one-way ANOVA. Source data are available online for this figure.

**Figure EV5:**
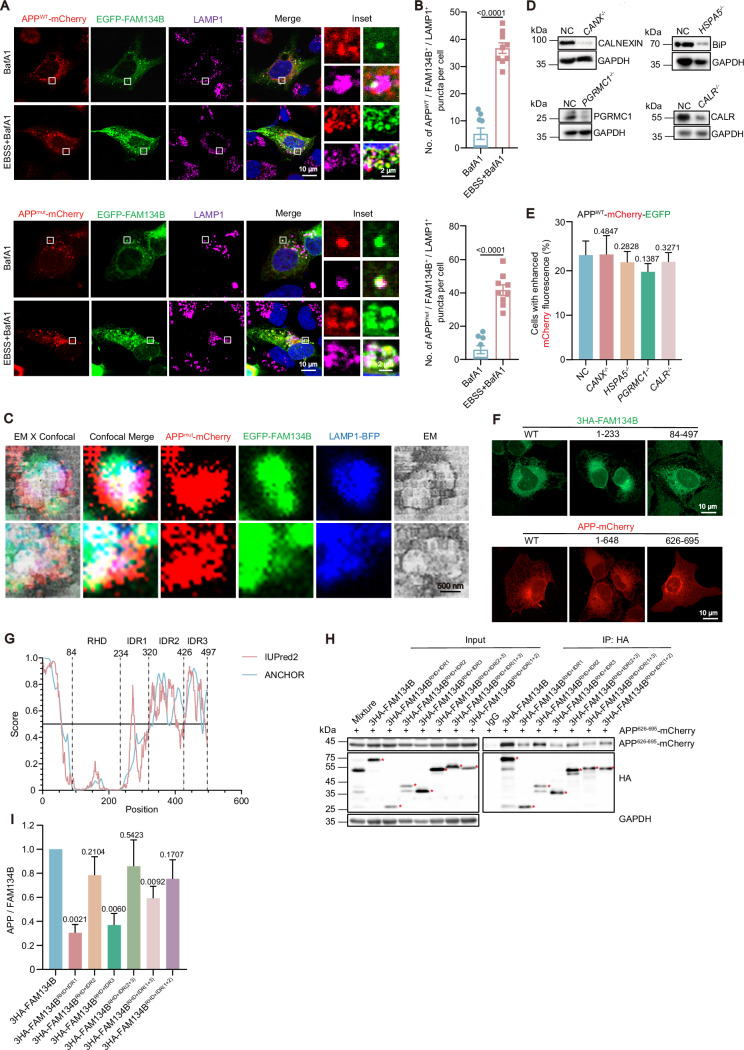
ER-localized APP is degraded by FAM134B-mediated ER-phagy, related to Fig. 3. (**A**) Confocal images showing colocalization of APP^WT/mut^-mCherry with EGFP-FAM134B and LAMP1. U2OS cells expressing DOX-inducible APP^WT/mut^-mCherry were transiently transfected with EGFP-FAM134B and treated with BafA1 or EBSS + BafA1 for 6 h. LAMP1 was detected by immunostaining. (**B**) Quantification of APP^WT/mut^-mCherry^+^/EGFP-FAM134B^+^/LAMP1^+^ puncta in (**A**). *N* = 10 cells. (**C**) CLEM images showing colocalization of EGFP-FAM134B, APP^mut^-mCherry, and LAMP1-BFP. U2OS cells expressing DOX-inducible APP^mut^-mCherry were transiently transfected with EGFP-FAM134B and LAMP1-BFP, then treated with EBSS + BafA1 for 6 h. Confocal and EM overlay images reveal triple-positive puncta colocalizing with autolysosomal structures. (**D**) Immunoblotting to validate the CRISPR-Cas9 knockout efficiency of ER chaperones. (**E**) Quantification of cells with an increased mCherry/EGFP (-APP) ratio by flow cytometry to assess lysosomal delivery of APP^WT^. WT, *CANX* (CALNEXIN)-, *HSPA5* (BiP)-, *PGRMC1*-, and *CALR* (Calreticulin)-knockout U2OS cells expressing DOX-inducible APP^WT^-mCherry-EGFP were cultured in nutrient-rich medium. *n* = 3. (**F**) Confocal images showing the subcellular localization of full-length and truncated 3HA-FAM134B and APP-mCherry. FAM134B was detected using anti-HA antibodies. (**G**) Predicted IDR structure of the C-terminus of FAM134B using IUPred2A. Three major IDRs were identified: IDR1 (aa 234–320), IDR2 (aa 321–426), and IDR3 (aa 427–497). IUPred2 (red) predicts IDRs (score > 0.5), while ANCHOR (blue) identifies binding-competent subregions within IDRs that fold upon protein interaction. (**H**) Co-IP of APP^626–695^–mCherry with IDR-truncated FAM134B to determine which IDR mediates binding to the APP C-terminus. Lysates were prepared from HEK293T cells co-transfected with APP^626–695^–mCherry and the indicated FAM134B constructs. The asterisks mark the correct band position of the target protein. (**I**) Quantification of APP co-immunoprecipitated with FAM134B in (**H**). *n* = 3. Error bars represent SEM; ns, no significance, *P* > 0.05, *P < 0.05, **P < 0.01, ****P < 0.0001; (**B**, **E**, **I**) were analyzed by unpaired Student’s *t* test.

We next investigated how FAM134B targets APP to the phagophore for ER-phagy degradation. Confocal imaging showed that EGFP-FAM134B colocalized with APP^WT/mut^-mCherry and the phagophore marker LC3B, with colocalization increasing under starvation (Fig. 3A,B). EGFP-FAM134B also colocalized with APP^WT/mut^-mCherry and LAMP1, again enhanced by starvation (Fig. EV5A,B). Correlative light and electron microscopy (CLEM) confirmed that triple-positive puncta (mCherry-APPW^T/mut+^/EGFP-FAM134B^+^/LAMP1-BFP^+^) localized within autolysosomes (Figs. 3C and EV5C). Together, these findings demonstrate that FAM134B escorts APP to the phagophore and promotes its delivery to autophagosomes and autolysosomes for degradation.

Lacking ER luminal domains, FAM134B depends on ER chaperones to bridge its interaction with substrates during ER-phagy. For instance, it binds CALNEXIN to mediate degradation of procollagen (Forrester et al, 2019) and transmembrane protein TrkB (Luningschror et al, 2023), and interacts with BiP to target substrates under hypoxic conditions (Chipurupalli et al, 2022). We therefore asked whether ER chaperones are involved in FAM134B-mediated ER-phagy of APP. Flow cytometry analysis showed that knockout of CALNEXIN or BiP did not change the mCherry/EGFP(-APP^WT^) ratio compared to control cells (Fig. EV5D,E). Notably, knockout of additional ER chaperones—including PGRMC1, which bridges RTN3L and its ER-phagy substrates (Chen et al, 2021b), as well as calreticulin (CALR), which binds APP (Johnson et al, 2001)—also had no effect on APP delivery to lysosomes (Fig. EV5D,E). These results indicate that the tested ER chaperones are not required for FAM134B-mediated ER-phagy of APP.

Like FAM134B, APP localizes to the ER membrane, raising the possibility that it is directly recognized by FAM134B. Co-IP experiments showed that both APP^WT^ and APP^mut^ interact with FAM134B (Fig. 3D). An in vitro pulldown assay using purified recombinant proteins demonstrated a direct interaction between APP^WT/mut^ and FAM134B (Fig. 3E). We next sought to map the interaction domains. FAM134B consists of a short N-terminal region (aa 1–83), two reticulon homology domains (RHDs; aa 84–233), and a cytosolic C-terminal region containing a LIR motif (aa 234–497) (Khaminets et al, 2015) (Fig. 3F). To preserve ER localization, we generated two FAM134B truncation mutants: FAM134B^1–233^ and FAM134B^84–497^, both retaining the RHDs. Confocal imaging showed that both mutants exhibited a typical ER network-like distribution, similar to wild-type FAM134B (Fig. EV5F). Co-IP results showed that FAM134B^1–233^ displayed a markedly reduced interaction with APP, indicating that the N-terminal region and RHDs alone are insufficient for binding. By contrast, FAM134B^84–497^ retained APP-binding capacity comparable to wild-type FAM134B (Fig. 3G,H), suggesting that the C-terminal region of FAM134B is essential for APP binding.

APP is a type I transmembrane protein with an N-terminal ectodomain (aa 1–625), a transmembrane domain (aa 626–648), and a short cytosolic C-terminal tail (aa 649–695) (Zhang et al, 2023) (Fig. 3F). To assess which domain mediates FAM134B binding while preserving ER localization, we generated two APP truncations: APP^1–648^ and APP^626–695^, both retaining the transmembrane domain. Confocal imaging showed that both mutants exhibited an ER network-like distribution, similar to wild-type APP (Fig. EV5F). Co-IP experiments revealed that FAM134B interaction was markedly reduced with APP^1–648^ but retained with APP^626–695^. Both full-length FAM134B and FAM134B^84–497^ bound to APP^626–695^ (Fig. 3I), suggesting that the C-terminal region (aa 649–695) of APP is essential for FAM134B binding. Notably, the familial AD mutations (K595N/M596L, I641V, and V642I) are located outside this interaction region (Fig. 3F), consistent with FAM134B recognizing APP independently of familial mutations and mediating the degradation of both wild-type and mutant APP.

Given that the C-terminal region of FAM134B is intrinsically disordered, we next sought to define which intrinsically disordered region (IDR) mediates APP binding. IUPred2A (Meszaros et al, 2018) analysis identified three major IDRs within the FAM134B C terminus: IDR1 (aa 234–320), IDR2 (aa 321–426), and IDR3 (aa 427–497) (Fig. EV5G). We therefore generated truncation mutants retaining only one IDR or lacking individual IDRs while preserving the RHD domains. Co-IP assays showed that APP binding was largely maintained when only IDR2 was present but markedly reduced upon deletion of IDR2, indicating that the three IDRs act cooperatively with IDR2 providing the primary contribution (Fig. EV5H,I). Together, these results demonstrate that FAM134B recognizes ER-localized APP through multivalent interactions between their C-terminal domains (Fig. 3J).

### Induced APP-FAM134B dimerization enhances APP degradation via ER-phagy

**Figure EV6:**
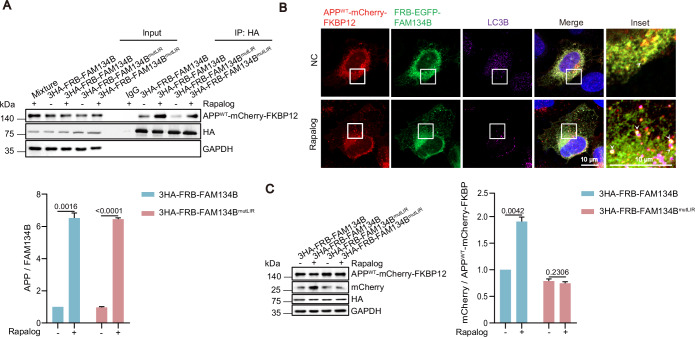
Induced APP-FAM134B dimerization enhances APP degradation via ER-phagy, related to Fig. 3. (**A**) Co-IP of APP^WT^ with FAM134B or FAM134B^mutLIR^. Lysates were prepared from U2OS cells expressing DOX-inducible APP^WT^-mCherry-FKBP12 and transiently transfected with 3HA-FRB-FAM134B or 3HA-FRB-FAM134B^mutLIR^, either untreated or treated with Rapalog. Bottom: Quantification of APP co-immunoprecipitated with FAM134B. *n* = 3. (**B**) Confocal images showing colocalization of APP^WT^ with FAM134B and LC3B. U2OS cells expressing DOX-inducible APP^WT^-mCherry-FKBP12 and transiently transfected with FRB-EGFP-FAM134B, either untreated or treated with Rapalog. LC3B was detected by immunostaining. (**C**) Immunoblotting of APP^WT^-mCherry-FKBP12 cleavage into free mCherry. U2OS cells expressing DOX-inducible APP^WT^-mCherry-FKBP12 transiently transfected with 3HA-FRB-FAM134B or 3HA-FRB-FAM134B^mutLIR^, either untreated or treated with Rapalog. *n* = 3. Error bars represent SEM; ns, no significance, *P* > 0.05, **P < 0.01, ***P < 0.001, ****P < 0.0001; (**A**, **C**) were analyzed by one-way ANOVA.

We next tested whether enhancing the interaction between APP and FAM134B could promote APP degradation using a chemically inducible dimerization system (Grumati et al, 2017b). The FRB domain (T2098L mutant of the FKBP12-rapamycin-binding domain of mTOR) was fused to the N-terminus of wild-type FAM134B or its LIR mutant, while human FKBP12 was fused to the C-terminus of APP^WT^. Co-IP showed that Rapalog-induced FKBP-FRB dimerization significantly enhanced the interaction between APP and both FAM134B and FAM134B^mutLIR^ (Fig. EV6A), suggesting that this system can be used to test the functional consequences of APP-FAM134B dimerization. Confocal imaging revealed that APP-mCherry-FKBP and FRB-EGFP-FAM134B colocalized within an ER-like network, and Rapalog treatment markedly increased the number of APP-mCherry⁺/EGFP-FAM134B⁺ puncta colocalizing with LC3B (Fig. EV6B), indicating that induced APP-FAM134B dimerization promotes recruitment of APP to the phagophore or autophagosome.

Flow cytometry analysis showed that Rapalog treatment significantly increased the mCherry/EGFP ratio in APP^WT^-mCherry-EGFP-FKBP cells co-expressing FRB-FAM134B, but not in those expressing FRB-FAM134B^mutLIR^ (Fig. 3K). Immunoblotting and quantification confirmed that Rapalog-induced APP degradation occurred only in APP-FKBP cells co-expressing FRB-FAM134B, but not in those with FRB-FAM134B^mutLIR^ (Fig. 3L). Consistent results were obtained from the mCherry cleavage assay in APP-mCherry-FKBP cells (Fig. EV6C). Together, these findings demonstrate that enforced APP-FAM134B interaction promotes APP degradation via ER-phagy in an LIR-dependent manner.

### Epigenetic repression of *FAM134B* limits TFEB/TFE3 binding and suppresses its transcription in AD

**Figure 4:**
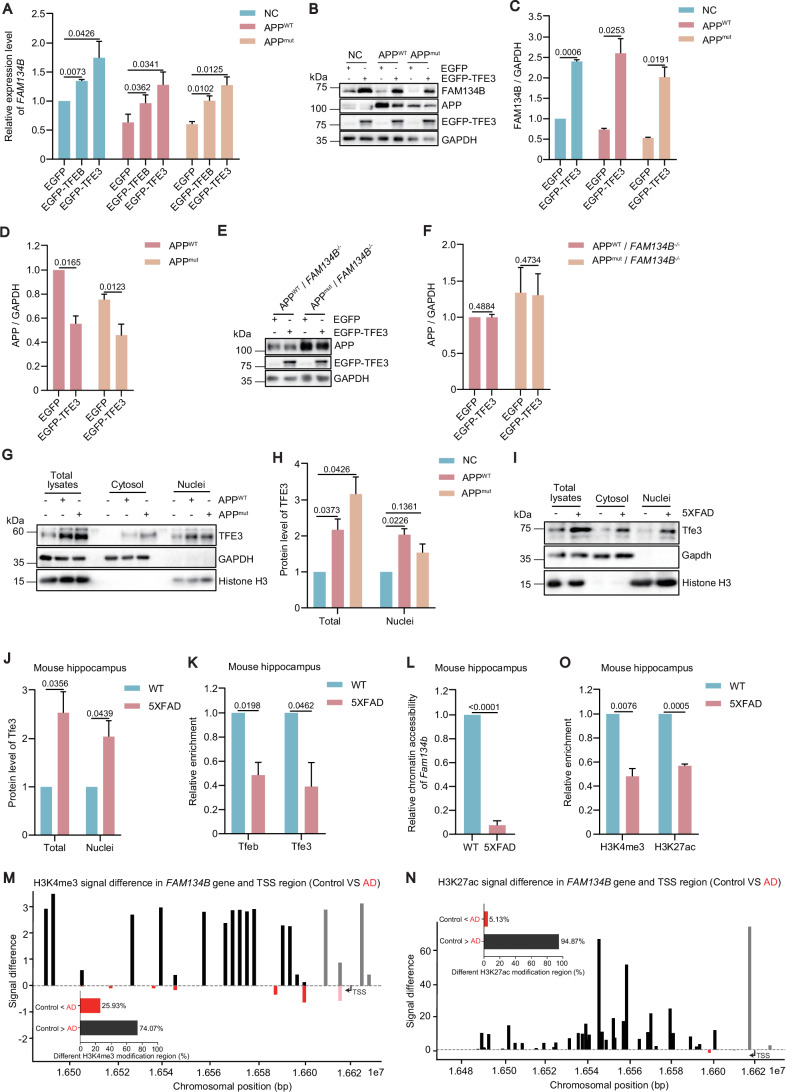
Epigenetic repression limits TFEB/TFE3 binding to the *FAM134B* promoter, leading to its transcriptional downregulation in AD. (**A**) qRT-PCR analysis of *FAM134B* mRNA levels in control and U2OS cells expressing DOX-inducible APP^WT/mut^. Cells were transiently transfected with EGFP, EGFP-TFEB, or EGFP-TFE3 plasmids. *n* = 3. (**B**) Immunoblotting of FAM134B and APP protein levels in control and U2OS cells expressing DOX-inducible APP^WT/mut^. Cells were transiently transfected with EGFP or EGFP-TFE3 plasmids. (**C**, **D**) Quantification of relative levels of FAM134B (**C**) and APP (**D**) in (**B**). *n* = 3. (**E**) Immunoblotting of APP protein levels in *FAM134B*-knockout U2OS cells expressing DOX-inducible APP^WT/mut^. Cells were transiently transfected with EGFP or EGFP-TFE3 plasmids. (**F**) Quantification of relative levels of APP in (**E**). *n* = 3. (**G**) Nuclear–cytosolic fractionation analysis of TFE3 localization in control and U2OS cells expressing DOX-inducible APP^WT/mut^. GAPDH: cytosolic marker; histone H3: nuclear marker. (**H**) Quantification of relative levels of total and nuclear TFE3 in (**G**). *n* = 3. (**I**) Nuclear–cytosolic fractionation analysis of Tfe3 localization in the hippocampus of WT and 5XFAD mice. (**J**) Quantification of relative levels of total and nuclear Tfe3 in (**I**). *n* = 3. (**K**) ChIP-qPCR assay showing enrichment of Tfeb and Tfe3 at the *Fam134b* promoter in hippocampal samples from WT and 5XFAD mice. ChIP was performed with Tfeb and Tfe3 antibodies, followed by quantitative PCR analysis of the *Fam134b* promoter. *n* = 3. (**L**) ATAC-seq analysis of the *Fam134b* promoter in the hippocampal samples from WT and 5XFAD mice. *n* = 4. (**M**, **N**) Comparative analysis of H3K4me3 (**M**) and H3K27ac (**N**) enrichment at the *FAM134B* gene body and TSS region ( ± 10 kb) in controls and AD patients. ChIP-seq datasets for H3K4me3 (GSE196413) and H3K27ac (GSE130746) were obtained from GEO. Signal differences between controls (black/gray) and AD patients (red/pink) are shown. Inset bar plots show the proportion of regions with increased (control >AD) or decreased (control < AD) histone modification signals in AD. (**O**) ChIP-qPCR assay showing enrichment of H3K4me3 and H3K27ac at the *Fam134b* promoter in the hippocampus of WT and 5XFAD mice. ChIP was performed with H3K4me3 and H3K27ac antibodies, followed by quantitative PCR analysis of the *Fam134b* promoter. *n* = 3. Error bars represent SEM; ns, no significance, *P* > 0.05, *P < 0.05, **P < 0.01, ***P < 0.001, ****P < 0.0001; (**A**, **H**) were analyzed by one-way ANOVA; (**C**, **D**, **F**, **J**–**L**, **O**) were analyzed by unpaired Student’s *t* test. Source data are available online for this figure.

**Figure EV7:**
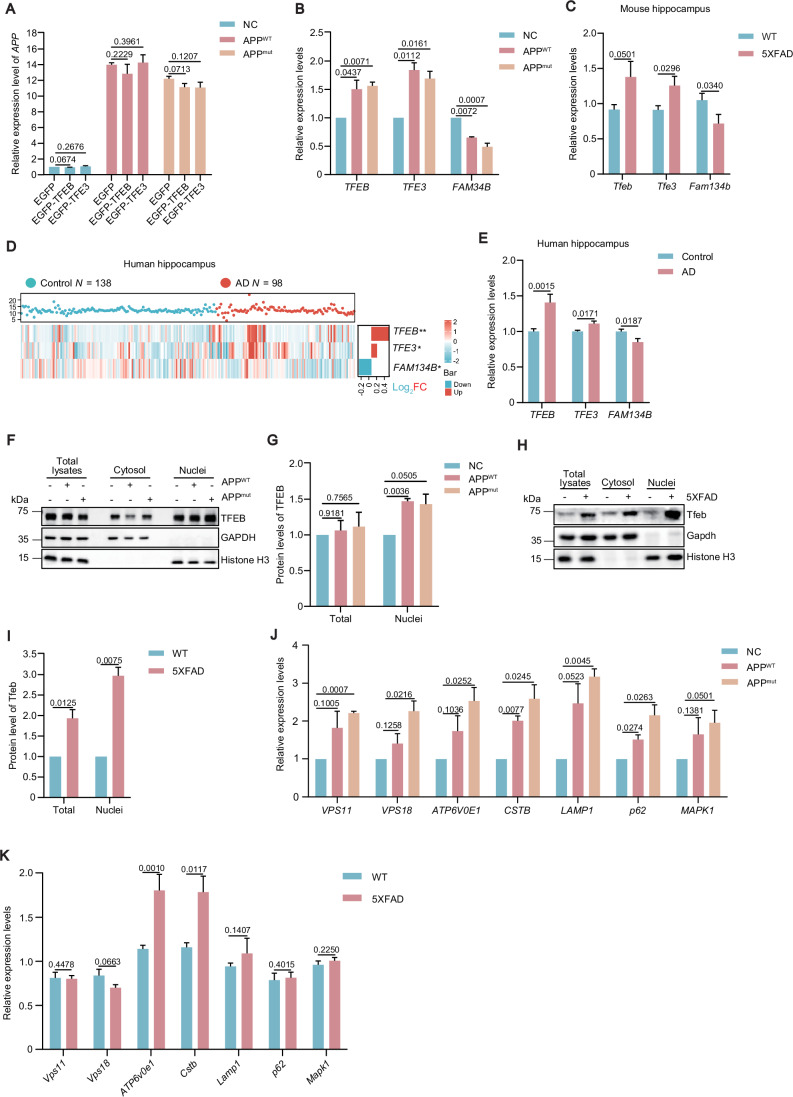
Nuclear translocation of TFEB/TFE3 is increased in AD, related to Fig. 4. (**A**) qRT-PCR analysis of *APP* mRNA levels in control and U2OS cells expressing DOX-inducible APP^WT/mut^ and transiently transfected with EGFP, EGFP-TFEB, or EGFP-TFE3 plasmids. *n* = 3. (**B**) qRT-PCR analysis of *TFEB*, *TFE3*, and *FAM134B* mRNA levels in control and U2OS cells expressing DOX-inducible APP^WT/mut^. *n* = 3. (**C**) qRT-PCR analysis of *Tfeb*, *Tfe3*, and *Fam134b* mRNA levels in the hippocampus of 6-month-old WT and 5XFAD mice (*N* = 3 per group). (**D**, **E**) Cross-database normalized microarray and RNA-seq analysis of *TFEB*, *TFE3*, and *FAM134B* mRNA levels in the hippocampus of AD patients (*N* = 98) and non-AD controls (*N* = 138). *P* values were adjusted according to the Benjamini–Hochberg false discovery rate (FDR) correction. (**F**) Nuclear–cytosolic fractionation analysis of TFEB localization in control and U2OS cells expressing DOX-inducible APP^WT/mut^. GAPDH: cytosolic marker; histone H3: nuclear marker. (**G**) Quantification of relative levels of total and nuclear TFEB in (**F**). *n* = 3. (**H**) Nuclear–cytosolic fractionation analysis of Tfeb localization in the hippocampus of WT and 5XFAD mice. (**I**) Quantification of relative levels of total and nuclear Tfeb in (**H**). *n* = 3. **(J)** qRT-PCR analysis of mRNA levels of TFEB/TFE3 downstream target genes in control and U2OS cells expressing DOX-inducible APP^WT/mut^. *n* = 3. **(K)** qRT-PCR analysis of mRNA levels of Tfeb/Tfe3 downstream target genes in the hippocampus of 6-month-old WT and 5XFAD mice (*N* = 3 per group). Error bars represent SEM; ns, no significance, *P* > 0.05, *P < 0.05, **P < 0.01, ***P < 0.001; (**A**, **B**, **G**, **J**) were analyzed by one-way ANOVA; (**C**, **E**, **I**, **K**) were analyzed by unpaired Student’s *t* test.

Given that FAM134B directly targets APP for degradation via ER-phagy, its reduced transcription in AD may contribute to APP accumulation and disease progression. We therefore investigated the mechanisms underlying FAM134B downregulation in AD. The transcription factors TFEB and TFE3, master regulators of lysosome biogenesis and autophagy, upregulate *FAM134B* transcription under starvation and FGF (fibroblast growth factor) stimulation (Cinque et al, 2020). Upon nuclear translocation, TFEB and TFE3 activate target genes by binding to the CLEAR element (Raben and Puertollano, 2016). qRT-PCR showed that TFEB or TFE3 overexpression significantly increased *FAM134B* mRNA levels in both control and APP^WT/mut^-expressing cells (Fig. 4A). Immunoblotting and quantification showed increased FAM134B protein levels upon TFE3 overexpression (Fig. 4B,C). Although *APP* mRNA levels remained unchanged (Fig. EV7A), APP protein levels decreased (Fig. 4B,D). This decrease was abolished in *FAM134B*-knockout cells (Fig. 4E,F), suggesting that TFE3 promotes APP degradation via ER-phagy by upregulating FAM134B.

We next investigated whether reduced expression or impaired nuclear translocation of TFEB and TFE3 contributes to the downregulation of FAM134B transcription in AD. qRT-PCR revealed increased *TFEB* and *TFE3* mRNA levels in APP^WT/mut^-expressing cells and in the hippocampus of 5XFAD mice (Fig. EV7B,C). Analysis of the published microarray and RNA-seq datasets used in Fig. 1A also revealed increased *TFEB* and *TFE3* transcription in the hippocampus of AD patients (Fig. EV7D,E). Subcellular fractionation demonstrated increased total and nuclear TFEB and TFE3 protein levels in both APP^WT/mut^-expressing cells and the hippocampus of 5XFAD mice (Figs. 4G–J and EV7F–I). Further, qRT-PCR revealed increased mRNA levels of known TFE3 and TFEB target genes, such as *ATP6V0E1*, *CSTB*, and *LAMP1*, in both APP^WT/mut^-expressing cells and the hippocampus of 5XFAD mice (Fig. EV7J,K). These findings collectively indicate that overall TFEB and TFE3 transcriptional activity is increased in AD, suggesting that reduced *FAM134B* transcription likely arises from other regulatory mechanisms.

**Figure EV8:**
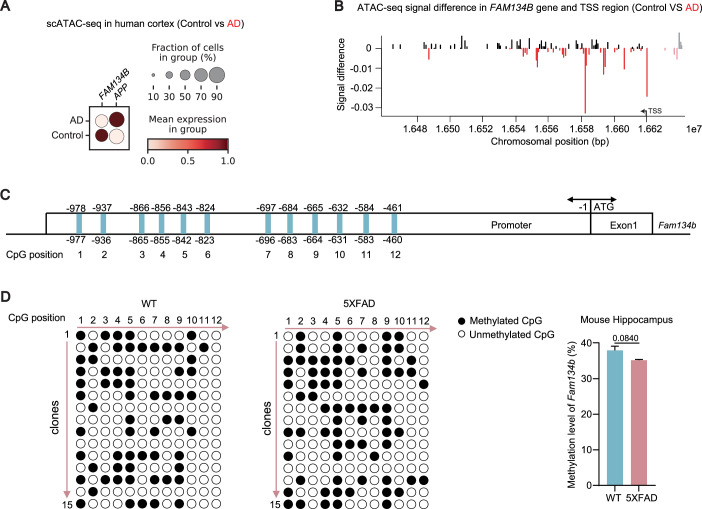
Chromatin accessibility at the FAM134B promoter is reduced, while DNA methylation remains unchanged in AD, related to Fig. 4. (**A**) Analysis of a published single-cell ATAC-seq (scATAC-seq) dataset (syn52293424) from cortex samples revealed a significant decrease in both the proportion of cells with accessible chromatin and the average accessibility at the *FAM134B* locus in AD patients (*N* = 15) compared to controls (*N* = 48). By contrast, chromatin accessibility at the *APP* locus was increased in AD samples. (**B**) scATAC-seq signal tracks showing chromatin accessibility at the *FAM134B* gene body and TSS region ( ± 10 kb) in cortex samples from controls and AD patients. All signals are normalized to reads per million (RPM). (**C**) Schematic of the 5′ region of the mouse *Fam134b* gene, showing the promoter, first exon, and translation start site (ATG). The nucleotide upstream of the ATG is designated as position –1. Twelve analyzed CpG sites are mapped relative to the ATG (e.g., –978/–977, –937/–936) and numbered 1–12. (**D**) Bisulfite sequencing PCR (BSP) analysis of CpG methylation patterns in the *Fam134b* promoter region in the hippocampus of WT and 5XFAD mice (*N* = 3 per group). In total, 15 randomly selected clones were analyzed for each sample. Error bars represent SEM; ns, no significance; unpaired Student’s *t* test.

Chromatin immunoprecipitation followed by qPCR (ChIP-qPCR) in 5XFAD mice revealed significantly reduced binding of TFE3 and TFEB to the CLEAR element within the *FAM134B* promoter compared to controls (Fig. 4K). Based on this, we hypothesized that epigenetic changes may underlie the decreased transcription factor binding. Analysis of published human single-cell ATAC-seq (assay for transposase-accessible chromatin using sequencing) data revealed decreased chromatin accessibility at the *FAM134B* gene body (including exons and introns) and its transcription start site (TSS) region in AD patients (Fig. EV8A,B). This was confirmed by ATAC-seq of the hippocampus from 5XFAD mice (Fig. 4L). Analysis of published human epigenetic sequencing data further revealed lower levels of active transcription marks of histone modification, H3K4me3 (GSE196413) and H3K27ac (GSE102538), across the *FAM134B* gene body and its TSS region in AD patients (Fig. 4M,N). ChIP-qPCR in the hippocampus of 5XFAD mice similarly demonstrated decreased enrichment of these marks (Fig. 4O). By contrast, DNA methylation at the *Fam134b* promoter remained unchanged in 5XFAD mice (Fig. EV8C,D). Together, these findings suggest that reduced *FAM134B* transcription in AD is driven by epigenetic reprogramming, including decreased chromatin accessibility and reduced activating histone modifications, rather than changes in DNA methylation, resulting in impaired TFE3 and TFEB binding.

### FAM134B overexpression ameliorates pathology in 5XFAD mice

**Figure 5:**
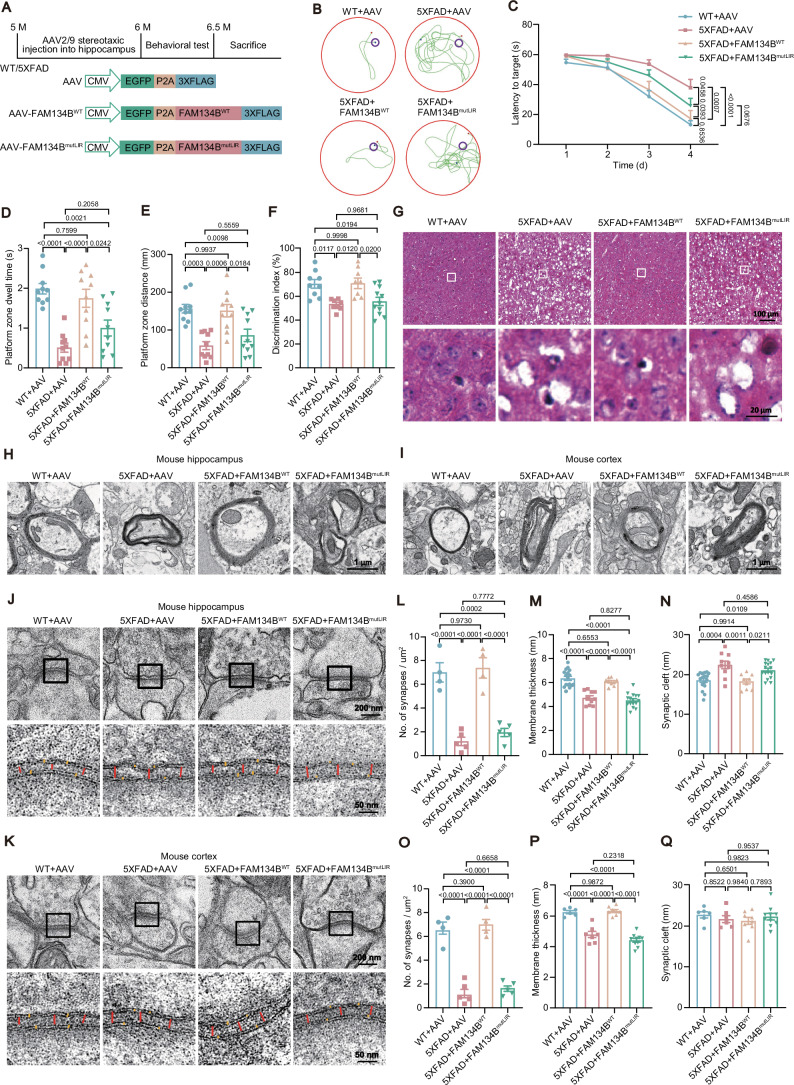
Upregulating *FAM134B* protects against cognitive and synaptic deficits in the 5XFAD mouse model. (**A**) Experimental timeline for AAV injection and behavior test in female mice. AAVs expressing FAM134B^WT^-EGFP, FAM134B^mutLIR^-EGFP, or EGFP control were stereotactically injected into the hippocampus of 5XFAD mice (5XFAD + FAM134B^WT^, 5XFAD + FAM134B^mutLIR^, 5XFAD + AAV), and control AAV was injected into WT mice (WT + AAV). (**B**) Morris water maze test. Representative swimming paths during the probe trial. (**C**) Latency to reach the hidden platform was recorded during the 4-day training (*N* = 10 per group). (**D**) Time spent in the platform zone during the 4-day Morris water maze test (*N* = 10 per group). (**E**) Distance traveled in the platform zone during the 4-day Morris water maze test (*N* = 10 per group). (**F**) Discrimination index in the novel object recognition test. WT + AAV (*N* = 9); 5XFAD + AAV (*N* = 8); 5XFAD + FAM134B^WT^ (*N* = 8); 5XFAD + FAM134B^mutLIR^ (*N* = 11). (**G**) H&E-stained images of the cortex. (**H**, **I**) TEM images showing myelin sheath structure. (**J**, **K**) TEM images showing synapse structure. The orange line represents the thickness of the synaptic membrane, and the red line represents the synaptic cleft. (**L**–**Q**) Quantification of synapse number, membrane thickness, and synaptic cleft width based on TEM images in (**J**, **K**). WT + AAV (*N* = 4); 5XFAD + AAV (*N* = 5); 5XFAD + FAM134B^WT^ (*N* = 4); 5XFAD + FAM134B^mutLIR^ (*N* = 5) in L and O. WT + AAV (*n* = 17 fields); 5XFAD + AAV (*n* = 10 fields); 5XFAD + FAM134B^WT^ (*n* = 9 fields); 5XFAD + FAM134B^mutLIR^ (*n* = 16 fields) in M and N. WT + AAV (*n* = 6 fields); 5XFAD + AAV (*n* = 7 fields); 5XFAD + FAM134B^WT^ (*n* = 8 fields); 5XFAD + FAM134B^mutLIR^ (*n* = 10 fields) in (**P**, **Q**). Error bars represent SEM; ns, no significance, *P* > 0.05, *P < 0.05, **P < 0.01, ***P < 0.001, ****P < 0.0001; (**C**–**F**, **L**–**Q**) were analyzed by two-way ANOVA. Source data are available online for this figure.

**Figure EV9:**
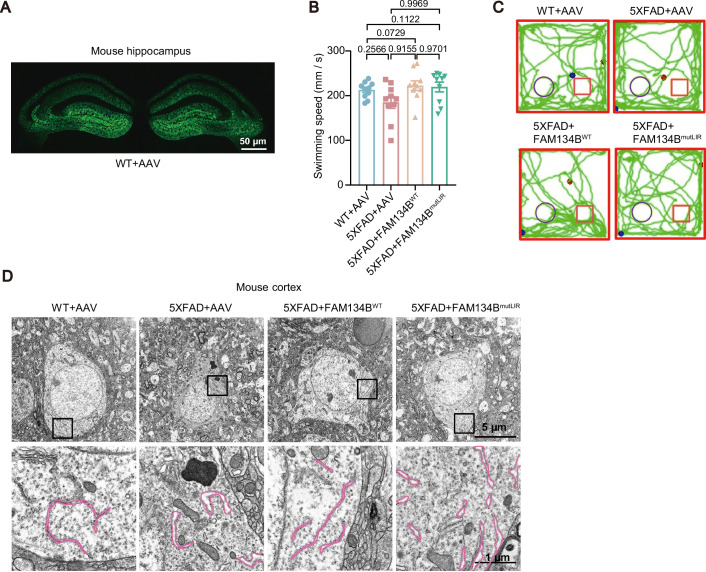
Upregulation of FAM134B protects against AD-related pathology in the 5XFAD mouse model, related to Figs. 5 and 6. (**A**) Representative images showing hippocampus-specific expression of AAV-delivered EGFP or EGFP-tagged fusion proteins following stereotactic brain injection. (**B**) Swimming speed during the 4-day Morris water maze test (*N* = 10 per group). WT + AAV; 5XFAD + AAV; 5XFAD + FAM134B^WT^; 5XFAD + FAM134B^mutLIR^. Error bars represent SEM; ns, no significance, *P* > 0.05; two-way ANOVA test. (**C**) Representative paths in the novel object recognition test. Blue circle indicates the novel object. (**D**) Representative TEM images of cortical neurons. ER membranes are outlined in magenta.

To investigate the therapeutic potential of FAM134B upregulation, we generated AAV2/9 vectors expressing EGFP-FAM134B^WT^ or EGFP-FAM134B^mutLIR^ under the control of the CMV promoter. These constructs were bilaterally injected into the hippocampus of 5-month-old 5XFAD female mice, yielding the 5XFAD + FAM134B^WT^ and 5XFAD + FAM134B^mutLIR^ groups. Control groups included 5XFAD and WT mice injected with an empty vector (5XFAD + AAV and WT + AAV, respectively) (Fig. 5A). Confocal imaging confirmed robust and region-specific EGFP expression in the hippocampus (Fig. EV9A). One month after injection, mice underwent behavior testing followed by sacrifice for subsequent analysis of the cortex and hippocampus (Fig. 5A).

The Morris water maze test showed that, compared with vector-injected 5XFAD mice, those injected with AAV-FAM134B^WT^, but not AAV-FAM134B^mutLIR^, exhibited improved spatial learning and memory. This was evidenced by significantly reduced path length and escape latency during acquisition trials, as well as increased platform crossings and time spent in the target quadrant during probe trials (Fig. 5B–E). Notably, swimming speed was comparable across groups, excluding motor impairments (Fig. EV9B). Further, 5XFAD mice injected with AAV-FAM134B^WT^ also showed improved performance in novel object recognition tests compared to vector- or AAV-FAM134B^mutLIR^-injected 5XFAD mice (Figs. 5F and EV9C). These findings demonstrate that FAM134B overexpression significantly improves learning and memory in AD mice, and that this effect is dependent on the LIR of FAM134B.

Hematoxylin and eosin (H&E) staining revealed neuronal abnormalities in 5XFAD mice injected with vector or AAV-FAM134B^mutLIR^, characterized by disorganized arrangement, nuclear shrinkage, and intense staining. By contrast, 5XFAD mice injected with AAV-FAM134B^WT^ exhibited preserved neuronal morphology with distinct cell borders and clear nuclei, comparable to age-matched vector-injected WT controls (Fig. 5G), suggesting that FAM134B overexpression rescues neuronal histopathological defects in 5XFAD mice. Myelin abnormalities are recognized contributors to cognitive decline in AD (Hirschfeld et al, 2022). Transmission electron microscopy (TEM) analysis showed abnormal myelin sheath morphology in both the hippocampus and cortex of 5XFAD mice injected with vector or AAV-FAM134B^mutLIR^ (Fig. 5H, I). Remarkably, myelin morphology was preserved in 5XFAD mice injected with AAV-FAM134B^WT^, similar to WT controls (Fig. 5H,I), demonstrating that FAM134B overexpression ameliorates myelin abnormalities in 5XFAD mice.

Synapse loss and degeneration are also major contributors of AD progression (Griffiths and Grant, 2023). TEM analysis and quantification revealed significant synaptic defects in the hippocampus and cortex of 5XFAD mice, including reduced synapse numbers and decreased synaptic membrane thickness (Fig. 5J–M,K–P). These defects were markedly rescued by AAV-FAM134B^WT^ injection (Fig. 5J–M,K–P). Notably, an increased synaptic cleft width was observed in the hippocampus, but not the cortex, of 5XFAD mice (Fig. 5J,N,K,Q). This hippocampal defect was also significantly rescued by AAV-FAM134B^WT^ (Fig. 5J,N). These data suggest that FAM134B overexpression rescues the synapse abnormalities in 5XFAD mice. Collectively, these findings demonstrate that FAM134B overexpression exerts a neuroprotective effect and improves cognitive function in the 5XFAD mouse model of AD.

### Enhancing FAM134B-mediated ER-phagy promotes APP degradation and decreases amyloid plaque deposition in 5XFAD mice

**Figure 6:**
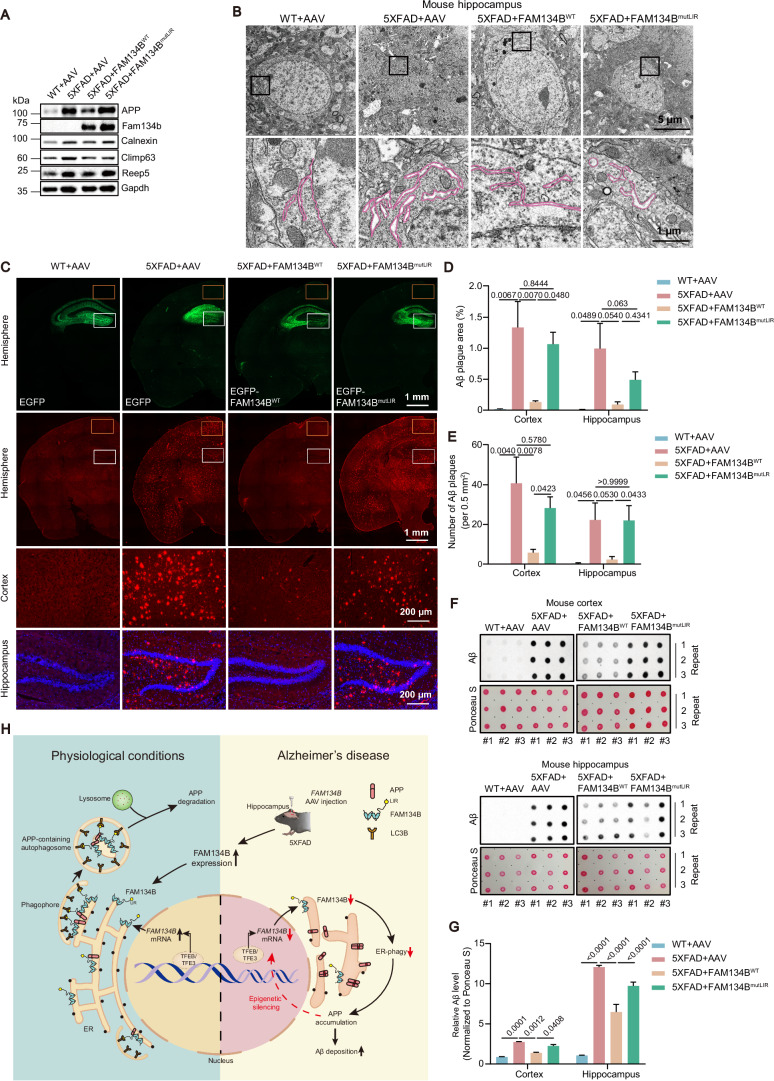
ER-phagy enhancement promotes APP degradation and reduces amyloid plaque deposition in 5XFAD mice. (**A**) Immunoblotting of protein levels in the hippocampus. (**B**) TEM images showing ER structure in hippocampal neurons. ER membranes are outlined in magenta. (**C**) Confocal images of mouse brains stereotactically injected into the hippocampus with AAVs expressing EGFP-FAM134B^WT^, EGFP-FAM134B^mutLIR^, or EGFP control. Immunostaining for Aβ (red) and nuclei (DAPI, blue) was performed. EGFP signals indicate predominant transduction in the hippocampus. WT + AAV (*N* = 3); 5XFAD + AAV (*N* = 4); 5XFAD + FAM134B^WT^ (*N* = 4); 5XFAD + FAM134B^mutLIR^ (*N* = 4). (**D**, **E**) Quantification of amyloid plaque deposition area and amyloid plaque number in (**C**). (**F**) Dot blot of insoluble Aβ in the cortex and hippocampus. Ponceau S staining served as a loading control. (**G**) Quantification of the relative Aβ levels in (**F**). WT + AAV was set to 1 for normalization. *n* = 3. (**H**) Schematic model: FAM134B-mediated ER-phagy degrades APP and mitigates AD pathology. Under physiological conditions, TFEB/TFE3 bind to the *FAM134B* promoter and induce its expression. FAM134B interacts with APP on the ER membrane via their C-terminal regions, recruits LC3B via its LIR motif, and mediates APP degradation through ER-phagy. In AD, epigenetic repression (reduced H3K27ac, H3K4me3, and chromatin accessibility) suppresses TFEB/TFE3 binding, leading to *FAM134B* downregulation, impaired ER-phagy, and APP accumulation, forming a feed-forward pathogenic loop that exacerbates Aβ deposition and disease progression. AAV-mediated hippocampal delivery of FAM134B restores ER-phagy, promotes APP degradation, and confers neuroprotection in 5XFAD mice. Error bars represent SEM; ns, no significance, *P* > 0.05, *P < 0.05, **P < 0.01, ***P < 0.001, ****P < 0.0001; (**D**, **E**, **G**) were analyzed by two-way ANOVA. Source data are available online for this figure.

We next investigated whether the observed neuroprotective effects were mediated by restored ER-phagy and enhanced APP degradation. Immunoblotting revealed markedly lower APP levels in 5XFAD mice injected with AAV-FAM134B^WT^ compared to those injected with the vector (Fig. 6A). Concurrently, levels of ER-resident proteins (Calnexin, Climp63, and Reep5) were reduced in AAV-FAM134B^WT^-injected mice, suggesting restoration of ER-phagy in these animals (Fig. 6A). TEM analysis revealed extensive dilated and fragmented ER structures in neurons of 5XFAD mice injected with the vector or AAV-FAM134B^mutLIR^ in both the hippocampus and cortex (Figs. 6B and EV9D). These ER abnormalities resemble those characteristic of ER-phagy deficiency previously observed in *Drosophila* expressing human APP and in *FAM134B-*knockdown mammalian cells (Khaminets et al, 2015; Mou et al, 2024). By contrast, 5XFAD mice injected with AAV-FAM134B^WT^ exhibited normal ER morphology, similar to WT controls (Figs. 6B and EV9D). These findings suggest that AAV-mediated FAM134B expression rescues ER-phagy and preserves ER morphology in 5XFAD mice.

Given that Aβ originates from APP cleavage, we assessed amyloid plaque deposition in these mice. Immunofluorescence staining revealed a significant reduction in Aβ levels in the hippocampus and cortex of 5XFAD mice injected with AAV-FAM134B^WT^ compared with vector or AAV-FAM134B^mutLIR^, reaching levels comparable to WT controls (Fig. 6C–E). Dot blot analysis with Aβ-specific antibodies further confirmed a marked decrease in total Aβ levels in the hippocampus and cortex of AAV-FAM134B^WT^-injected mice (Fig. 6F,G). Notably, AAV delivery predominantly transduced hippocampal cells with limited spread to other brain regions (Fig. 6C), yet broader phenotypic improvements were observed, consistent with secondary effects of enhanced hippocampal APP clearance on downstream neural circuits. These findings demonstrate that AAV-mediated FAM134B overexpression restores ER-phagy in 5XFAD mice, leading to enhanced APP degradation, reduced Aβ accumulation, and improved synaptic integrity and cognitive performance, supporting its potential as a therapeutic strategy for AD.

## Discussion

Here we define a role for FAM134B-mediated ER-phagy in the turnover of APP and its contribution to amyloid pathology (Fig. 6H). FAM134B interacts with APP at the ER membrane and targets it for lysosomal degradation through an LIR-dependent ER-phagy pathway. In AD, both FAM134B expression and ER-phagy flux are reduced. Despite elevated nuclear TFEB/TFE3, epigenetic constraints at the *FAM134B* promoter, including altered histone modifications and reduced chromatin accessibility, restrict transcriptional activation. Restoring FAM134B, but not its LIR mutant, ameliorates amyloid accumulation and behavioral deficits in 5XFAD mice. Together, these results position impaired FAM134B-mediated ER-phagy as a contributing mechanism to APP accumulation in AD and suggest that enhancing ER-phagy may represent a strategy to limit amyloidogenic substrate availability. This is particularly relevant given that increased APP dosage itself is pathogenic independent of extracellular amyloid deposition.

Consistent with this concept, genetic studies have shown that APP gene dosage alone can drive AD-related pathology (Rovelet-Lecrux et al, 2006; Wiseman et al, 2015). Transgenic expression of wild-type human APP induces synaptic dysfunction, tau pathology and neurodegeneration even in the absence of detectable amyloid deposition (Simon et al, 2009; Yamaguchi et al, 1991). Our findings identify FAM134B-mediated ER-phagy as a mechanism that degrades both wild-type and familial mutant APP. Constitutive turnover of APP may therefore serve to prevent accumulation of toxic APP-derived intermediates and maintain proteostatic control of the cellular APP pool. Despite their shared dependence on FAM134B-dependent ER-phagy, wild-type and familial mutant APP display subtle differences that suggest distinct engagement of cytosolic quality-control pathways. Wild-type APP overexpression reduces p62 levels, whereas mutant APP shows little effect, and the two forms respond differently to proteasome inhibition. These observations indicate differential accessibility to p62-mediated autophagy and potentially ER-associated degradation. Such variation may influence the relative pathogenicity of mutant APP despite their similar regulation by ER-phagy.

Among the ER-phagy receptors examined, APP degradation selectively depends on FAM134B. This specificity likely reflects both its sub-ER localization and its mode of substrate recognition. FAM134B resides at ER sheets, a region enriched in newly synthesized membrane proteins and therefore particularly sensitive to protein folding stress (Khaminets et al, 2015). Consistent with this environment, FAM134B has been implicated in the clearance of aggregation-prone substrates such as ATZ, procollagen, and α-synuclein (Forrester et al, 2019; Fregno et al, 2018; Kim et al, 2023). In addition, FAM134B directly interacts with APP through their C-terminal regions, supporting a selective recognition mechanism. Unlike previously described cases in which FAM134B cooperates with ER chaperones including CALNEXIN or BiP (Chipurupalli et al, 2022; Forrester et al, 2019; Luningschror et al, 2023), APP clearance occurs independently of chaperone engagement, indicating a distinct mode of substrate selection. Direct recognition is consistent with emerging evidence that ER-phagy receptors can act without obligate chaperone intermediates, as shown for CCPG1 substrates (Ishii et al, 2023).

Epigenetic dysregulation is increasingly recognized in neurodegenerative disorders (Berson et al, 2018). In AD patient samples and 5XFAD mice, reduced H3K4me3 and H3K27ac marks at the *FAM134B* locus coincide with decreased chromatin accessibility, limiting TFEB/TFE3-dependent transcriptional activation. This provides a potential explanation for previous observations that TFEB overexpression in 5XFAD mice fails to substantially reduce APP levels or amyloid deposition (Polito et al, 2014), suggesting that activation of lysosomal regulators alone is insufficient when chromatin constraints prevent induction of specific target genes such as *FAM134B*. The upstream mechanisms driving these epigenetic alterations remain unclear. In parallel, FAM134B displays reduced ubiquitination and oligomerization in AD models, consistent with decreased receptor activity. Together, these transcriptional and post-translational defects impair ER-phagy, leading to accumulation of ER-resident proteins together with APP. Such impairment establishes a feed-forward cycle in which reduced FAM134B-dependent clearance further promotes APP accumulation and amyloidogenic processing.

Current AD therapies largely focus on eliminating toxic Aβ species, yet these approaches have achieved limited success (Huang, 2020). Our findings offer an alternative strategy by targeting the precursor of Aβ, APP, thereby potentially reducing Aβ production at its source. Future therapeutic strategies could include: (1) developing small molecules to enhance FAM134B transcription, which is suppressed in AD; (2) promoting FAM134B ubiquitination and oligomerization to boost its receptor activity, also impaired under AD conditions; and (3) designing molecular glues to enhance the APP-FAM134B interaction to promote APP degradation via ER-phagy, as supported by our FRB-FKBP tethering experiments. These approaches may promote the clearance of aggregation-prone proteins like APP via ER-phagy and suppress downstream Aβ generation, offering new avenues for AD treatment.

## Methods

**Table:** Reagents and tools table

Reagent/resource	Reference or source	Identifier or catalog number
**Experimental models**		
Brain tissue samples	The Emory Alzheimer’s Disease Research Center	
5XFAD mice (*M. musculus*)	Jackson Laboratory	RRID: MMRRC_034848-JAX B6.Cg-Tg (APPSwFlLon, PSEN1*M146L*L286V) 6799 Vas/Mmjax
SH-SY5Y cells	Procell Co. (Wuhan, China)	CL-0208
U2OS and HEK293T cells	A gift from Prof. Hongbing Shu, Wuhan University, China	
**Recombinant DNA**		
pcDNA3.1-3HA	A gift from Prof. Jingfeng Tang, Hubei University of Technology, China	
pcDNA3.1-3HA-FAM134B	Qian et al, 2023	
pcDNA3.1-3HA-FAM134B^mutLIR^	Qian et al, 2023	
pcDNA3.1-3HA-ATL3	Qian et al, 2023	
pcDNA3.1-3HA-RTN3L	Qian et al, 2023	
pcDNA3.1-3HA-CCPG1	Qian et al, 2023	
pcDNA3.1-3HA-TEX264	Constructed in-house	
pcDNA3.1-3HA-CALCOCO1	Qian et al, 2023	
pcDNA3.1-3HA-SEC62	Qian et al, 2023	
pcDNA3.1-3HA-C53	Constructed in-house	
pcDNA3.1-3HA-FAM134A	Qian et al, 2023	
pcDNA3.1-3HA-FAM134C	Qian et al, 2023	
pLVX-TetOne	A gift from Prof. Junjie Zhang, Wuhan University, China	
pLVX-TetOne-APP^WT^	Constructed in-house	
pLVX-TetOne -APP^mut^	Constructed in-house	
pLVX-TetOne-APP^WT^-mCherry	Constructed in-house	
pLVX-TetOne-APP^mut^-mCherry	Constructed in-house	
pLVX-TetOne-APP^WT^-mCherry-EGFP	Constructed in-house	
pLVX-TetOne-APP^mut^-mCherry-EGFP	Constructed in-house	
EGFP-FAM134B	Constructed in-house	
pCW57-CMV-ssRFP-EGFP-KDEL	A gift from Prof. Jingfeng Tang, Hubei University of Technology, China	
LAMP1-BFP	A gift from Prof. Yueguang Rong, Huazhong University of Science and Technology, China	
mCherry-EGFP-RAMP4	Constructed in-house	
GST-pGEX-4T1	A gift from Prof. Jingfeng Tang, Hubei University of Technology, China	
GST-FAM134B-pGEX-4T1	Constructed in-house	
pET28a	Constructed in-house	
6HIS-APP^WT^-pET28a	Constructed in-house	
6HIS-APP^mut^-pET28a	Constructed in-house	
pCMV-Myc-APP^WT^-mCherry	Constructed in-house	
pCMV-Myc-APP^1-648^-mCherry	Constructed in-house	
pCMV-Myc-APP^626-695^-mCherry	Constructed in-house	
pCDNA3.1-3HA-FAM134B^1-233^	Constructed in-house	
pCDNA3.1-3HA-FAM134B^84-497^	Constructed in-house	
pCMV-Myc-APP^WT^-FKBP12	Constructed in-house	
pCMV-Myc-APP^WT^-mCherry-FKBP12	Constructed in-house	
pCMV-Myc-APP^WT^-mCherry-EGFP-FKBP12	Constructed in-house	
pCDNA3.1-3HA-FRB^T2098L^-FAM134B	Constructed in-house	
pCDNA3.1-3HA-FRB^T2098L^-EGFP-FAM134B	Constructed in-house	
lentiCRISPR v2	A gift from Prof. Junjie Zhang, Wuhan University, China	
lentiCRISPR v2 NC	A gift from Prof. Junjie Zhang, Wuhan University, China	
EGFP-TFEB	A gift from Prof. Wei Liu, Zhejiang University, China	
EGFP-TFE3	A gift from Prof. Wei Liu, Zhejiang University, China	
pCDNA3.1-3HA-FAM134B^RHD+IDR1^	Constructed in-house	
pCDNA3.1-3HA-FAM134B^RHD+IDR2^	Constructed in-house	
pCDNA3.1-3HA-FAM134B^RHD+IDR3^	Constructed in-house	
pCDNA3.1-3HA-FAM134B^RHD+IDR(2+3)^	Constructed in-house	
pCDNA3.1-3HA-FAM134B^RHD+IDR(1+3)^	Constructed in-house	
pCDNA3.1-3HA-FAM134B^RHD+IDR1(1+2)^	Constructed in-house	
**Antibodies**		
Rabbit polyclonal anti-FAM134B	Proteintech	Cat# 21537-1-AP, RRID:AB_2878879
Rabbit polyclonal anti-FAM134A	Proteintech	Cat# 24650-1-AP, RRID:AB_3085745
Rabbit polyclonal anti-FAM134C	Proteintech	Cat# 26330-1-AP, RRID:AB_3085859
Rabbit polyclonal anti-ATL3	Proteintech	Cat# 16921-1-AP, RRID:AB_2290228
Rabbit polyclonal anti-TEX264	Proteintech	Cat# 25858-1-AP, RRID:AB_2880272
Rabbit polyclonal anti-CCPG1	Proteintech	Cat# 13861-1-AP, RRID:AB_2074010
Rabbit polyclonal anti-CALCOCO1	Proteintech	Cat# 19843-1-AP, RRID:AB_10637265
Rabbit polyclonal anti-SEC62	Proteintech	Cat# 28693-1-AP, RRID:AB_3086078
Rabbit polyclonal anti-C53	Proteintech	Cat# 11007-1-AP, RRID:AB_2076869
Rabbit polyclonal anti-REEP5	Proteintech	Cat# 14643-1-AP, RRID:AB_2178440
Rabbit polyclonal anti-CLIMP63	Proteintech	Cat# 16686-1-AP, RRID:AB_2276275
Rabbit polyclonal anti-CALNEXIN	Proteintech	Cat# 10427-2-AP, RRID:AB_2069033
Mouse Monoclonal Anti-GAPDH	Proteintech	Cat# 60004-1-Ig, RRID:AB_2107436
LC3B (E5Q2K) mouse mAb	Cell Signaling Technology	Cat# 83506, RRID:AB_2800018
Rabbit polyclonal anti-p62/SQSTM1	Proteintech	Cat# 18420-1-AP, RRID:AB_10694431
anti-LAMP1 (human) antibody	DSHB	Cat# H4A3, RRID:AB_2296838
Mouse monoclonal anti-APP	Proteintech	Cat# 60342-1-Ig, RRID:AB_2881451
Rabbit monoclonal anti-beta amyloid	Thermo Fisher Scientific	Cat# 700254, RRID:AB_2532306
Mouse monoclonal anti-GOLPH3	Proteintech	Cat# 67777-1-Ig, RRID:AB_2918542
Rabbit polyclonal anti-TOM20	Proteintech	Cat# 11802-1-AP, RRID:AB_2207530
Mouse polyclonal anti-α-Tubulin	Sigma-Aldrich	Cat# T6199, RRID:AB_477583
Mouse monoclonal anti-HA	Proteintech	Cat# 66006-2-Ig, RRID:AB_2881490
Rabbit polyclonal anti-HA	Proteintech	Cat# 51064-2-AP, RRID:AB_11042321
Rabbit IgG	Proteintech	Cat# B900610, RRID:AB_3674206
Mouse IgG	Proteintech	Cat# B900620, RRID:AB_2883054
Mouse monoclonal anti-BiP (4F11)	Zenbio	Cat# 200310-4F11
Rabbit polyclonal anti-PGRMC1	Proteintech	Cat# 12990-1-AP, RRID:AB_2164342
Rabbit polyclonal anti-CALR	Proteintech	Cat# 10292-1-AP, RRID:AB_513777
Rabbit polyclonal anti-TFEB	Proteintech	Cat# 13372-1-AP, RRID:AB_2199611
Rabbit polyclonal anti-TFE3	Proteintech	Cat# 14480-1-AP, RRID:AB_2199587
Rabbit monoclonal anti-Acetyl-Histone H3-K27	ABclonal	Cat# A22264, RRID:AB_3698464
Rabbit monoclonal anti-TriMethyl-Histone H3-K4	Zenbio	Cat# 502357
Mouse monoclonal anti-Histone H3	Proteintech	Cat# 68345-1-Ig, RRID:AB_3086558
Rabbit polyclonal anti-GST	Proteintech	Cat# 10000-0-AP, RRID:AB_11042316
Mouse monoclonal anti-HIS	Proteintech	Cat# 66005-1-Ig, RRID:AB_11232599
Rabbit polyclonal anti-GFP	Proteintech	Cat# 50430-2-AP, RRID:AB_11042881
Rabbit polyclonal anti-mCherry	Proteintech	Cat# 26765-1-AP, RRID:AB_2876881
Rabbit monoclonal anti-ubiquitin	HUABIO	Cat# HA750165
Mouse monoclonal anti-beta amyloid 17-24	BioLegend	Cat# 800708, RRID:AB_2734547
Mouse monoclonal anti-beta amyloid	Covance	Cat# SIG-39320, RRID:AB_662798
PERK Rabbit pAb	ABclonal	ABclonal Cat# A18196, RRID:AB_2861973
Phospho-PERK-T982 Rabbit pAb	ABclonal	ABclonal Cat# AP0886, RRID:AB_2771413
Rabbit polyclonal anti-IRE1	Proteintech	Cat# 27528-1-AP, RRID:AB_2880899
Phospho-IRE1-S724 Rabbit pAb	ABclonal	ABclonal Cat# AP0878, RRID:AB_2771207
Rabbit polyclonal anti-ATF6	Proteintech	Cat# 24169-1-AP, RRID:AB_2876891
Rabbit polyclonal anti-AMFR	Proteintech	Cat# 16675-1-AP, RRID:AB_2226463
Rabbit monoclonal anti-FAM134B-pS151	Gift from Prof. Qiming Sun, Zhejiang University, China	
Rabbit monoclonal anti- FAM134B-AcK160	Gift from Prof. Qiming Sun, Zhejiang University, China	
Multi-rAb HRP-Goat Anti-Rabbit Recombinant Secondary Antibody(H + L)	Proteintech	Cat# RGAR001, RRID:AB_3073505
Multi-rAb HRP-Goat Anti-Mouse Recombinant Secondary Antibody (H + L)	Proteintech	Cat# RGAM001, RRID:AB_3068333
Alexa Fluor 488-conjugated anti-Rabbit IgG	Abcam	Cat# ab150077, RRID:AB_2630356
Alexa Fluor 647-conjugated Goat Anti-Mouse lgG(H + L)	ABclonal	Cat# A5059, RRID:AB_2768329
**Oligonucleotides and other sequence-based reagents**		
PCR primers	This study	Table EV2
qPCR primers	This study	Table EV2
ATAC-qPCR primers	This study	Table EV2
ChIP-qPCR primers	This study	Table EV2
gRNA for knock-out	This study	Table EV3
**Chemicals, enzymes and other reagents**		
TIANamp Genomic DNA Kit	TIANGEN	Cat# DP304
DNA Bisulfite Conversion Kit	Beyotime	Cat# D0068S
TIANprep Mini Plasmids Kit	TIANGEN	Cat# DP103
Endo-free Plasmid DNA Mini Kit II	Omega	Cat# D6950-02
Gel Extraction Kit	Omega	Cat# D2500-02
HiScript II Q Select RT SuperMix for qPCR	Vazyme	Cat# R233-01
2X MultiF Seamless Assembly Mix	ABclonal	Cat# RK21020
inNova II High-Fidelity DNA Polymerase	Innovagene	Cat# HP132-12
2×Taq Master Mix	Vazyme	Cat# P112-03
Puromycin	InvivoGen	Cat# ANT-PR-1
Blasticidin	InvivoGen	Cat# ANT-BL-05
Hygromycin B	Solarbio	Cat# H8080
Trizol	Thermo Fisher	Cat# 15596026
Triton-X-100	Sigma-Aldrich	Cat# V900502
Protein A/G beads	Biolinkedin	Cat# L1004A
Polybrene	Beyotime	Cat# C0351
GoldBand 3-color Regular Range Protein Marker (10-180 kDa)	Yeasen	Cat# 20351ES90
cOmplete EDTA-free	Sigma-Aldrich	Cat# 4693132001
Ponceau S	Sigma-Aldrich	Cat# P7170-1L
Paraformaldehyde	Sigma-Aldrich	Cat# P6148-500G
Sodium deoxycholate	Sangon	Cat# A600150-0050
Ammonium Persulfate	Sigma-Aldrich	Cat# A3678-100G
Glycine	Sigma-Aldrich	Cat# V900144-5KG
Tris-base	BioFroxx	Cat# 115KG001
SDS	BioFroxx	Cat# 3250GR500
TEMED	Aladdin	Cat# T105496-100ML
DTT	Sangon	Cat# A620058-0025
β-mercaptoethanol	Sigma-Aldrich	Cat# M3148-100ML
DMSO	Sangon	Cat# A600163-0250
PMSF	Sangon	Cat# 329-98-6
**Software**		
ImageJ	https://imagej.nih.gov/ij/	
GraphPad Prism 8	https://www.graphpad.com/	
R 4.2.0	https://www.r-project.org/	
Seurat 4.3.0.1	https://github.com/satijalab/seurat	
SingleCellExperiment 1.20.8	https://github.com/drisso/SingleCellExperiment	
GenomicRanges 1.50.0	https://github.com/Bioconductor/GenomicRanges	
ComplexHeatmap 2.13.1	https://bioconductor.org/packages/InteractiveComplexHeatmap/	
Limma 3.64.1	https://bioinf.wehi.edu.au/limma/	
edgeR 4.6.2	https://bioconductor.org/packages/edgeR	
ggplot2 3.5.2	https://github.com/tidyverse/ggplot2/	
Python 3.10.9	https://github.com/python/	
Loompy 3.0.7	https://github.com/linnarsson-lab/loompy	
Matplotlib 3.8.2	https://github.com/matplotlib/matplotlib	
Numpy 1.26.4	https://github.com/numpy/numpy.org	
h5py 3.8.0	https://www.h5py.org	
Pandas 2.1.4	https://github.com/pandas-dev/pandas	
Scanpy 1.10.4	https://github.com/scverse/scanpy	
Jupyterlab 3.6.6	https://github.com/jupyterlab/	
**Other**		
Hydrophobic PVDF membranes	Sigma-Aldrich	Cat# IPFL00010
Nitrocellulose Membrane	Sigma-Aldrich	Cat# HATF02500
Amicon® Ultra-15 Centrifugal Filter (10 kDa MWCO)	Sigma-Aldrich	Cat# UFC9010
Millipore 0.45 µm membrane filter	Sigma-Aldrich	Cat# HABG02500

### Human samples

Post-mortem samples were dissected from frozen brains of AD patients and age-matched control subjects from the Emory Alzheimer’s Disease Research Center. The study was approved by the Biospecimen Committee. AD was diagnosed according to the criteria of the Consortium to Establish a Registry for AD and the National Institute on Aging. The patients’ information is summarized in Table EV1.

### Animals

All mice were housed and bred at the Wuhan University Laboratory Animal Center. Animals were maintained on a 12-h light/dark cycle under standard laboratory conditions (19–22 °C, 50%–60% humidity), and had free access to food and water. All experiments were conducted in accordance with protocols approved by the Institutional Animal Care and Use Committee of Wuhan University. The 5XFAD mice were purchased from the Jackson Laboratory (RRID: MMRRC_034848-JAX, B6.Cg-Tg (APPSwFlLon, PSEN1*M146L*L286V) 6799 Vas/Mmjax). Heterozygous males and females were intercrossed, and genotyping of the offspring was performed using PCR. Primer sequences are provided in Table EV2. Female heterozygous mice were selected as the experimental (5XFAD) group, and their wild-type littermates served as controls (WT).

### Cell culture, transfection and chemicals

SH-SY5Y cells were purchased from Procell Co. (Wuhan, China) and were cultured with serum-containing medium (Procell, CM-0208). U2OS and HEK293T cells were cultured in DMEM (GIBCO, C11965500BT) supplemented with 10% FBS (GIBCO, 10270106) and 1% Penicillin-Streptomycin (Sangon Biotech, E607011-0100) in a 37 °C, 5% CO_2_ incubator. DNA constructs were transfected using Neofect^TM^ DNA transfection reagent (Neofect Biotech).

To induce gene expression in the TetOne system, U2OS cells stably expressing pLVX-TetOne-Puro-APP^WT/mut^ or pLVX-TetOne-Puro-APP^WT/mut^-mCherry or pLVX-TetOne-Puro-APP^WT/mut^-mCherry-EGFP were cultured with 12 μg/mL doxycycline (Aladdin, 564-25-0) for 24 h. For nutrient starvation, the cells were cultured in EBSS (GIBCO, 24010043) for 6 h. Cells were treated with 250 nM Bafilomycin A1 (BafA1, Sangon, A601116-0025) or 10 µM MG-132 (MedChemExpress, HY-13259).

### Plasmids

The details of the plasmids used in this study are provided in Reagents and tools table. The sequences of ER-phagy receptors were amplified from the cDNA of HEK293T cells and cloned into pcDNA3.1-3HA. To perform rescue experiments in FAM134B-knockout cells, synonymous mutations were introduced into the 246-265 nucleotide region (the sgRNA target site) of FAM134B and FAM134B^mutLIR^ using pcDNA3.1-3HA-FAM134B/FAM134B^mutLIR^ as templates for site-directed mutagenesis PCR. APP^WT^ (APP695) was amplified from cDNA of SH-SY5Y cells and cloned into pLVX-TetOne-Puro vector. We also generated APP^mut^ by introducing the K607N and M608L mutations into parental pLVX-TetOne-Puro-APP^WT^ plasmid. To produce the GST-FAM134B^WT/mutLIR^ and 6HIS-APP^WT/mut^ fusion proteins, the coding sequences of FAM134B^WT/mutLIR^ and 6HIS-APP^WT/mut^ were amplified by PCR and cloned into GST-pGEX-4T1 and pET28a, respectively. All plasmids used in the study were subjected to sequencing verification.

### Bioinformatics analysis

A complete list of software and packages used in this study is provided in Reagents and tools table. Gene expression data related to Alzheimer’s disease (AD) were retrieved from the Gene Expression Omnibus (GEO) database (https://www.ncbi.nlm.nih.gov/geo/). The following datasets were included: GSE1297 (Blalock et al, 2004), GSE5281 (Liang et al, 2007), GSE28146 (Blalock et al, 2011), GSE29378 (Miller et al, 2013), GSE36980 (Hokama et al, 2014), GSE48350 (Berchtold et al, 2008), GSE159699 (Nativio et al, 2020), GSE173955 (Mizuno et al, 2021), GSE184942 (Gao et al, 2022), and GSE280268 (Ferrari et al, 2024). All datasets contain gene expression data from hippocampal tissue, and GSE5281, GSE36980, GSE48350, and GSE280268 also include cortical tissue expression data. If cornu ammonis (CA) regions were included, analyses were focused on the CA1 subregion. The CA1 region of the hippocampus plays a crucial role in memory formation, consolidation, and retrieval (Bartsch et al, 2011). For the unification of microarray and RNA-seq data, we used Rank-in algorithm (Tang et al, 2021) to correct the systematic difference between the two technologies. Data normalization and differential expression analysis were performed using the GEOquery, limma, and edgeR R packages, and combined heatmap visualization was generated using the ComplexHeatmap package. All raw *P* values were adjusted for false discovery rate (FDR) using the Benjamini–Hochberg (BH) method.

For ATAC-seq enrichment analysis of cortical tissue from AD cases in the Synapse database (dataset syn52293424 (Xiong et al, 2023)), data were processed using the Seurat, SingleCellExperiment, and GenomicRanges R packages. Histone modification datasets for H3K4me3 (GSE196413 (Persico et al, 2022)) and H3K27ac (GSE102538 (Marzi et al, 2018)) were obtained from GEO and analyzed using Pandas, Matplotlib, and Scanpy (Python). Differential enrichment analysis was performed to investigate the epigenetic regulation of *FAM134B* transcription in Alzheimer’s disease. To enhance the detection of regulatory signals, the traditional transcription start site (TSS) window was expanded from 1–3 kb to 10 kb.

### Quantitative reverse transcription polymerase chain reaction (qRT-PCR)

Total RNA was extracted with TRIzol (Invitrogen), and 1 µg of RNA was reverse-transcribed into cDNA using the HiScript III RT SuperMix (Vazyme, R323-01). The cDNA served as the template for quantitative PCR analysis using the ChamQ Universal SYBR qPCR Master Mix (Vazyme, Q711-02) and Real-Time PCR System (Bio-Rad). The primers used are detailed in Table EV2.

### Co-immunoprecipitation and immunoblotting

Cells were cultured to 80%–90% confluence in a 6-cm dish. After removing the medium, the cells were washed three times with PBS and lysed with 1 mL NP-40 lysis buffer (50 mM Tris-HCl, pH 7.4, 150 mM NaCl, 1% NP-40, 1 mM EDTA, 1x PMSF, and 1× protease inhibitor cocktail [Roche, 4693132001]) on ice for 30 min. After centrifugation at 12,000 × *g* for 30 min, a 100 µL aliquot of the supernatant was removed as an input sample for immunoblotting. The remaining supernatant was incubated with primary antibodies overnight, and then incubated with Protein A/G magnetic beads (Biolinkedin, L-1004/L-1004A) for 2 h at 4 °C. The beads were collected and washed three times with PBS. The immunoprecipitates were eluted from the beads by adding 1× SDS loading buffer (50 mM Tris-HCl, pH 6.8, 2.4% SDS, 12% glycerol, 50 mM DTT, 0.01% bromophenol blue) followed by boiling for 10 min at 65 °C and then subjected to SDS-PAGE and immunoblotting.

The protein samples were separated using SDS-PAGE and transferred onto polyvinylidene difluoride membranes (Millipore, 0.45 μm, IPVH0001). The membranes were blocked with 5% skimmed milk in TBST (TBS + 0.1% Tween-20) for 1 h at room temperature (RT) and then probed overnight with primary antibodies at 4 °C, followed by incubation with peroxidase-conjugated goat anti-rabbit or mouse IgG (H + L) secondary antibodies for 1 h at RT. Signals were detected using the BIO-RAD ChemiDoc MP Imaging System.

### Fluorescence microscopy and immunofluorescence staining

For fluorescence microscopy, cells were grown on coverslips to 70%–80% confluence and treated with the indicated conditions. The coverslips were then washed three times with PBS and fixed with 4% paraformaldehyde (PFA) at RT for 15 min. After washing the coverslips three more times with PBS, they were mounted with antifade mounting medium containing DAPI (Sigma, F6057-20ML). The coverslips were then examined using a laser scanning confocal microscope (ZEISS, LSM980; LEICA, Leica Stellaris 5 WLL) equipped with a ×63 oil immersion objective lens.

For immunofluorescence staining, cells were cultured and treated as described for fluorescence microscopy. The coverslips were then washed three times with PBS and fixed at RT for 15 min using 4% PFA. After fixation, the coverslips were washed three times with PBS. The samples were permeabilized with 0.1% Triton-X-100 for 15 min, followed by three PBS washes. Cells were then blocked with a buffer containing 10% goat serum in PBS for 2 h at RT. After blocking, the cells were incubated with primary antibodies overnight at 4 °C. The next day, cells were washed three times with PBS, followed by a 2-h incubation at RT with Alexa Fluor-conjugated secondary antibodies. The coverslips were thoroughly rinsed three times with PBS and mounted onto microscope slides using mounting medium containing DAPI. Finally, the slides were examined using a laser scanning confocal microscope (ZEISS, LSM980; LEICA, Leica Stellaris 5 WLL) or Multi-SIM (NanoInsights), equipped with a ×63 oil immersion objective lens.

Regarding the staining of three types of endogenous proteins, the pre-incubation procedures were consistent with the aforementioned protocol. Cells were incubated overnight at 4 °C with a mixture of primary antibodies containing anti-CALNEXIN (Proteintech, 10427-2-AP) and anti-APP (Proteintech, 60342-1-Ig) antibodies at a 1:200 dilution. Following three PBS washes, cells were incubated for 2 h at RT with a mixture of Alexa Fluor-conjugated secondary antibodies. Post-incubation, cells underwent another three PBS washes and were re-blocked with PBS containing 10% goat serum for 4 h at RT. Subsequent immunostaining was performed using either LAMP1 (DSHB, H4A3) or LC3B (Cell Signaling Technology, 83506) antibody overnight at 4 °C. All remaining steps followed the previously described methodology.

### Quantification of microscopy images to evaluate autophagic flux

U2OS cells stably expressing RFP-EGFP-KDEL or DOX-inducible APP^WT/mut^-mCherry-EGFP were randomly selected from three independent experiments, and mCherry⁺/EGFP^−^ puncta were manually quantified per cell. Image analysis was performed in a blinded manner using identical criteria across all samples.

### Generation of knockout cell lines using CRISPR-Cas9 gene editing

The CRISPR-Cas9 lentiviral system was used to generate knockout cell lines. gRNAs targeting ER-phagy receptors and ER chaperons were cloned into the lentiCRISPR v2-Blast vector. A complete list of gRNA sequences is provided in Table EV3. Note that the gRNA targeting FAM134B was designed to delete the canonical long isoform (FAM134B) without affecting the alternative isoform, FAM134B-2. Lentivirus particles were generated by transfecting gRNA vectors into HEK293T cells. Briefly, HEK293T cells were cultured in 6-well plates with 2 mL DMEM supplemented with 10% FBS until they reached 60% confluence. The cells were then transfected with 1.0 μg lentiviral plasmids carrying Cas9 and sgRNA guides, along with the two packaging vectors, 1.5 μg psPAX2 and 0.5 μg VSV-G. The lentivirus-containing supernatant was collected after 48 h and filtered through a 0.45-µm filter. The filtered supernatant was used to infect target cells for specific gene knockout. After a 24–36-h incubation, the cells were selected with 20 μg/mL blasticidin (further reduced to 5 μg/mL for the maintenance) for 4 days, until all non-transduced wild-type cells were eliminated. All antibiotic selections were performed for at least three passages to ensure complete selection.

### Autophagy flux analysis by flow cytometry

U2OS cells stably expressing mCherry-EGFP-RAMP4 or RFP-EGFP-KDEL, as well as cells expressing DOX-inducible APP^WT/mut^-mCherry-EGFP, were pelleted at 2000 ×g for 1 min and resuspended in PBS. Samples were analyzed on a CytoFlex LX flow cytometer (Beckman Coulter) equipped with 488-nm and 561-nm lasers. EGFP fluorescence was collected using a 530/30-nm filter, and mCherry/RFP fluorescence using a 610/20-nm filter. Data were processed in FlowJo. Live single cells were gated based on FSC-A/SSC-A and FSC-H/FSC-A parameters. Cells positive for both EGFP and mCherry were selected, and at least 10,000 events were acquired per sample. Autophagic flux was quantified as the percentage of cells exhibiting an increased red-to-green fluorescence ratio (mCherry/EGFP or RFP/EGFP), reflecting quenching of EGFP in acidic lysosomes. The gate defining an “enhanced ratio” was established based on control cells cultured in nutrient-rich medium and was then applied uniformly to all samples.

### Correlative light and electron microscopy (CLEM)

Cells were cultured on gridded dishes and treated with EBSS and BafA1 for 6 h. Following fixation in 0.1 M phosphate buffer (pH 7.4) containing 4% PFA and 0.2% glutaraldehyde for 30 min at room temperature, fluorescent images were acquired using a ZEISS LSM980 confocal microscope. Subsequent electron microscopy processing and correlative alignment with confocal images were performed as previously described (Yang et al, 2025).

### GST pull-down

The recombinant vectors were transformed into *E. coli* BL21 (DE3) cells and induced by adding 1 mM IPTG, followed by incubation overnight at 18 °C with shaking at 220 rpm. The fusion proteins were purified using GSTSep Glutathione MagBeads (YEASEN, 20562ES08) and Ni-NTA Beads 6FF (Smart-Lifescience, SA005010). The Ni-NTA beads were then eluted three times with 500 μL of elution buffer (50 mM NaH_2_PO_4_, pH 8.0, 300 mM NaCl, 250 mM imidazole), filtered through a 0.22 μm filter. The elution buffer was exchanged with binding buffer (50 mM Tris-HCl, pH 8.0, 200 mM NaCl, 1% NP40, 1 mM EDTA, 1 mM DTT, 10 mM MgCl_2_) via ultrafiltration. The GST-fusion protein beads were collected and washed three times with PBS. HIS-fusion proteins were then added to the GST-fusion protein beads and incubated on a rotator at 4 °C overnight. After incubation, the supernatant was aspirated, and the beads were washed three times with 1 mL of PBS. After washing, 60 μL of 1× SDS loading buffer was added to the beads and incubated for 10 min at 65 °C. The resulting supernatants were loaded onto SDS-PAGE gels.

### Dimerization assay

Cells were co-transfected with 3HA-FRB(T2098L)-FAM134B and Myc-APP-FKBP12 constructs. After 24 h, 500 nM Rapalog (Clontech, #635057) was added for 6 h to induce dimerization. Co-IP: HEK293T cells (6 cm dish) were transfected with 1.5 µg each of 3HA-FRB(T2098L)-FAM134B and Myc-APP-mCherry-FKBP12. Cells were harvested for co-immunoprecipitation. Immunofluorescence: U2OS cells on coverslips were transfected with 0.35 µg each of 3HA-FRB(T2098L)-EGFP-FAM134B and Myc-APP-mCherry-FKBP12. After treatment, cells were fixed with 4% PFA and stained for LC3B. Flow Cytometry: HEK293T cells (6-well) were transfected with 0.75 µg each of 3HA-FRB(T2098L)-FAM134B and Myc-APP-mCherry-EGFP-FKBP12. Cells were collected post-treatment for analysis.

### Nuclear/cytosol fractionation

Cells were transferred from 6 cm plates into a 1.5 mL EP tube with 300 μL of fractionation buffer (10 mM HEPES, pH 7.8, 10 mM KCl, 1.5 mM MgCl_2_, 0.5 mM β-mercaptoethanol, 1 mM DTT, 1× PMSF, 1× proteinase inhibitor cocktail) and incubated on ice for 30 min. Add 6 μL of 10% NP-40 to the tube and vortex for 2 min. An aliquot of 100 μL lysate was incubated at 65 °C for 30 min as the total cell lysate fraction. Centrifuge the remaining sample at 16,000 × *g* for 30 min at 4 °C. 20 μL of 6× SDS loading buffer was added to 100 μL of the supernatant for the cytosol fraction. The pellet was washed three times with 1 mL PBS and then resuspended in 120 μL of 1× SDS loading buffer and incubated for 30 min at 65 °C for the nuclear fraction.

### Chromatin immunoprecipitation followed by quantitative PCR (ChIP-qPCR)

ChIP-qPCR was performed as described previously (Xiao et al, 2019). Briefly, approximately 1 × 10^7^ cells or 10 mg hippocampus from WT and 5XFAD mice were fixed in 1% formaldehyde and resuspended in lysis buffer (10 mM Tris-Cl, pH 8.0, 10 mM NaCl, 0.5% NP-40, 1× proteinase inhibitor cocktail) on ice for 20 min with inversion every 4 min. Chromatin was sheared to 100–500 bp using a Branson Sonifier cell disrupter (output: 4; sonicate for 10 s for seven times and wait 1 min in between to cool). 5 µg TFEB, TFE3, H3K4me3, H3K27ac antibodies and magnetic beads were coupled, then incubated with solubilized chromatin overnight at 4 °C. Target-chromatin complexes were pulled down using a magnet stand, washed sequentially with TSE buffer, wash buffer, and TE buffer and then eluted with Elution buffer. Immunoprecipitated samples were reverse crosslinked overnight at 65 °C on thermomixer. Through RNase treatment, proteinase K treatment, immunoprecipitated DNA was extracted with phenol-chloroform, ethanol precipitated. The DNA samples were purified and analyzed via qRT-PCR. The primers used are detailed in Table EV2.

### Assay for transposase-accessible chromatin followed by qPCR (ATAC-qPCR)

ATAC-qPCR was performed using the Hyperactive ATAC-Seq Library Prep Kit for Illumina (Vazyme, TD711-01), following the manufacturer’s instructions. Briefly, ~10 mg of hippocampal tissue was lysed in cold buffer (48.5 µL RS buffer, 0.5 µL 10% NP-40, 0.5 µL 1% Digitonin, 0.5 µL 10% Tween-20) on ice for 5 min. Nuclei were pelleted at 500 × *g* for 5 min at 4 °C, and the supernatant discarded. Each sample was then resuspended in 50 µL of pre-chilled fragmentation mix (16.5 µL TW Buffer, 0.5 µL 10% Tween-20, 0.5 µL 1% Digitonin, 18.5 µL nuclease-free H₂O, 10 µL 5× TTBL, 4 µL TTE Mix V50), mixed gently by pipetting, and incubated at 37 °C for 30 min without agitation. After brief centrifugation, 5 µL stop buffer was added to quench the reaction, followed by incubation at room temperature for 5 min. DNA was purified using ATAC DNA Extract Beads. Nuclear integrity was assessed using Trypan Blue staining, requiring >70% intact nuclei. Purified DNA was then analyzed by qRT-PCR. The primers used are detailed in Table EV2.

### Stereotactic injection of adeno-associated virus

AAV2/9-CMV-EGFP-P2A-FAM134B^WT^-3XFLAG, AAV2/9-CMV-EGFP-P2A-FAM134B^mutLIR^-3XFLAG, and AAV2/9-CMV-EGFP-P2A-3XFLAG viruses were purchased from OBiO Technology (Shanghai). In total, 5 × 10^8^ vg of virus (500 nL 1 × 10^12^ vg/mL) was stereotactically injected into the bilateral hippocampal area (X = ± 1.5 mm. Y = -2.0 mm. Z = -2.0 mm) of 5-month-old mice. One month after injection, mice were sacrificed after behavioral tests, and their brains were harvested for immunofluorescence staining and western blotting.

### Morris water maze behavioral assessment

The Morris water maze test was used to assess spatial learning and memory in mice. Experiments were conducted in a circular tank (120 cm diameter, 50 cm depth) filled with opaque water maintained at room temperature. Four distinct visual cues of different shapes were placed on the walls of the tank as spatial references. A 10-cm diameter escape platform was submerged 1 cm below the water surface. Mice were trained for four consecutive days to locate the platform within 60 s and remain on it for at least 3 s. During training, mice were randomly released from one of four quadrants (N, S, E, W). On the test day, the platform was removed, and the mice were placed at the NE point and allowed to swim for 1 min. Escape latency, number of platform crossings, and time spent in each quadrant were recorded.

### Novel object recognition (NOR) test

The NOR test assesses hippocampus-dependent recognition memory in mice by exploiting their innate preference for novel objects. The test is conducted in a white opaque cubic chamber (45 × 45 × 45 cm) and comprises three phases: (1) a 10-min habituation to the empty chamber; (2) a 5-min familiarization with two identical objects; and (3) a 5-min test in which one object is replaced with a novel one. Exploration behavior is recorded using VisuTrack software, and the novelty preference index is calculated as (time exploring the novel object)/(total exploration time of both objects) for statistical analysis.

### Hematoxylin and eosin (H&E) staining

Mouse brain sections were subjected to sequential processing, beginning with xylene clearing (3 × 15 min) followed by graded ethanol rehydration (100% ethanol 5 min, 95% ethanol 2 min, 75% ethanol 2 min) and distilled water rinsing. Nuclear staining is achieved through 8-min hematoxylin immersion with subsequent running water rinses, differentiated in 1% acid alcohol (20 s), followed by cytoplasmic counterstaining with eosin (2 min). Sections are then dehydrated in 95% ethanol and 100% ethanol (5 min each), cleared in xylene (2 × 5 min), air-dried in a fume hood for 20 min, and finally mounted with neutral resin for microscopic imaging (Leica Aperio VERSA 8).

### Transmission electron microscopy (TEM) analysis

The hippocampus and prefrontal cortex were harvested from WT and 5XFAD mice and fixed in 2.5% GA solution for 2 h. Subsequently, the tissues were fixed in 1% osmium tetroxide for 2 h, followed by dehydration through a series of graded ethanol concentrations ranging from 50% to 100%. The tissues were then embedded in Spurr’s epoxy resin and sectioned into ultrathin slices of 60 nm. Finally, the sections were stained with uranyl acetate and lead citrate. TEM (JEOL, JEM-1400plus) was used to acquire images.

### Dot blot to measure Aβ levels

A grid was drawn on the nitrocellulose membrane (NC) with a pencil. In total, 2 µL of each sample was spotted onto the NC membrane. Let the membrane dry for 10 min at RT. The NC membrane was blocked with 5% skimmed milk in TBST (TBS + 0.1% Tween-20) for 1 h at RT and then probed overnight with Aβ antibody (Thermo Fisher Scientific, 700254) at 4 °C, followed by incubation with peroxidase-conjugated goat anti-mouse IgG (H + L) secondary antibody for 1 h at RT. Signals were detected using the BIO-RAD ChemiDoc MP Imaging System.

### FAM134B ubiquitination assay

APP^WT^ or APP^mut^ expressing cells were transfected with 3 µg of EGFP-FAM134B plasmid. After 24 h, cells were lysed in 250 µL of lysis buffer (50 mM Tris-HCl, pH 7.5, 150 mM NaCl, 0.5 mM EDTA, 1% NP-40, supplemented with Roche EDTA-free protease inhibitors) for 30 min at 4 °C. Lysates were collected using a scraper and centrifuged at 12,000 × *g* for 30 min at 4 °C. A 50 µL aliquot was reserved as input control. For mouse samples, 10 mg of hippocampal tissue was homogenized and processed in the same way.

To denature the remaining lysate, 20 µL of 10% SDS was added to 180 µL of lysate and the mixture was heated at 95 °C for 5 min. The denatured lysates were incubated overnight with anti-GFP or anti-FAM134B antibodies at 4 °C, followed by incubation with Protein A/G magnetic beads for 2 h. Beads were washed three times with PBS, and boiled in 60 µL of 1× SDS loading buffer. Samples were analyzed by SDS-PAGE and immunoblotting.

### Native PAGE for oligomerization

APP^WT^ or APP^mut^ expressing cells were transfected with 3 µg of EGFP-FAM134B plasmid in 6 cm dishes. After 24 h, the culture medium was removed, and the cells (or 10 mg mouse hippocampal tissue) were washed three times with ice-cold PBS. Samples were lysed in 250 µL of native lysis buffer (25 mM Tris-HCl, pH 7.5, 150 mM NaCl, 1% Triton-X-100, 1 mM EDTA) for 30 min at 4 °C. Lysates were collected using a cell scraper and centrifuged at 12,000 × *g* for 30 min at 4 °C. An aliquot (50 µL) was taken as the input sample for SDS-PAGE and immunoblotting. The remaining supernatant was mixed with 4× native loading buffer (250 mM Tris-HCl, pH 6.8, 40% glycerol, 4% sodium deoxycholate, 0.06% bromophenol blue), and samples were analyzed by native PAGE followed by immunoblotting.

### Cell fractionation based on density gradient

Sucrose solutions for step gradients (60%, 55%, 50%, 45%, 40%, 35%, 30%, 25%, and 20%) were prepared in advance. Harvested cells were resuspended in 4.5 mL of ice-cold MIB-S buffer (10 mM HEPES, pH 8.0, 0.25 M D-sucrose, 25 mM KCl, 1 mM EGTA) and centrifuged at 600 × *g* for 5 min at 4 °C. The resulting pellet was resuspended in three volumes of SB buffer (50 mM HEPES, pH 8.0, 125 mM KCl, 5 mM EGTA), incubated for 30 min at 4 °C, and centrifuged again at 600 × *g* for 5 min at 4 °C. The pellet was then homogenized in 8 mL of MIB-S buffer using a Dounce homogenizer (tight pestle, 20 strokes). A 150 μL aliquot of the homogenate was saved as a control and stored at −20 °C.

The remaining homogenate was clarified by centrifugation at 600 × *g* for 5 min at 4 °C, and this step was repeated once. The supernatant was then mixed with 1.4 volumes of 60% sucrose solution. A step gradient (from bottom to top: 60% to 20% sucrose in 5% increments, 1 mL per layer) was carefully layered. The samples were subjected to ultracentrifugation at 21,000 rpm for 2 h at 4 °C using a Beckman Optima XE-100 ultracentrifuge. Distinct organelle-enriched milky bands at the interfaces were collected for further analysis.

### Bisulfite sequencing PCR

Total DNAs were isolated from hippocampus using the TIANamp Genomic DNA Kit (TIANGEN, DP304), and unmethylated cytosine-to-uracil conversion was performed using the DNA Bisulfite Conversion Kit (Beyotime, D0068S). BSP procedures were performed as previously described (Chen et al, 2021a).

For BSP assay, Fam134b promoter-specific primers were used: forward primer 5′- GTAGGTTGGATATAGAGTATGTTTAGAGG (−1029/−1001) and reverse primer 5′- CATTCTCACTTTCTCCAACCAAAAC (−427/−451). PCR products were resolved on agarose gel, purified, and cloned into the pCE2 TA/Blunt-Zero Vector (Vazyme Biotech, C601-01). Fifteen colonies from each PCR reaction were randomly selected for sequencing, and the ratio of methylated cytosines to total cytosines in the cloned fragments was determined.

### Statistical analysis

“*N*” indicates the number of individual samples (e.g., cells, humans, or mice) analyzed, and “*n*” denotes the number of independent biological replicates. Data are presented as mean ± SEM. For normally distributed data, comparisons between two groups were made using a two-tailed unpaired Student’s *t* test. One-way ANOVA was used for comparisons across multiple groups, and two-way ANOVA followed by Tukey’s post hoc test was performed to assess interactions between independent variables. Statistical analyses were performed using GraphPad Prism v8.0 (GraphPad Software, San Diego, CA). Exact *P* values and sample sizes are provided in the figures and figure legends. Differences were considered statistically significant at *P* < 0.05.

## Supplementary information

Table EV1

Table EV2

Table EV3

Peer Review File

Source data Fig. 1

Source data Fig. 2

Source data Fig. 3

Source data Fig. 4

Source data Fig. 5

Source data Fig. 6

Expanded View Figures

## Data Availability

This study includes no data deposited in external repositories. The source data of this paper are collected in the following database record: biostudies:S-SCDT-10_1038-S44318-026-00818-9.
