# Supplementary material for: FAM134B-mediated ER-phagy degrades APP and suppresses Alzheimer’s disease pathology
Source: EMBO J. 2026 May 26;45(13):4492–530. doi: 10.1038/s44318-026-00818-9 (PMC13324857; doi:10.1038/s44318-026-00818-9)
Supplement: Supplementary file 1 — Table EV1 [file 44318_2026_818_MOESM1_ESM.docx]

**Table EV1**: Complete information on human tissue samples.

| **Case**  **No.** | **Primary**  **neuropathologic**  **diagnosis** | **Secondary neuropathologic diagnosis** | **Age at death/Bx** | **Sex** | **Brain regions** | **Braak score** |
| --- | --- | --- | --- | --- | --- | --- |
| 1 | Control |  | 75 | m | Hippocampus | I |
| 2 | Control |  | 57 | f | Hippocampus | I |
| 3 | Control |  | 65 | m | Hippocampus | I |
| 4 | AD | LBD-amygdala | 54 | m | Hippocampus | II |
| 5 | AD |  | 57 | m | Hippocampus | III |
| 6 | AD | Microinfarcts | 77 | f | Hippocampus | V |
| 7 | AD |  | 69 | m | Hippocampus | IV |
