# Supplementary material for: FAM134B-mediated ER-phagy degrades APP and suppresses Alzheimer’s disease pathology
Source: EMBO J. 2026 May 26;45(13):4492–530. doi: 10.1038/s44318-026-00818-9 (PMC13324857; doi:10.1038/s44318-026-00818-9)
Supplement: Supplementary file 2 — Table EV2 [file 44318_2026_818_MOESM2_ESM.docx]

**Table EV2**: A complete list of primers used for mouse genotyping, qPCR, ChIP–qPCR, and ATAC–qPCR. Note: Human genes are indicated in uppercase italics (e.g., *FAM134B*), whereas mouse genes are indicated with an initial uppercase letter followed by lowercase italics (e.g., *Fam134b*).

| **Target gene** | **Primer sequence (5’-3’)** | **Note** |
| --- | --- | --- |
| Mouse-Com | CATGTCCTACAGCCCCTCTC | F-primer PCR |
| WT mouse | ACCATTTGCAAGGAAAGCAC | R-primer PCR |
| 5XFAD-mouse | AAGCTAGCTGCAGTAACGCCATTT | R-primer PCR |
| *FAM134B* | CTCAGCCACTGTATTGCAGAA | F-primer qPCR |
|  | AACAATGGACACAAAAATGCACA | R-primer qPCR |
| *FAM134A* | ACACGACAAGAGGAAGCGTC | F-primer qPCR |
|  | ACTCTCCAAGATAGAAGCCTCC | R-primer qPCR |
| *FAM134C* | TTTGCCCTGACATCTCTTCGT | F-primer qPCR |
|  | CTCATTGTCTAATGCGTCGGG | R-primer qPCR |
| *ATL3* | ACAGCCAGTCAACTGTGAAAG | F-primer qPCR |
|  | CCAGACGACCGTATTCTGTGA | R-primer qPCR |
| *RTN3* | ACTGGGTTTGTCTTTGGCAC | F-primer qPCR |
|  | ATGACGGACTTGTAGATCCTGA | R-primer qPCR |
| *CCPG1* | AATGGCACAGTGCTTATGGAA | F-primer qPCR |
|  | GGTGGCTCAAGGGTAACAATATC | R-primer qPCR |
| *TEX264* | ATGTCGGACCTGCTACTACTG | F-primer qPCR |
|  | GCCCCATGTGGAACTTGTAGG | R-primer qPCR |
| *C53* | GAGTCTGGTGCTGACGATCC | F-primer qPCR |
|  | TGTGAAGAGTATCGGCCAAAAAT | R-primer qPCR |
| *CALCOCO1* | GTAGCCCGGACCTACATCC | F-primer qPCR |
|  | GCACGGAAGACCACACAAAT | R-primer qPCR |
| *SEC62* | CCAGCAGAAATGAGAGTAGGTG | F-primer qPCR |
|  | GAGTCAATGAAGCCCACATCA | R-primer qPCR |
| *TFEB* | ACCTGTCCGAGACCTATGGG | F-primer qPCR |
|  | CGTCCAGACGCATAATGTTGTC | R-primer qPCR |
| *TFE3* | CCGTGTTCGTGCTGTTGGA | F-primer qPCR |
|  | GCTCGTAGAAGCTGTCAGGAT | R-primer qPCR |
| *APP* | GGTACCCGAACAAAAACTCATCT | F-primer qPCR |
|  | GCAGGATGCCTTCCTTGGTA | R-primer qPCR |
| *LAMP1* | TCTCAGTGAACTACGACACCA | F-primer qPCR |
|  | AGTGTATGTCCTCTTCCAAAAGC | R-primer qPCR |
| *ATP6V0E1* | GTCCTAACCGGGGAGTTATCA | F-primer qPCR |
|  | AAAGAGAGGGTTGAGTTGGGC | R-primer qPCR |
| *CTSB* | GAGCTGGTCAACTATGTCAACA | F-primer qPCR |
|  | GCTCATGTCCACGTTGTAGAAGT | R-primer qPCR |
| *VPS11* | CGGCGCTTCGTTTTCTTCG | F-primer qPCR |
|  | CCCGTAGTTTGTAGGCTTGGAA | R-primer qPCR |
| *VPS18* | CACTCGGGGTATGTGAATGCC | F-primer qPCR |
|  | TCGGAAGGGGTGAAGTCAATG | R-primer qPCR |
| *p62* | GCACCCCAATGTGATCTGC | F-primer qPCR |
|  | CGCTACACAAGTCGTAGTCTGG | R-primer qPCR |
| *MAPK1* | TACACCAACCTCTCGTACATCG | F-primer qPCR |
|  | CATGTCTGAAGCGCAGTAAGATT | R-primer qPCR |
| *GAPDH* | GTCTCCTCTGACTTCAACAGCG | F-primer qPCR |
|  | ACCACCCTGTTGCTGTAGCCAA | R-primer qPCR |
| *Fam134b* | AAACAGCAGAGTCCTGGCAAG | F-primer qPCR |
|  | AGGTAGCTGAGTATGACCCCA | R-primer qPCR |
| *Fam134a* | GAGGCAGTGGGACGTTTAGC | F-primer qPCR |
|  | GAAGAGGCCGTTGAGTGTGG | R-primer qPCR |
| *Fam134c* | CTTCGGGGTTGGCTTTCAG | F-primer qPCR |
|  | AGCAAAGGCTCGTAAGGGC | R-primer qPCR |
| *Atl3* | CTGGACTTTATGCTGCGATACTT | F-primer qPCR |
|  | AGCCTCCTCGCCATGAAAATC | R-primer qPCR |
| *Rtn3* | AGGTGCCCCTACGATGTCTC | F-primer qPCR |
|  | GGTTTGCTTGAGTTTTCCTCCA | R-primer qPCR |
| *Ccpg1* | AGCGACAGCGACTCATCTTG | F-primer qPCR |
|  | TCACCTCCATGCCCTACATAAA | R-primer qPCR |
| *Tex264* | CCAATCCGCAACATAACTGTGG | F-primer qPCR |
|  | GGGTTGTCATAGTAGACAGCGAT | R-primer qPCR |
| *C53* | ACTGCCTAAGAATAGTGGACCT | F-primer qPCR |
|  | TTTCATCCGTTGTGAAGAGTACC | R-primer qPCR |
| *Calcoco1* | CAAGGTGGAATGTCACTACACTT | F-primer qPCR |
|  | CGTGTGATAATCTCGAACGCAG | R-primer qPCR |
| *Sec62* | TGATTGCAGTAATAGCAGCCAC | F-primer qPCR |
|  | GCCCACACTGAGGTAATAAACAC | R-primer qPCR |
| *Tfeb* | GCTCCAACCCCGAGAAAGAG | F-primer qPCR |
|  | CAGCGTGTTAGGCATCTGC | R-primer qPCR |
| *Tfe3* | CCCAGCTACACTCTCTGCG | F-primer qPCR |
|  | GCAAGACCCGTGATGAAGAAG | R-primer qPCR |
| *Lamp1* | CAGCACTCTTTGAGGTGAAAAAC | F-primer qPCR |
|  | CCATTCGCAGTCTCGTAGGTG | R-primer qPCR |
| *Atp6v0e1* | GCATACCACGGCCTTACTGT | F-primer qPCR |
|  | TGATAACTCCCCGGTTAGGAC | R-primer qPCR |
| *Cstb* | AGTCCCAGCTTGAATCGAAAG | F-primer qPCR |
|  | GGGGAGGGGTTGAAACACC | R-primer qPCR |
| *Vps11* | CGGCGTTTCGTTTTCTTCGAG | F-primer qPCR |
|  | GCCTGAGTCGCAGACAGTG | R-primer qPCR |
| *Vps18* | AGTACGAGGACTCATTGTCCC | F-primer qPCR |
|  | TGGGCACTTACATACCCAGAAT | R-primer qPCR |
| *p62* | GAGGCACCCCGAAACATGG | F-primer qPCR |
|  | ACTTATAGCGAGTTCCCACCA | R-primer qPCR |
| *Mapk1* | GGTTGTTCCCAAATGCTGACT | F-primer qPCR |
|  | CAACTTCAATCCTCTTGTGAGGG | R-primer qPCR |
| *Pgk1* | TGCACGCTTCAAAAGCGCACG | F-primer qPCR |
|  | AAGTCCACCCTCATCACGACCC | R-primer qPCR |
| *Bip* | AGGACAAGAAGGAGGATGTGGG | F-primer qPCR |
|  | ACCGAAGGGTCATTCCAAGTG | R-primer qPCR |
| *Ire1* | GAGCAAGCTAACGCCTACTCTGT | F-primer qPCR |
|  | CACCATTGAGGGAGAGGCATA | R-primer qPCR |
| *sXbp1* | GGTCTGCTGAGTCCGCAGCAGG | F-primer qPCR |
|  | CTCTGGGGAAGGACATTTGA | R-primer qPCR |
| *Perk* | GGTATTTCAACGCCTGGCTG | F-primer qPCR |
|  | GGCCAGTCTGTGCTTTCGTC | R-primer qPCR |
| *Chop* | CACATCCCAAAGCCCTCG | F-primer qPCR |
|  | CTCAGTCCCCTCCTCAGC | R-primer qPCR |
| *Atf4* | GGACAGATTGGATGTTGGAGAAAATG | F-primer qPCR |
|  | GGAGATGGCCAATTGGGTTCAC | R-primer qPCR |
| *Atf6* | ACAGCTACCTAACCATGTG | F-primer qPCR |
|  | AGCGATATCCGAACCCATAC | R-primer qPCR |
| *Fam134b* | GAGCACGCCACACAAGGAT | F-primer ChIP-qPCR |
|  | TGCTTCCTCTCCGGGAACT | R-primer ChIP-qPCR |
| *Fam134b* | TCGTTGGTCCCAGGCTCTC | F-primer ATAC-qPCR |
|  | GGAAAGAGGGTGCGGTCAAG | R-primer ATAC-qPCR |
| *Gapdh* | GGGCACCGATTCCTAGGTTG | F-primer ChIP/ATAC-qPCR |
|  | TATGGACTGTTTCTGGGCCG | R-primer ChIP/ATAC-qPCR |
