# Supplementary material for: FAM134B-mediated ER-phagy degrades APP and suppresses Alzheimer’s disease pathology
Source: EMBO J. 2026 May 26;45(13):4492–530. doi: 10.1038/s44318-026-00818-9 (PMC13324857; doi:10.1038/s44318-026-00818-9)
Supplement: Supplementary file 3 — Table EV3 [file 44318_2026_818_MOESM3_ESM.docx]

**Table EV3**: A complete list of gRNA information.

| **Target gene** | **Nucleotide sequence (5’-3’)** |
| --- | --- |
| *FAM134B* | CGCCGACGAGCTGCTGAGCTT |
| *FAM134A* | ACGCTGTGGCTGCGGCTCCG |
| *FAM134C* | CGGGCACGCTGAGCAACCG |
| *ATL3* | CAGCTGCTGCCACTCGCTGA |
| *RTN3* | AATTGACTTGGTCTGATCTC |
| *CCPG1* | GGCTCACAGCTGTCAGTGG |
| *TEX264* | TACTCAGGGCTACTGGCTG |
| *C53* | TGACTCTGGCATCTCTGCCG |
| *CALCOCO1* | CACTGGGCATGGTGCCTGG |
| *SEC62* | CCGGTGACCCATCATATTGG |
| *CALNEXIN* | CAAAGCTCCAGTTCCAACAG |
| *HSPA5* | GAGGTGAGCTGGTTCTTGG |
| *PGRMC1* | CGCCGCTCTCCAGATCGCTT |
| *CALR* | CATGAGCAGAACATCGACTG |
| *NC* | GAAGTTCGAGGGCGACACCC |
