# Supplementary material for: FAM134B-mediated ER-phagy degrades APP and suppresses Alzheimer’s disease pathology
Source: EMBO J. 2026 May 26;45(13):4492–530. doi: 10.1038/s44318-026-00818-9 (PMC13324857; doi:10.1038/s44318-026-00818-9)
Supplement: Supplementary file 5 — Source data Fig. 1 [file 44318_2026_818_MOESM5_ESM.zip › Figure 1/Figure 1J/RHD与IDR序列.pdf]

FAM134B：五四次跨膜；1-497 aa; RHD 80-260 aa；IDR：1-79 aa+261-497 aa

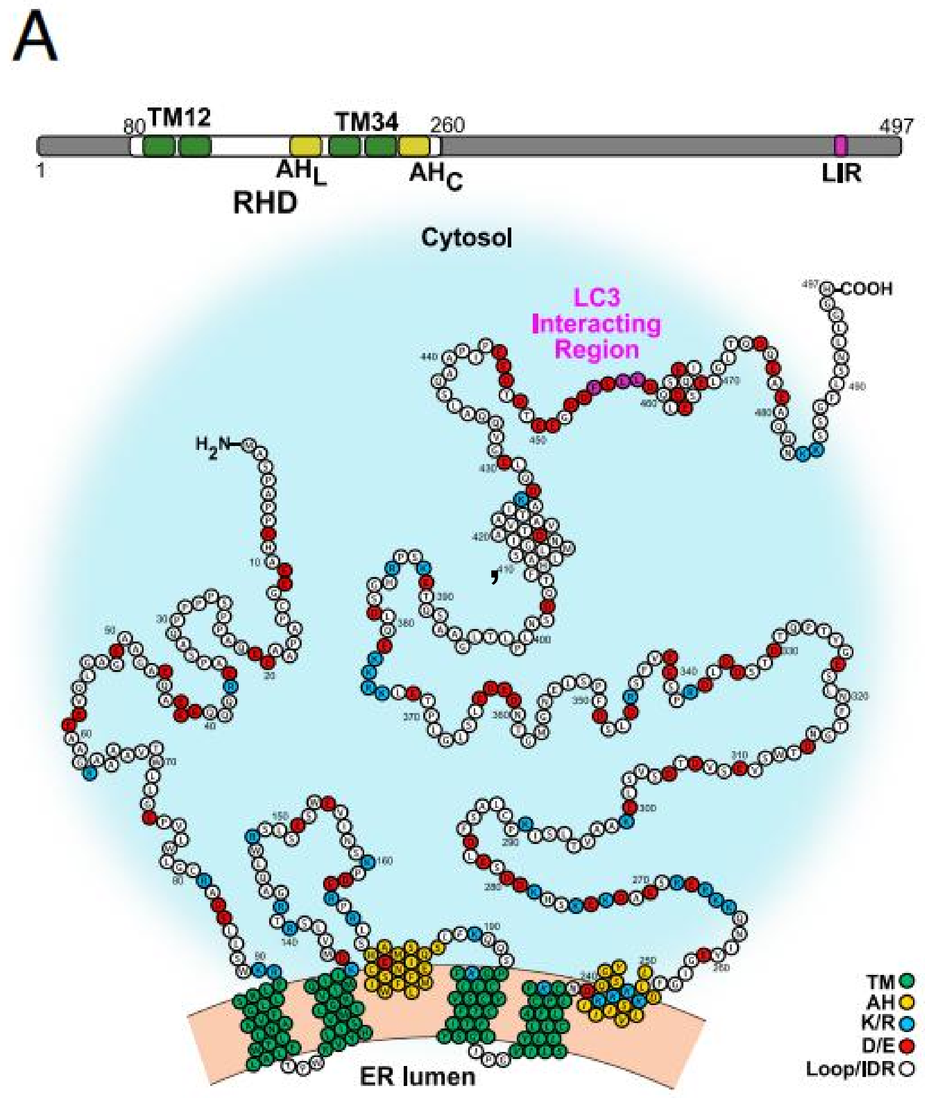


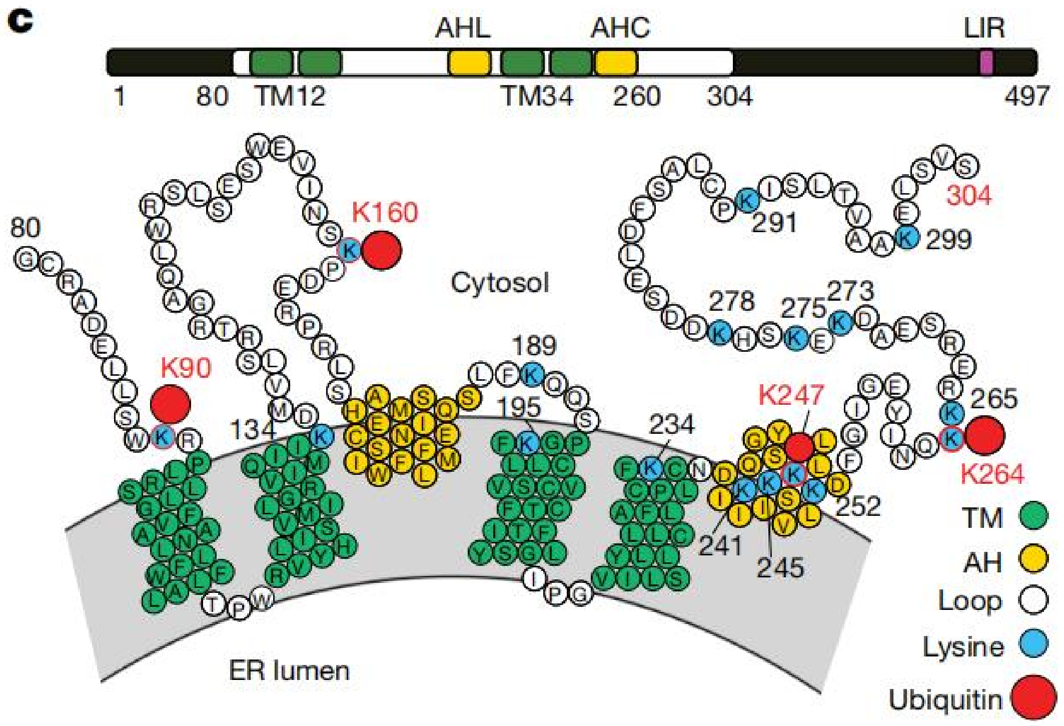


FAM134B：Q9H6L5；LC3B：Q9GZQ8

# YIPF3：五次跨膜；1-350 aa; TMD 149-297 aa；IDR预测：1-74 aa

TMHMM result

```
# NP_056203.2 Length: 350
# NP_056203.2 Number of predicted TMHs:  5
# NP_056203.2 Exp number of AAs in TMHs: 109.84169
# NP_056203.2 Exp number, first 60 AAs:  0.0002
# NP_056203.2 Total prob of N-in:        0.94631
NP_056203.2     TMHMM2.0        inside       1   148
NP_056203.2     TMHMM2.0        TMhelix    149   171
NP_056203.2     TMHMM2.0        outside    172   185
NP_056203.2     TMHMM2.0        TMhelix    186   208
NP_056203.2     TMHMM2.0        inside     209   214
NP_056203.2     TMHMM2.0        TMhelix    215   237
NP_056203.2     TMHMM2.0        outside    238   240
NP_056203.2     TMHMM2.0        TMhelix    241   263
NP_056203.2     TMHMM2.0        inside     264   274
NP_056203.2     TMHMM2.0        TMhelix    275   297
NP_056203.2     TMHMM2.0        outside    298   350
```

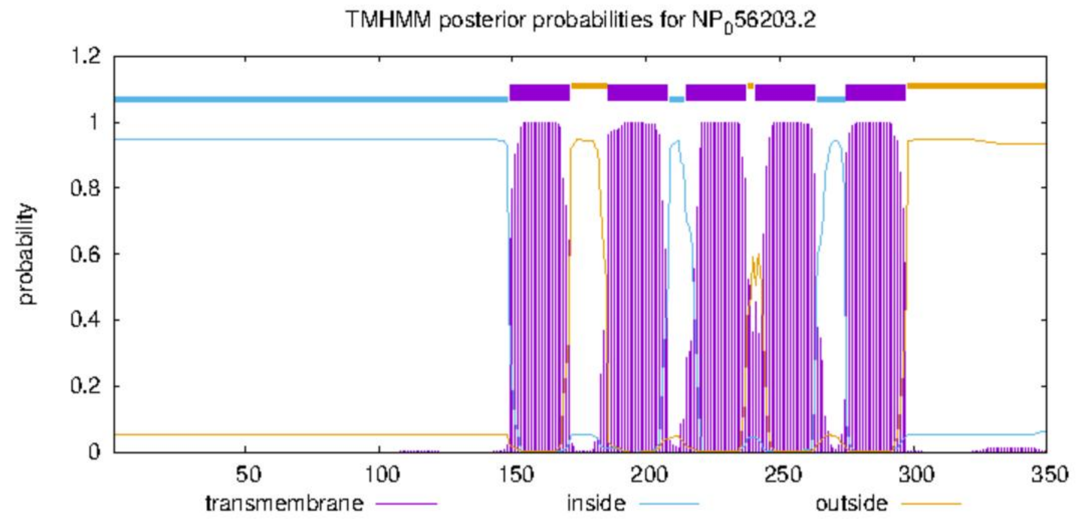


# plot in postscript, script for making the plot in gnuplot, data for plot

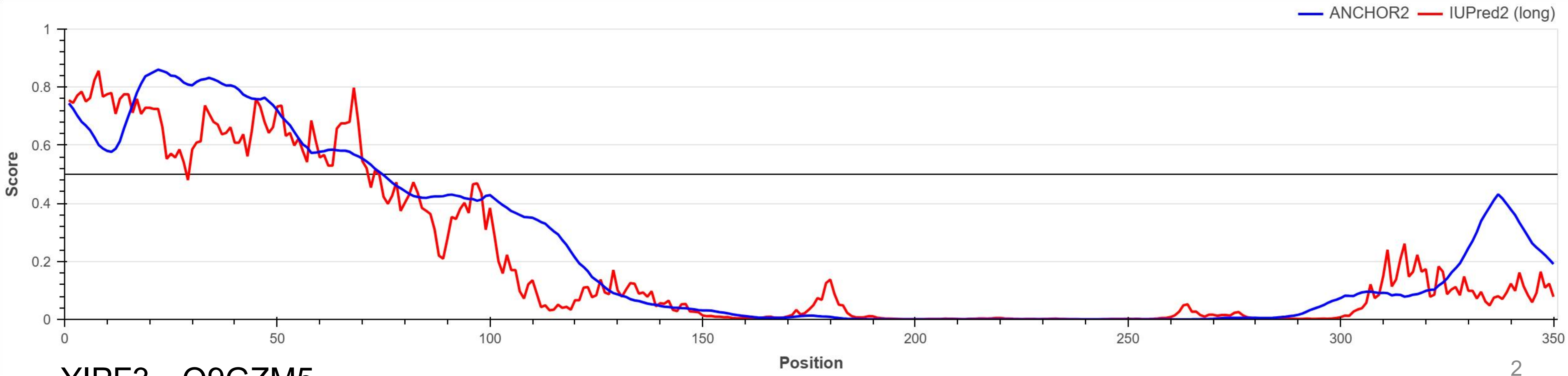


YIPF3：Q9GZM5

# YIPF4：五次跨膜；1-244 aa; TMD 115-243 aa；IDR预测：1-7 aa

## TMHMM result

```
# NP_115688.1 Length: 244
# NP_115688.1 Number of predicted TMHs:  5
# NP_115688.1 Exp number of AAs in TMHs: 101.38442
# NP_115688.1 Exp number, first 60 AAs:  0.00053
# NP_115688.1 Total prob of N-in:        0.36872
NP_115688.1     TMHMM2.0        inside       1   114
NP_115688.1     TMHMM2.0        TMhelix    115   134
NP_115688.1     TMHMM2.0        outside    135   137
NP_115688.1     TMHMM2.0        TMhelix    138   160
NP_115688.1     TMHMM2.0        inside     161   166
NP_115688.1     TMHMM2.0        TMhelix    167   189
NP_115688.1     TMHMM2.0        outside    190   192
NP_115688.1     TMHMM2.0        TMhelix    193   215
NP_115688.1     TMHMM2.0        inside     216   223
NP_115688.1     TMHMM2.0        TMhelix    224   243
NP_115688.1     TMHMM2.0        outside    244   244
```

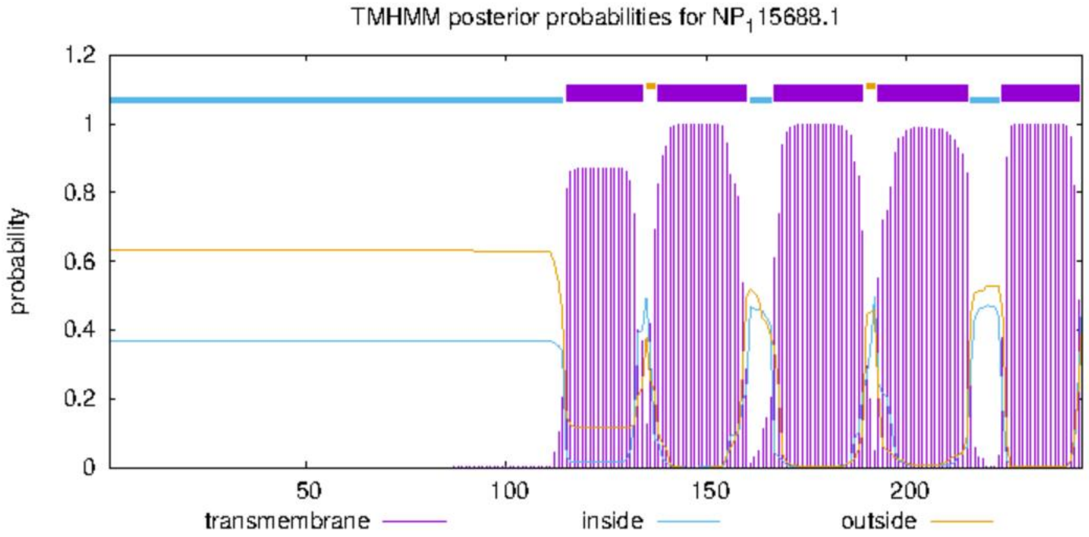


# plot in postscript, script for making the plot in gnuplot, data for plot

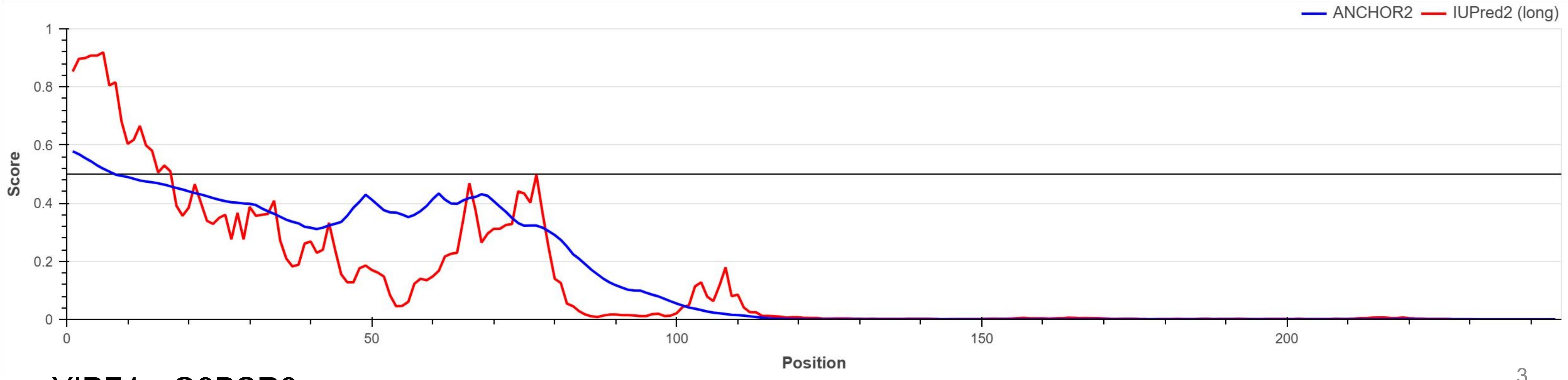


YIPF4：Q9BSR8
