# Supplementary figures and images for: FAM134B-mediated ER-phagy degrades APP and suppresses Alzheimer’s disease pathology (part 1 of 3)
Source: EMBO J. 2026 May 26;45(13):4492–530. doi: 10.1038/s44318-026-00818-9 (PMC13324857; doi:10.1038/s44318-026-00818-9)

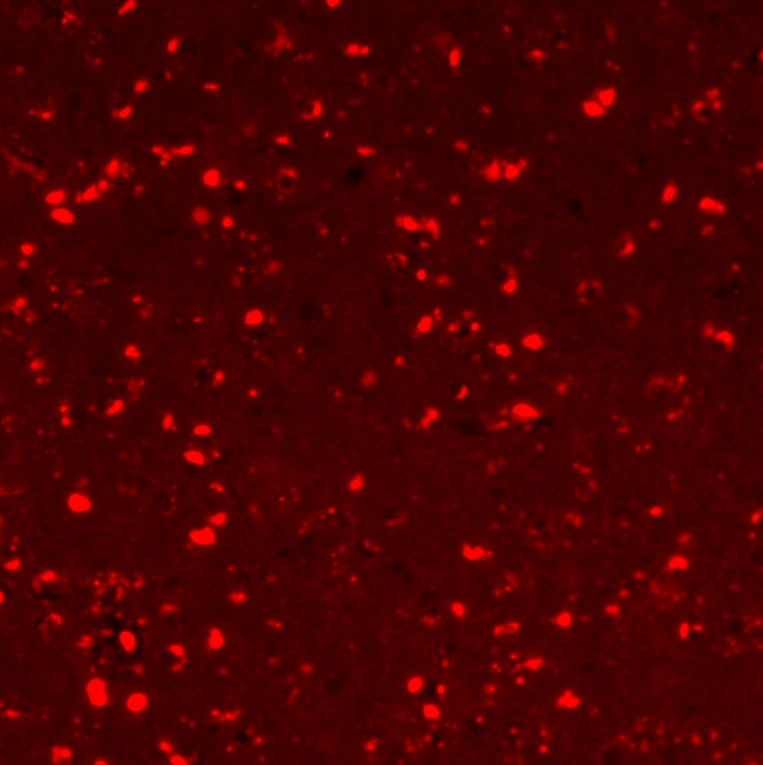

Supplement: Supplementary file 5 — Source data Fig. 1 [file 44318_2026_818_MOESM5_ESM.zip › Figure 1/Figure 1E/AD 1-4/AD #1-Aβ.tif]

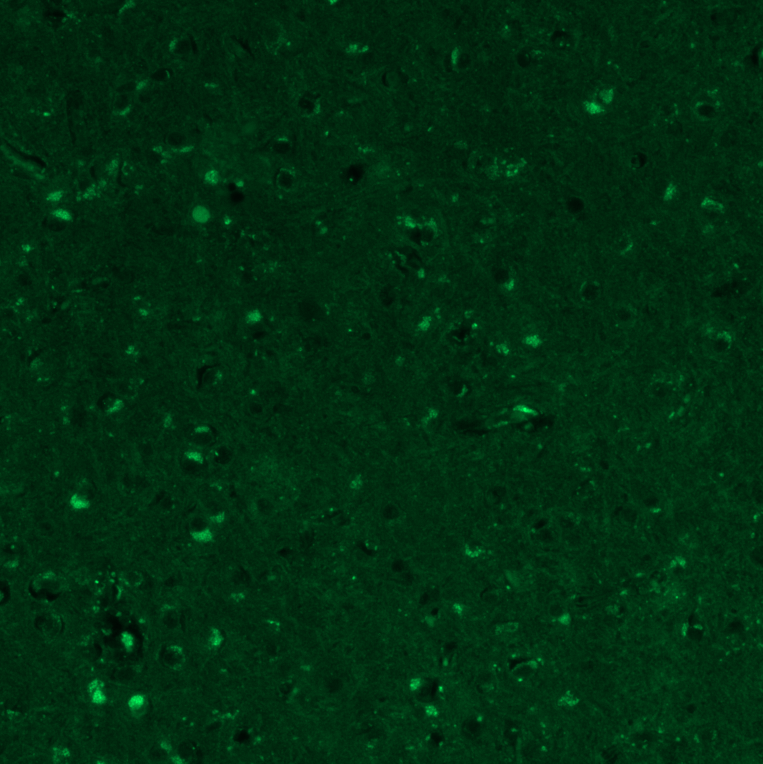

Supplement: Supplementary file 5 — Source data Fig. 1 [file 44318_2026_818_MOESM5_ESM.zip › Figure 1/Figure 1E/AD 1-4/AD #1-FAM134B.tif]

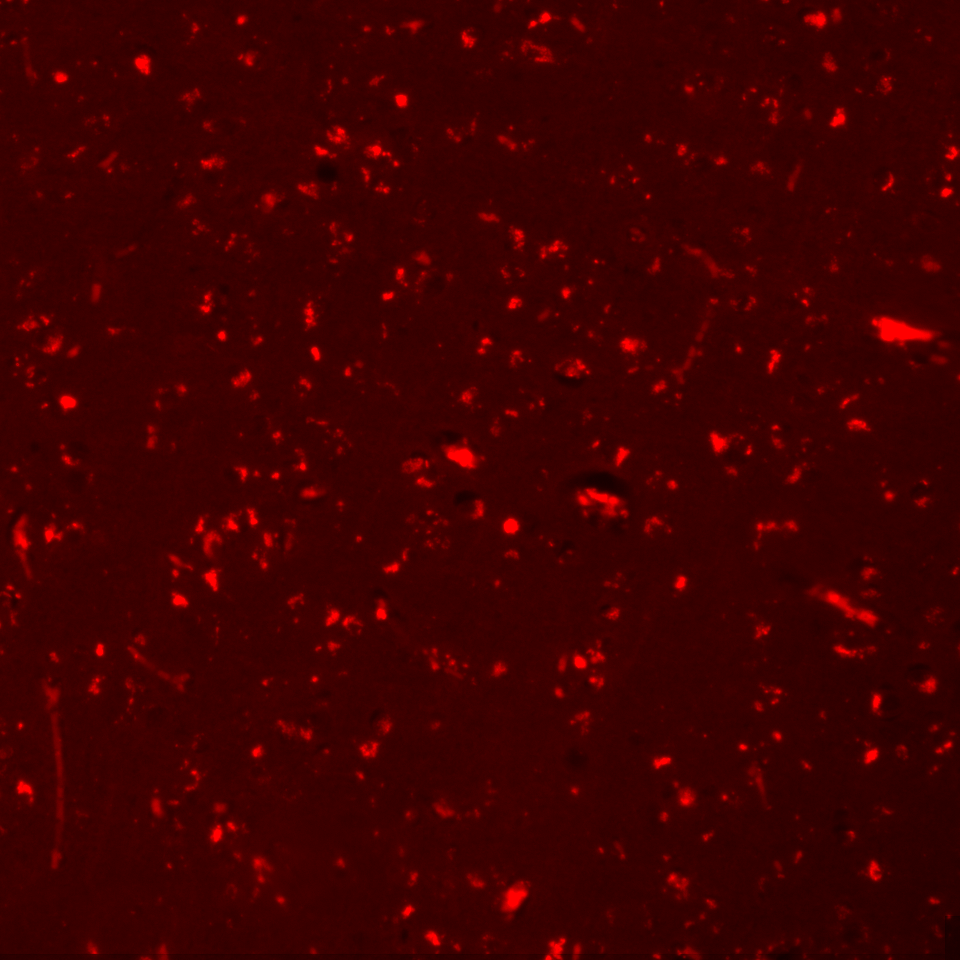

Supplement: Supplementary file 5 — Source data Fig. 1 [file 44318_2026_818_MOESM5_ESM.zip › Figure 1/Figure 1E/AD 1-4/AD #2-Aβ.tif]

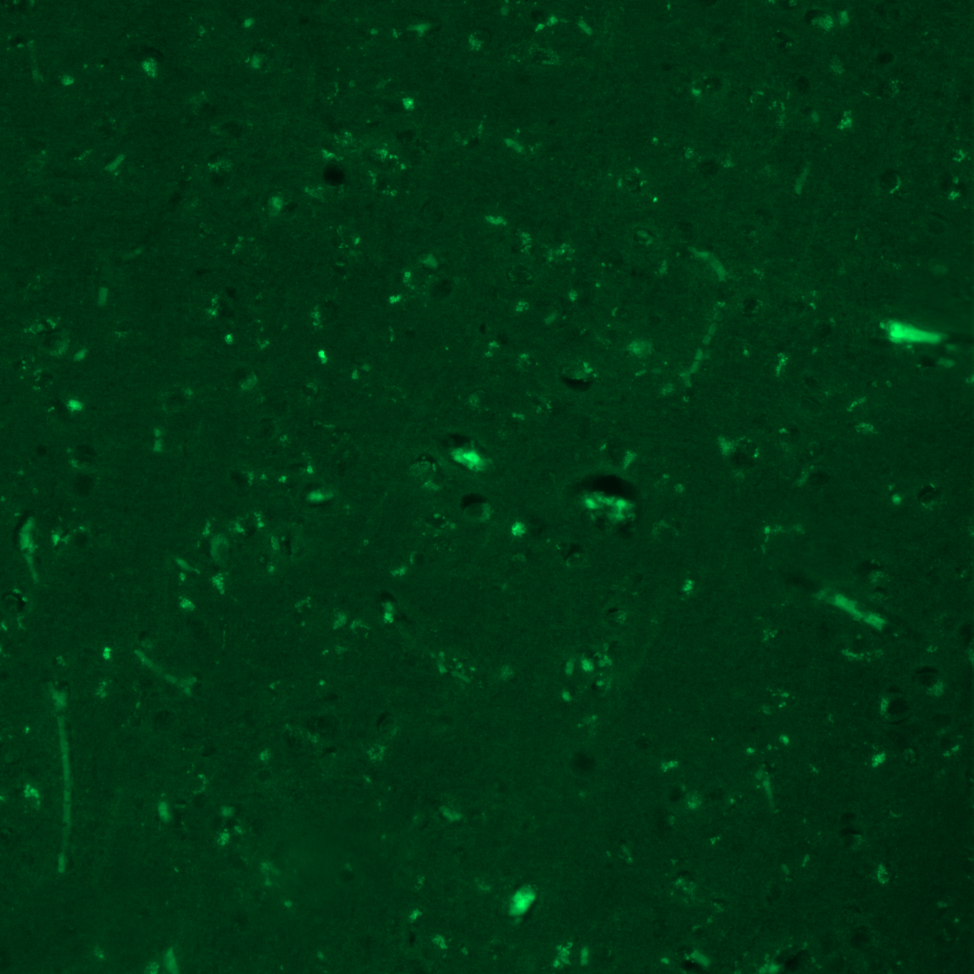

Supplement: Supplementary file 5 — Source data Fig. 1 [file 44318_2026_818_MOESM5_ESM.zip › Figure 1/Figure 1E/AD 1-4/AD #2-FAM134B_EGFP.tif]

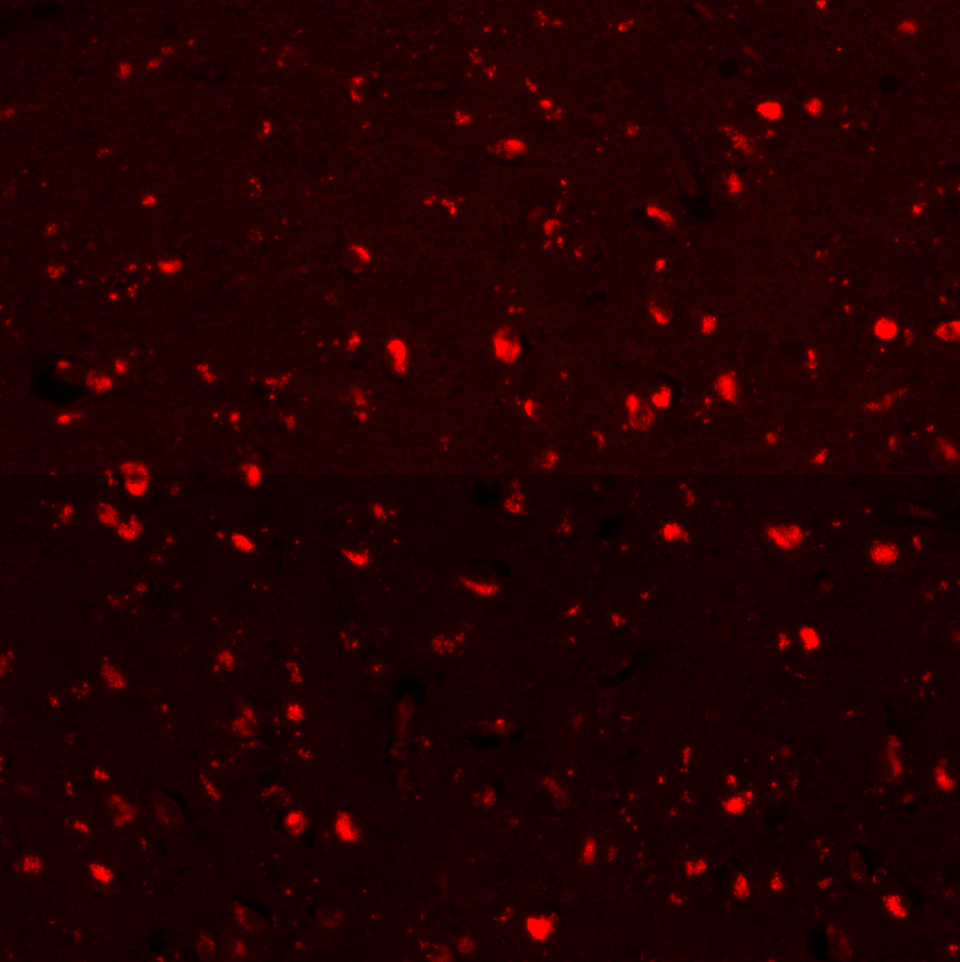

Supplement: Supplementary file 5 — Source data Fig. 1 [file 44318_2026_818_MOESM5_ESM.zip › Figure 1/Figure 1E/AD 1-4/AD #3-Aβ.tif]

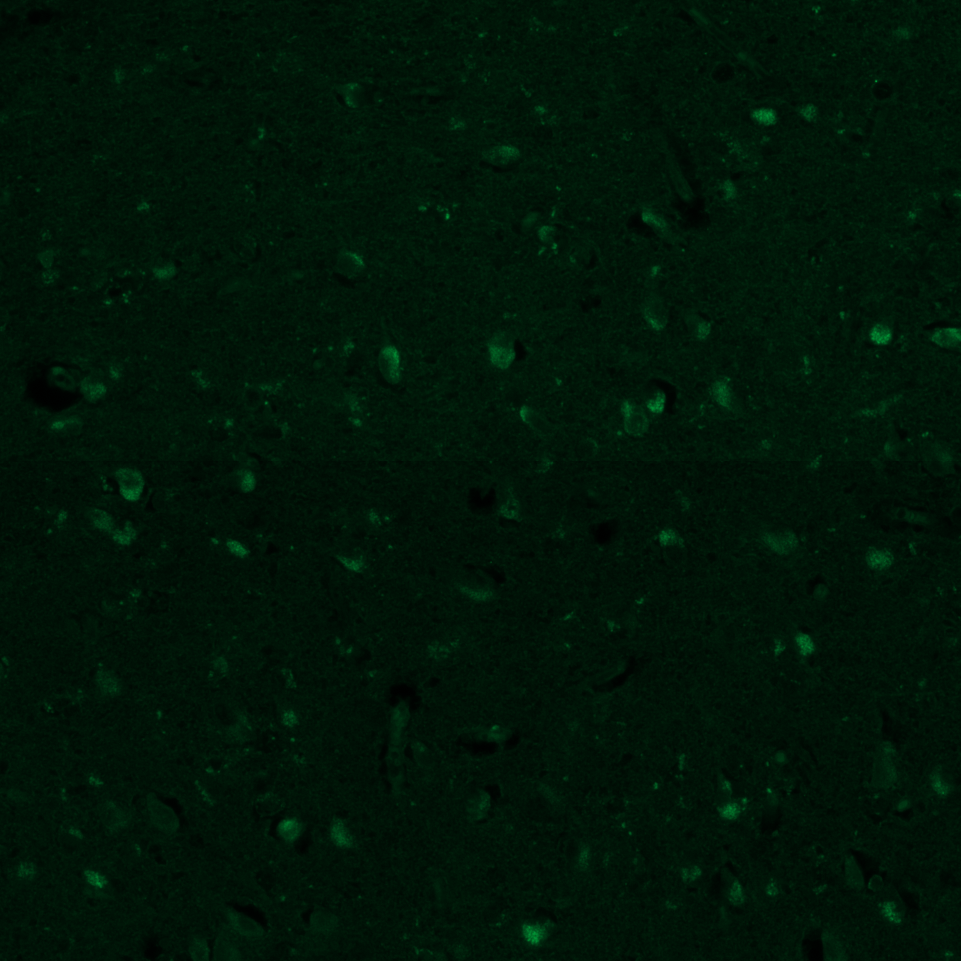

Supplement: Supplementary file 5 — Source data Fig. 1 [file 44318_2026_818_MOESM5_ESM.zip › Figure 1/Figure 1E/AD 1-4/AD #3-FAM134B.tif]

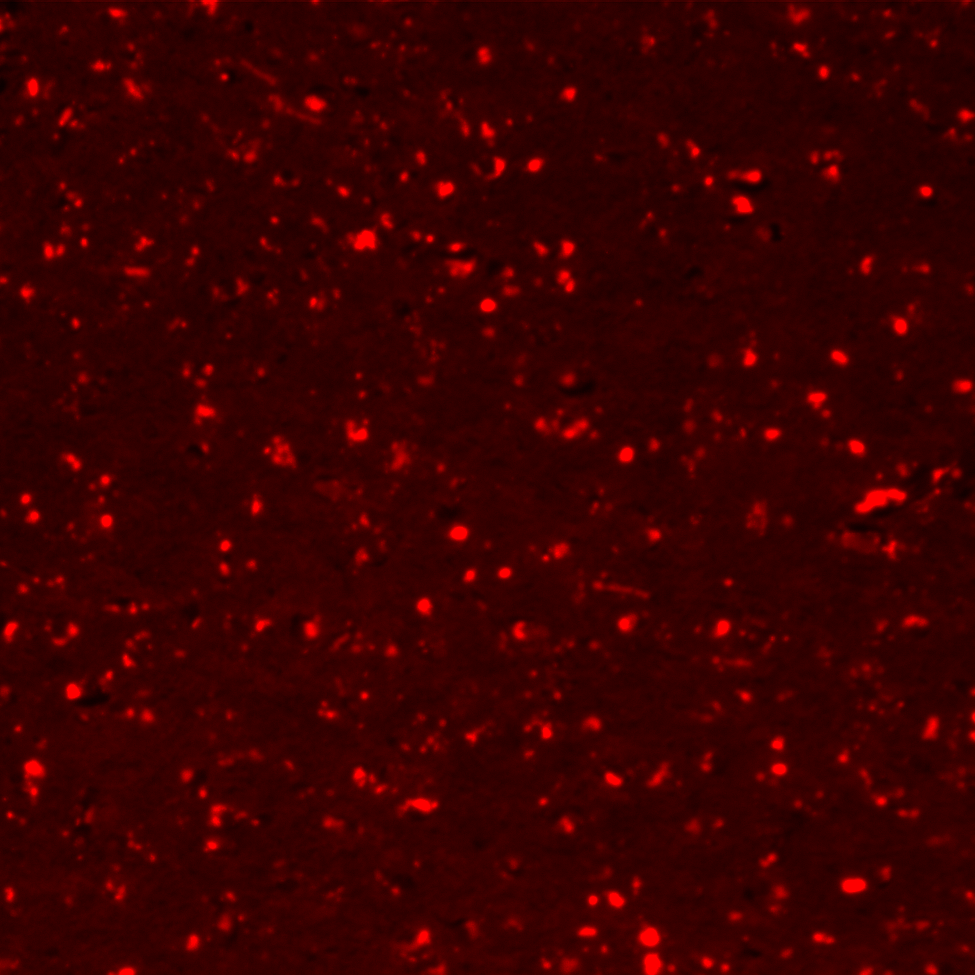

Supplement: Supplementary file 5 — Source data Fig. 1 [file 44318_2026_818_MOESM5_ESM.zip › Figure 1/Figure 1E/AD 1-4/AD #4-Aβ.tif]

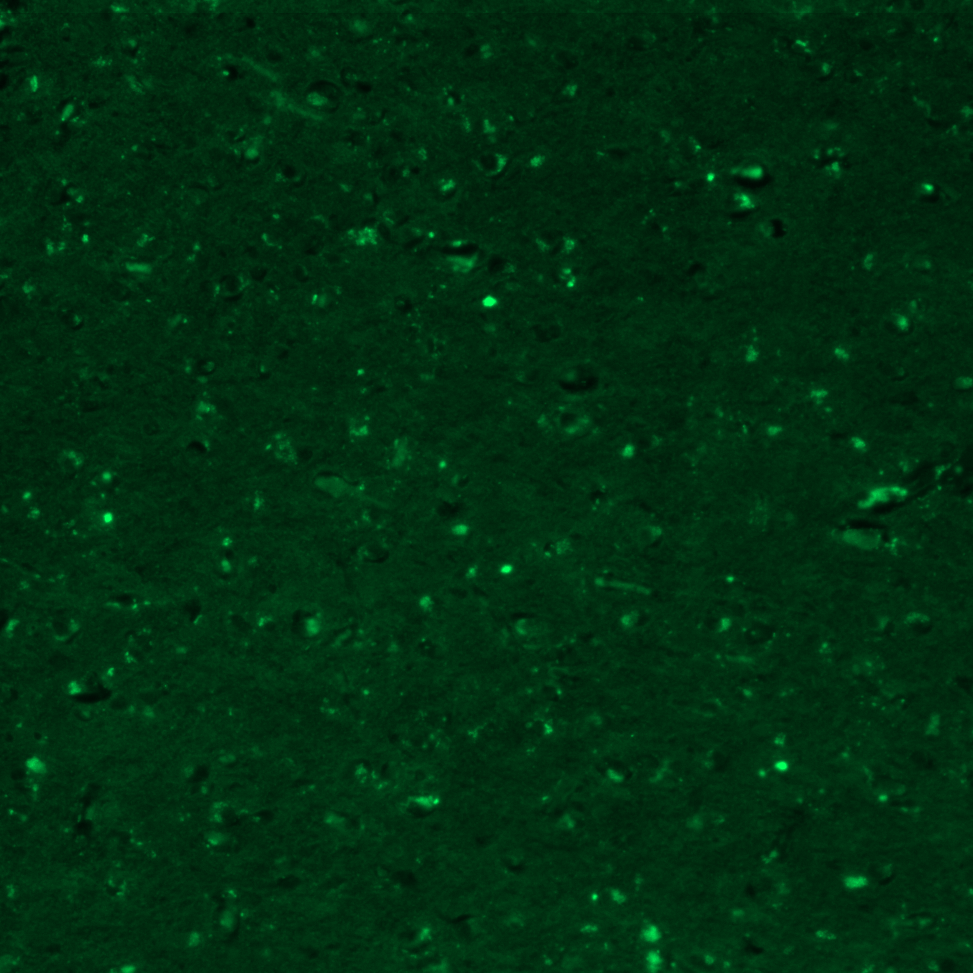

Supplement: Supplementary file 5 — Source data Fig. 1 [file 44318_2026_818_MOESM5_ESM.zip › Figure 1/Figure 1E/AD 1-4/AD #4-FAM134B.tif]

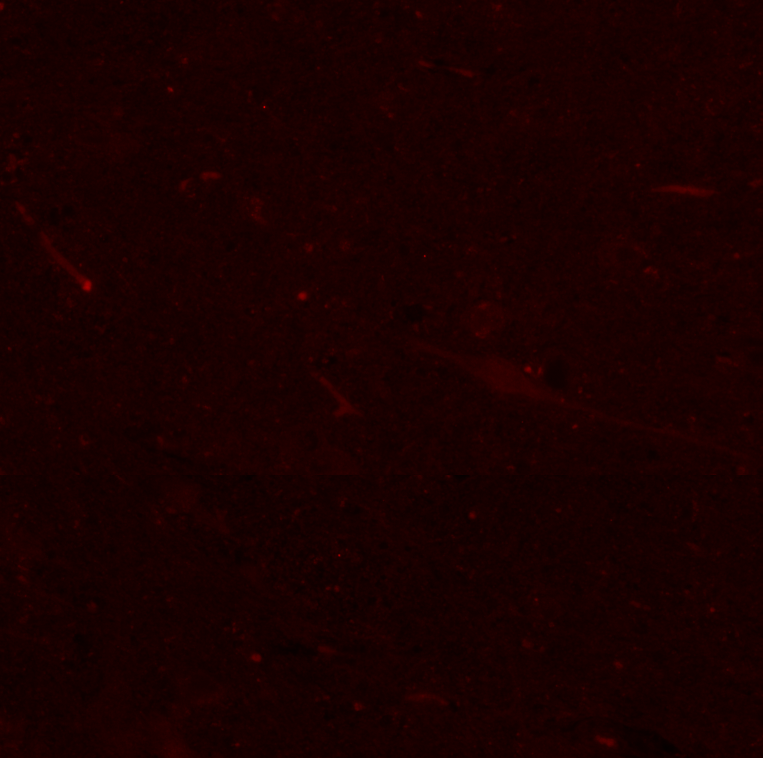

Supplement: Supplementary file 5 — Source data Fig. 1 [file 44318_2026_818_MOESM5_ESM.zip › Figure 1/Figure 1E/Control 1-3/Control #1-Aβ.tif]

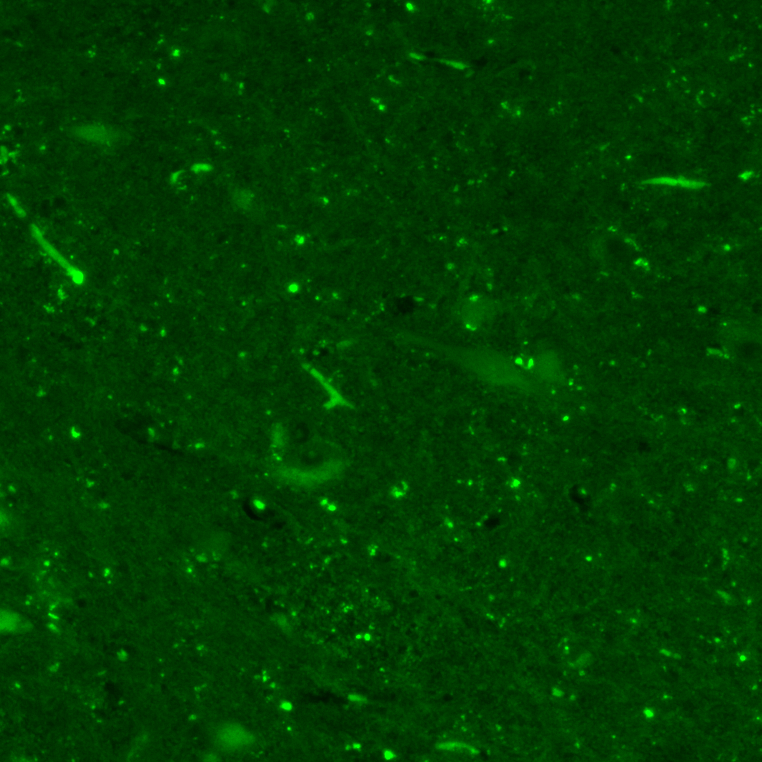

Supplement: Supplementary file 5 — Source data Fig. 1 [file 44318_2026_818_MOESM5_ESM.zip › Figure 1/Figure 1E/Control 1-3/Control #1-FAM134B.tif]

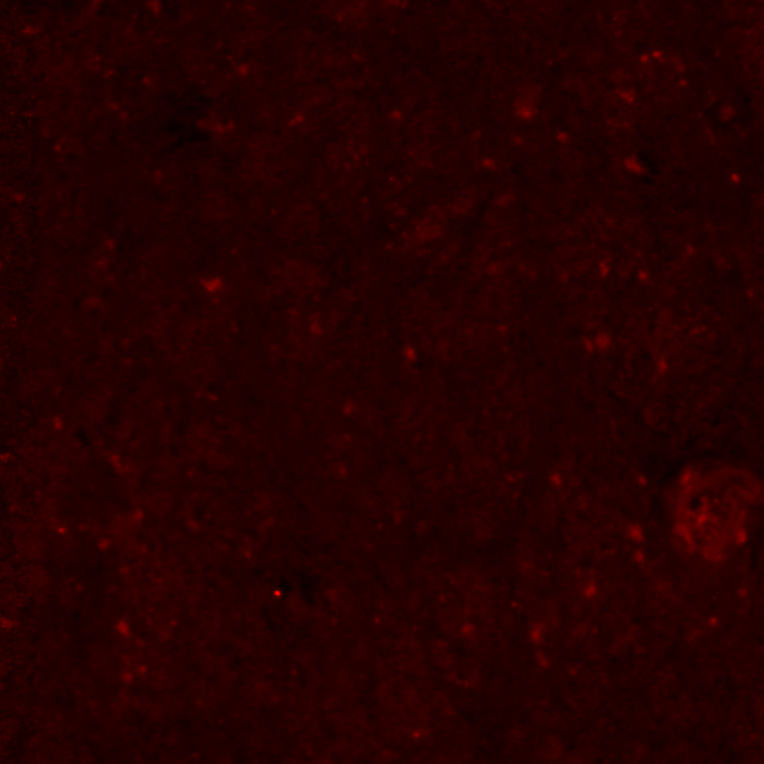

Supplement: Supplementary file 5 — Source data Fig. 1 [file 44318_2026_818_MOESM5_ESM.zip › Figure 1/Figure 1E/Control 1-3/Control #2-Aβ.tif]

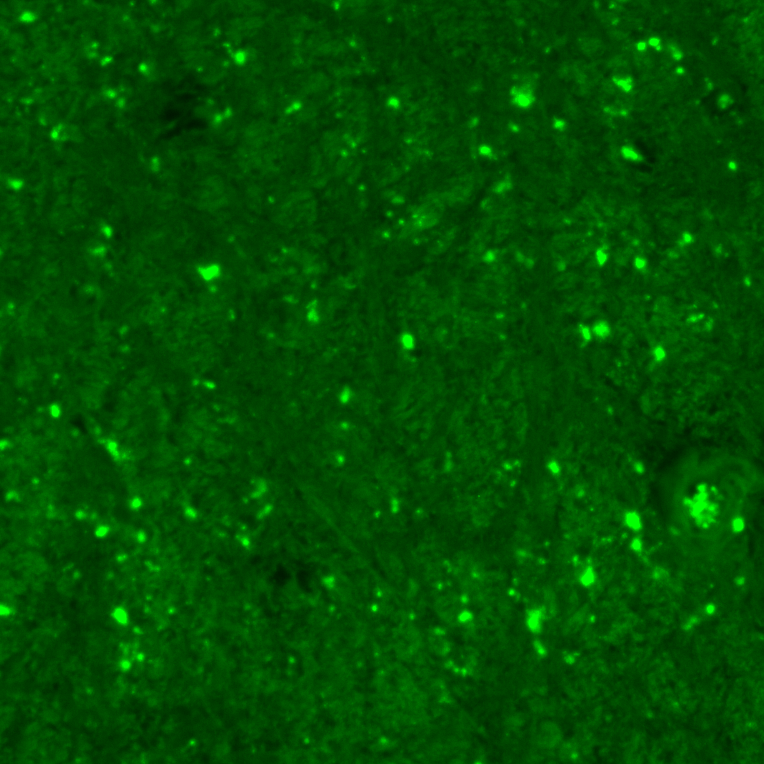

Supplement: Supplementary file 5 — Source data Fig. 1 [file 44318_2026_818_MOESM5_ESM.zip › Figure 1/Figure 1E/Control 1-3/Control #2-FAM134B.tif]

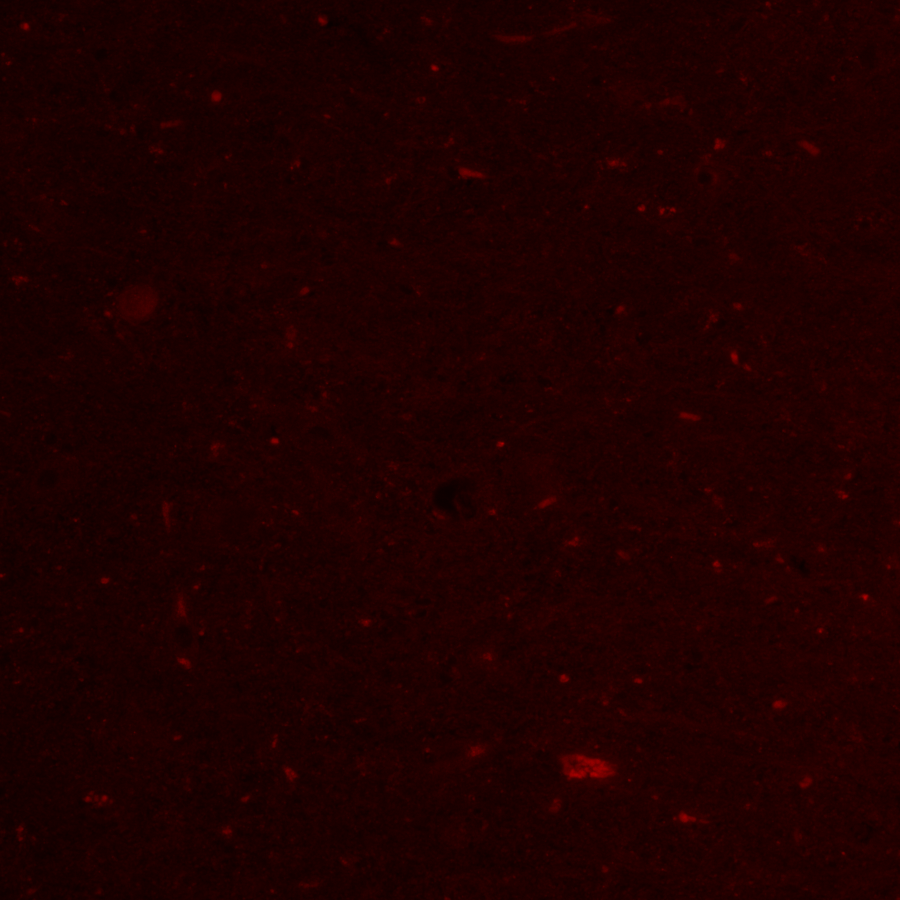

Supplement: Supplementary file 5 — Source data Fig. 1 [file 44318_2026_818_MOESM5_ESM.zip › Figure 1/Figure 1E/Control 1-3/Control #3-Aβ.tif]

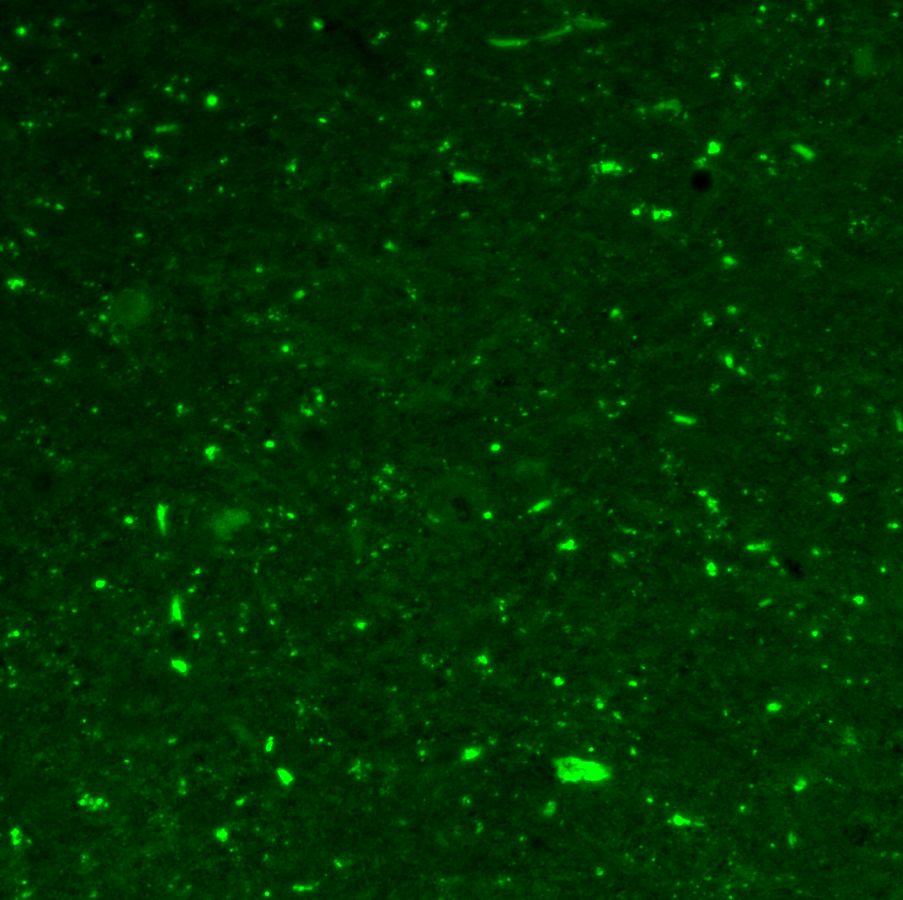

Supplement: Supplementary file 5 — Source data Fig. 1 [file 44318_2026_818_MOESM5_ESM.zip › Figure 1/Figure 1E/Control 1-3/Control #3-FAM134B.tif]

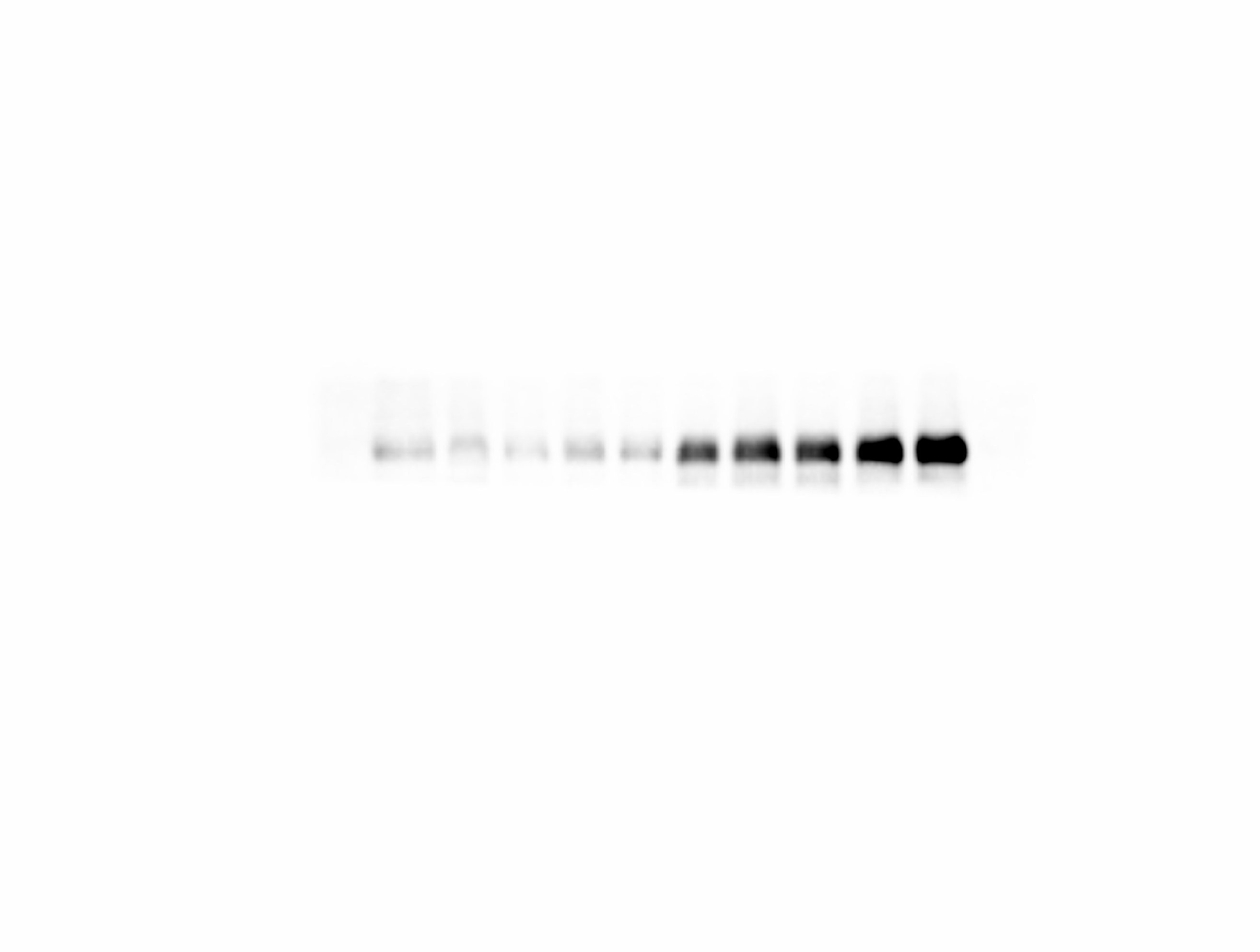

Supplement: Supplementary file 5 — Source data Fig. 1 [file 44318_2026_818_MOESM5_ESM.zip › Figure 1/Figure 1G/APP.tif]

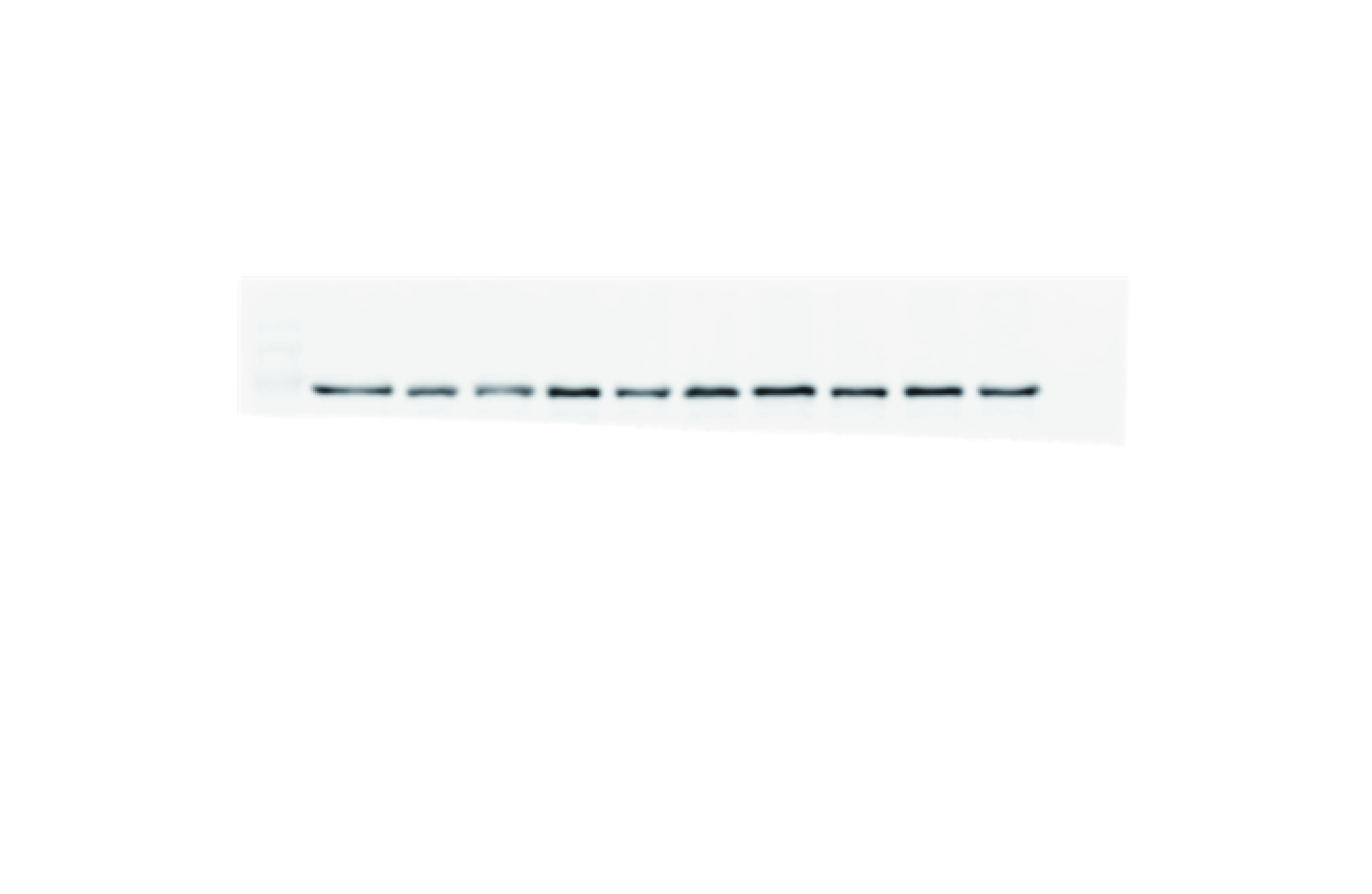

Supplement: Supplementary file 5 — Source data Fig. 1 [file 44318_2026_818_MOESM5_ESM.zip › Figure 1/Figure 1G/Calnexin.tif]

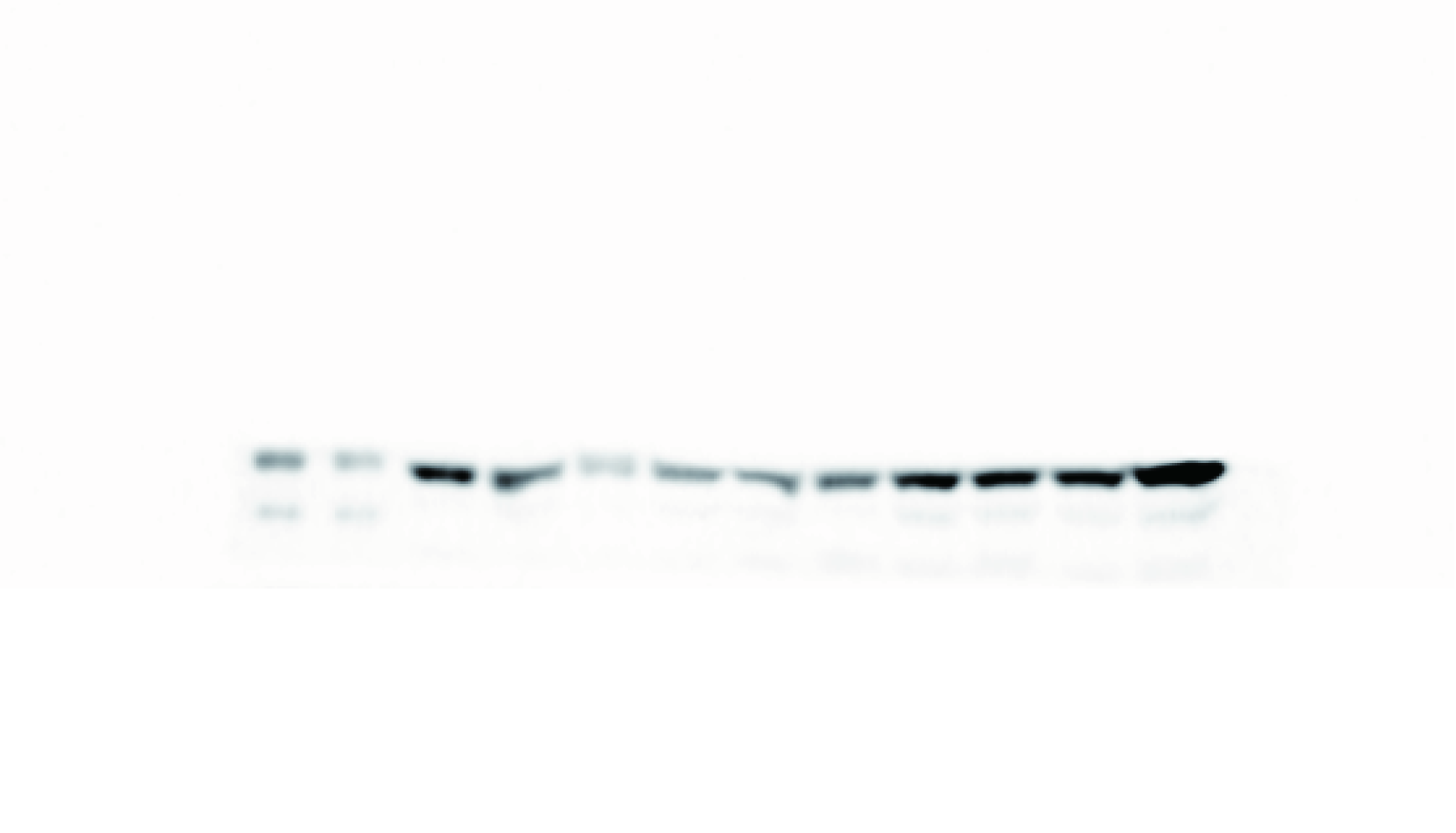

Supplement: Supplementary file 5 — Source data Fig. 1 [file 44318_2026_818_MOESM5_ESM.zip › Figure 1/Figure 1G/Climp63.tif]

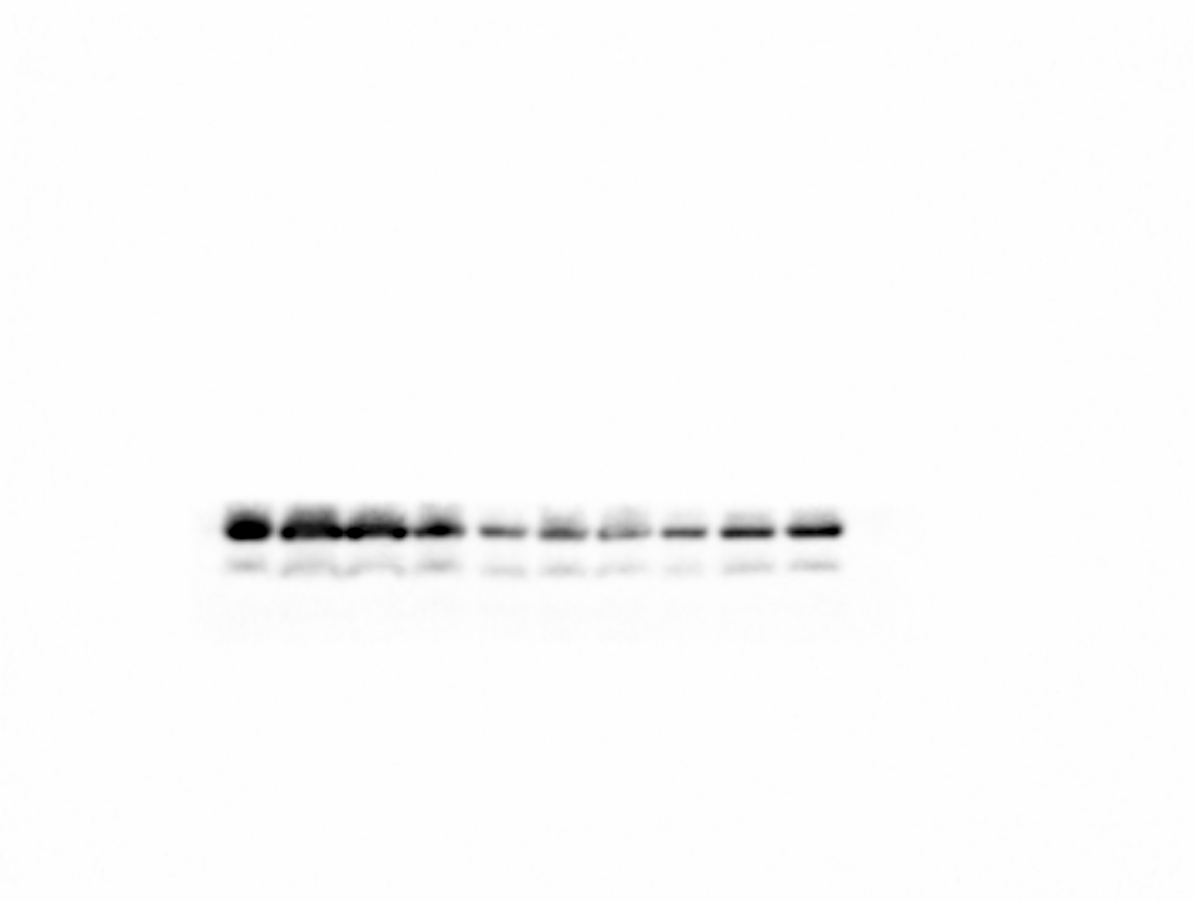

Supplement: Supplementary file 5 — Source data Fig. 1 [file 44318_2026_818_MOESM5_ESM.zip › Figure 1/Figure 1G/Fam134b.tif]

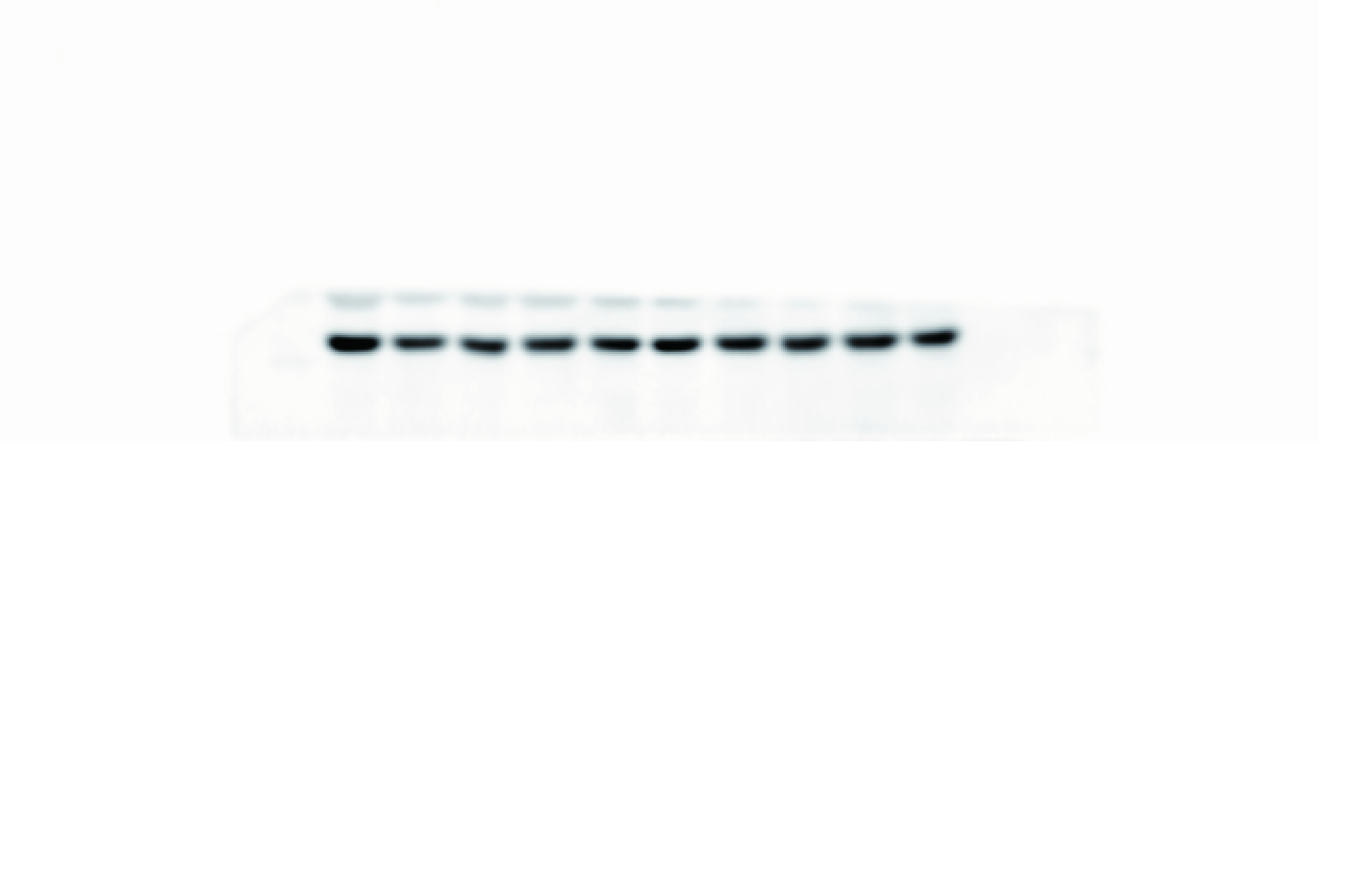

Supplement: Supplementary file 5 — Source data Fig. 1 [file 44318_2026_818_MOESM5_ESM.zip › Figure 1/Figure 1G/Gapdh.tif]

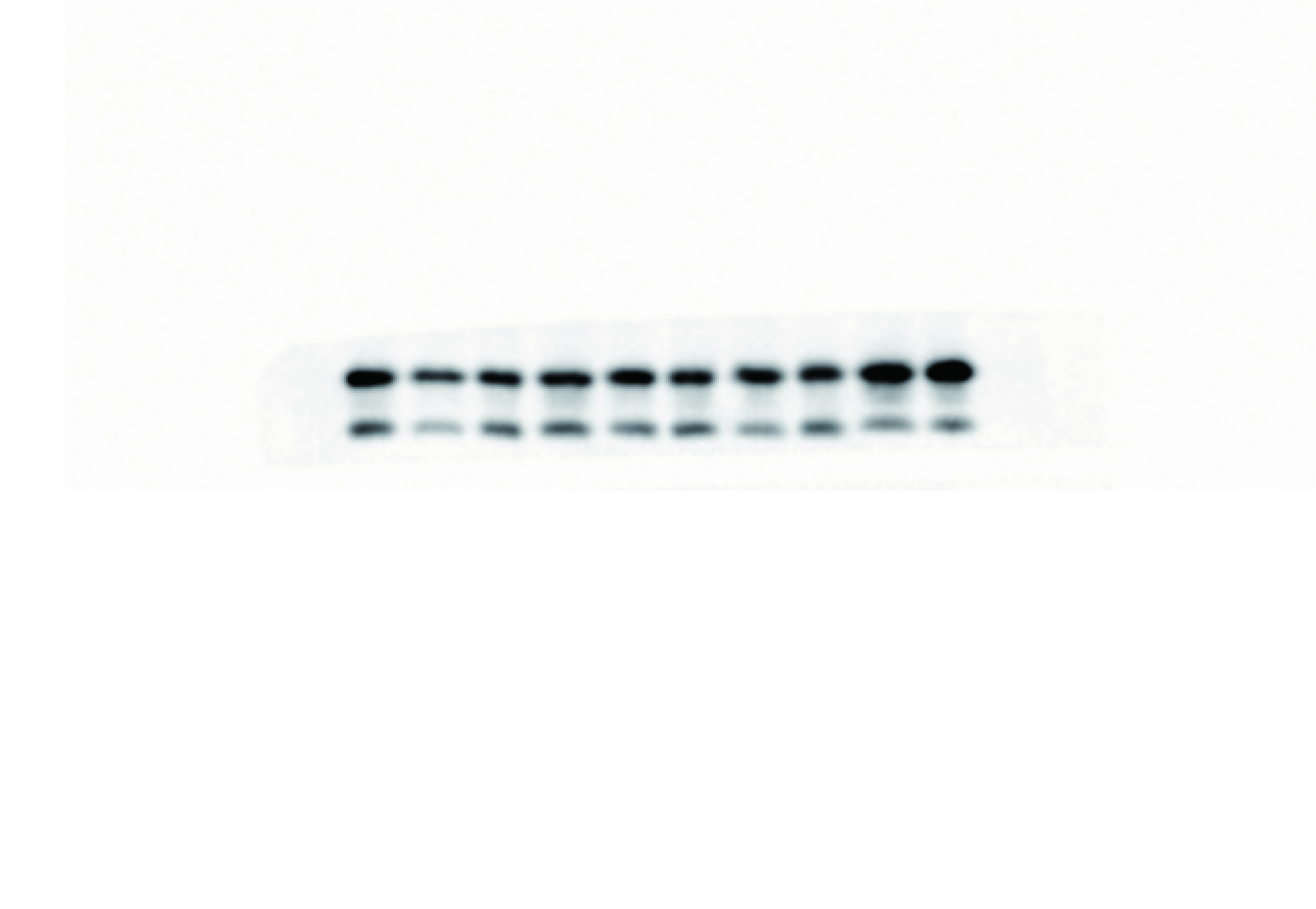

Supplement: Supplementary file 5 — Source data Fig. 1 [file 44318_2026_818_MOESM5_ESM.zip › Figure 1/Figure 1G/Lc3b.tif]

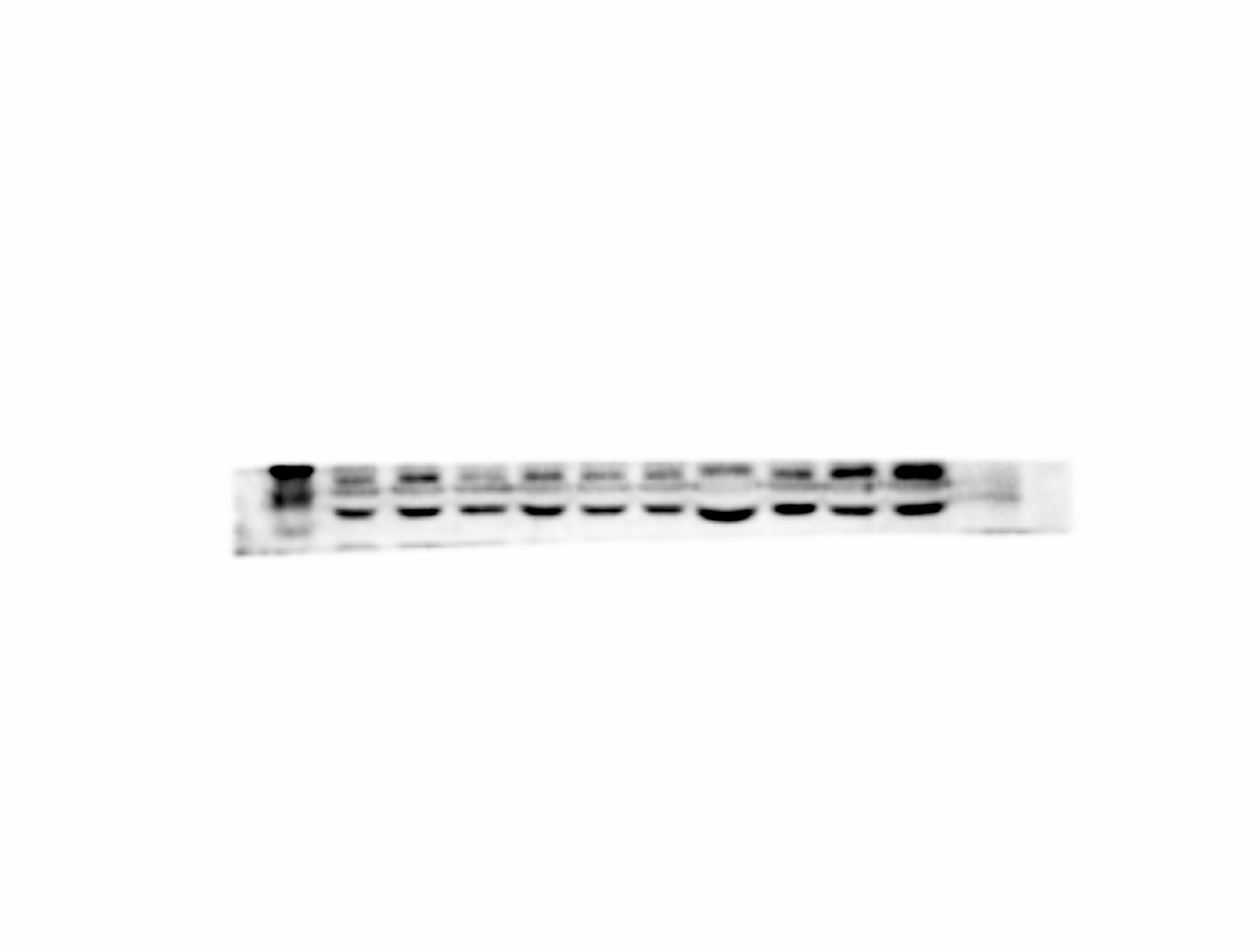

Supplement: Supplementary file 5 — Source data Fig. 1 [file 44318_2026_818_MOESM5_ESM.zip › Figure 1/Figure 1G/p62 (down).tif]

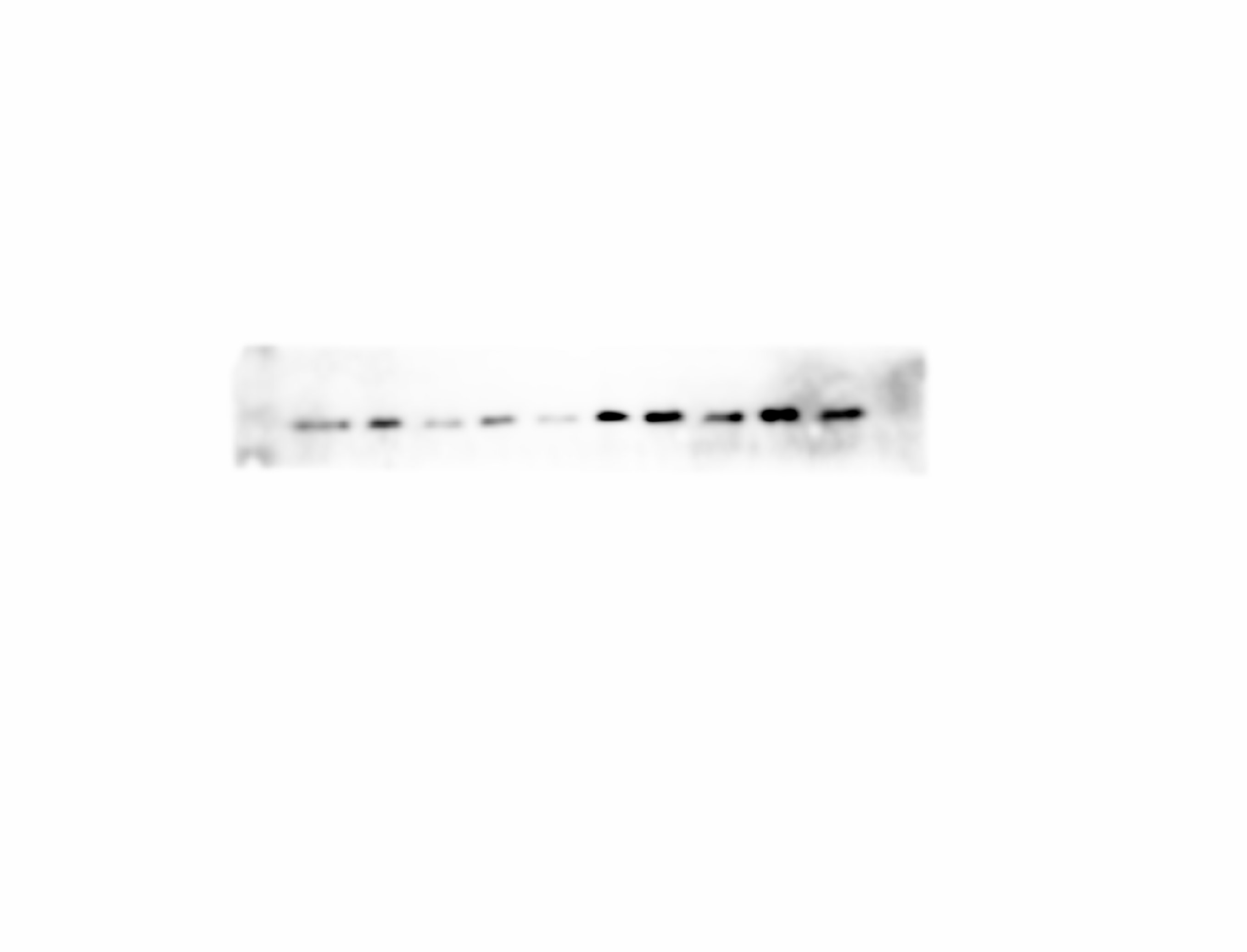

Supplement: Supplementary file 5 — Source data Fig. 1 [file 44318_2026_818_MOESM5_ESM.zip › Figure 1/Figure 1G/Reep5.tif]

Figure 1G

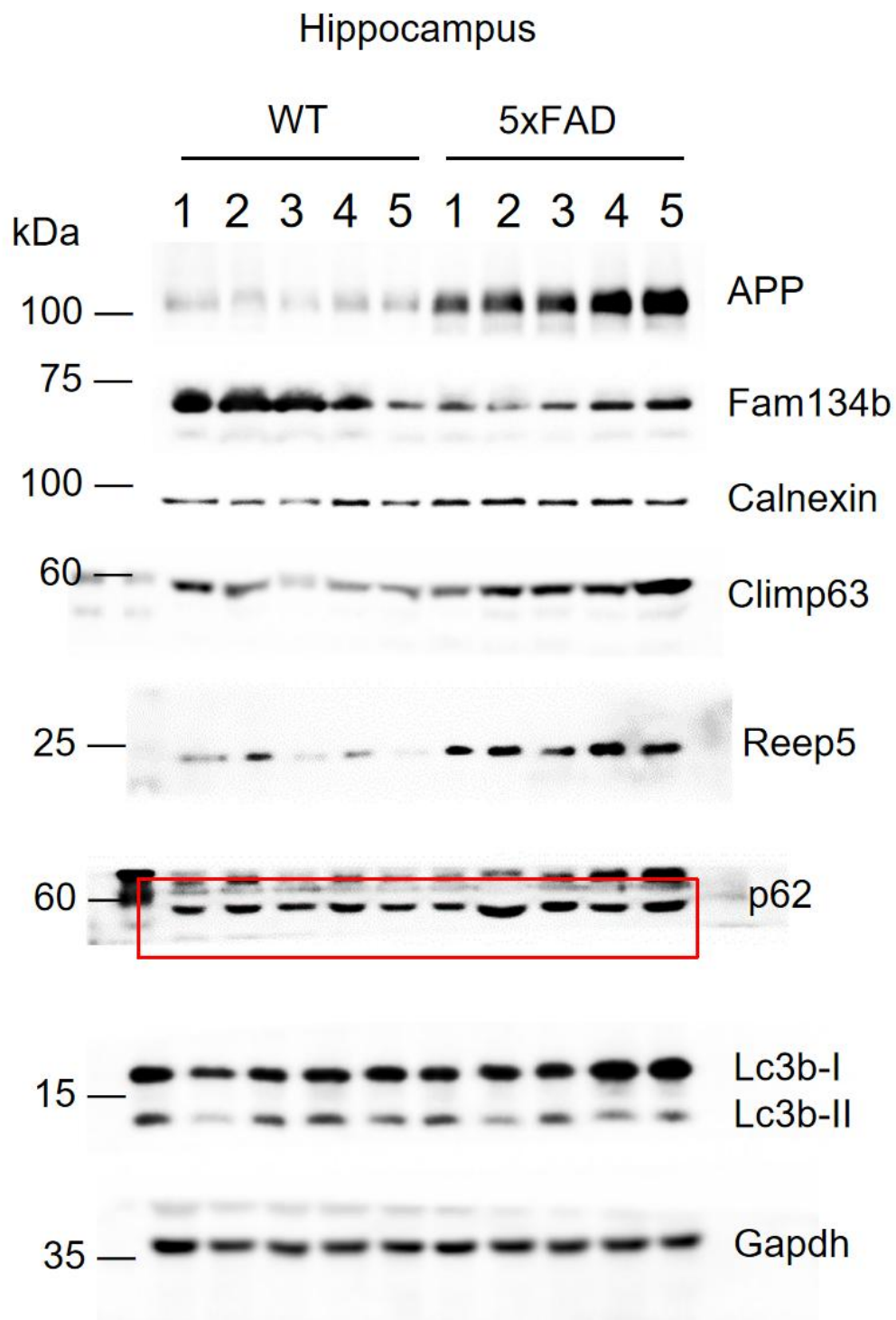

Supplement: Supplementary file 5 — Source data Fig. 1 [file 44318_2026_818_MOESM5_ESM.zip › Figure 1/Figure 1G/WB for Figure 1G.pdf]

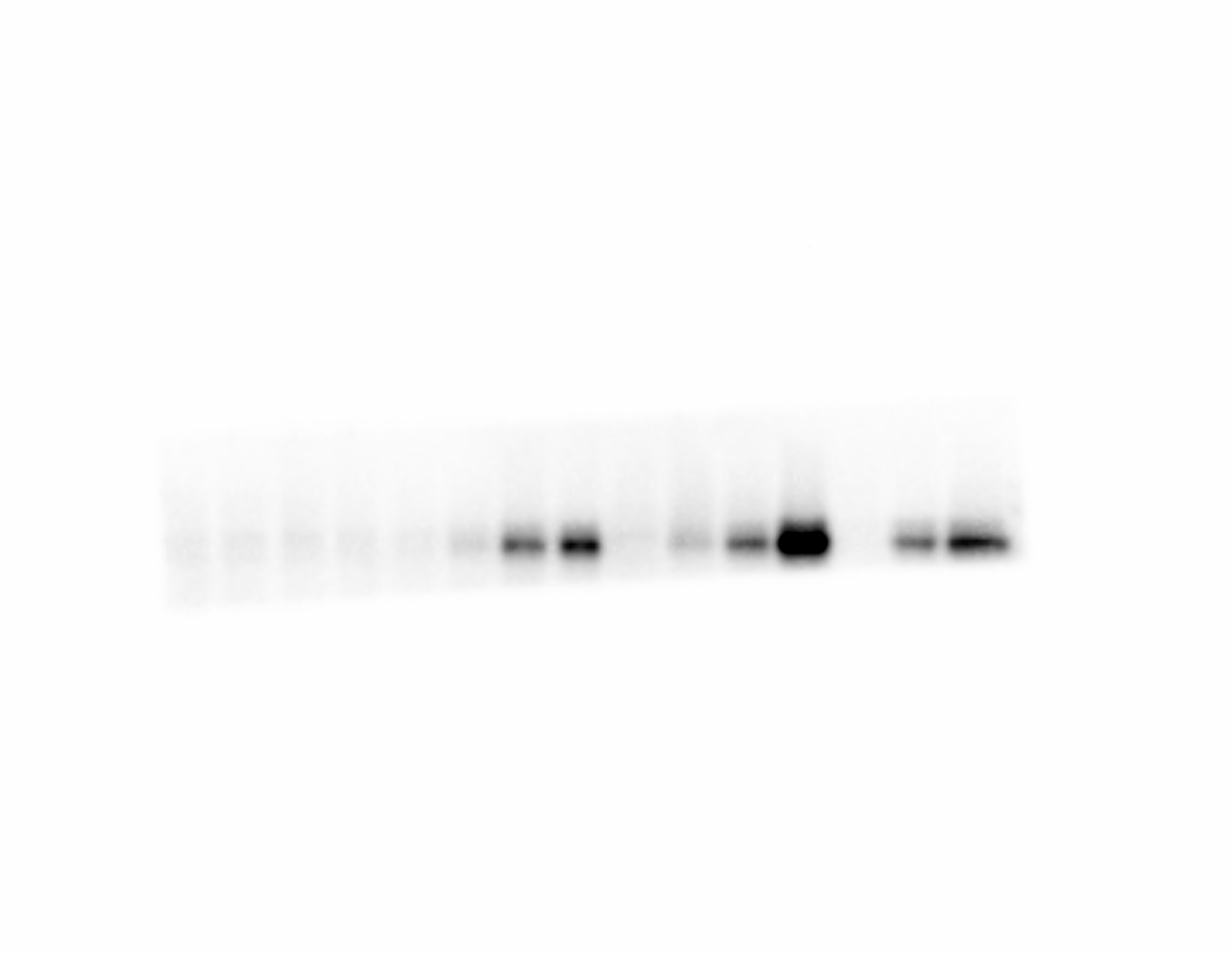

Supplement: Supplementary file 5 — Source data Fig. 1 [file 44318_2026_818_MOESM5_ESM.zip › Figure 1/Figure 1I/APP (lane 1-12).tif]

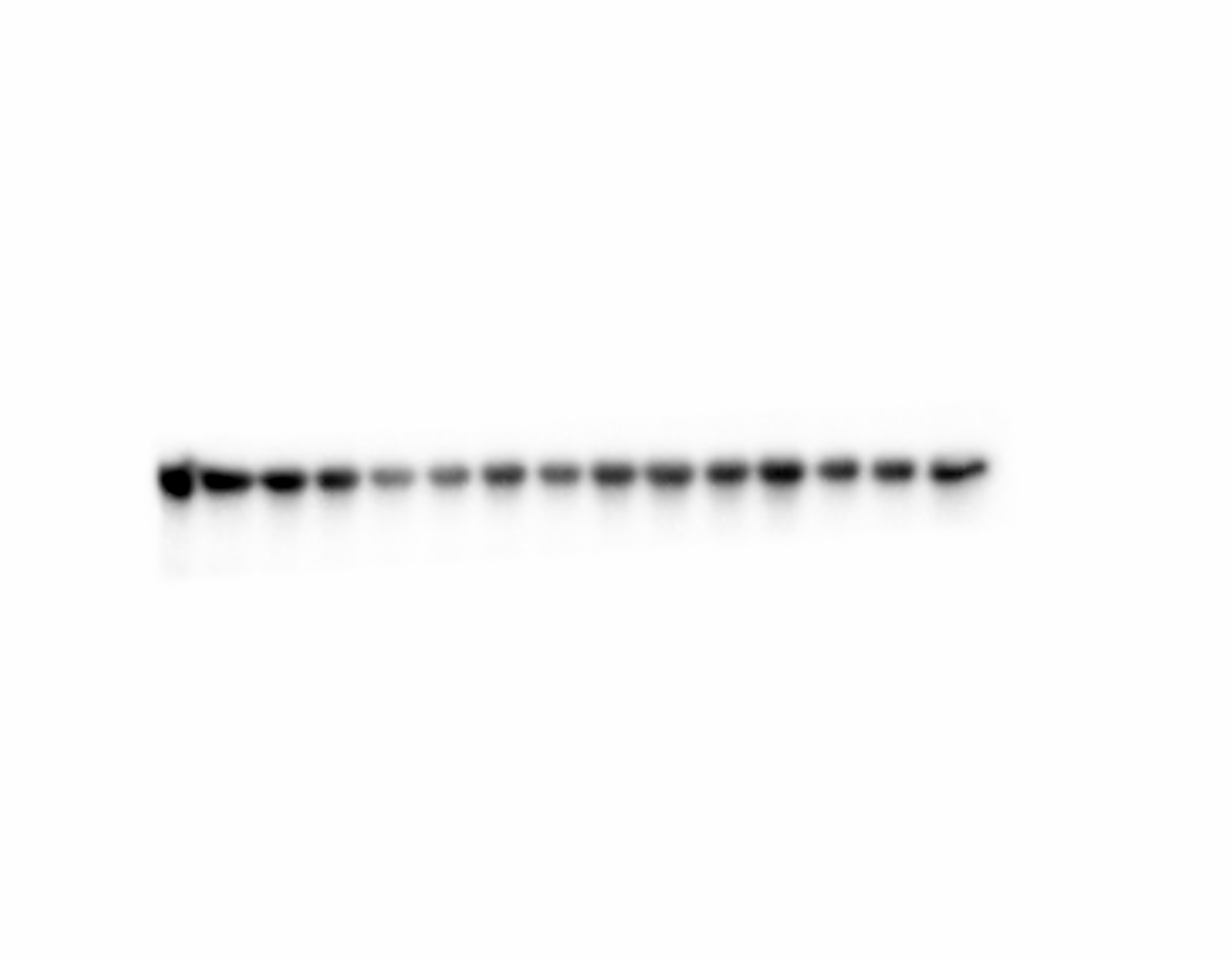

Supplement: Supplementary file 5 — Source data Fig. 1 [file 44318_2026_818_MOESM5_ESM.zip › Figure 1/Figure 1I/GAPDH (lane 1-12).tif]

Figure 1I

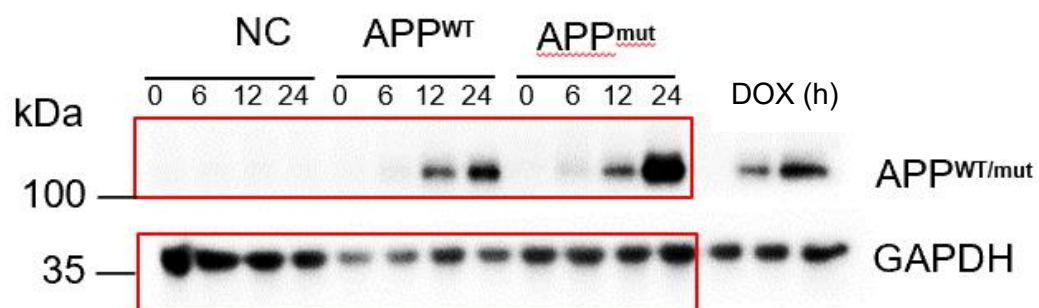

Supplement: Supplementary file 5 — Source data Fig. 1 [file 44318_2026_818_MOESM5_ESM.zip › Figure 1/Figure 1I/WB for Figure 1I.pdf]

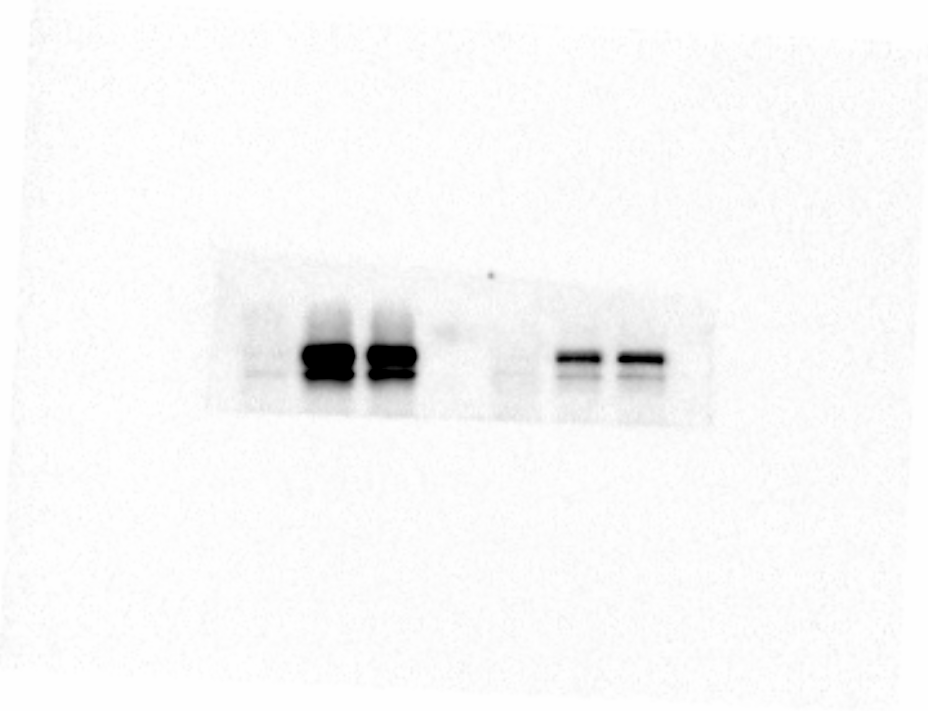

Supplement: Supplementary file 5 — Source data Fig. 1 [file 44318_2026_818_MOESM5_ESM.zip › Figure 1/Figure 1J/APP (lane 4-6).tif]

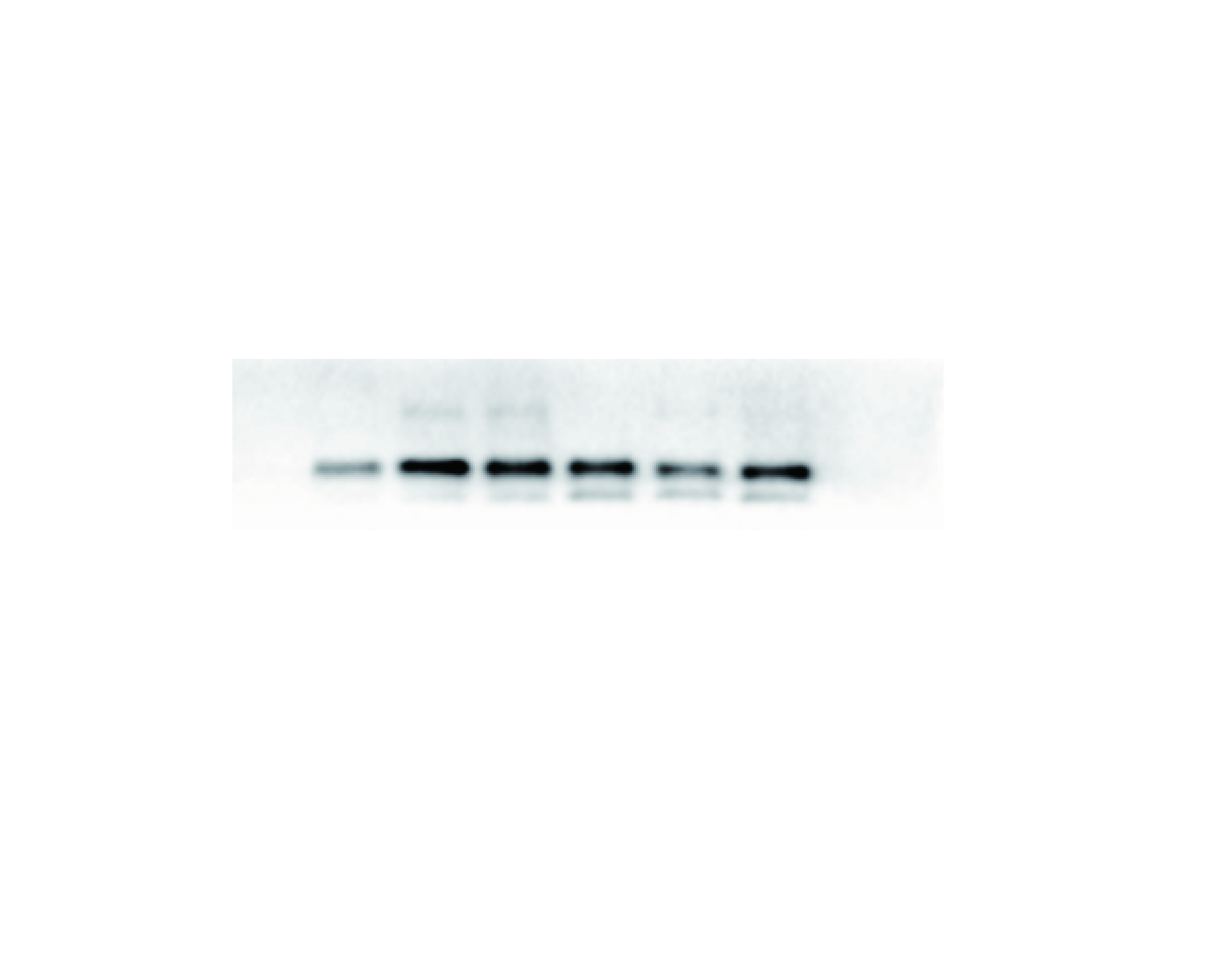

Supplement: Supplementary file 5 — Source data Fig. 1 [file 44318_2026_818_MOESM5_ESM.zip › Figure 1/Figure 1J/CALNEXIN (1-3).tif]

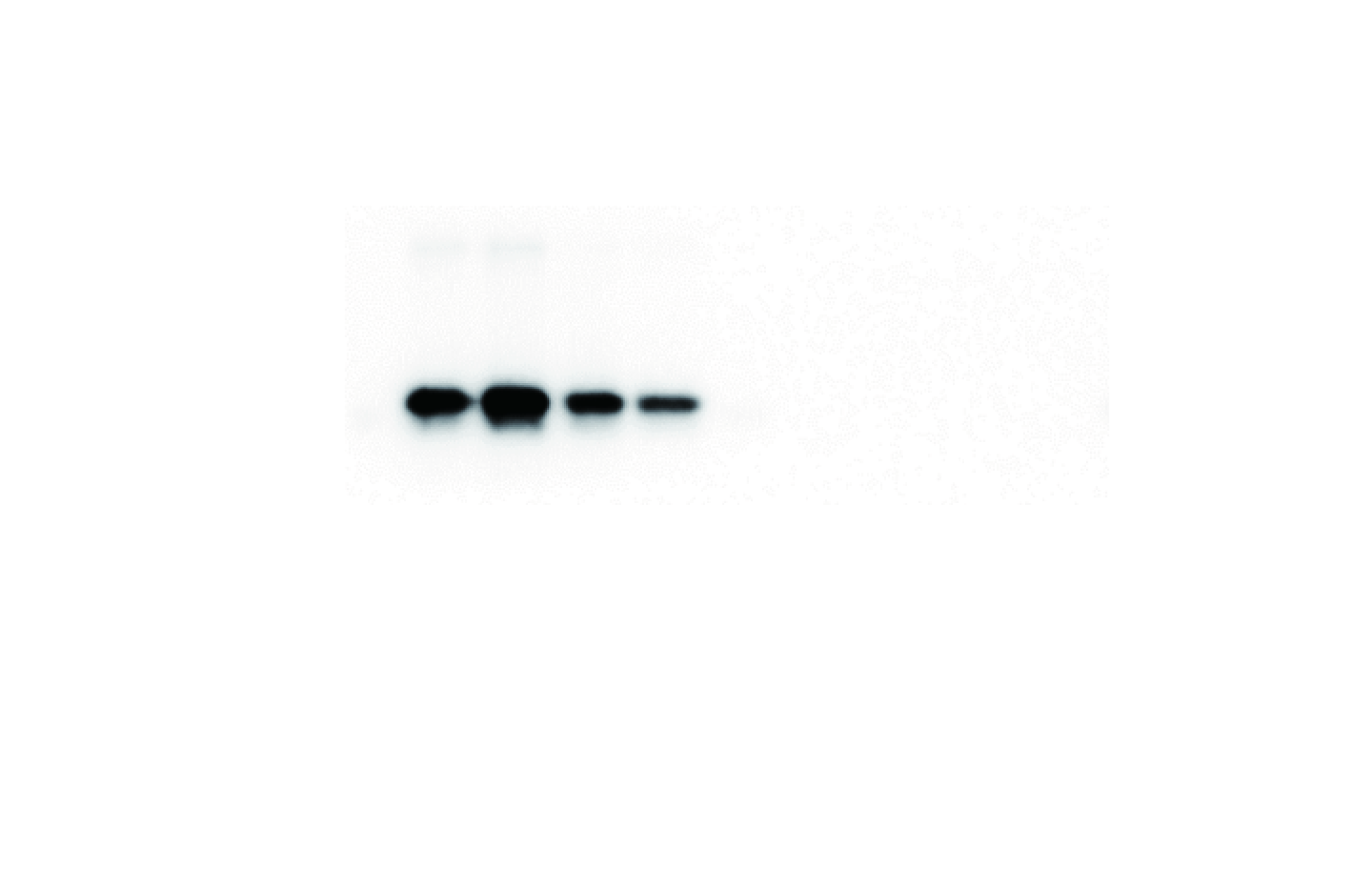

Supplement: Supplementary file 5 — Source data Fig. 1 [file 44318_2026_818_MOESM5_ESM.zip › Figure 1/Figure 1J/FAM134B (lane 2-4).tif]

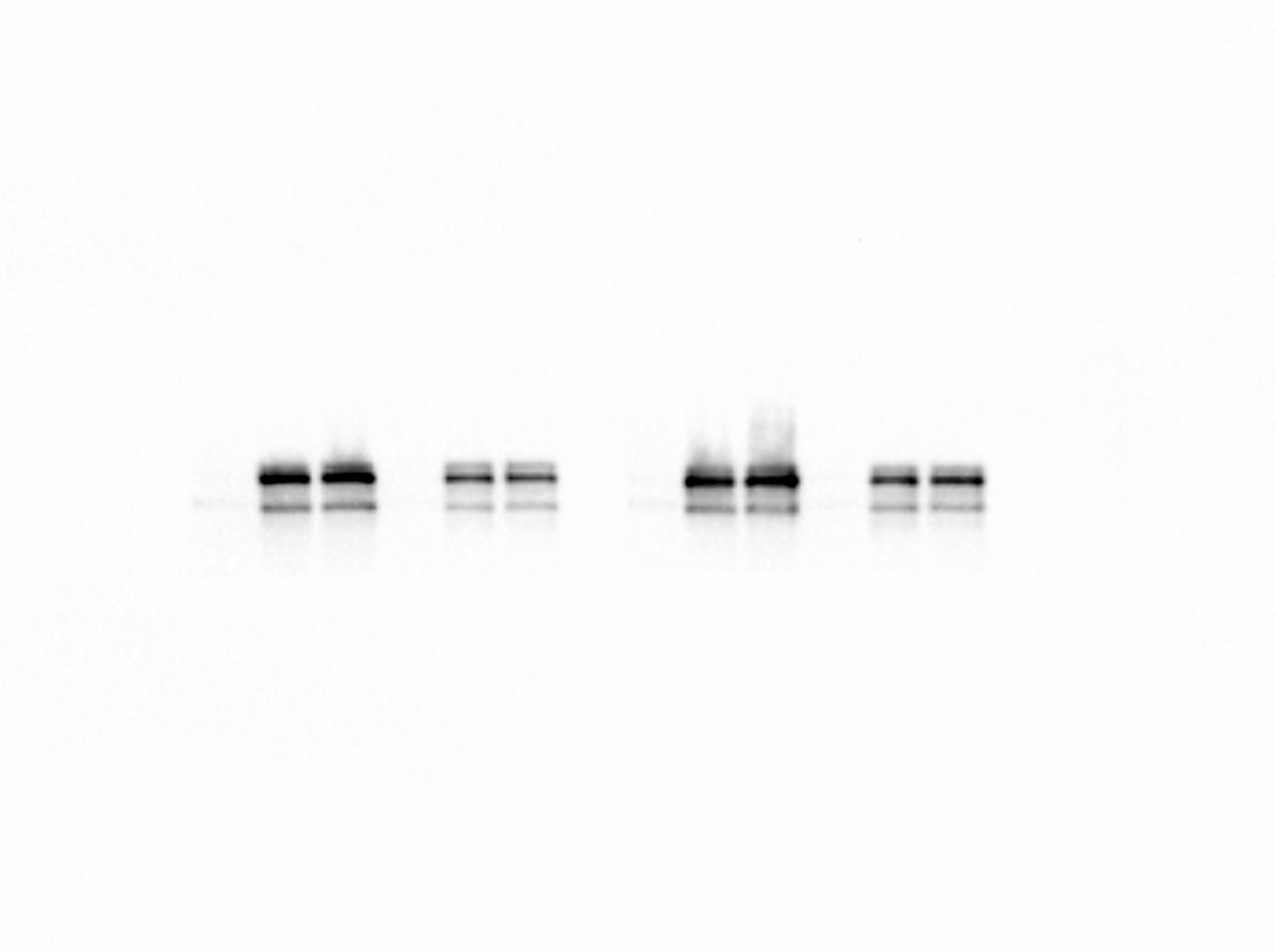

Supplement: Supplementary file 5 — Source data Fig. 1 [file 44318_2026_818_MOESM5_ESM.zip › Figure 1/Figure 1J/Figure 1J Replicate 1/APP (lane 1-3).tif]

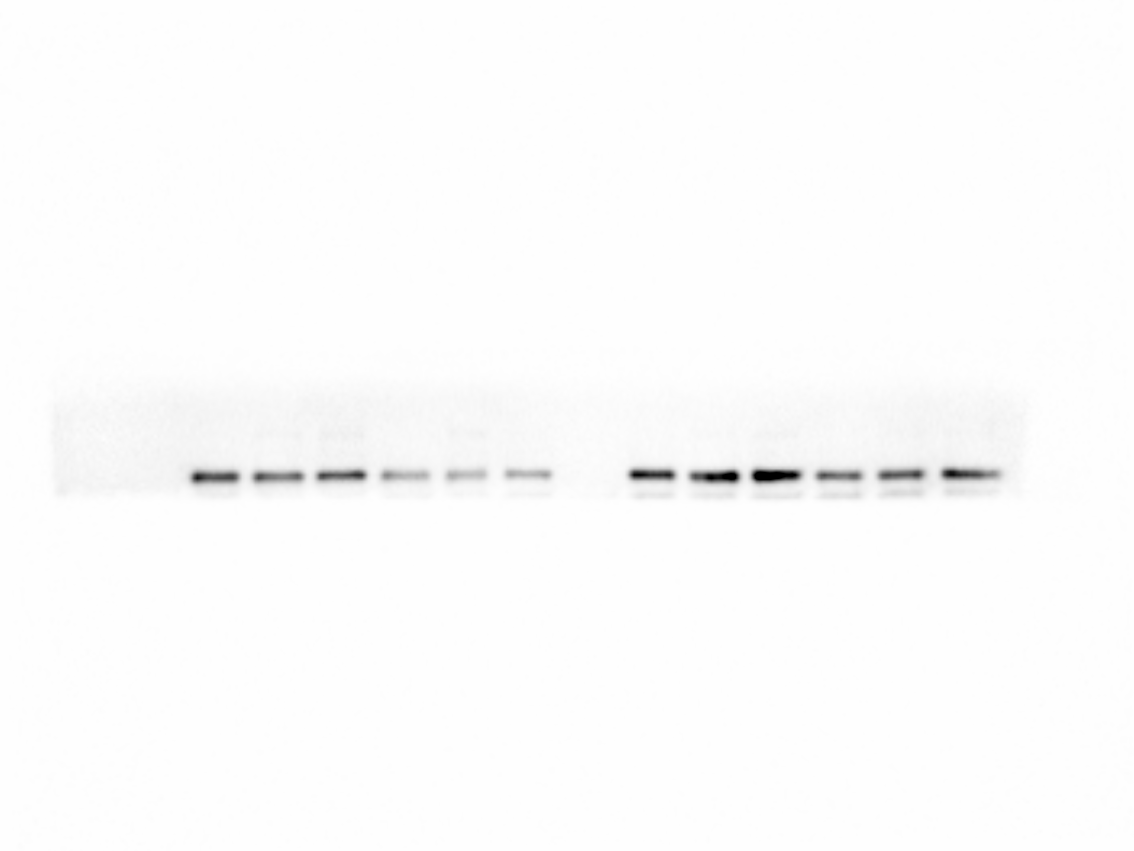

Supplement: Supplementary file 5 — Source data Fig. 1 [file 44318_2026_818_MOESM5_ESM.zip › Figure 1/Figure 1J/Figure 1J Replicate 1/CALNEXIN (lane 1-3).tif]

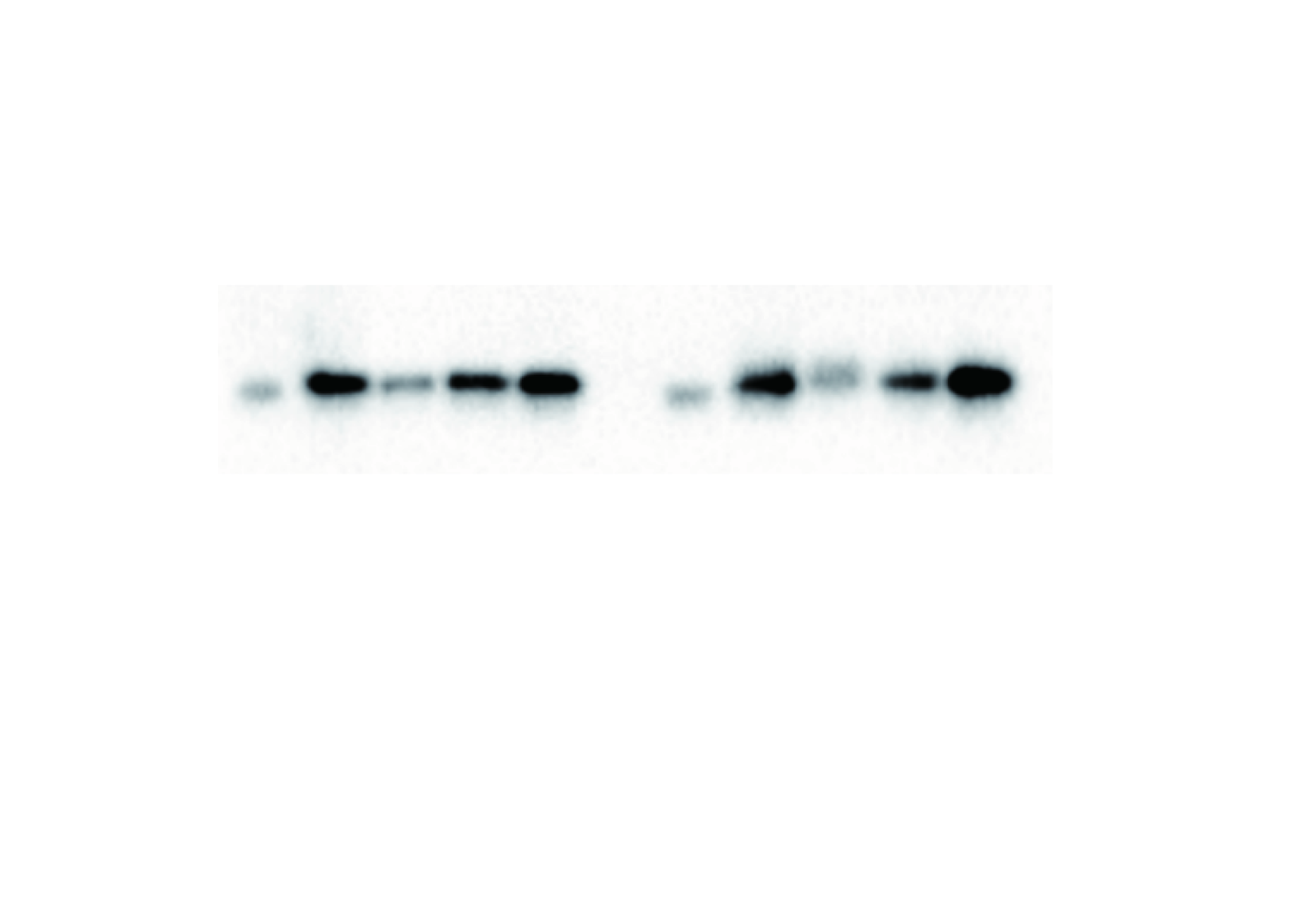

Supplement: Supplementary file 5 — Source data Fig. 1 [file 44318_2026_818_MOESM5_ESM.zip › Figure 1/Figure 1J/Figure 1J Replicate 1/FAM134B (lane 1-3).tif]

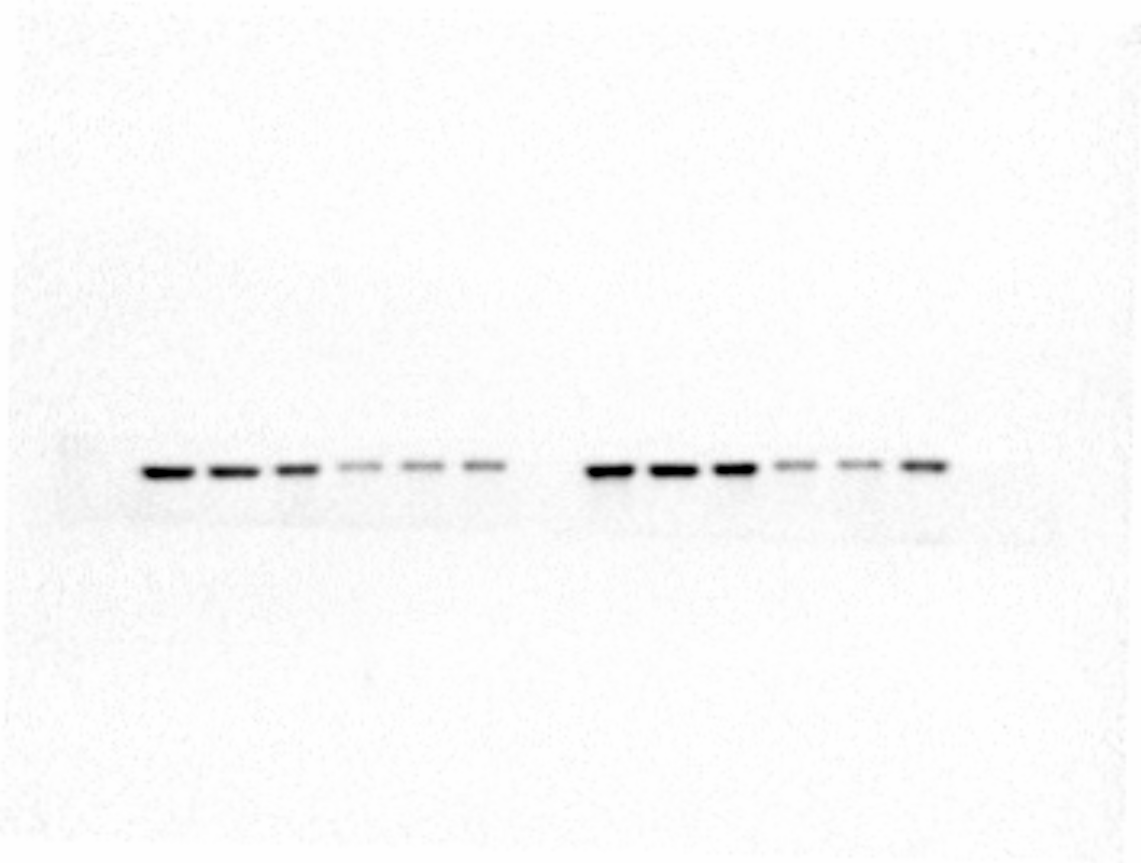

Supplement: Supplementary file 5 — Source data Fig. 1 [file 44318_2026_818_MOESM5_ESM.zip › Figure 1/Figure 1J/Figure 1J Replicate 1/GAPDH (lane 1-3).tif]

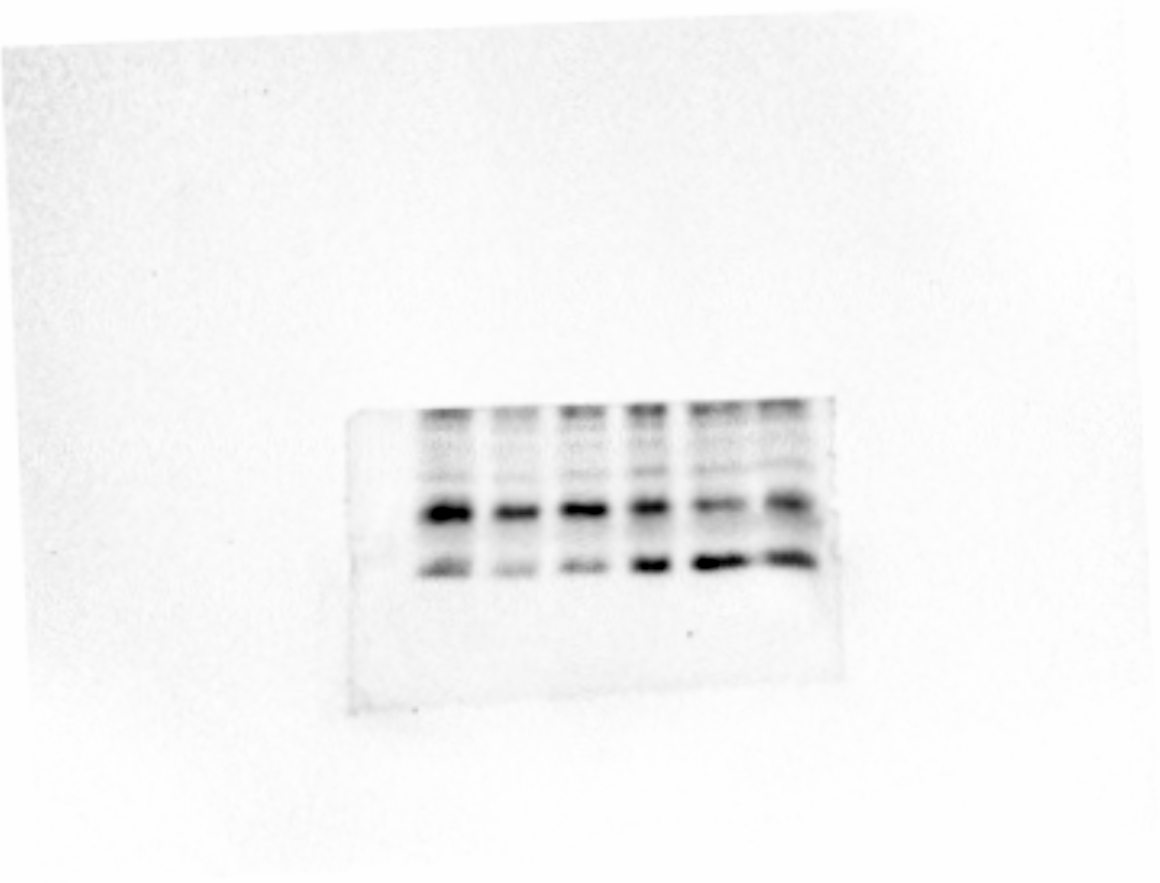

Supplement: Supplementary file 5 — Source data Fig. 1 [file 44318_2026_818_MOESM5_ESM.zip › Figure 1/Figure 1J/Figure 1J Replicate 1/LC3B (lane 1-3).tif]

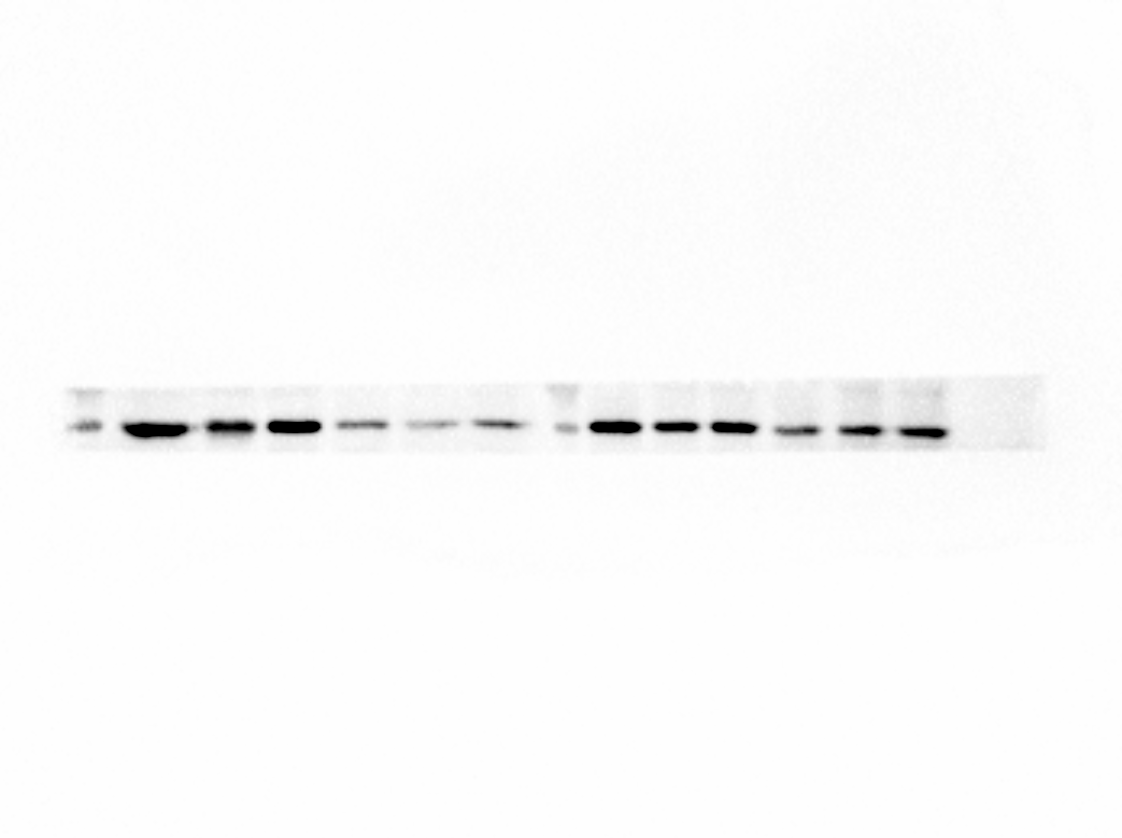

Supplement: Supplementary file 5 — Source data Fig. 1 [file 44318_2026_818_MOESM5_ESM.zip › Figure 1/Figure 1J/Figure 1J Replicate 1/p62 (lane 1-3).tif]

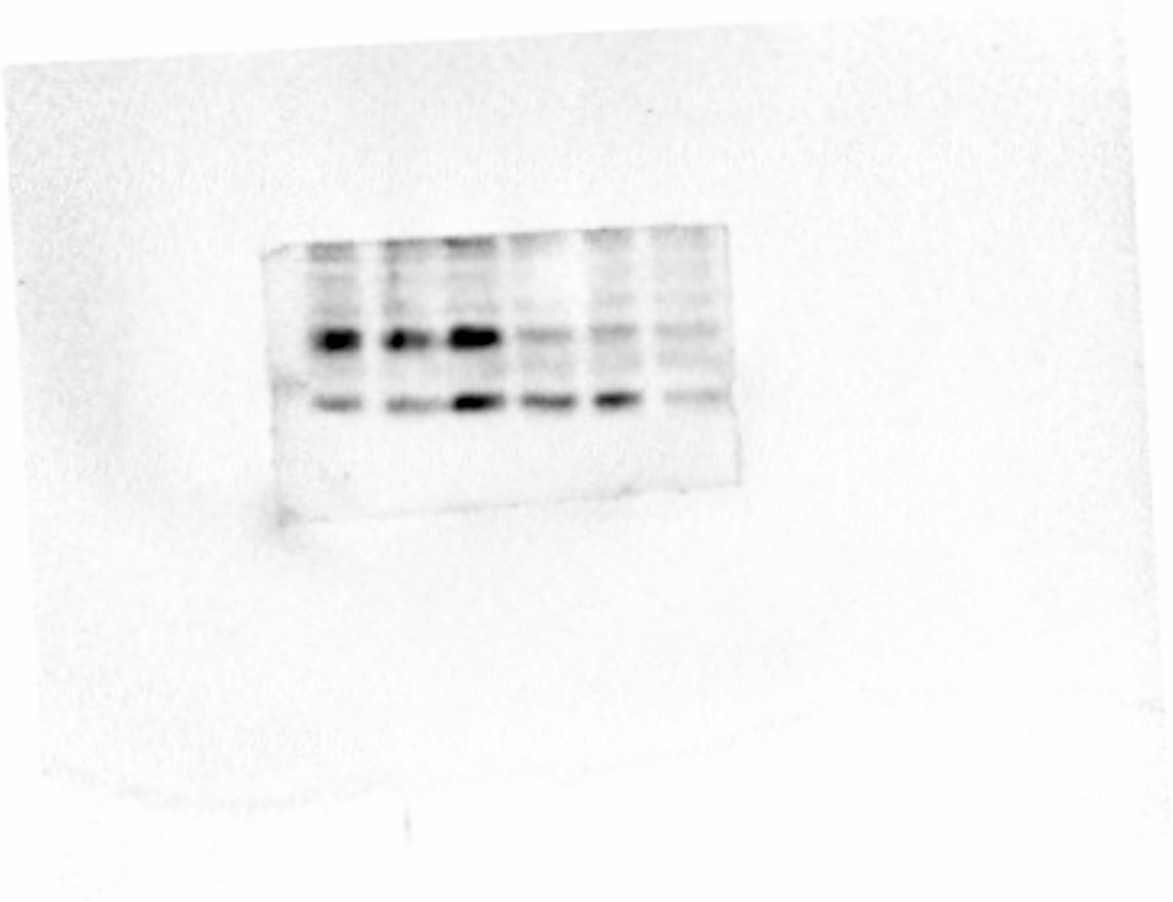

Supplement: Supplementary file 5 — Source data Fig. 1 [file 44318_2026_818_MOESM5_ESM.zip › Figure 1/Figure 1J/Figure 1J Replicate 2/LC3B (lane 1-3).tif]

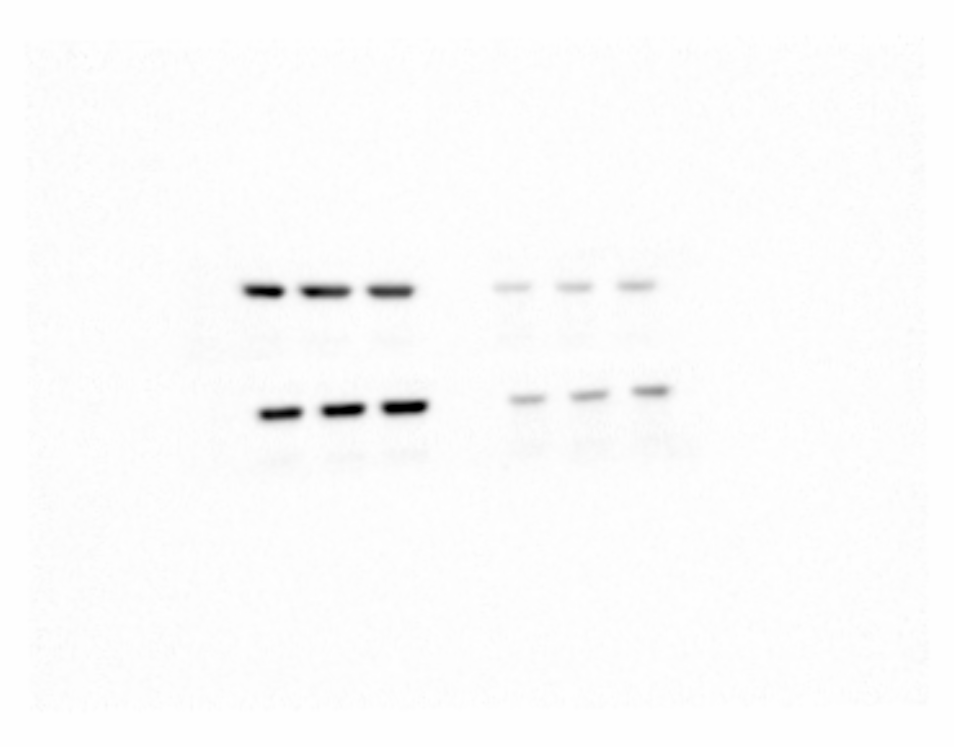

Supplement: Supplementary file 5 — Source data Fig. 1 [file 44318_2026_818_MOESM5_ESM.zip › Figure 1/Figure 1J/GAPDH (up lane 1-3).tif]

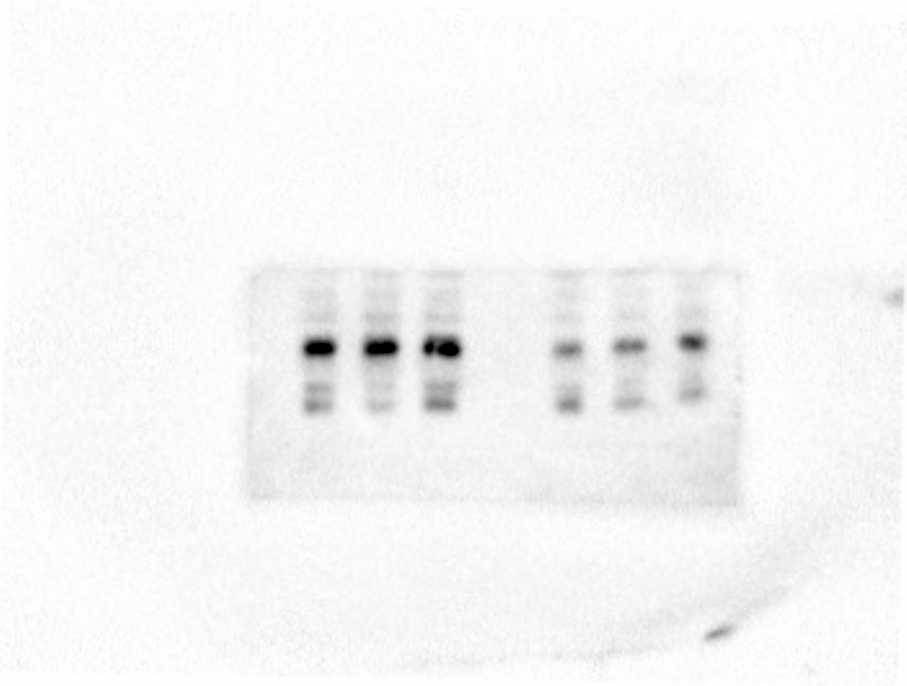

Supplement: Supplementary file 5 — Source data Fig. 1 [file 44318_2026_818_MOESM5_ESM.zip › Figure 1/Figure 1J/LC3B (lane 1-3).tif]

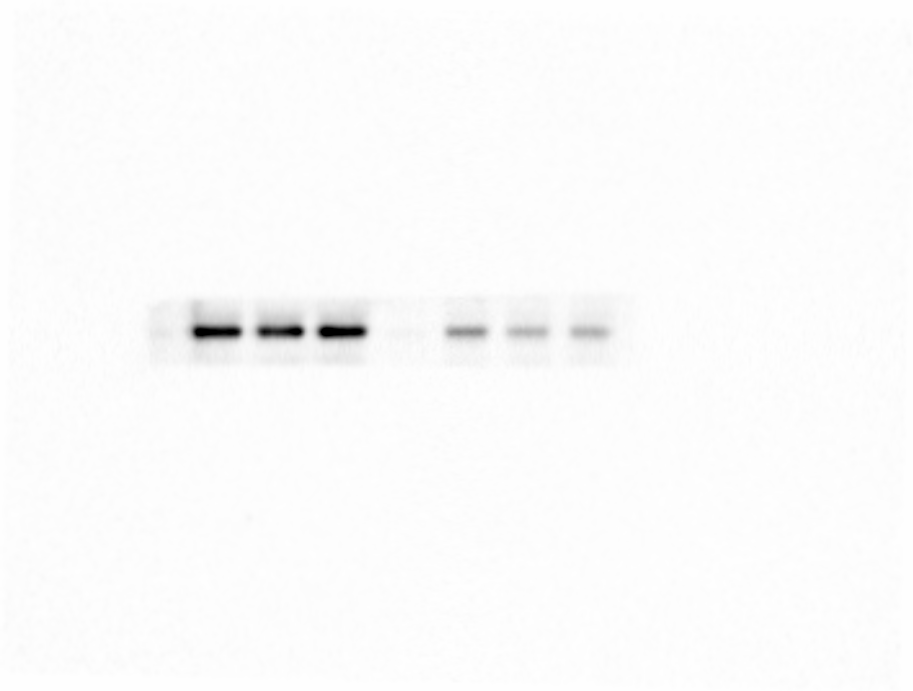

Supplement: Supplementary file 5 — Source data Fig. 1 [file 44318_2026_818_MOESM5_ESM.zip › Figure 1/Figure 1J/p62 (lane 1-3).tif]

Figure 1J

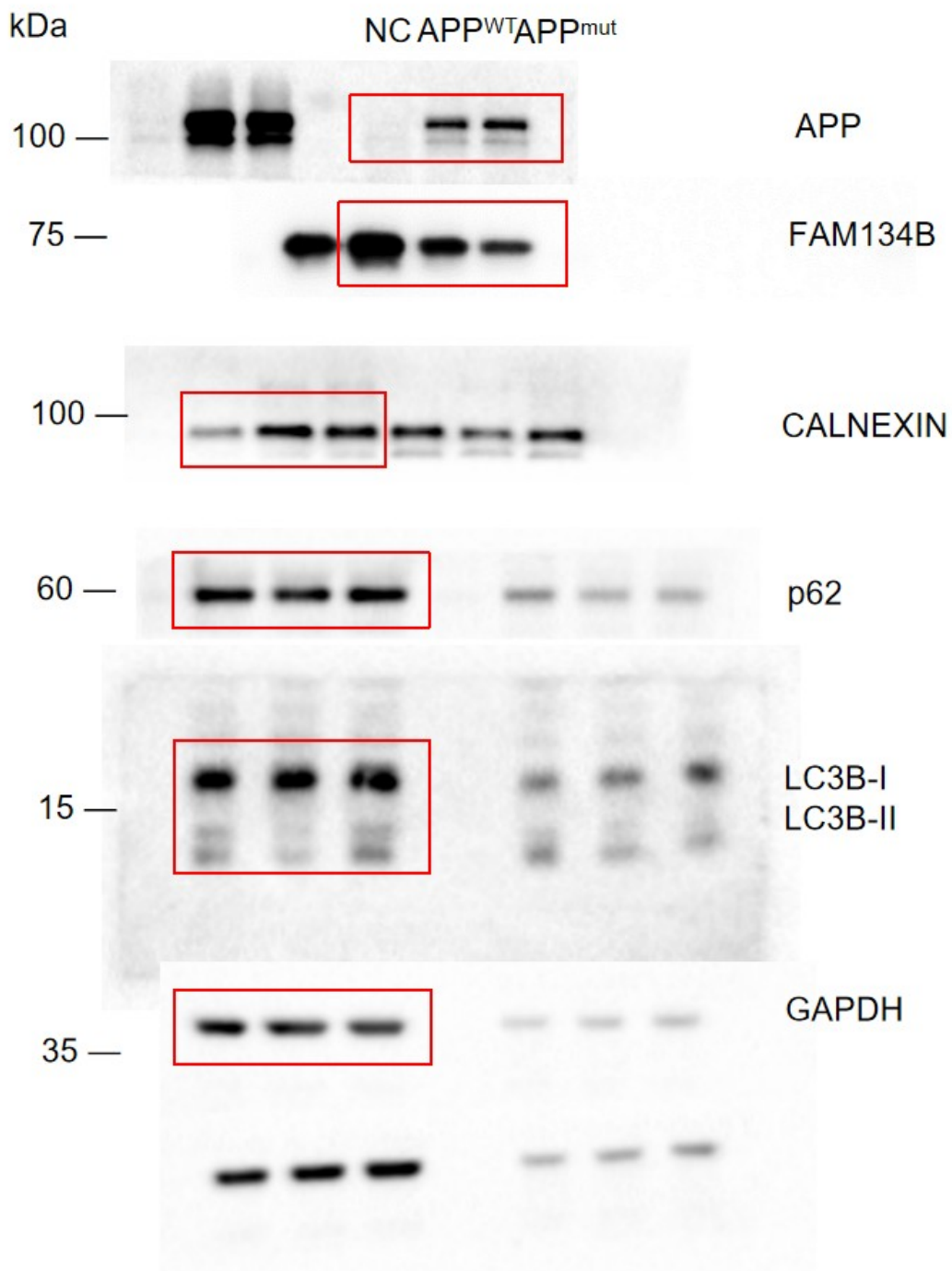

## Replicate 1

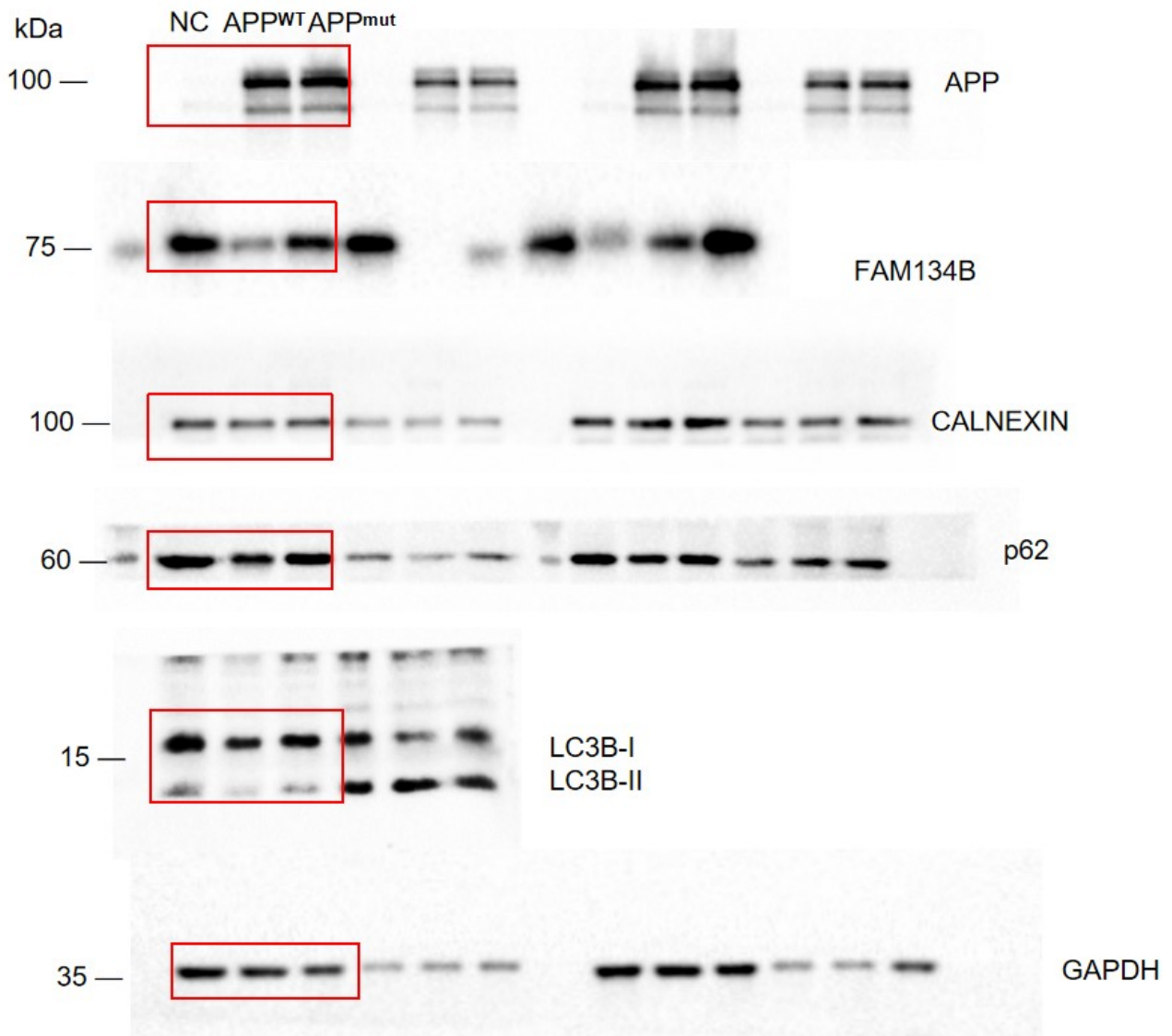

## Replicate 2

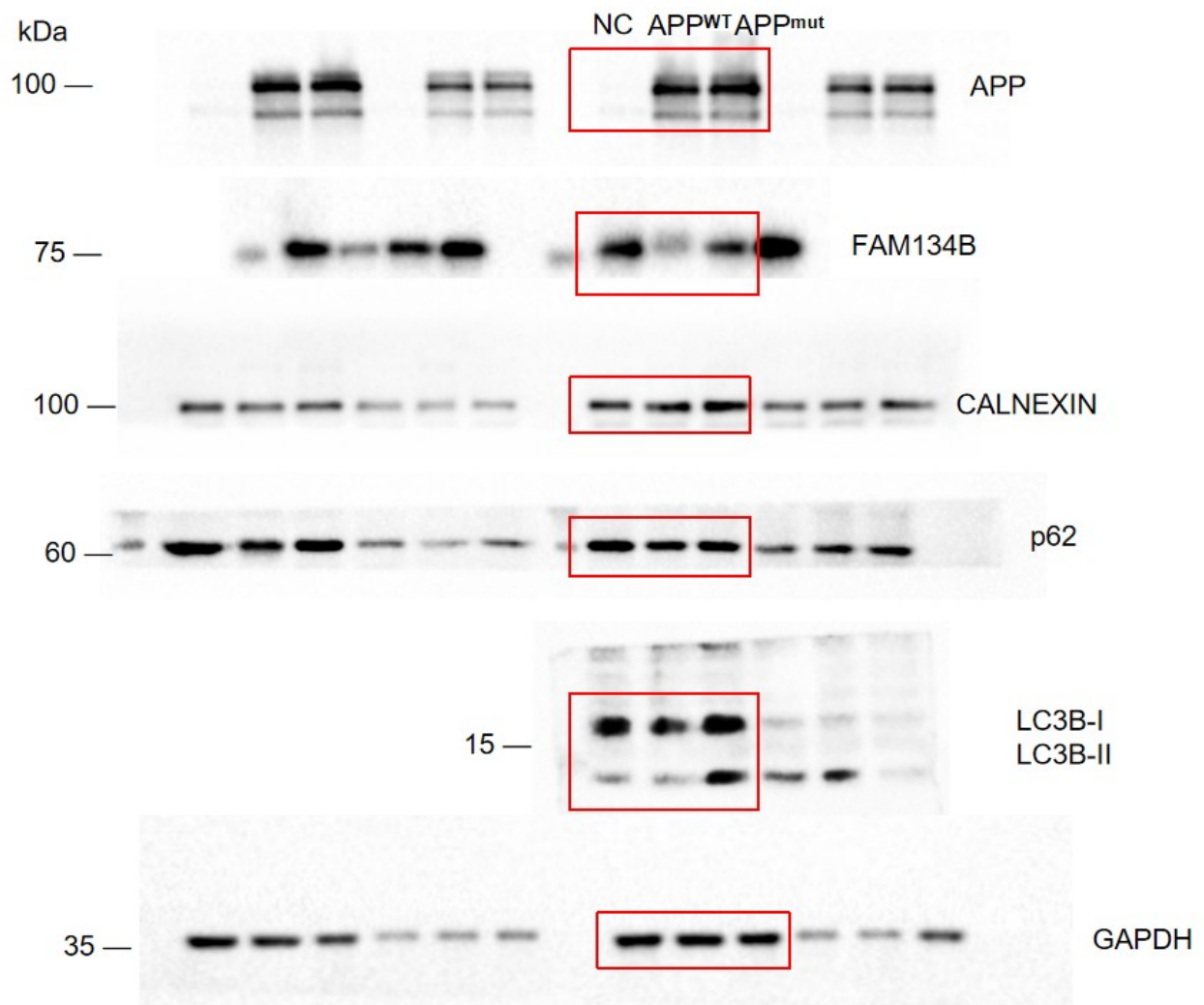

Supplement: Supplementary file 5 — Source data Fig. 1 [file 44318_2026_818_MOESM5_ESM.zip › Figure 1/Figure 1J/WB for Figure 1J.pdf]

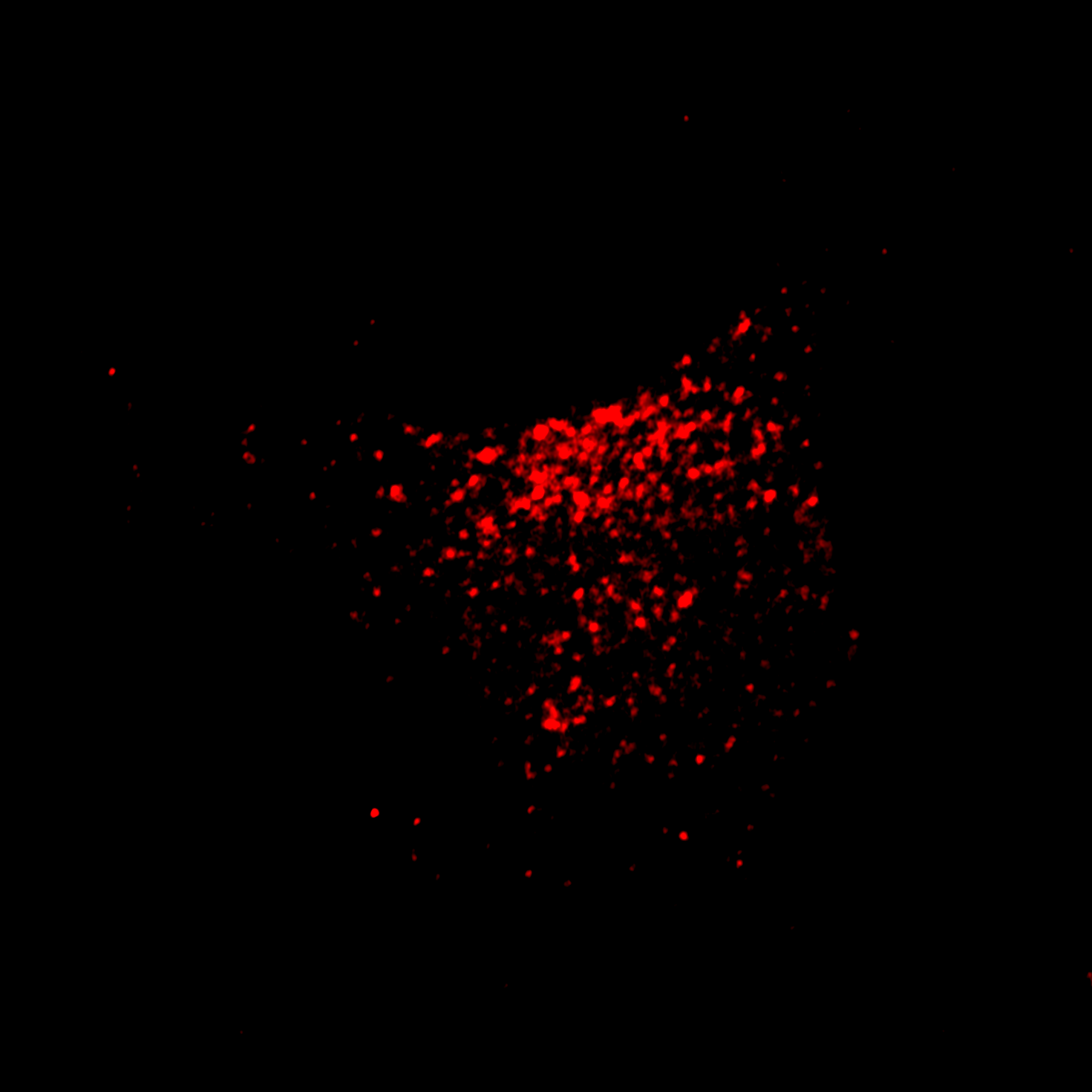

Supplement: Supplementary file 6 — Source data Fig. 2 [file 44318_2026_818_MOESM6_ESM.zip › Figure 2/Figure 2A/BafA1/APP.tif]

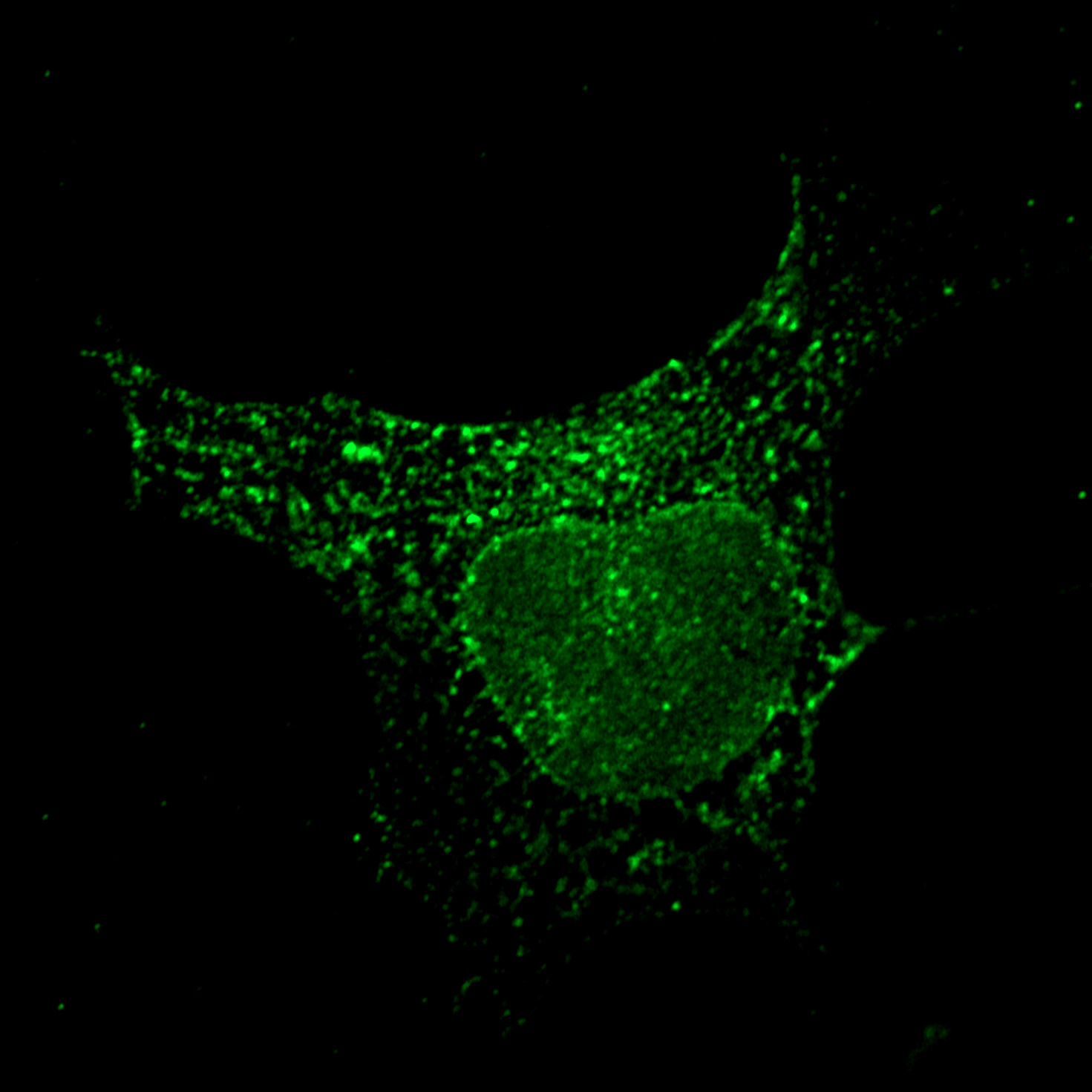

Supplement: Supplementary file 6 — Source data Fig. 2 [file 44318_2026_818_MOESM6_ESM.zip › Figure 2/Figure 2A/BafA1/CALNEXIN.tif]

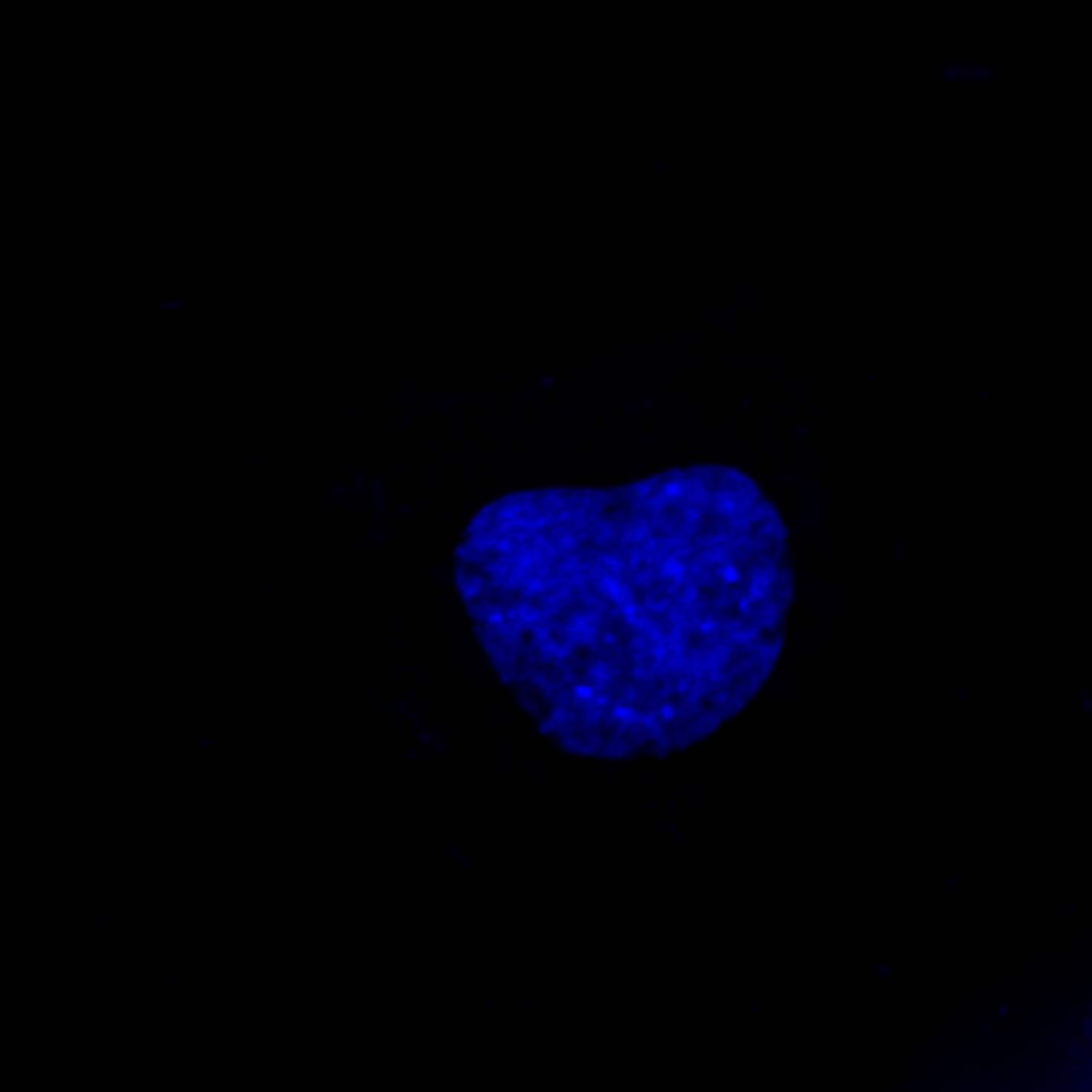

Supplement: Supplementary file 6 — Source data Fig. 2 [file 44318_2026_818_MOESM6_ESM.zip › Figure 2/Figure 2A/BafA1/DAPI.tif]

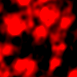

Supplement: Supplementary file 6 — Source data Fig. 2 [file 44318_2026_818_MOESM6_ESM.zip › Figure 2/Figure 2A/BafA1/Inset-APP.tif]

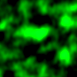

Supplement: Supplementary file 6 — Source data Fig. 2 [file 44318_2026_818_MOESM6_ESM.zip › Figure 2/Figure 2A/BafA1/Inset-CALNEXIN.tif]

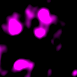

Supplement: Supplementary file 6 — Source data Fig. 2 [file 44318_2026_818_MOESM6_ESM.zip › Figure 2/Figure 2A/BafA1/Inset-LAMP1.tif]

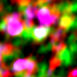

Supplement: Supplementary file 6 — Source data Fig. 2 [file 44318_2026_818_MOESM6_ESM.zip › Figure 2/Figure 2A/BafA1/Inset-Merge.tif]

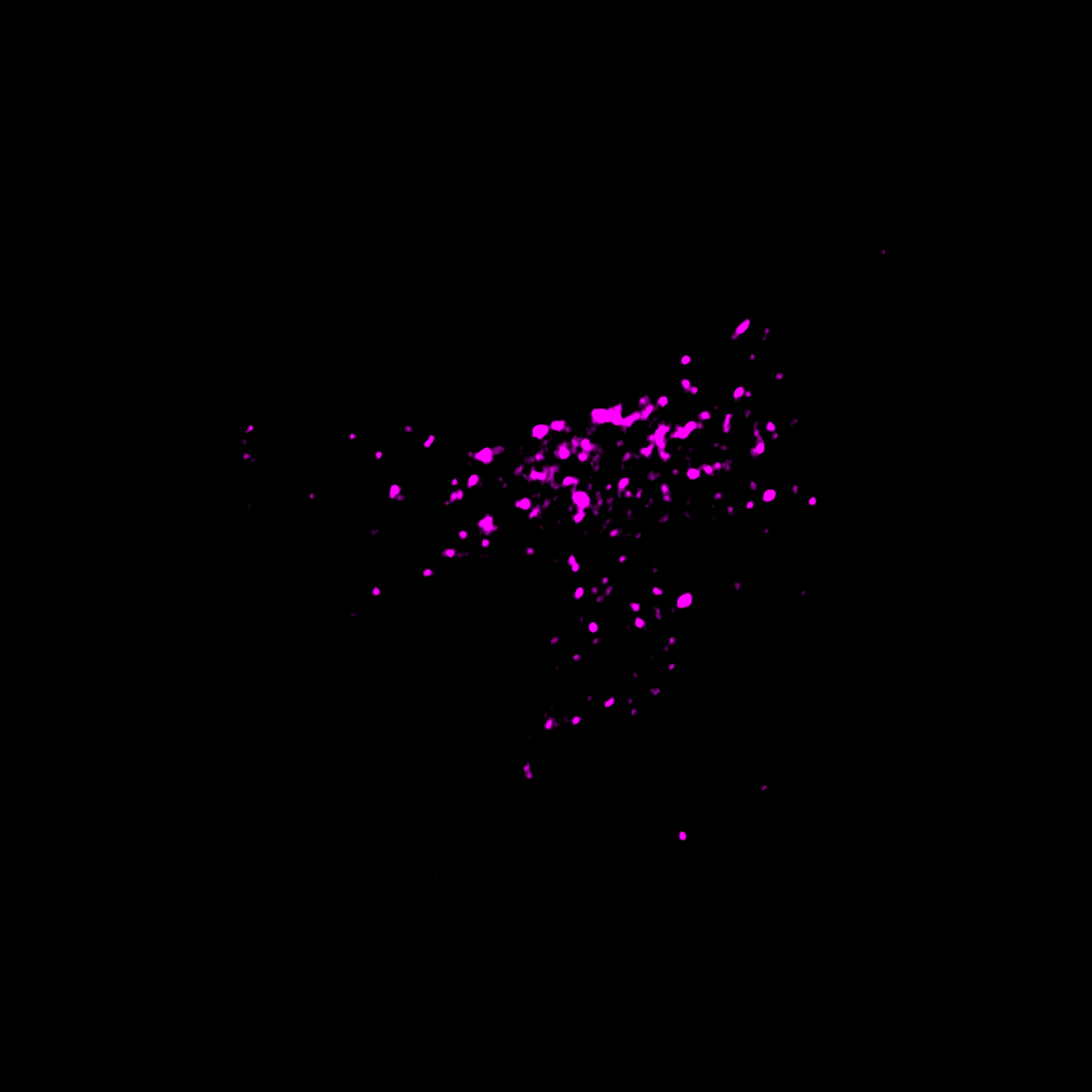

Supplement: Supplementary file 6 — Source data Fig. 2 [file 44318_2026_818_MOESM6_ESM.zip › Figure 2/Figure 2A/BafA1/LAMP1.tif]

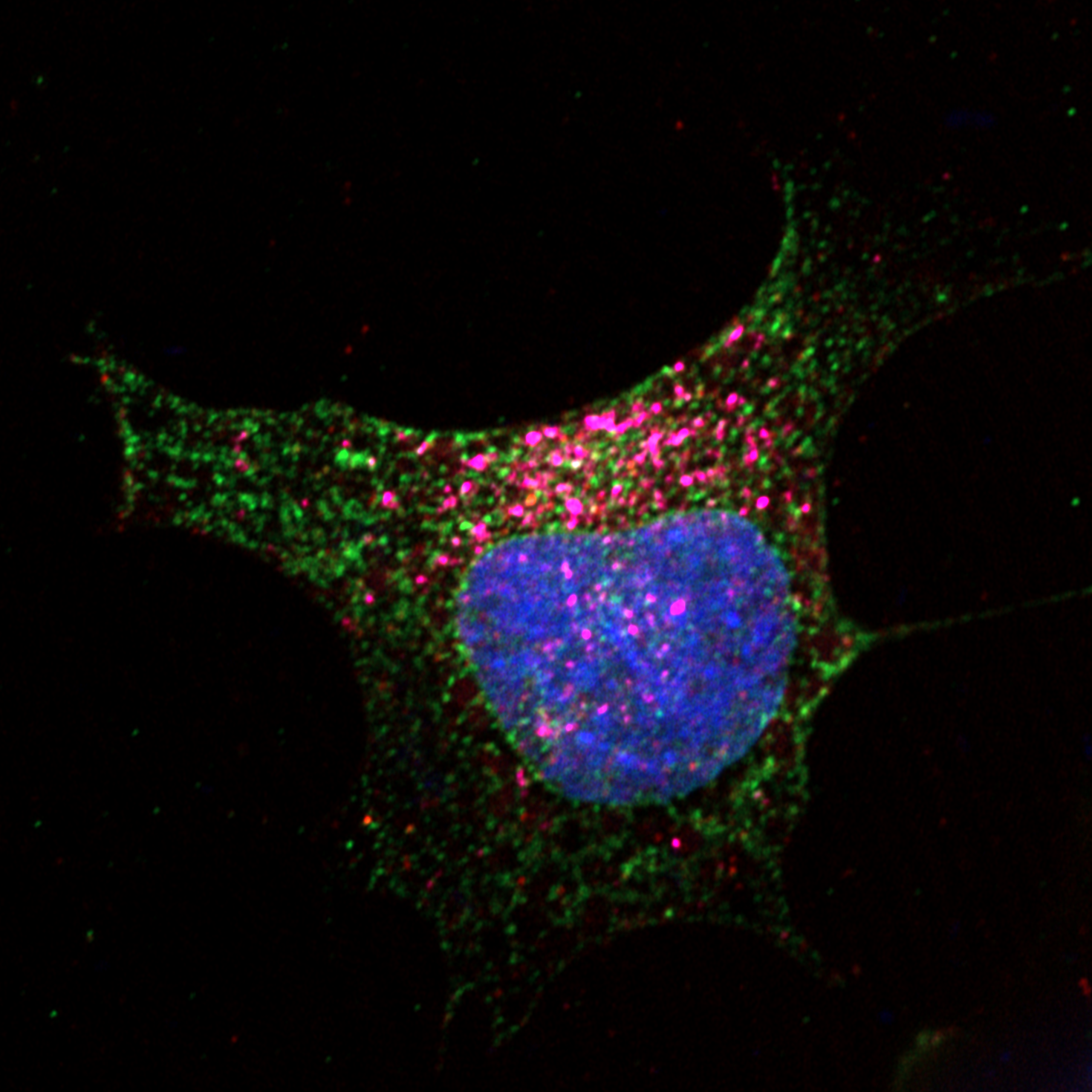

Supplement: Supplementary file 6 — Source data Fig. 2 [file 44318_2026_818_MOESM6_ESM.zip › Figure 2/Figure 2A/BafA1/Merge.tif]

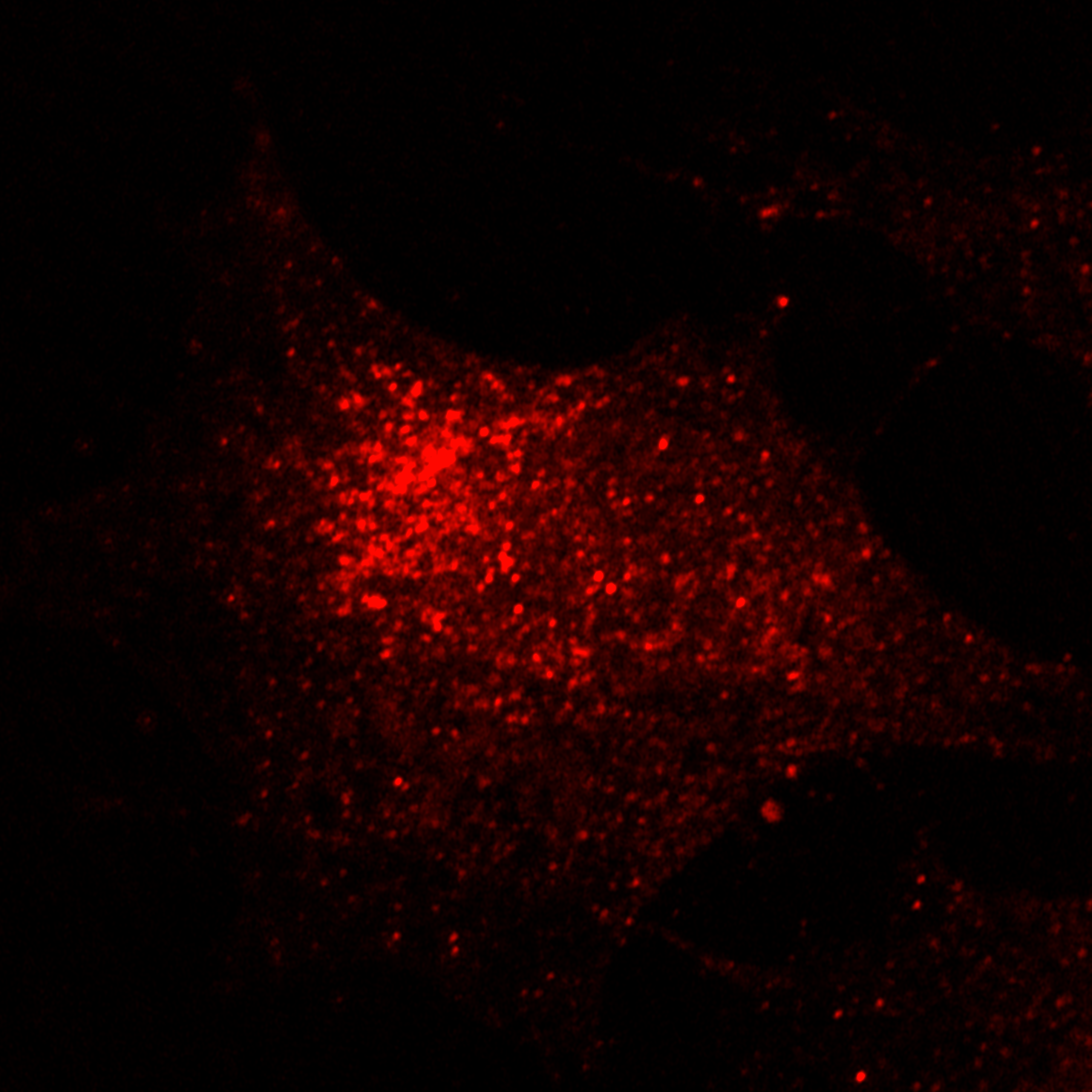

Supplement: Supplementary file 6 — Source data Fig. 2 [file 44318_2026_818_MOESM6_ESM.zip › Figure 2/Figure 2A/EBSS+BafA1/APP.tif]

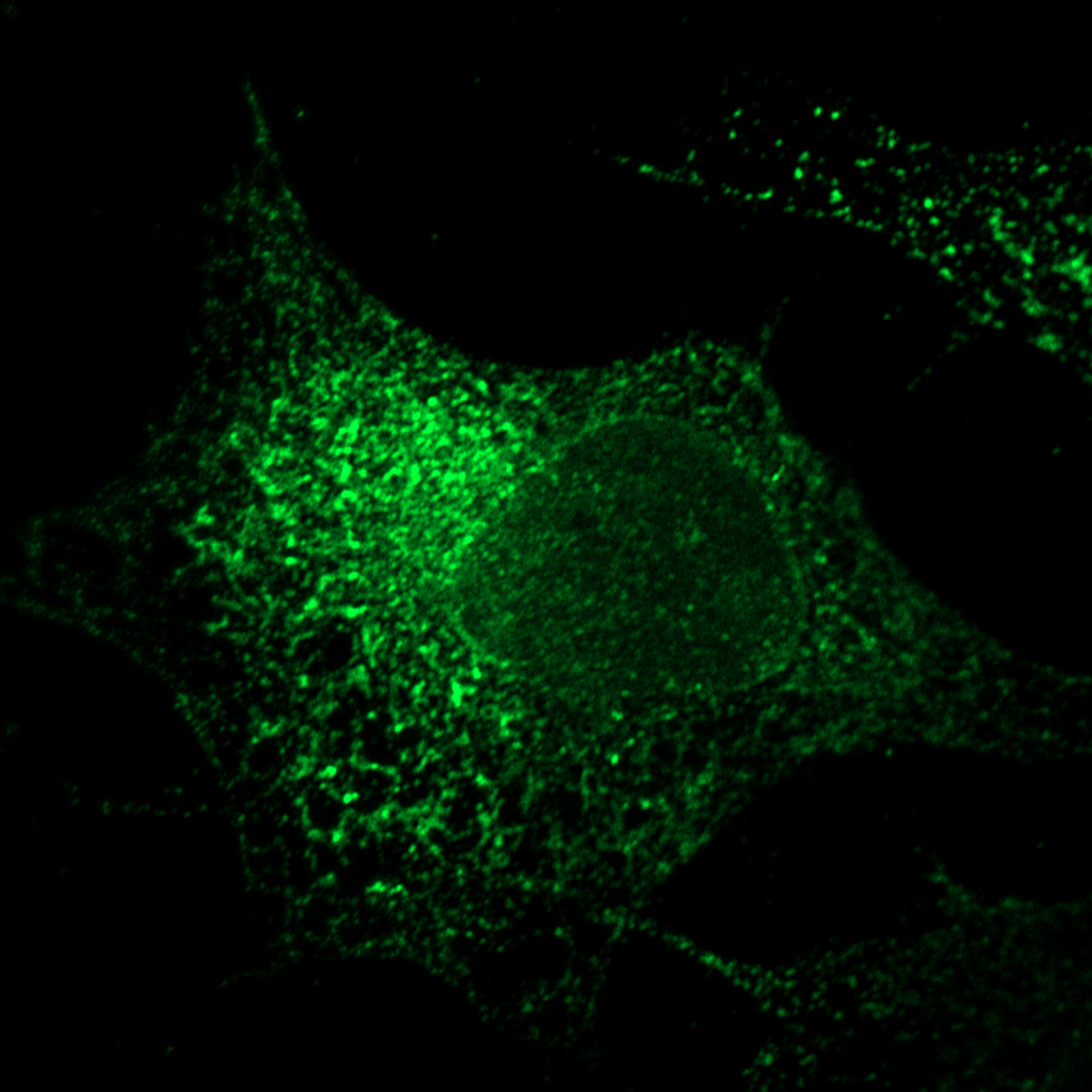

Supplement: Supplementary file 6 — Source data Fig. 2 [file 44318_2026_818_MOESM6_ESM.zip › Figure 2/Figure 2A/EBSS+BafA1/CALNEXIN.tif]

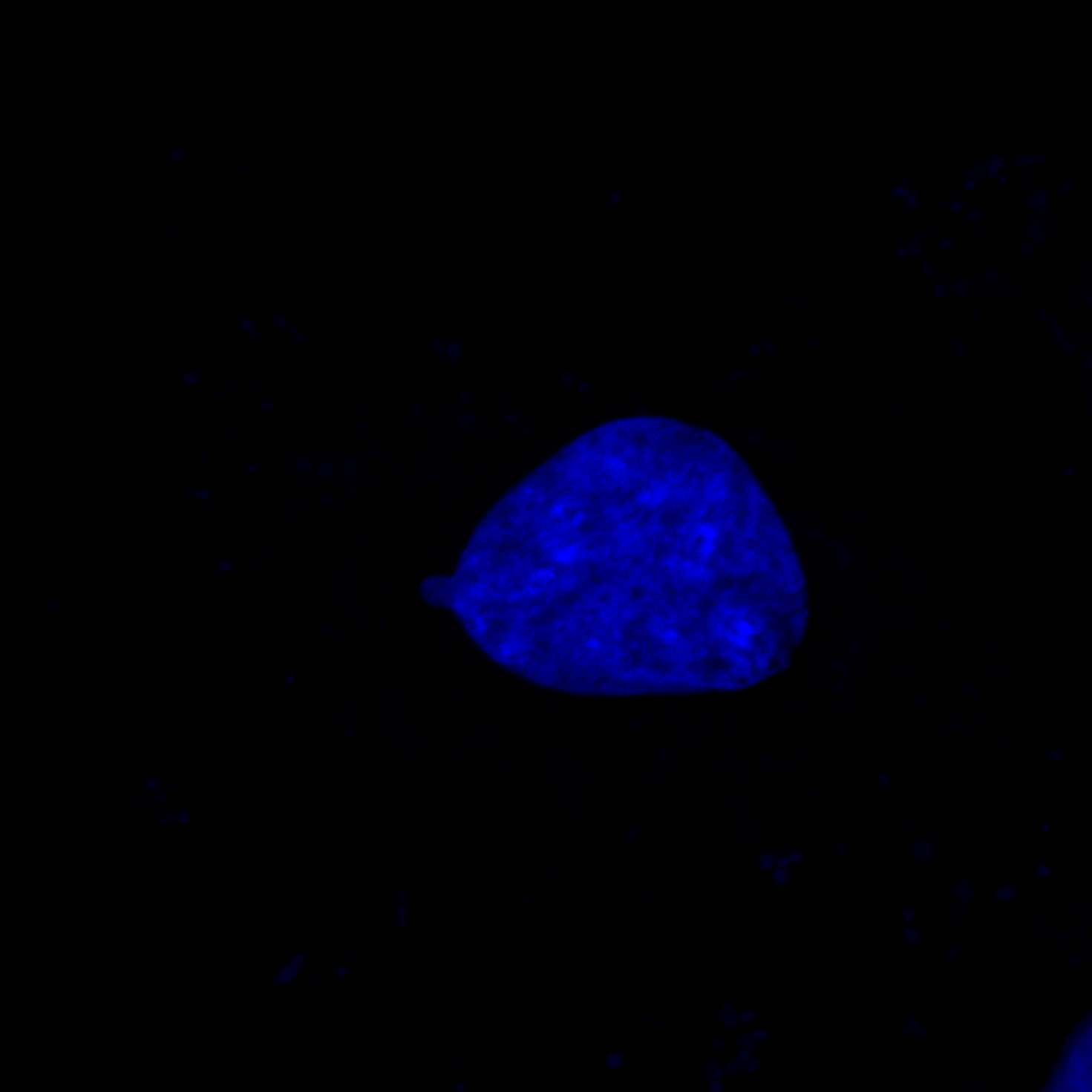

Supplement: Supplementary file 6 — Source data Fig. 2 [file 44318_2026_818_MOESM6_ESM.zip › Figure 2/Figure 2A/EBSS+BafA1/DAPI.tif]

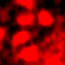

Supplement: Supplementary file 6 — Source data Fig. 2 [file 44318_2026_818_MOESM6_ESM.zip › Figure 2/Figure 2A/EBSS+BafA1/Inset-APP.tif]

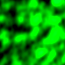

Supplement: Supplementary file 6 — Source data Fig. 2 [file 44318_2026_818_MOESM6_ESM.zip › Figure 2/Figure 2A/EBSS+BafA1/Inset-CALNEXIN.tif]

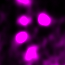

Supplement: Supplementary file 6 — Source data Fig. 2 [file 44318_2026_818_MOESM6_ESM.zip › Figure 2/Figure 2A/EBSS+BafA1/Inset-LAMP1.tif]

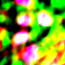

Supplement: Supplementary file 6 — Source data Fig. 2 [file 44318_2026_818_MOESM6_ESM.zip › Figure 2/Figure 2A/EBSS+BafA1/Inset-Merge.tif]

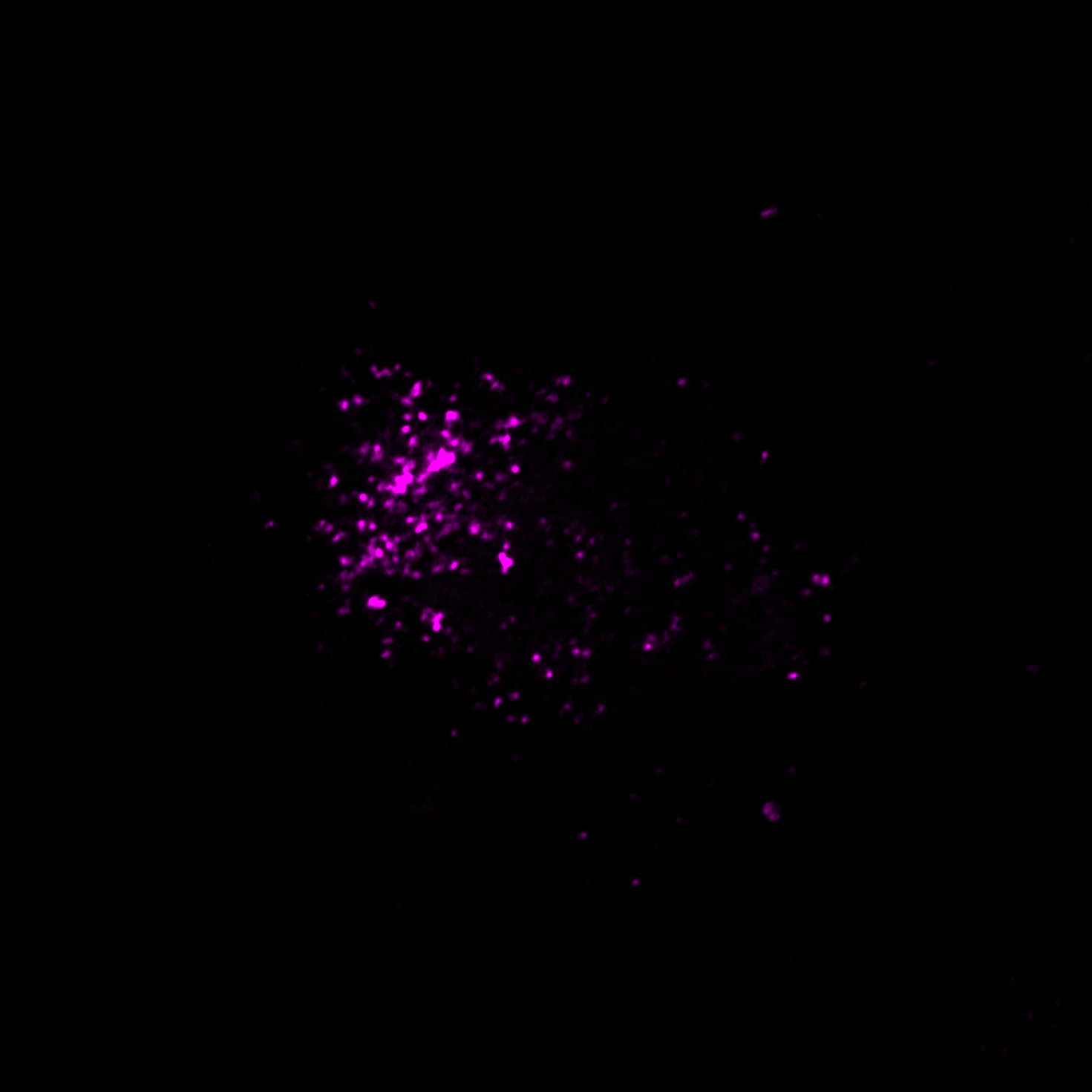

Supplement: Supplementary file 6 — Source data Fig. 2 [file 44318_2026_818_MOESM6_ESM.zip › Figure 2/Figure 2A/EBSS+BafA1/LAMP1.tif]

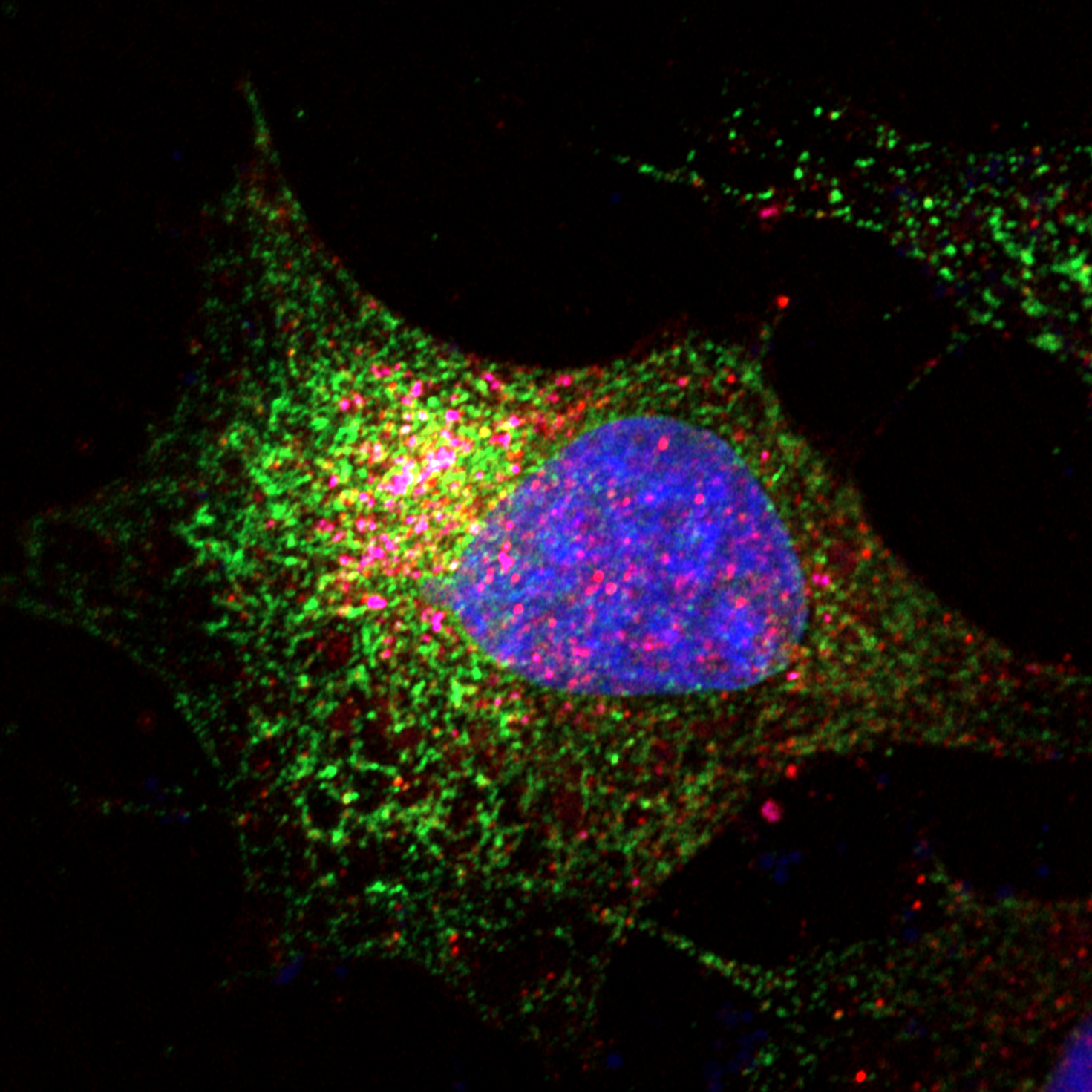

Supplement: Supplementary file 6 — Source data Fig. 2 [file 44318_2026_818_MOESM6_ESM.zip › Figure 2/Figure 2A/EBSS+BafA1/merge.tif]

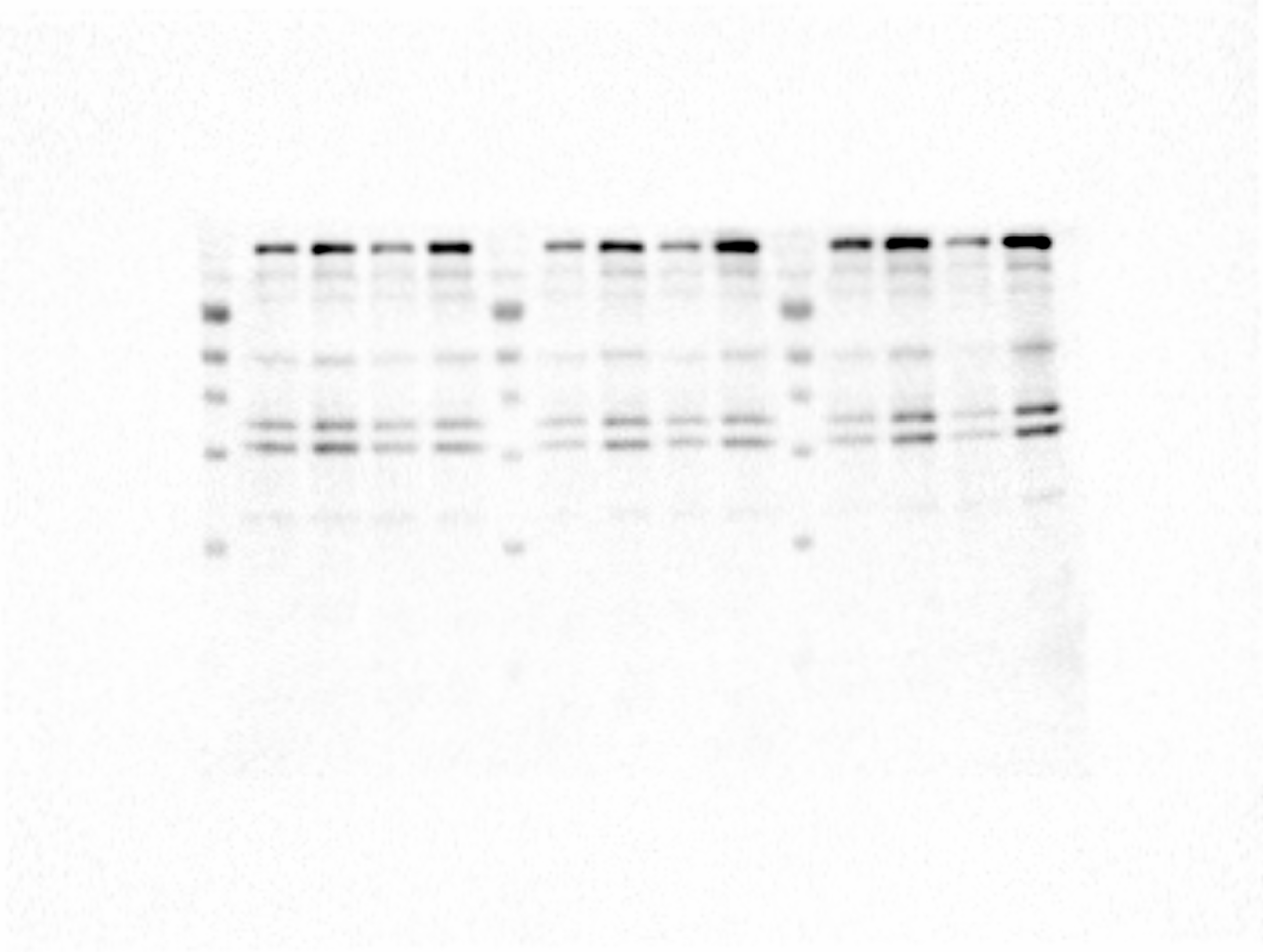

Supplement: Supplementary file 6 — Source data Fig. 2 [file 44318_2026_818_MOESM6_ESM.zip › Figure 2/Figure 2D/APP-mCherry+mCherry (lane 5-8).tif]

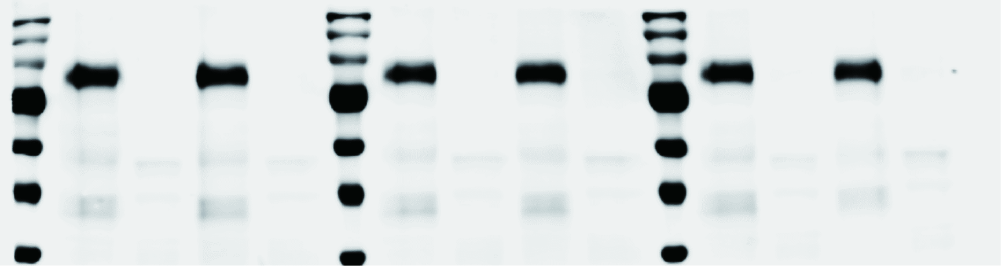

Supplement: Supplementary file 6 — Source data Fig. 2 [file 44318_2026_818_MOESM6_ESM.zip › Figure 2/Figure 2D/FAM134B (lane 5-8).tif]

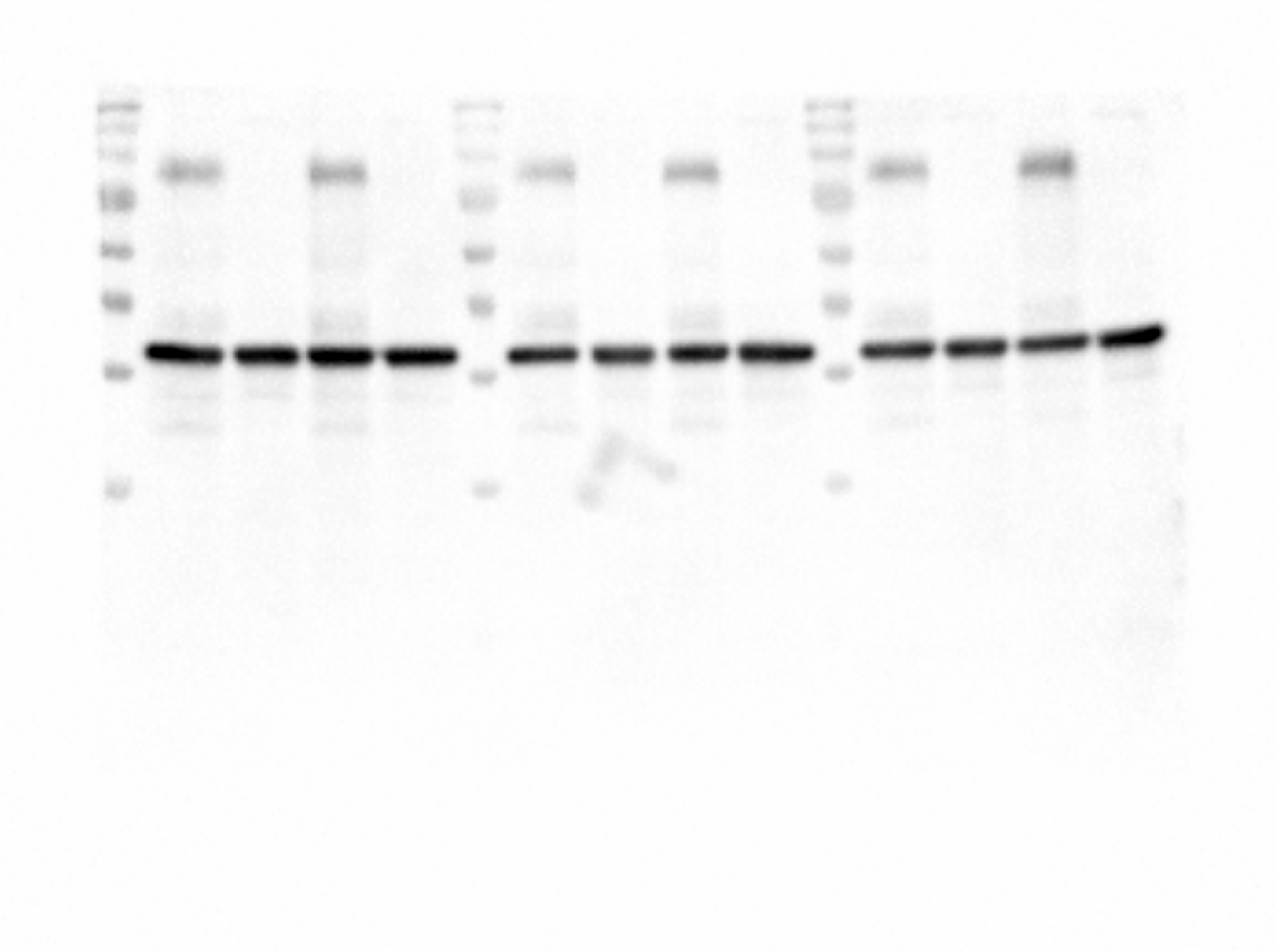

Supplement: Supplementary file 6 — Source data Fig. 2 [file 44318_2026_818_MOESM6_ESM.zip › Figure 2/Figure 2D/Figure 2D Replicate 1/GAPDH (lane 1-4).tif]

Figure 2D

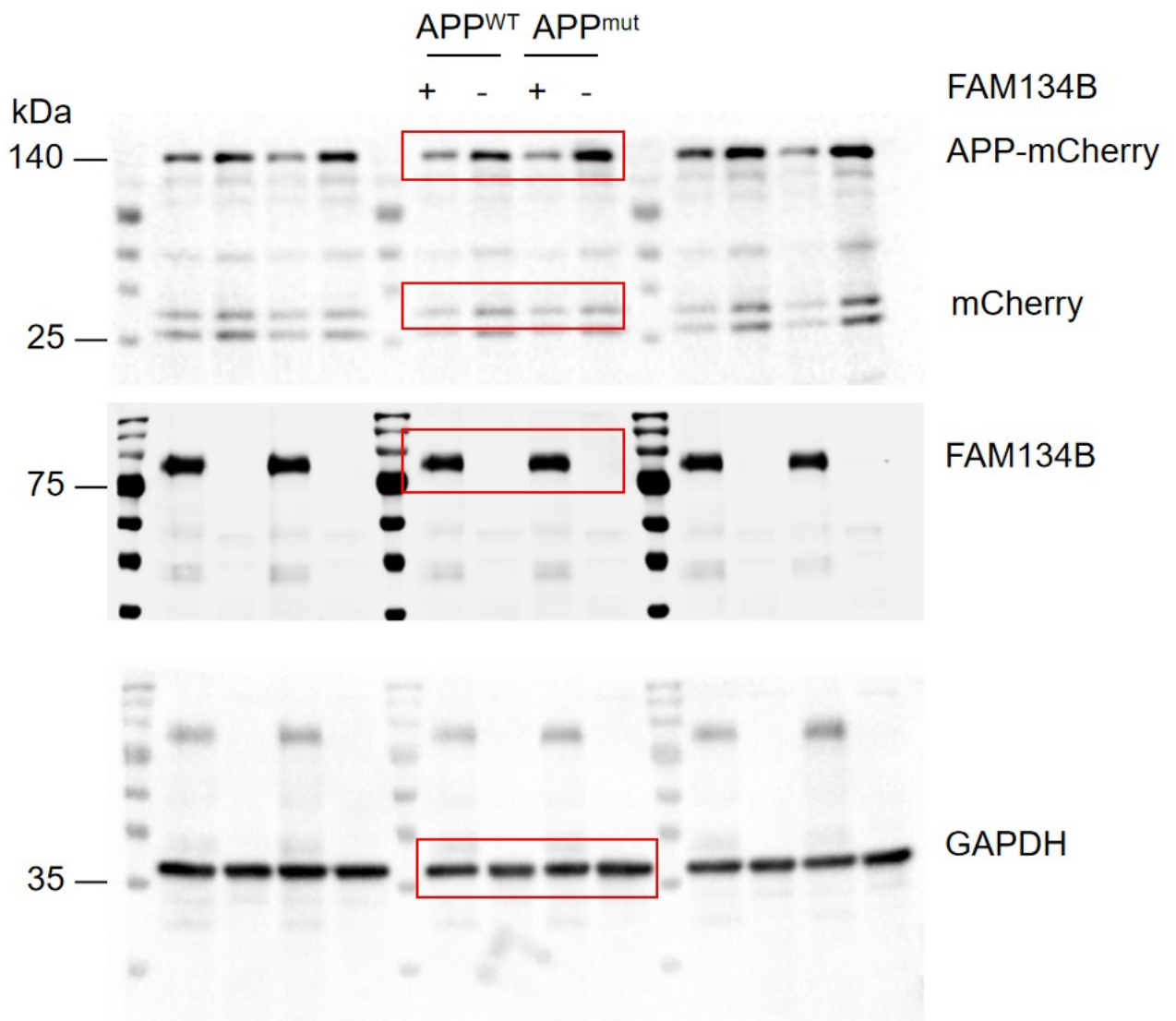

## Replicate 1

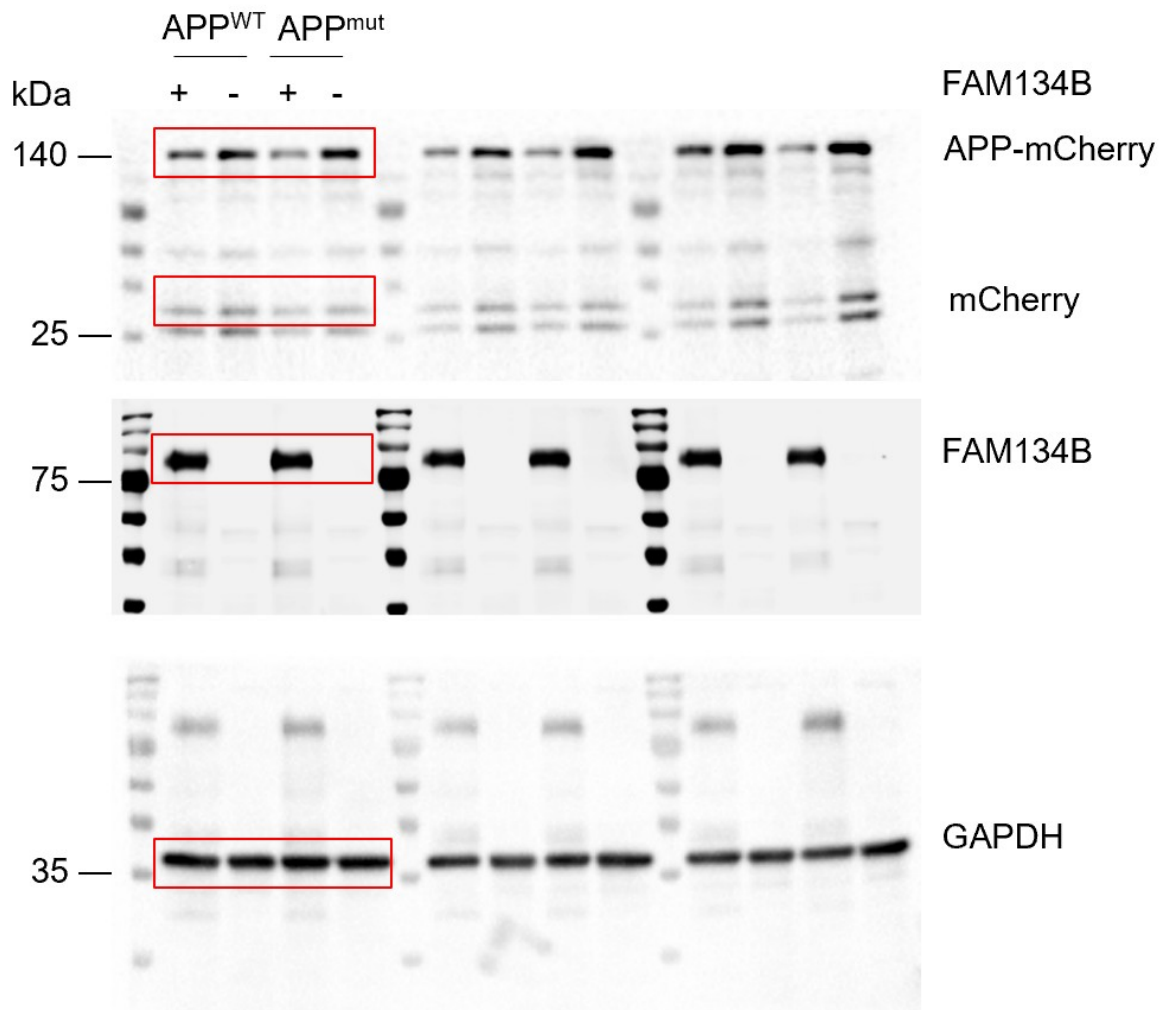

## Replicate 2

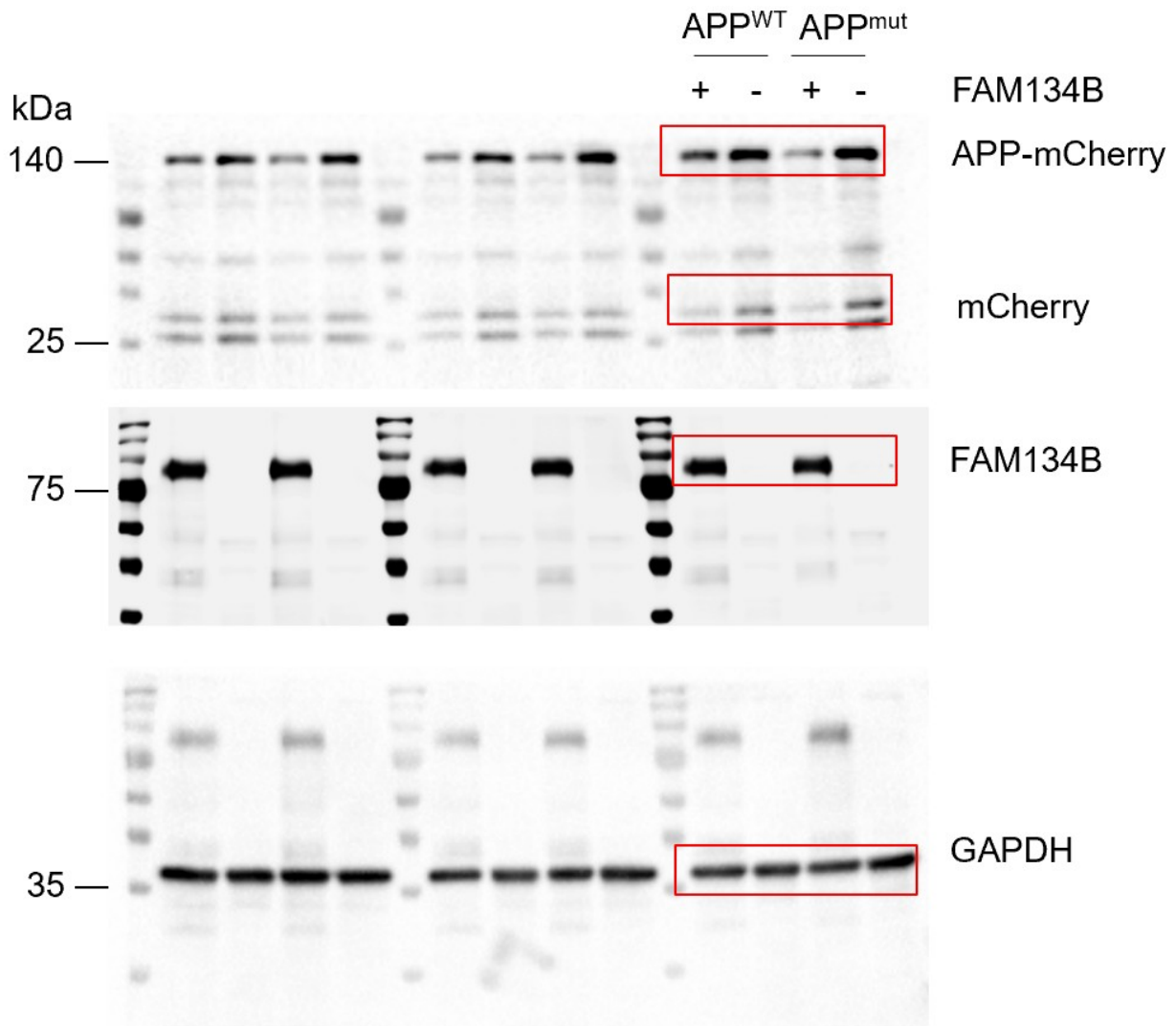

Supplement: Supplementary file 6 — Source data Fig. 2 [file 44318_2026_818_MOESM6_ESM.zip › Figure 2/Figure 2D/WB for Figure 2D.pdf]

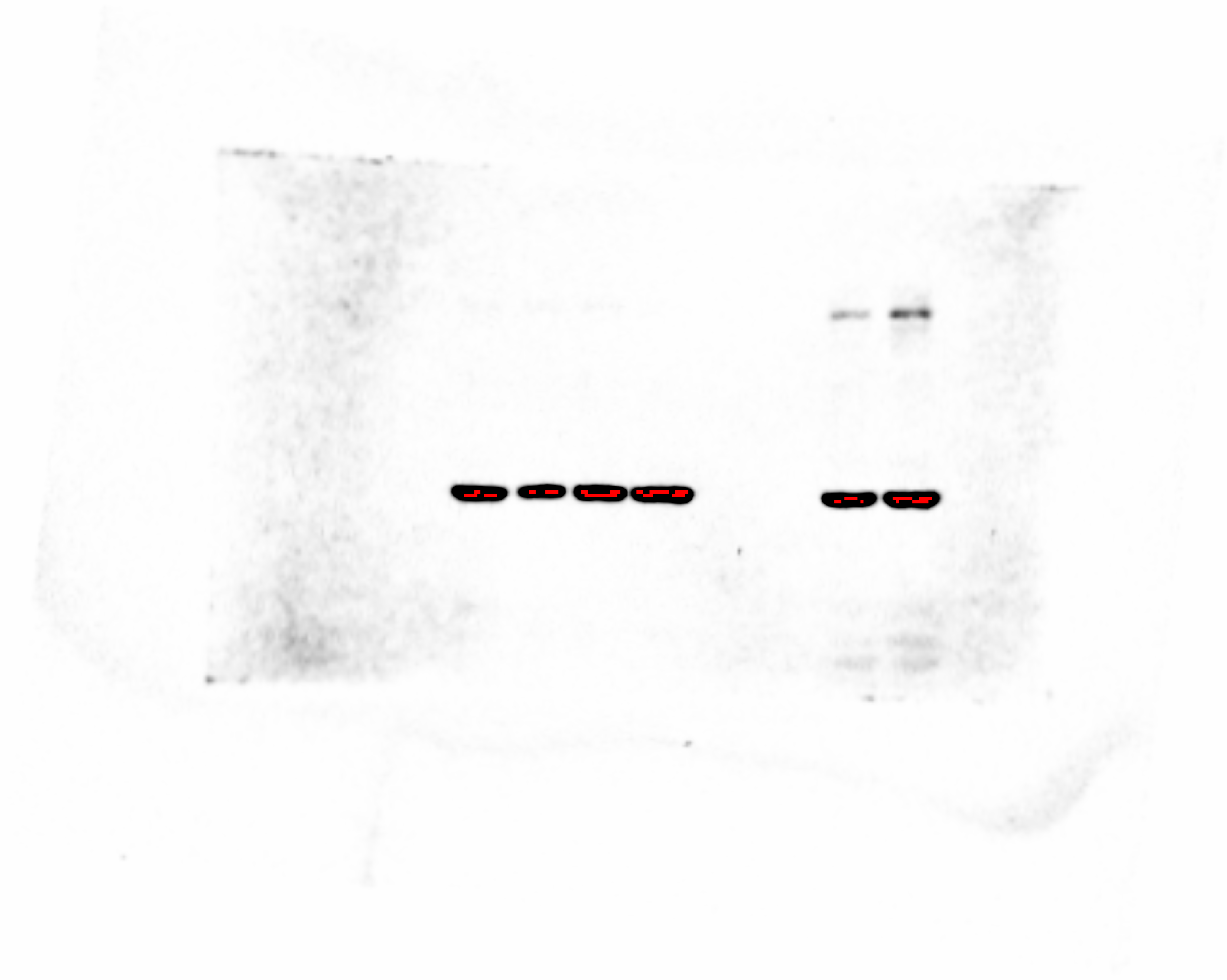

Supplement: Supplementary file 6 — Source data Fig. 2 [file 44318_2026_818_MOESM6_ESM.zip › Figure 2/Figure 2E/APP (up lane 5-6).tif]

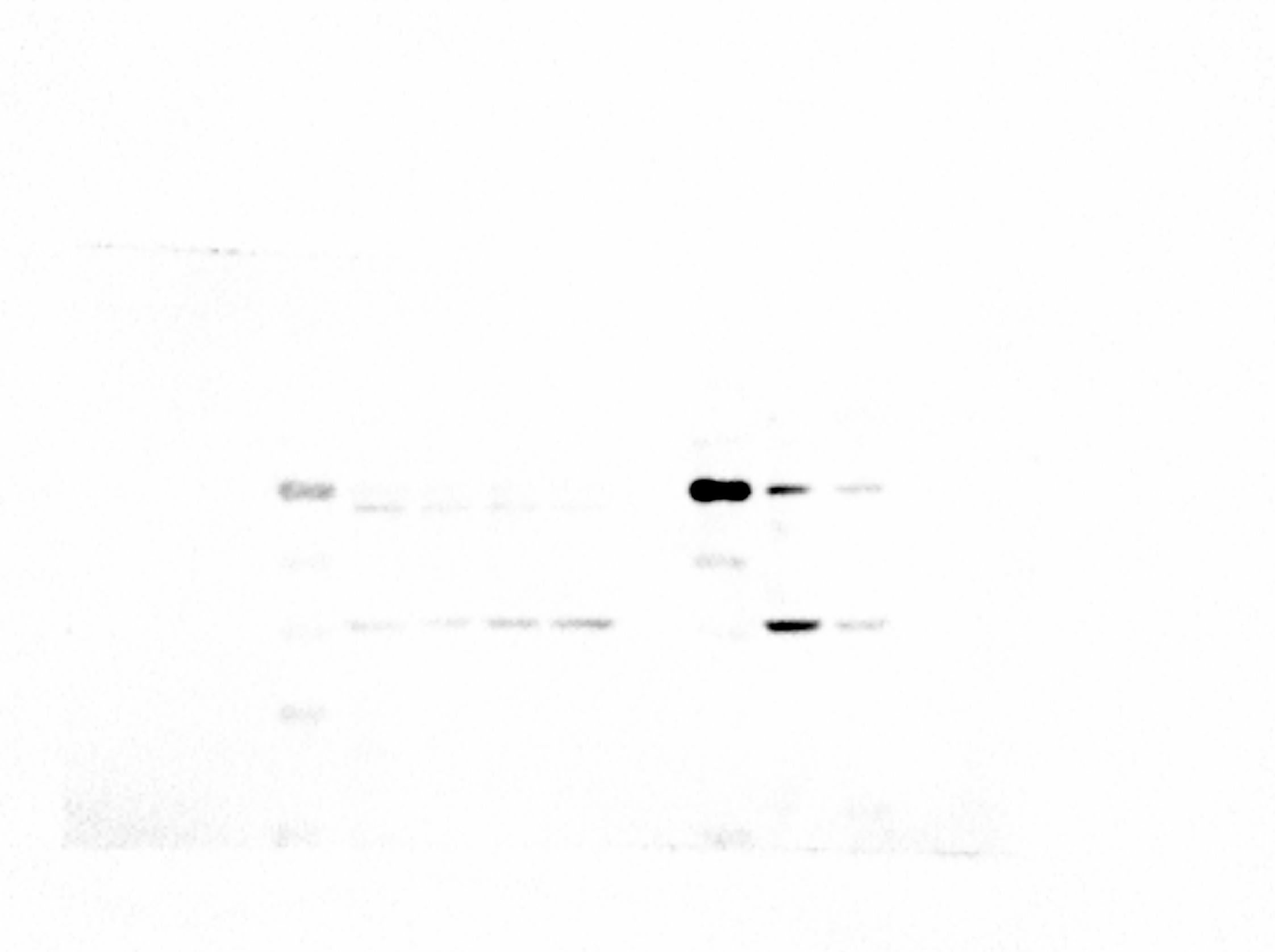

Supplement: Supplementary file 6 — Source data Fig. 2 [file 44318_2026_818_MOESM6_ESM.zip › Figure 2/Figure 2E/FAM134B (up lane 5-6).tif]

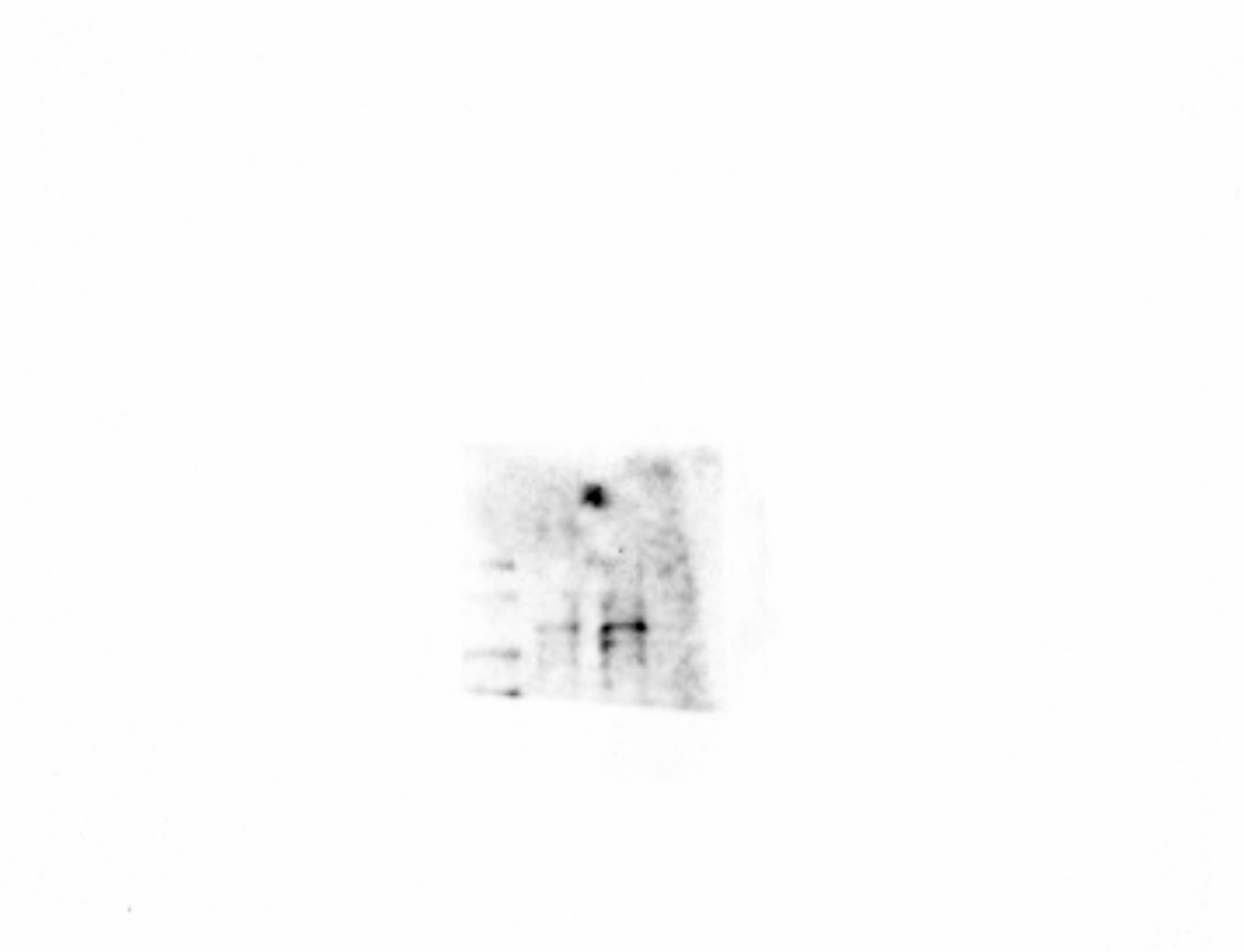

Supplement: Supplementary file 6 — Source data Fig. 2 [file 44318_2026_818_MOESM6_ESM.zip › Figure 2/Figure 2E/Figure 2E Replicate 1/APP.tif]

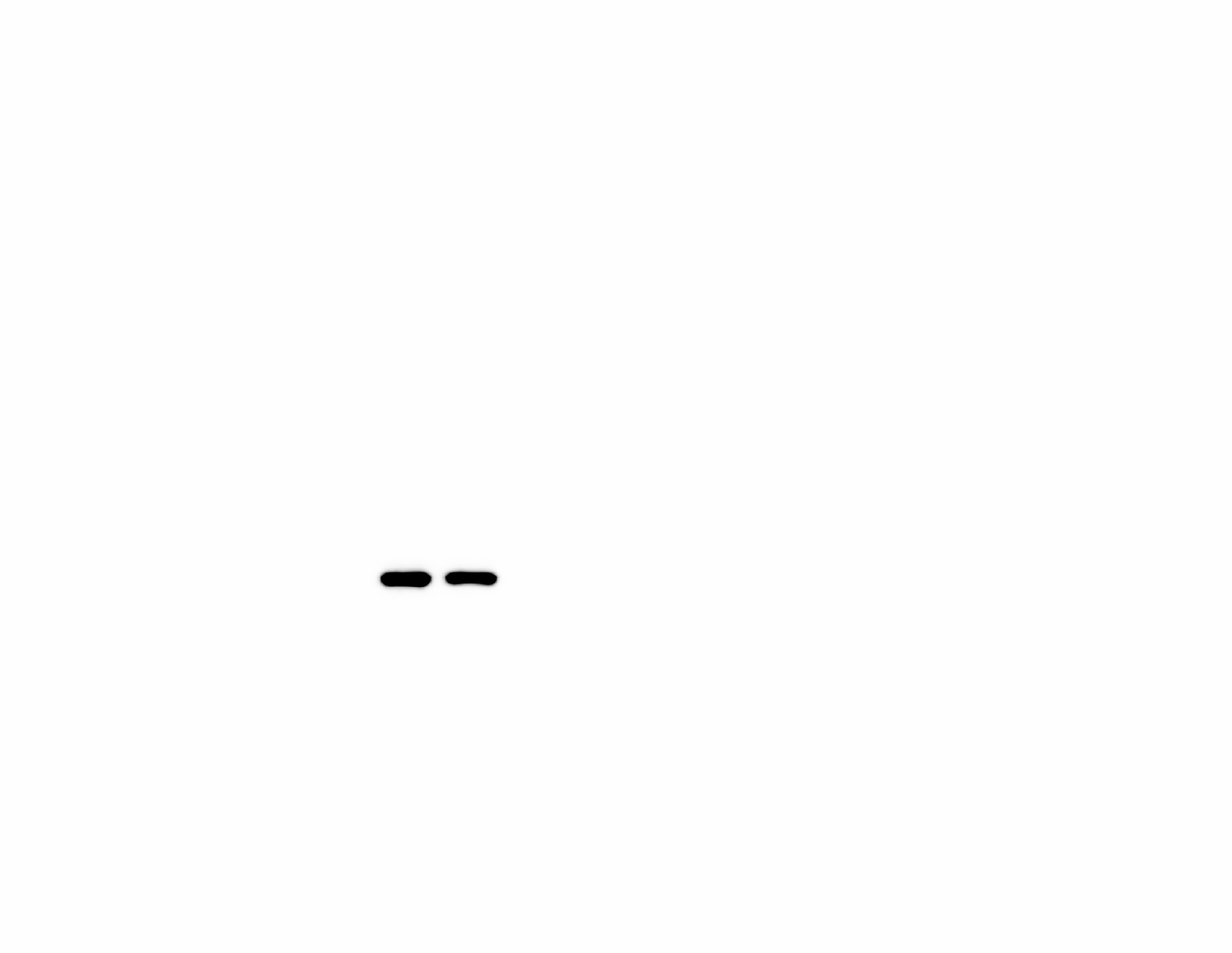

Supplement: Supplementary file 6 — Source data Fig. 2 [file 44318_2026_818_MOESM6_ESM.zip › Figure 2/Figure 2E/Figure 2E Replicate 1/GAPDH.tif]

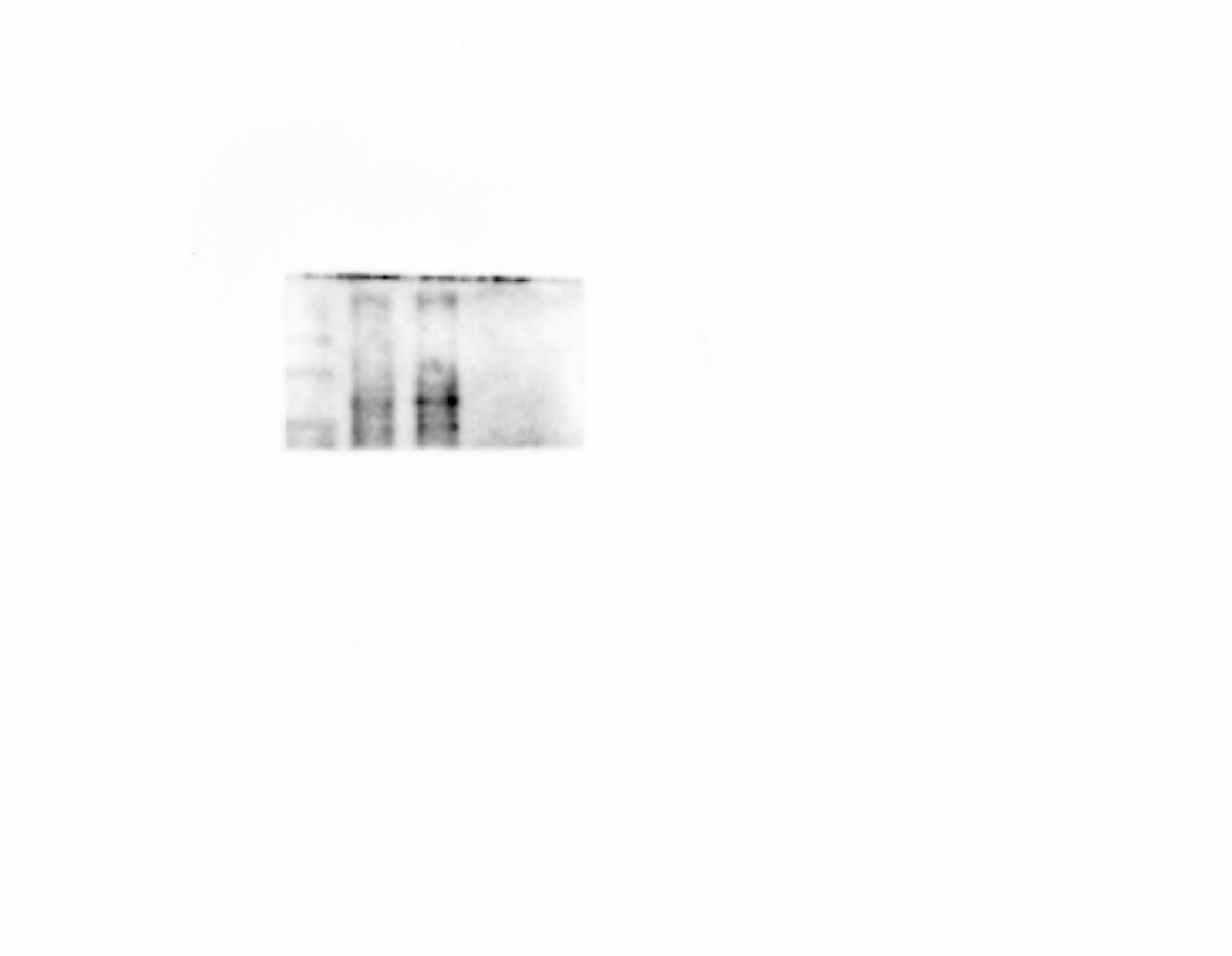

Supplement: Supplementary file 6 — Source data Fig. 2 [file 44318_2026_818_MOESM6_ESM.zip › Figure 2/Figure 2E/Figure 2E Replicate 2/APP.tif]

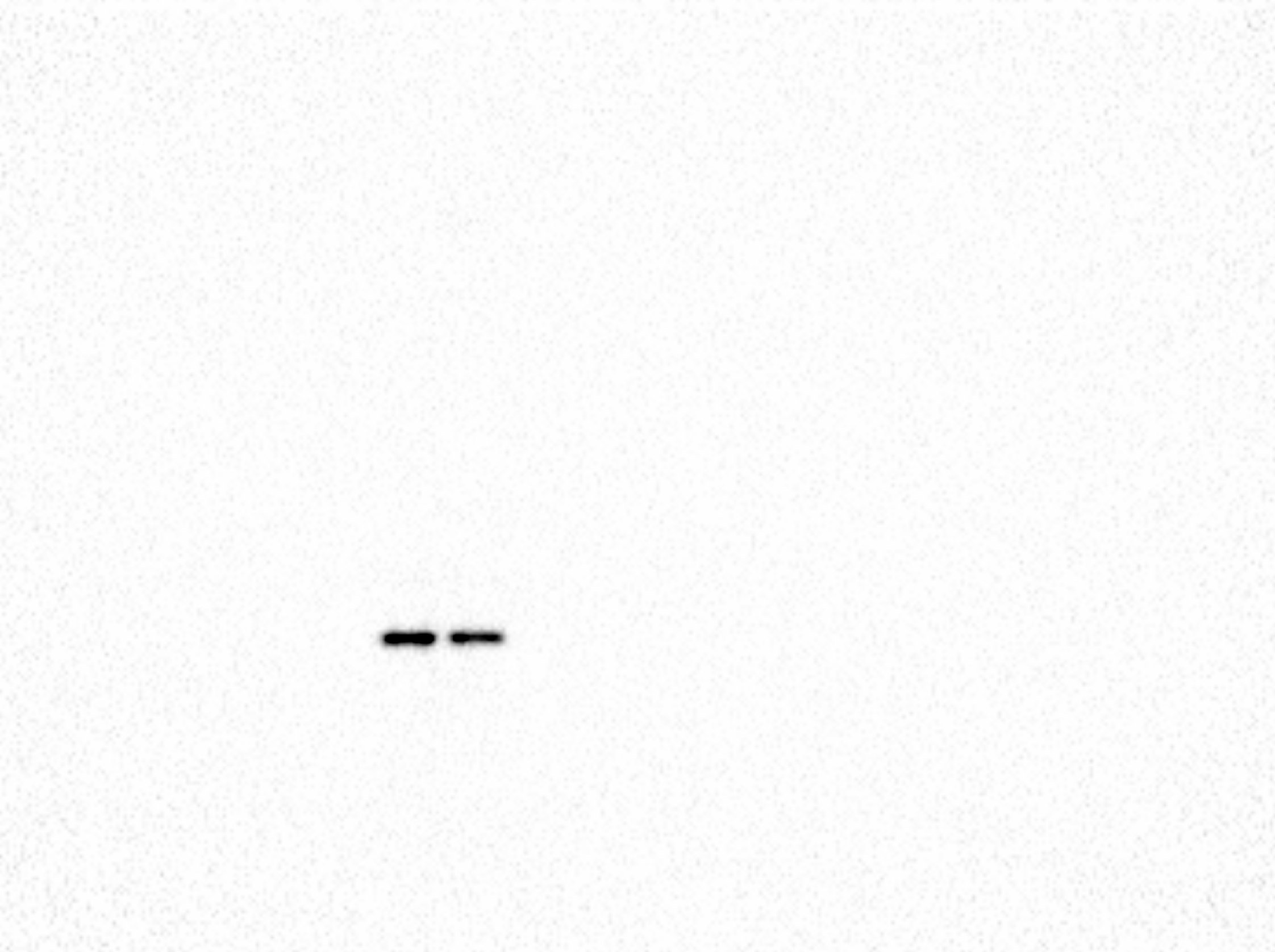

Supplement: Supplementary file 6 — Source data Fig. 2 [file 44318_2026_818_MOESM6_ESM.zip › Figure 2/Figure 2E/Figure 2E Replicate 2/GAPDH.tif]

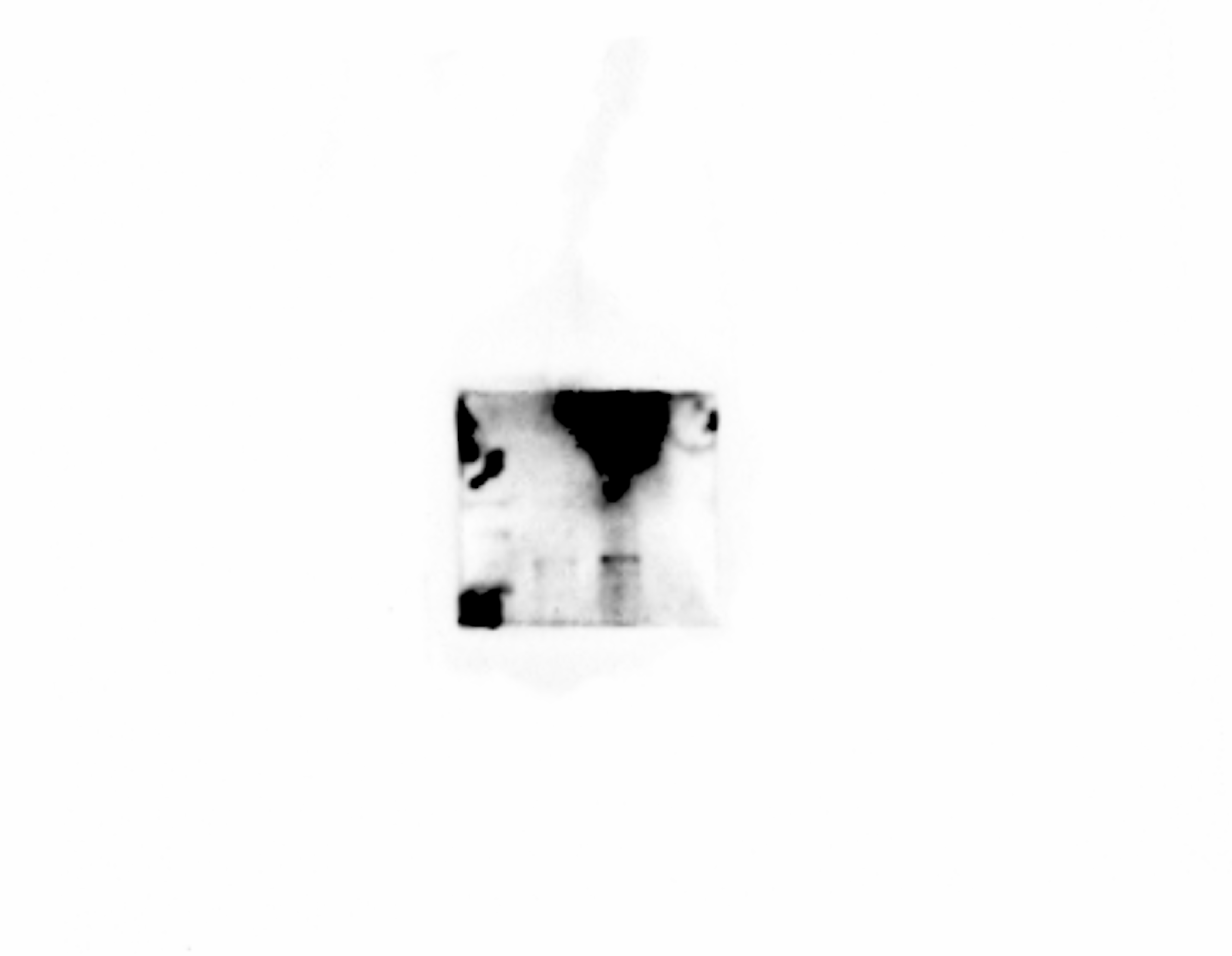

Supplement: Supplementary file 6 — Source data Fig. 2 [file 44318_2026_818_MOESM6_ESM.zip › Figure 2/Figure 2E/Figure 2E Replicate 3/APP.tif]

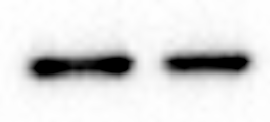

Supplement: Supplementary file 6 — Source data Fig. 2 [file 44318_2026_818_MOESM6_ESM.zip › Figure 2/Figure 2E/Figure 2E Replicate 3/GAPDH.tif]

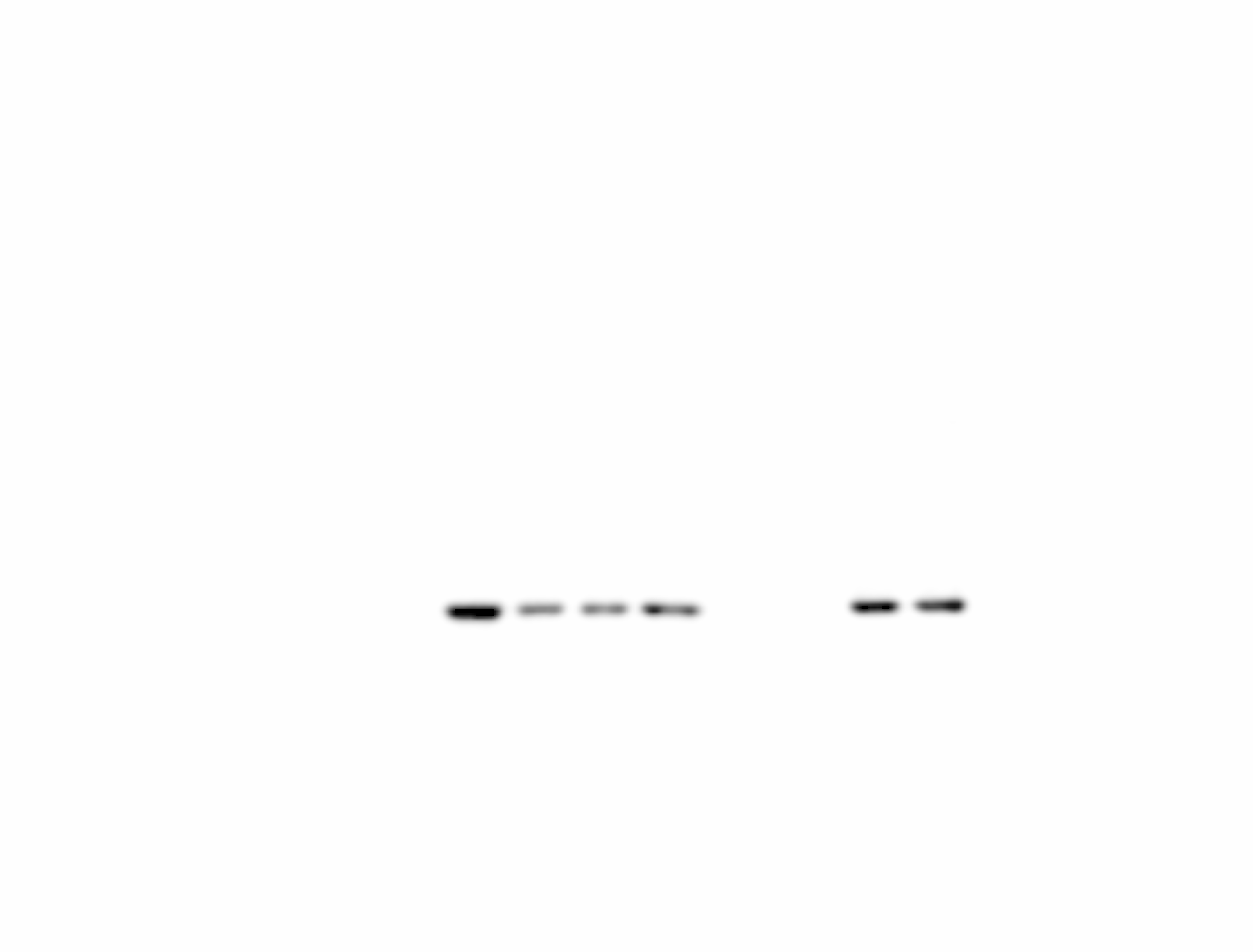

Supplement: Supplementary file 6 — Source data Fig. 2 [file 44318_2026_818_MOESM6_ESM.zip › Figure 2/Figure 2E/GAPDH (lane 5-6).tif]

Figure 2E

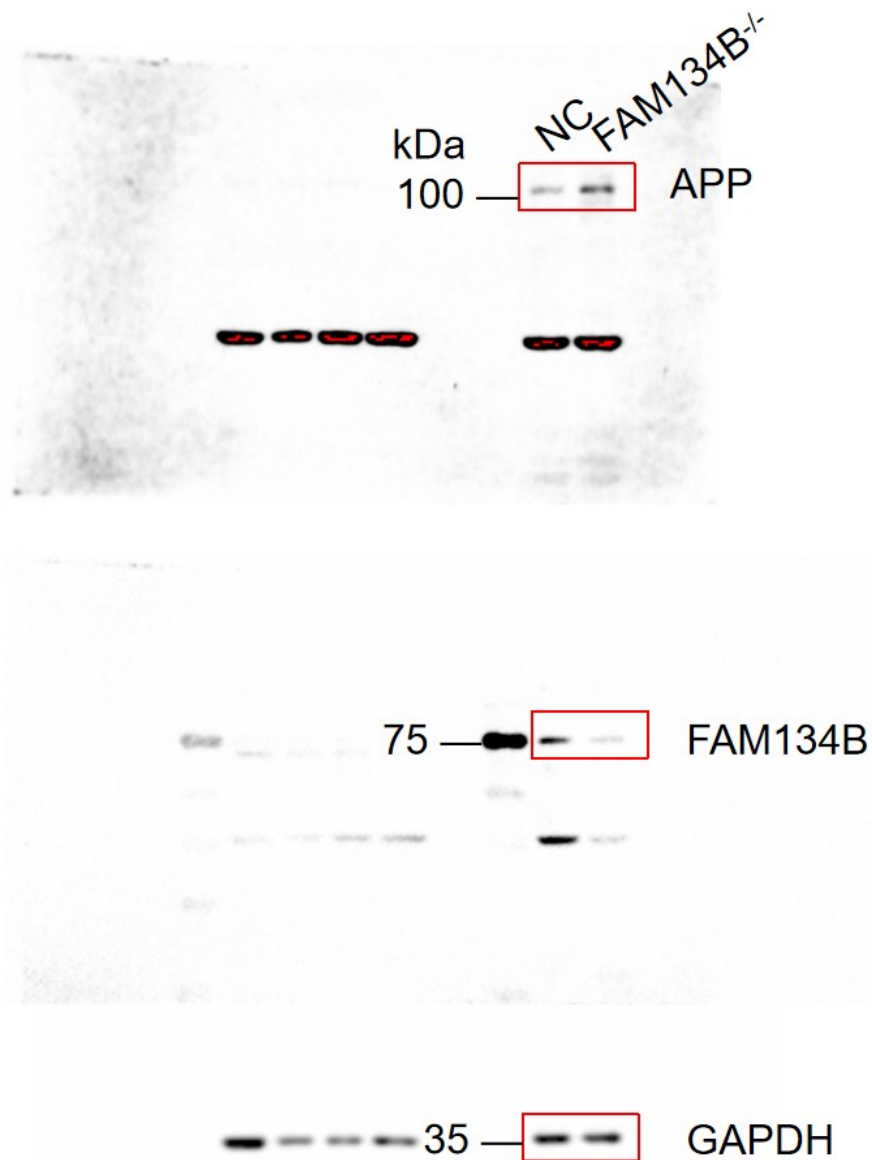

# Replicate 1

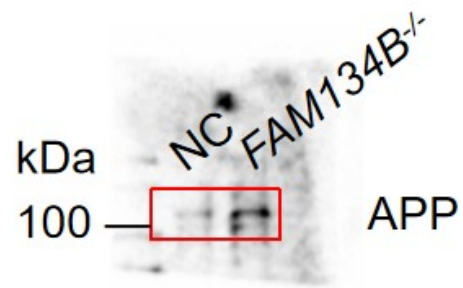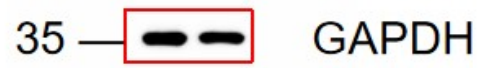

## Replicate 2

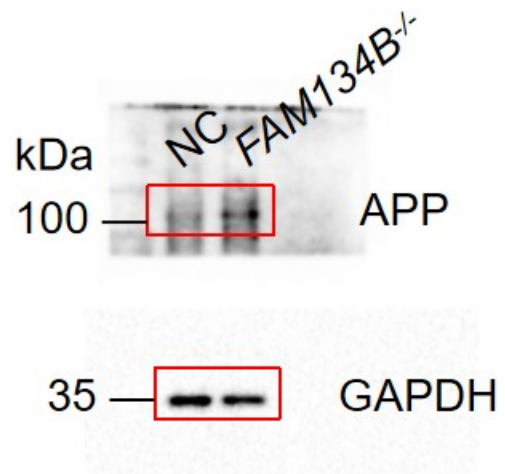

Replicate 3

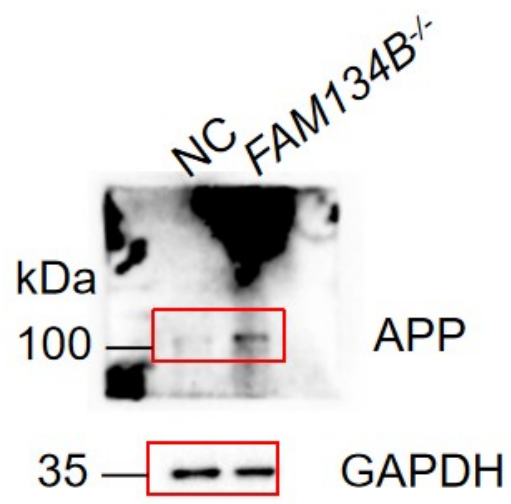

Supplement: Supplementary file 6 — Source data Fig. 2 [file 44318_2026_818_MOESM6_ESM.zip › Figure 2/Figure 2E/WB for Figure 2E.pdf]

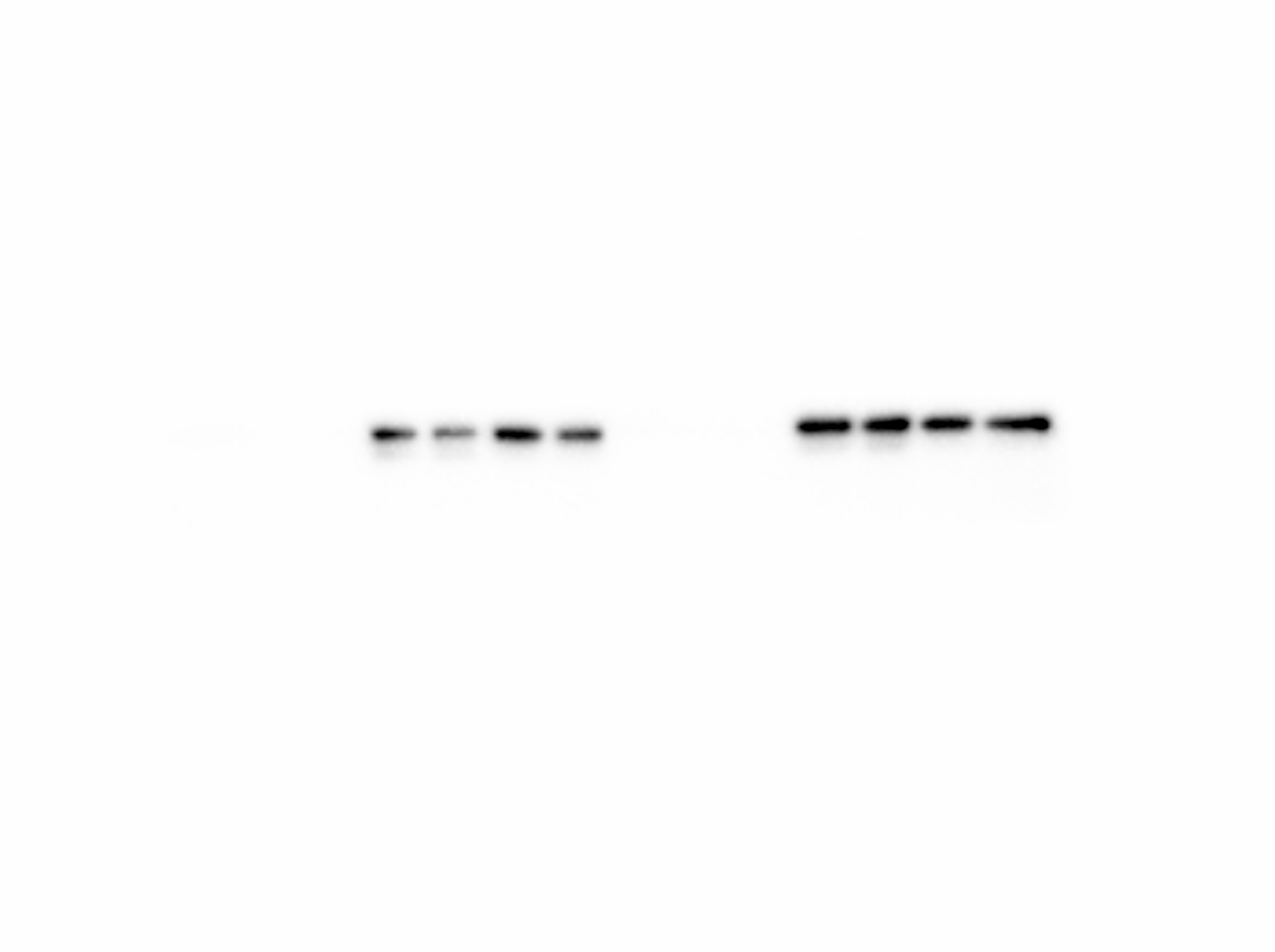

Supplement: Supplementary file 6 — Source data Fig. 2 [file 44318_2026_818_MOESM6_ESM.zip › Figure 2/Figure 2J/3HA-FAM134B (lane 7-12).tif]

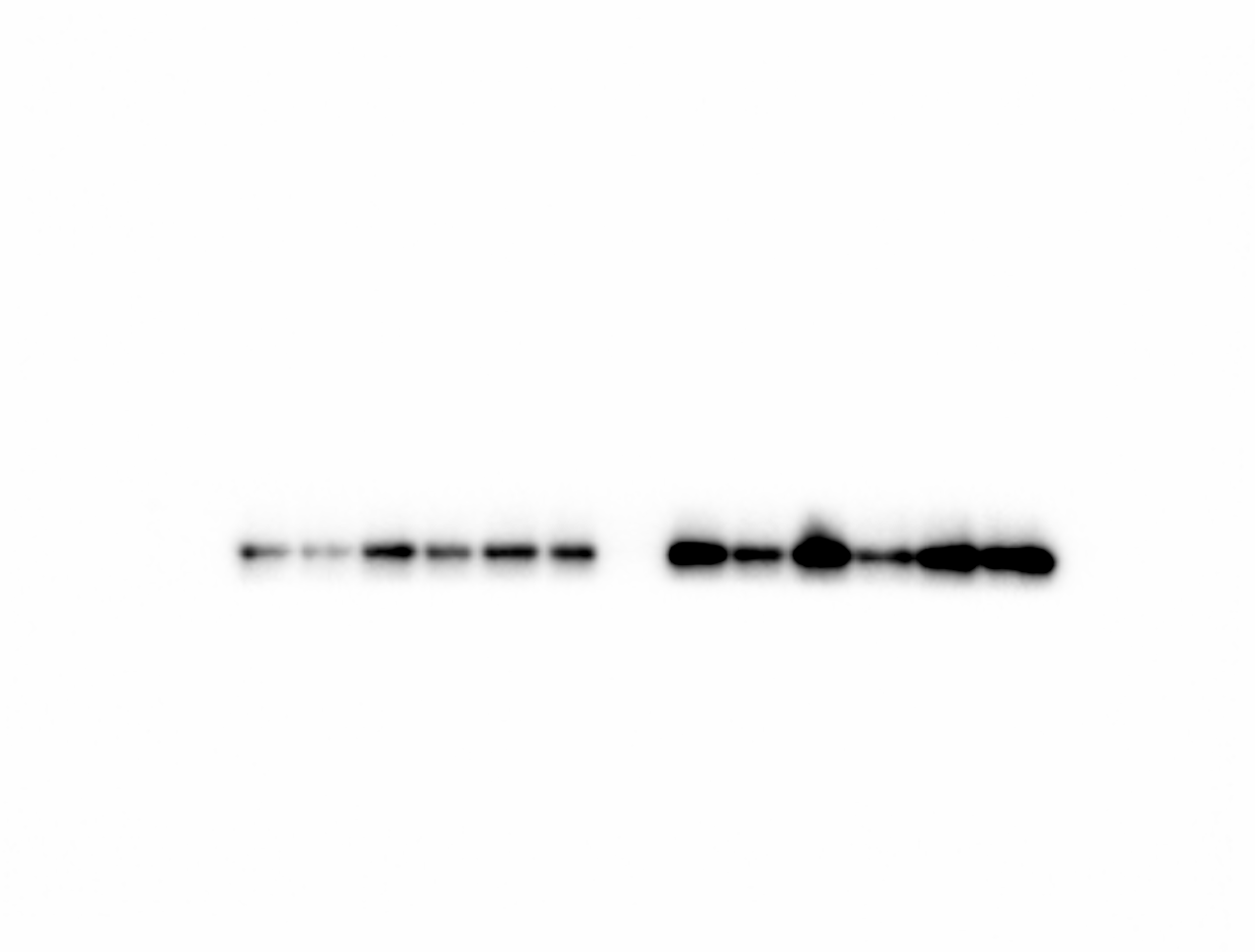

Supplement: Supplementary file 6 — Source data Fig. 2 [file 44318_2026_818_MOESM6_ESM.zip › Figure 2/Figure 2J/APPWT-mCherry (lane 7-12).tif]

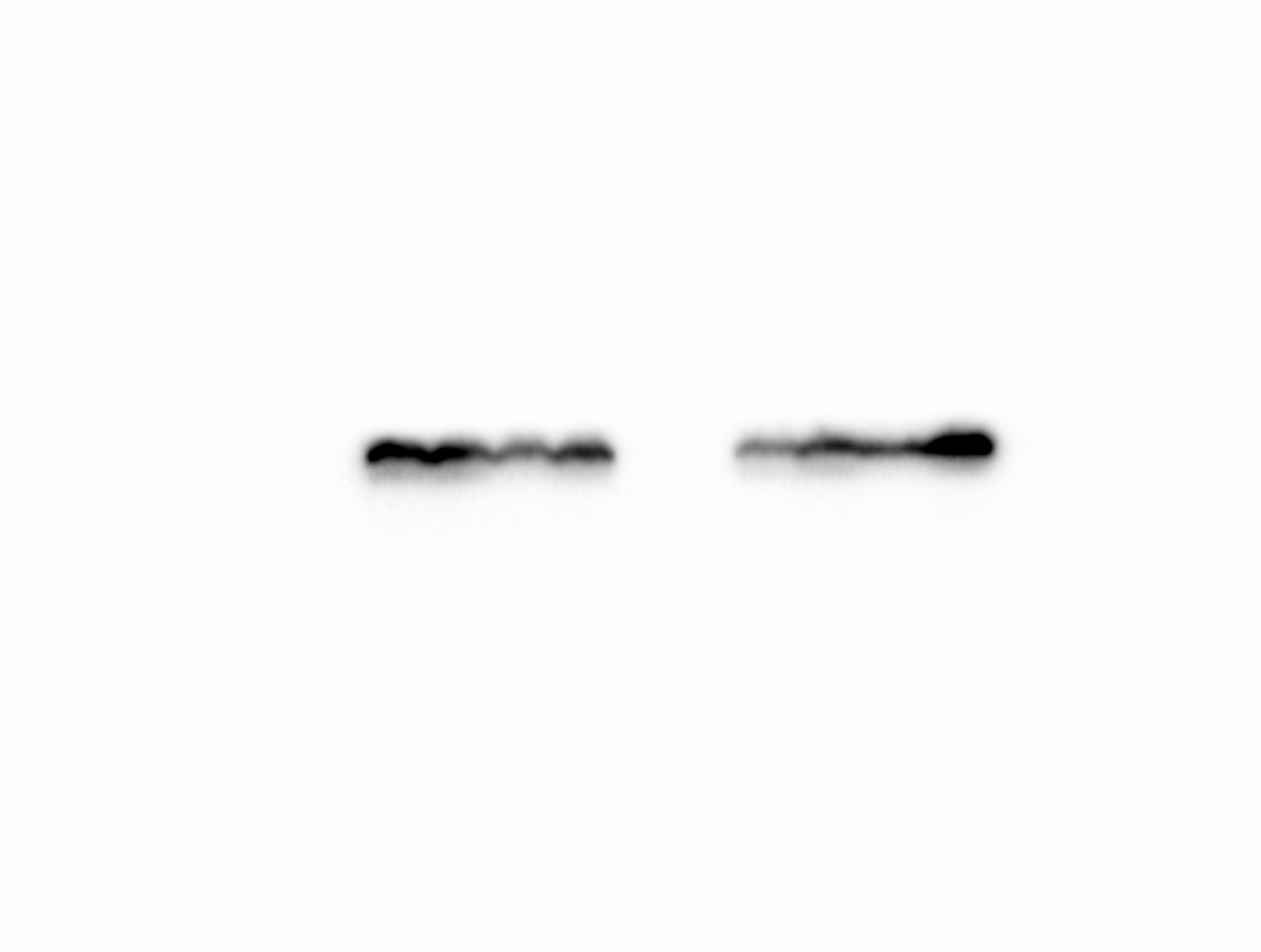

Supplement: Supplementary file 6 — Source data Fig. 2 [file 44318_2026_818_MOESM6_ESM.zip › Figure 2/Figure 2J/Figure 2J Replicate 1/3HA-FAM134B (lane 7-12).tif]

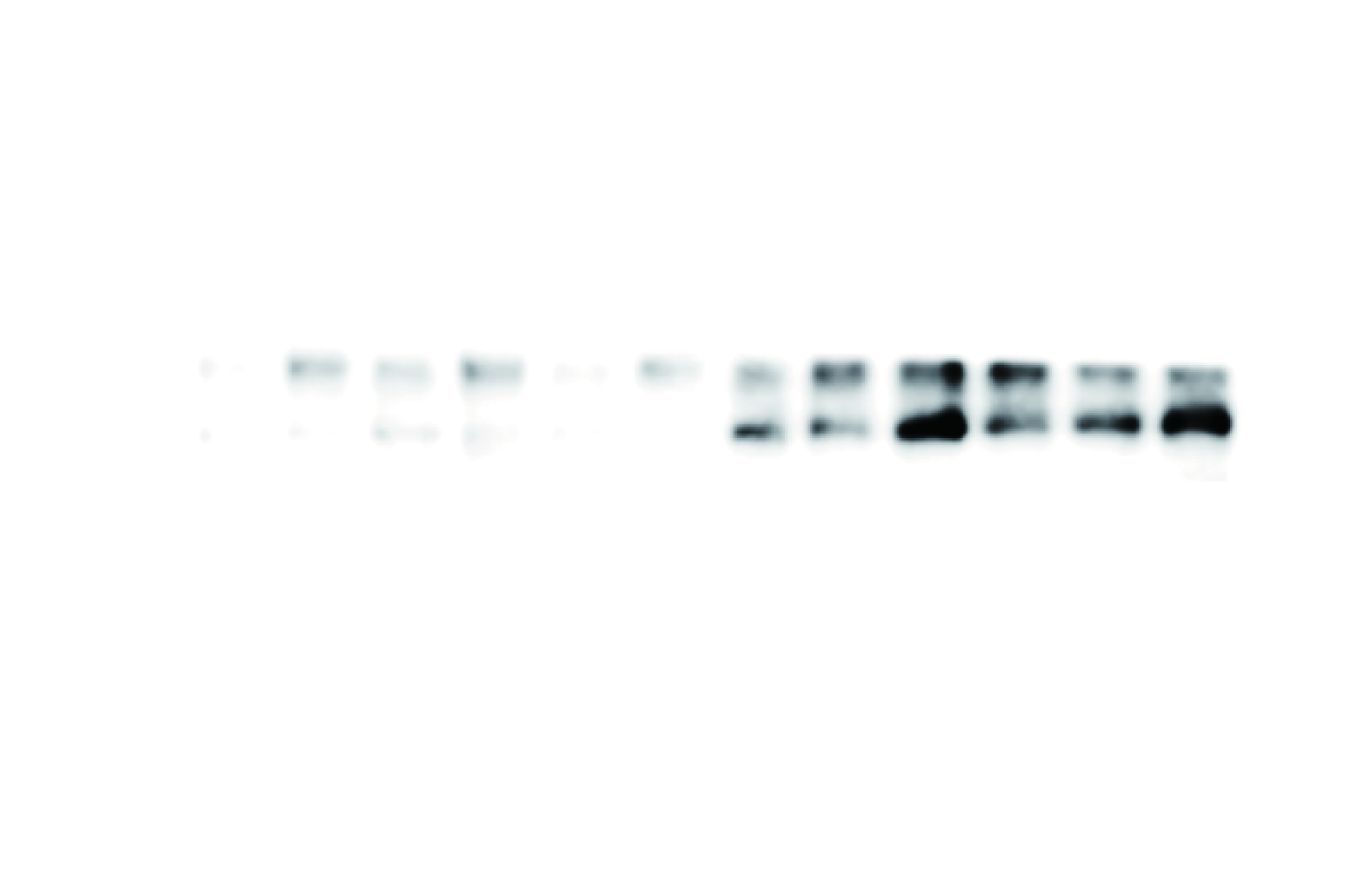

Supplement: Supplementary file 6 — Source data Fig. 2 [file 44318_2026_818_MOESM6_ESM.zip › Figure 2/Figure 2J/Figure 2J Replicate 1/APPWT-mCherry (lane 7-12).tif]

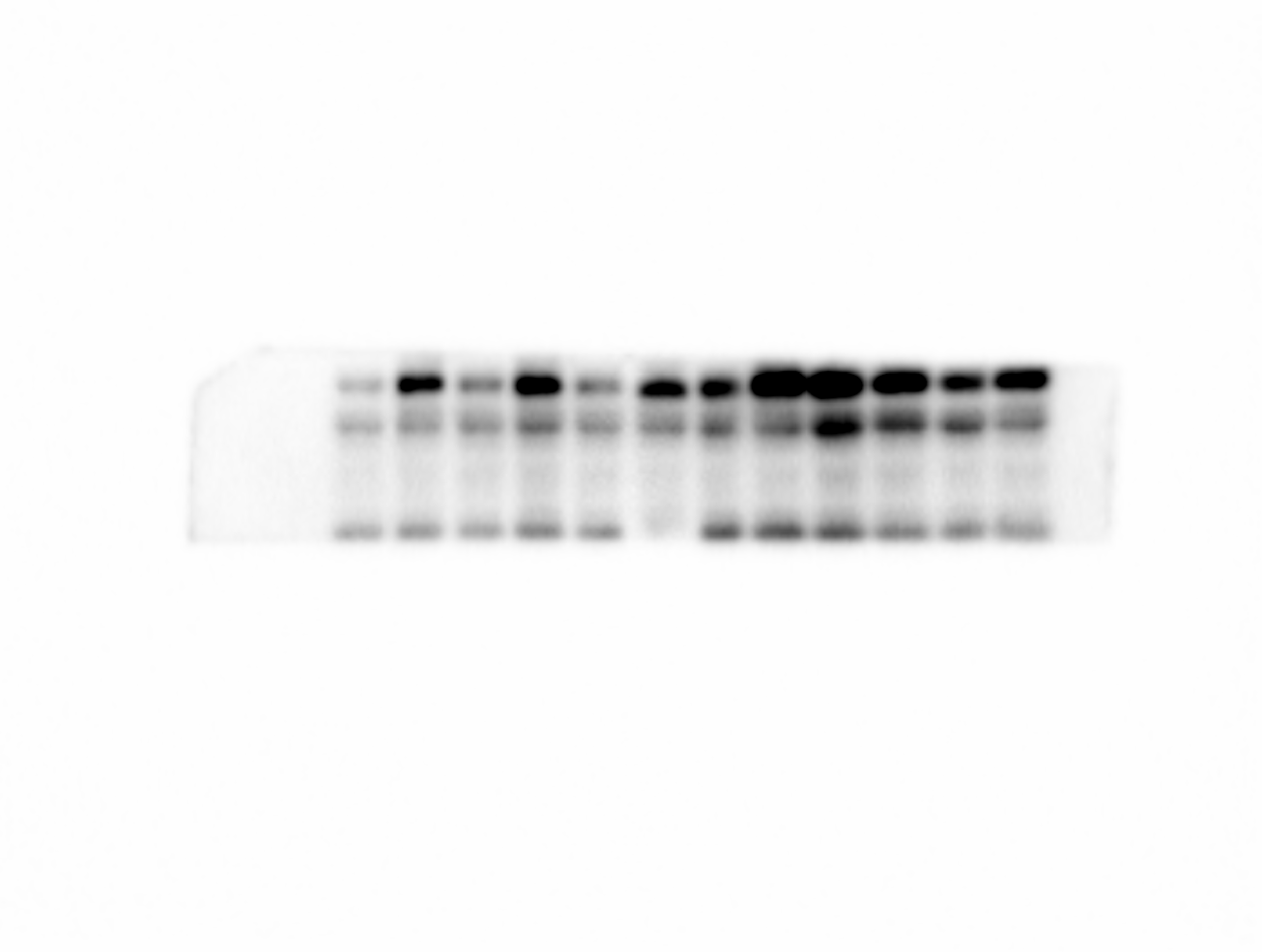

Supplement: Supplementary file 6 — Source data Fig. 2 [file 44318_2026_818_MOESM6_ESM.zip › Figure 2/Figure 2J/Figure 2J Replicate 1/GAPDH (lane 7-12).tif]

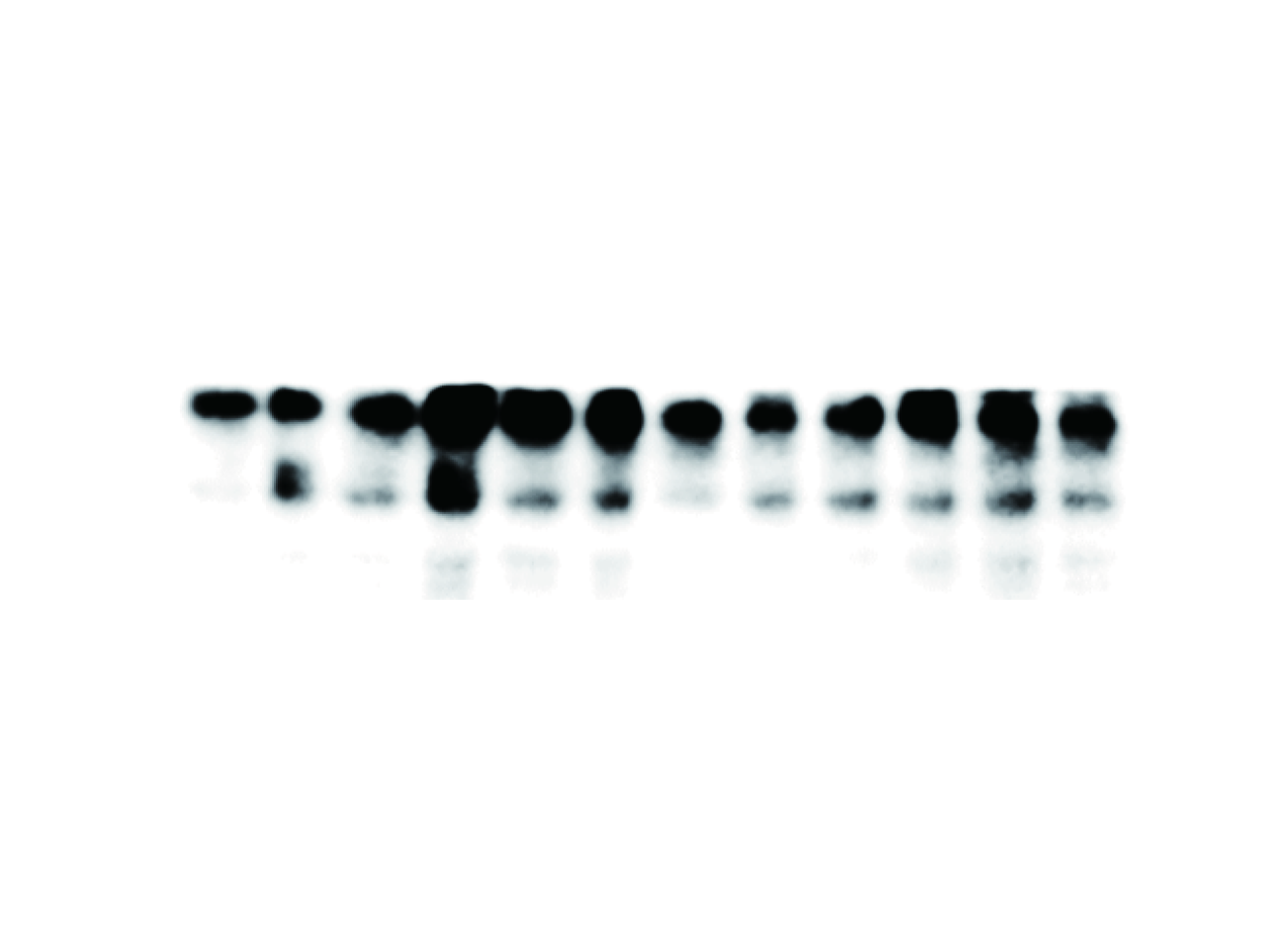

Supplement: Supplementary file 6 — Source data Fig. 2 [file 44318_2026_818_MOESM6_ESM.zip › Figure 2/Figure 2J/Figure 2J Replicate 1/mCherry (lane 7-12).tif]

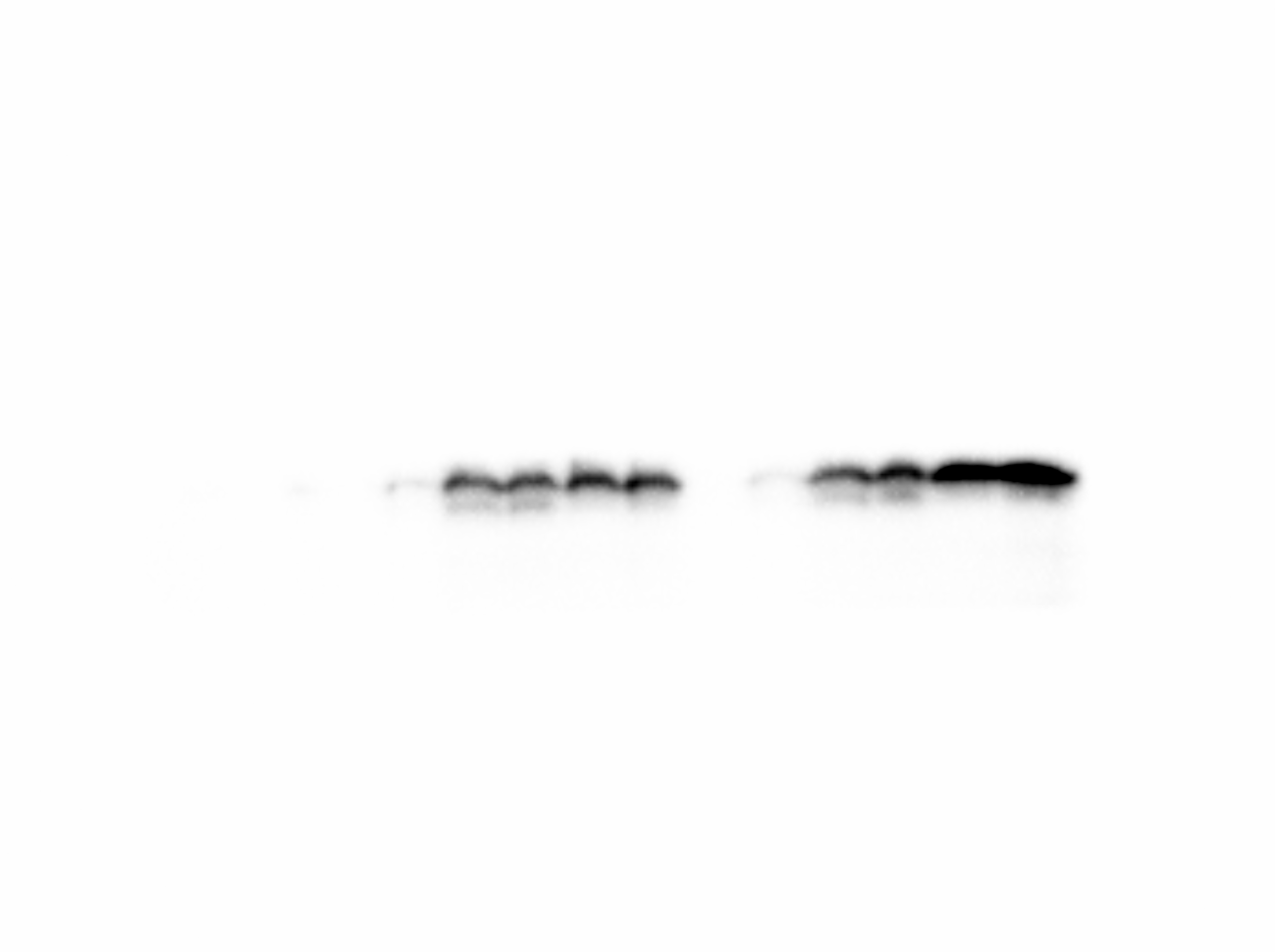

Supplement: Supplementary file 6 — Source data Fig. 2 [file 44318_2026_818_MOESM6_ESM.zip › Figure 2/Figure 2J/Figure 2J Replicate 2/3HA-FAM134B (lane 7-12).tif]

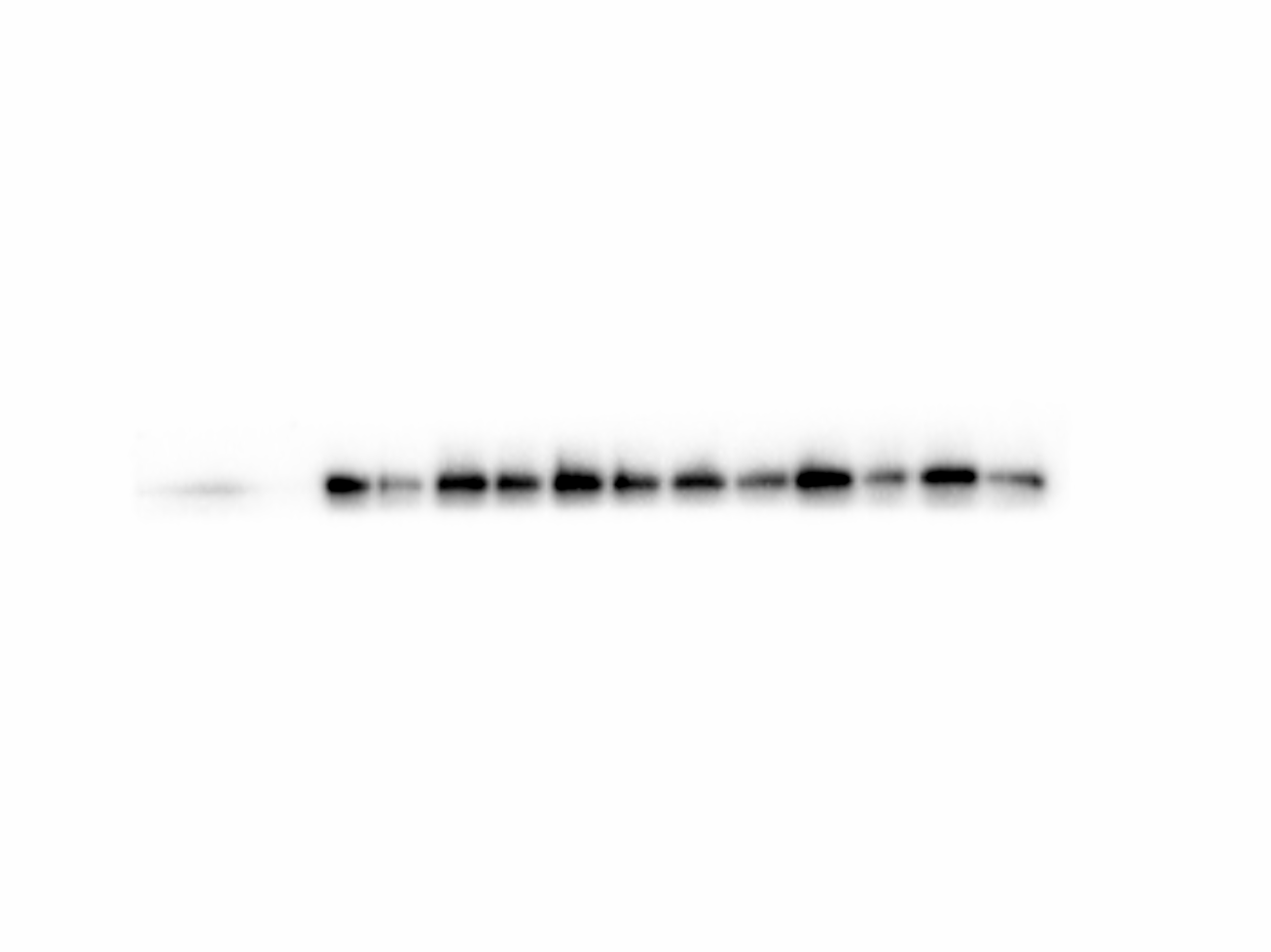

Supplement: Supplementary file 6 — Source data Fig. 2 [file 44318_2026_818_MOESM6_ESM.zip › Figure 2/Figure 2J/Figure 2J Replicate 2/APPWT-mCherry (lane 7-12).tif]

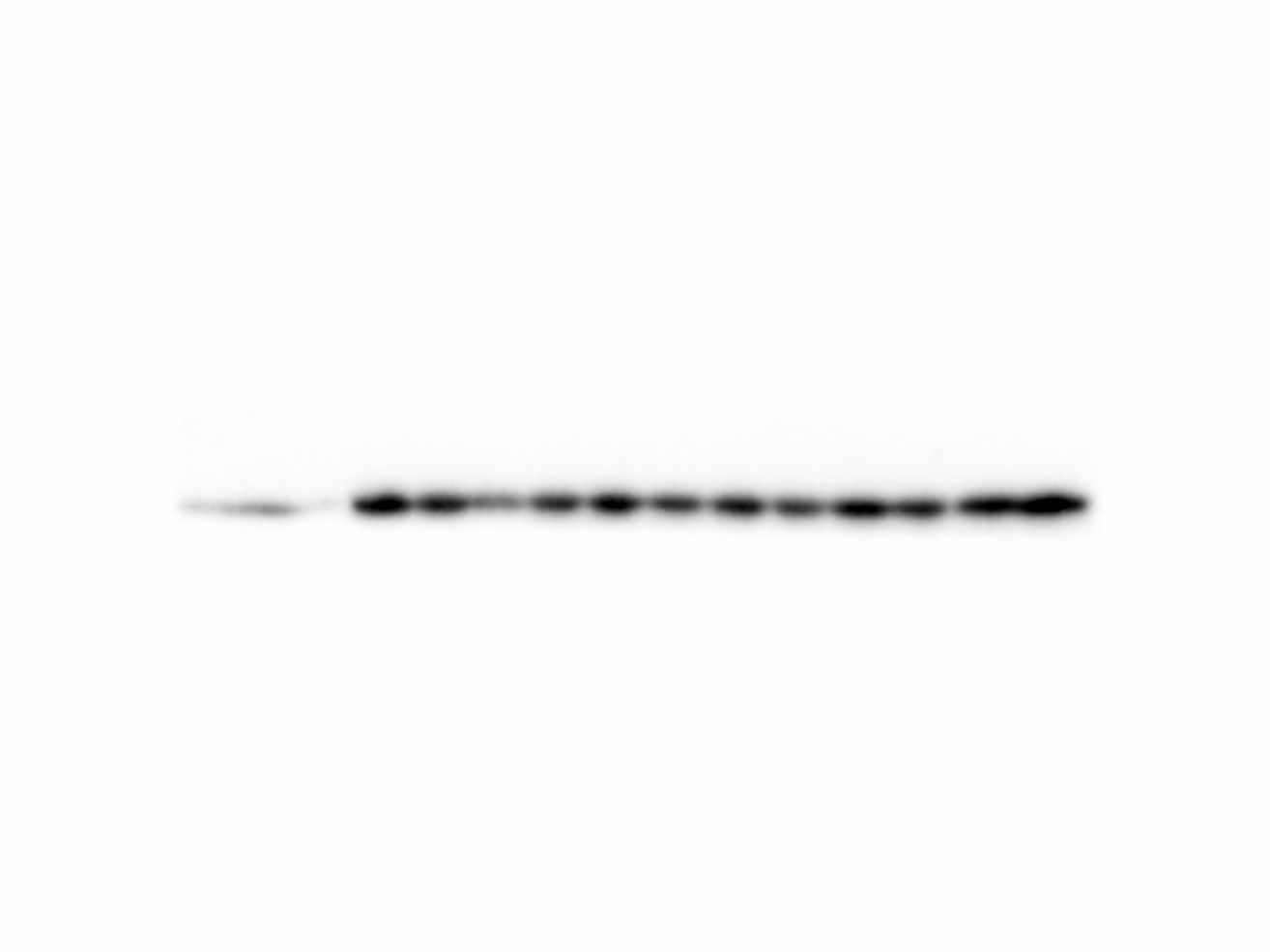

Supplement: Supplementary file 6 — Source data Fig. 2 [file 44318_2026_818_MOESM6_ESM.zip › Figure 2/Figure 2J/Figure 2J Replicate 2/GAPDH (lane 7-12).tif]

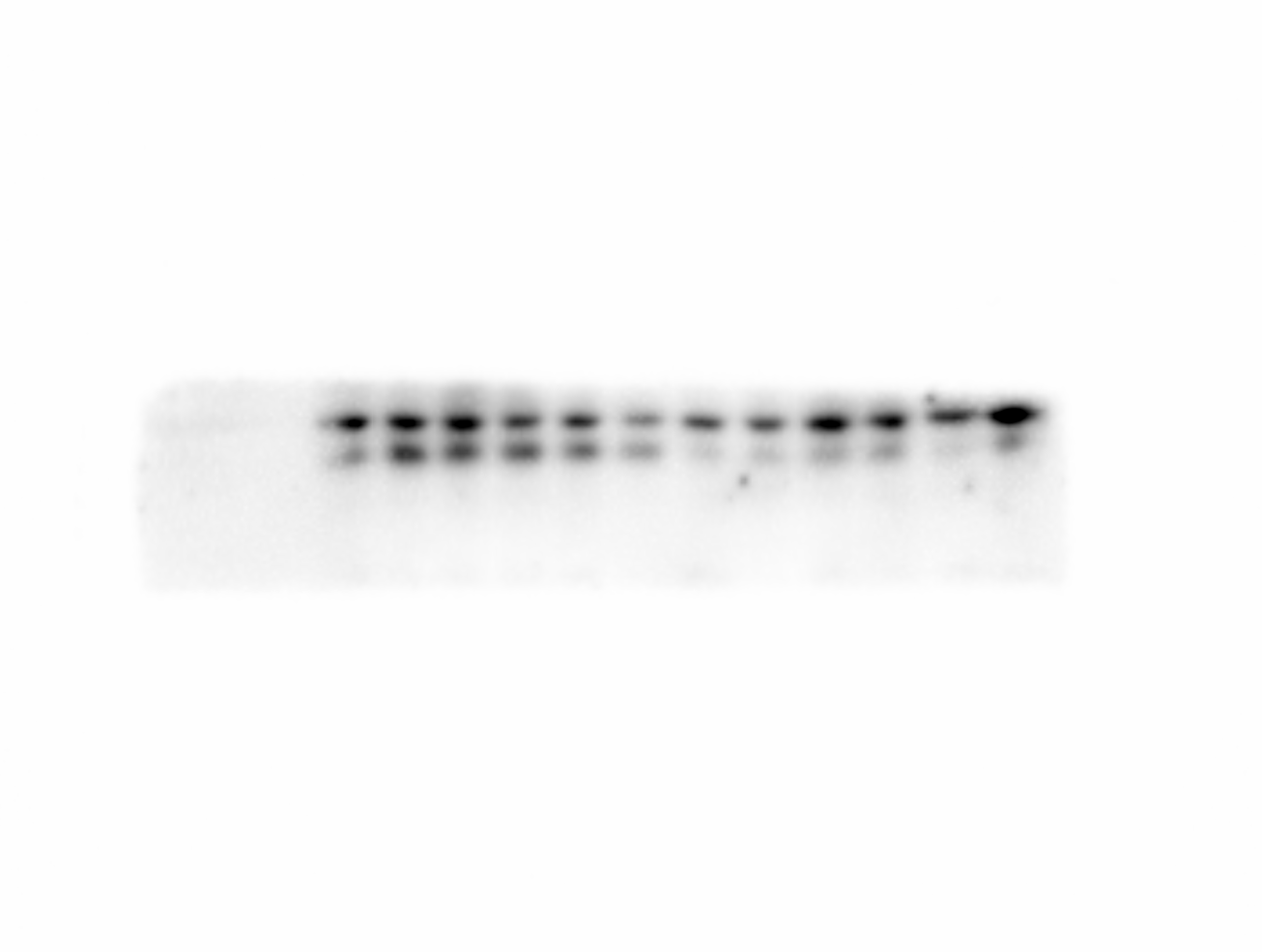

Supplement: Supplementary file 6 — Source data Fig. 2 [file 44318_2026_818_MOESM6_ESM.zip › Figure 2/Figure 2J/Figure 2J Replicate 2/mCherry (lane 7-12).tif]

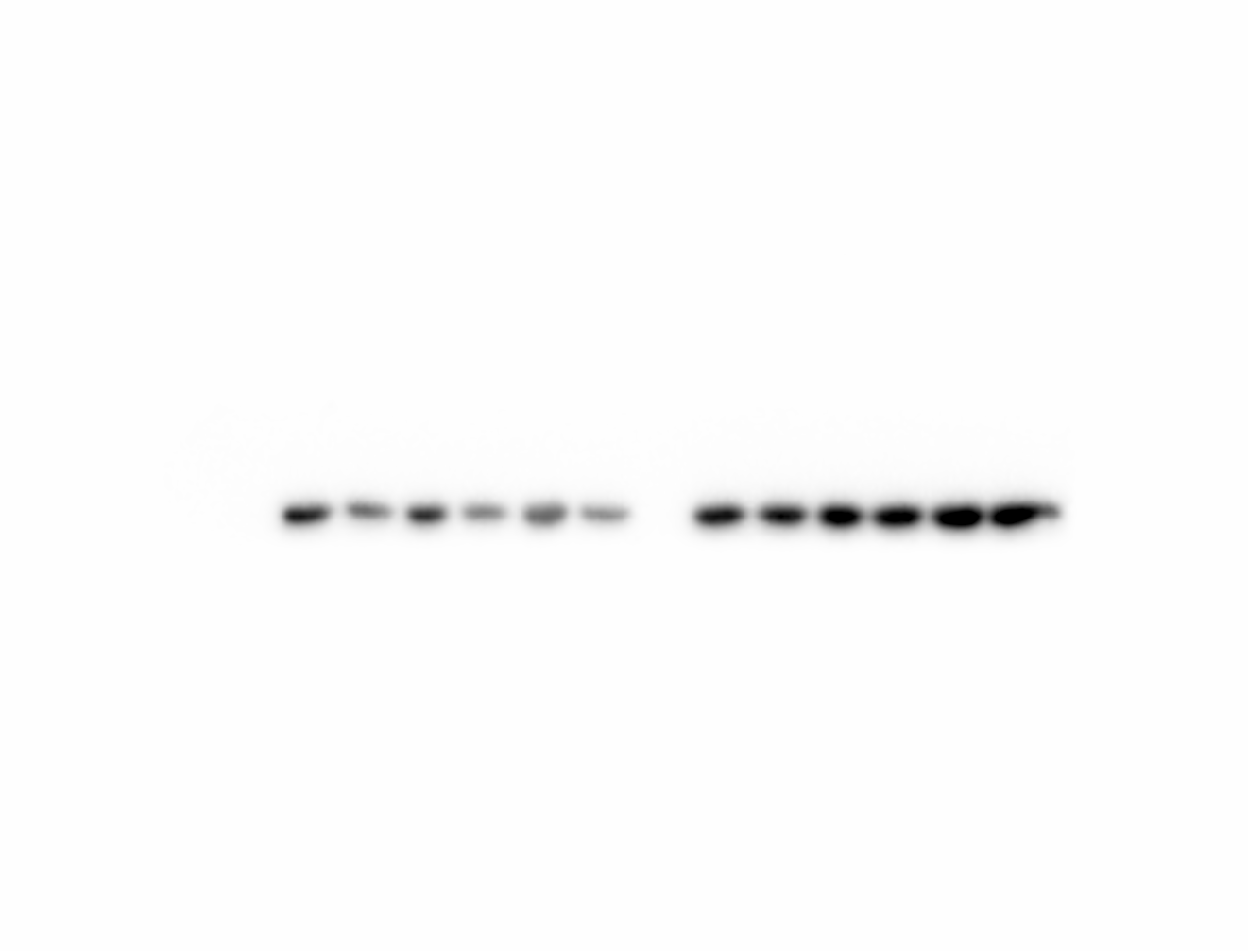

Supplement: Supplementary file 6 — Source data Fig. 2 [file 44318_2026_818_MOESM6_ESM.zip › Figure 2/Figure 2J/GAPDH (lane 7-12).tif]

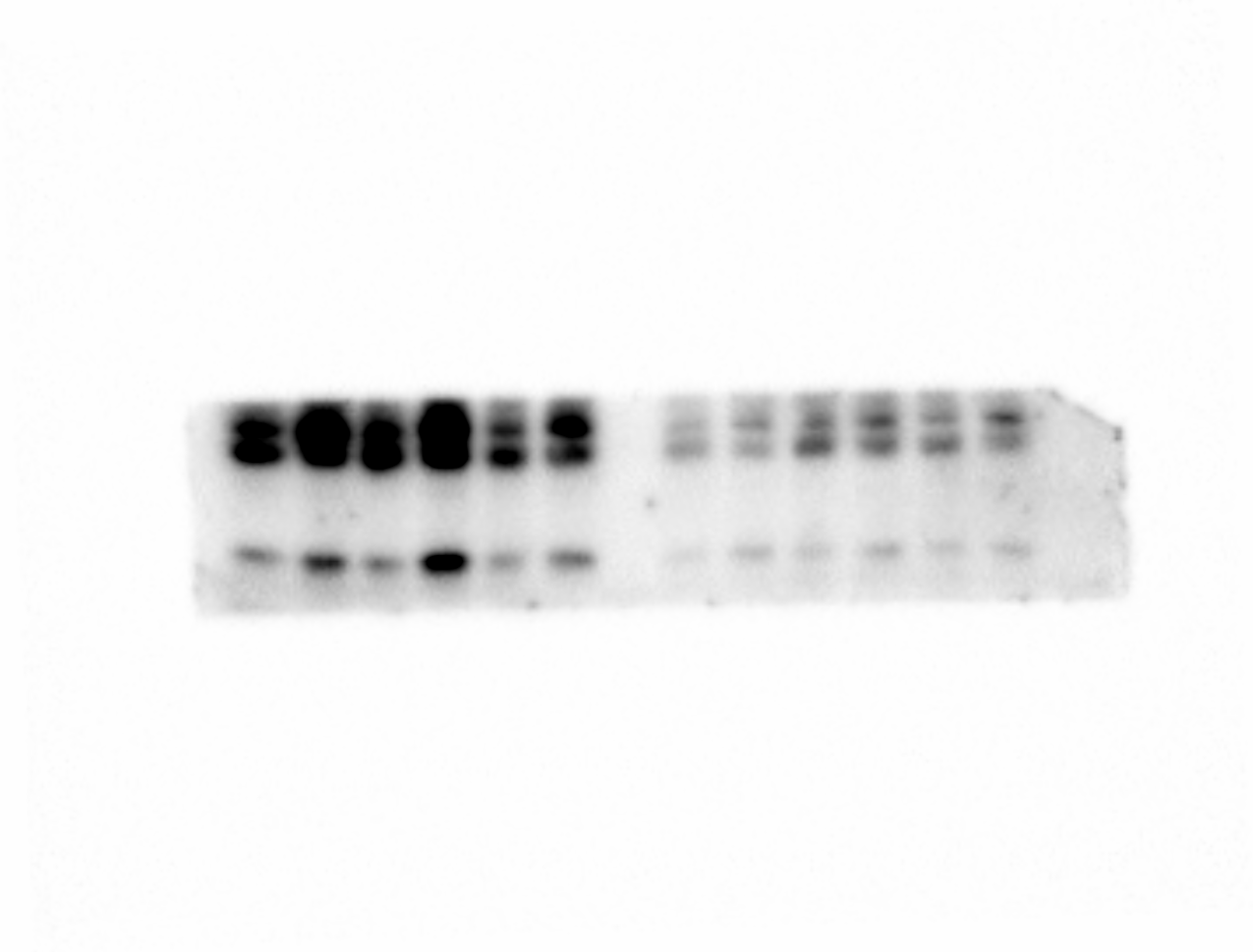

Supplement: Supplementary file 6 — Source data Fig. 2 [file 44318_2026_818_MOESM6_ESM.zip › Figure 2/Figure 2J/mCherry (lane 1-6).tif]

Figure 2J

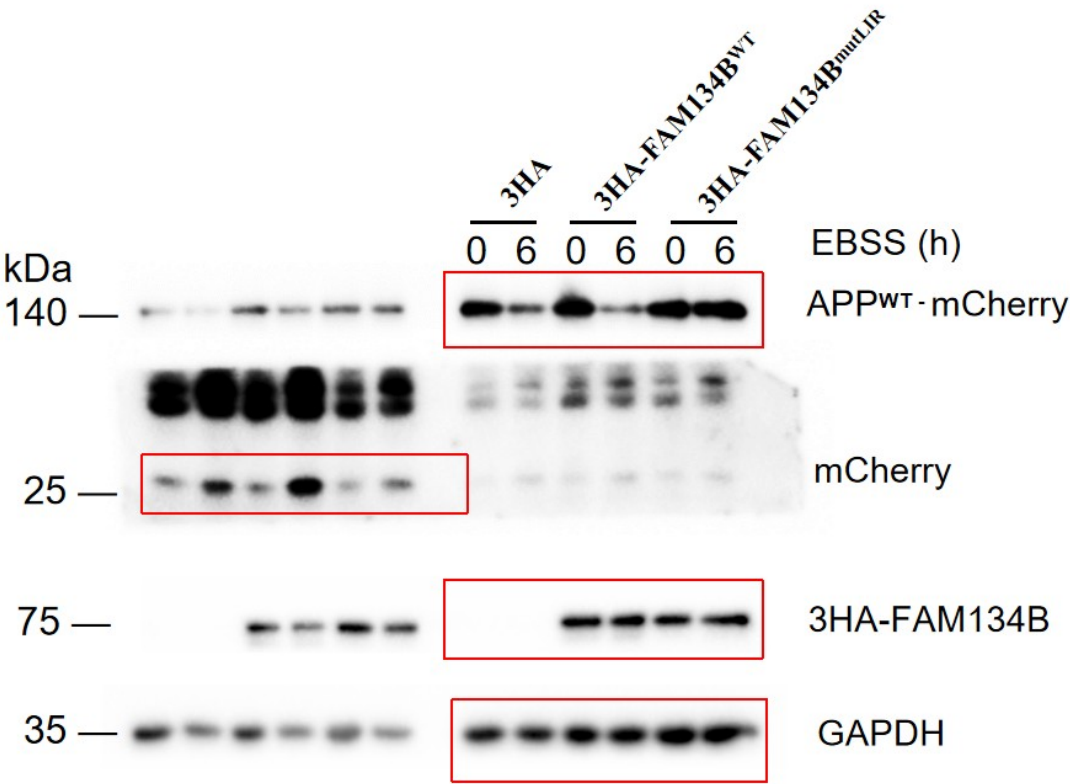

Replicate 1

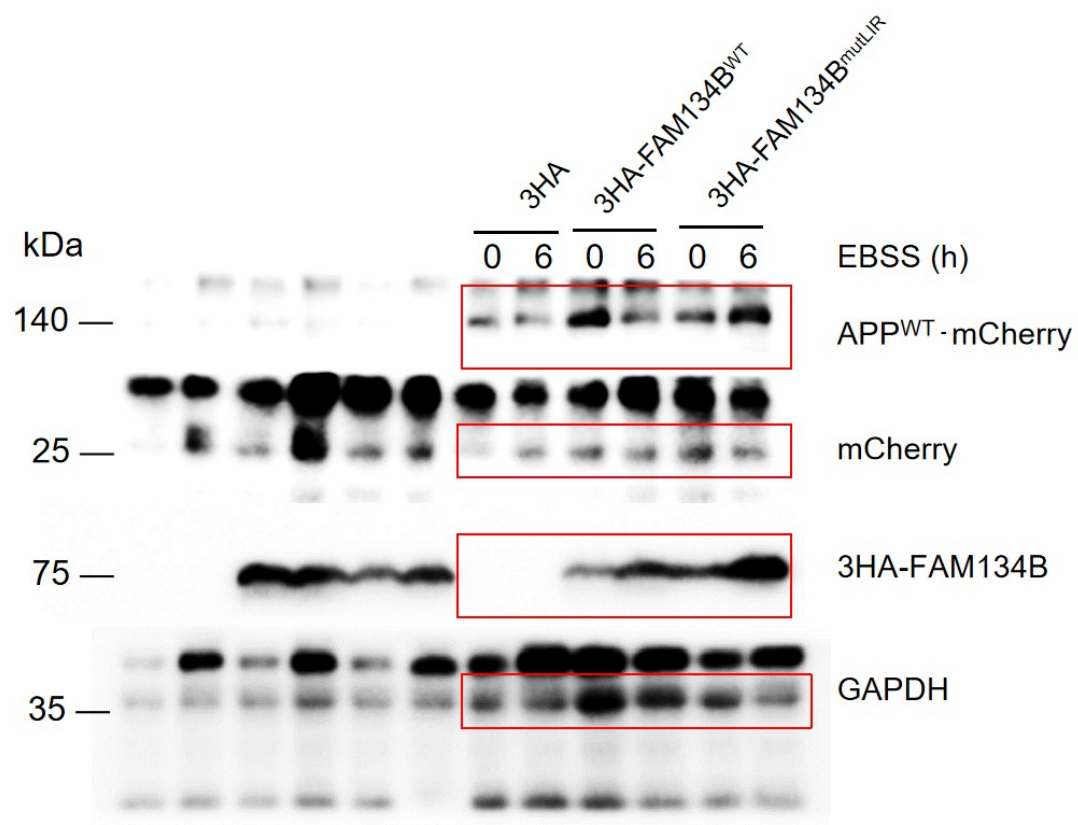

Replicate 2

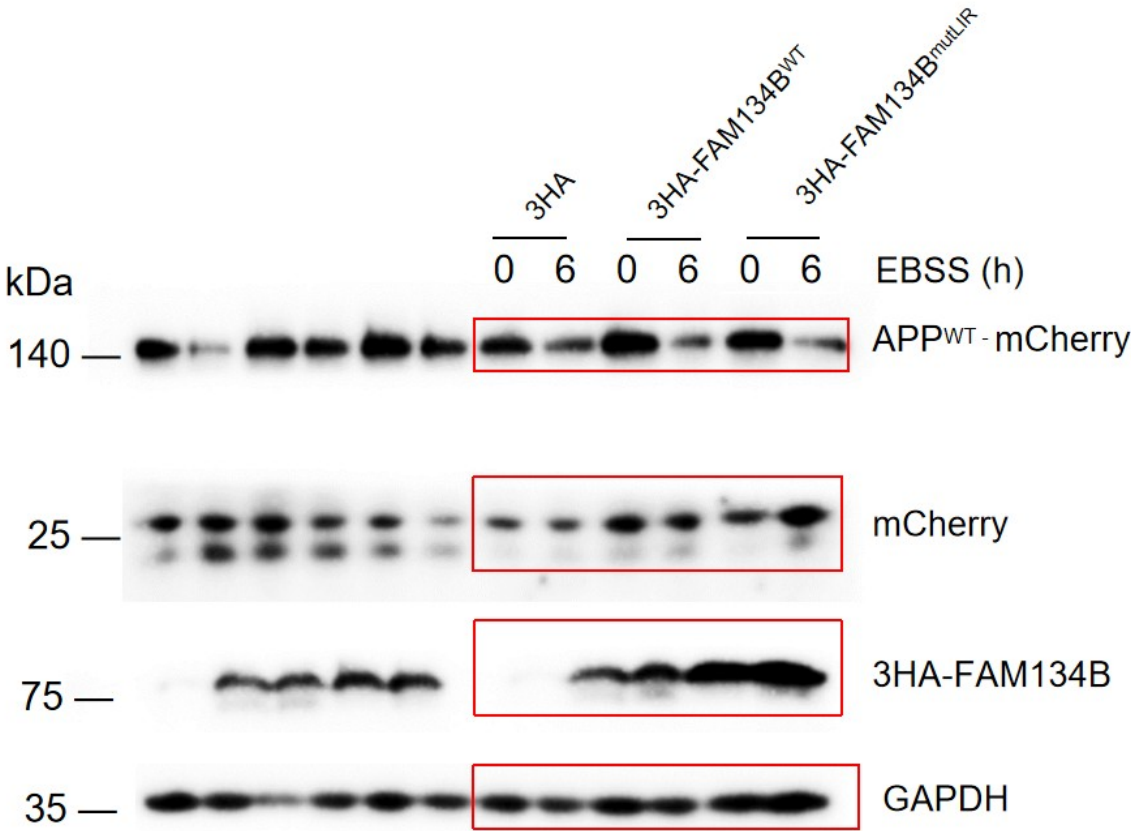

Supplement: Supplementary file 6 — Source data Fig. 2 [file 44318_2026_818_MOESM6_ESM.zip › Figure 2/Figure 2J/WB for Figure 2J.pdf]

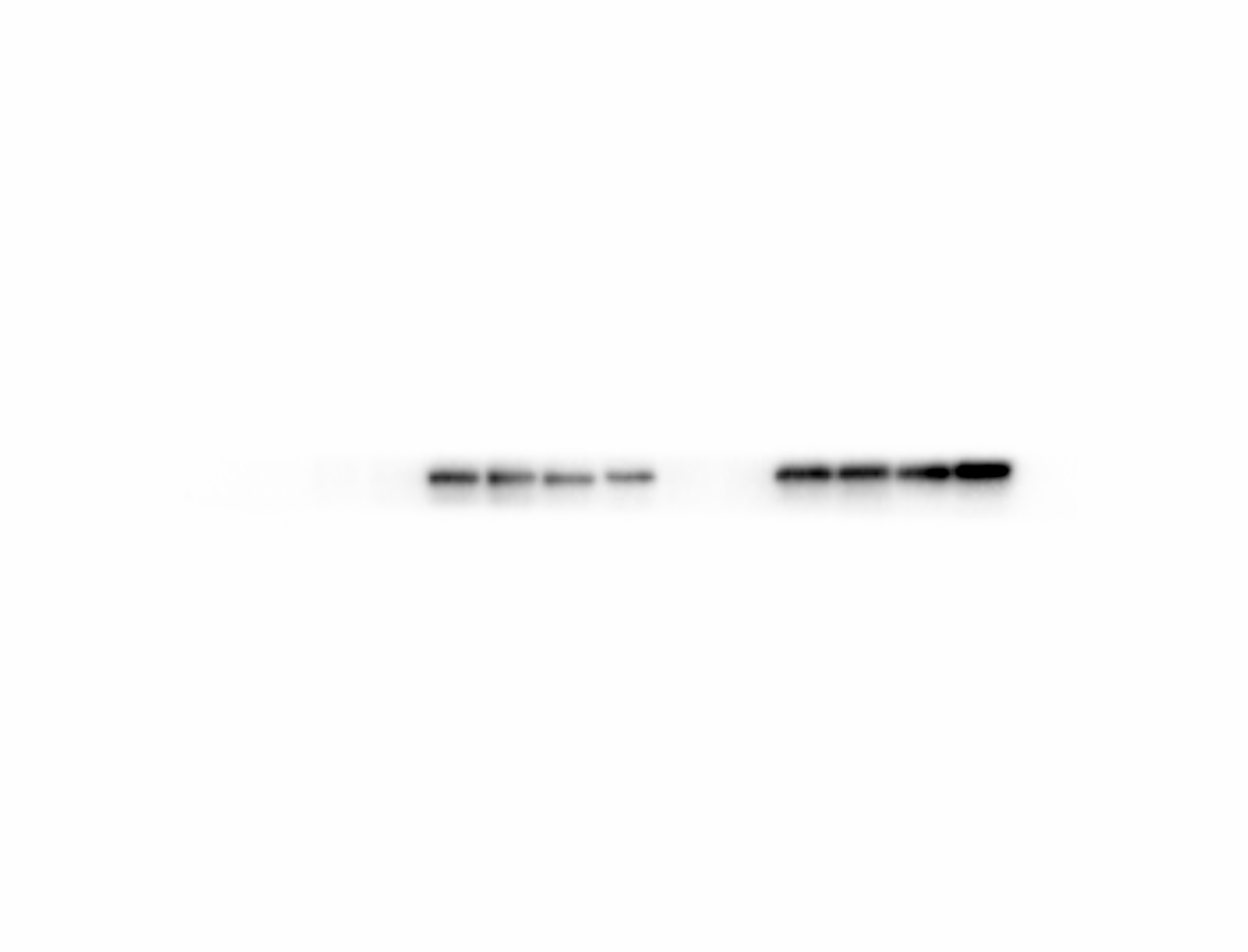

Supplement: Supplementary file 6 — Source data Fig. 2 [file 44318_2026_818_MOESM6_ESM.zip › Figure 2/Figure 2K/3HA-FAM134B (lane 1-6).tif]

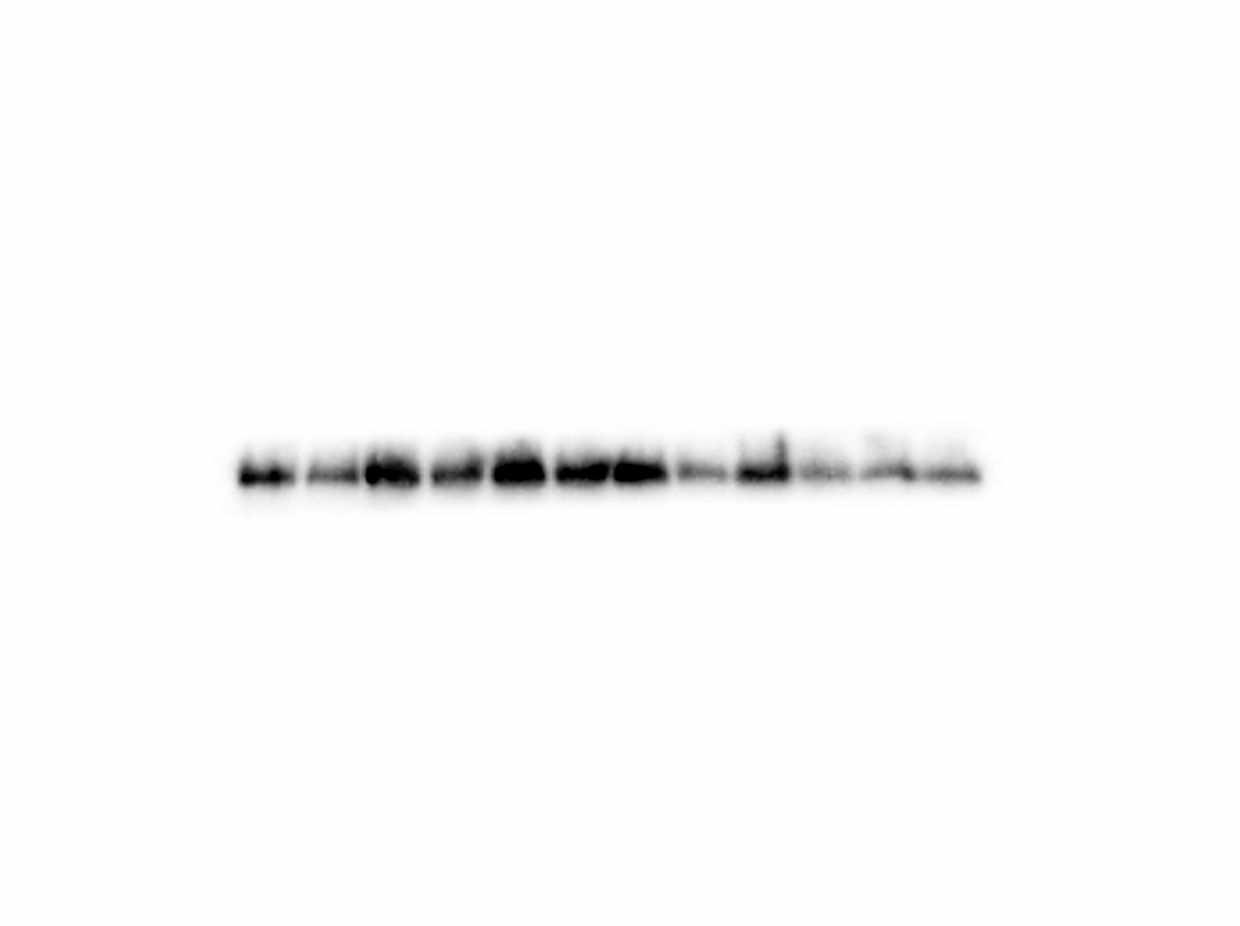

Supplement: Supplementary file 6 — Source data Fig. 2 [file 44318_2026_818_MOESM6_ESM.zip › Figure 2/Figure 2K/APPmut-mCherry (lane 1-6).tif]

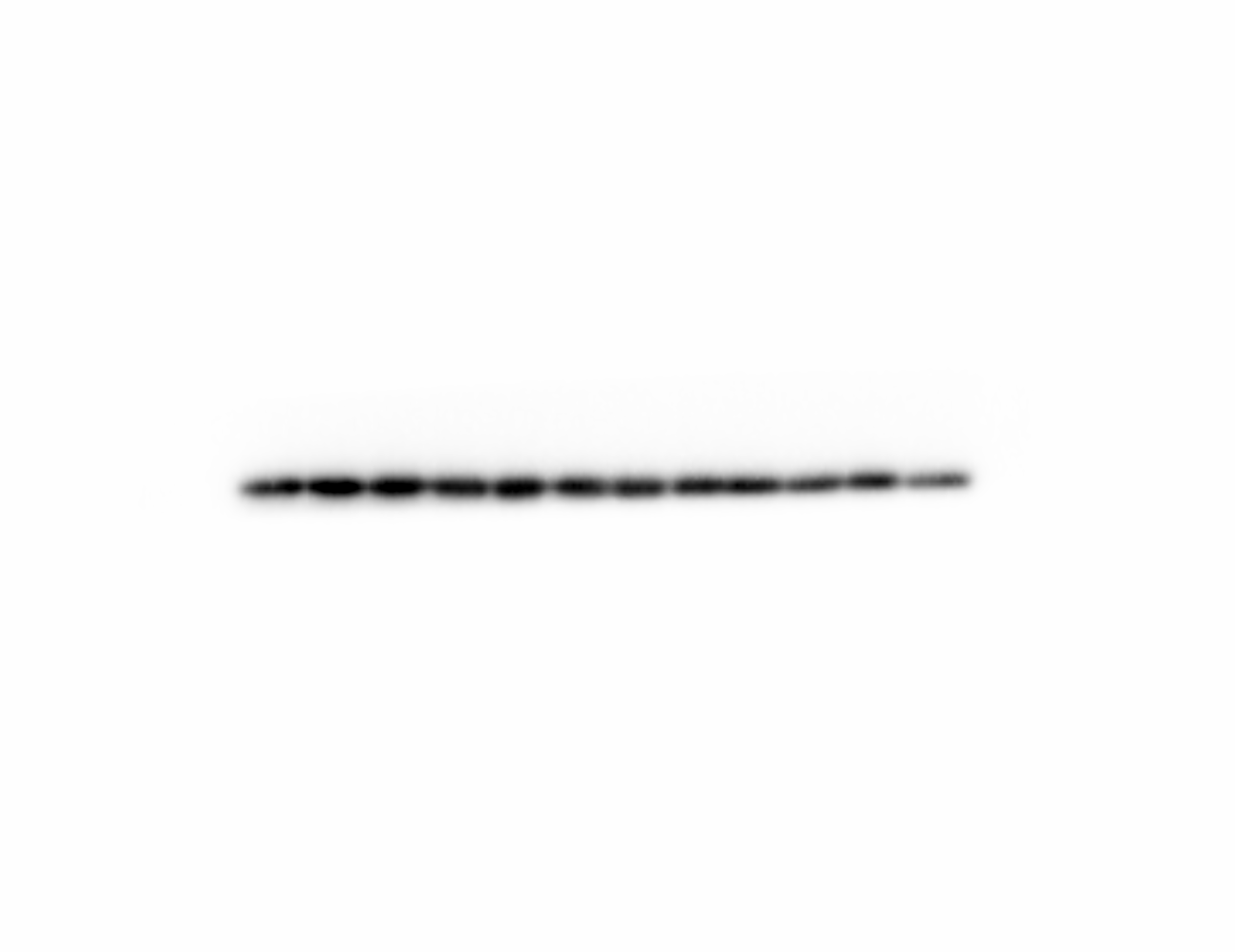

Supplement: Supplementary file 6 — Source data Fig. 2 [file 44318_2026_818_MOESM6_ESM.zip › Figure 2/Figure 2K/Figure 2K Replicate 1/GAPDH (lane 7-12).tif]

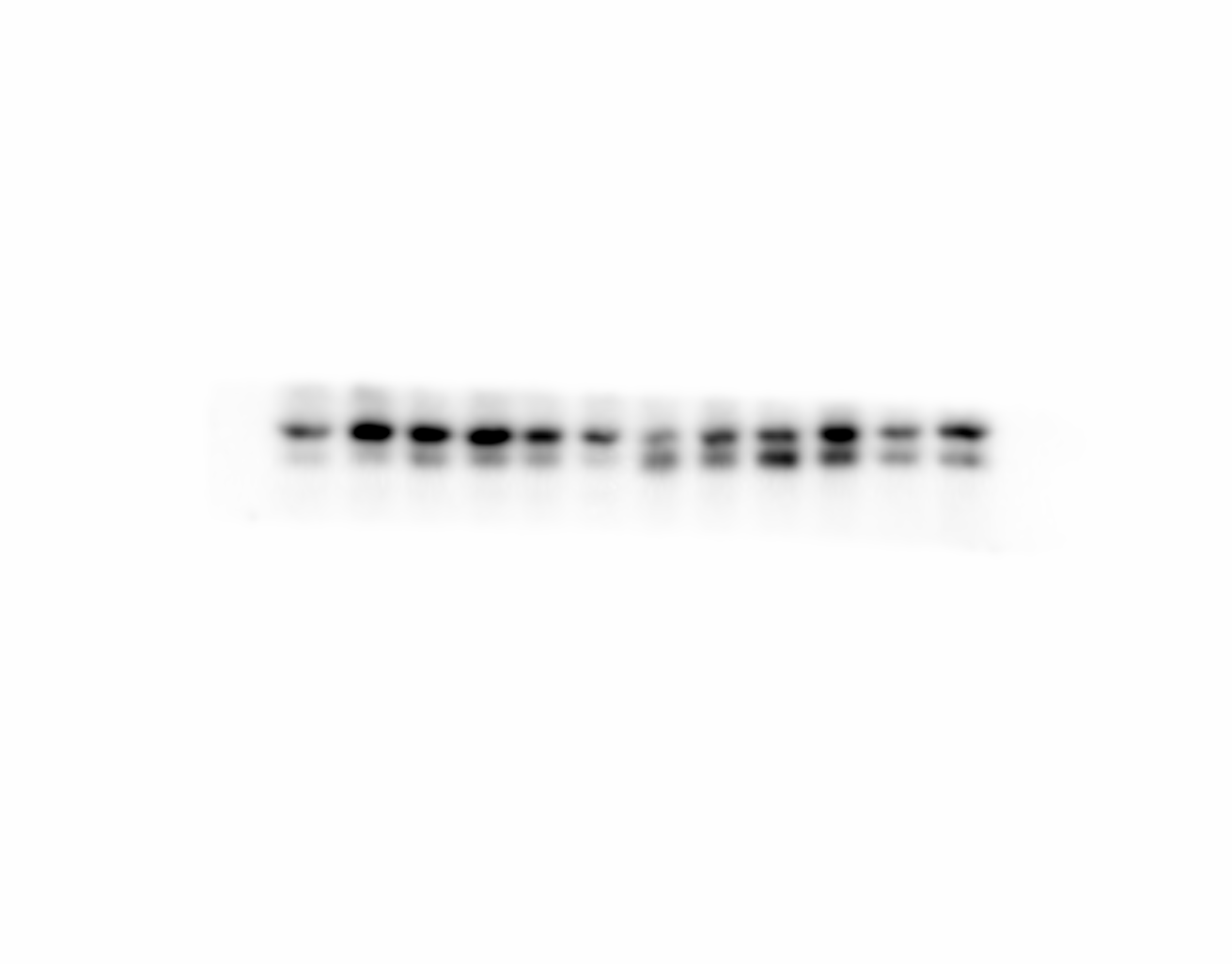

Supplement: Supplementary file 6 — Source data Fig. 2 [file 44318_2026_818_MOESM6_ESM.zip › Figure 2/Figure 2K/Figure 2K Replicate 1/mCherry (lane 7-12).tif]

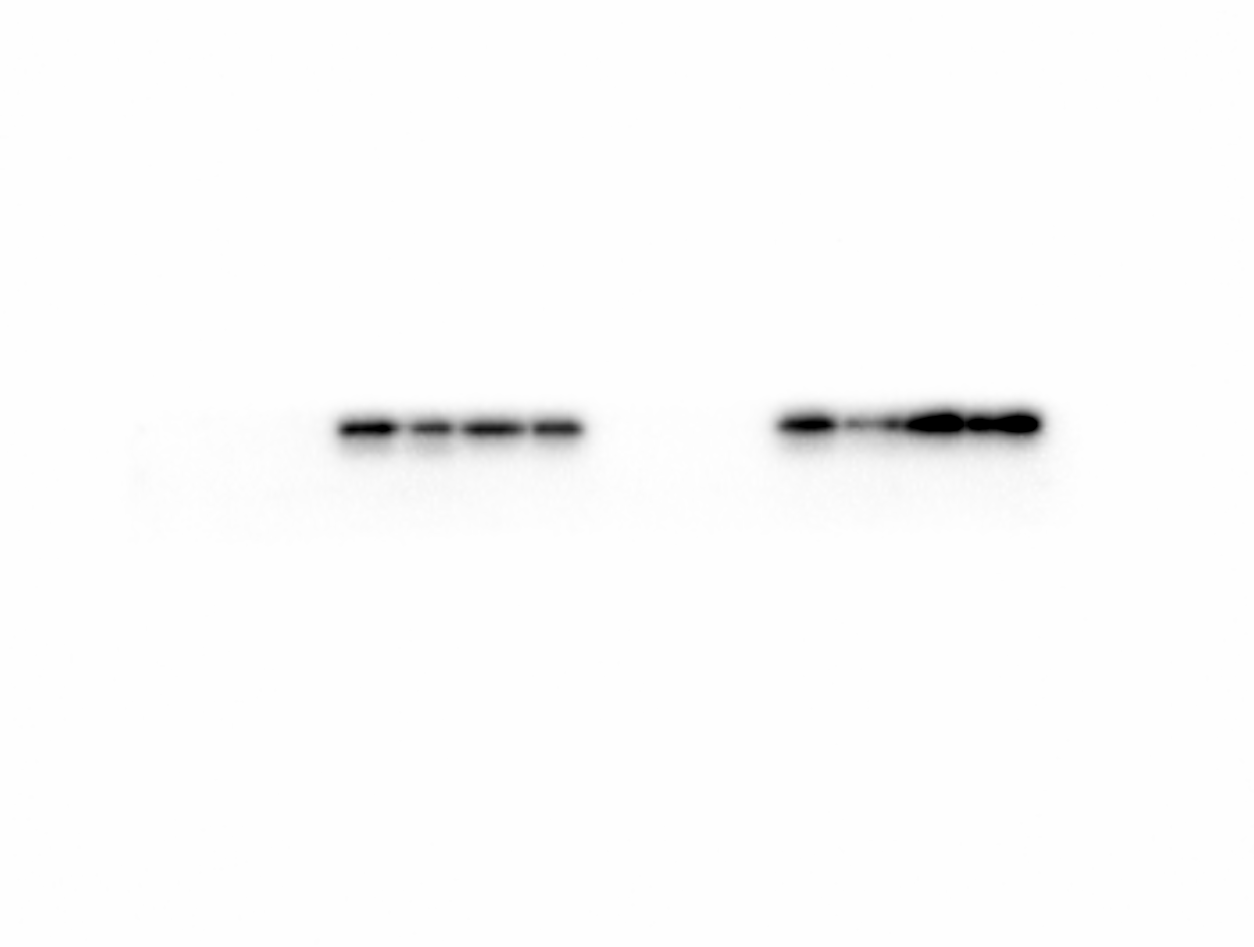

Supplement: Supplementary file 6 — Source data Fig. 2 [file 44318_2026_818_MOESM6_ESM.zip › Figure 2/Figure 2K/Figure 2K Replicate 2/3HA-FAM134B (lane 7-12).tif]

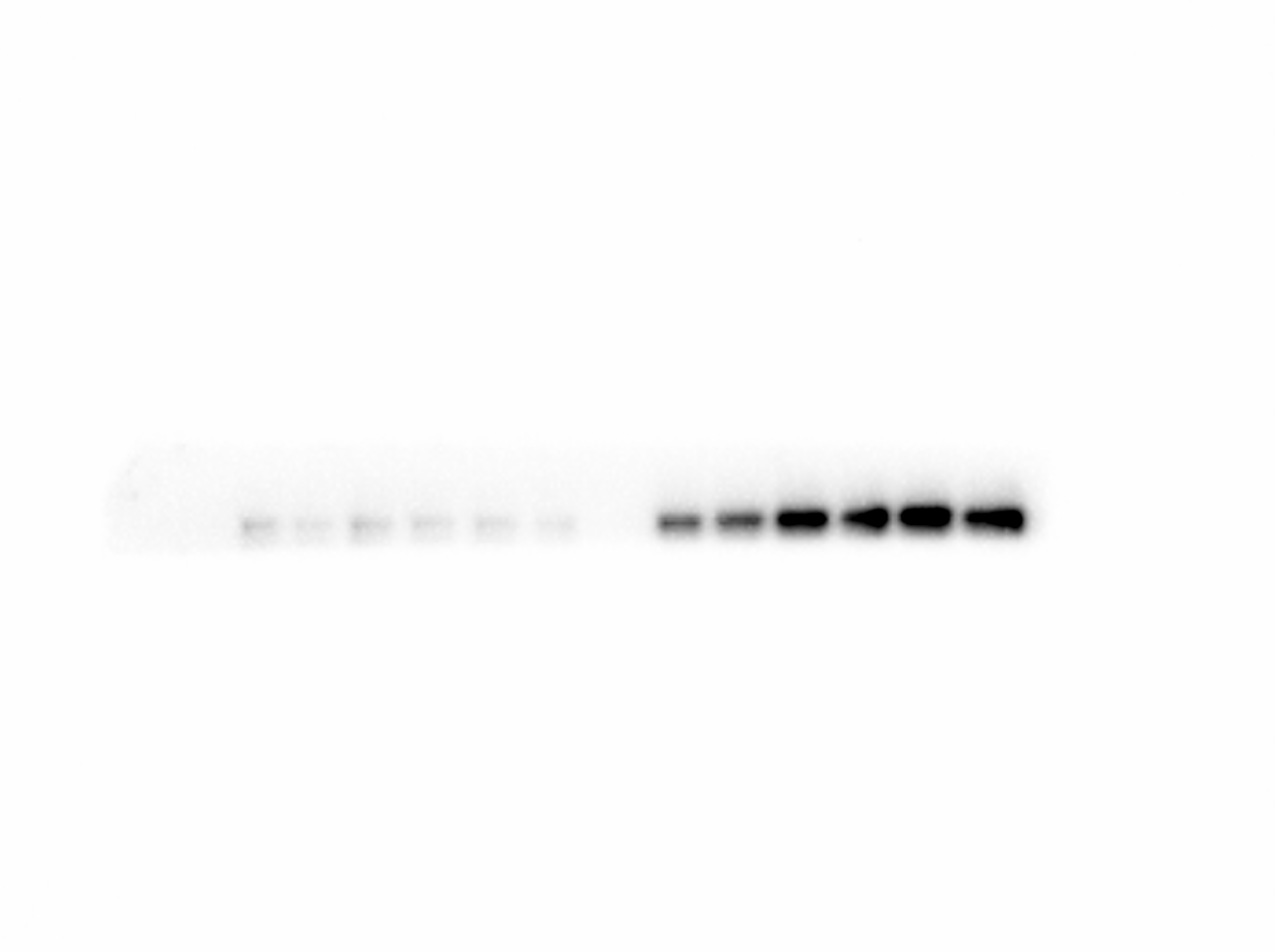

Supplement: Supplementary file 6 — Source data Fig. 2 [file 44318_2026_818_MOESM6_ESM.zip › Figure 2/Figure 2K/Figure 2K Replicate 2/APPmut-mCherry (lane 7-12).tif]

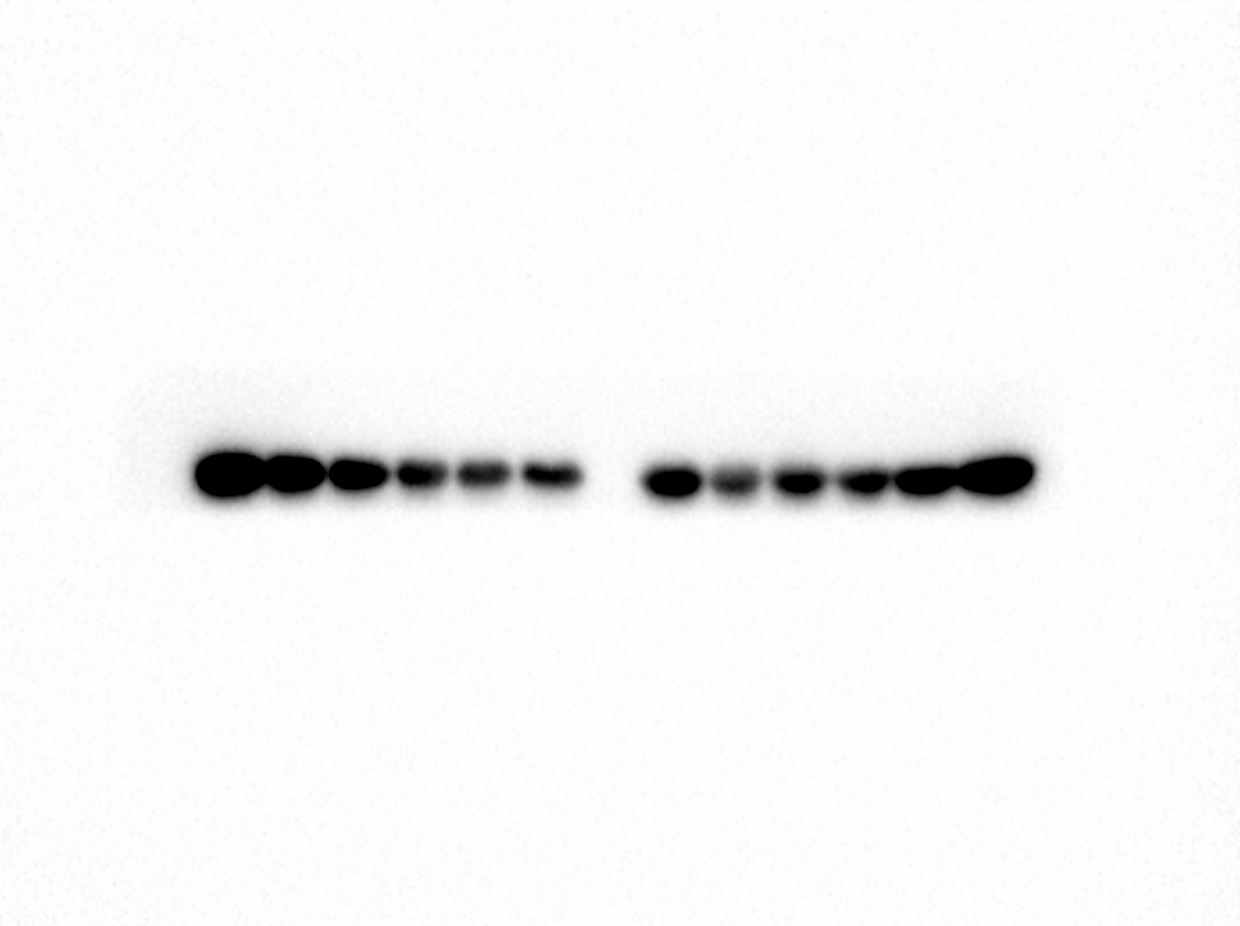

Supplement: Supplementary file 6 — Source data Fig. 2 [file 44318_2026_818_MOESM6_ESM.zip › Figure 2/Figure 2K/Figure 2K Replicate 2/GAPDH (lane 7-12).tif]

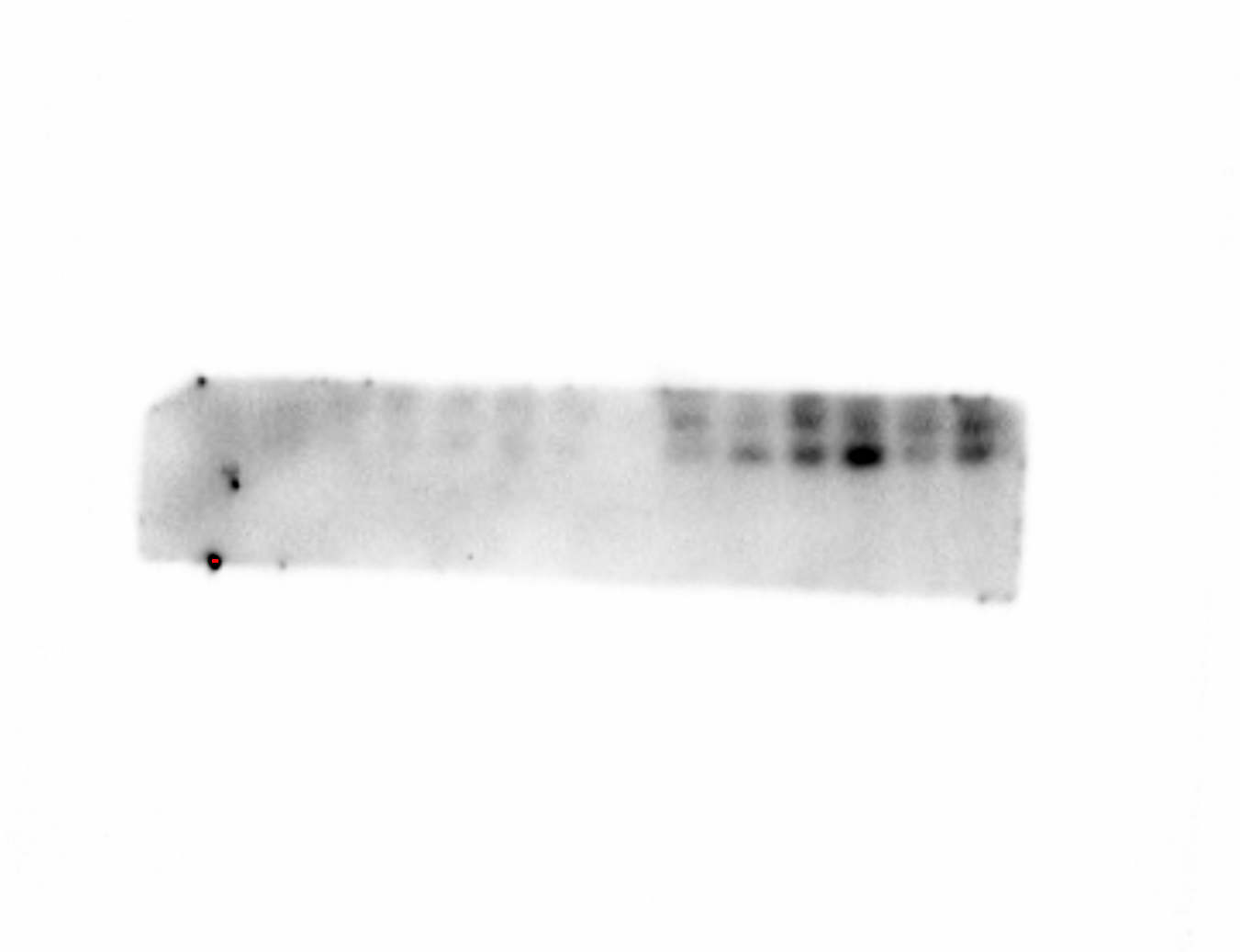

Supplement: Supplementary file 6 — Source data Fig. 2 [file 44318_2026_818_MOESM6_ESM.zip › Figure 2/Figure 2K/Figure 2K Replicate 2/mCherry (lane 7-12).tif]

Figure 2K

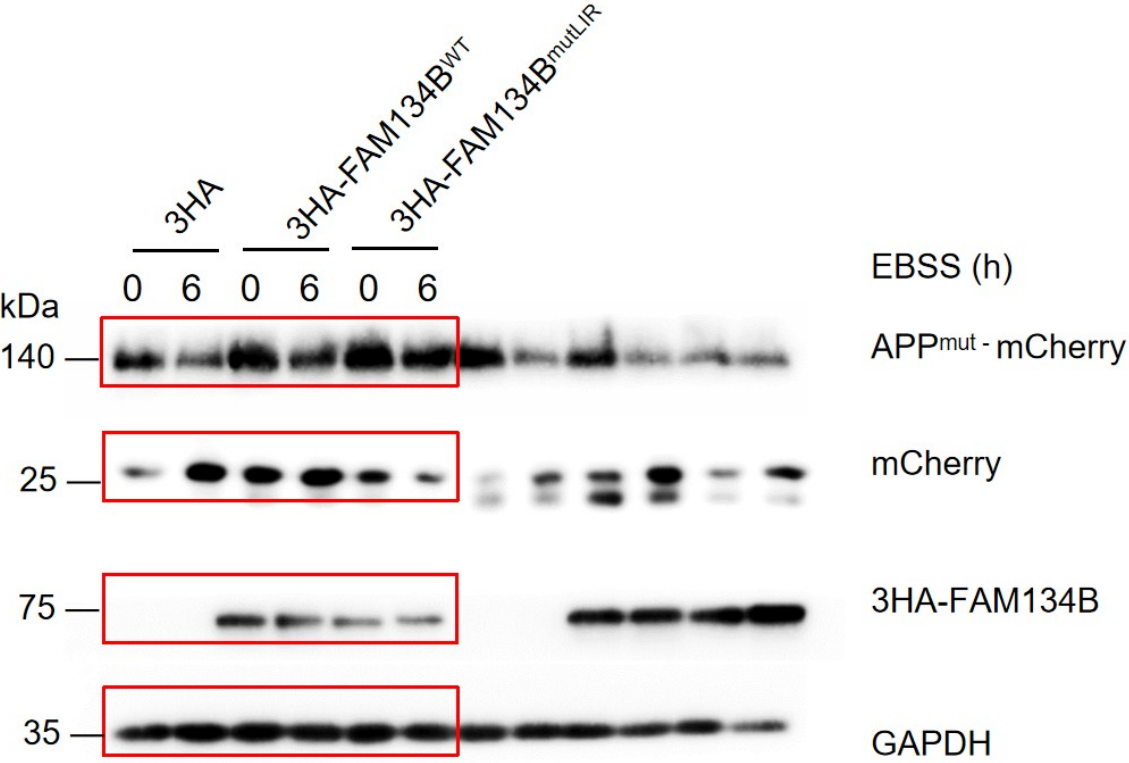

Replicate 1

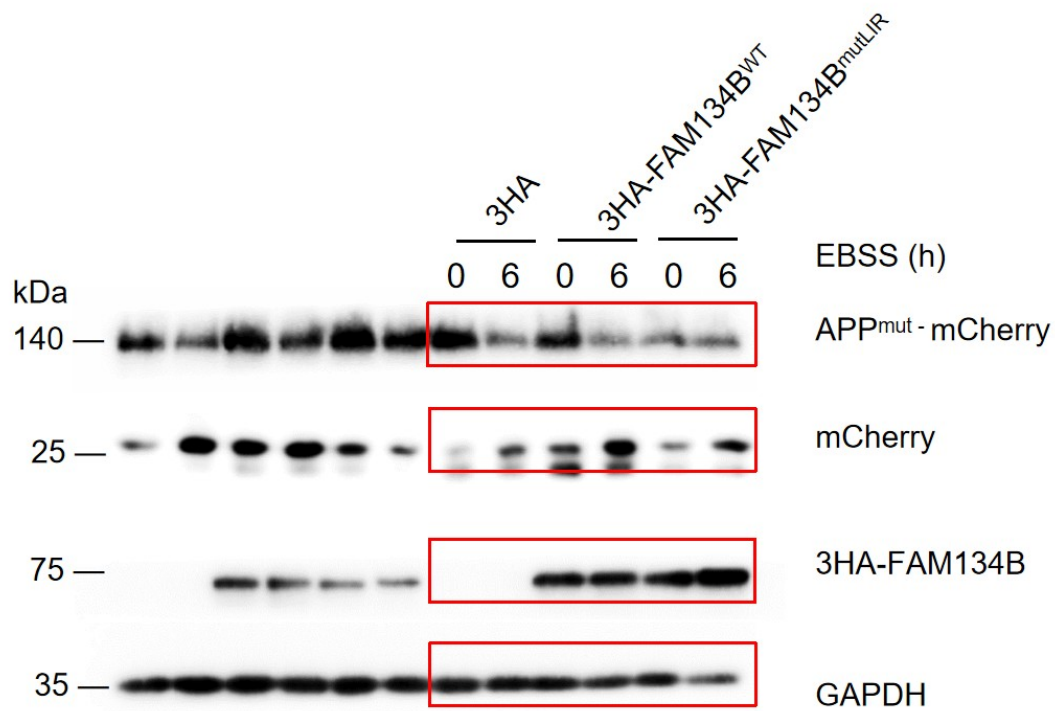

## Replicate 2

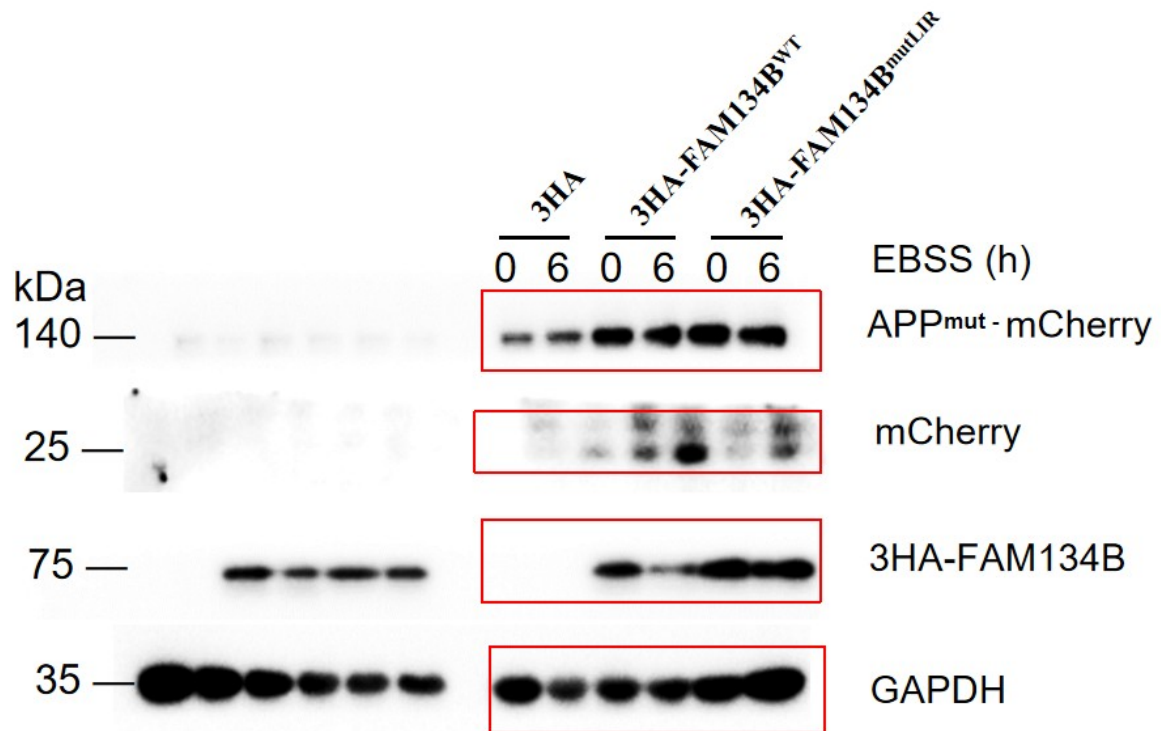

Supplement: Supplementary file 6 — Source data Fig. 2 [file 44318_2026_818_MOESM6_ESM.zip › Figure 2/Figure 2K/WB for Figure 2K.pdf]

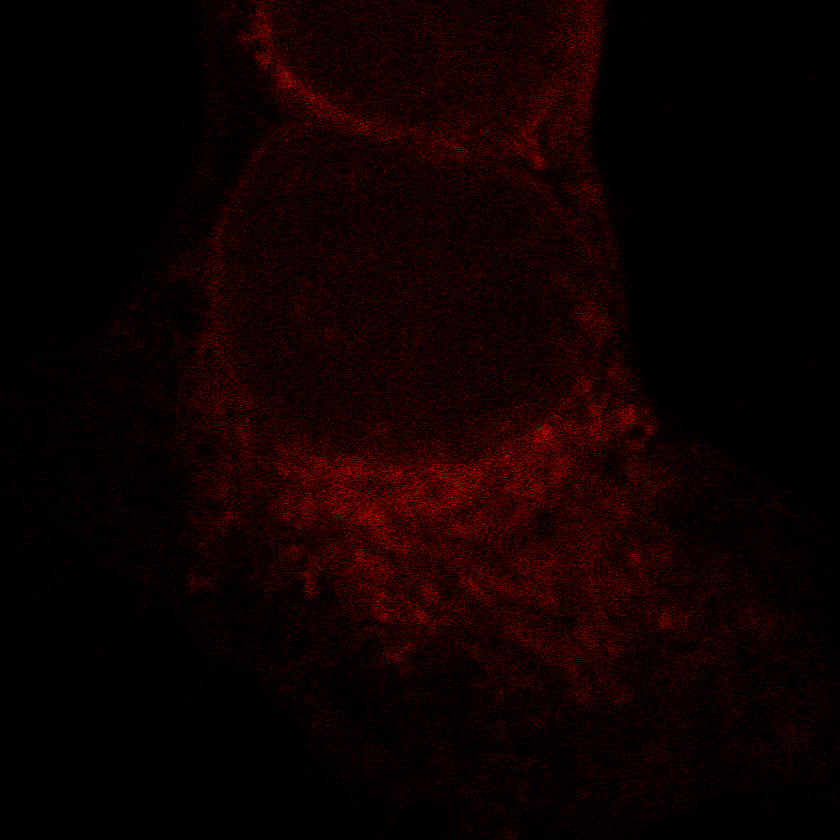

Supplement: Supplementary file 7 — Source data Fig. 3 [file 44318_2026_818_MOESM7_ESM.zip › Figure 3/Figure 3A/APPmut/BafA1/APPmut-mCherry.tif]

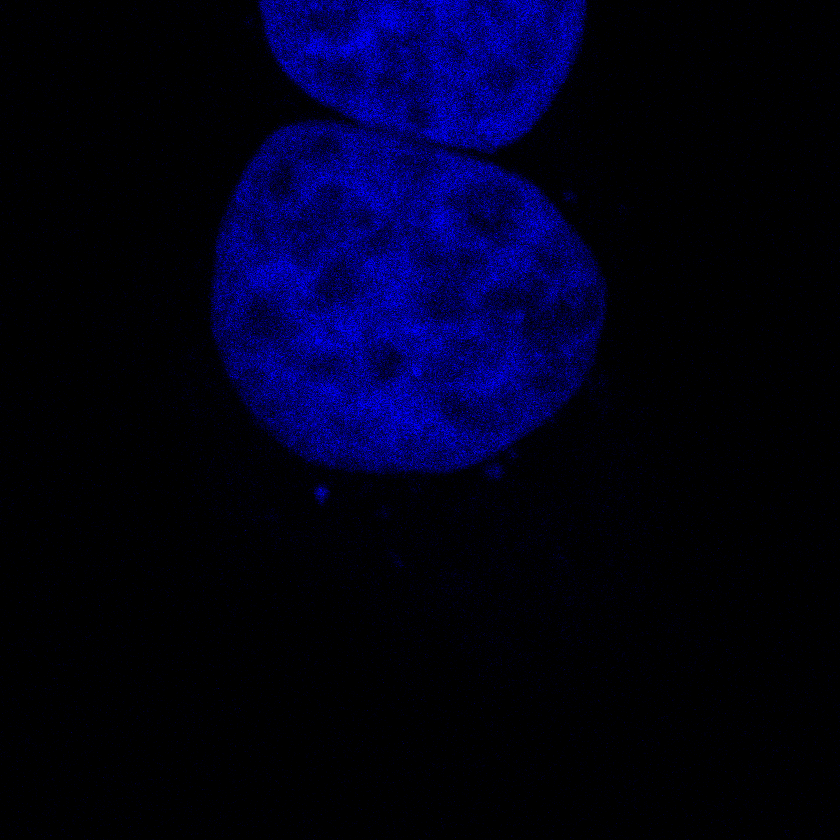

Supplement: Supplementary file 7 — Source data Fig. 3 [file 44318_2026_818_MOESM7_ESM.zip › Figure 3/Figure 3A/APPmut/BafA1/DAPI.tif]

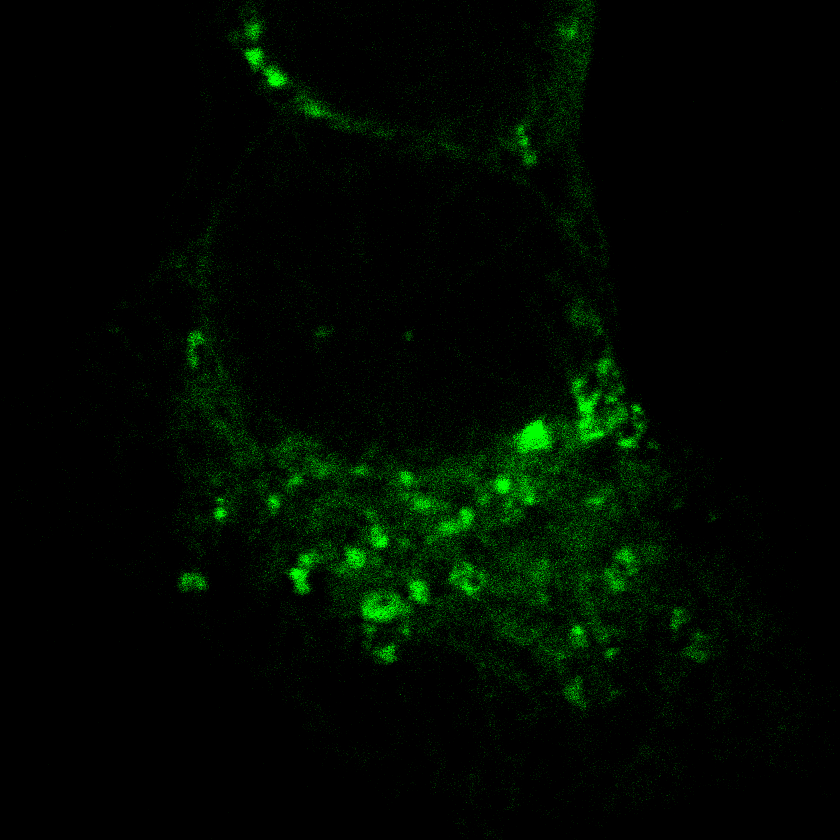

Supplement: Supplementary file 7 — Source data Fig. 3 [file 44318_2026_818_MOESM7_ESM.zip › Figure 3/Figure 3A/APPmut/BafA1/EGFP-FAM134B.tif]

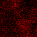

Supplement: Supplementary file 7 — Source data Fig. 3 [file 44318_2026_818_MOESM7_ESM.zip › Figure 3/Figure 3A/APPmut/BafA1/Inset-APPmut-mCherry.tif]

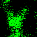

Supplement: Supplementary file 7 — Source data Fig. 3 [file 44318_2026_818_MOESM7_ESM.zip › Figure 3/Figure 3A/APPmut/BafA1/Inset-EGFP-FAM134B.tif]

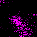

Supplement: Supplementary file 7 — Source data Fig. 3 [file 44318_2026_818_MOESM7_ESM.zip › Figure 3/Figure 3A/APPmut/BafA1/Inset-LC3B.tif]
