# Supplementary figures and images for: FAM134B-mediated ER-phagy degrades APP and suppresses Alzheimer’s disease pathology (part 2 of 3)
Source: EMBO J. 2026 May 26;45(13):4492–530. doi: 10.1038/s44318-026-00818-9 (PMC13324857; doi:10.1038/s44318-026-00818-9)

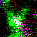

Supplement: Supplementary file 7 — Source data Fig. 3 [file 44318_2026_818_MOESM7_ESM.zip › Figure 3/Figure 3A/APPmut/BafA1/Inset-merge.tif]

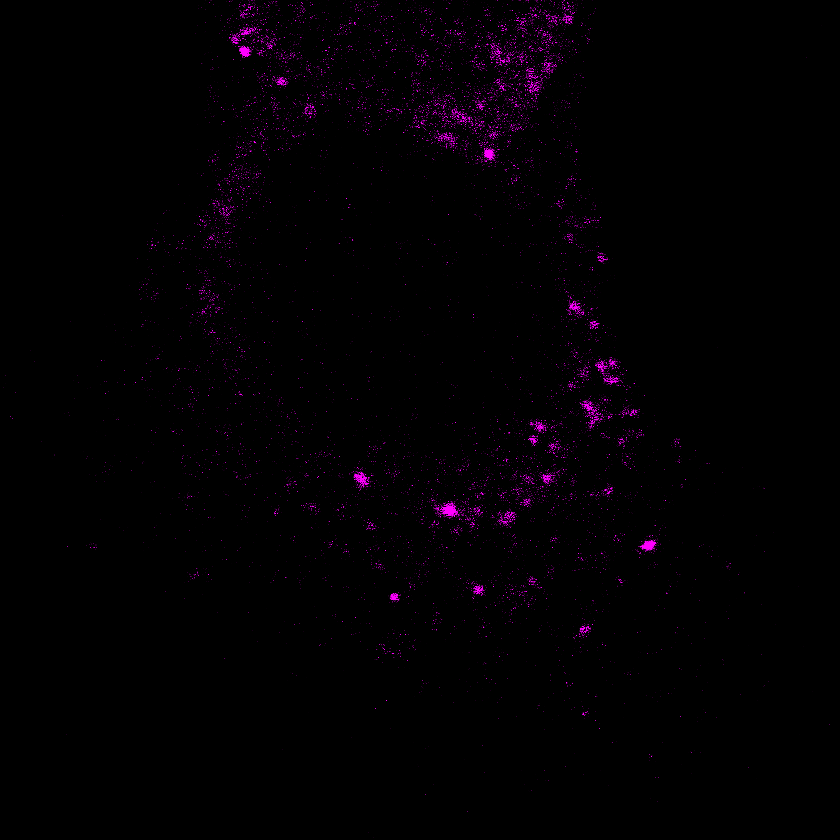

Supplement: Supplementary file 7 — Source data Fig. 3 [file 44318_2026_818_MOESM7_ESM.zip › Figure 3/Figure 3A/APPmut/BafA1/LC3B.tif]

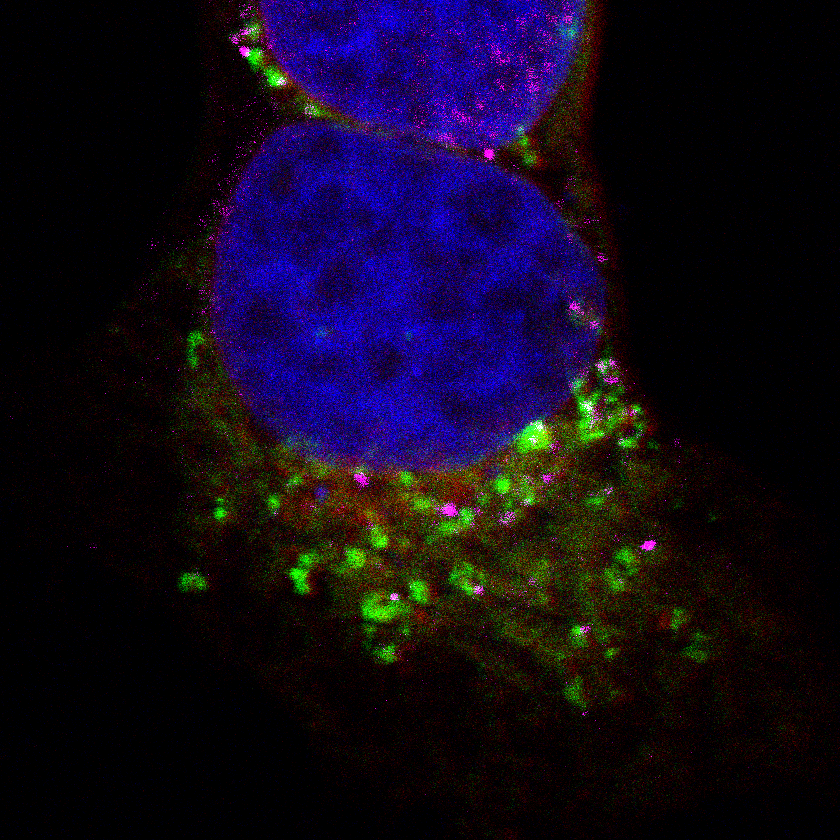

Supplement: Supplementary file 7 — Source data Fig. 3 [file 44318_2026_818_MOESM7_ESM.zip › Figure 3/Figure 3A/APPmut/BafA1/Merge.tif]

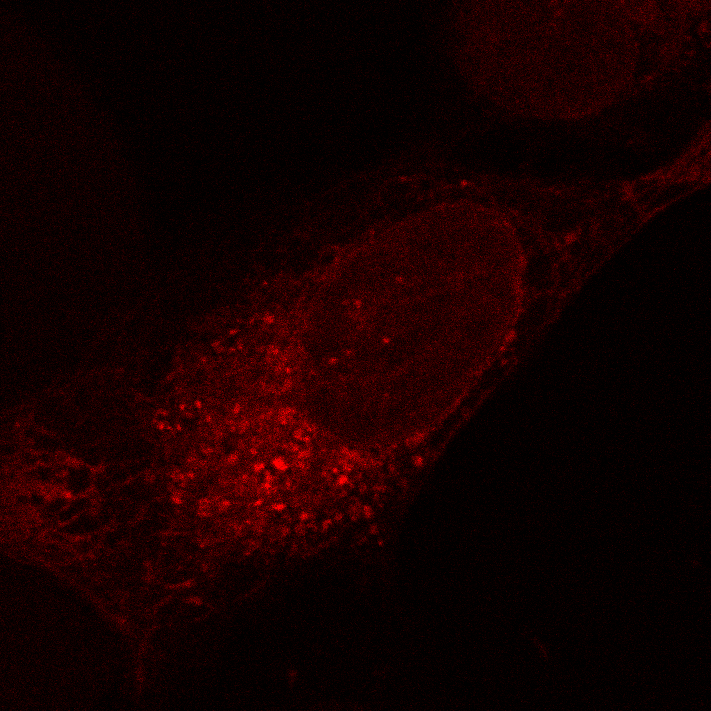

Supplement: Supplementary file 7 — Source data Fig. 3 [file 44318_2026_818_MOESM7_ESM.zip › Figure 3/Figure 3A/APPmut/EBSS+BafA1/APPmut-mCherry.tif]

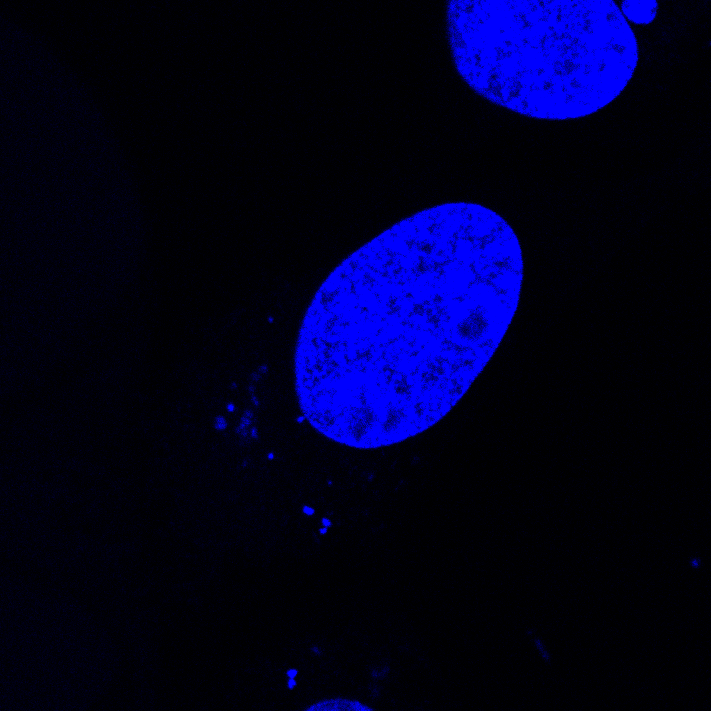

Supplement: Supplementary file 7 — Source data Fig. 3 [file 44318_2026_818_MOESM7_ESM.zip › Figure 3/Figure 3A/APPmut/EBSS+BafA1/DAPI.tif]

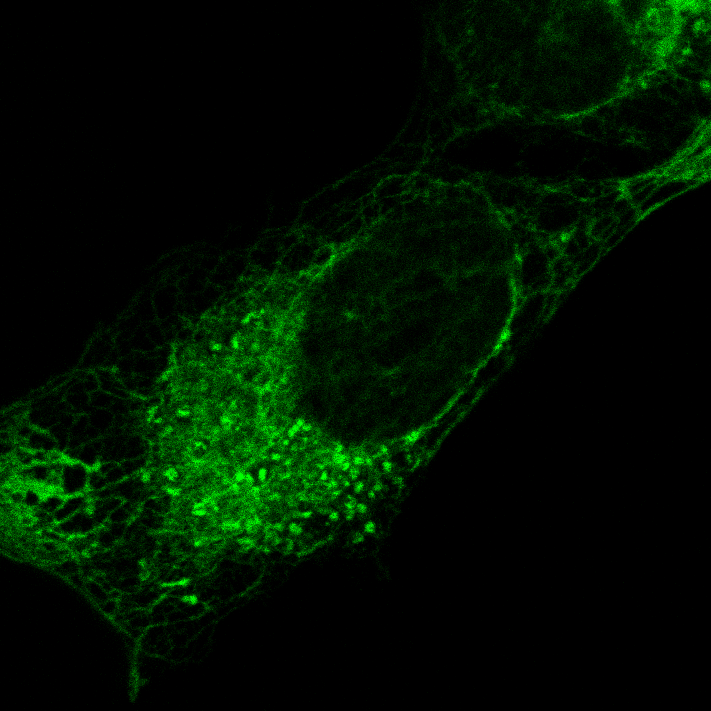

Supplement: Supplementary file 7 — Source data Fig. 3 [file 44318_2026_818_MOESM7_ESM.zip › Figure 3/Figure 3A/APPmut/EBSS+BafA1/EGFP-FAM134B.tif]

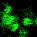

Supplement: Supplementary file 7 — Source data Fig. 3 [file 44318_2026_818_MOESM7_ESM.zip › Figure 3/Figure 3A/APPmut/EBSS+BafA1/Inset-EGFP-FAM134B.tif]

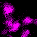

Supplement: Supplementary file 7 — Source data Fig. 3 [file 44318_2026_818_MOESM7_ESM.zip › Figure 3/Figure 3A/APPmut/EBSS+BafA1/Inset-LC3B.tif]

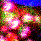

Supplement: Supplementary file 7 — Source data Fig. 3 [file 44318_2026_818_MOESM7_ESM.zip › Figure 3/Figure 3A/APPmut/EBSS+BafA1/Inset-merge.tif]

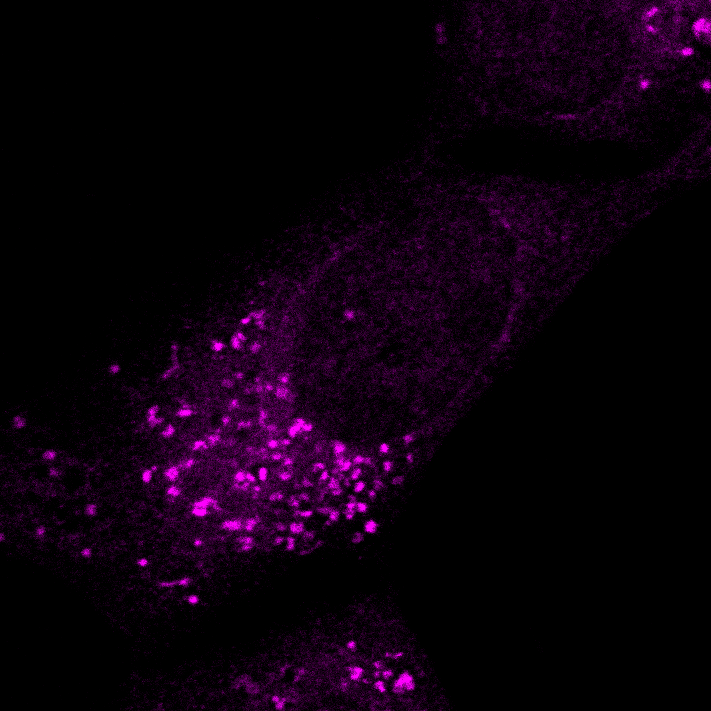

Supplement: Supplementary file 7 — Source data Fig. 3 [file 44318_2026_818_MOESM7_ESM.zip › Figure 3/Figure 3A/APPmut/EBSS+BafA1/LC3B.tif]

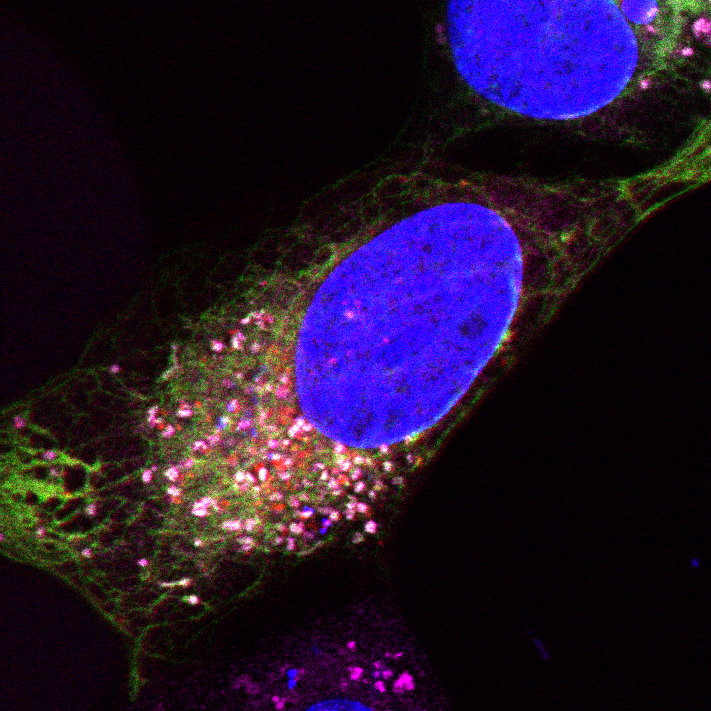

Supplement: Supplementary file 7 — Source data Fig. 3 [file 44318_2026_818_MOESM7_ESM.zip › Figure 3/Figure 3A/APPmut/EBSS+BafA1/Merge.tif]

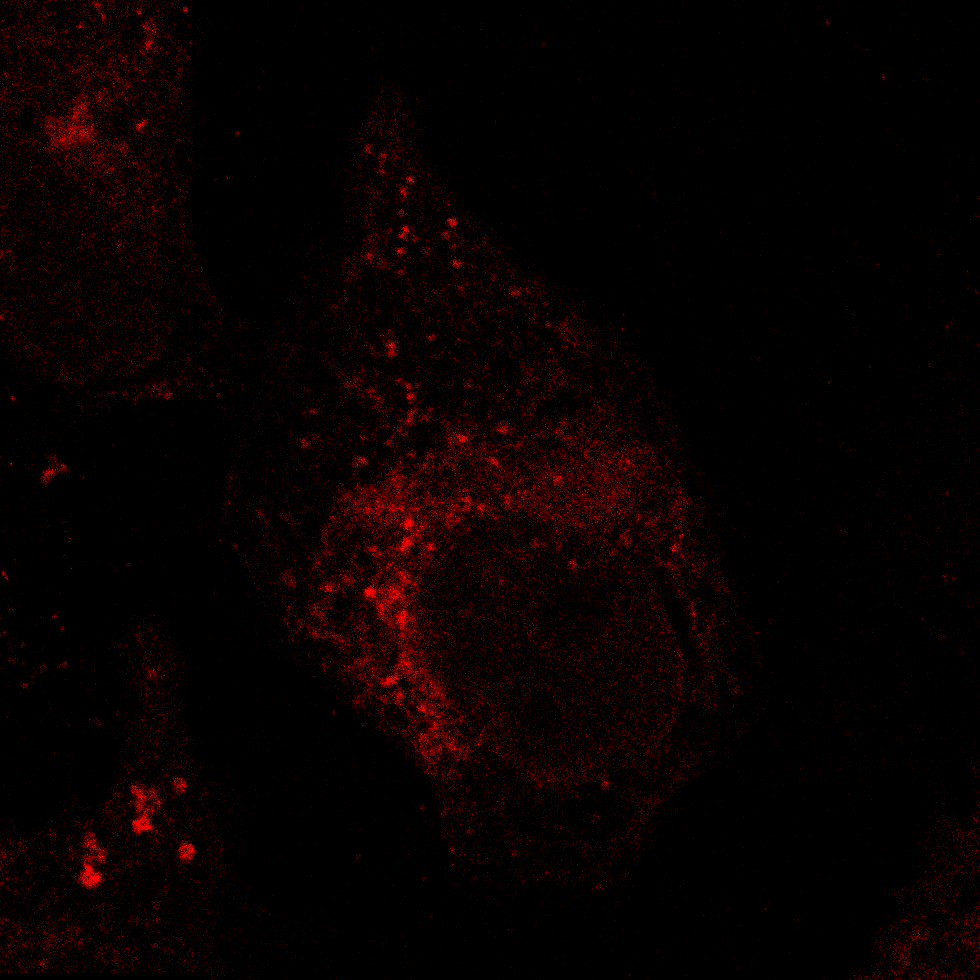

Supplement: Supplementary file 7 — Source data Fig. 3 [file 44318_2026_818_MOESM7_ESM.zip › Figure 3/Figure 3A/APPWT/BafA1/APPWT-mCherry.tif]

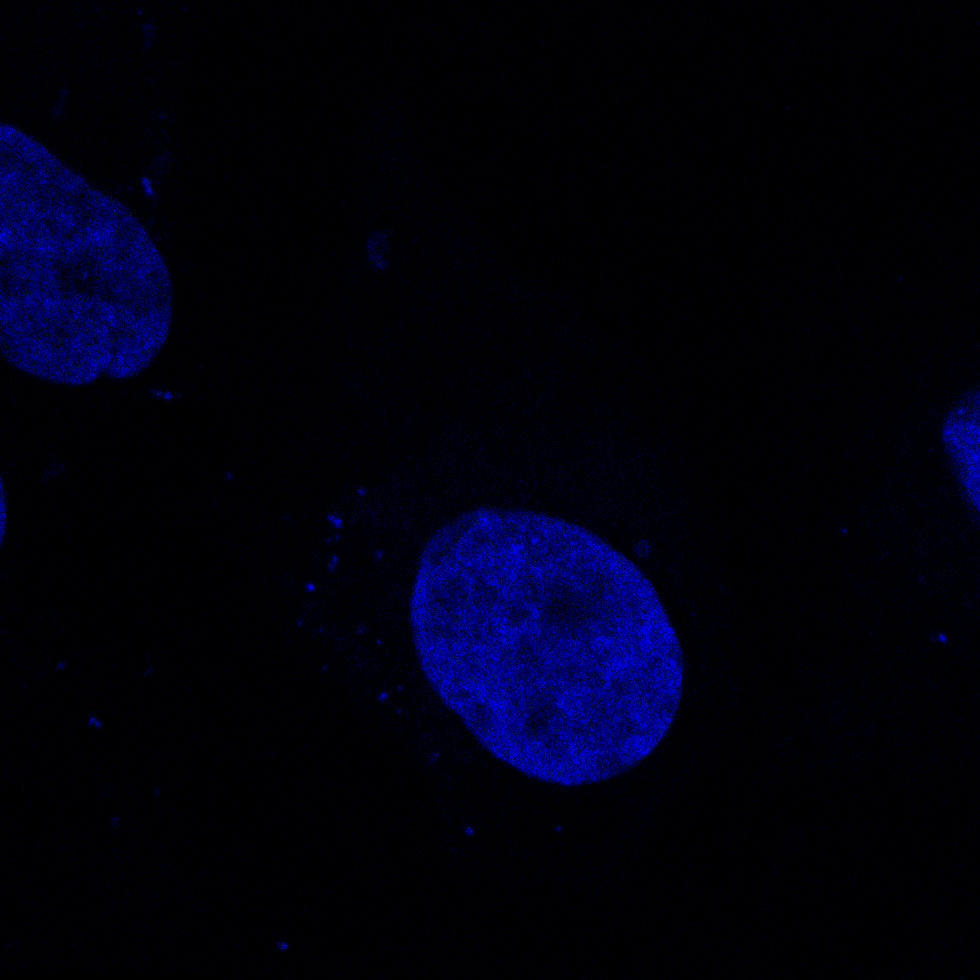

Supplement: Supplementary file 7 — Source data Fig. 3 [file 44318_2026_818_MOESM7_ESM.zip › Figure 3/Figure 3A/APPWT/BafA1/DAPI.tif]

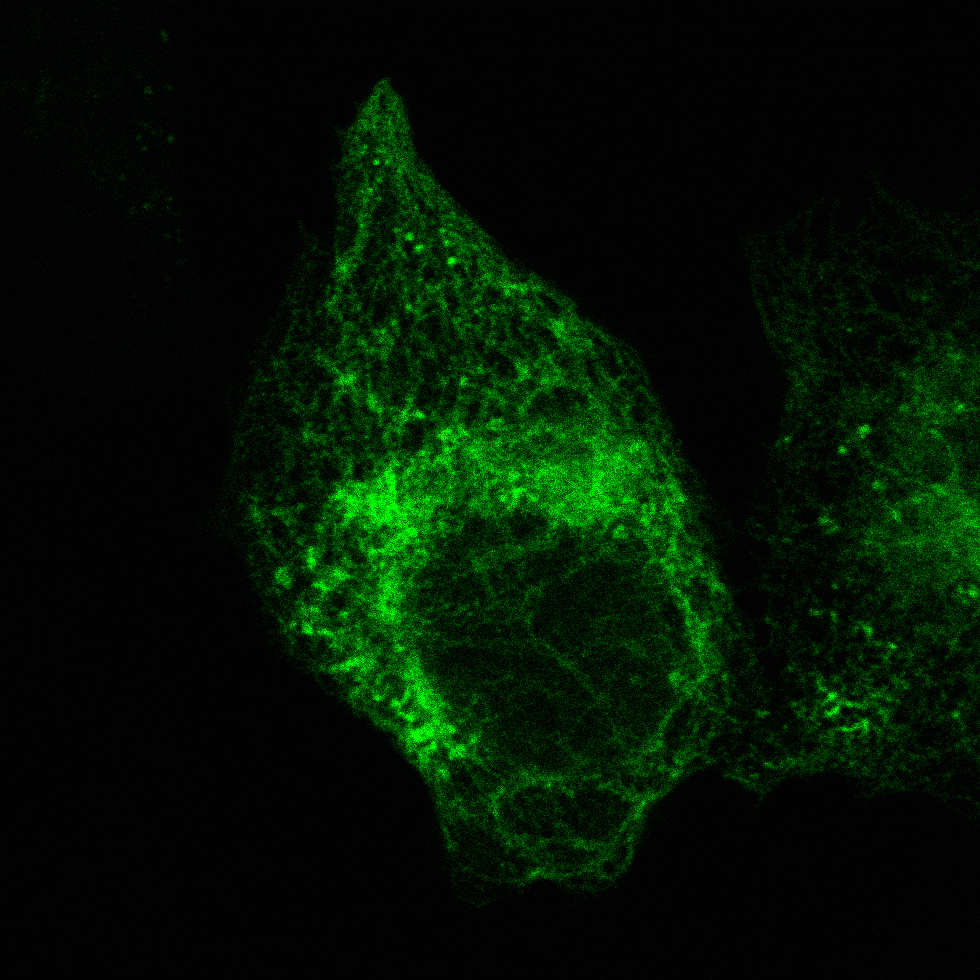

Supplement: Supplementary file 7 — Source data Fig. 3 [file 44318_2026_818_MOESM7_ESM.zip › Figure 3/Figure 3A/APPWT/BafA1/EGFP-FAM134B.tif]

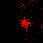

Supplement: Supplementary file 7 — Source data Fig. 3 [file 44318_2026_818_MOESM7_ESM.zip › Figure 3/Figure 3A/APPWT/BafA1/Inset-APPWT-mCherry.tif]

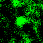

Supplement: Supplementary file 7 — Source data Fig. 3 [file 44318_2026_818_MOESM7_ESM.zip › Figure 3/Figure 3A/APPWT/BafA1/Inset-EGFP-FAM134B.tif]

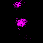

Supplement: Supplementary file 7 — Source data Fig. 3 [file 44318_2026_818_MOESM7_ESM.zip › Figure 3/Figure 3A/APPWT/BafA1/Inset-LC3B.tif]

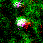

Supplement: Supplementary file 7 — Source data Fig. 3 [file 44318_2026_818_MOESM7_ESM.zip › Figure 3/Figure 3A/APPWT/BafA1/Inset-merge.tif]

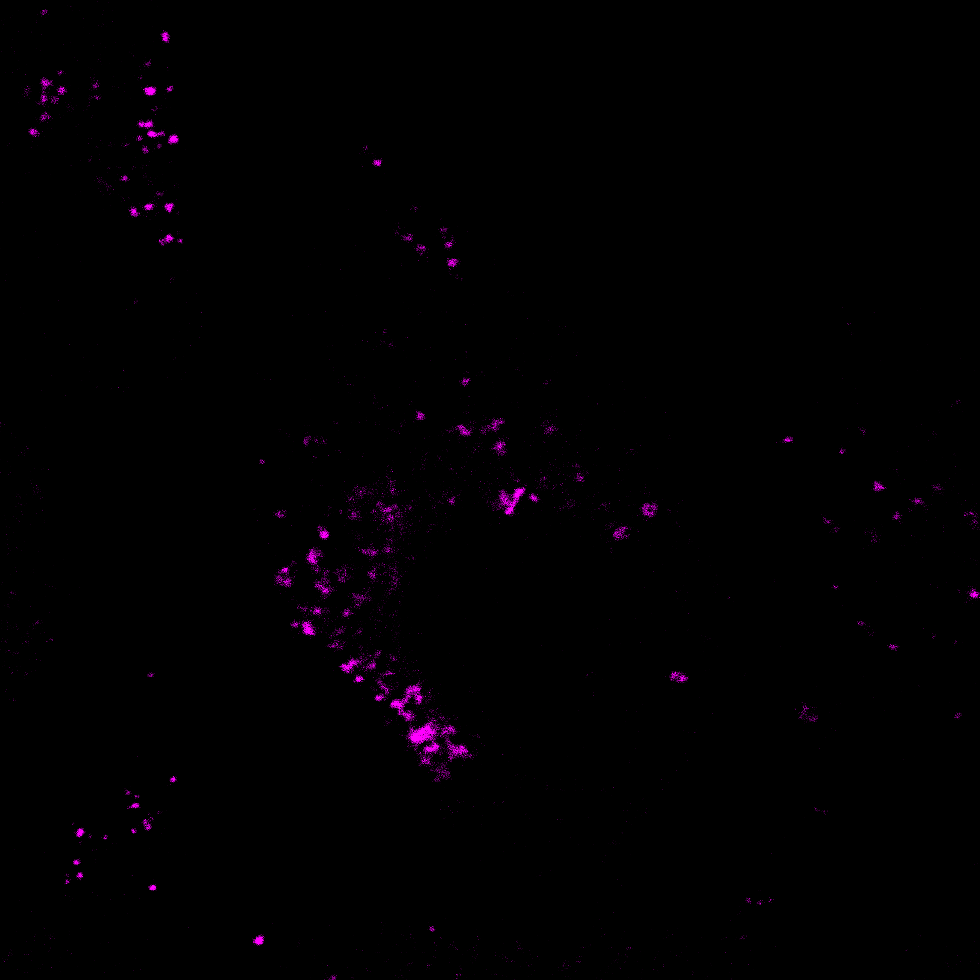

Supplement: Supplementary file 7 — Source data Fig. 3 [file 44318_2026_818_MOESM7_ESM.zip › Figure 3/Figure 3A/APPWT/BafA1/LC3B.tif]

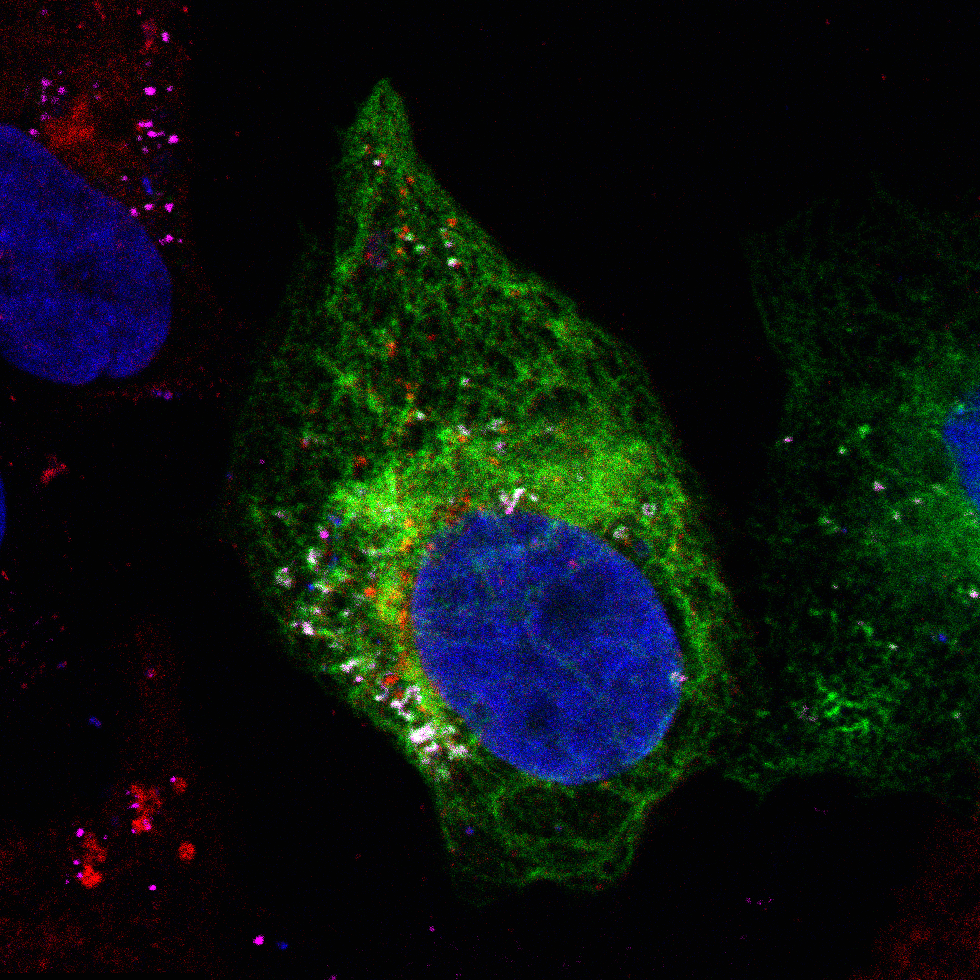

Supplement: Supplementary file 7 — Source data Fig. 3 [file 44318_2026_818_MOESM7_ESM.zip › Figure 3/Figure 3A/APPWT/BafA1/Merge.tif]

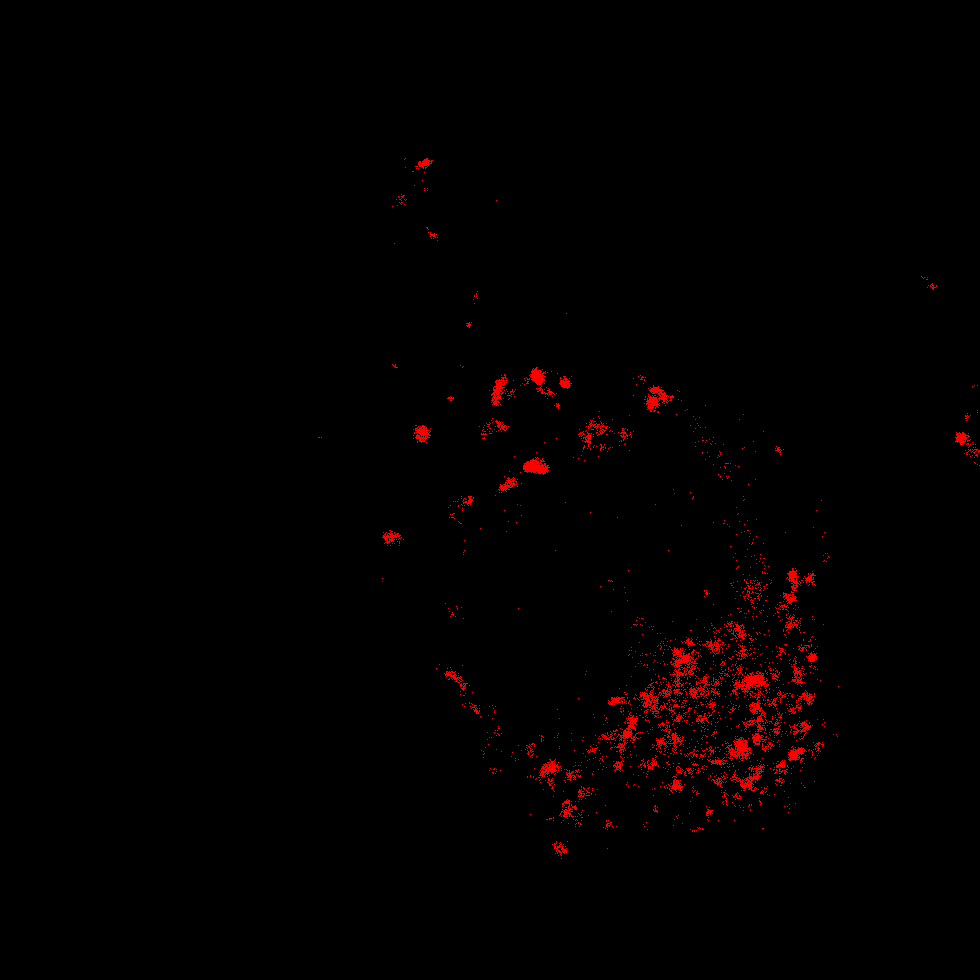

Supplement: Supplementary file 7 — Source data Fig. 3 [file 44318_2026_818_MOESM7_ESM.zip › Figure 3/Figure 3A/APPWT/EBSS+BafA1/APPWT-mCherry.tif]

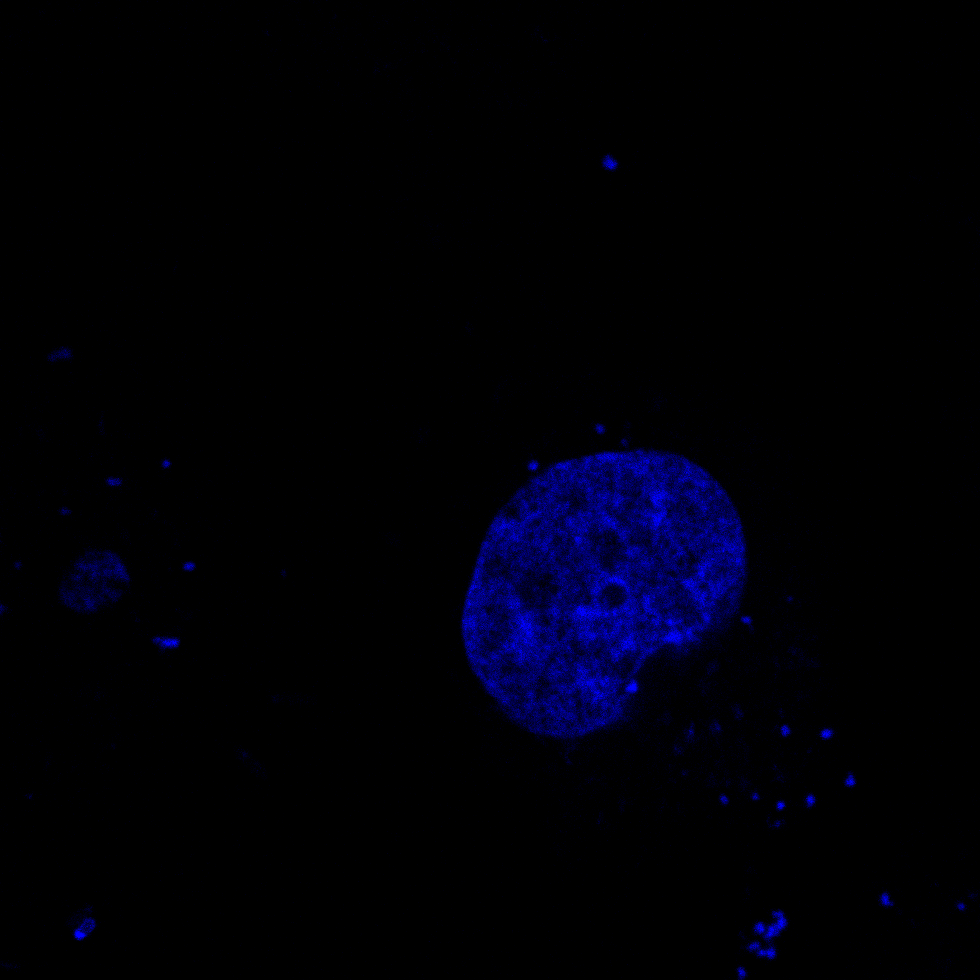

Supplement: Supplementary file 7 — Source data Fig. 3 [file 44318_2026_818_MOESM7_ESM.zip › Figure 3/Figure 3A/APPWT/EBSS+BafA1/DAPI.tif]

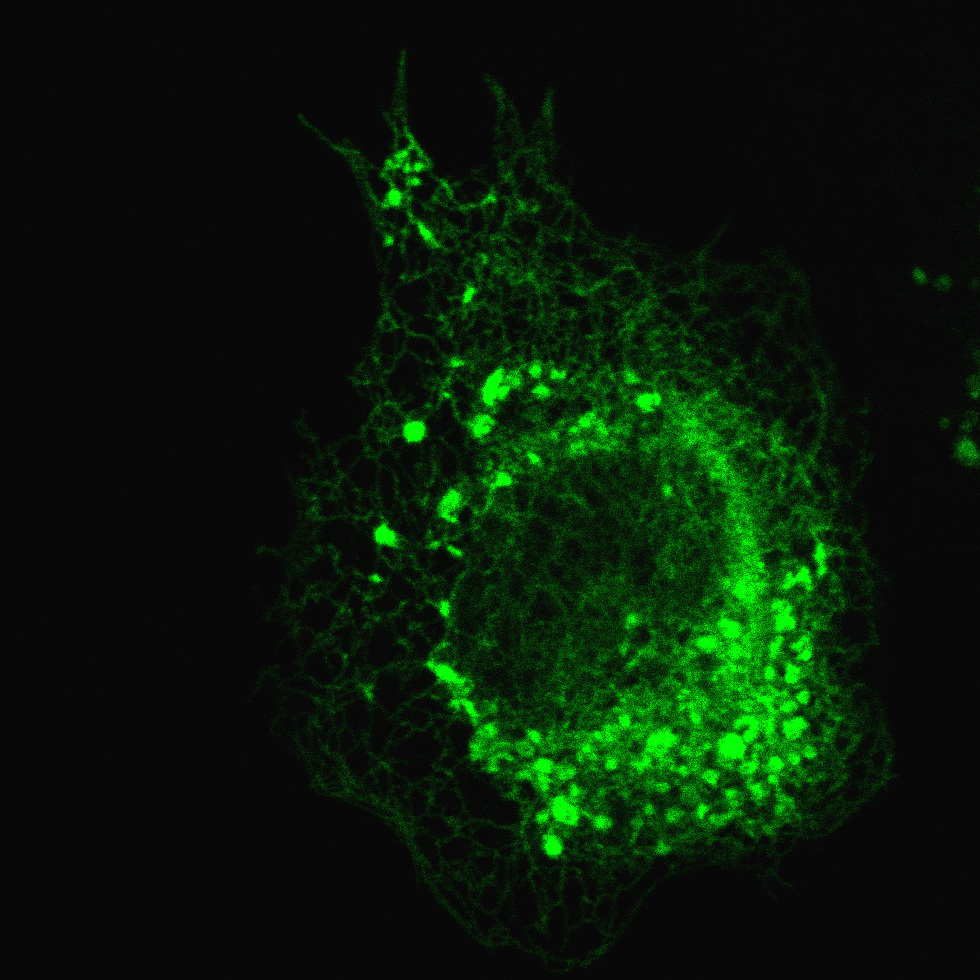

Supplement: Supplementary file 7 — Source data Fig. 3 [file 44318_2026_818_MOESM7_ESM.zip › Figure 3/Figure 3A/APPWT/EBSS+BafA1/EGFP-FAM134B.tif]

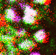

Supplement: Supplementary file 7 — Source data Fig. 3 [file 44318_2026_818_MOESM7_ESM.zip › Figure 3/Figure 3A/APPWT/EBSS+BafA1/Inset-merge.tif]

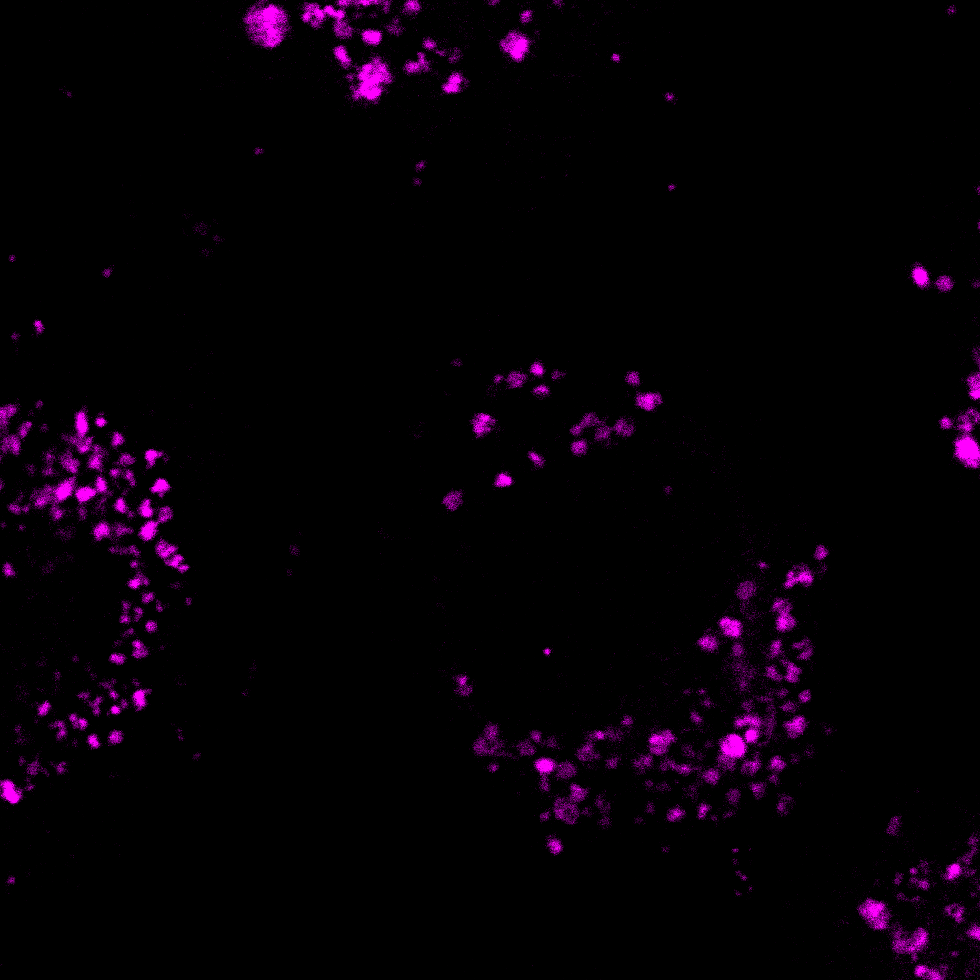

Supplement: Supplementary file 7 — Source data Fig. 3 [file 44318_2026_818_MOESM7_ESM.zip › Figure 3/Figure 3A/APPWT/EBSS+BafA1/LAMP1.tif]

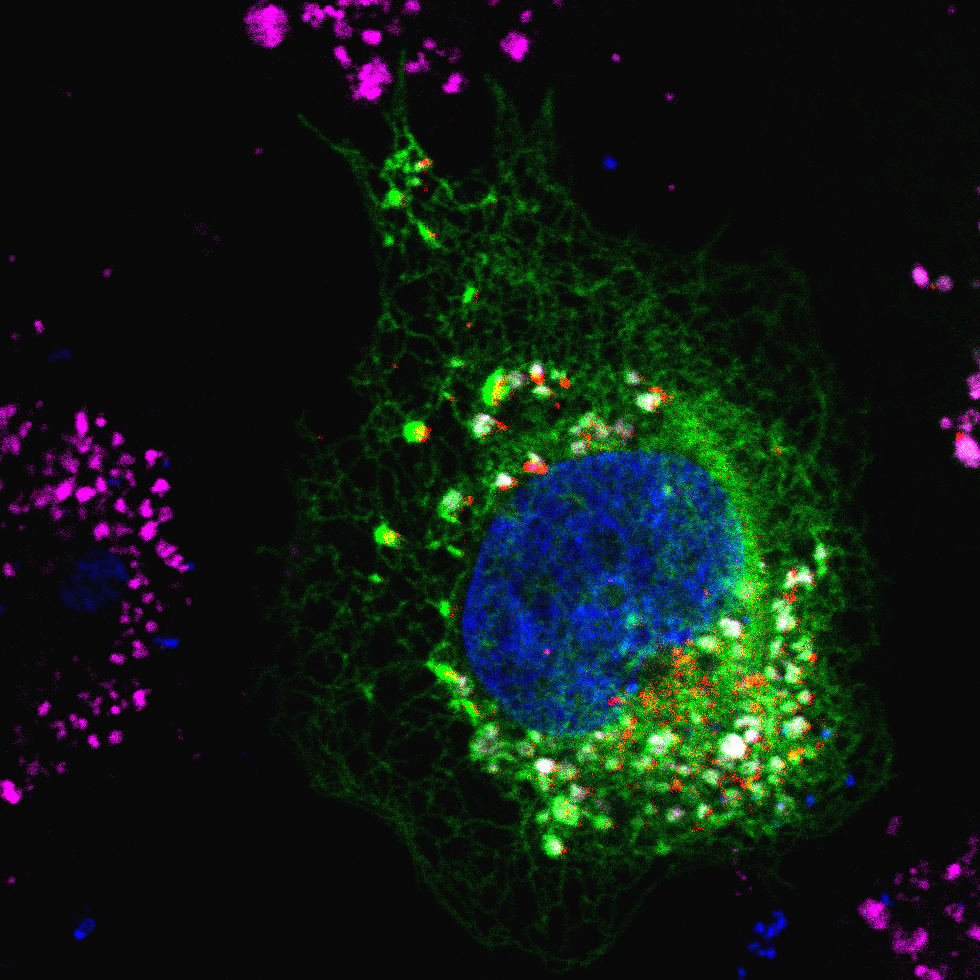

Supplement: Supplementary file 7 — Source data Fig. 3 [file 44318_2026_818_MOESM7_ESM.zip › Figure 3/Figure 3A/APPWT/EBSS+BafA1/Merge.tif]

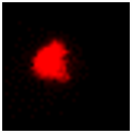

Supplement: Supplementary file 7 — Source data Fig. 3 [file 44318_2026_818_MOESM7_ESM.zip › Figure 3/Figure 3C/3C Bottom/APP-mCherry.tif]

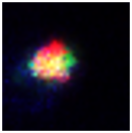

Supplement: Supplementary file 7 — Source data Fig. 3 [file 44318_2026_818_MOESM7_ESM.zip › Figure 3/Figure 3C/3C Bottom/Confocal Merge.tif]

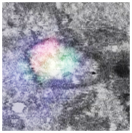

Supplement: Supplementary file 7 — Source data Fig. 3 [file 44318_2026_818_MOESM7_ESM.zip › Figure 3/Figure 3C/3C Bottom/Confocal X EM.tif]

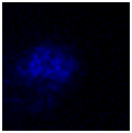

Supplement: Supplementary file 7 — Source data Fig. 3 [file 44318_2026_818_MOESM7_ESM.zip › Figure 3/Figure 3C/3C Bottom/DAPI.tif]

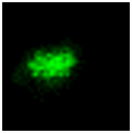

Supplement: Supplementary file 7 — Source data Fig. 3 [file 44318_2026_818_MOESM7_ESM.zip › Figure 3/Figure 3C/3C Bottom/EGFP-FAM134B.tif]

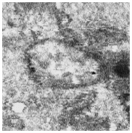

Supplement: Supplementary file 7 — Source data Fig. 3 [file 44318_2026_818_MOESM7_ESM.zip › Figure 3/Figure 3C/3C Bottom/EM.tif]

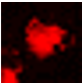

Supplement: Supplementary file 7 — Source data Fig. 3 [file 44318_2026_818_MOESM7_ESM.zip › Figure 3/Figure 3C/3C Top/APP.tif]

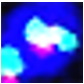

Supplement: Supplementary file 7 — Source data Fig. 3 [file 44318_2026_818_MOESM7_ESM.zip › Figure 3/Figure 3C/3C Top/Confocal Merge.png]

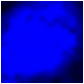

Supplement: Supplementary file 7 — Source data Fig. 3 [file 44318_2026_818_MOESM7_ESM.zip › Figure 3/Figure 3C/3C Top/DAPI.tif]

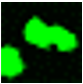

Supplement: Supplementary file 7 — Source data Fig. 3 [file 44318_2026_818_MOESM7_ESM.zip › Figure 3/Figure 3C/3C Top/EGFP-FAM134B.tif]

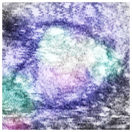

Supplement: Supplementary file 7 — Source data Fig. 3 [file 44318_2026_818_MOESM7_ESM.zip › Figure 3/Figure 3C/3C Top/EM X Confocal.tif]

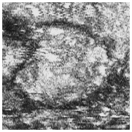

Supplement: Supplementary file 7 — Source data Fig. 3 [file 44318_2026_818_MOESM7_ESM.zip › Figure 3/Figure 3C/3C Top/EM.tif]

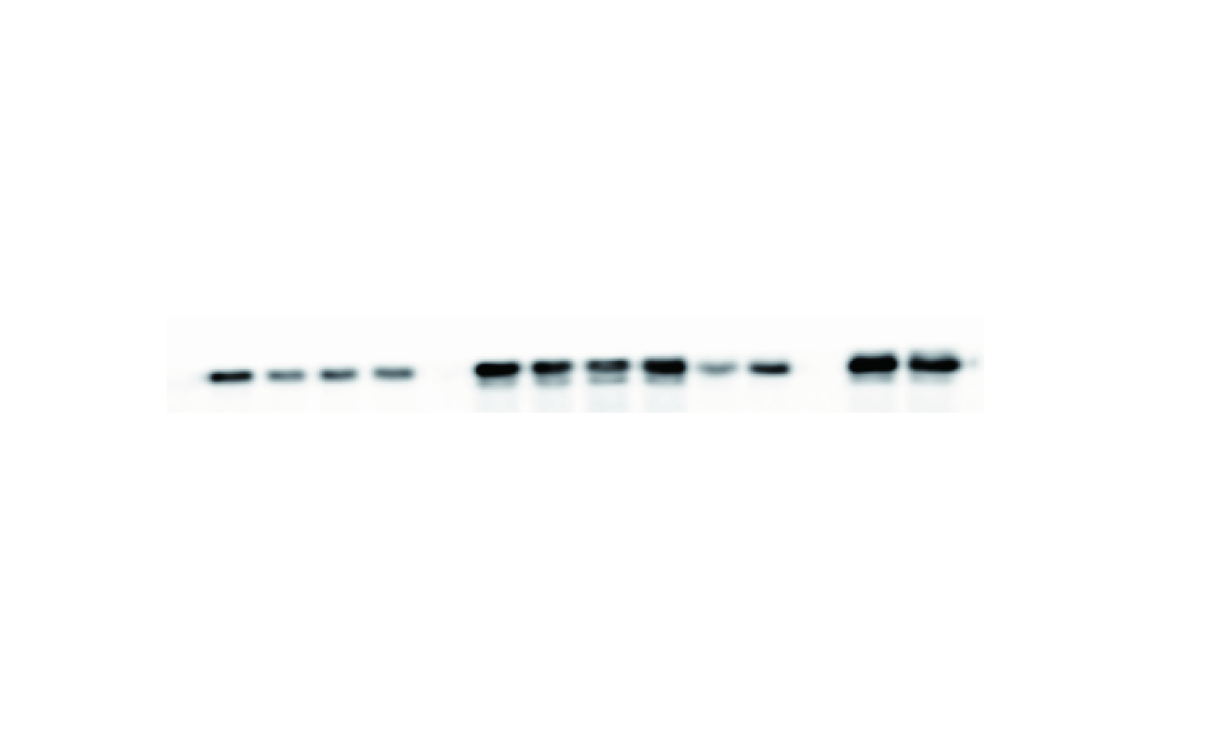

Supplement: Supplementary file 7 — Source data Fig. 3 [file 44318_2026_818_MOESM7_ESM.zip › Figure 3/Figure 3D/3HA-FAM134B.tif]

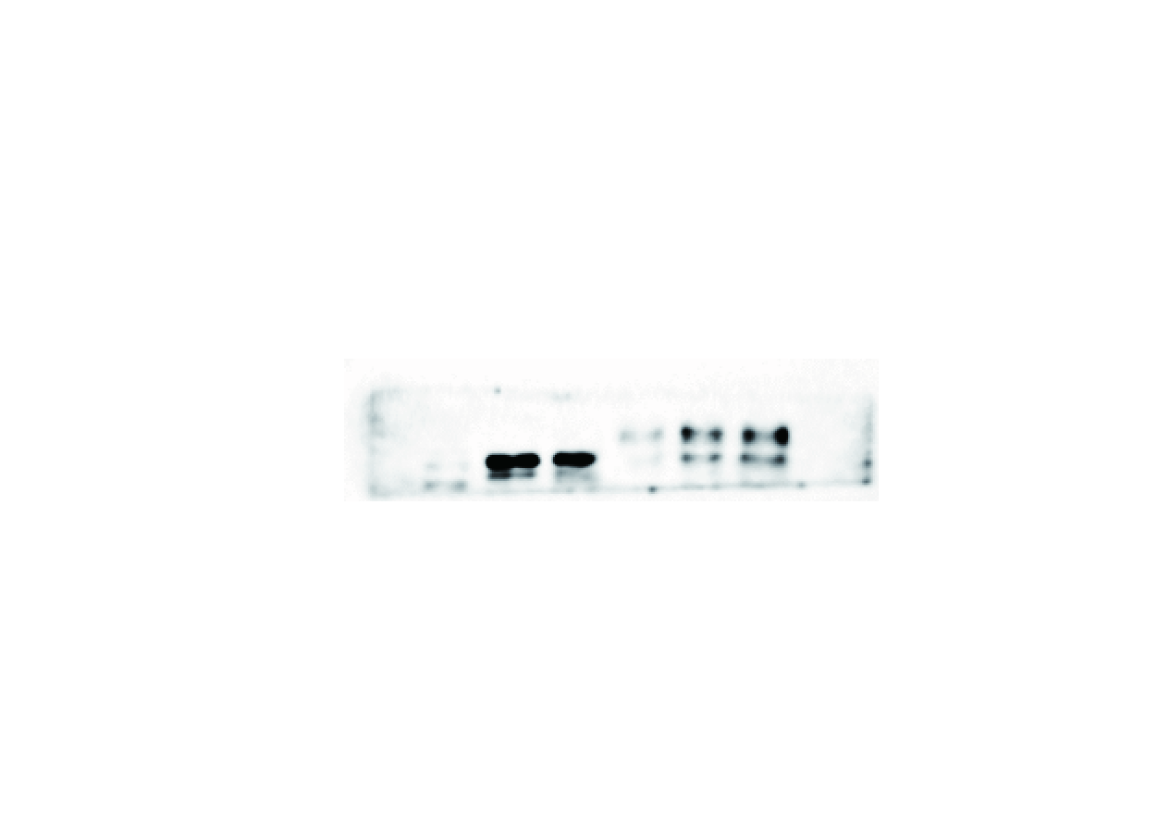

Supplement: Supplementary file 7 — Source data Fig. 3 [file 44318_2026_818_MOESM7_ESM.zip › Figure 3/Figure 3D/APP.tif]

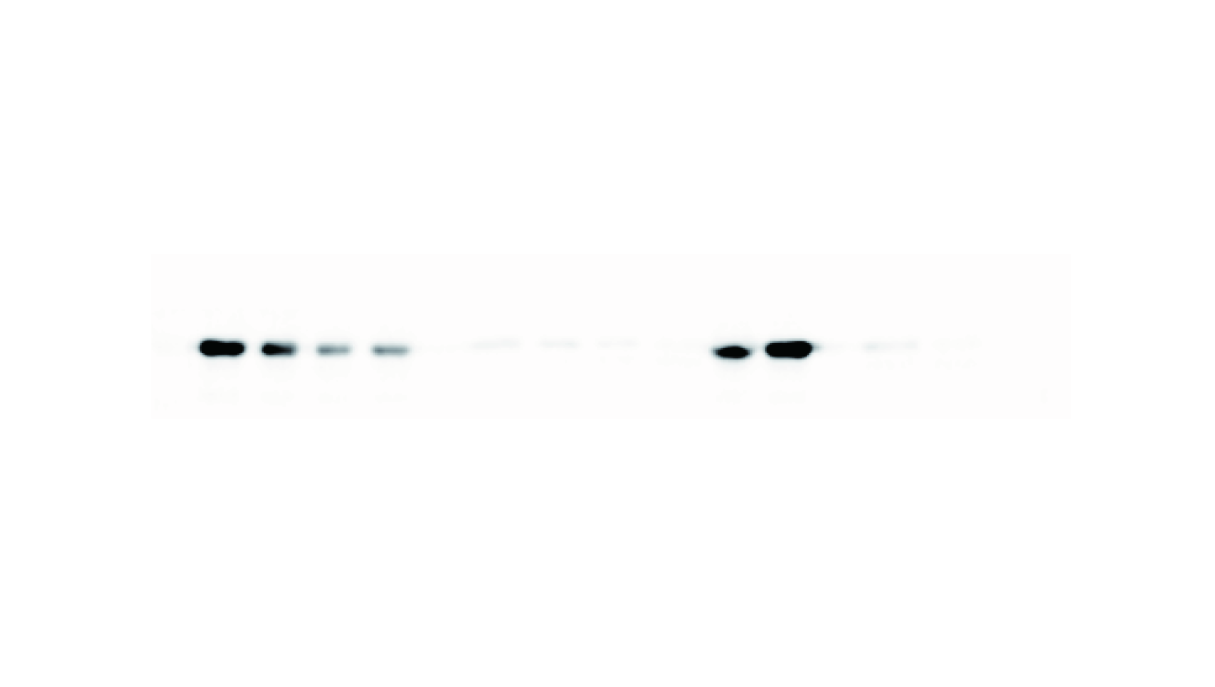

Supplement: Supplementary file 7 — Source data Fig. 3 [file 44318_2026_818_MOESM7_ESM.zip › Figure 3/Figure 3D/GAPDH.tif]

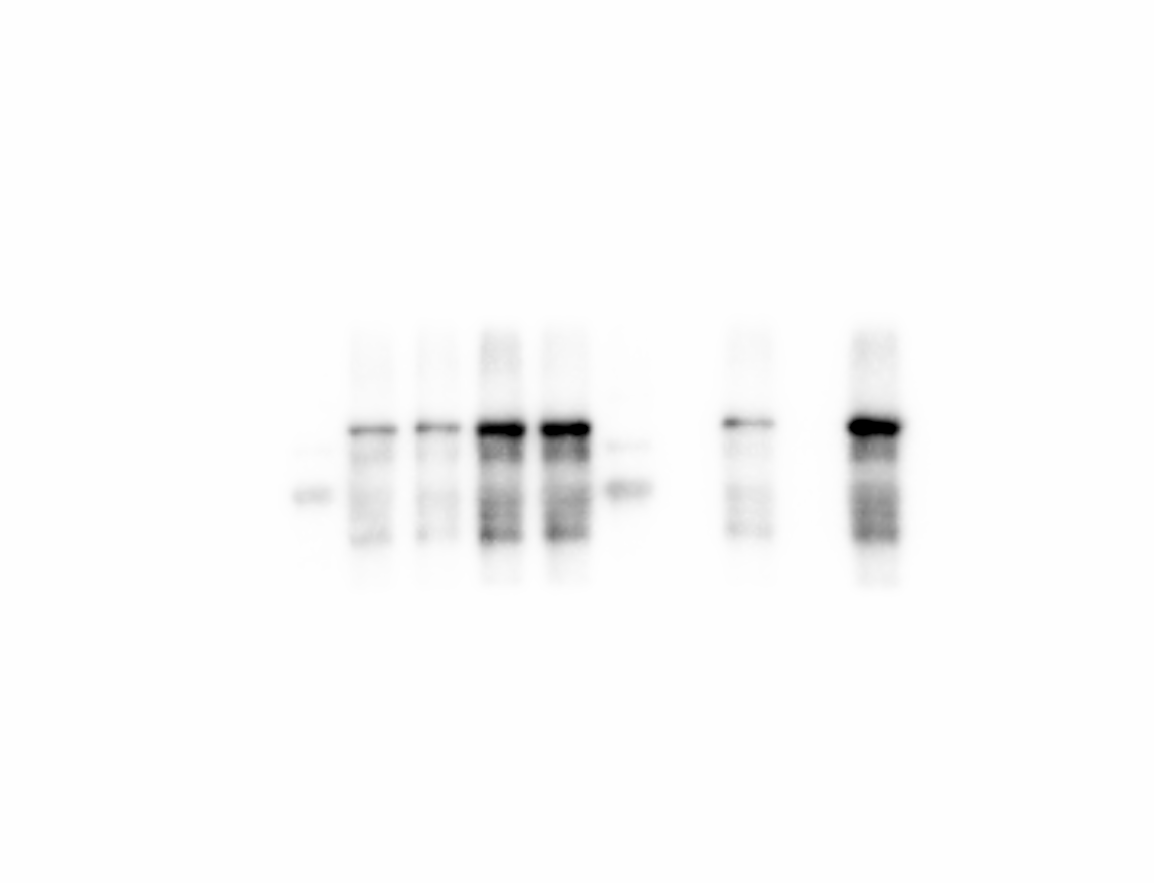

Supplement: Supplementary file 7 — Source data Fig. 3 [file 44318_2026_818_MOESM7_ESM.zip › Figure 3/Figure 3E/6His-APP.tif]

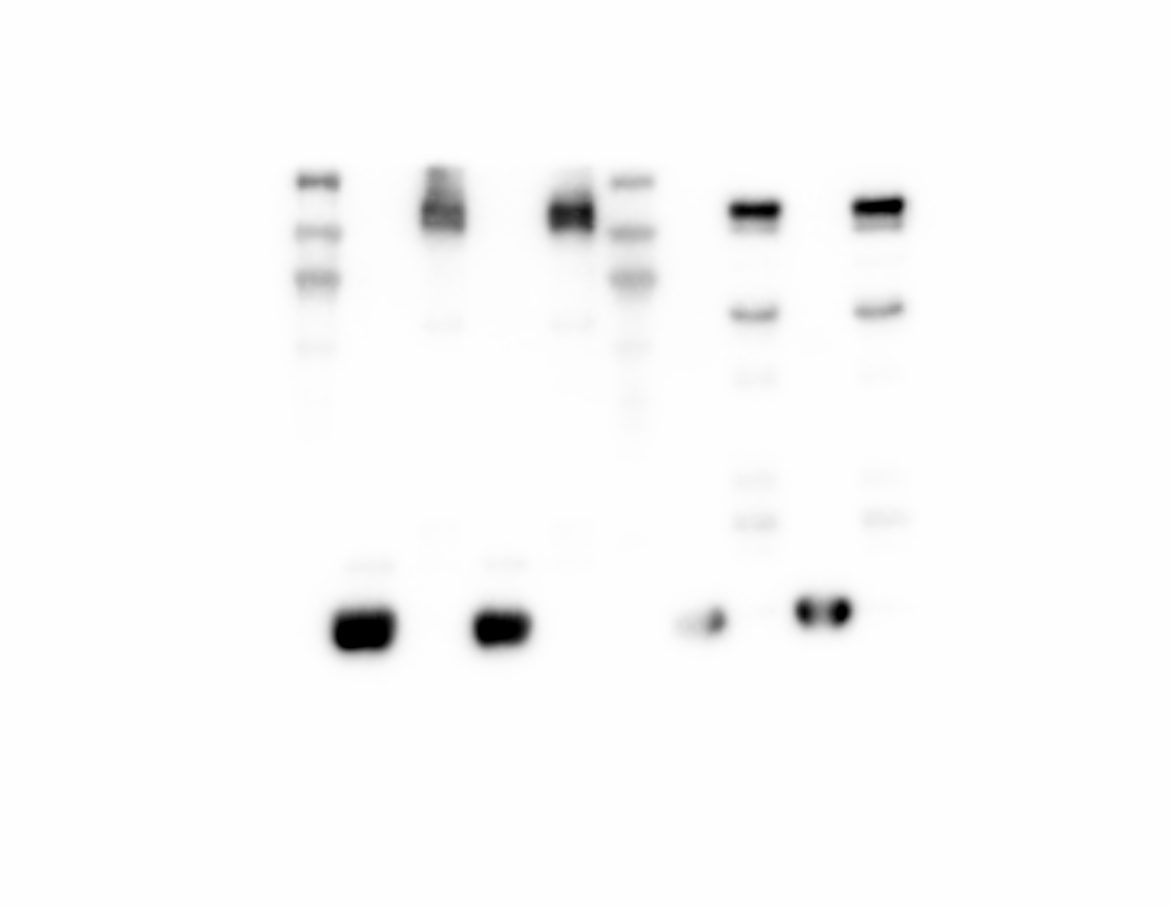

Supplement: Supplementary file 7 — Source data Fig. 3 [file 44318_2026_818_MOESM7_ESM.zip › Figure 3/Figure 3E/GST-FAM134B (left).tif]

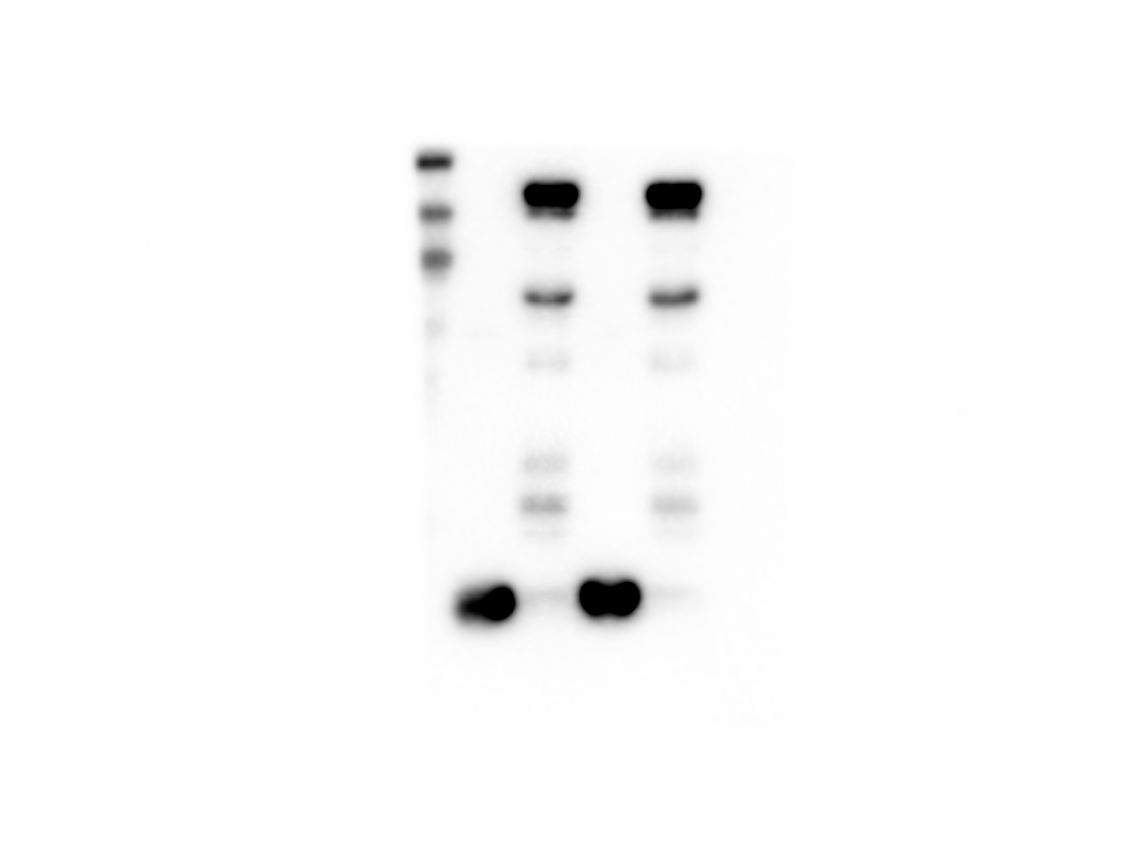

Supplement: Supplementary file 7 — Source data Fig. 3 [file 44318_2026_818_MOESM7_ESM.zip › Figure 3/Figure 3E/GST-FAM134B (right).tif]

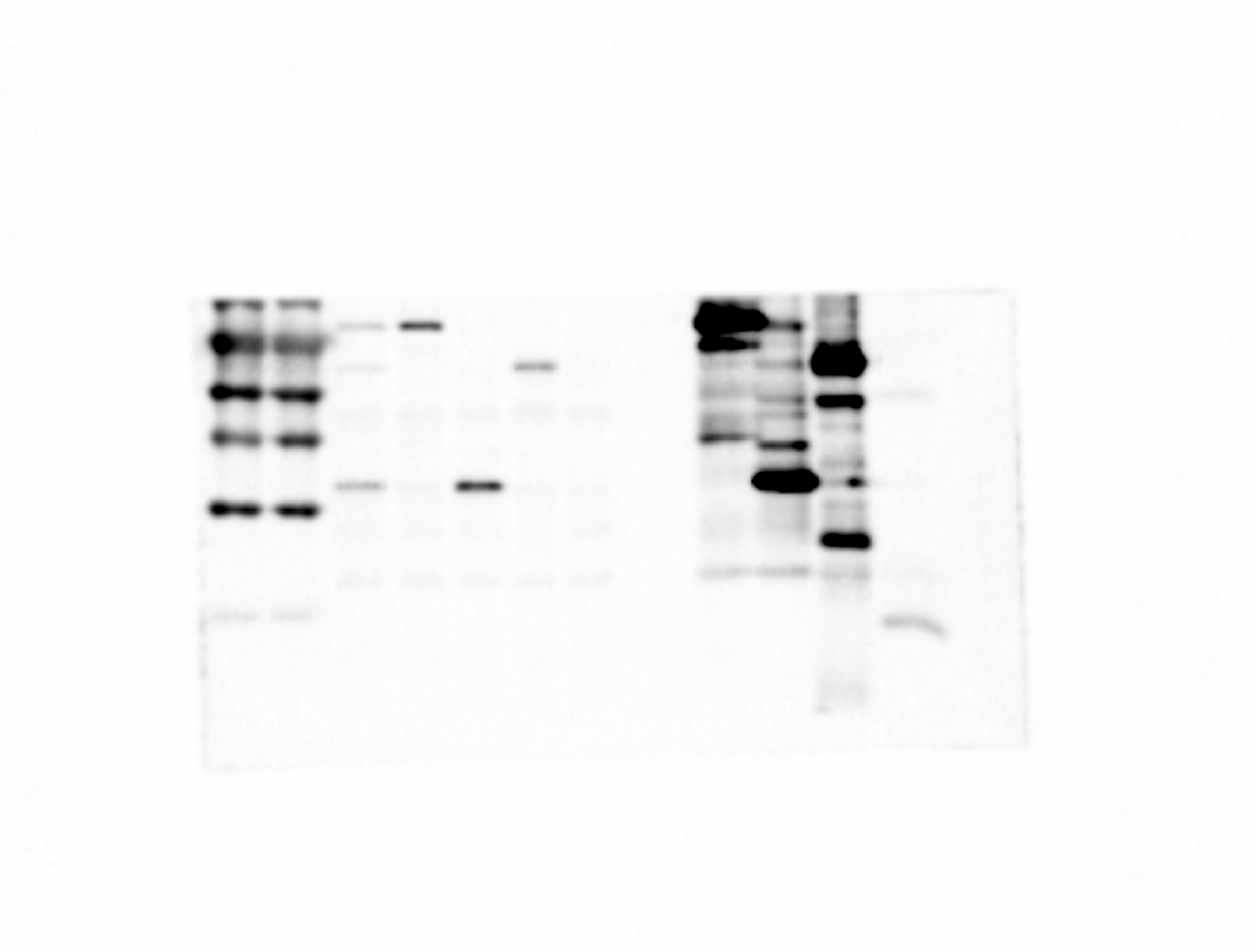

Supplement: Supplementary file 7 — Source data Fig. 3 [file 44318_2026_818_MOESM7_ESM.zip › Figure 3/Figure 3G/3HA-FAM134B (left).tif]

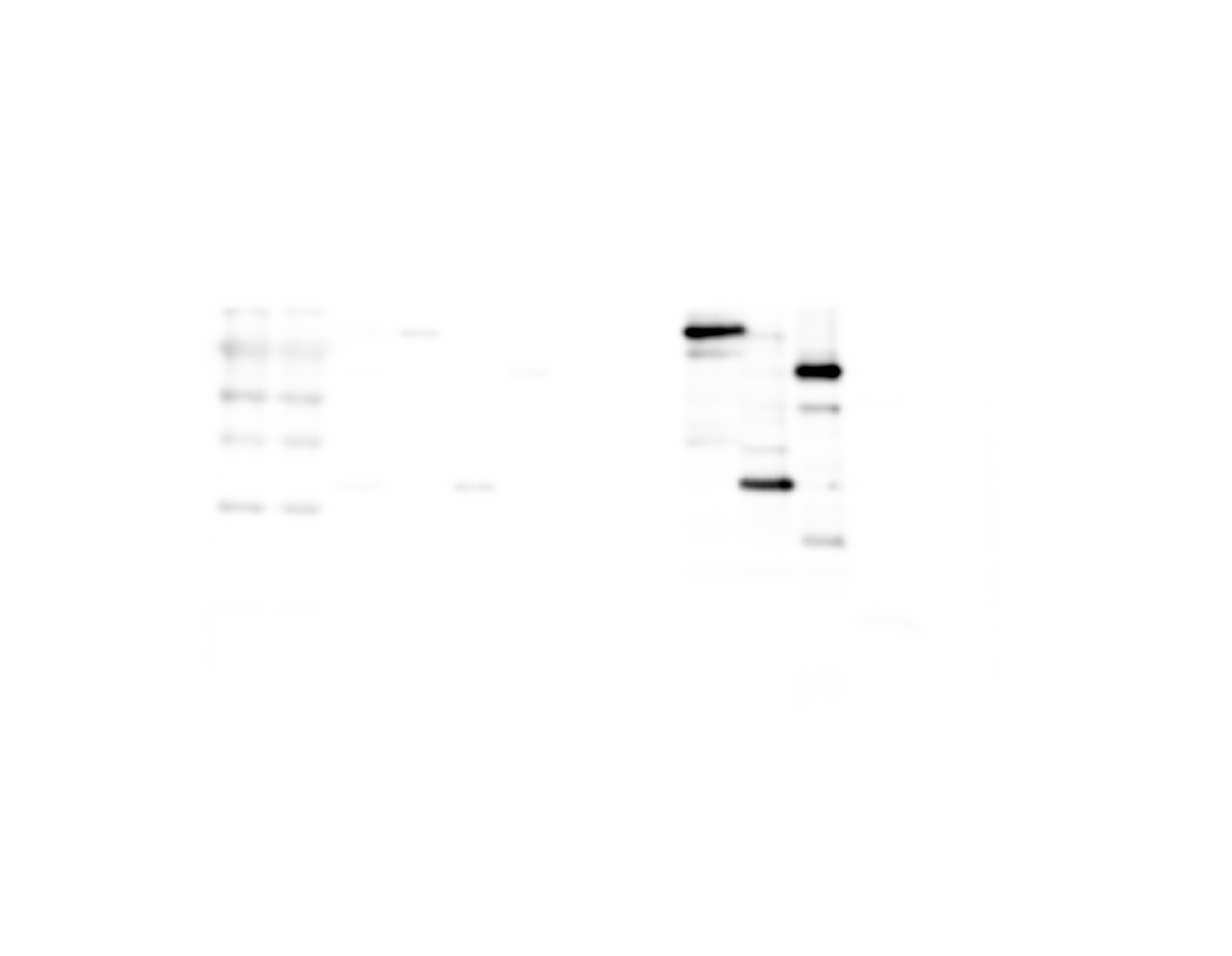

Supplement: Supplementary file 7 — Source data Fig. 3 [file 44318_2026_818_MOESM7_ESM.zip › Figure 3/Figure 3G/3HA-FAM134B (right).tif]

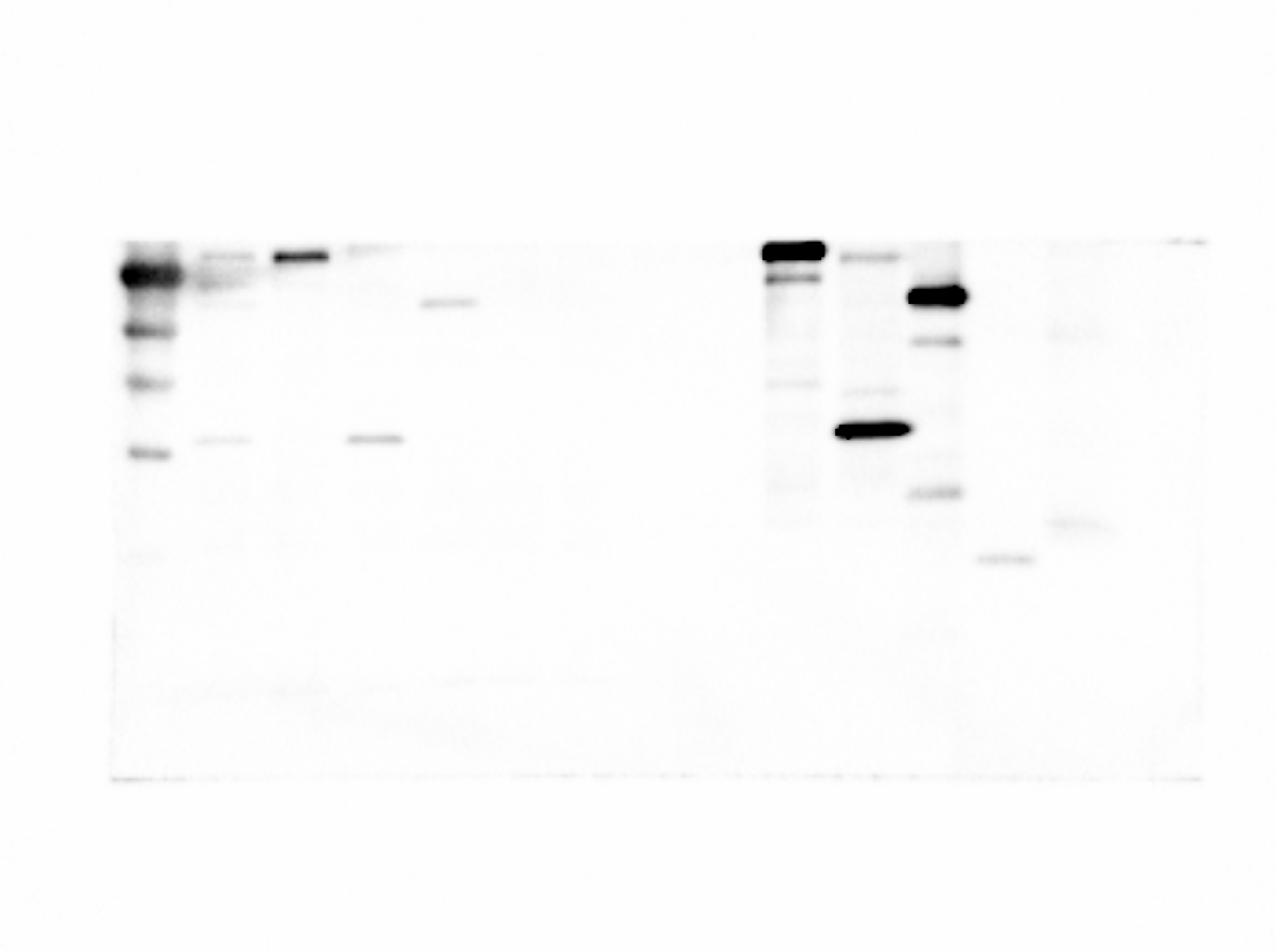

Supplement: Supplementary file 7 — Source data Fig. 3 [file 44318_2026_818_MOESM7_ESM.zip › Figure 3/Figure 3G/Figure 3G Replicate 1/3HA-FAM134B (left).tif]

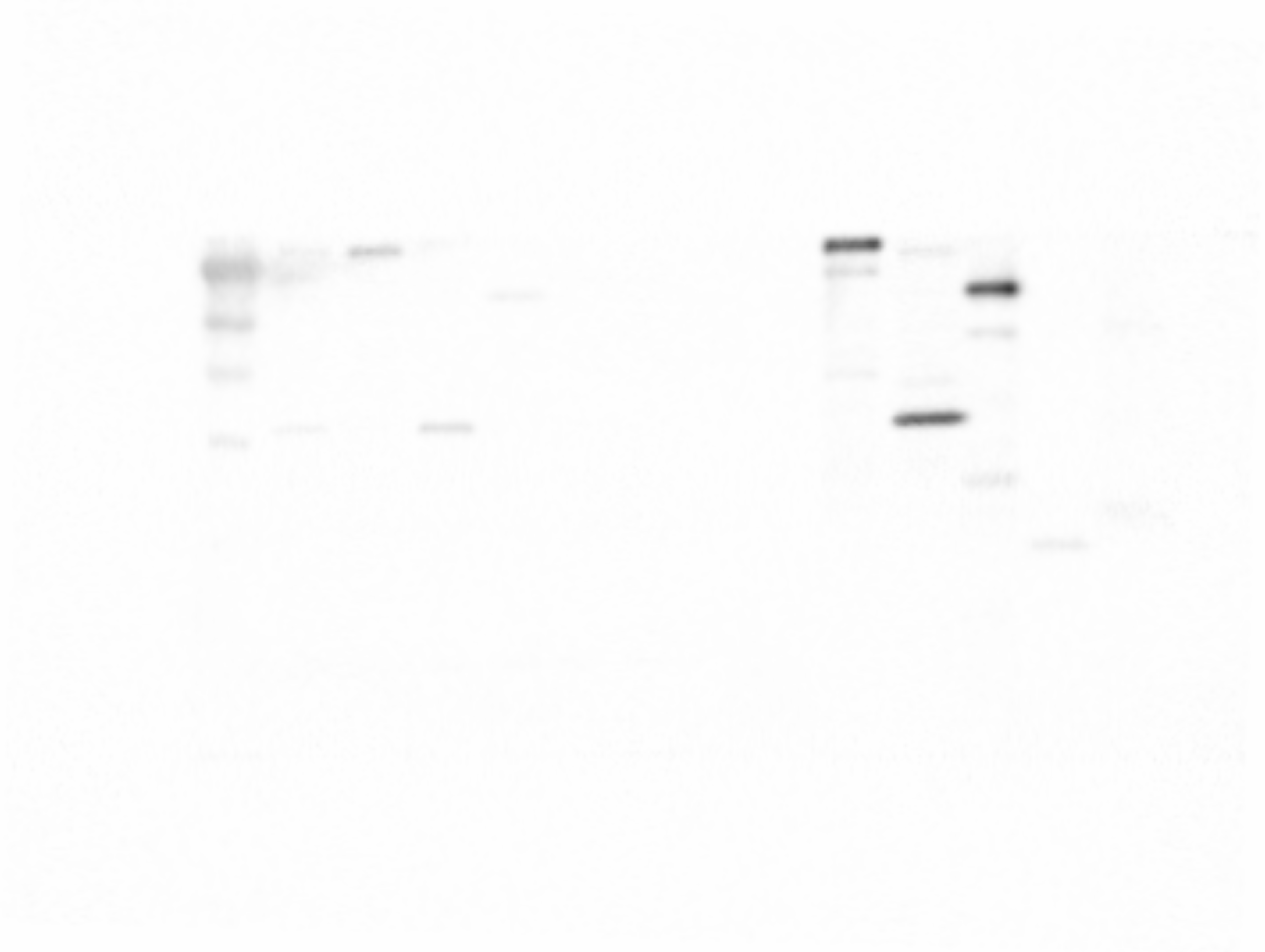

Supplement: Supplementary file 7 — Source data Fig. 3 [file 44318_2026_818_MOESM7_ESM.zip › Figure 3/Figure 3G/Figure 3G Replicate 1/3HA-FAM134B (right).tif]

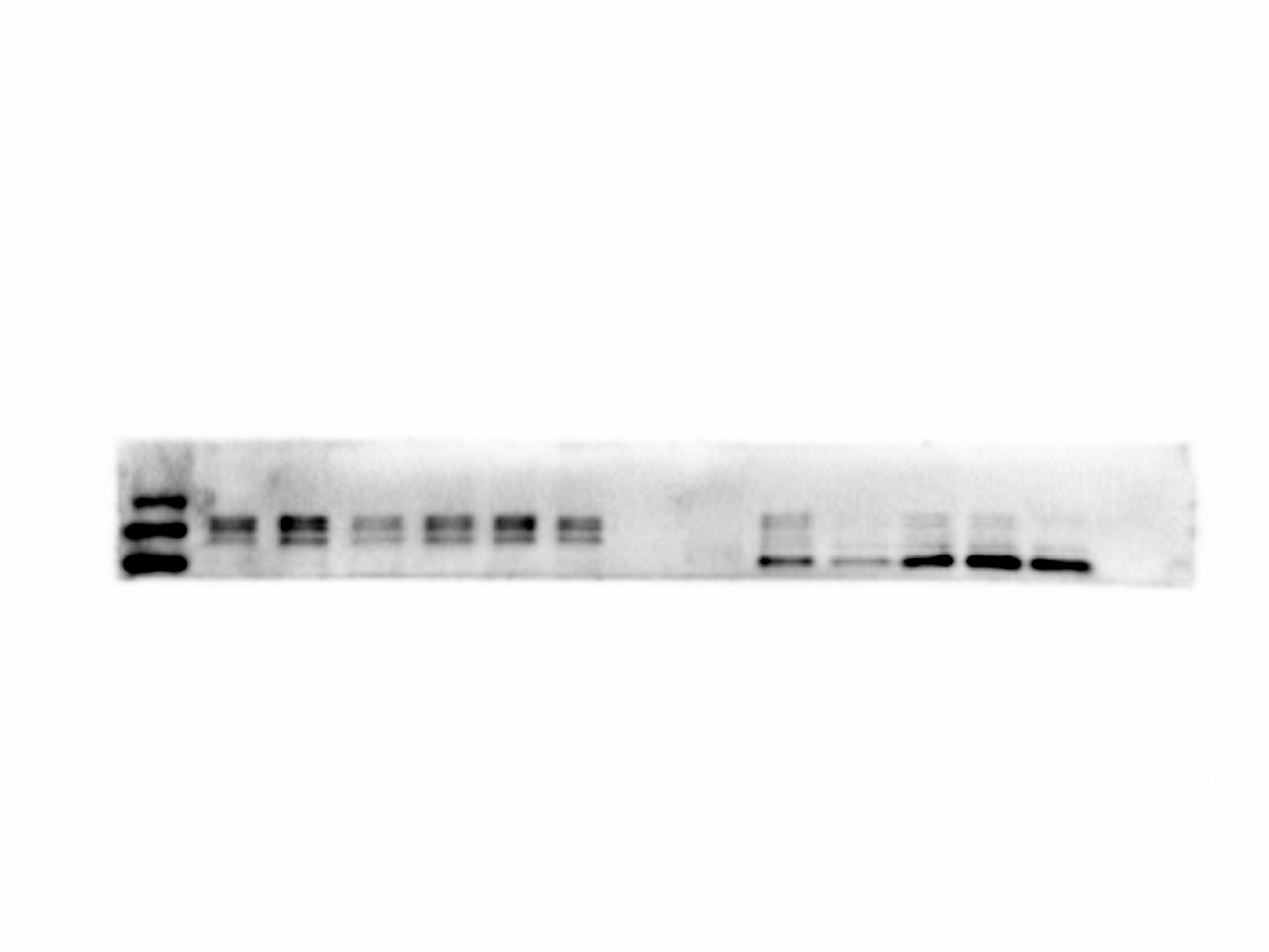

Supplement: Supplementary file 7 — Source data Fig. 3 [file 44318_2026_818_MOESM7_ESM.zip › Figure 3/Figure 3G/Figure 3G Replicate 1/Myc-APP-mCherry (left).tif]

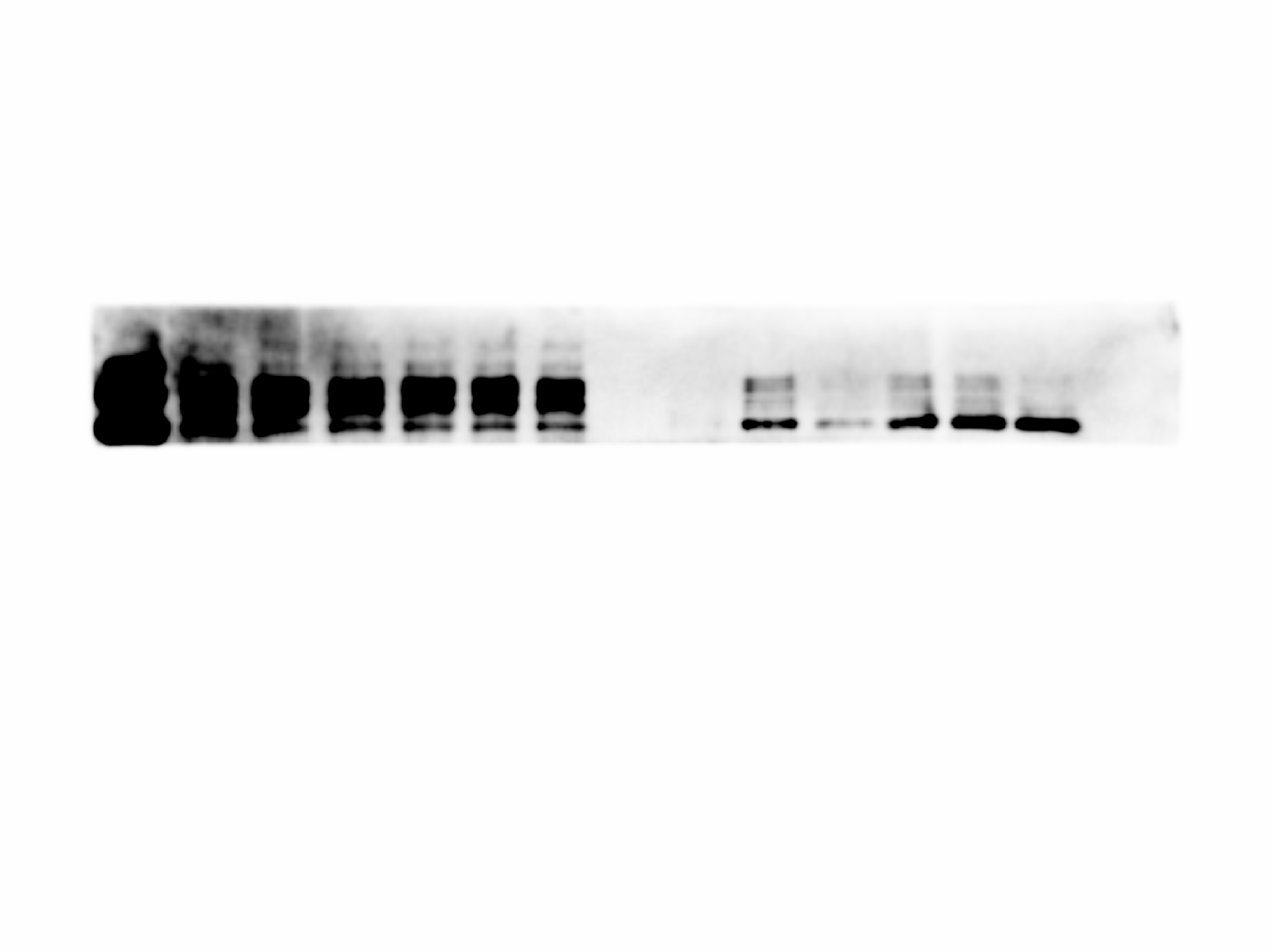

Supplement: Supplementary file 7 — Source data Fig. 3 [file 44318_2026_818_MOESM7_ESM.zip › Figure 3/Figure 3G/Figure 3G Replicate 1/Myc-APP-mCherry (right).tif]

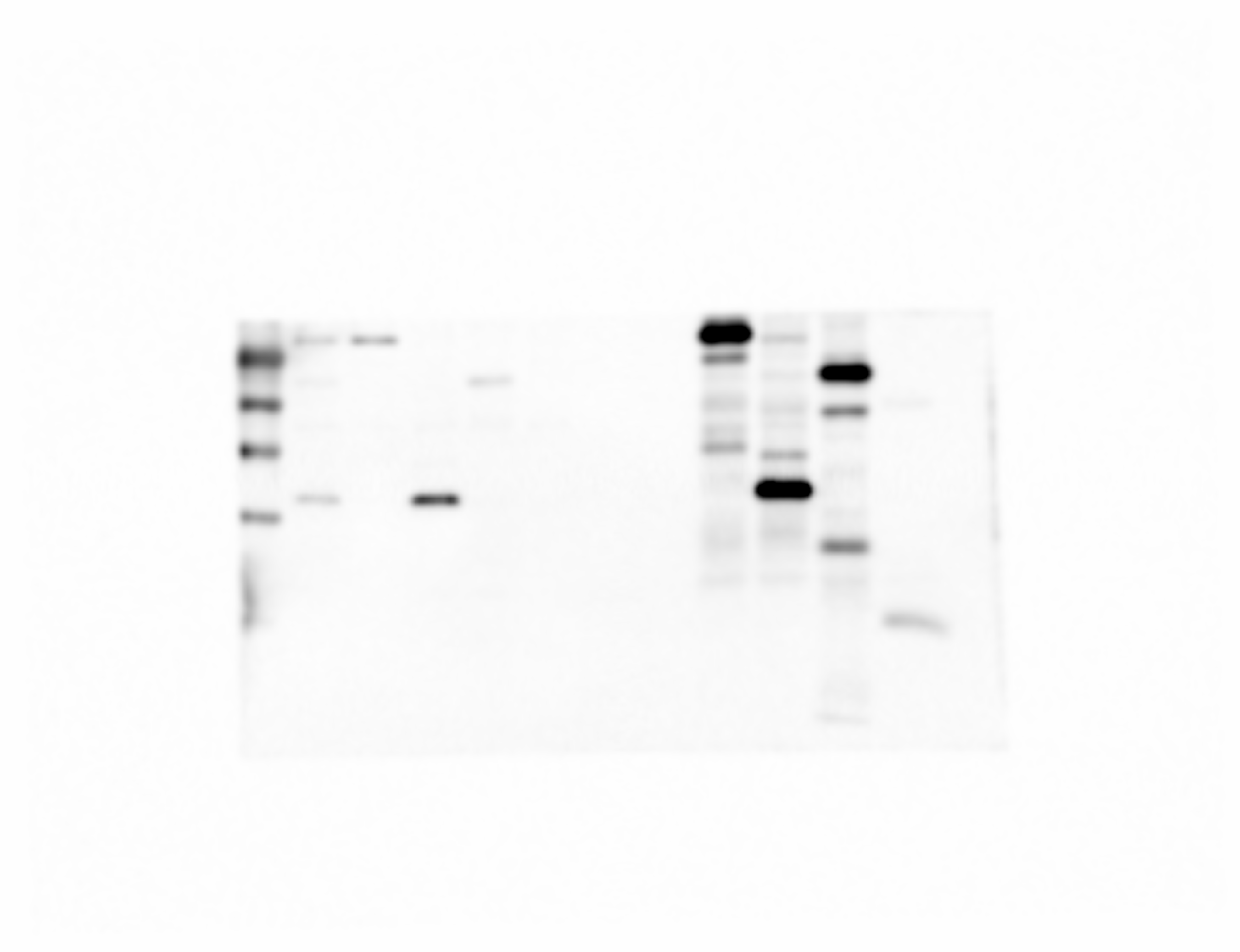

Supplement: Supplementary file 7 — Source data Fig. 3 [file 44318_2026_818_MOESM7_ESM.zip › Figure 3/Figure 3G/Figure 3G Replicate 2/3HA-FAM134B (left).tif]

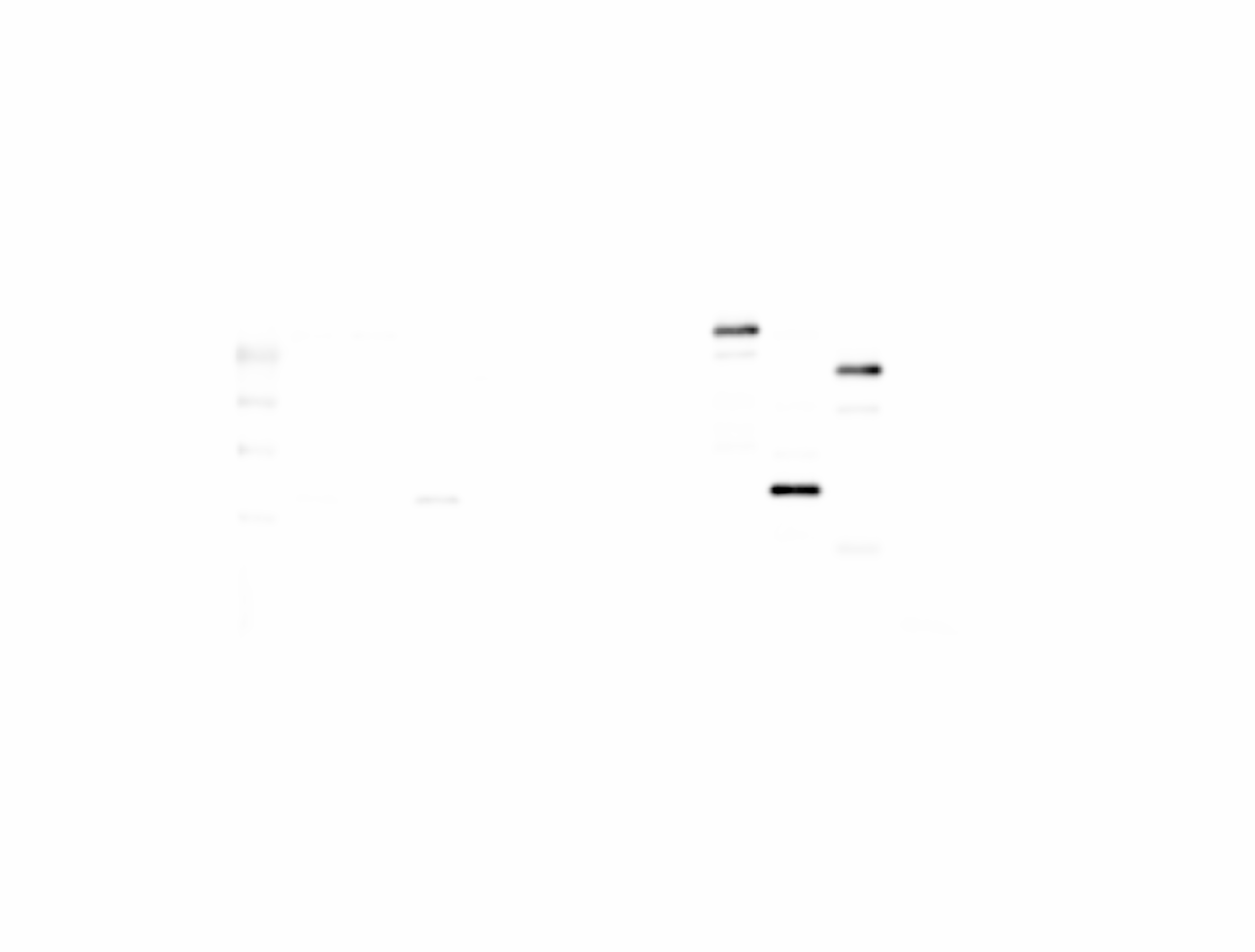

Supplement: Supplementary file 7 — Source data Fig. 3 [file 44318_2026_818_MOESM7_ESM.zip › Figure 3/Figure 3G/Figure 3G Replicate 2/3HAFAM134B (right).tif]

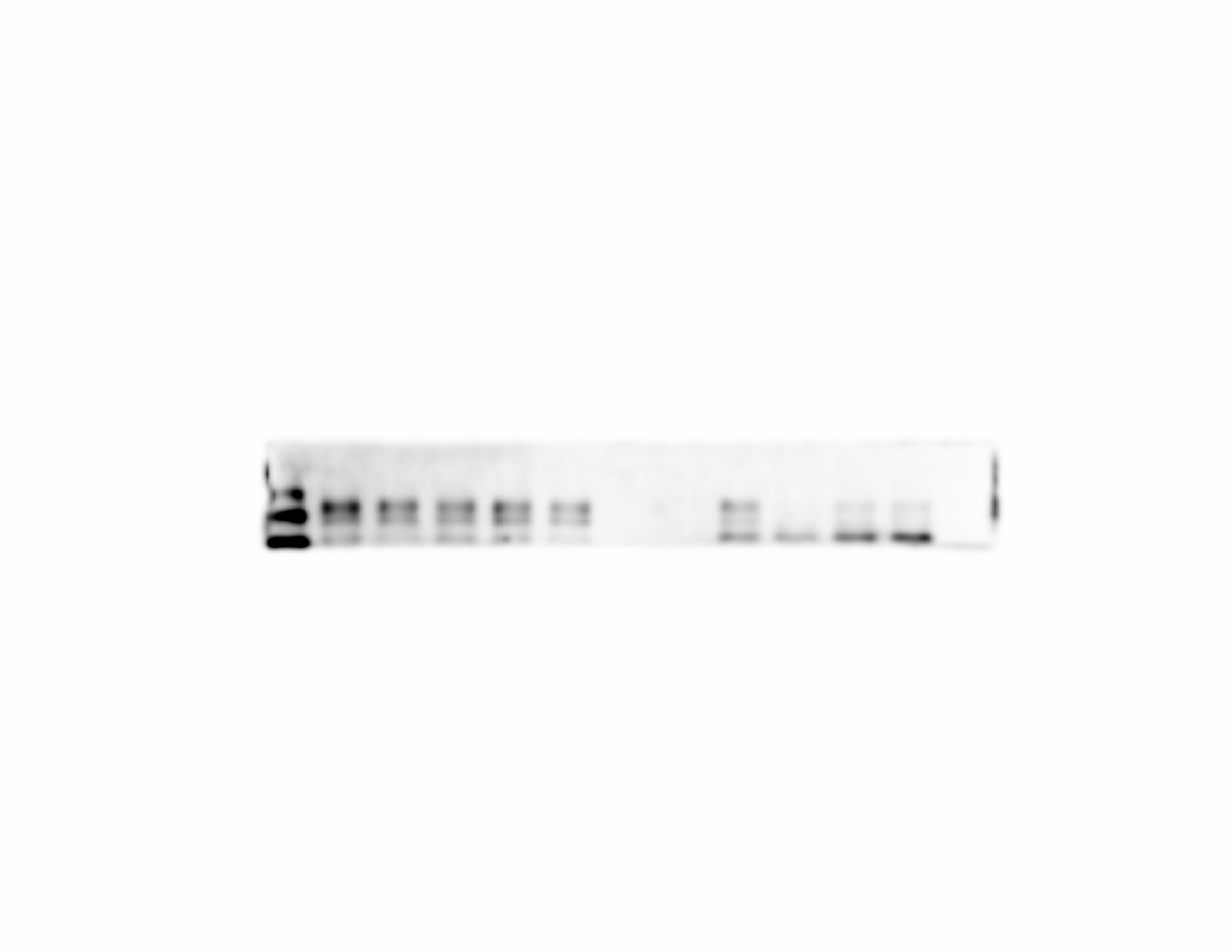

Supplement: Supplementary file 7 — Source data Fig. 3 [file 44318_2026_818_MOESM7_ESM.zip › Figure 3/Figure 3G/Figure 3G Replicate 2/Myc-APP-mCherry (left).tif]

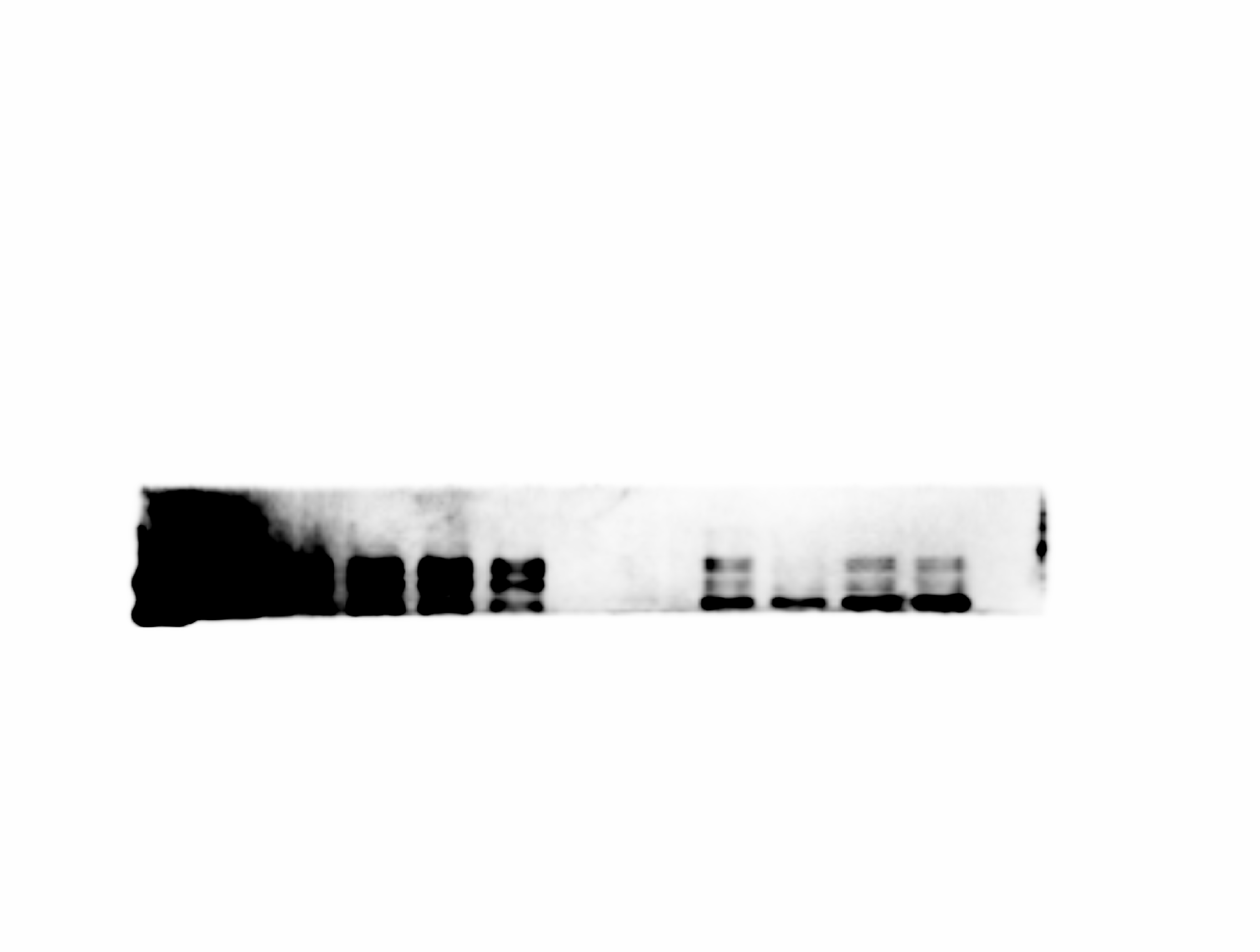

Supplement: Supplementary file 7 — Source data Fig. 3 [file 44318_2026_818_MOESM7_ESM.zip › Figure 3/Figure 3G/Figure 3G Replicate 2/Myc-APP-mCherry (right).tif]

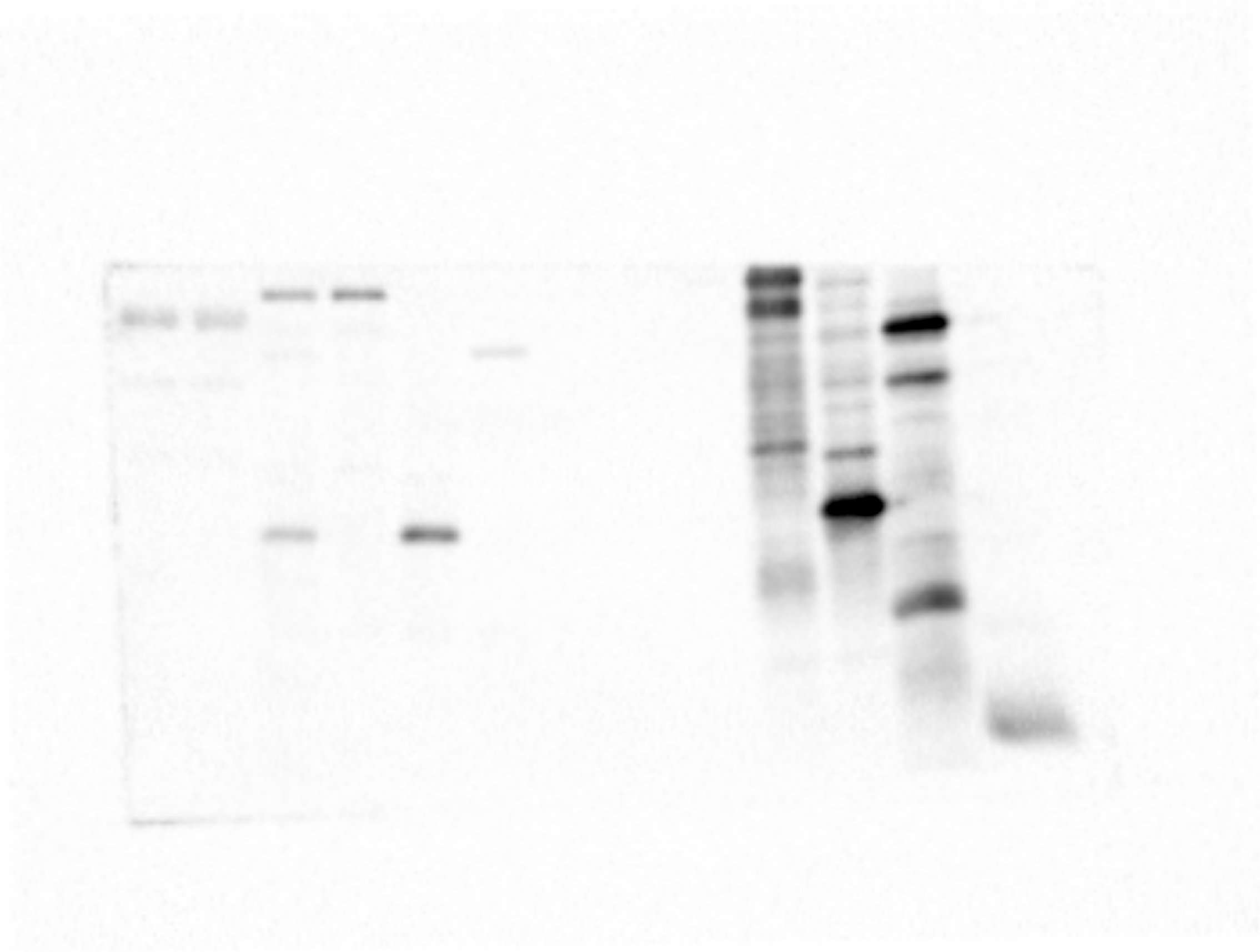

Supplement: Supplementary file 7 — Source data Fig. 3 [file 44318_2026_818_MOESM7_ESM.zip › Figure 3/Figure 3G/Figure 3G Replicate 3/3HA-FAM134B (left).tif]

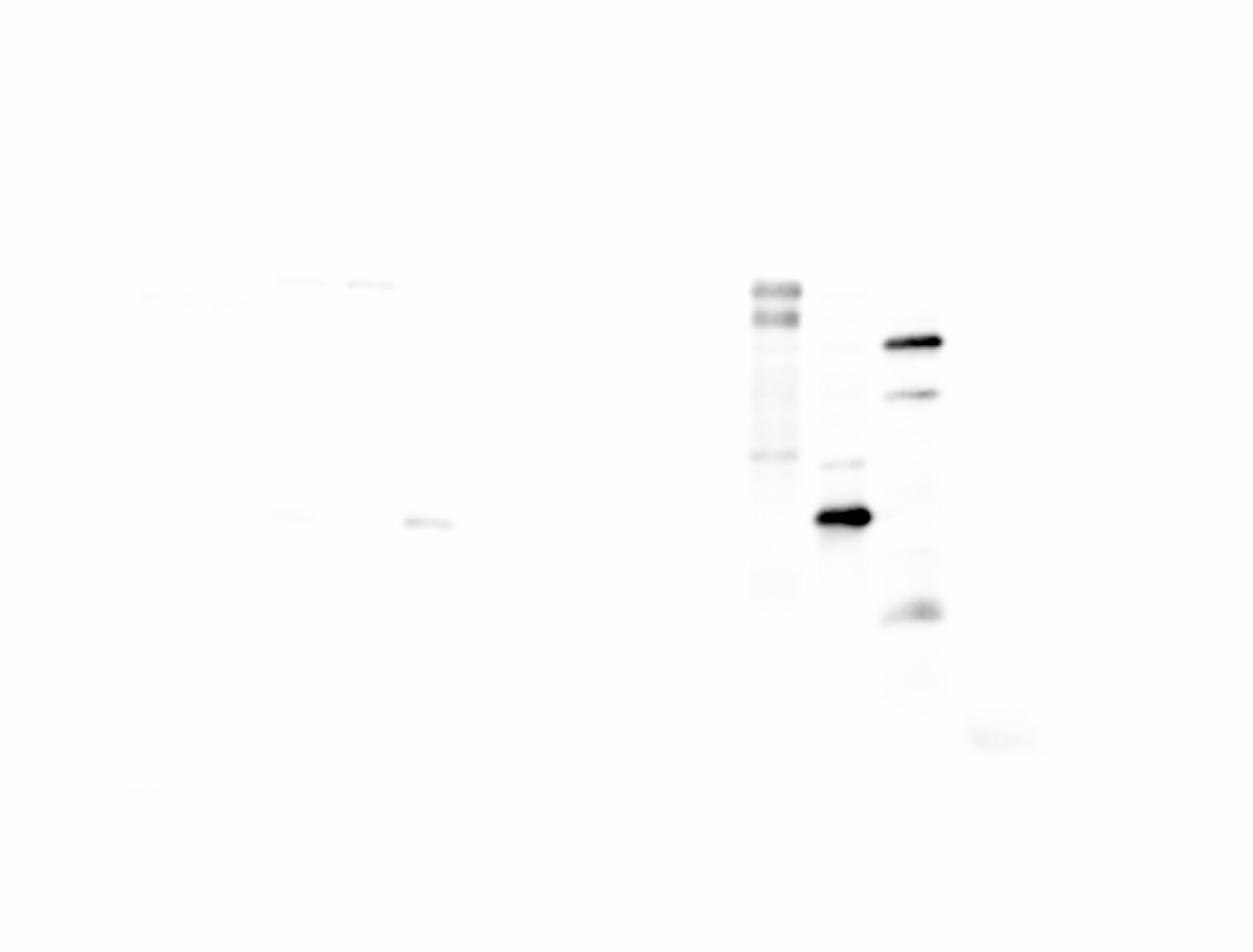

Supplement: Supplementary file 7 — Source data Fig. 3 [file 44318_2026_818_MOESM7_ESM.zip › Figure 3/Figure 3G/Figure 3G Replicate 3/3HA-FAM134B (right).tif]

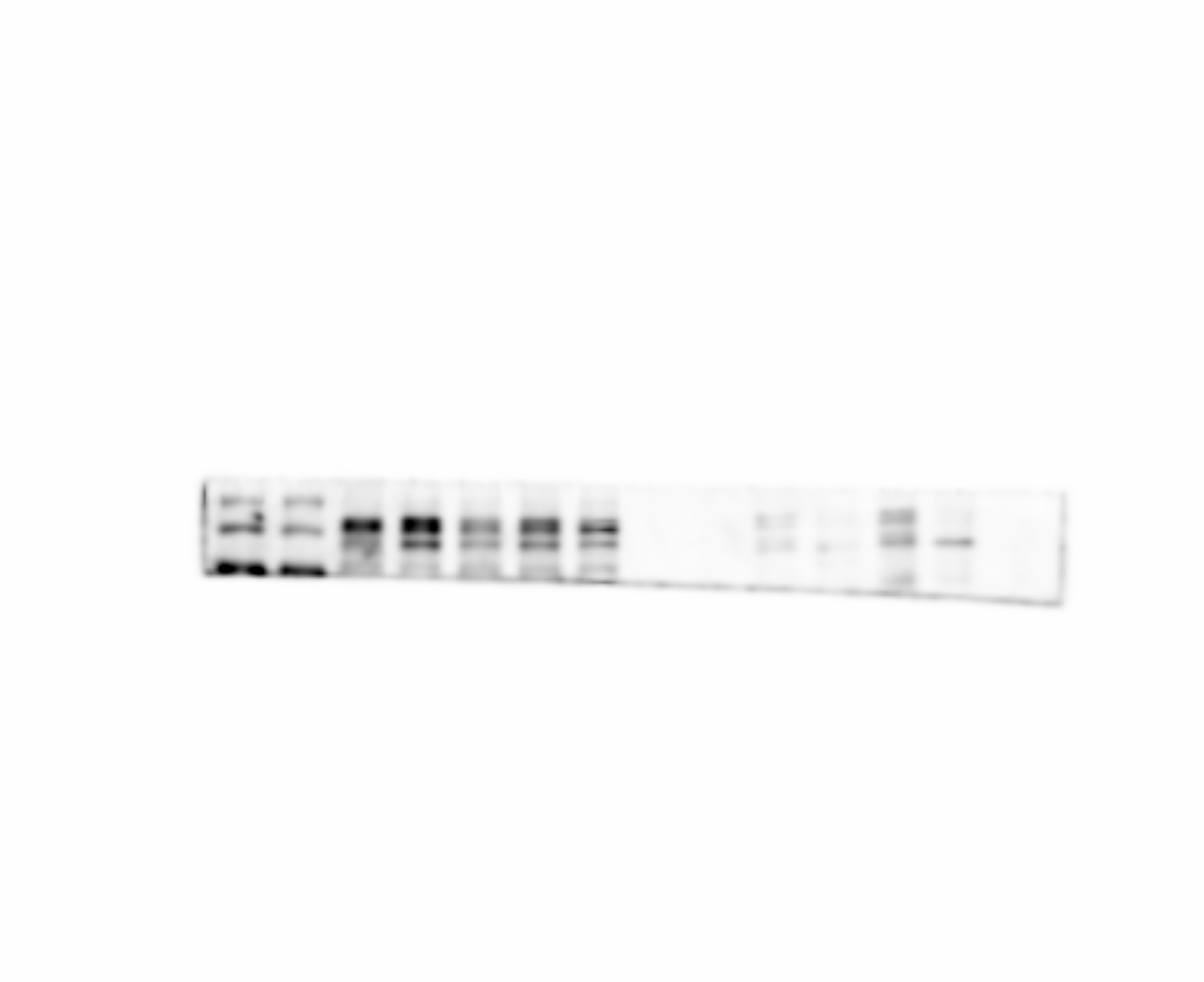

Supplement: Supplementary file 7 — Source data Fig. 3 [file 44318_2026_818_MOESM7_ESM.zip › Figure 3/Figure 3G/Figure 3G Replicate 3/Myc-APP-mCherry (left).tif]

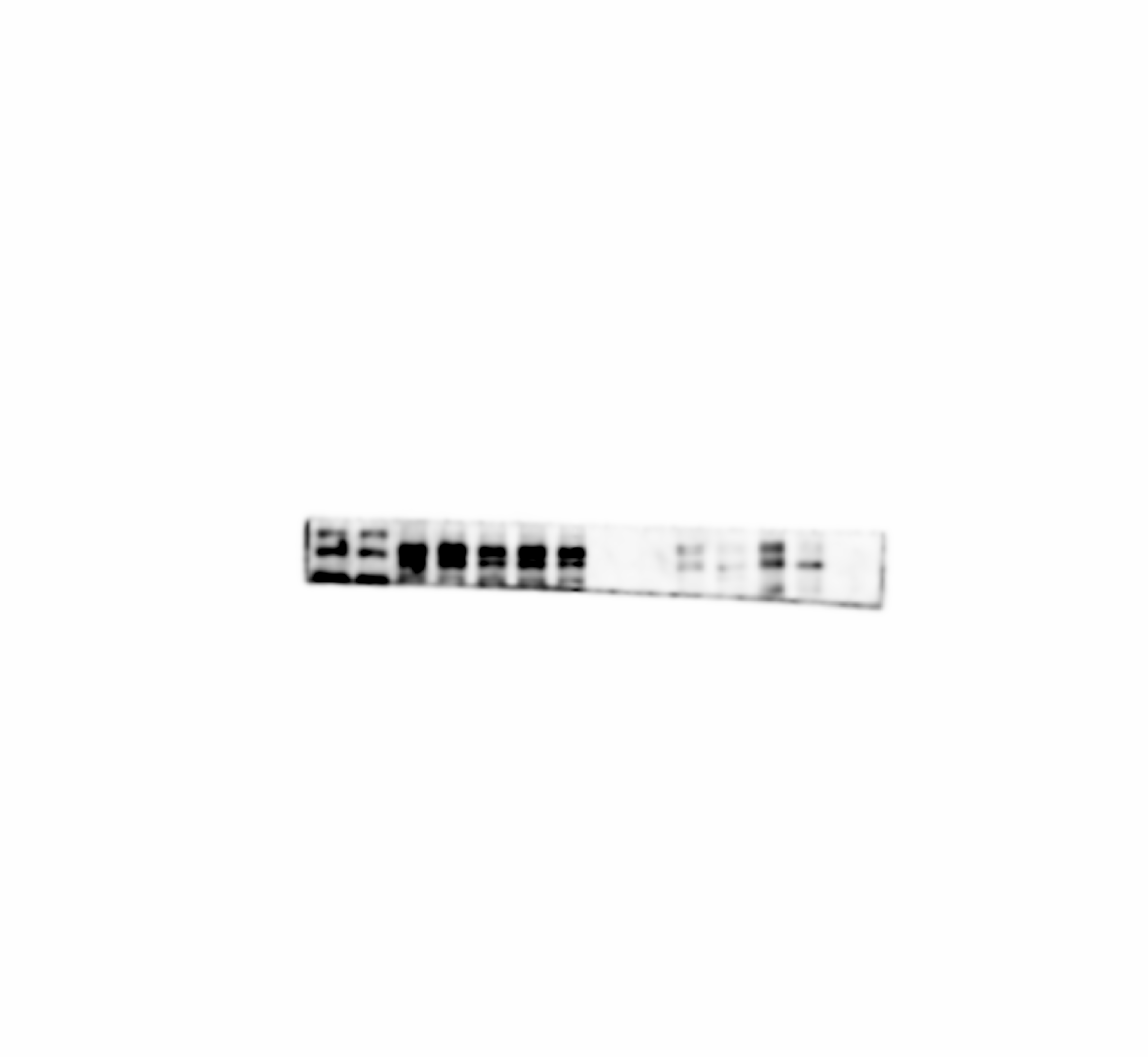

Supplement: Supplementary file 7 — Source data Fig. 3 [file 44318_2026_818_MOESM7_ESM.zip › Figure 3/Figure 3G/Figure 3G Replicate 3/Myc-APP-mCherry (right).tif]

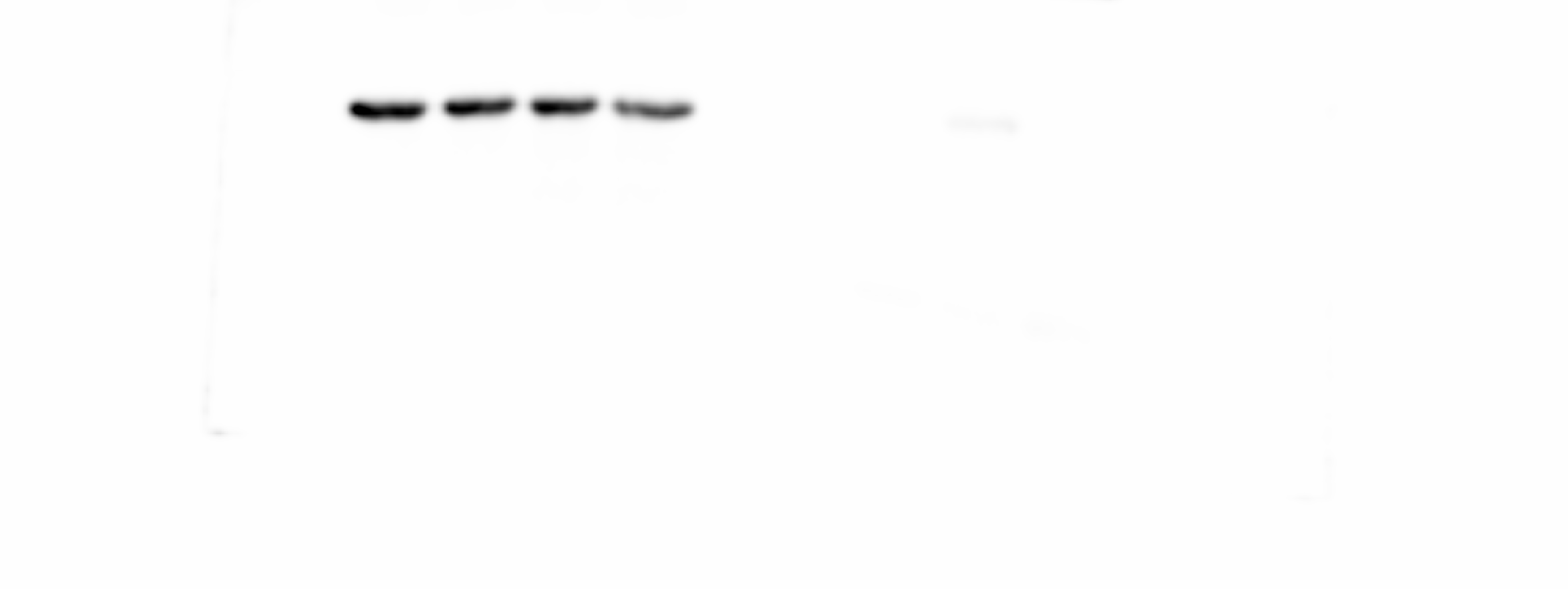

Supplement: Supplementary file 7 — Source data Fig. 3 [file 44318_2026_818_MOESM7_ESM.zip › Figure 3/Figure 3G/GAPDH.tif]

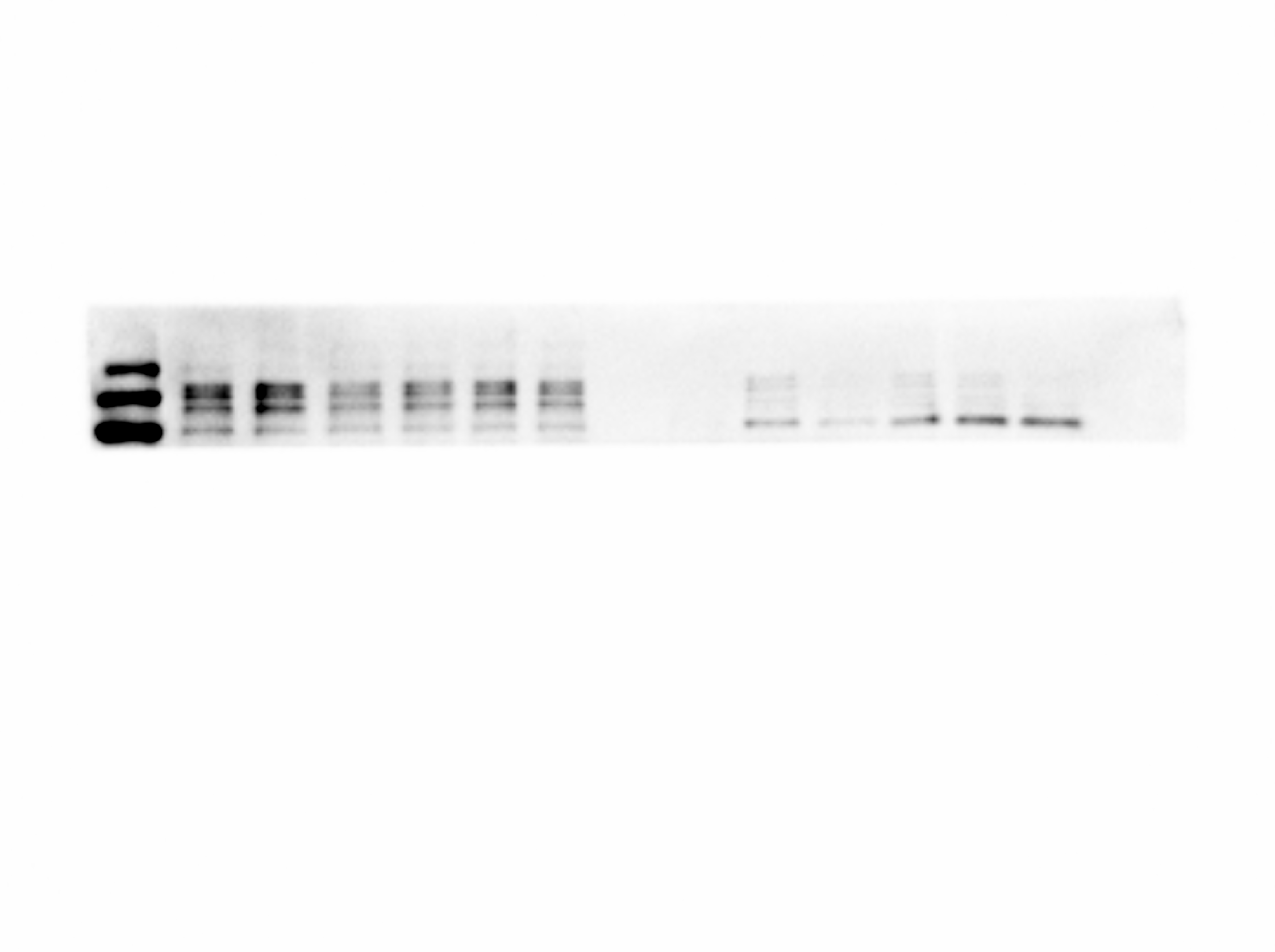

Supplement: Supplementary file 7 — Source data Fig. 3 [file 44318_2026_818_MOESM7_ESM.zip › Figure 3/Figure 3G/Myc-APP-mCherry (left).tif]

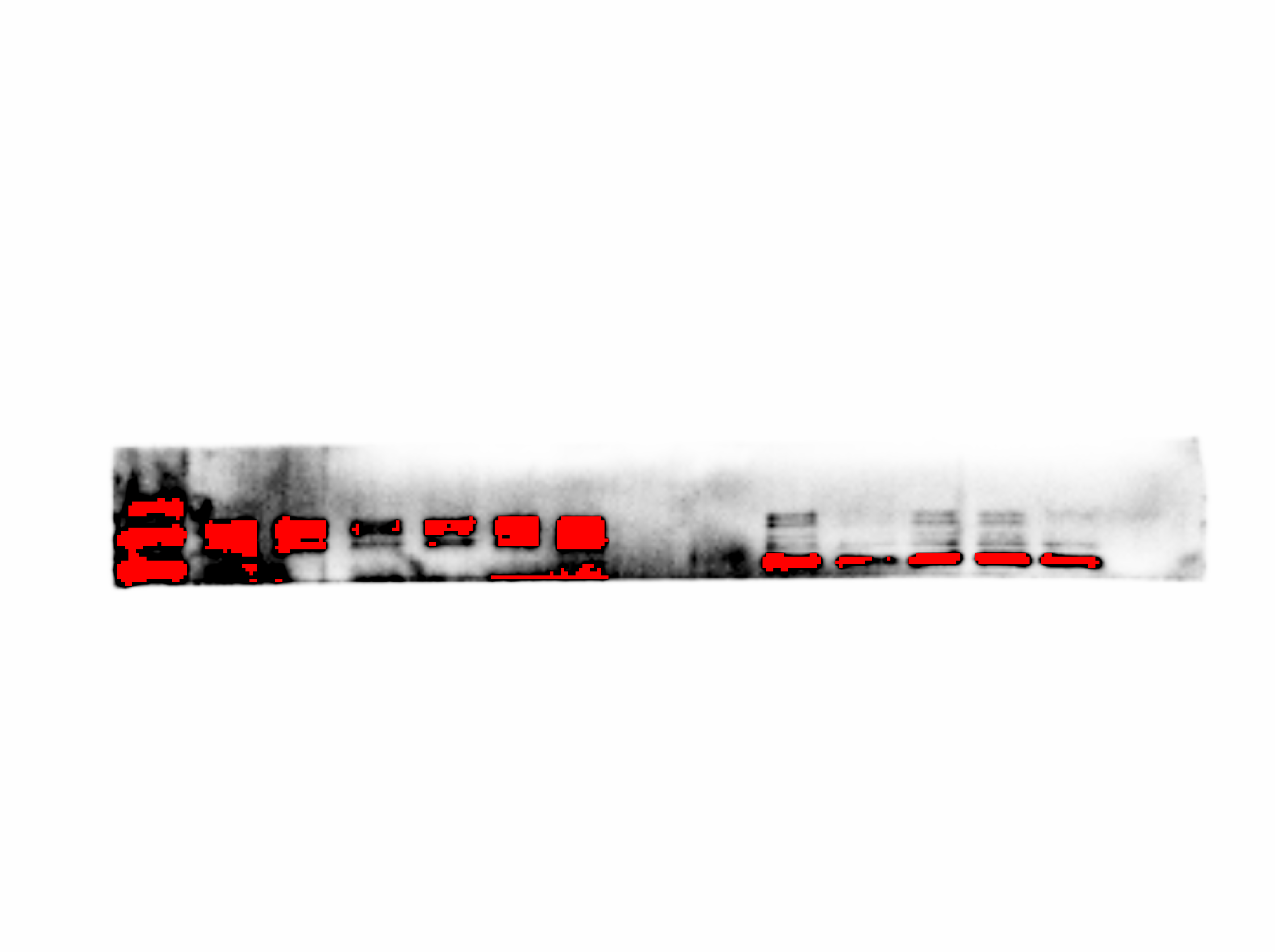

Supplement: Supplementary file 7 — Source data Fig. 3 [file 44318_2026_818_MOESM7_ESM.zip › Figure 3/Figure 3G/Myc-APP-mCherry (right).tif]

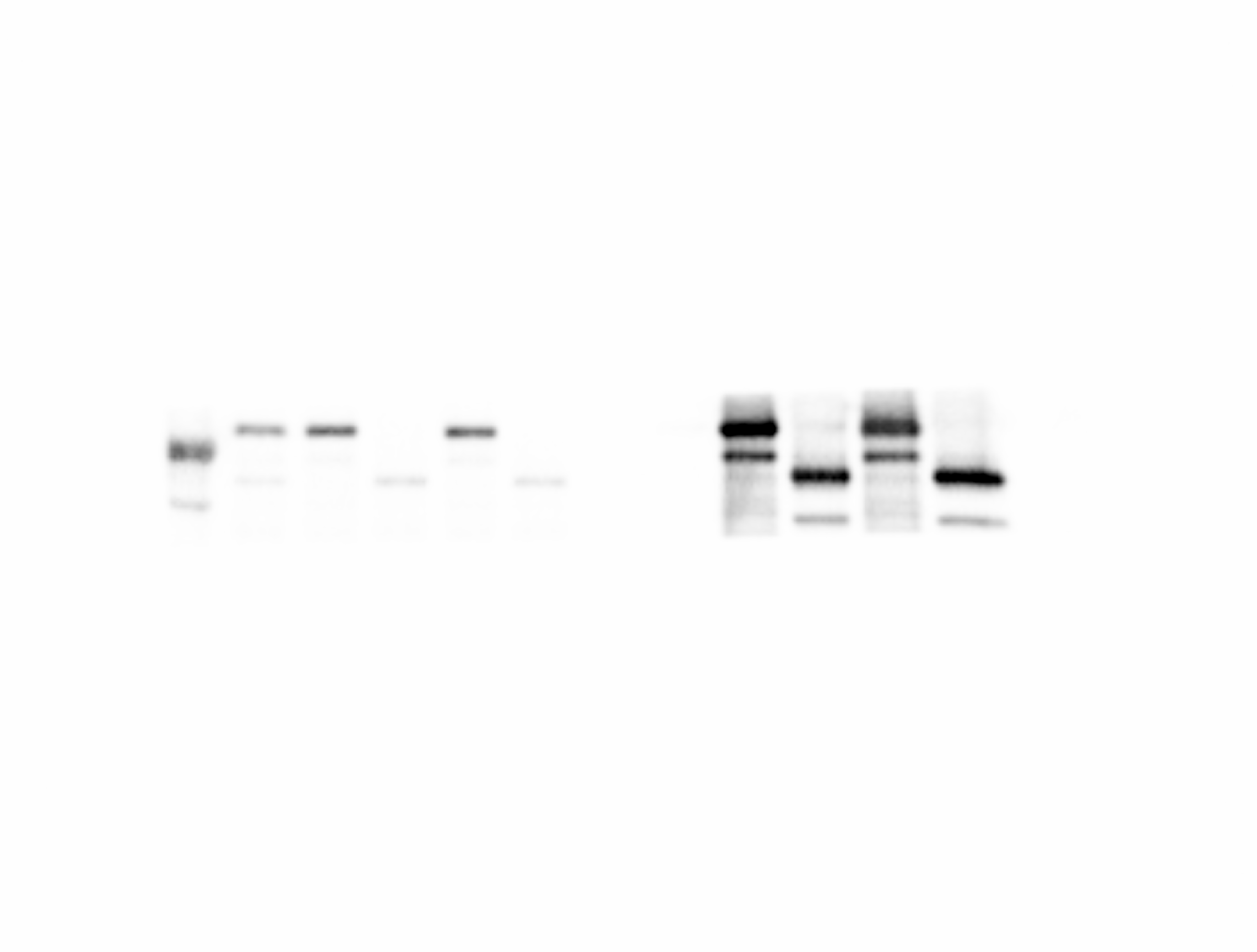

Supplement: Supplementary file 7 — Source data Fig. 3 [file 44318_2026_818_MOESM7_ESM.zip › Figure 3/Figure 3I/3HA.tif]

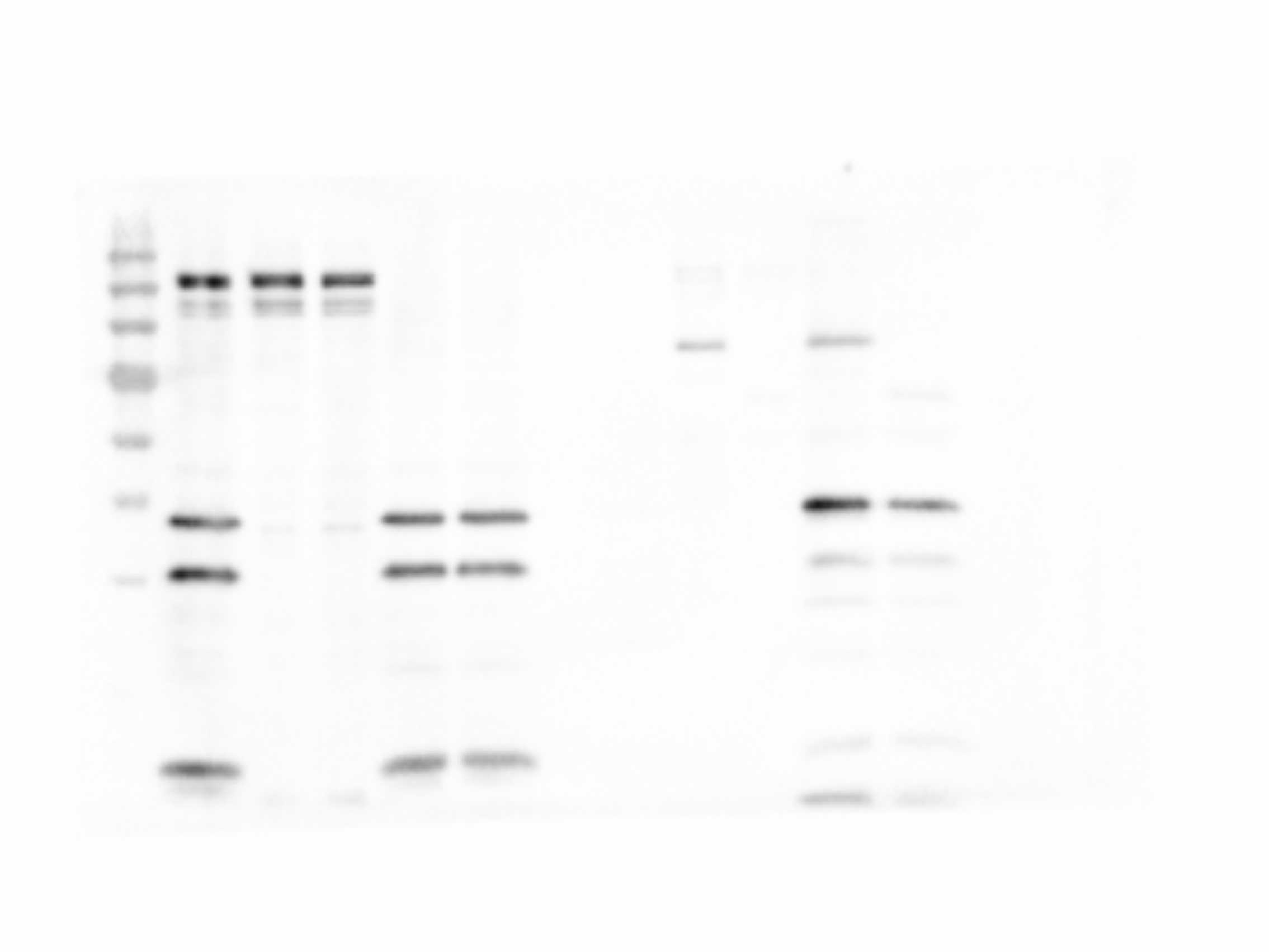

Supplement: Supplementary file 7 — Source data Fig. 3 [file 44318_2026_818_MOESM7_ESM.zip › Figure 3/Figure 3I/APP 1-648-mCherry.tif]

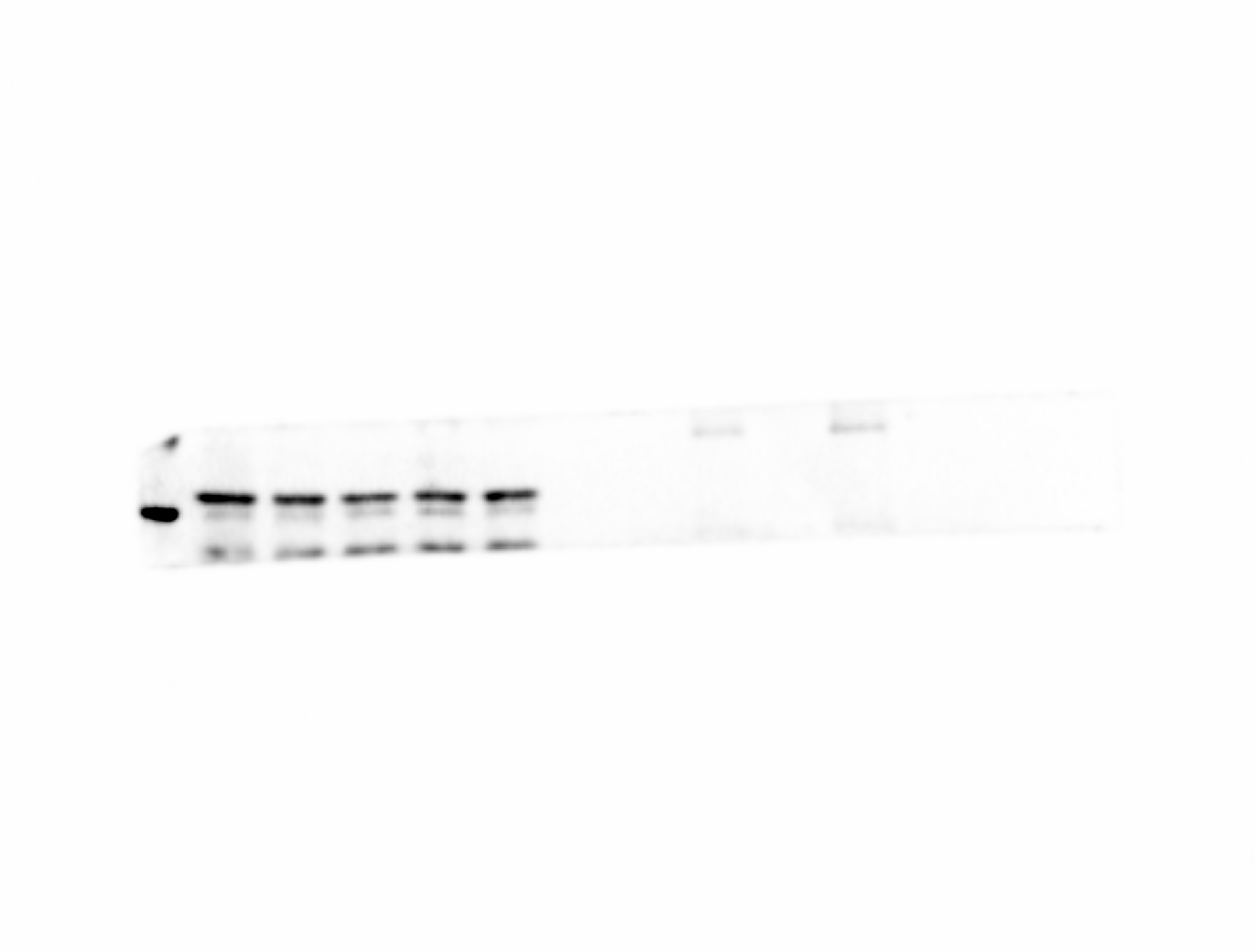

Supplement: Supplementary file 7 — Source data Fig. 3 [file 44318_2026_818_MOESM7_ESM.zip › Figure 3/Figure 3I/GAPDH.tif]

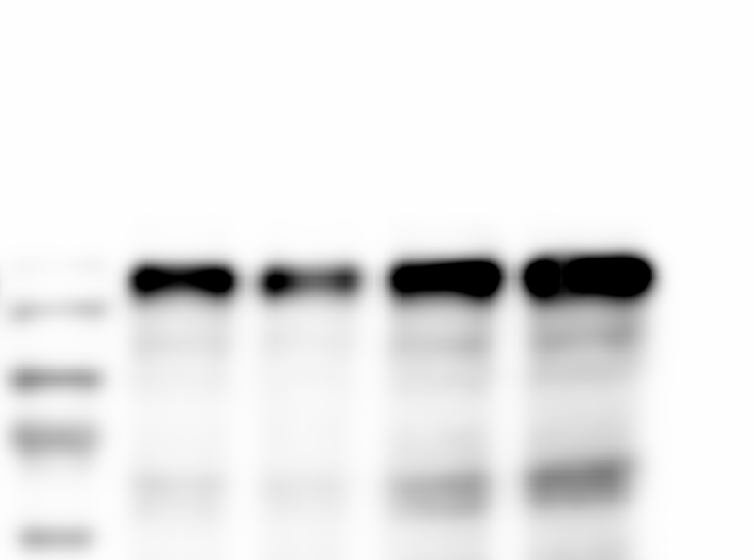

Supplement: Supplementary file 7 — Source data Fig. 3 [file 44318_2026_818_MOESM7_ESM.zip › Figure 3/Figure 3L/APPWT-FKBP12.tif]

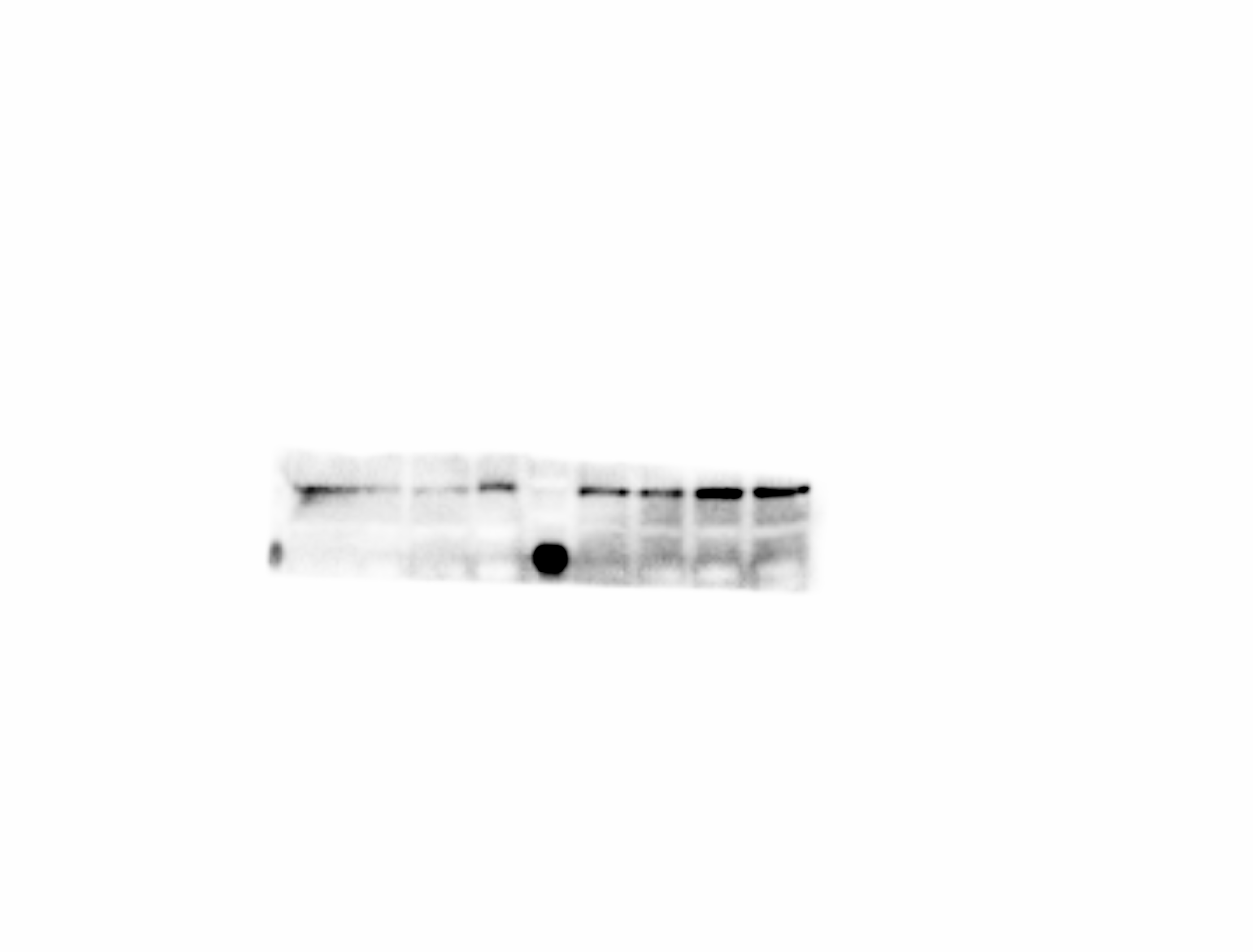

Supplement: Supplementary file 7 — Source data Fig. 3 [file 44318_2026_818_MOESM7_ESM.zip › Figure 3/Figure 3L/Figure 3L Replicate 1/APP (lane 5-8).tif]

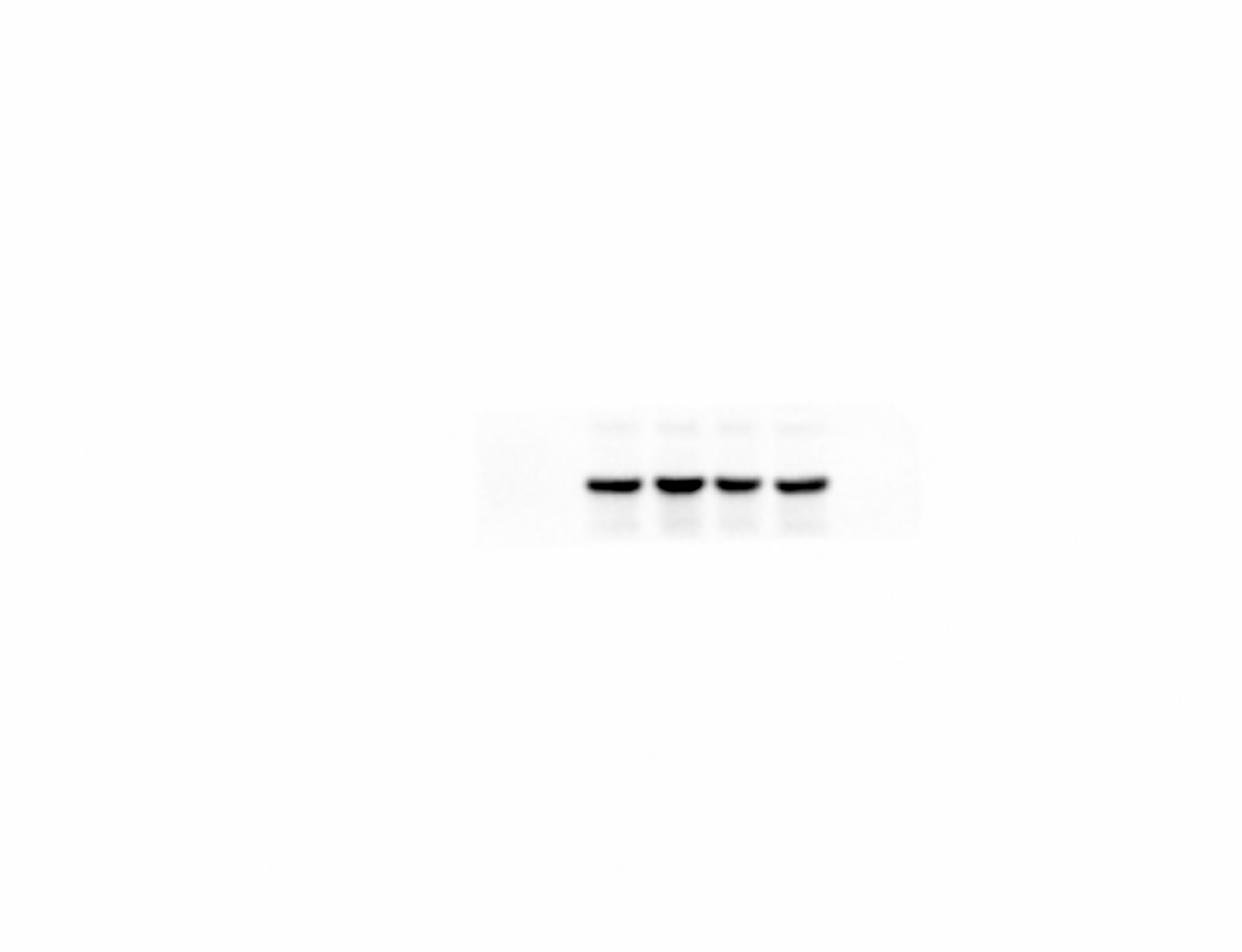

Supplement: Supplementary file 7 — Source data Fig. 3 [file 44318_2026_818_MOESM7_ESM.zip › Figure 3/Figure 3L/Figure 3L Replicate 1/GAPDH.tif]

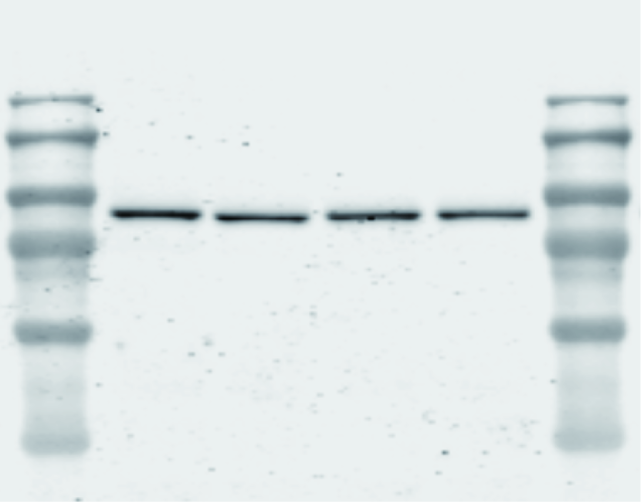

Supplement: Supplementary file 7 — Source data Fig. 3 [file 44318_2026_818_MOESM7_ESM.zip › Figure 3/Figure 3L/Figure 3L Replicate 1/HA.tif]

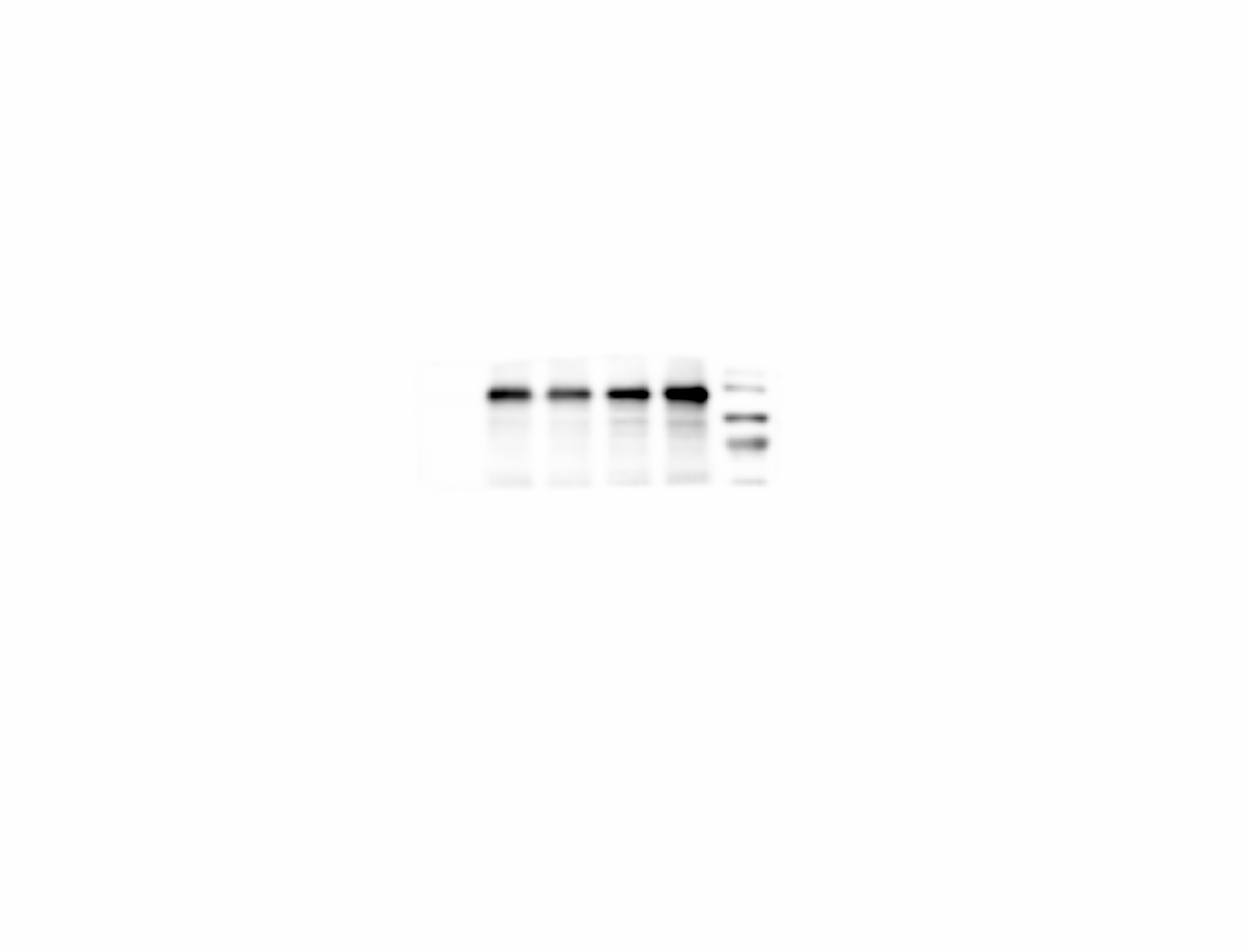

Supplement: Supplementary file 7 — Source data Fig. 3 [file 44318_2026_818_MOESM7_ESM.zip › Figure 3/Figure 3L/Figure 3L Replicate 2/APP.tif]

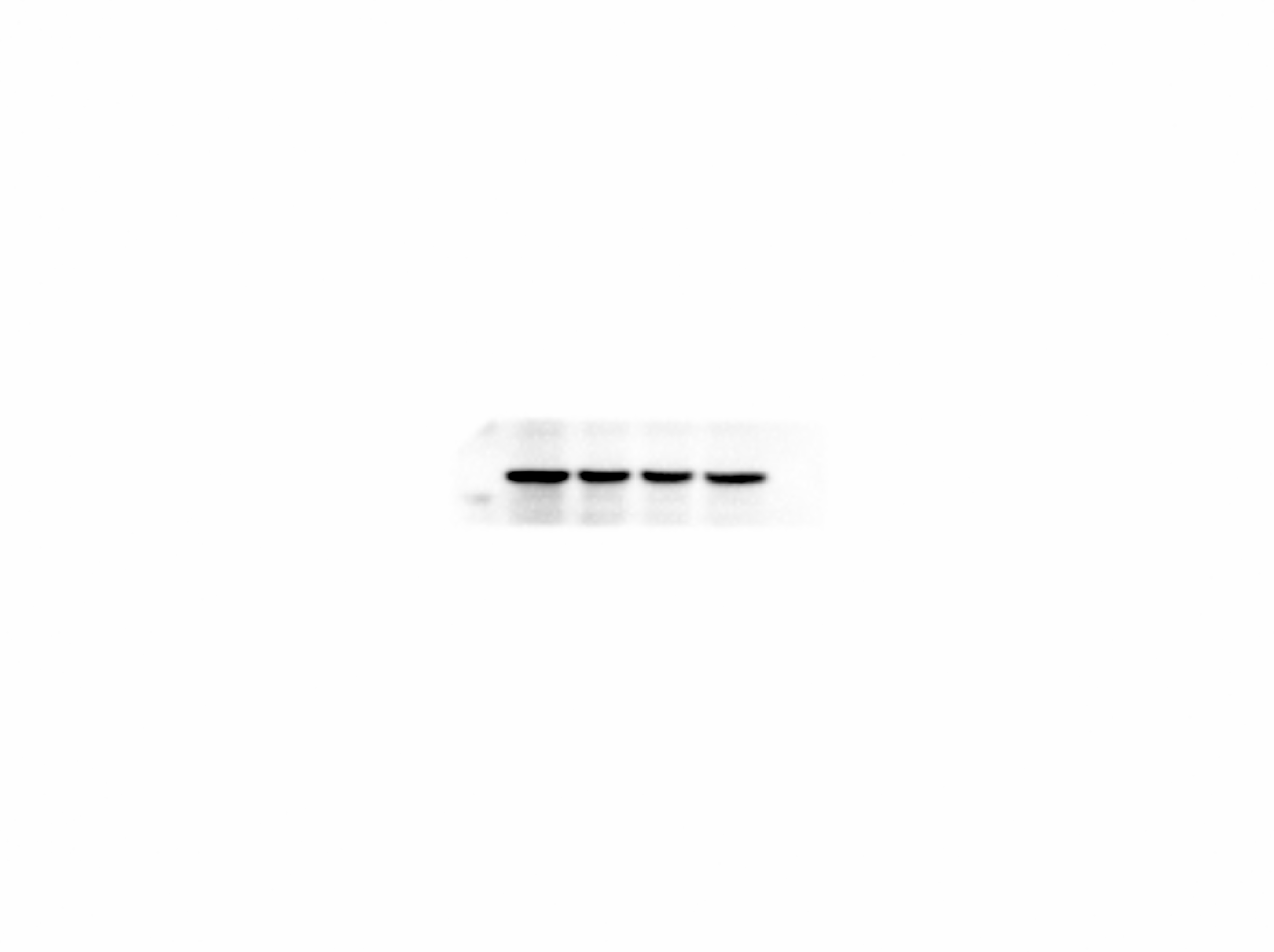

Supplement: Supplementary file 7 — Source data Fig. 3 [file 44318_2026_818_MOESM7_ESM.zip › Figure 3/Figure 3L/Figure 3L Replicate 2/GAPDH.tif]

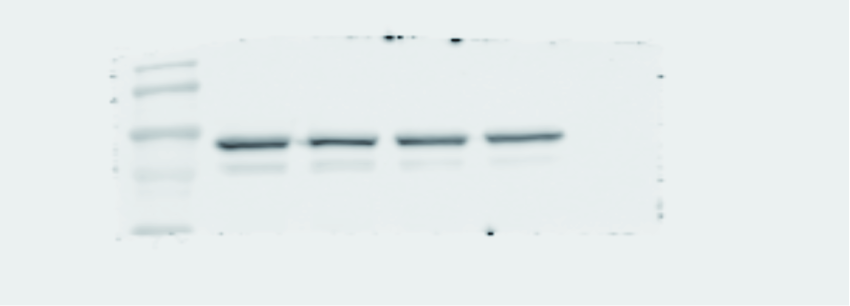

Supplement: Supplementary file 7 — Source data Fig. 3 [file 44318_2026_818_MOESM7_ESM.zip › Figure 3/Figure 3L/Figure 3L Replicate 2/HA.tif]

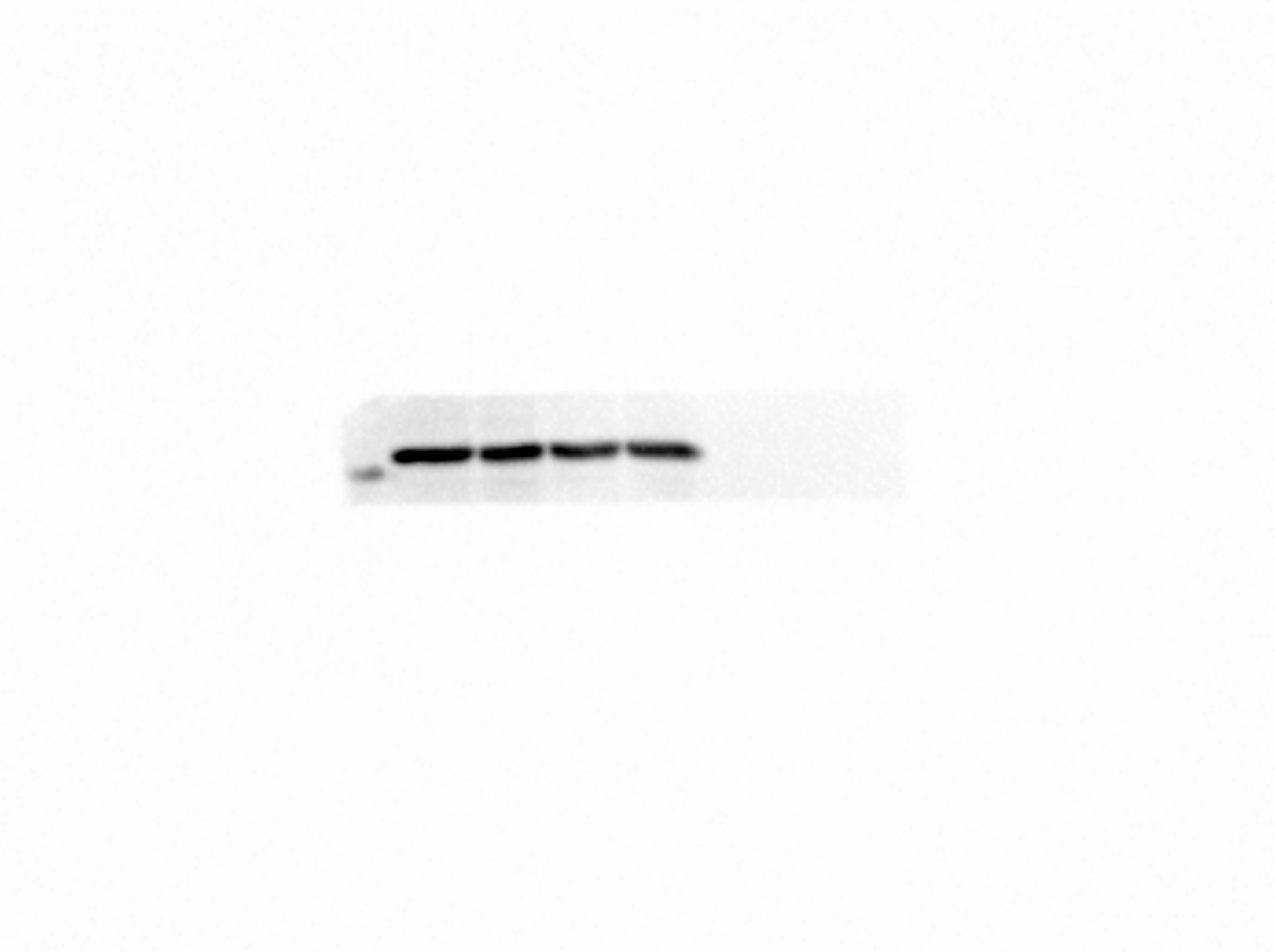

Supplement: Supplementary file 7 — Source data Fig. 3 [file 44318_2026_818_MOESM7_ESM.zip › Figure 3/Figure 3L/GAPDH.tif]

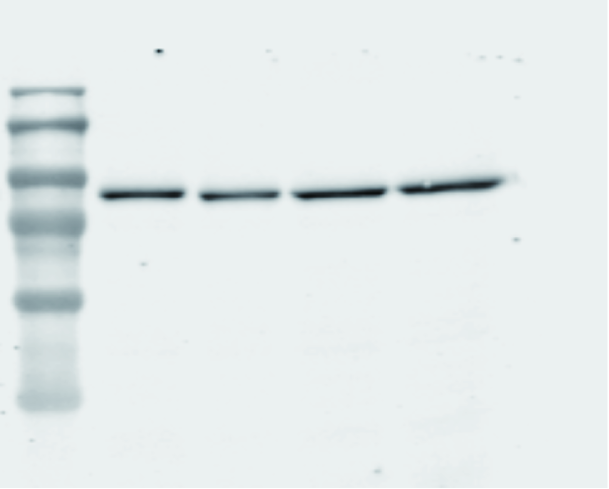

Supplement: Supplementary file 7 — Source data Fig. 3 [file 44318_2026_818_MOESM7_ESM.zip › Figure 3/Figure 3L/HA.tif]

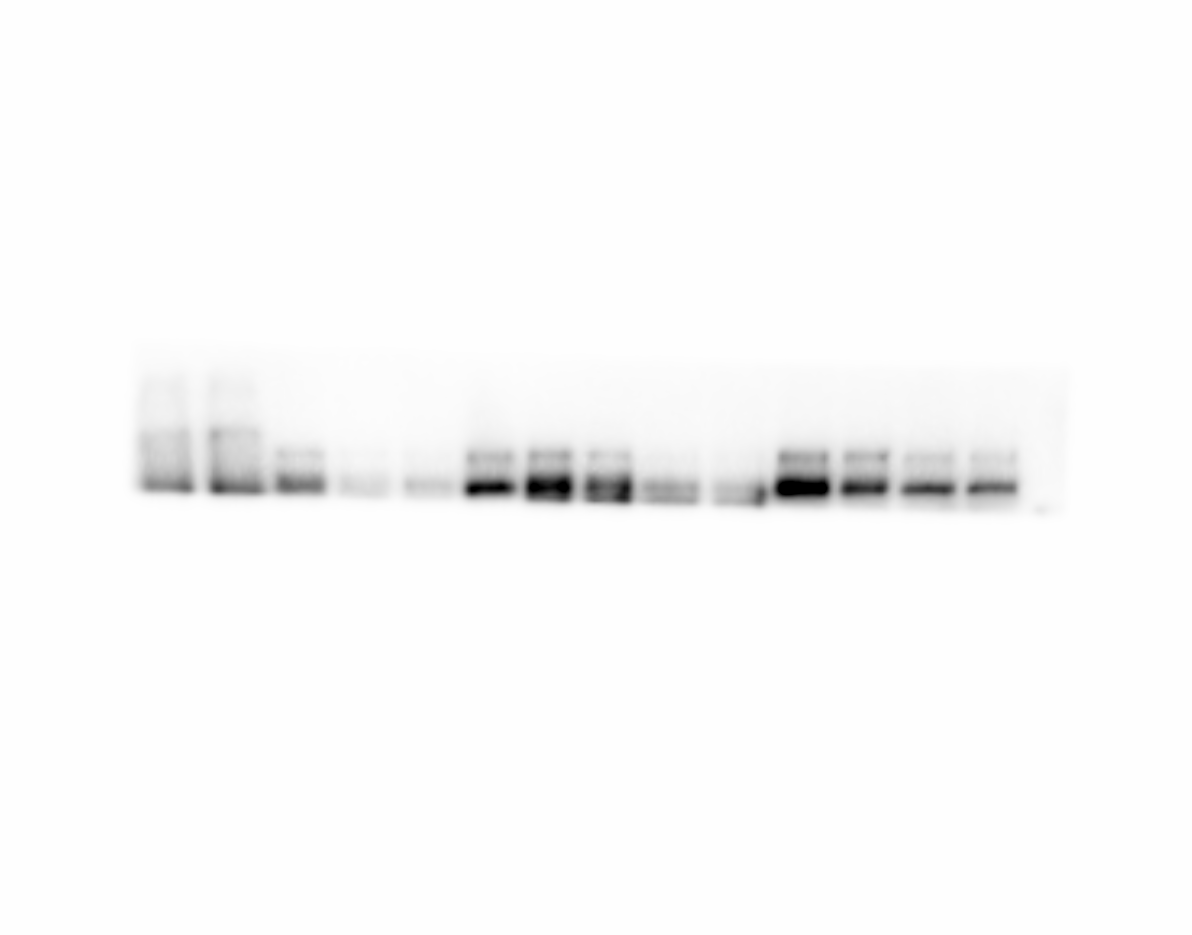

Supplement: Supplementary file 8 — Source data Fig. 4 [file 44318_2026_818_MOESM8_ESM.zip › Figure 4/Figure 4B Blot for Figure 4C and 4D/APP (lane 9-14).tif]

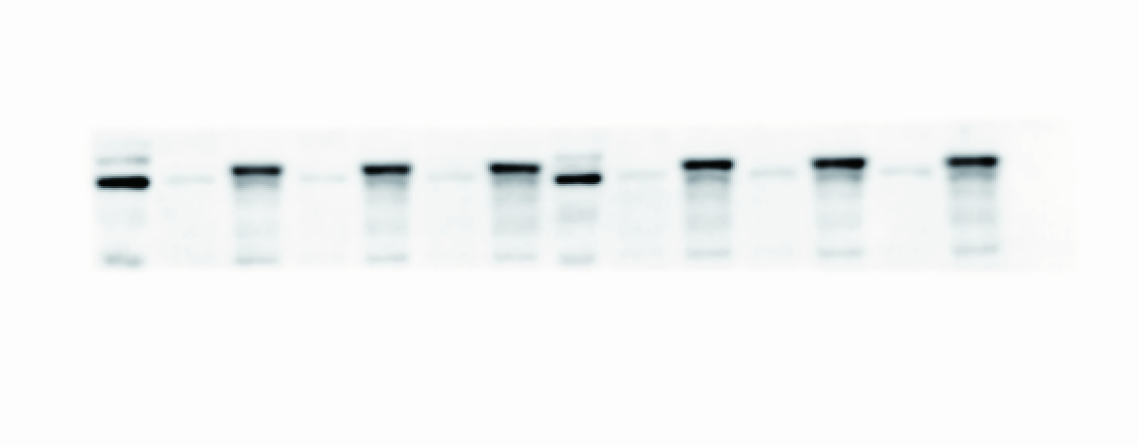

Supplement: Supplementary file 8 — Source data Fig. 4 [file 44318_2026_818_MOESM8_ESM.zip › Figure 4/Figure 4B Blot for Figure 4C and 4D/EGFP-TFE3 (lane 1-6).tif]

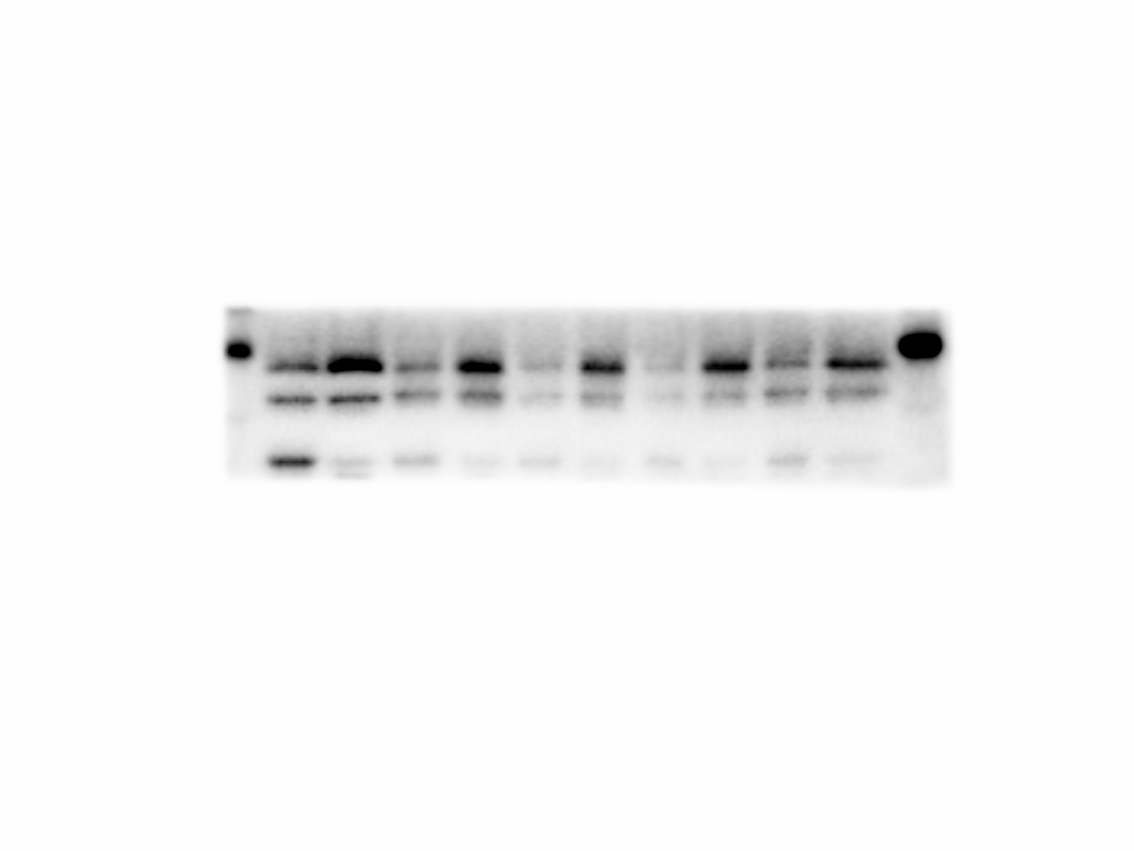

Supplement: Supplementary file 8 — Source data Fig. 4 [file 44318_2026_818_MOESM8_ESM.zip › Figure 4/Figure 4B Blot for Figure 4C and 4D/FAM134B (lane 1-6).tif]

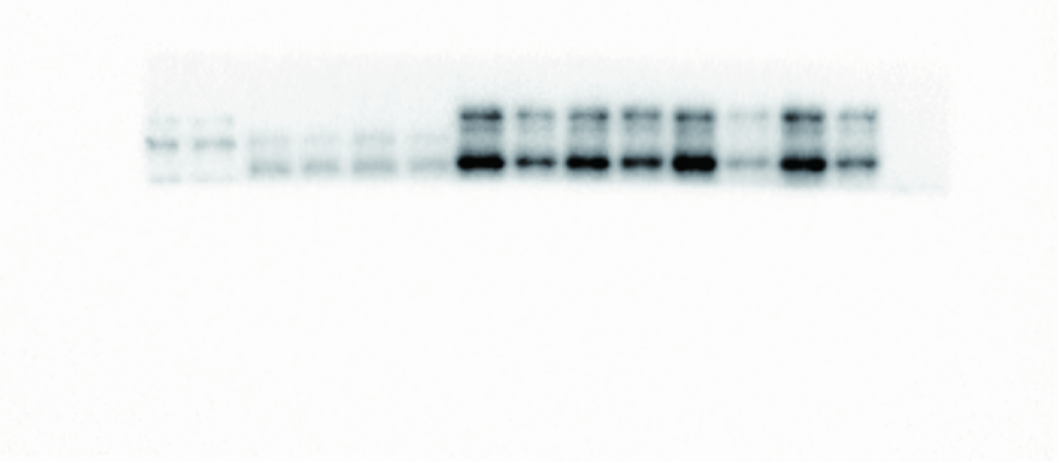

Supplement: Supplementary file 8 — Source data Fig. 4 [file 44318_2026_818_MOESM8_ESM.zip › Figure 4/Figure 4B Blot for Figure 4C and 4D/Figure 4B Replicate 1/APP (lane 7-12).tif]

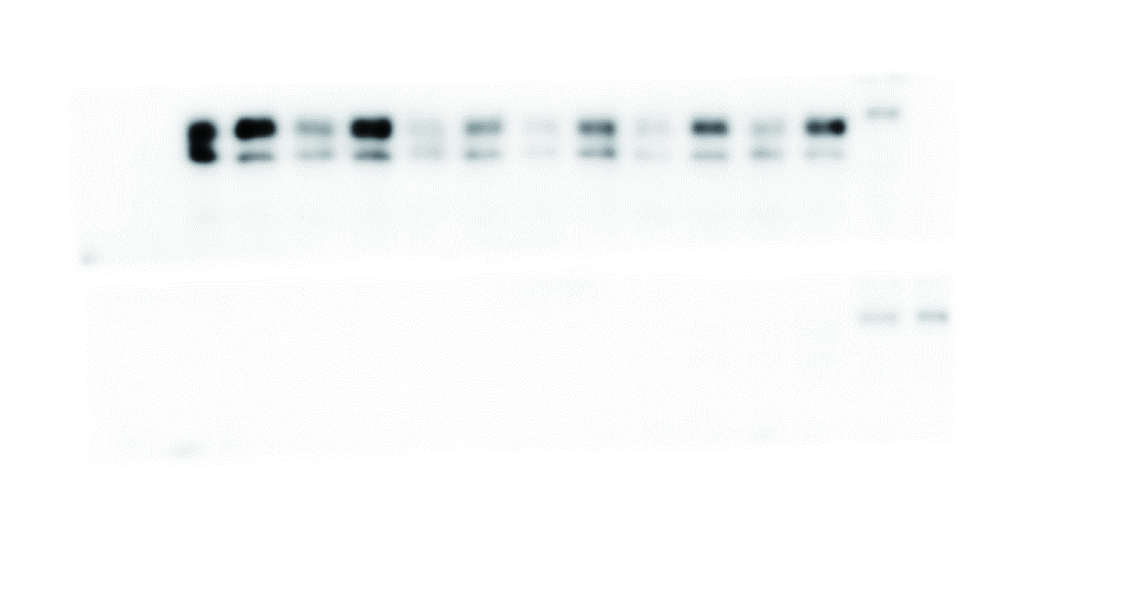

Supplement: Supplementary file 8 — Source data Fig. 4 [file 44318_2026_818_MOESM8_ESM.zip › Figure 4/Figure 4B Blot for Figure 4C and 4D/Figure 4B Replicate 1/FAM134B (lane 1-6).tif]

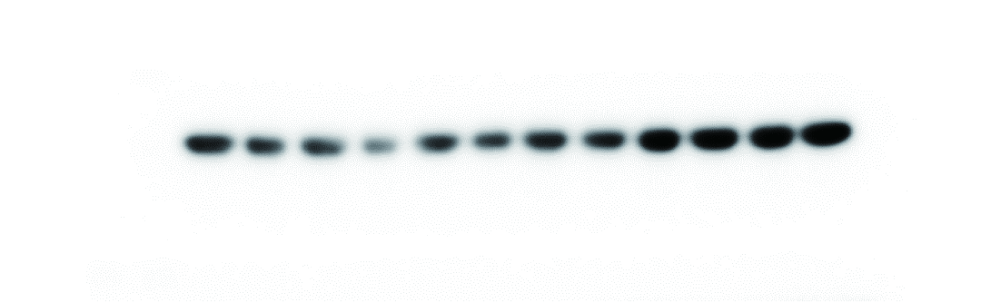

Supplement: Supplementary file 8 — Source data Fig. 4 [file 44318_2026_818_MOESM8_ESM.zip › Figure 4/Figure 4B Blot for Figure 4C and 4D/Figure 4B Replicate 1/GAPDH (lane 1-6).tif]

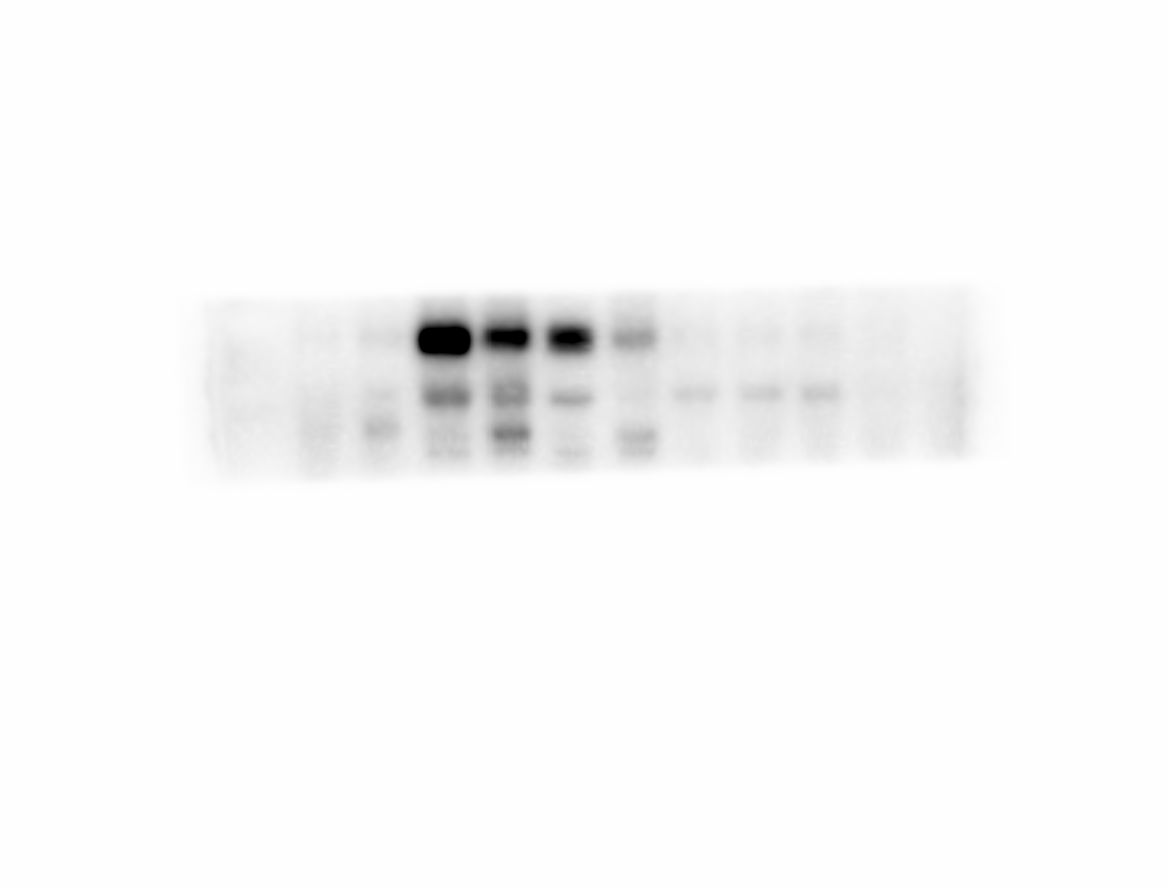

Supplement: Supplementary file 8 — Source data Fig. 4 [file 44318_2026_818_MOESM8_ESM.zip › Figure 4/Figure 4B Blot for Figure 4C and 4D/Figure 4B Replicate 2/APP (up lane 1-6).tif]

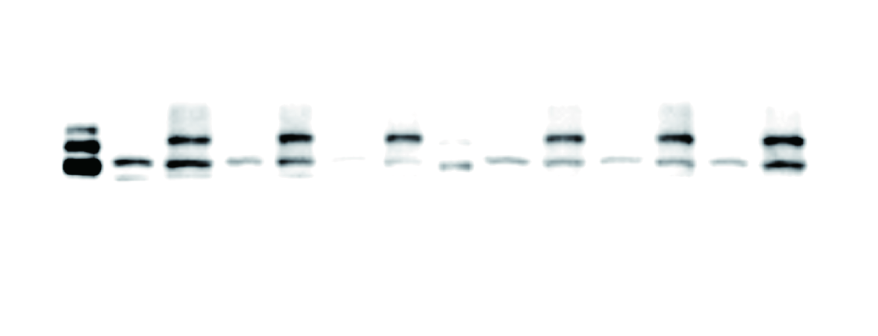

Supplement: Supplementary file 8 — Source data Fig. 4 [file 44318_2026_818_MOESM8_ESM.zip › Figure 4/Figure 4B Blot for Figure 4C and 4D/Figure 4B Replicate 2/EGFP-TFE3 (lane 1-6).tif]

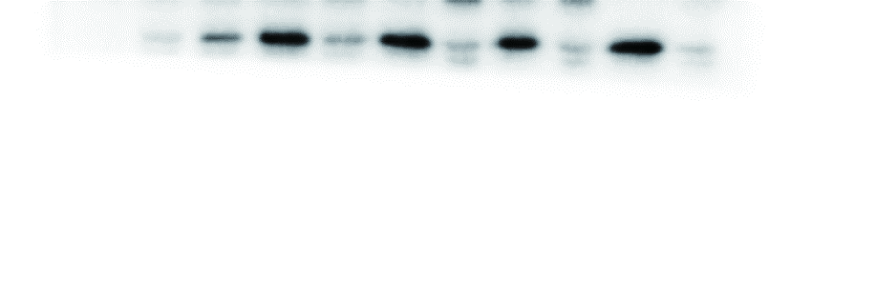

Supplement: Supplementary file 8 — Source data Fig. 4 [file 44318_2026_818_MOESM8_ESM.zip › Figure 4/Figure 4B Blot for Figure 4C and 4D/Figure 4B Replicate 2/FAM134B (lane 1-6).tif]

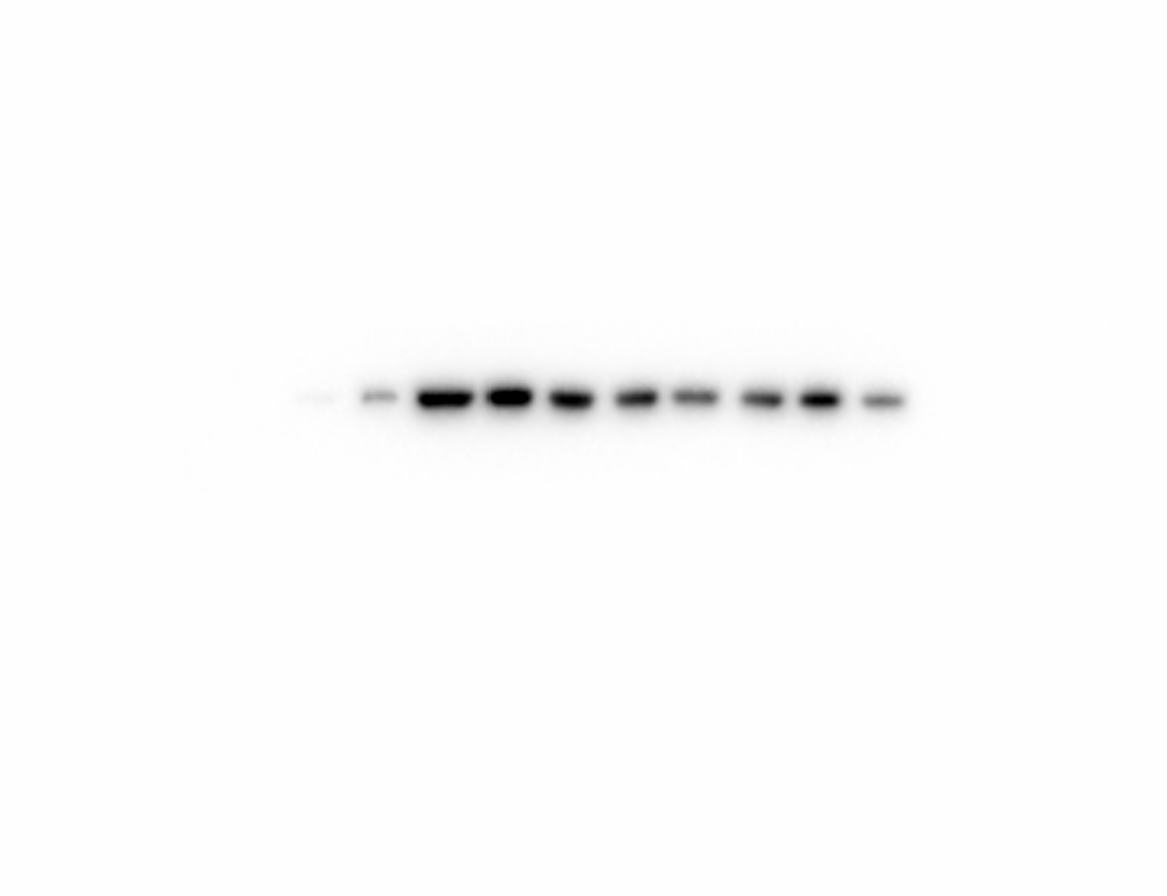

Supplement: Supplementary file 8 — Source data Fig. 4 [file 44318_2026_818_MOESM8_ESM.zip › Figure 4/Figure 4B Blot for Figure 4C and 4D/Figure 4B Replicate 2/GAPDH (lane 1-6).tif]

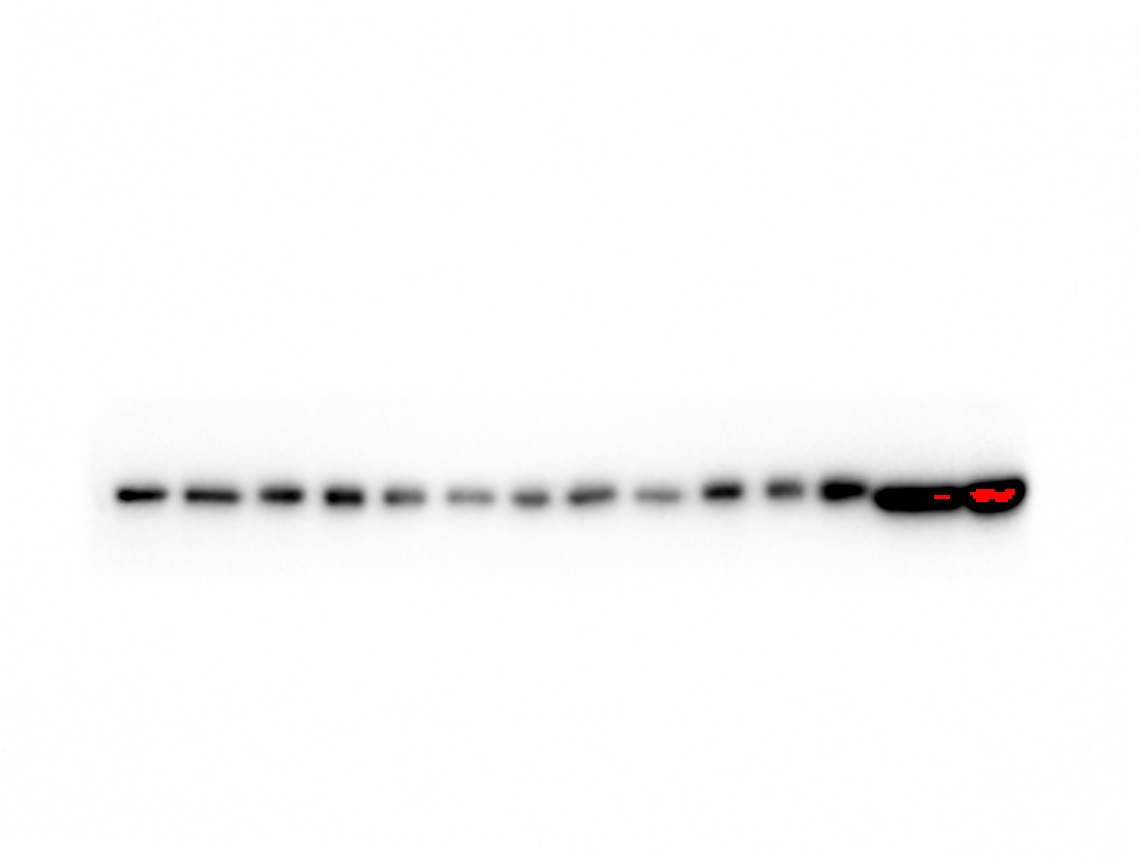

Supplement: Supplementary file 8 — Source data Fig. 4 [file 44318_2026_818_MOESM8_ESM.zip › Figure 4/Figure 4B Blot for Figure 4C and 4D/GAPDH (lane 1-6).tif]

Figure 4B

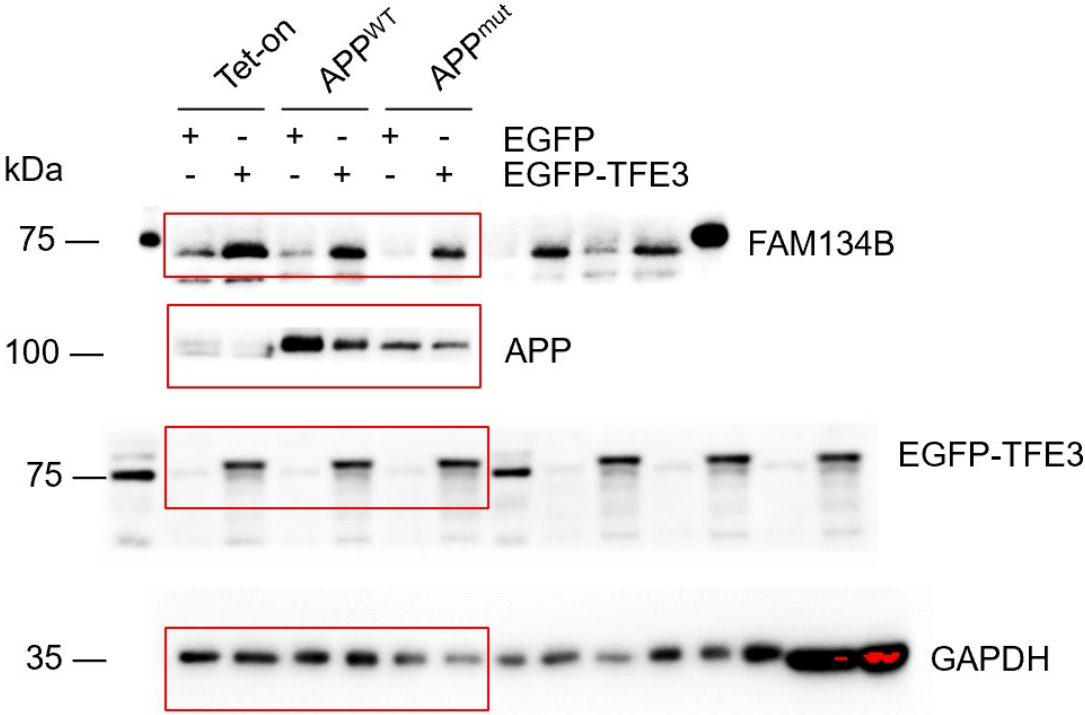

Replicate 1

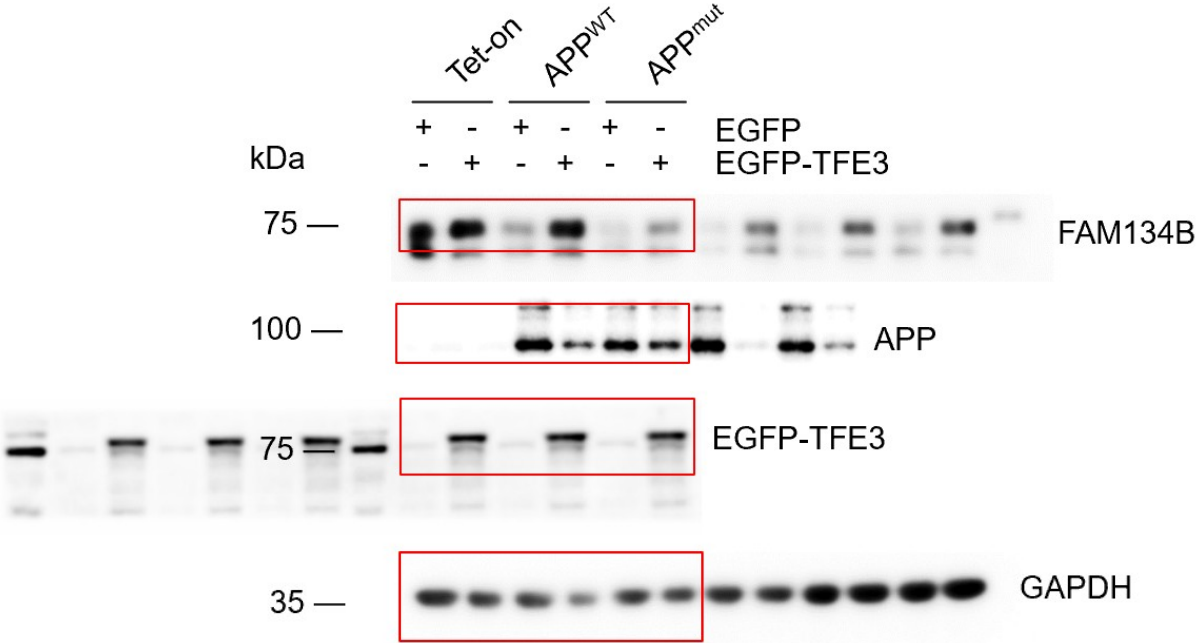

Replicate 2

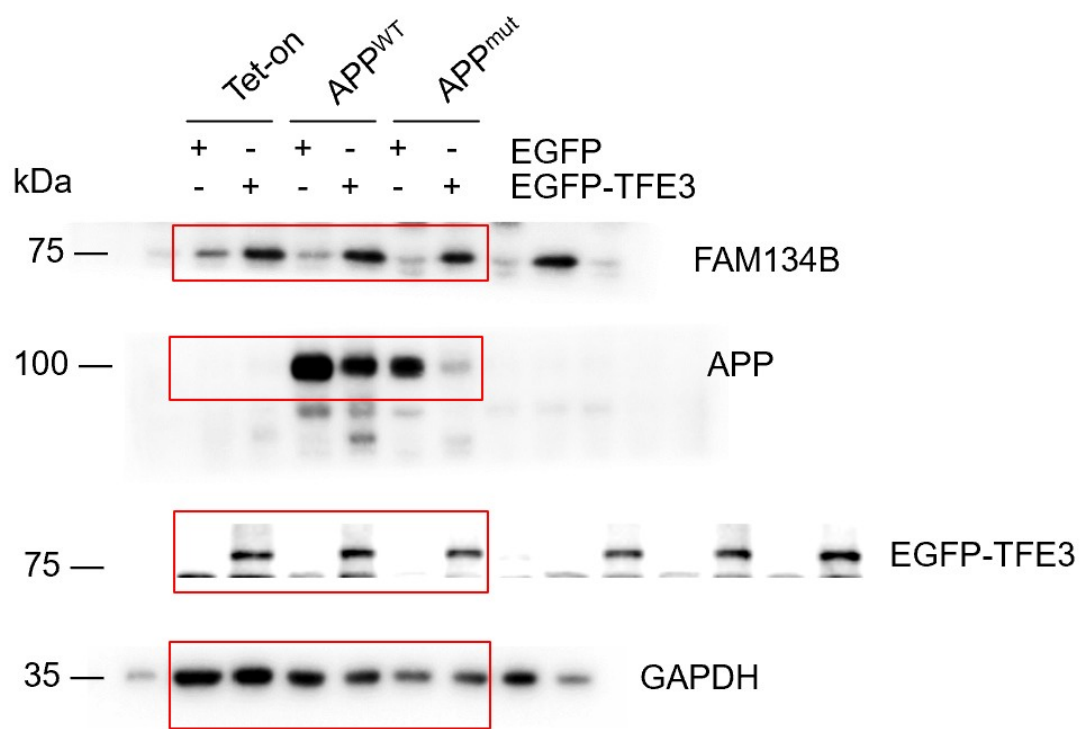

Supplement: Supplementary file 8 — Source data Fig. 4 [file 44318_2026_818_MOESM8_ESM.zip › Figure 4/Figure 4B Blot for Figure 4C and 4D/WB for Figure 4B (Blot for Figure 4C and 4D).pdf]

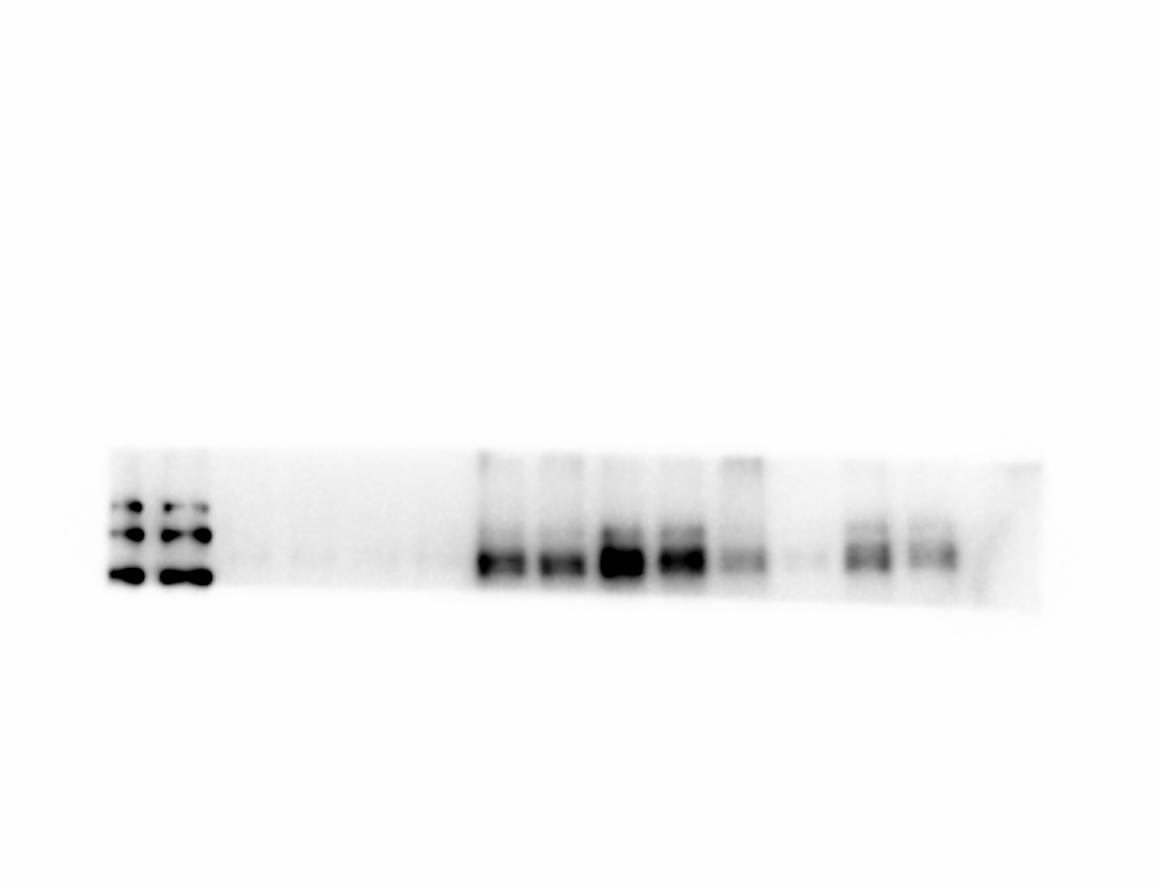

Supplement: Supplementary file 8 — Source data Fig. 4 [file 44318_2026_818_MOESM8_ESM.zip › Figure 4/Figure 4E/APP (lane 1-4).tif]

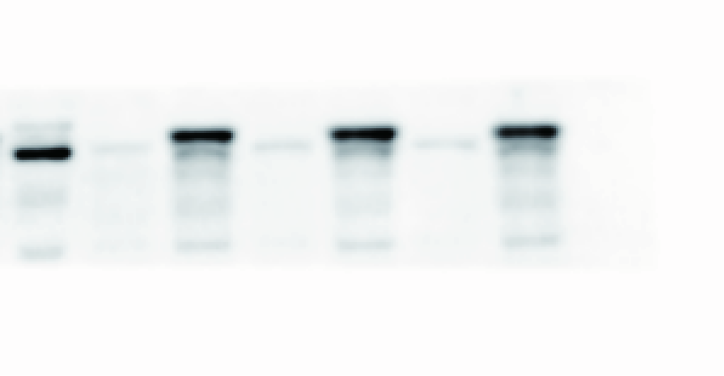

Supplement: Supplementary file 8 — Source data Fig. 4 [file 44318_2026_818_MOESM8_ESM.zip › Figure 4/Figure 4E/EGFP-TFE3 (lane 4-7).tif]

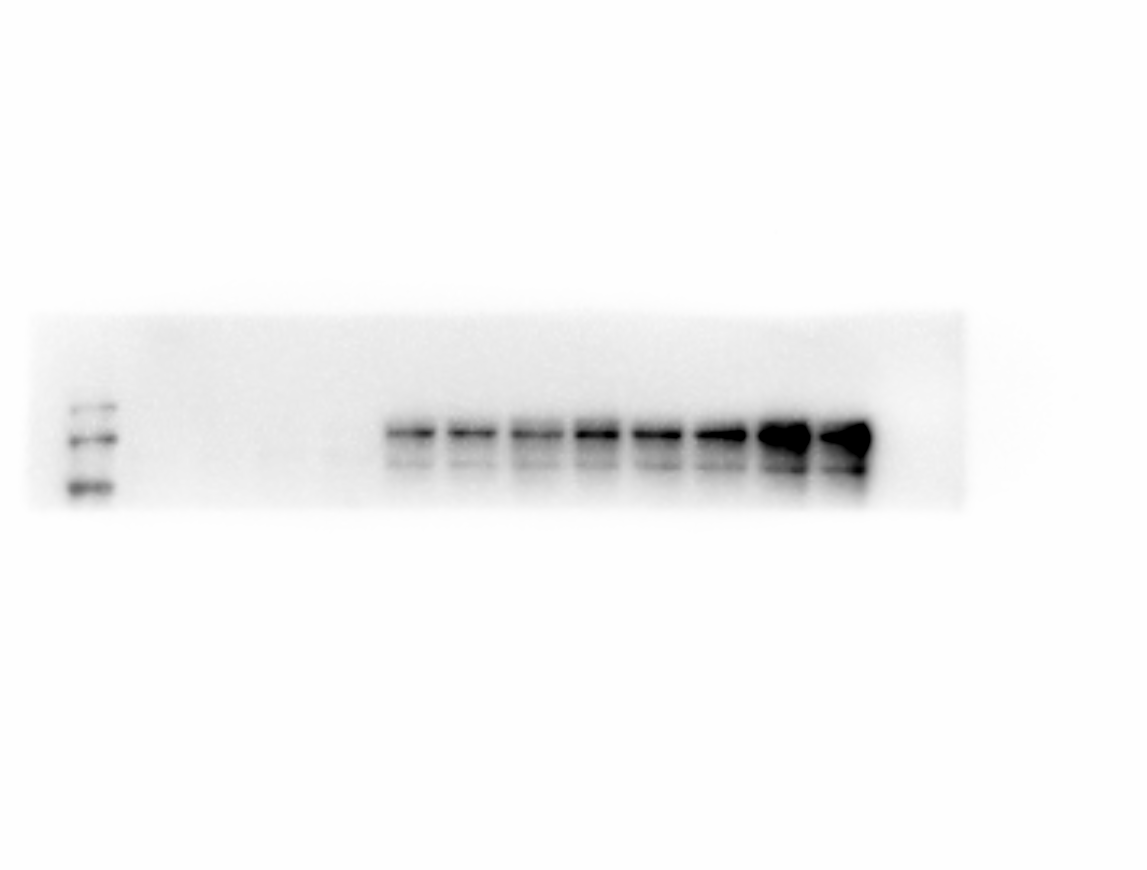

Supplement: Supplementary file 8 — Source data Fig. 4 [file 44318_2026_818_MOESM8_ESM.zip › Figure 4/Figure 4E/Figure 4E Replicate 1/APP (lane 1-4).tif]

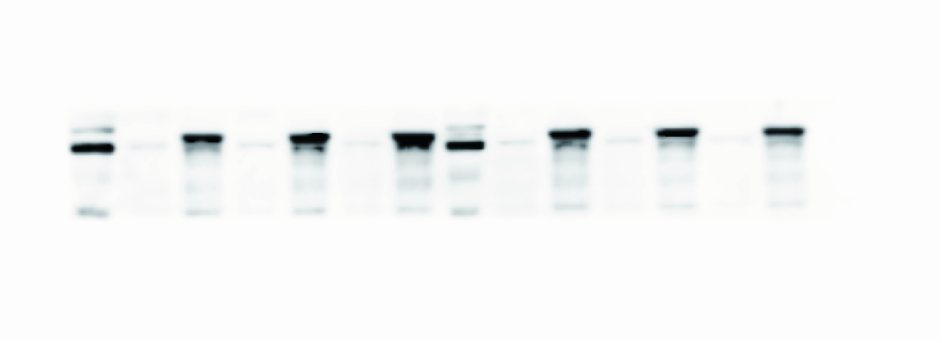

Supplement: Supplementary file 8 — Source data Fig. 4 [file 44318_2026_818_MOESM8_ESM.zip › Figure 4/Figure 4E/Figure 4E Replicate 1/EGFP-TFE3 (lane 4-7).tif]

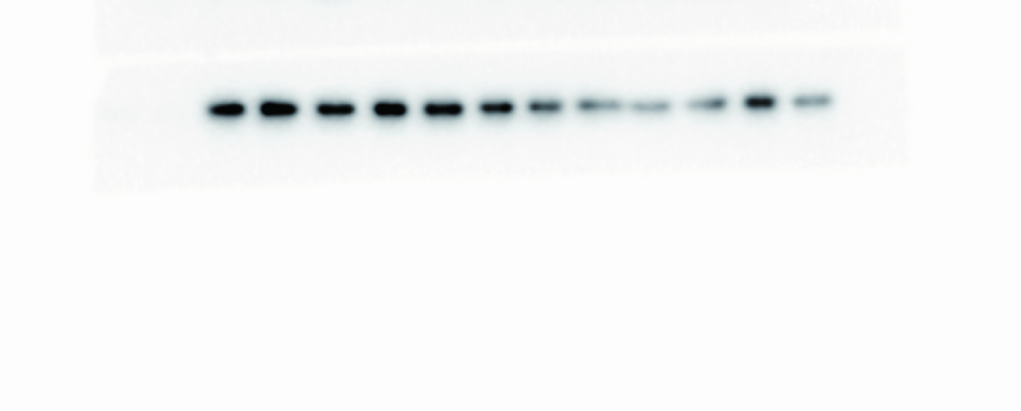

Supplement: Supplementary file 8 — Source data Fig. 4 [file 44318_2026_818_MOESM8_ESM.zip › Figure 4/Figure 4E/Figure 4E Replicate 1/GAPDH (lane 4-7).tif]

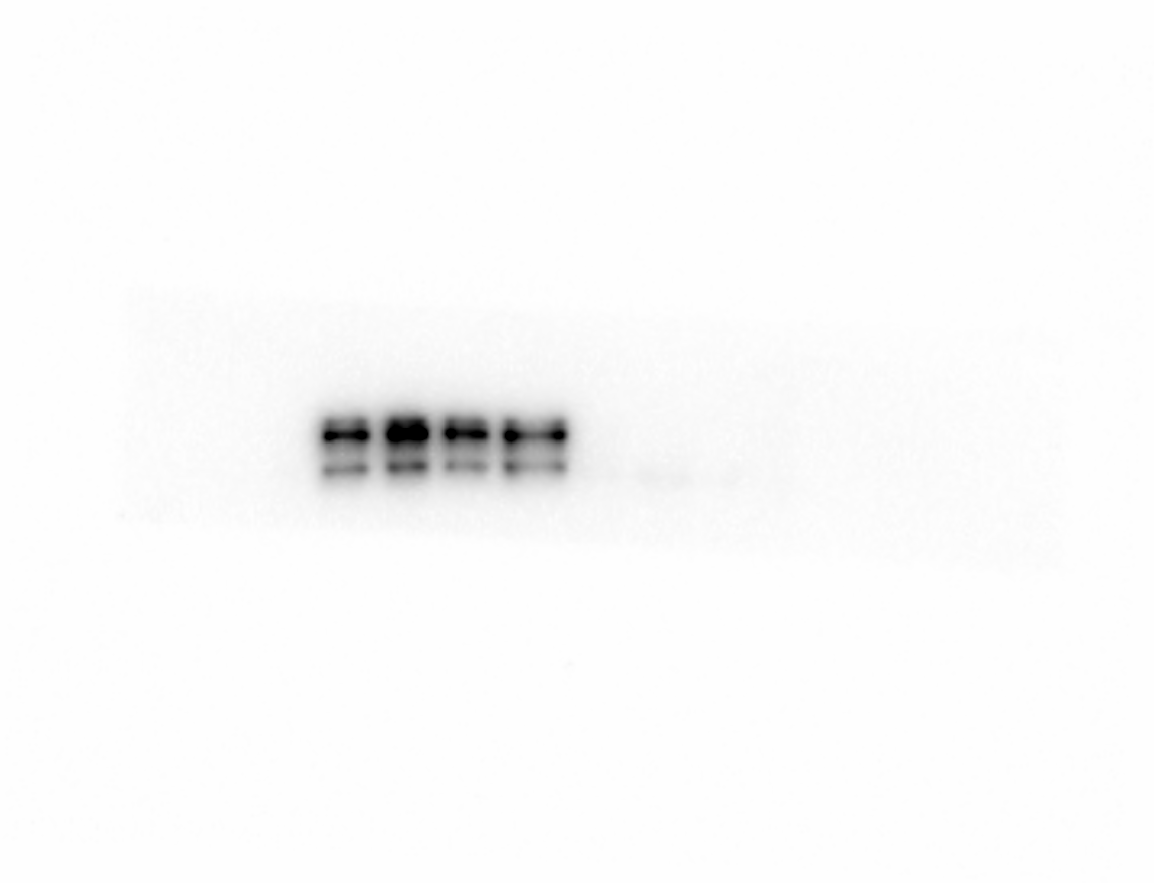

Supplement: Supplementary file 8 — Source data Fig. 4 [file 44318_2026_818_MOESM8_ESM.zip › Figure 4/Figure 4E/Figure 4E Replicate 2/APP.tif]

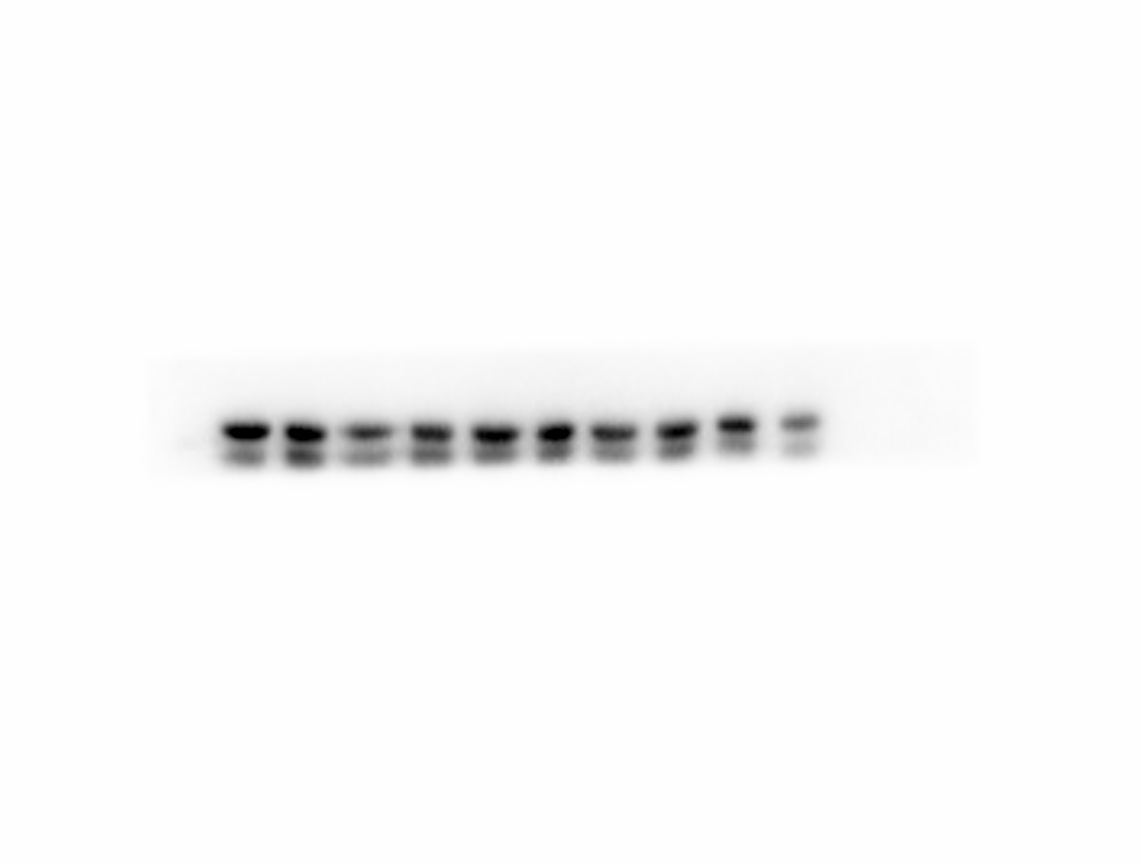

Supplement: Supplementary file 8 — Source data Fig. 4 [file 44318_2026_818_MOESM8_ESM.zip › Figure 4/Figure 4E/Figure 4E Replicate 2/GAPDH (lane 5-8).tif]

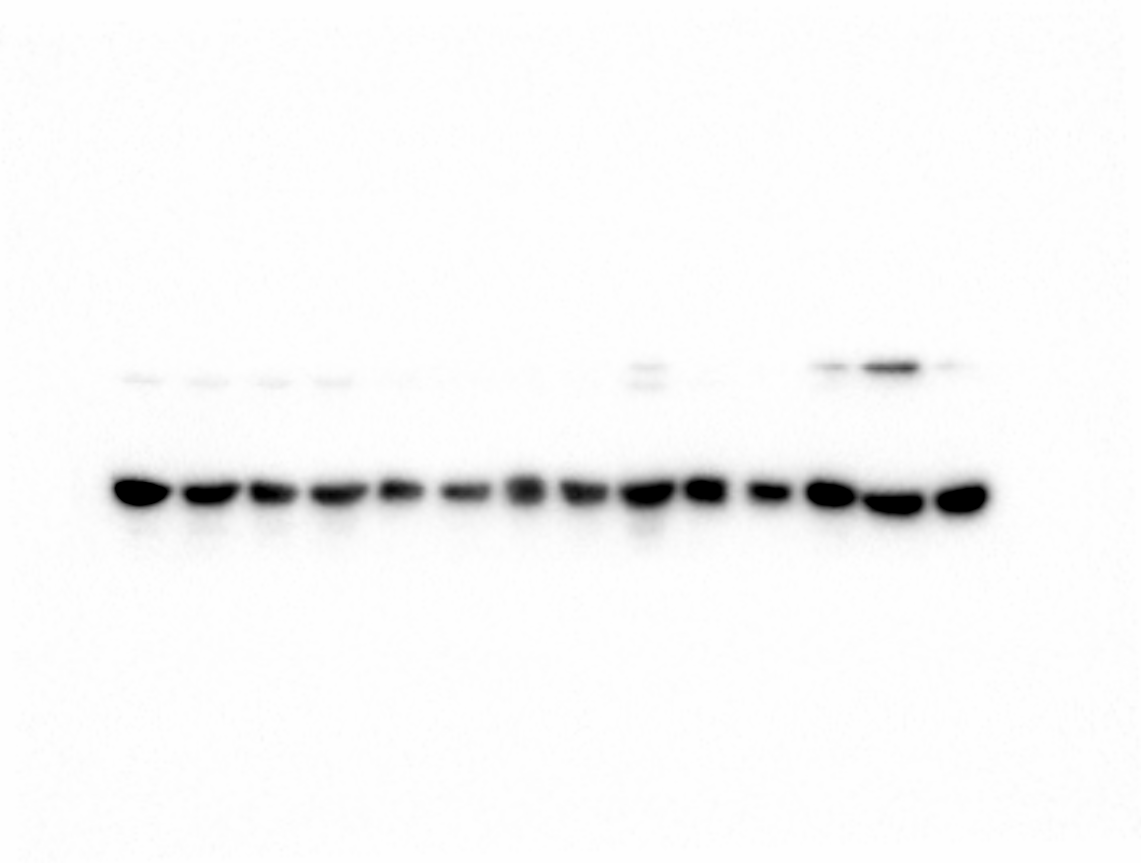

Supplement: Supplementary file 8 — Source data Fig. 4 [file 44318_2026_818_MOESM8_ESM.zip › Figure 4/Figure 4E/GAPDH (lane 5-8).tif]

Figure 4E

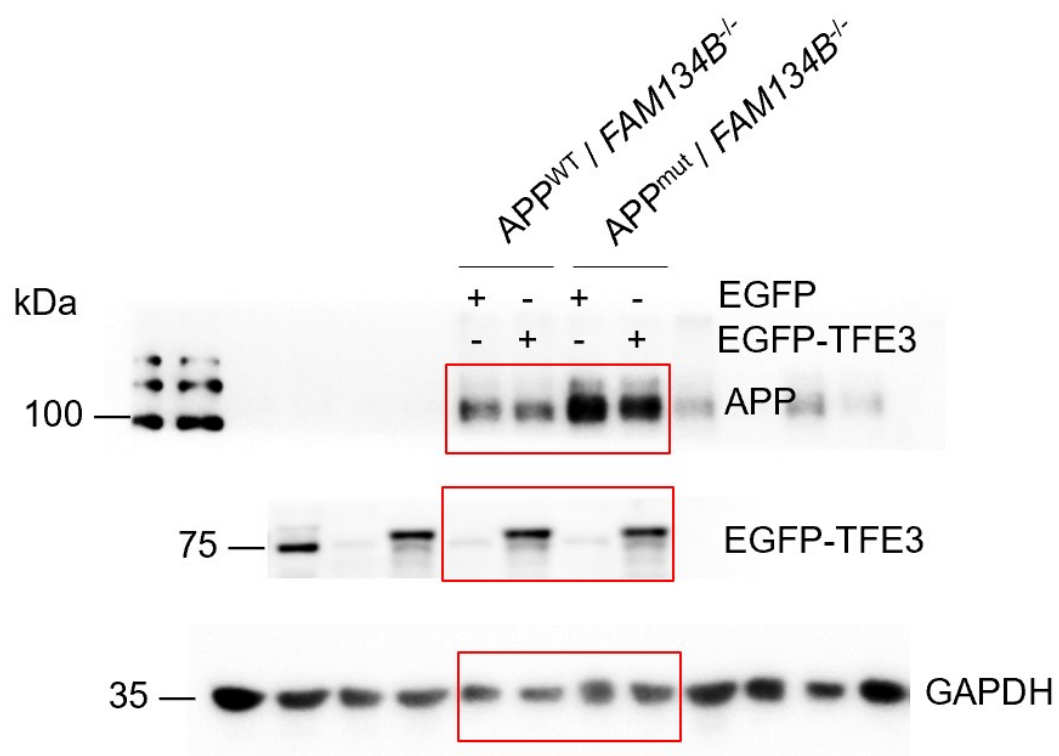

## Replicate 1

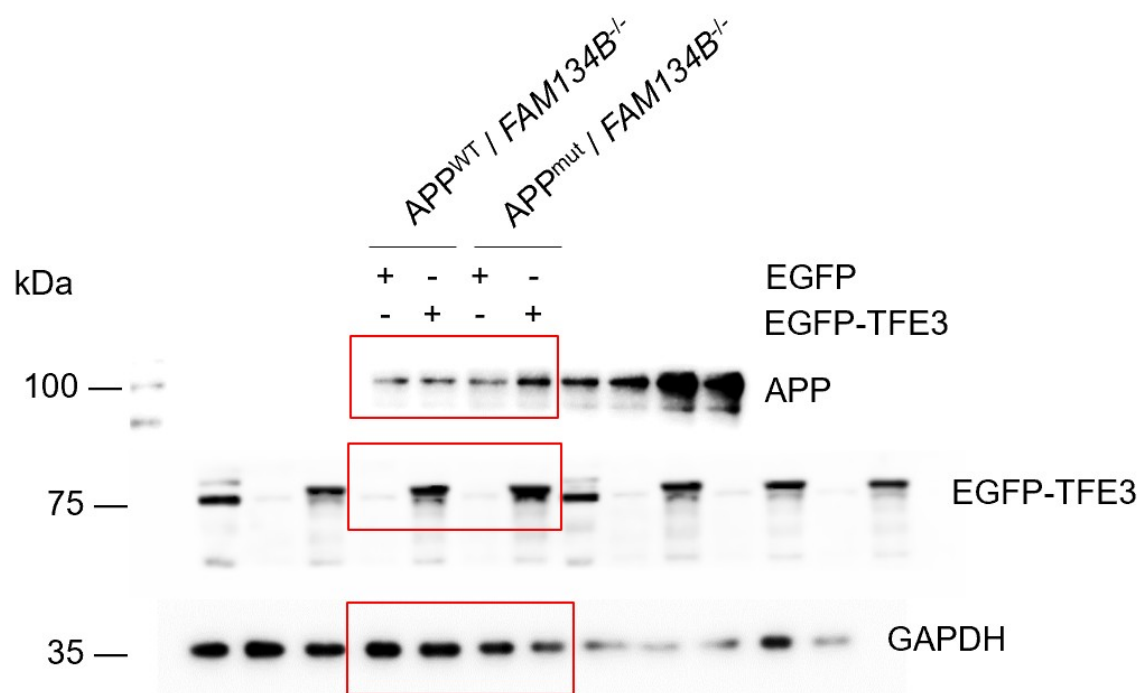

## Replicate 2

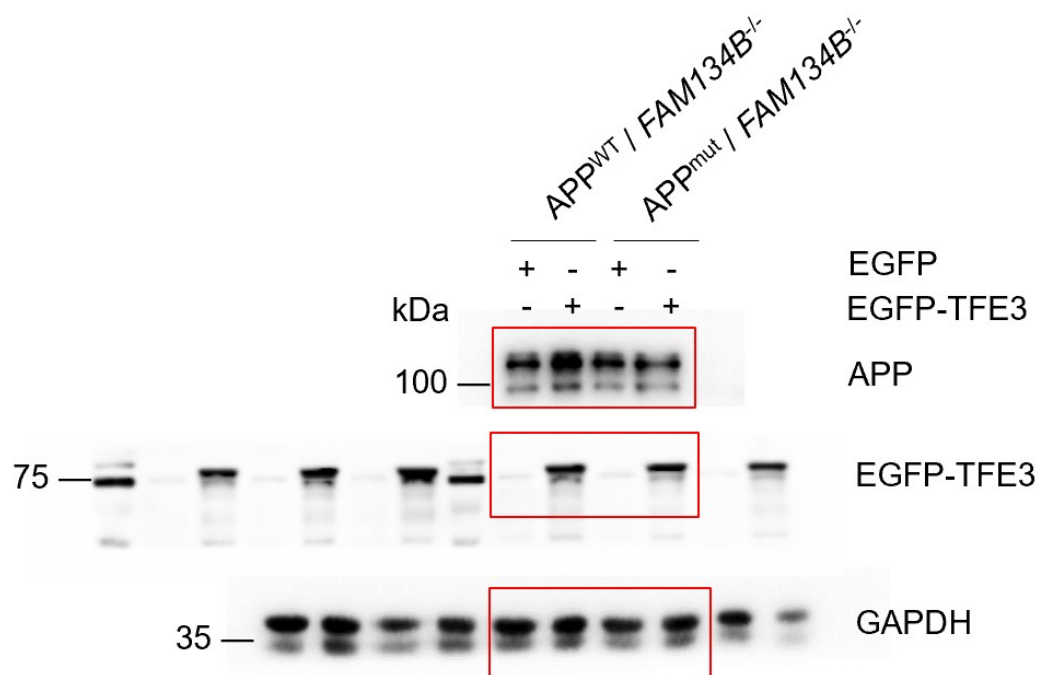

Supplement: Supplementary file 8 — Source data Fig. 4 [file 44318_2026_818_MOESM8_ESM.zip › Figure 4/Figure 4E/WB for Figure 4E.pdf]

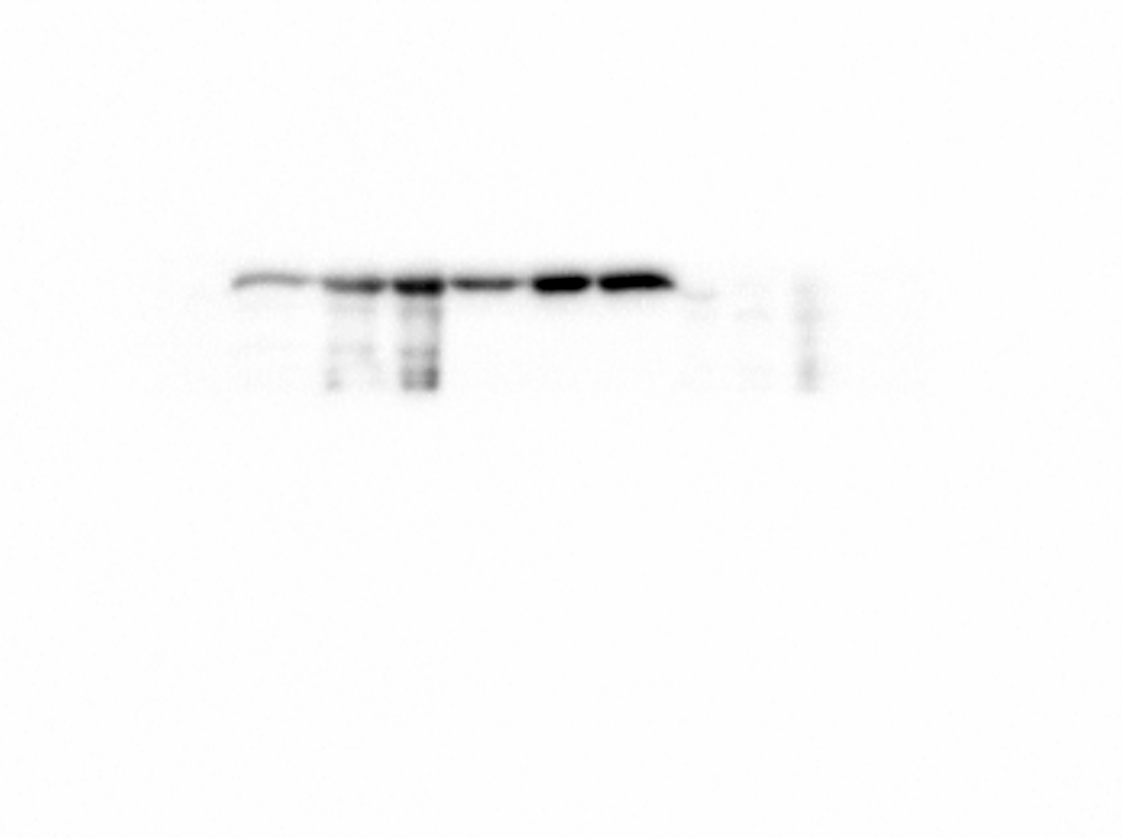

Supplement: Supplementary file 8 — Source data Fig. 4 [file 44318_2026_818_MOESM8_ESM.zip › Figure 4/Figure 4G/Figure 4G Replicate 1/GAPDH.tif]

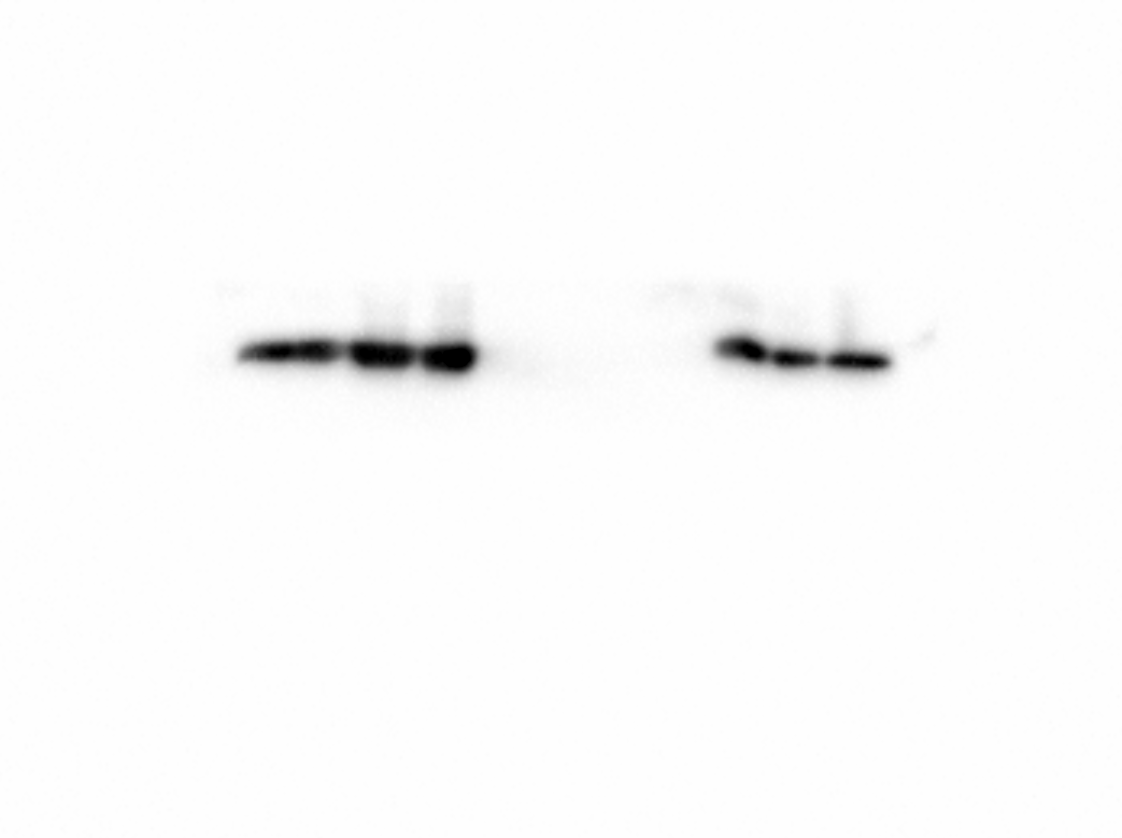

Supplement: Supplementary file 8 — Source data Fig. 4 [file 44318_2026_818_MOESM8_ESM.zip › Figure 4/Figure 4G/Figure 4G Replicate 1/H3.tif]

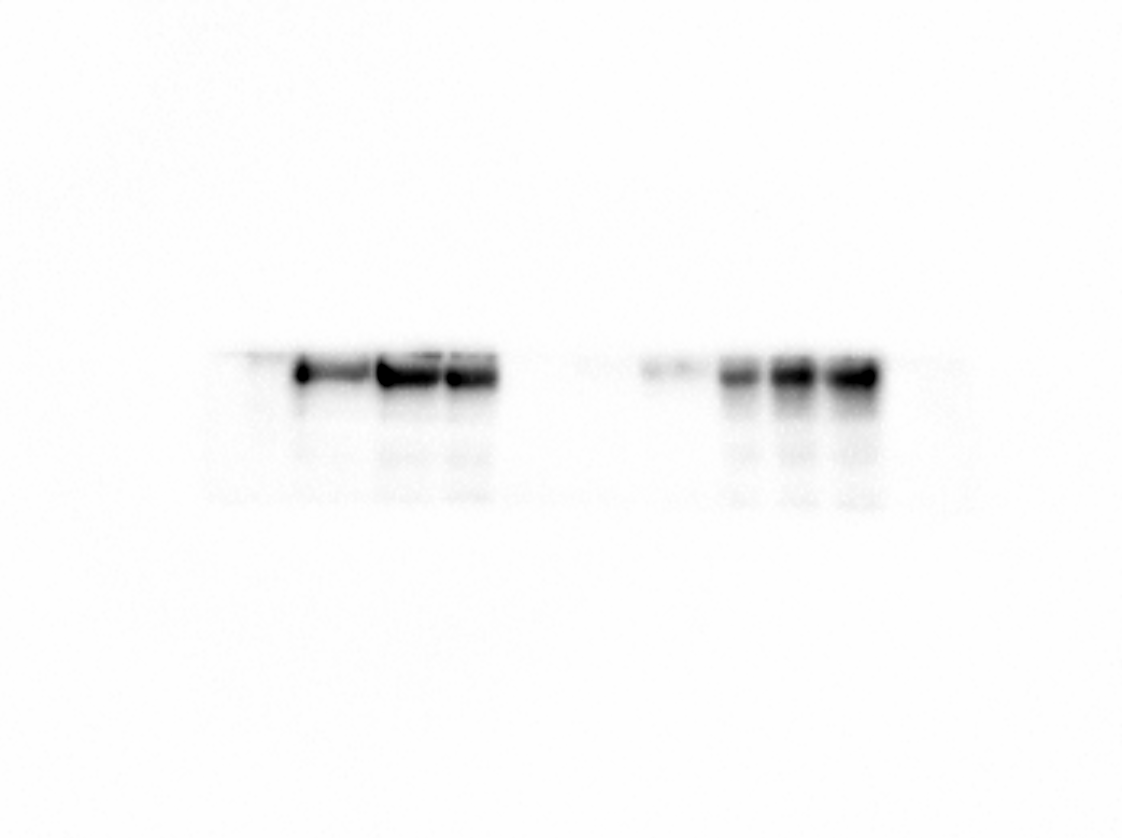

Supplement: Supplementary file 8 — Source data Fig. 4 [file 44318_2026_818_MOESM8_ESM.zip › Figure 4/Figure 4G/Figure 4G Replicate 1/TFE3.tif]

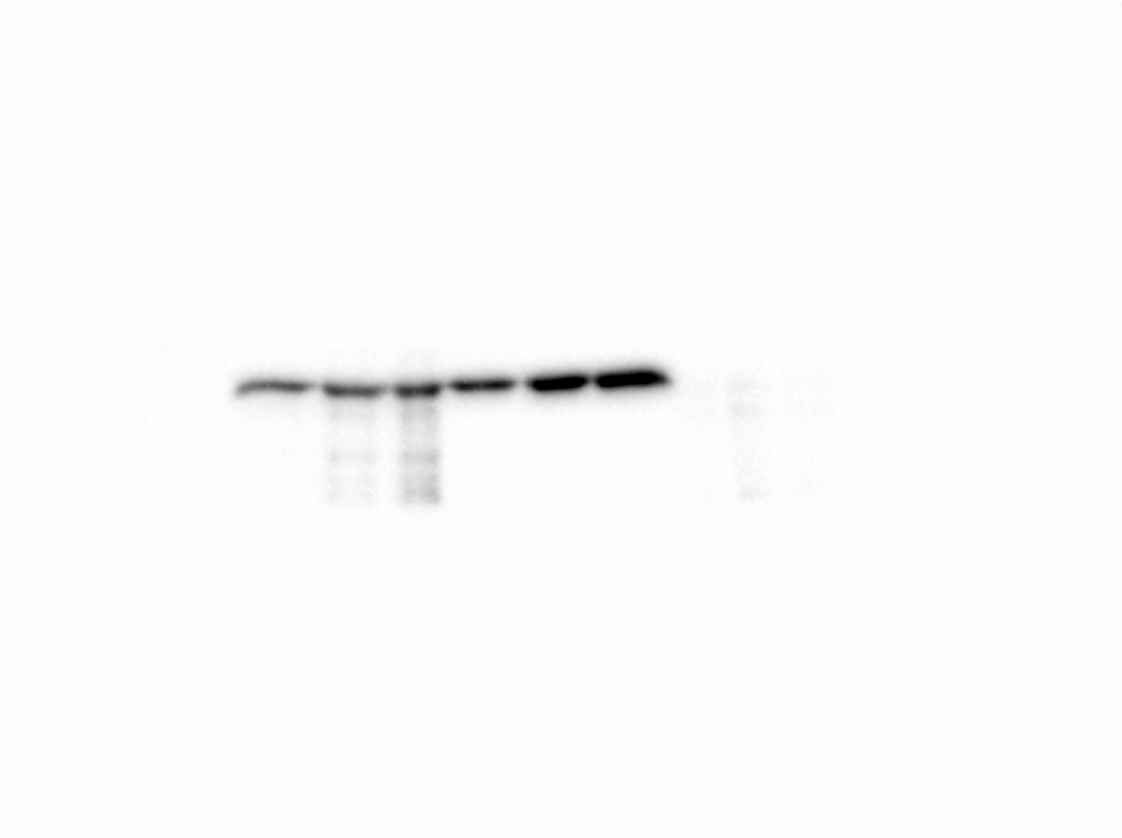

Supplement: Supplementary file 8 — Source data Fig. 4 [file 44318_2026_818_MOESM8_ESM.zip › Figure 4/Figure 4G/Figure 4G Replicate 2/GAPDH.tif]

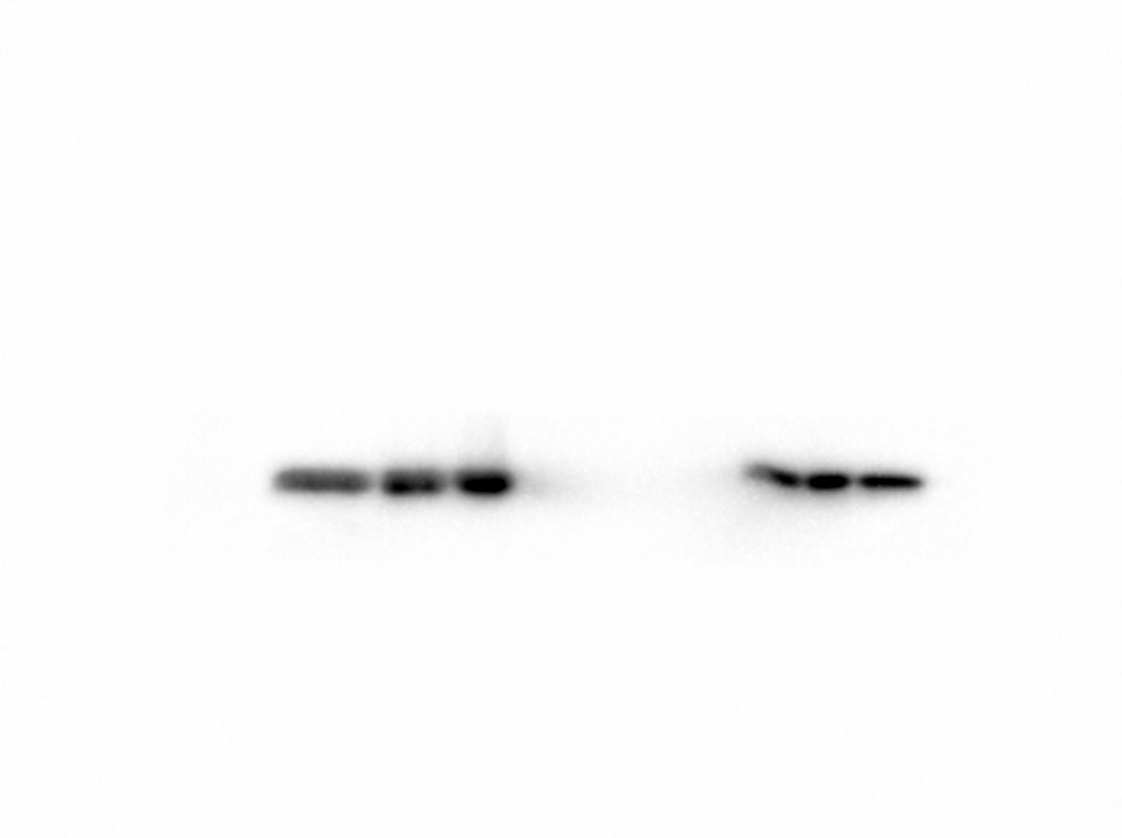

Supplement: Supplementary file 8 — Source data Fig. 4 [file 44318_2026_818_MOESM8_ESM.zip › Figure 4/Figure 4G/Figure 4G Replicate 2/H3.tif]

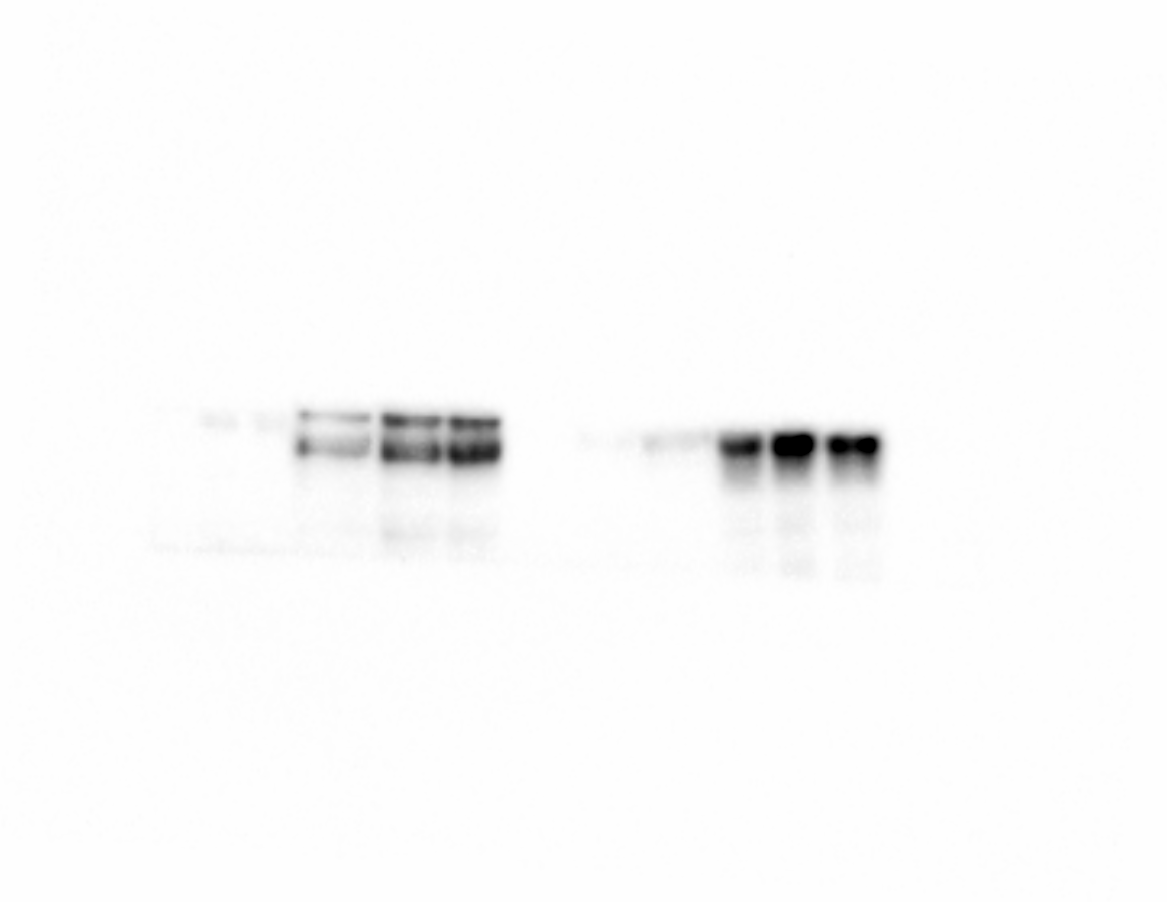

Supplement: Supplementary file 8 — Source data Fig. 4 [file 44318_2026_818_MOESM8_ESM.zip › Figure 4/Figure 4G/Figure 4G Replicate 2/TFE3.tif]
