# Supplementary figures and images for: FAM134B-mediated ER-phagy degrades APP and suppresses Alzheimer’s disease pathology (part 3 of 3)
Source: EMBO J. 2026 May 26;45(13):4492–530. doi: 10.1038/s44318-026-00818-9 (PMC13324857; doi:10.1038/s44318-026-00818-9)

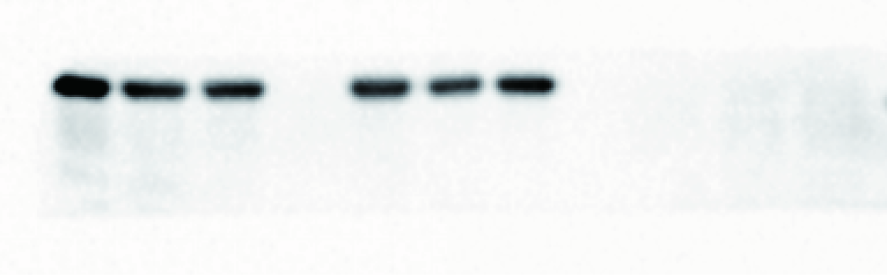

Supplement: Supplementary file 8 — Source data Fig. 4 [file 44318_2026_818_MOESM8_ESM.zip › Figure 4/Figure 4G/GAPDH.tif]

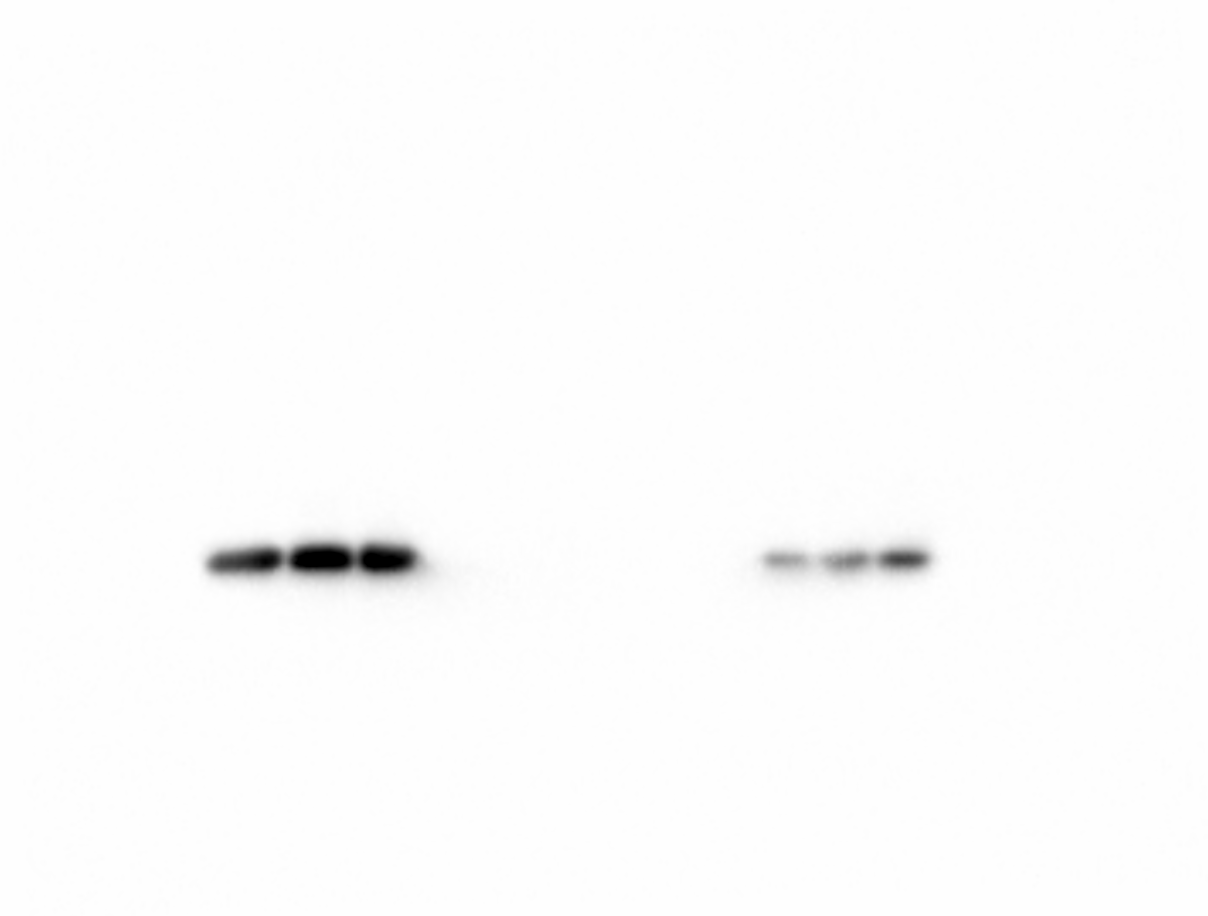

Supplement: Supplementary file 8 — Source data Fig. 4 [file 44318_2026_818_MOESM8_ESM.zip › Figure 4/Figure 4G/H3.tif]

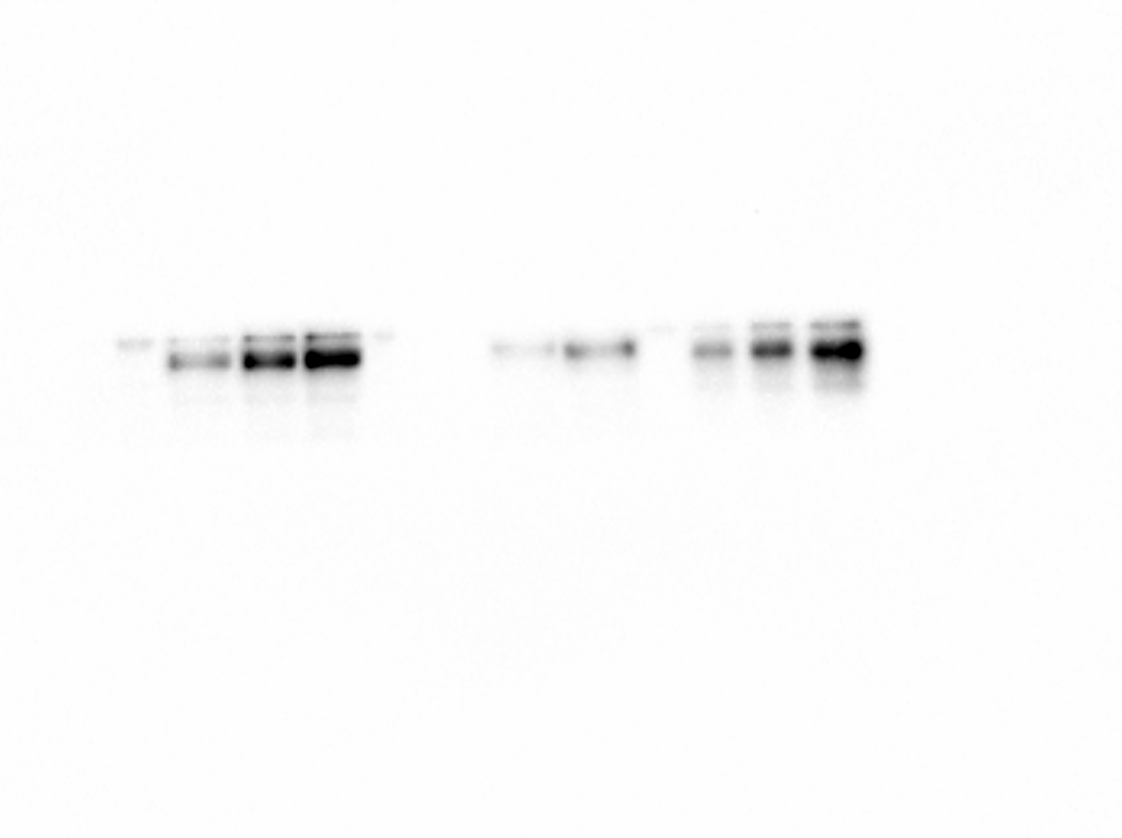

Supplement: Supplementary file 8 — Source data Fig. 4 [file 44318_2026_818_MOESM8_ESM.zip › Figure 4/Figure 4G/TFE3.tif]

Figure 4G

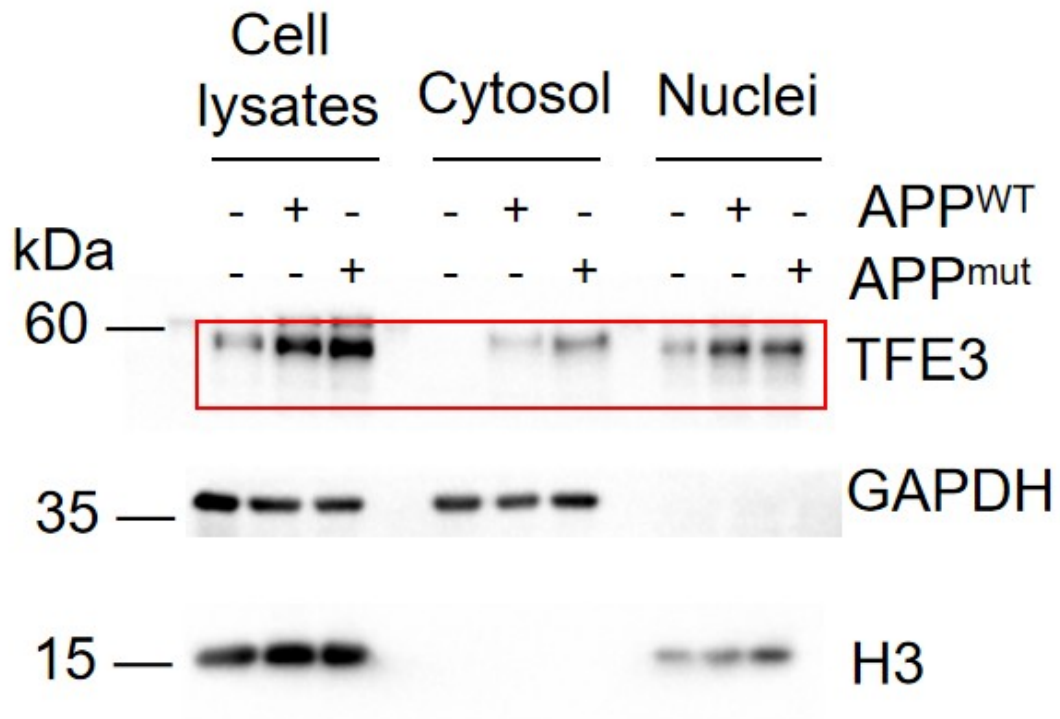

## Replicate 1

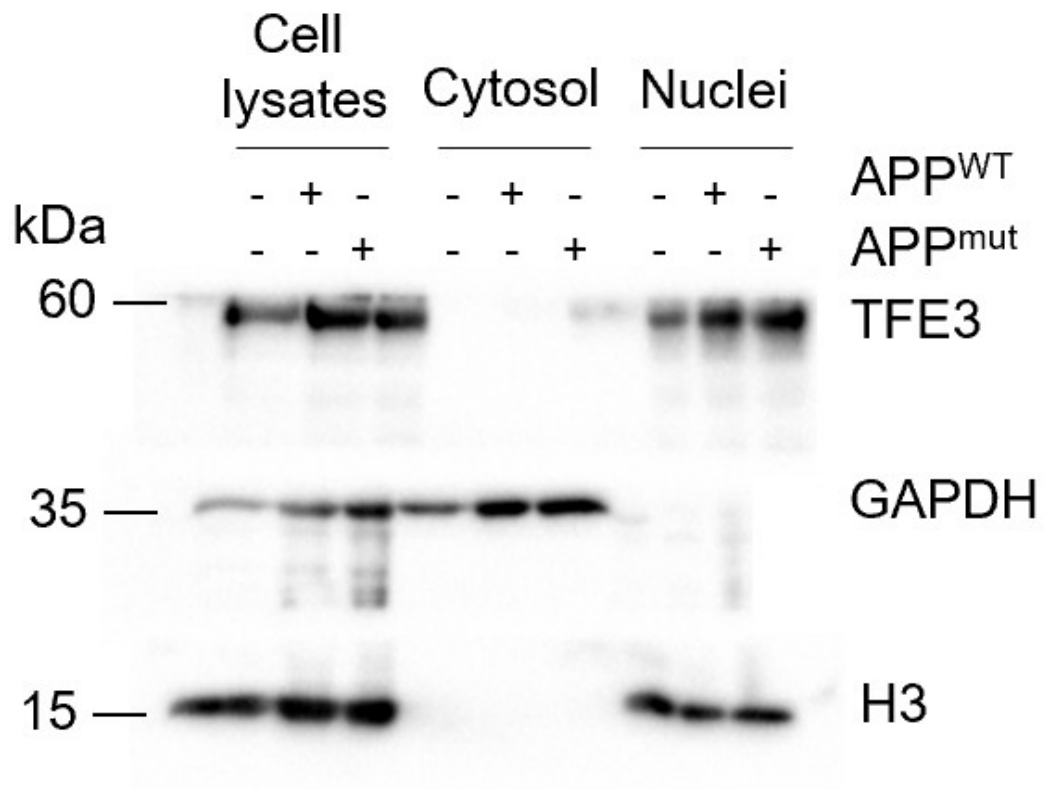

# Replicate 2

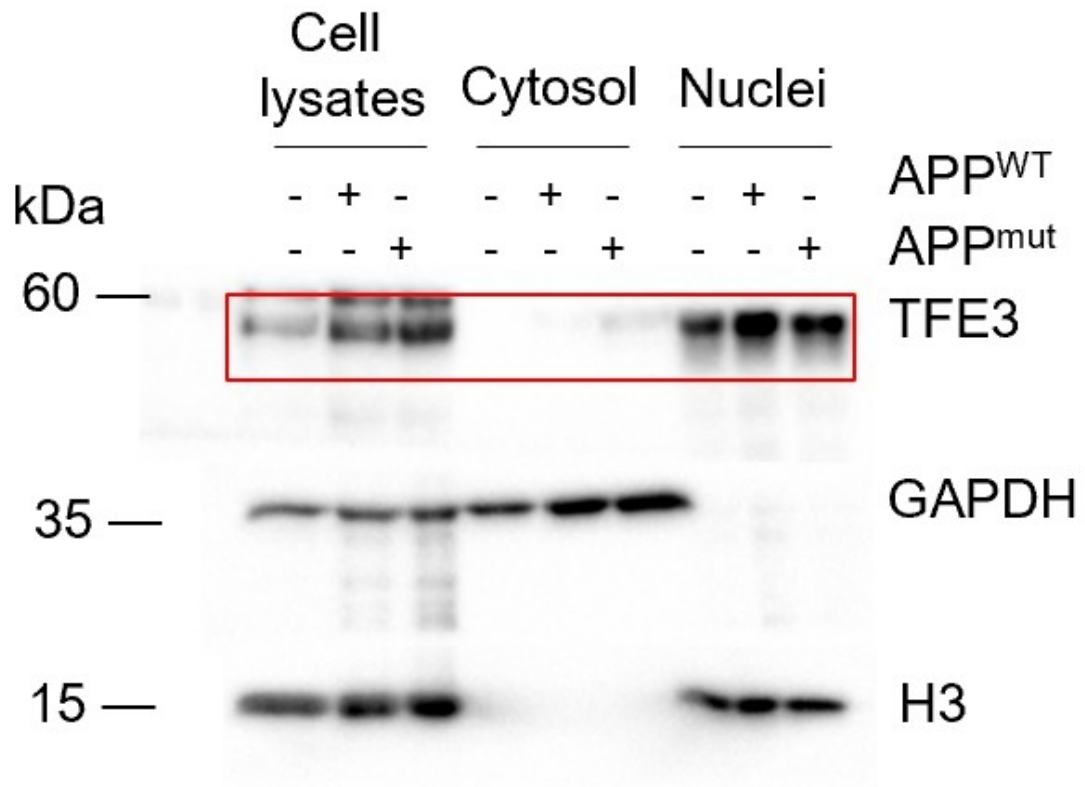

Supplement: Supplementary file 8 — Source data Fig. 4 [file 44318_2026_818_MOESM8_ESM.zip › Figure 4/Figure 4G/WB for Figure 4G.pdf]

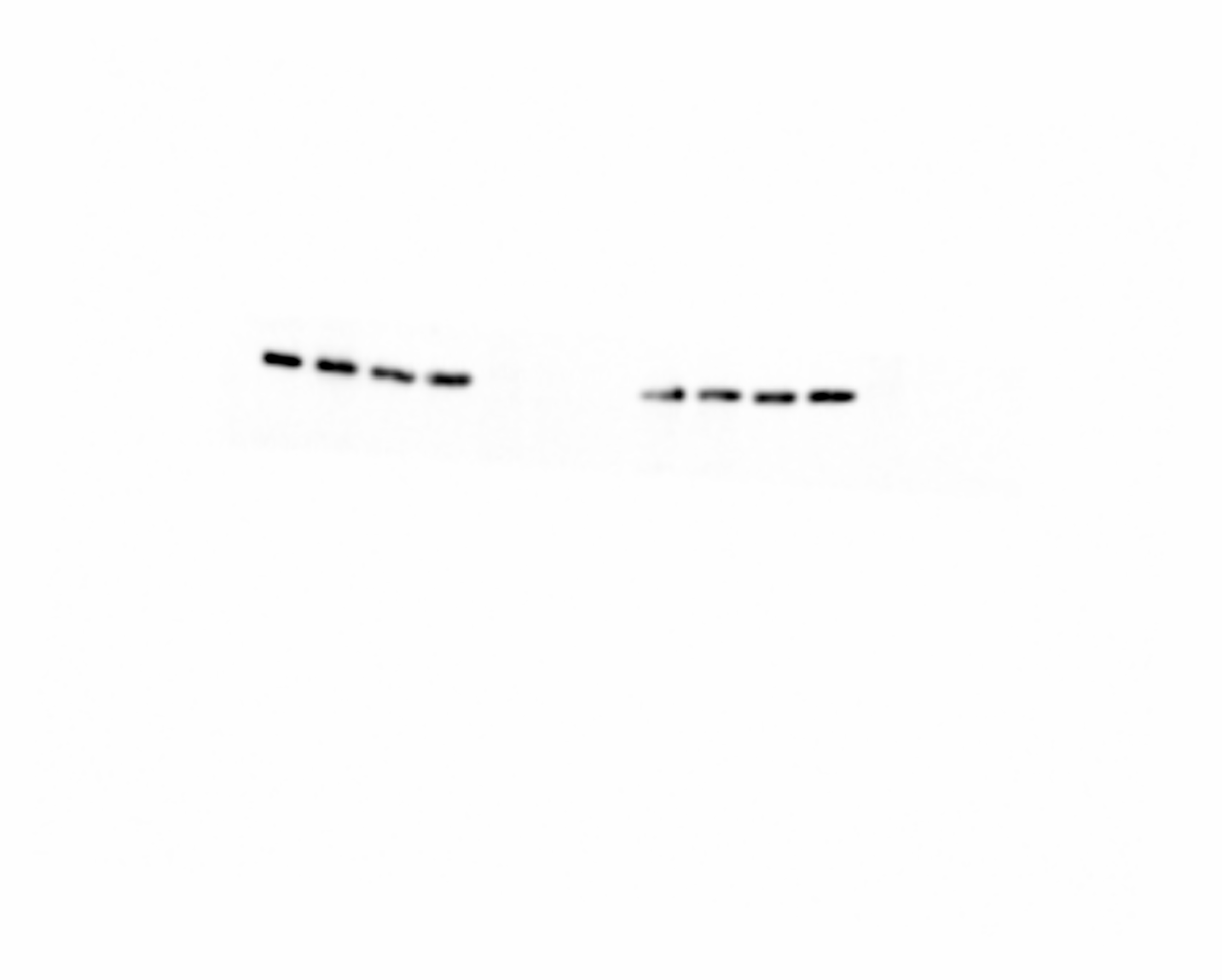

Supplement: Supplementary file 8 — Source data Fig. 4 [file 44318_2026_818_MOESM8_ESM.zip › Figure 4/Figure 4I/Figure 4I Replicate 1/GAPDH (lane 1-6).tif]

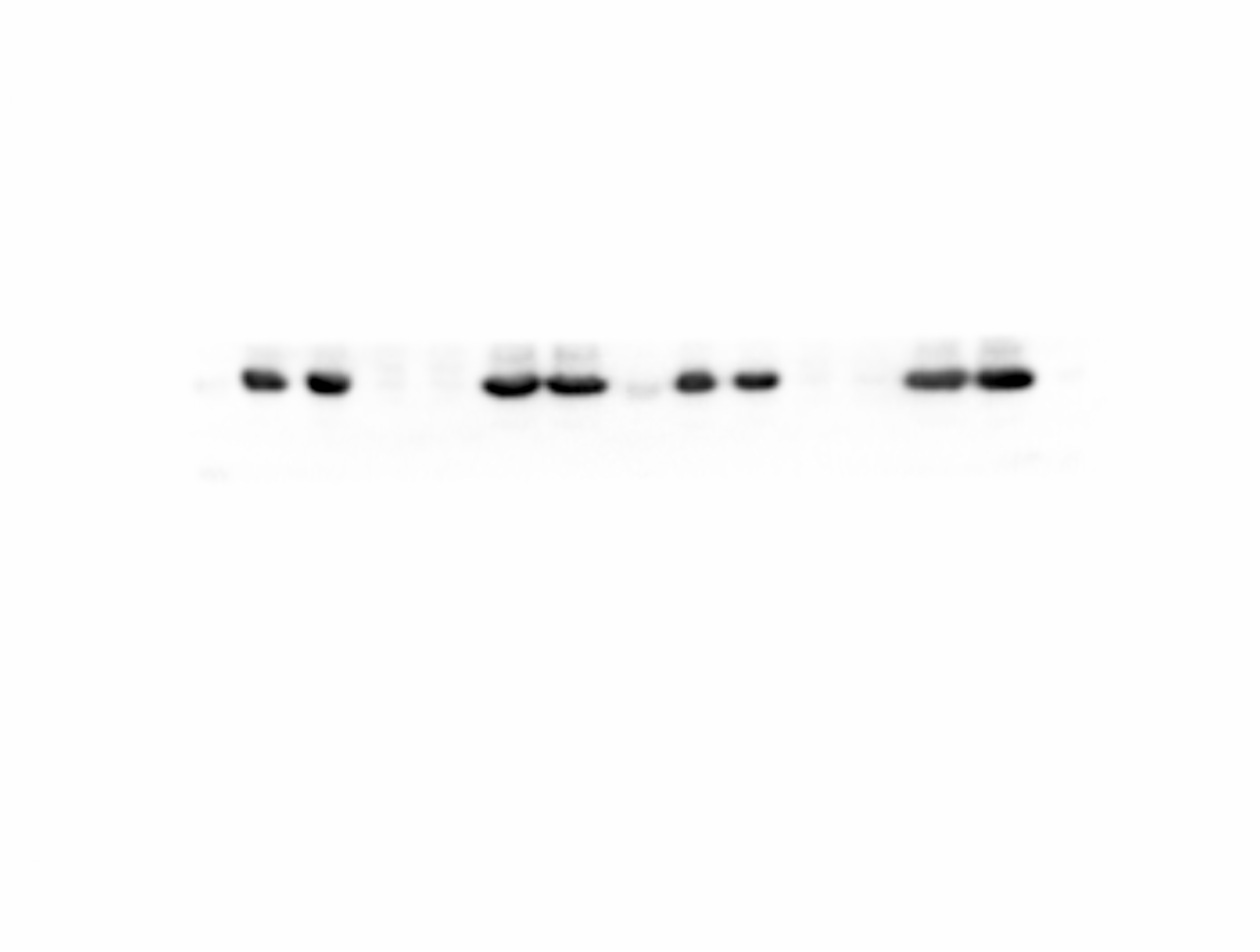

Supplement: Supplementary file 8 — Source data Fig. 4 [file 44318_2026_818_MOESM8_ESM.zip › Figure 4/Figure 4I/Figure 4I Replicate 1/H3 (lane 1-6).tif]

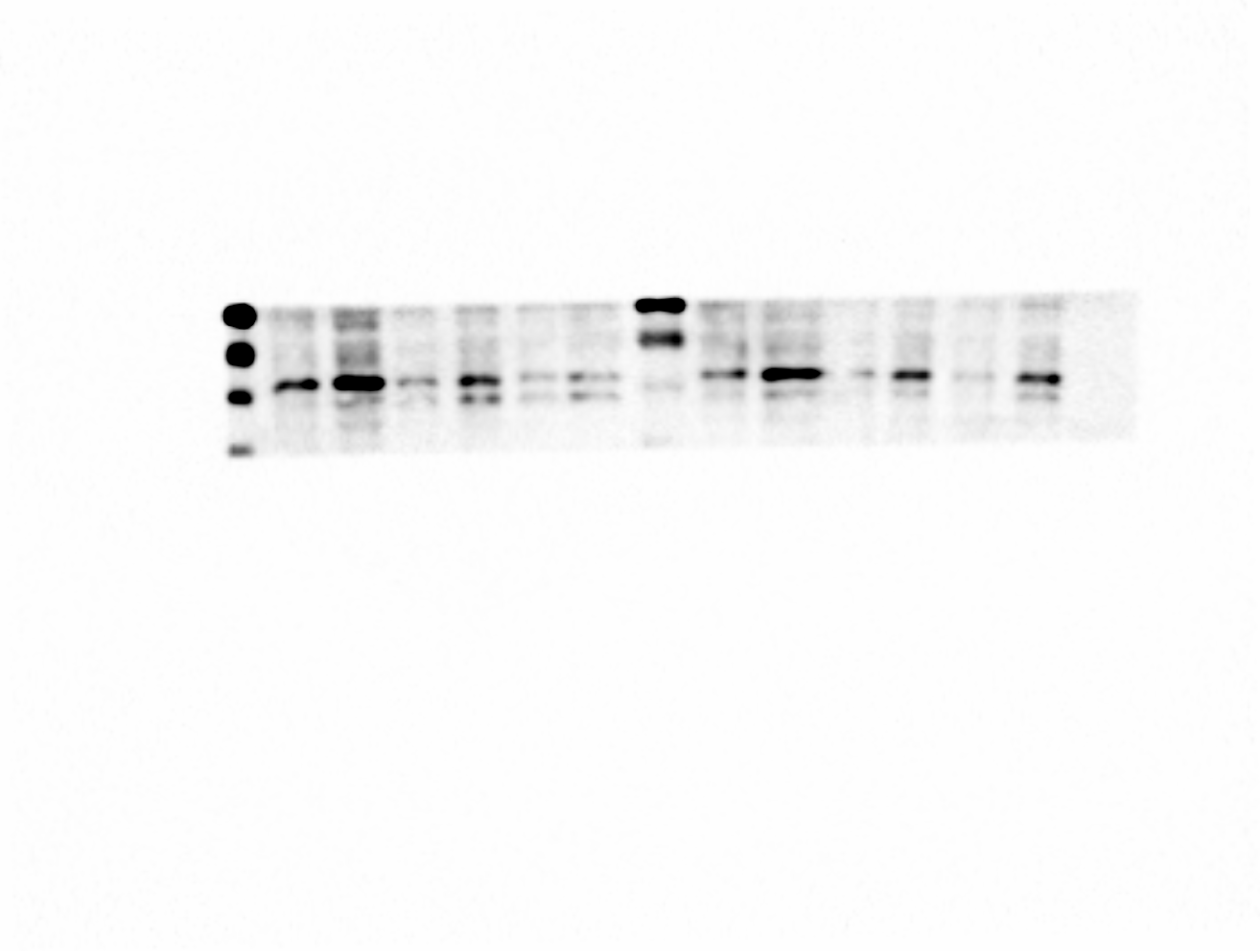

Supplement: Supplementary file 8 — Source data Fig. 4 [file 44318_2026_818_MOESM8_ESM.zip › Figure 4/Figure 4I/Figure 4I Replicate 1/TFE3 (lane 1-6).tif]

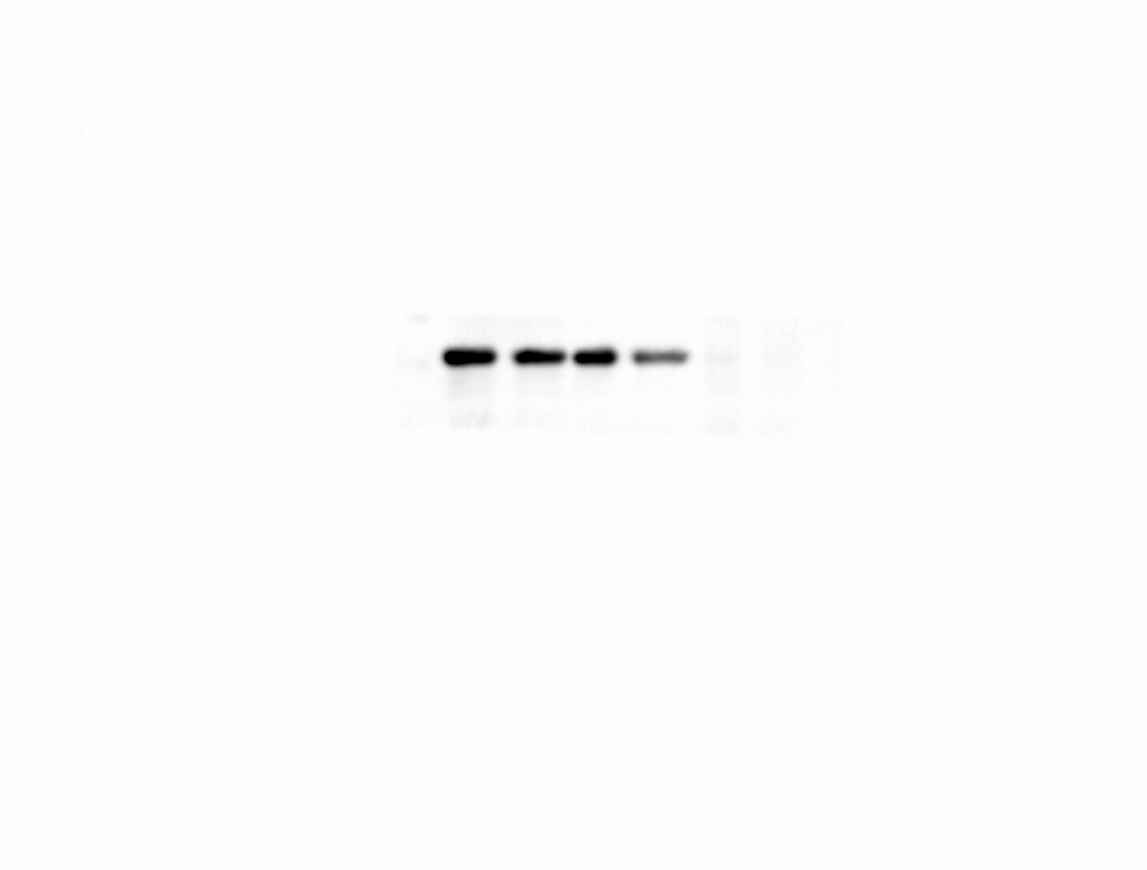

Supplement: Supplementary file 8 — Source data Fig. 4 [file 44318_2026_818_MOESM8_ESM.zip › Figure 4/Figure 4I/Figure 4I Replicate 2/GAPDH.tif]

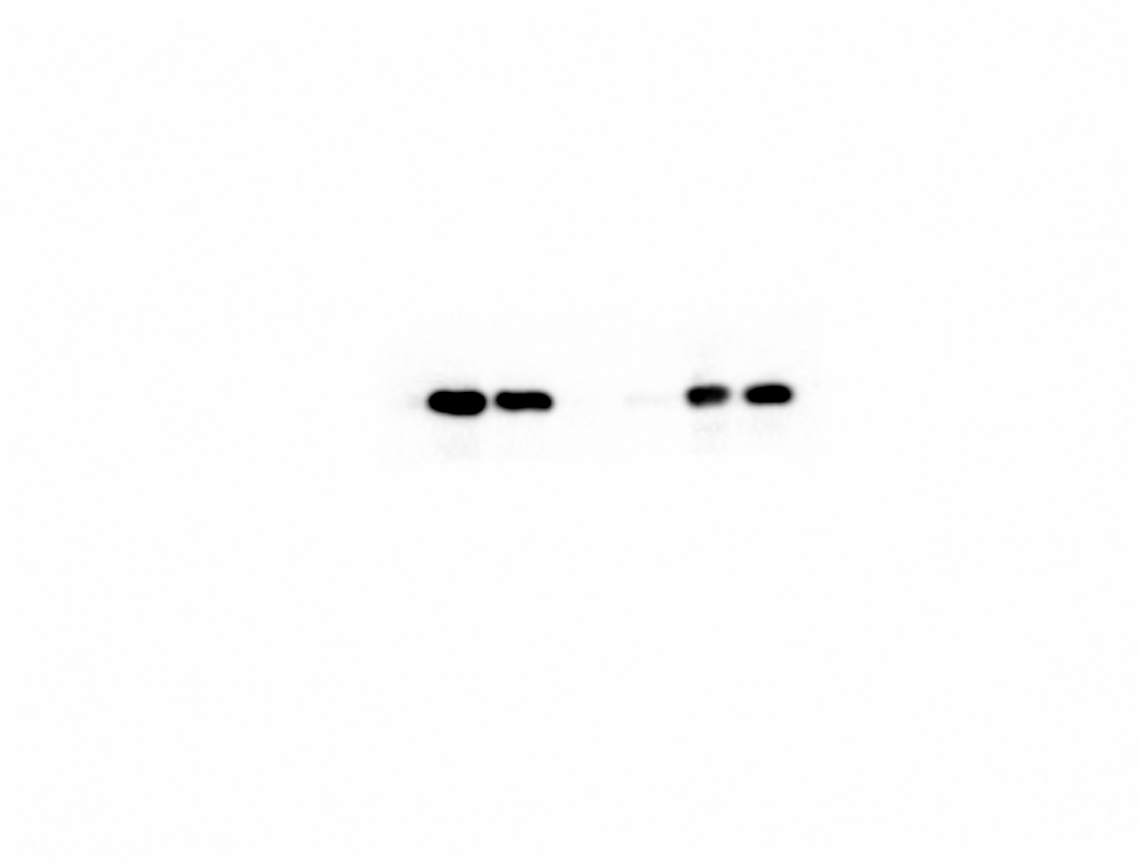

Supplement: Supplementary file 8 — Source data Fig. 4 [file 44318_2026_818_MOESM8_ESM.zip › Figure 4/Figure 4I/Figure 4I Replicate 2/H3.tif]

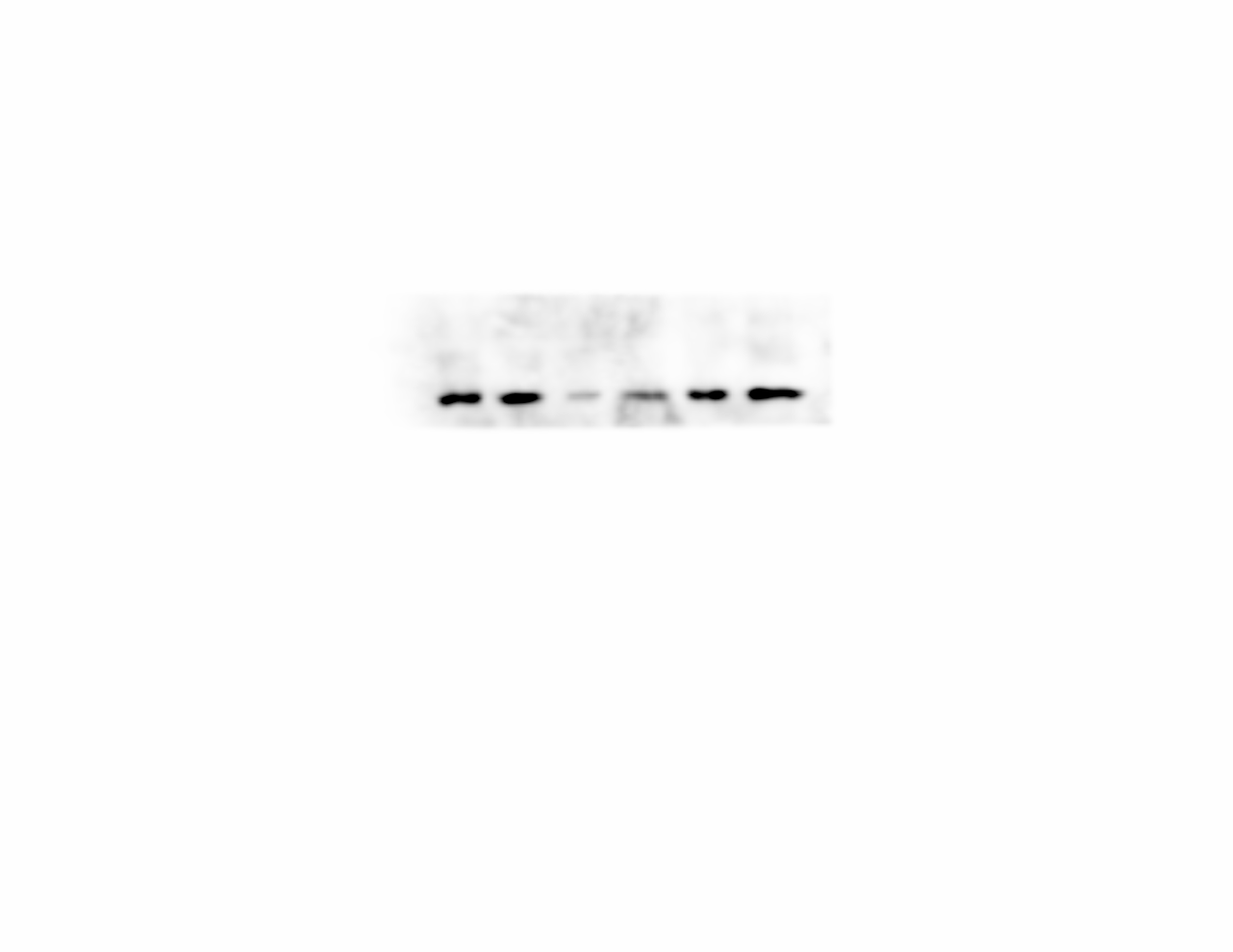

Supplement: Supplementary file 8 — Source data Fig. 4 [file 44318_2026_818_MOESM8_ESM.zip › Figure 4/Figure 4I/Figure 4I Replicate 2/TFE3.tif]

Figure 4I

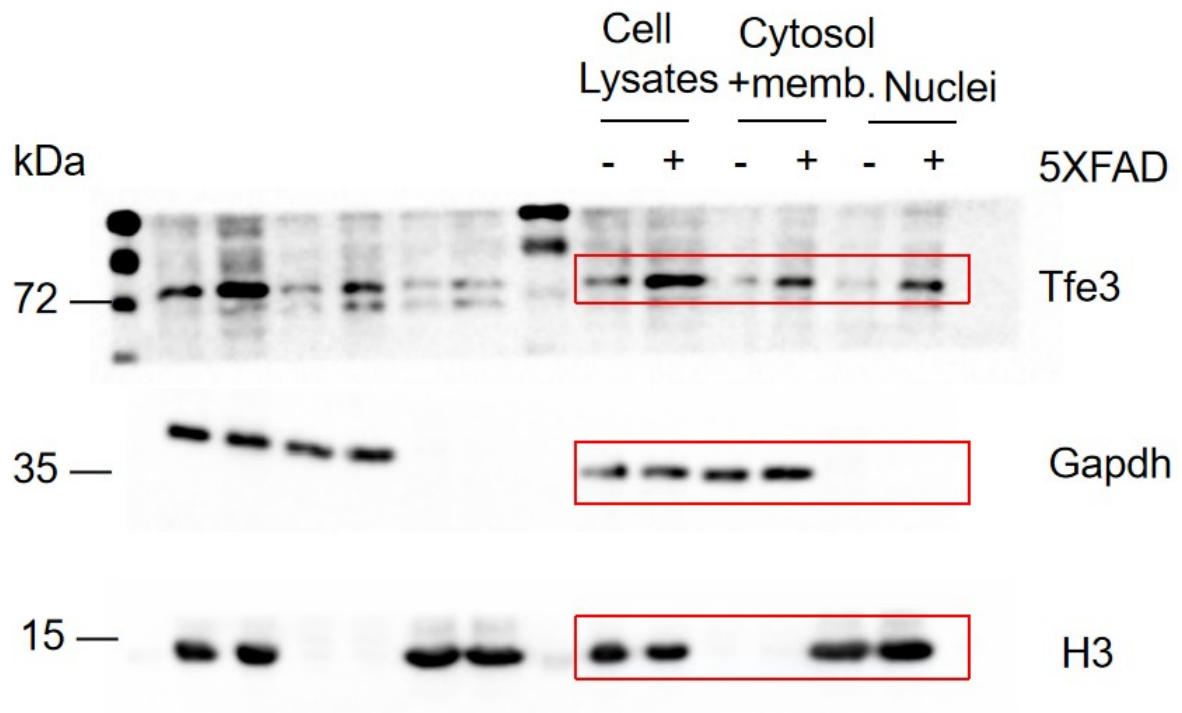

# Replicate 1

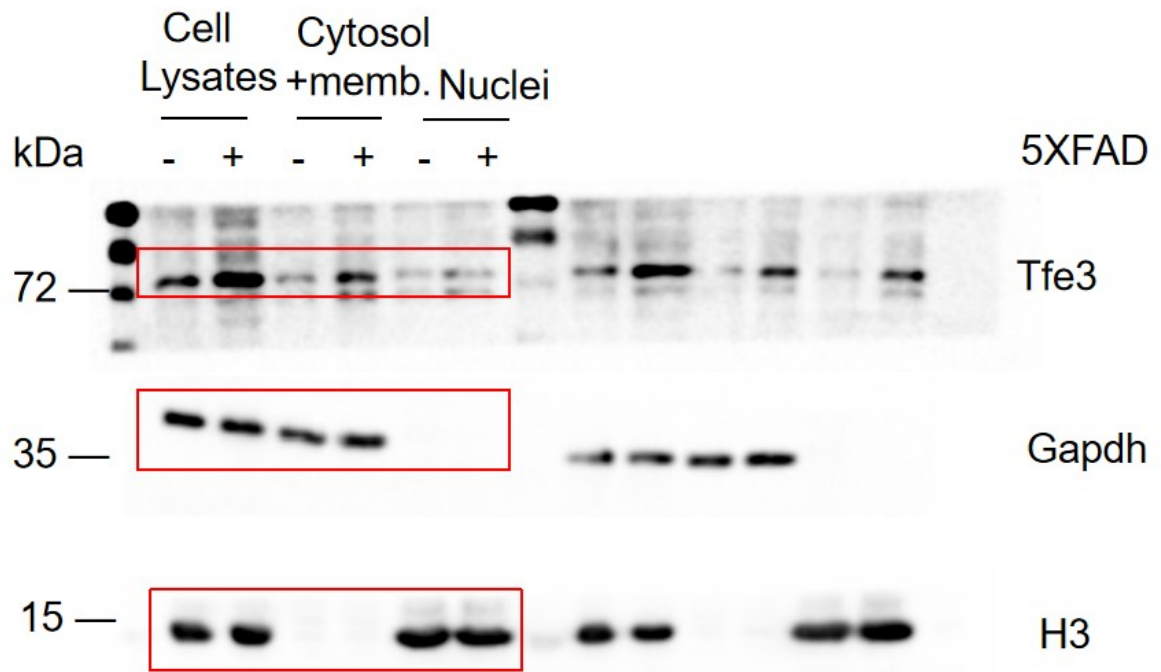

## Replicate 2

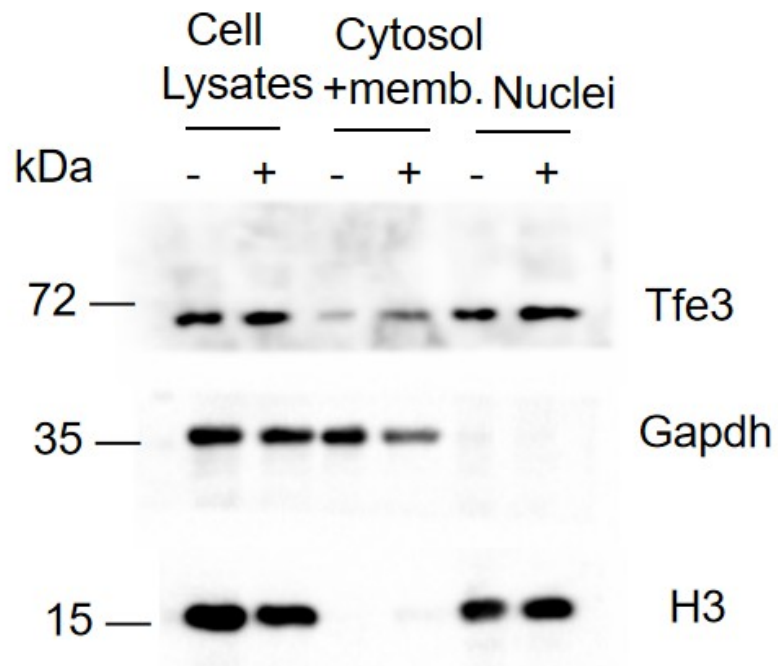

Supplement: Supplementary file 8 — Source data Fig. 4 [file 44318_2026_818_MOESM8_ESM.zip › Figure 4/Figure 4I/WB for Figure 4I.pdf]

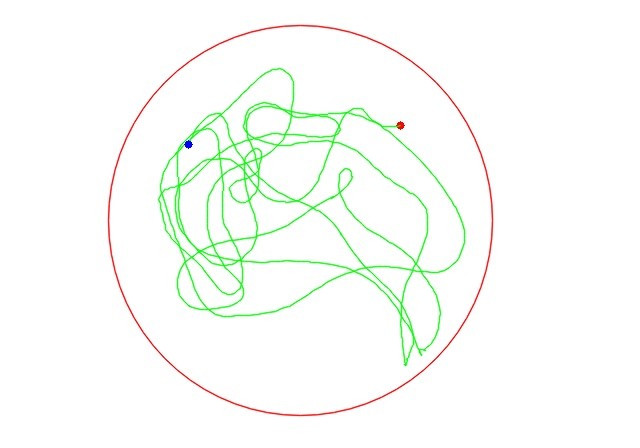

Supplement: Supplementary file 9 — Source data Fig. 5 [file 44318_2026_818_MOESM9_ESM.zip › Figure 5/Figure 5B/5XFAD+AAV.jpg]

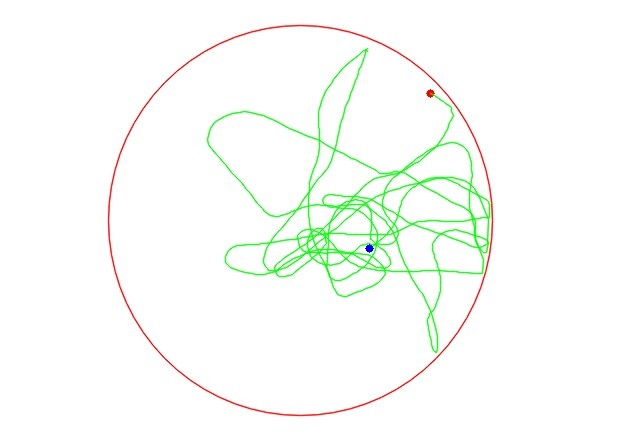

Supplement: Supplementary file 9 — Source data Fig. 5 [file 44318_2026_818_MOESM9_ESM.zip › Figure 5/Figure 5B/5XFAD+FAM134BmutLIR.jpg]

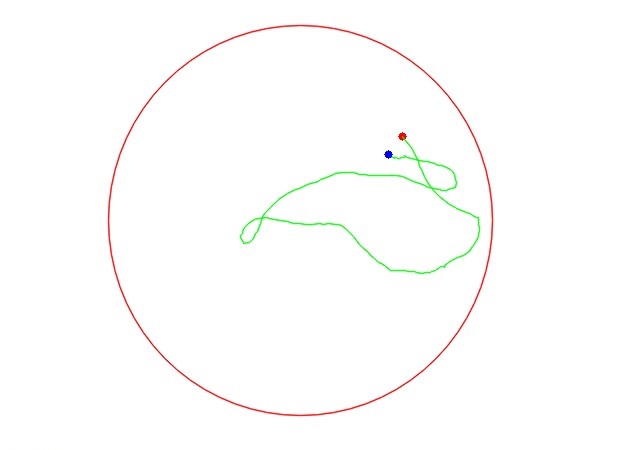

Supplement: Supplementary file 9 — Source data Fig. 5 [file 44318_2026_818_MOESM9_ESM.zip › Figure 5/Figure 5B/5XFAD+FAM134BWT.jpg]

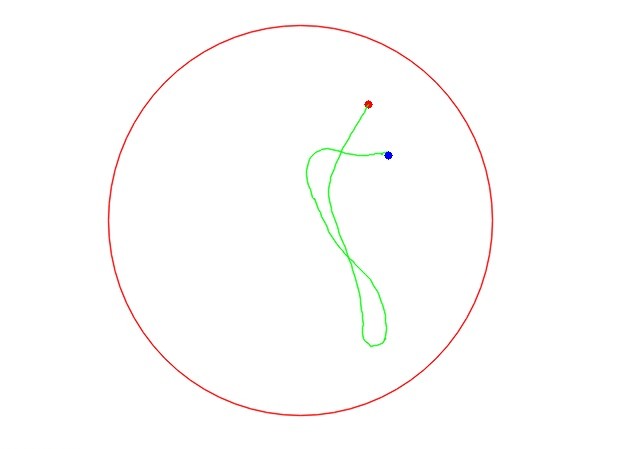

Supplement: Supplementary file 9 — Source data Fig. 5 [file 44318_2026_818_MOESM9_ESM.zip › Figure 5/Figure 5B/WT+AAV.jpg]

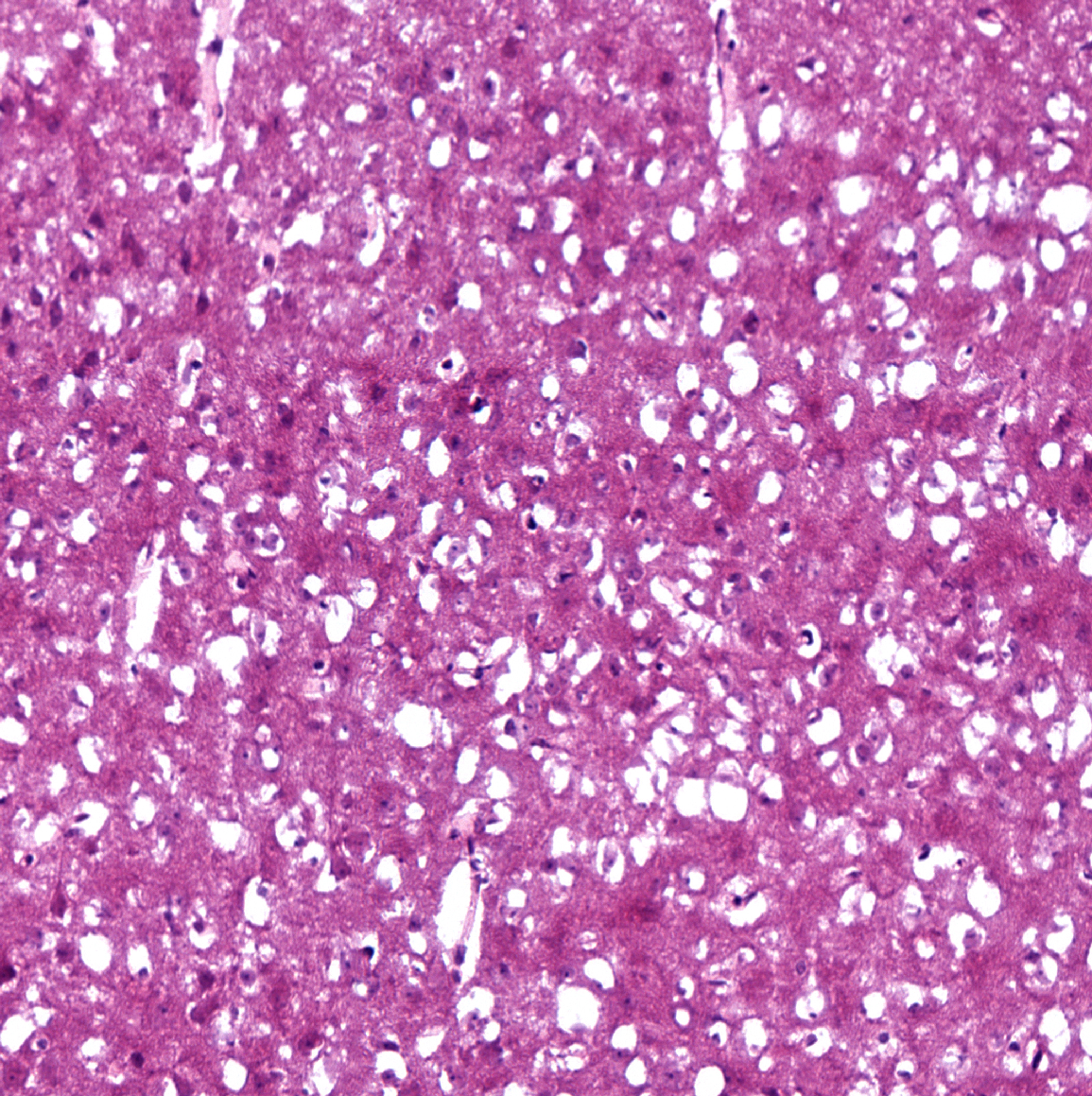

Supplement: Supplementary file 9 — Source data Fig. 5 [file 44318_2026_818_MOESM9_ESM.zip › Figure 5/Figure 5G/5XFAD+AAV.tif]

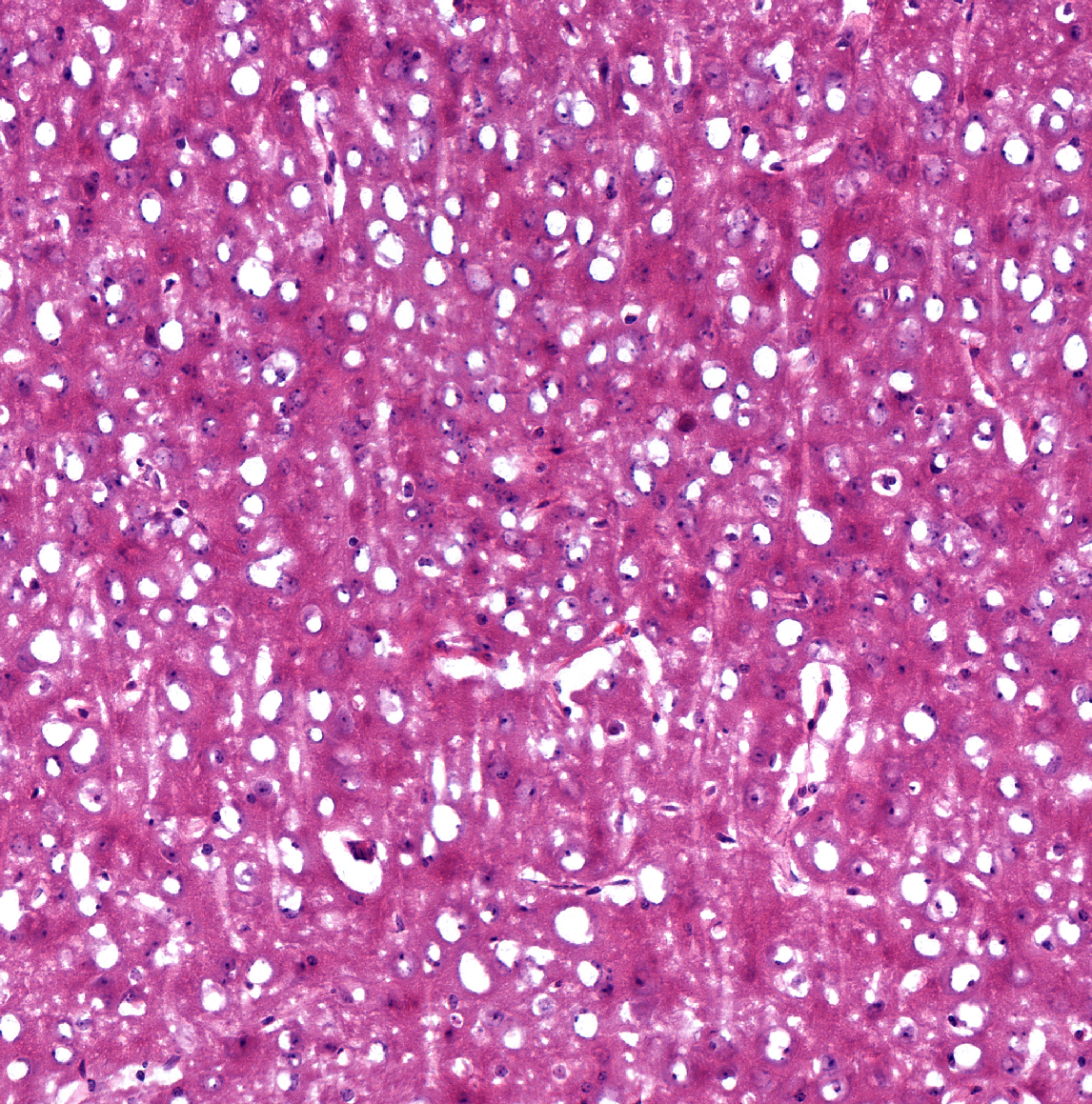

Supplement: Supplementary file 9 — Source data Fig. 5 [file 44318_2026_818_MOESM9_ESM.zip › Figure 5/Figure 5G/5XFAD+FAM134BmutLIR.tif]

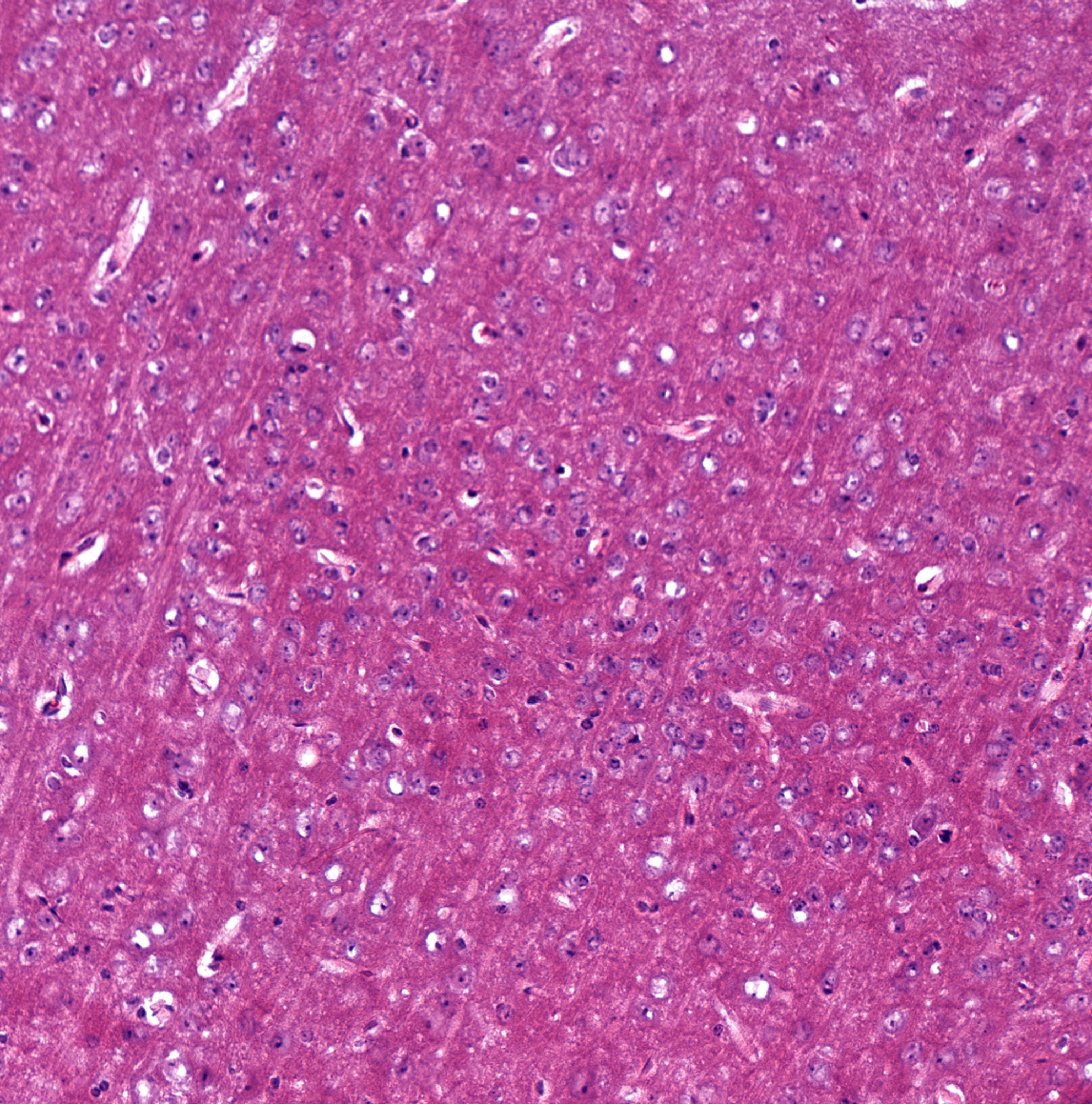

Supplement: Supplementary file 9 — Source data Fig. 5 [file 44318_2026_818_MOESM9_ESM.zip › Figure 5/Figure 5G/5XFAD+FAM134BWT.tif]

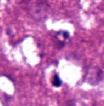

Supplement: Supplementary file 9 — Source data Fig. 5 [file 44318_2026_818_MOESM9_ESM.zip › Figure 5/Figure 5G/Inset-5XFAD+AAV.tif]

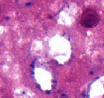

Supplement: Supplementary file 9 — Source data Fig. 5 [file 44318_2026_818_MOESM9_ESM.zip › Figure 5/Figure 5G/Inset-5XFAD+FAM134BmutLIR.tif]

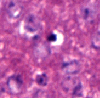

Supplement: Supplementary file 9 — Source data Fig. 5 [file 44318_2026_818_MOESM9_ESM.zip › Figure 5/Figure 5G/Inset-5XFAD+FAM134BWT.tif]

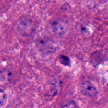

Supplement: Supplementary file 9 — Source data Fig. 5 [file 44318_2026_818_MOESM9_ESM.zip › Figure 5/Figure 5G/Inset-WT+AAV.tif]

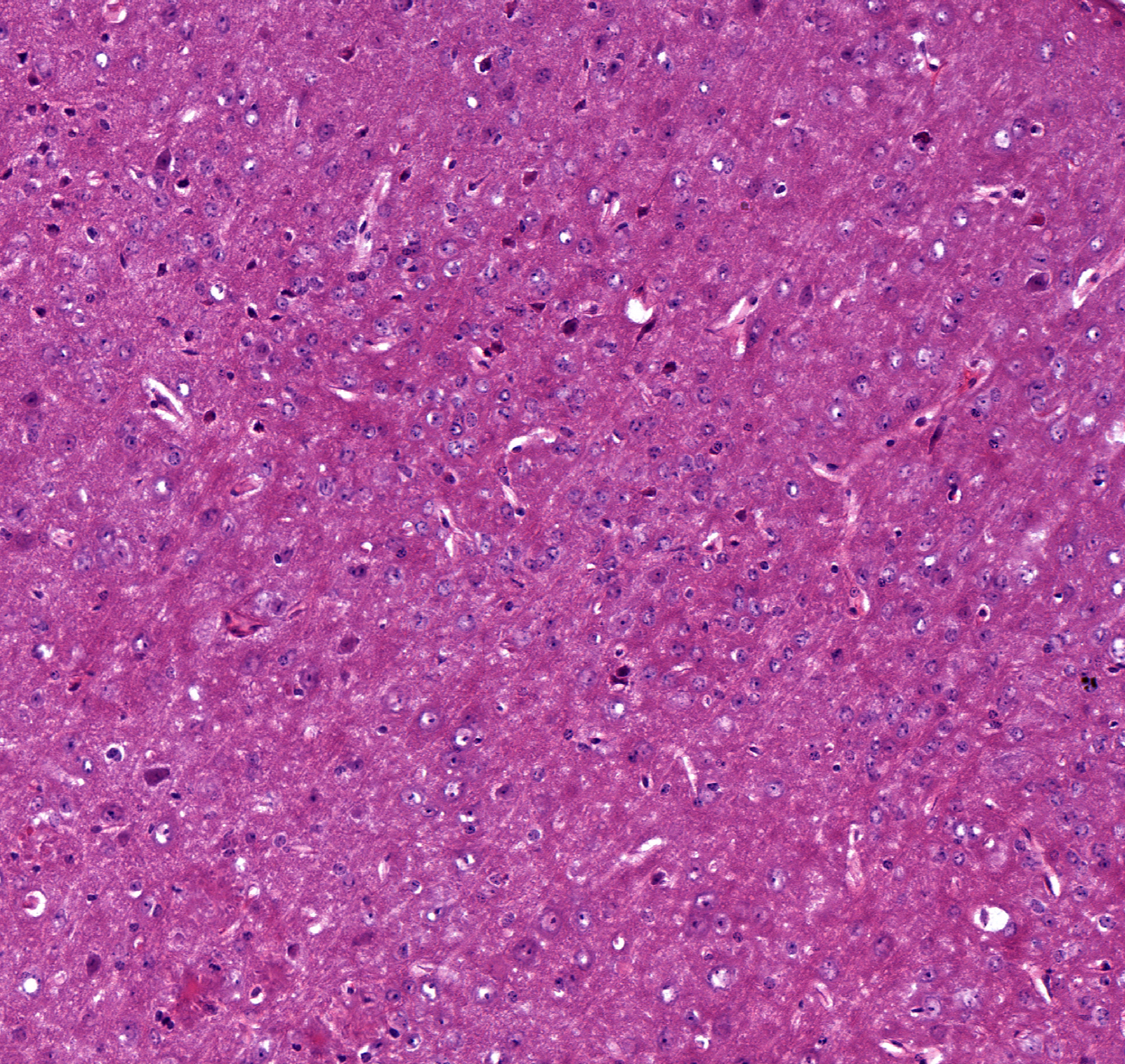

Supplement: Supplementary file 9 — Source data Fig. 5 [file 44318_2026_818_MOESM9_ESM.zip › Figure 5/Figure 5G/WT+AAV.tif]

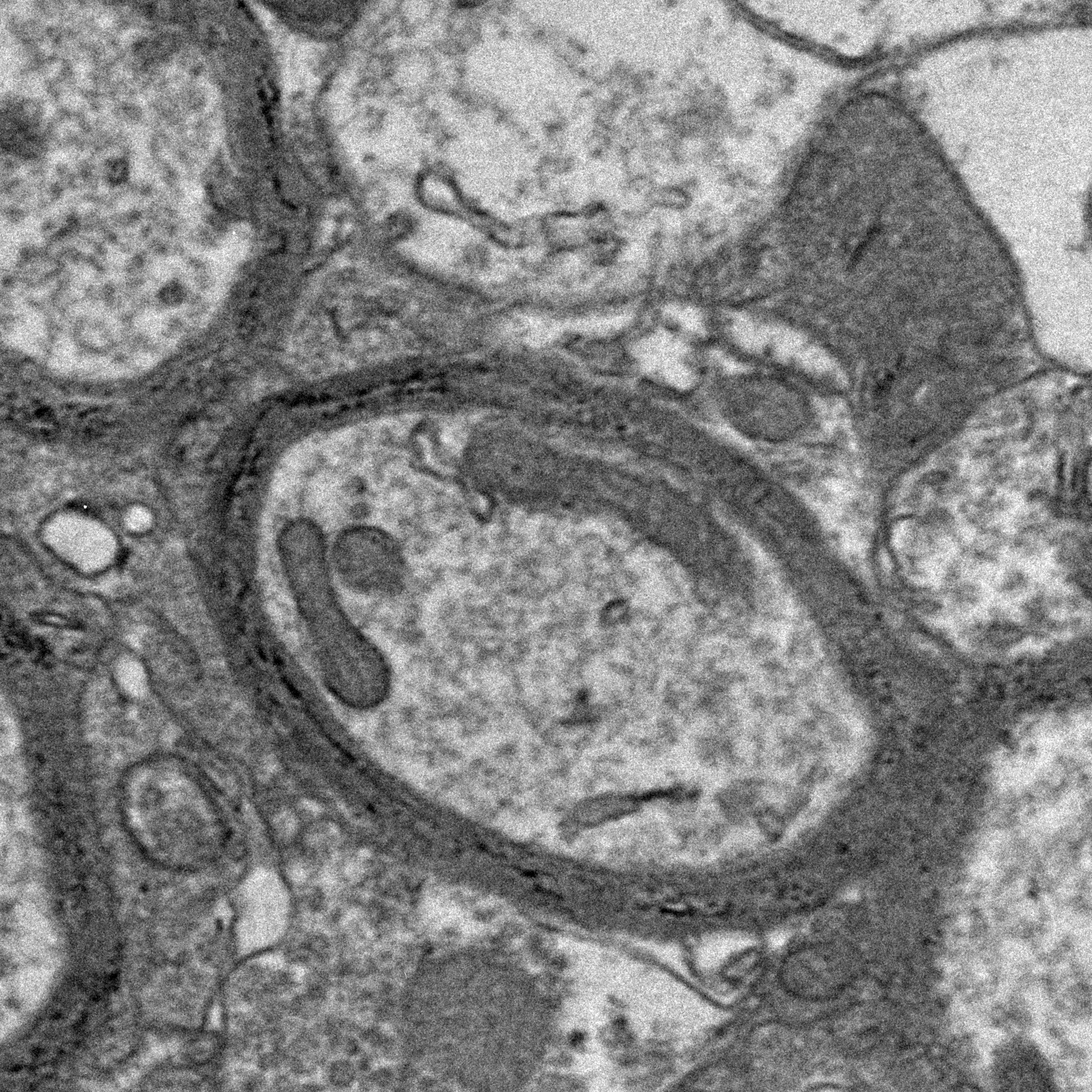

Supplement: Supplementary file 9 — Source data Fig. 5 [file 44318_2026_818_MOESM9_ESM.zip › Figure 5/Figure 5H/WT+AAV.tif]

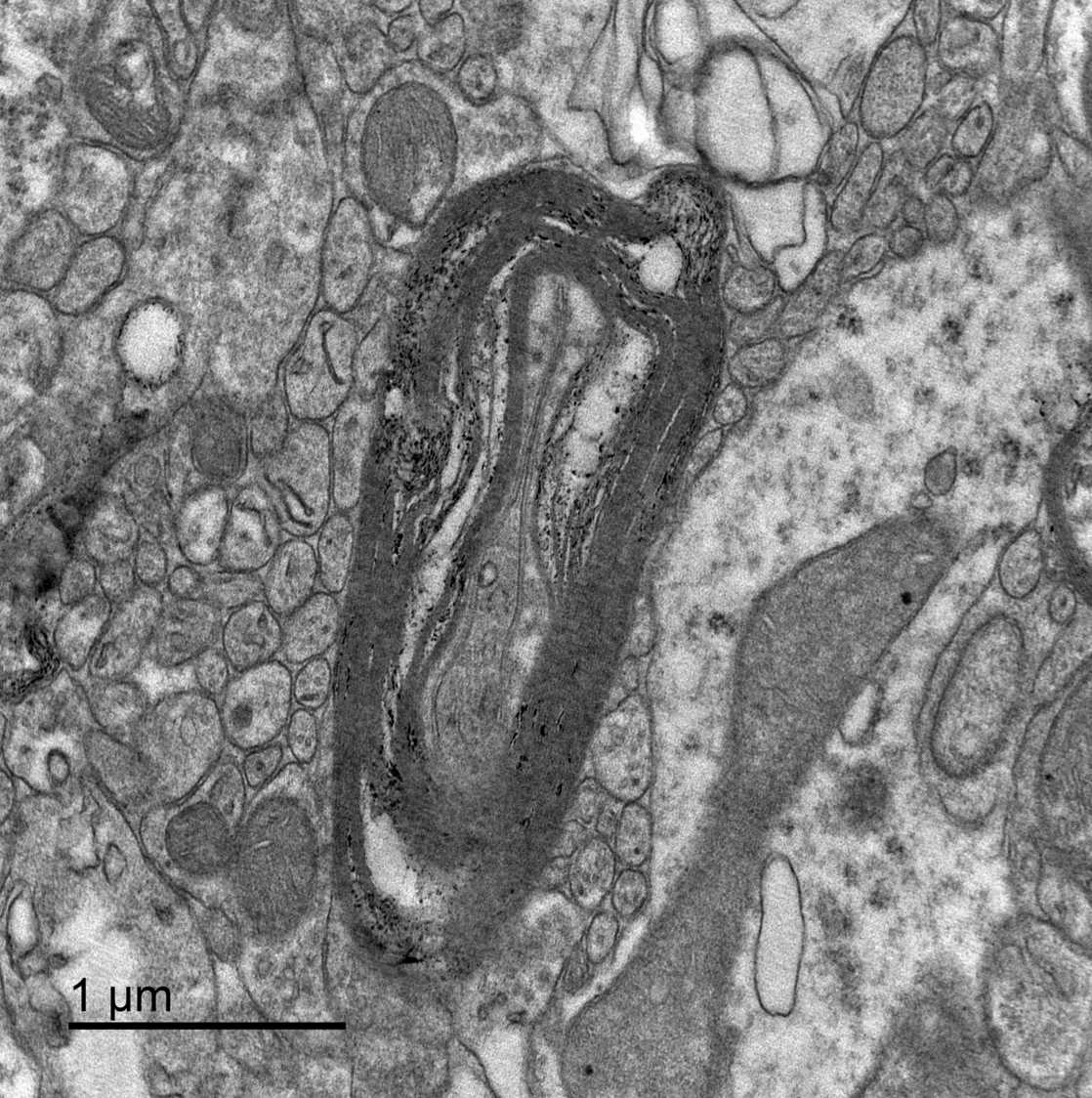

Supplement: Supplementary file 9 — Source data Fig. 5 [file 44318_2026_818_MOESM9_ESM.zip › Figure 5/Figure 5I/5XFAD+AAV.tif]

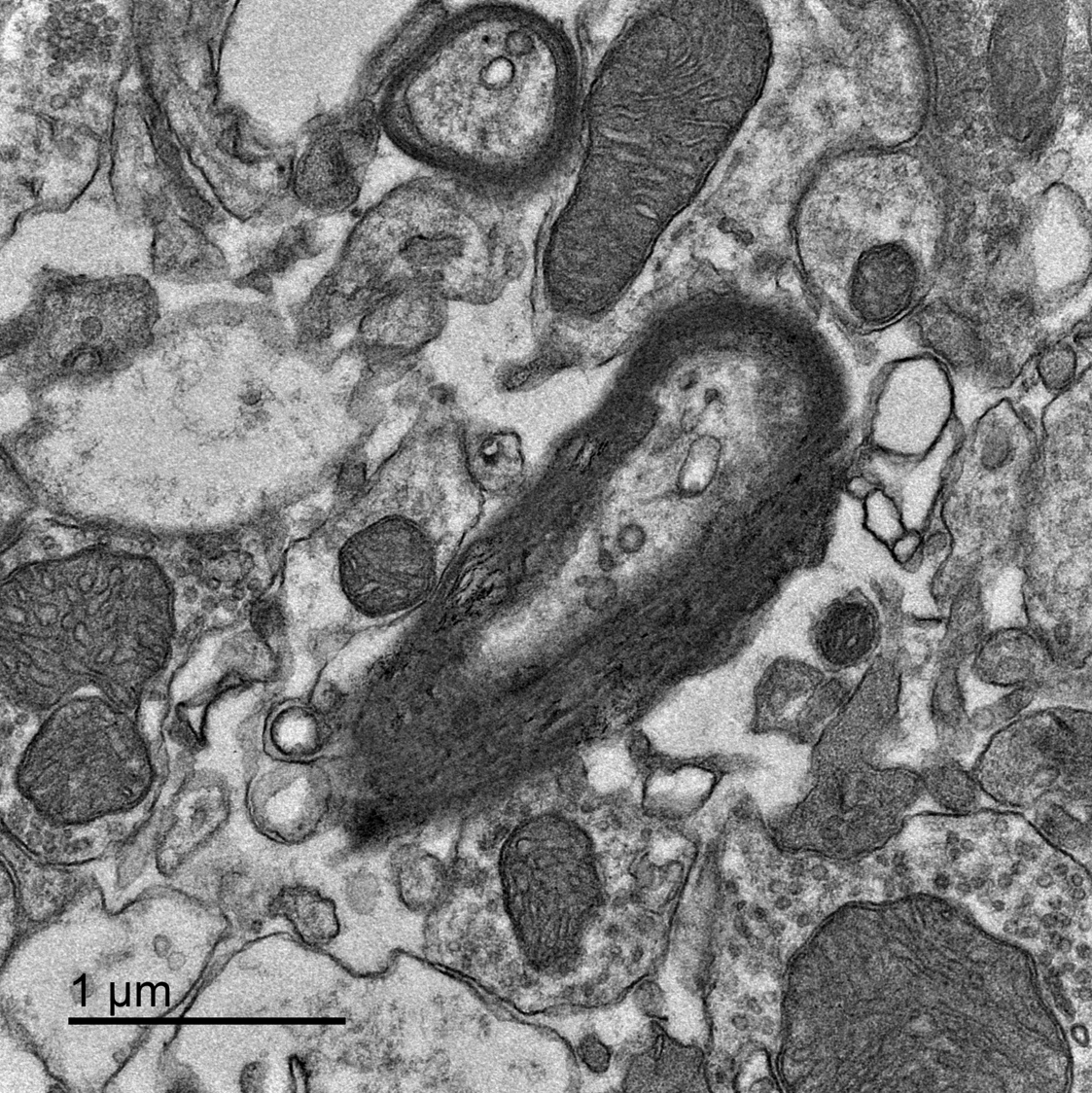

Supplement: Supplementary file 9 — Source data Fig. 5 [file 44318_2026_818_MOESM9_ESM.zip › Figure 5/Figure 5I/5XFAD+FAM134BmutLIR.tif]

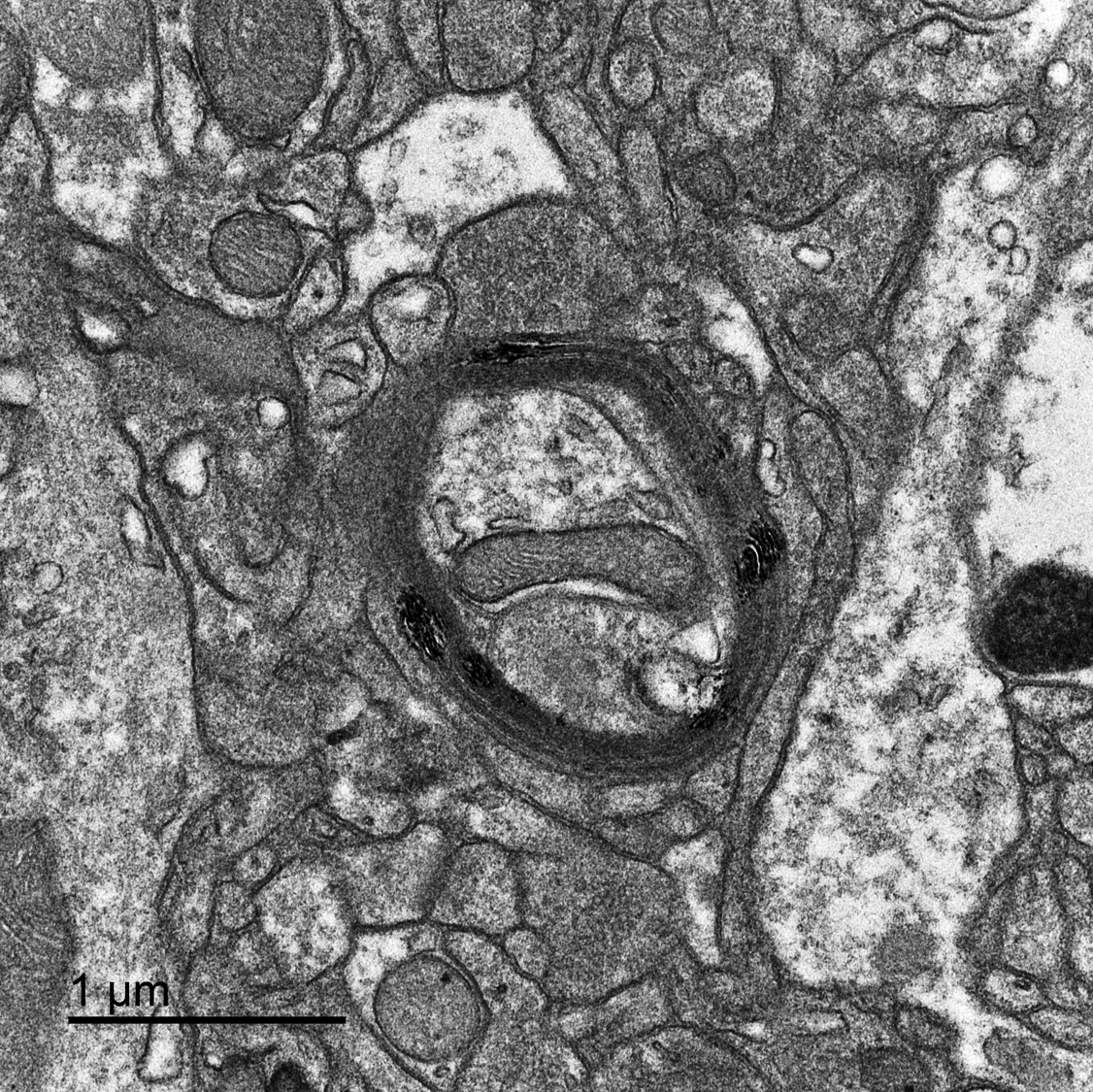

Supplement: Supplementary file 9 — Source data Fig. 5 [file 44318_2026_818_MOESM9_ESM.zip › Figure 5/Figure 5I/5XFAD+FAM134BWT.tif]

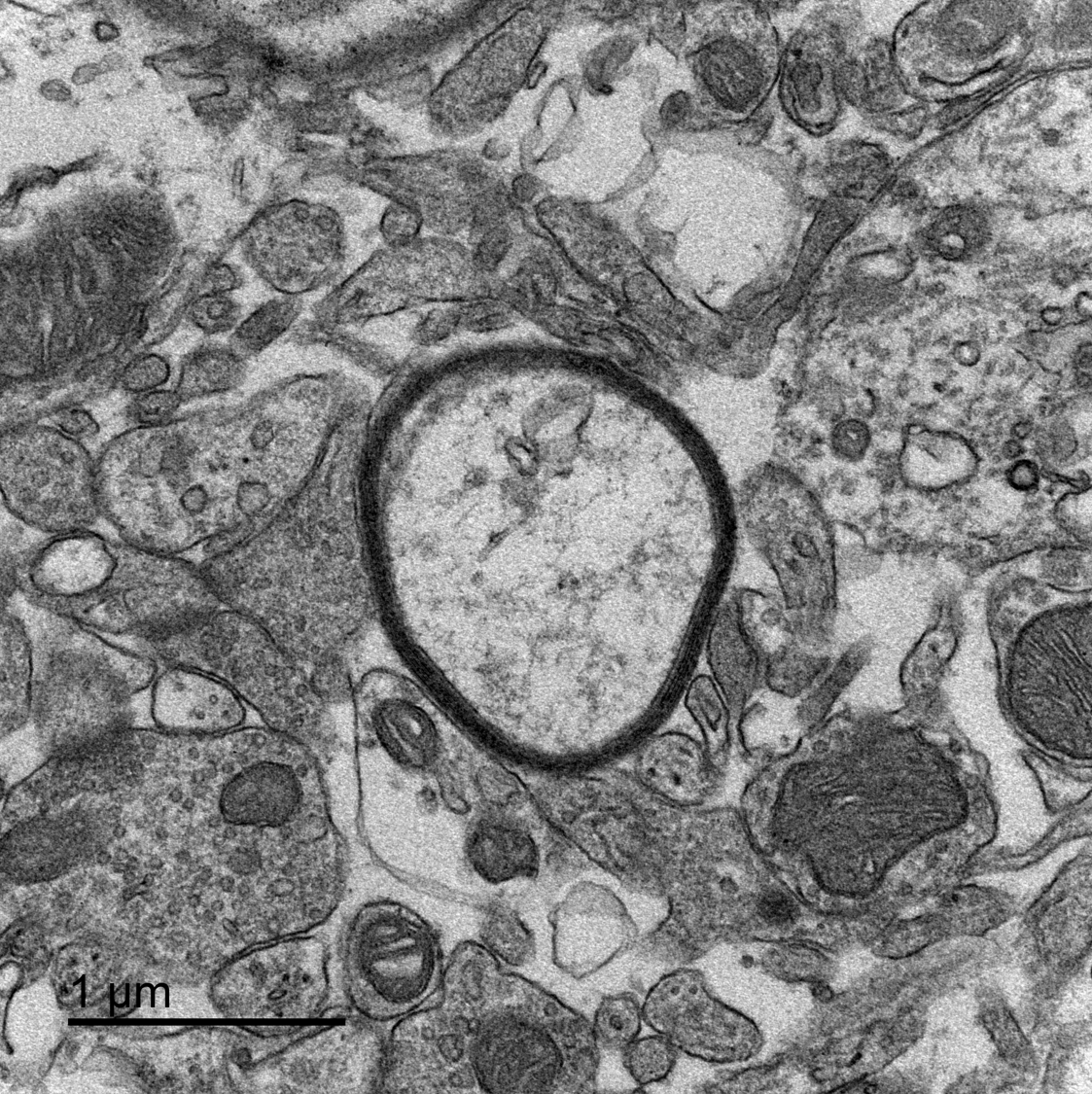

Supplement: Supplementary file 9 — Source data Fig. 5 [file 44318_2026_818_MOESM9_ESM.zip › Figure 5/Figure 5I/WT+AAV.tif]

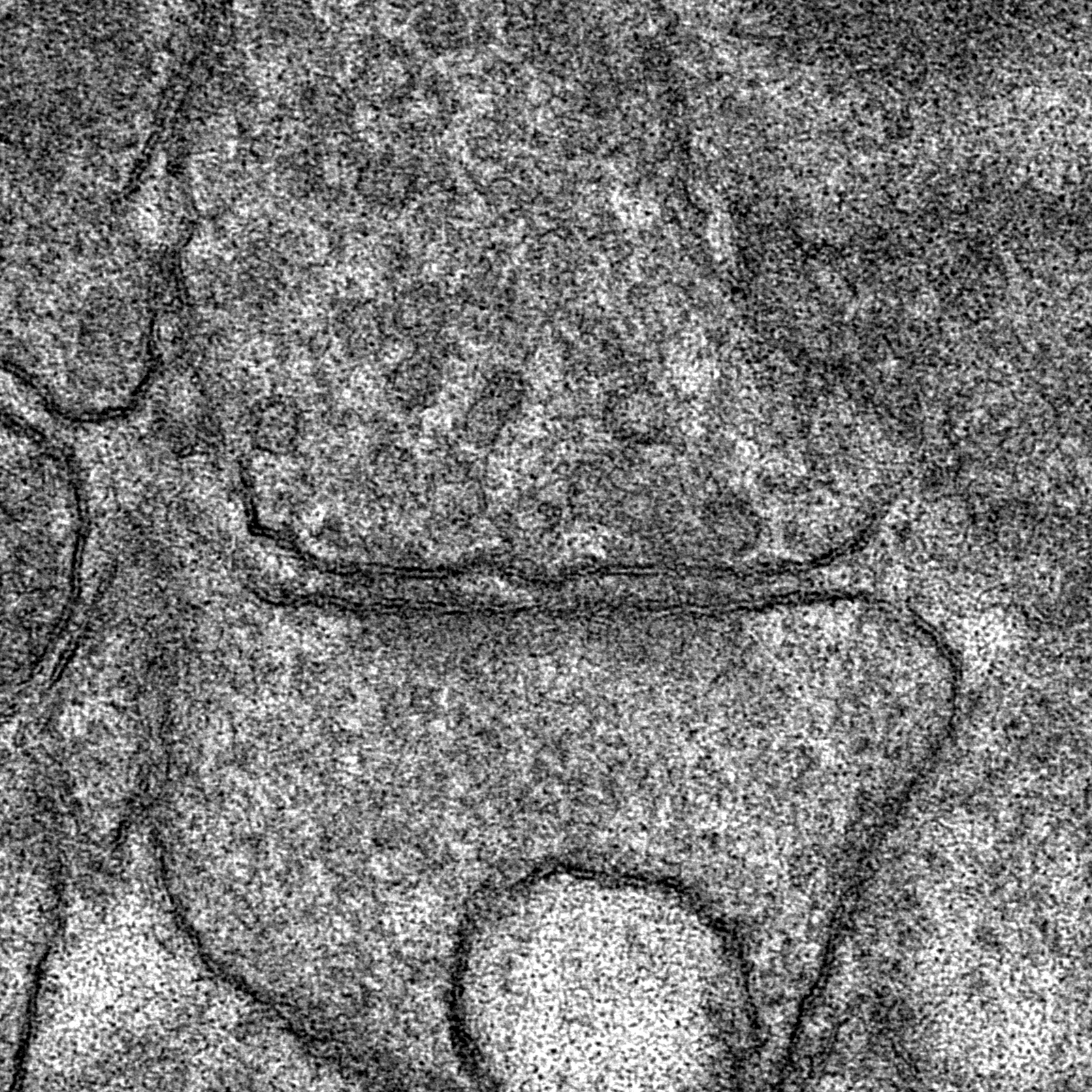

Supplement: Supplementary file 9 — Source data Fig. 5 [file 44318_2026_818_MOESM9_ESM.zip › Figure 5/Figure 5J/5XFAD+AAV.tif]

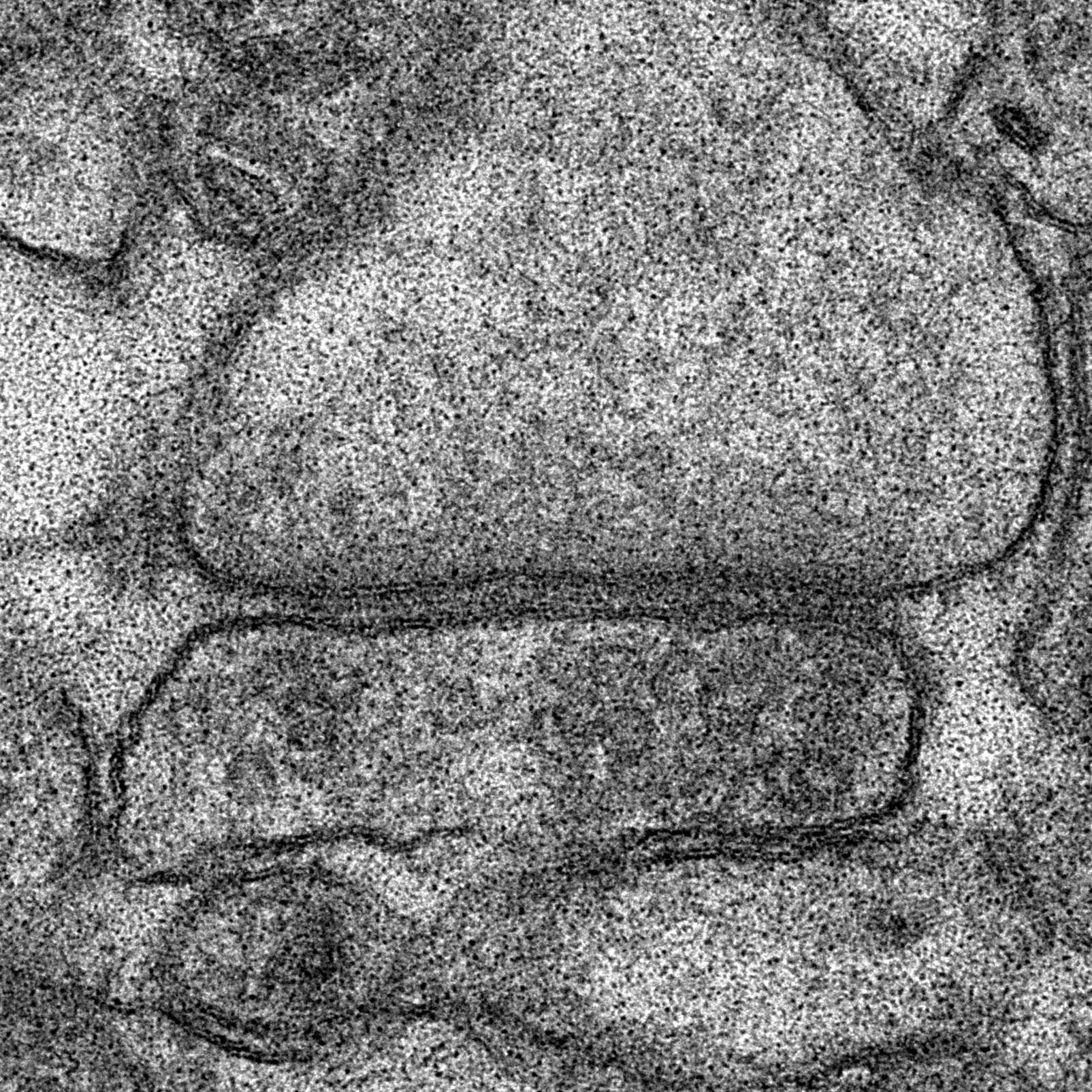

Supplement: Supplementary file 9 — Source data Fig. 5 [file 44318_2026_818_MOESM9_ESM.zip › Figure 5/Figure 5J/5XFAD+FAM134BmutLIR.tif]

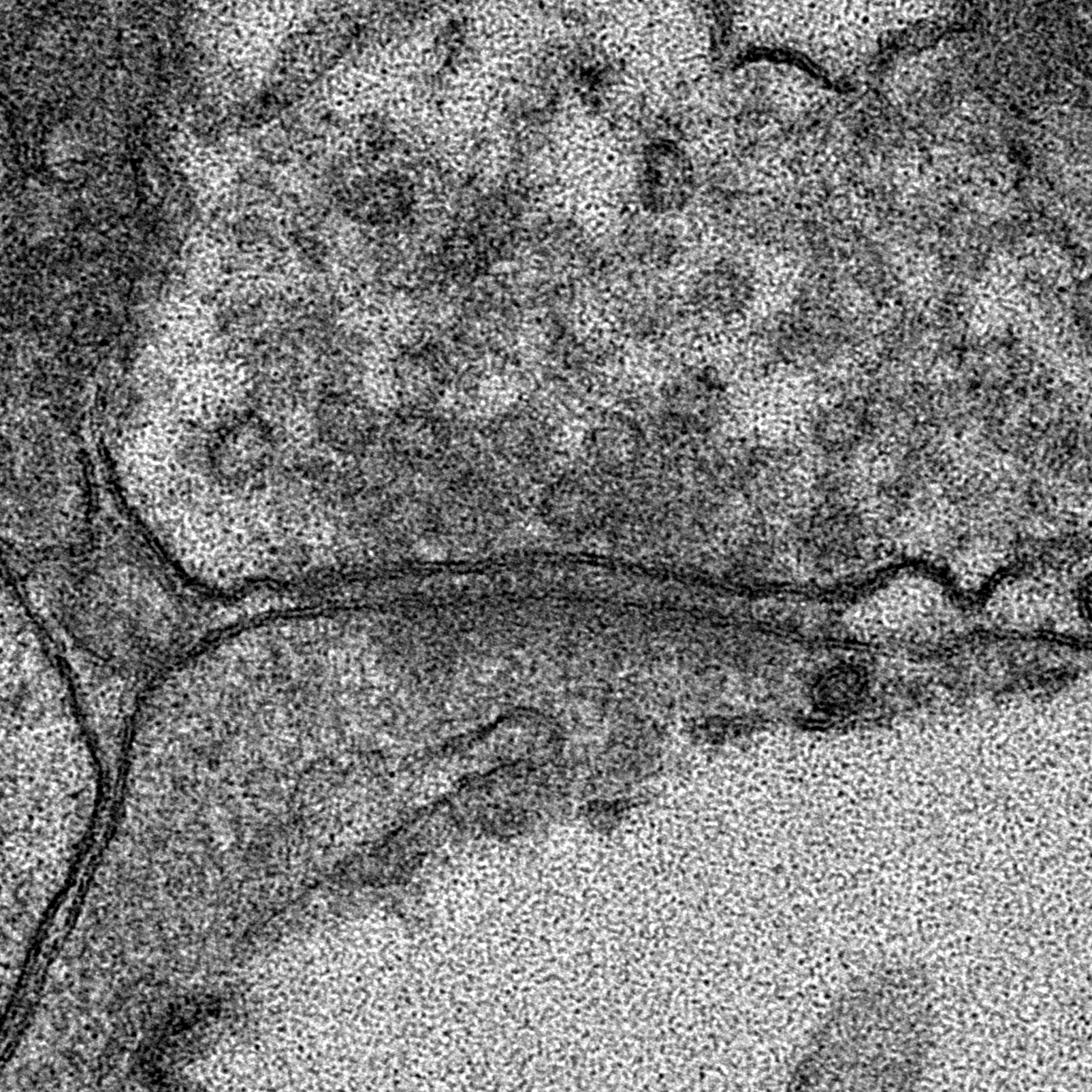

Supplement: Supplementary file 9 — Source data Fig. 5 [file 44318_2026_818_MOESM9_ESM.zip › Figure 5/Figure 5J/5XFAD+FAM134BWT.tif]

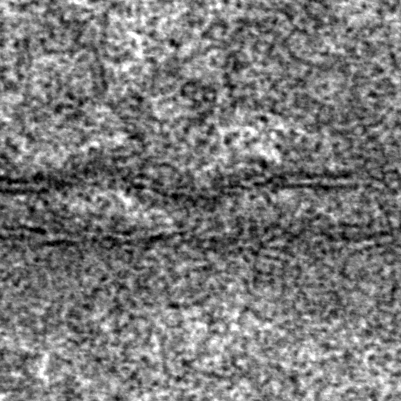

Supplement: Supplementary file 9 — Source data Fig. 5 [file 44318_2026_818_MOESM9_ESM.zip › Figure 5/Figure 5J/Inset-5XFAD+AAV.tif]

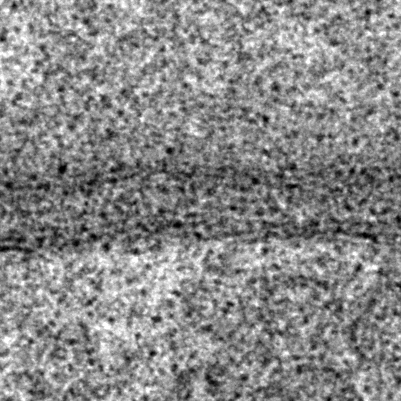

Supplement: Supplementary file 9 — Source data Fig. 5 [file 44318_2026_818_MOESM9_ESM.zip › Figure 5/Figure 5J/Inset-5XFAD+FAM134BmutLIR.tif]

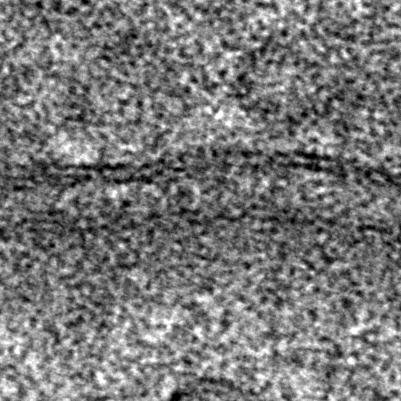

Supplement: Supplementary file 9 — Source data Fig. 5 [file 44318_2026_818_MOESM9_ESM.zip › Figure 5/Figure 5J/Inset-5XFAD+FAM134BWT.tif]

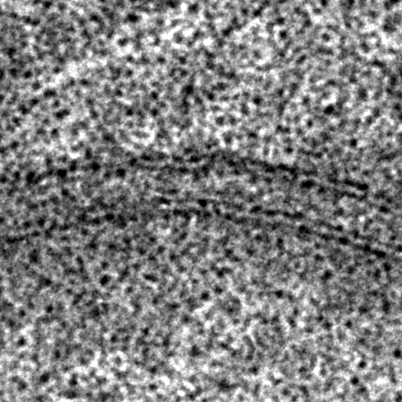

Supplement: Supplementary file 9 — Source data Fig. 5 [file 44318_2026_818_MOESM9_ESM.zip › Figure 5/Figure 5J/Inset-WT+AAV.tif]

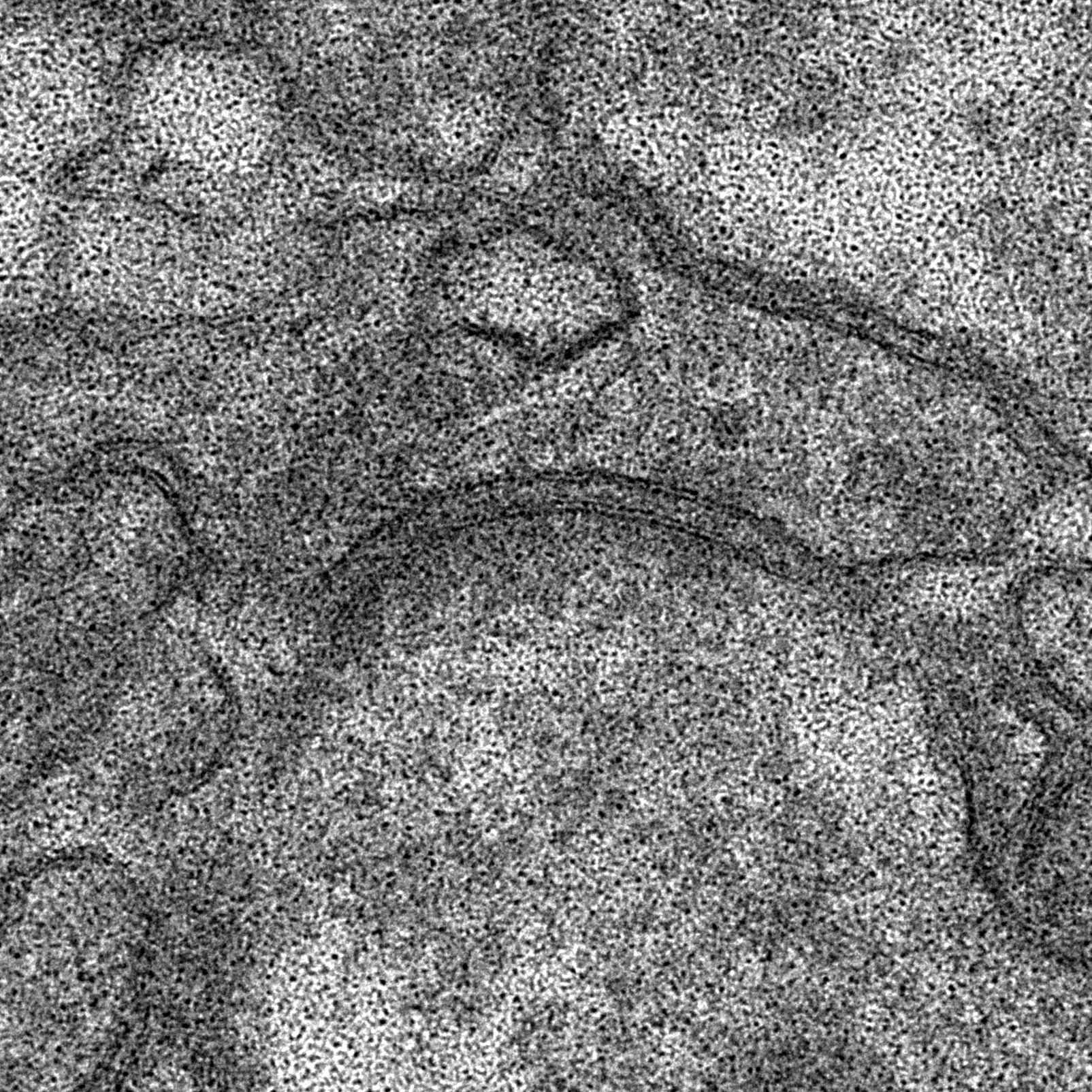

Supplement: Supplementary file 9 — Source data Fig. 5 [file 44318_2026_818_MOESM9_ESM.zip › Figure 5/Figure 5J/WT+AAV.tif]

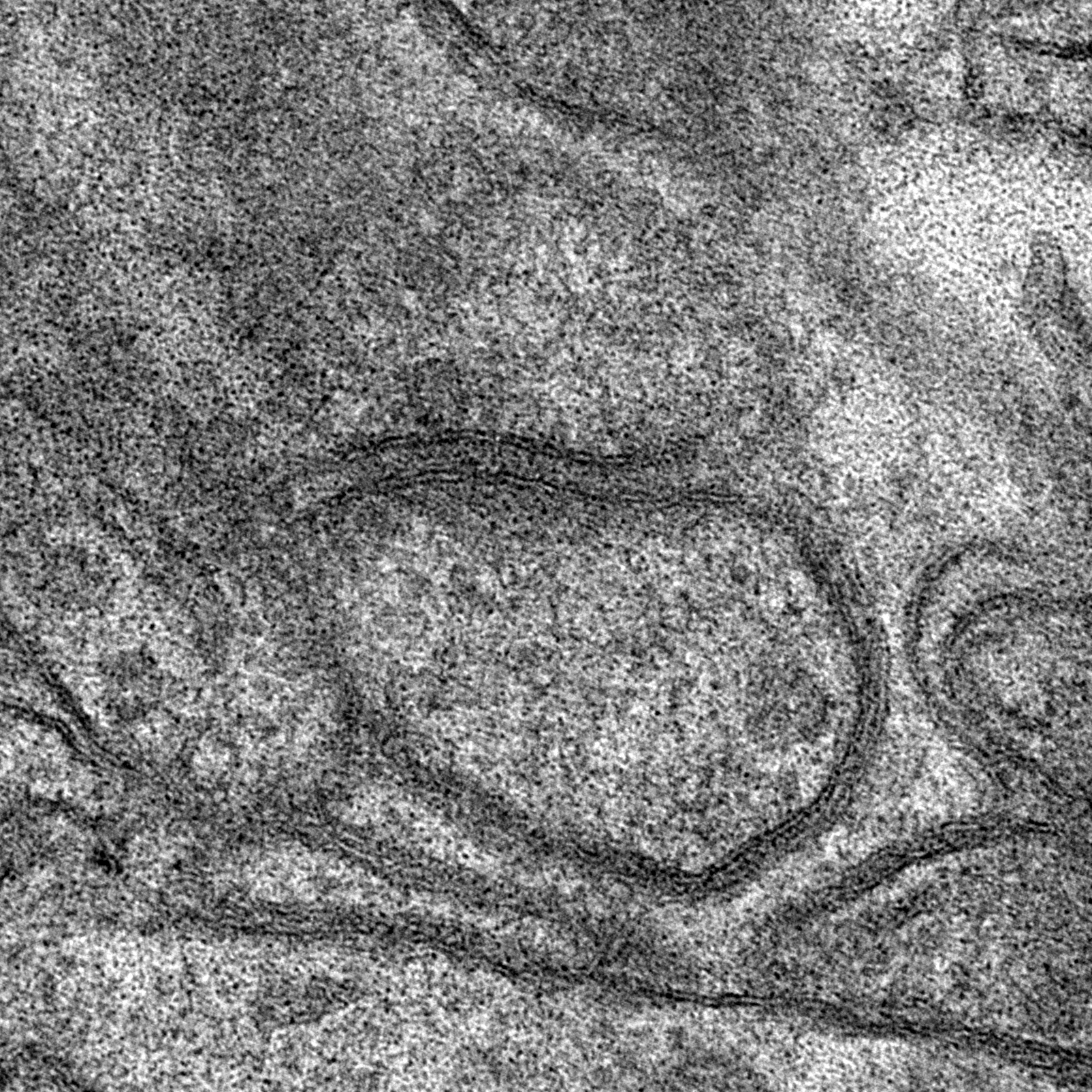

Supplement: Supplementary file 9 — Source data Fig. 5 [file 44318_2026_818_MOESM9_ESM.zip › Figure 5/Figure 5K/5XFAD+AAV.tif]

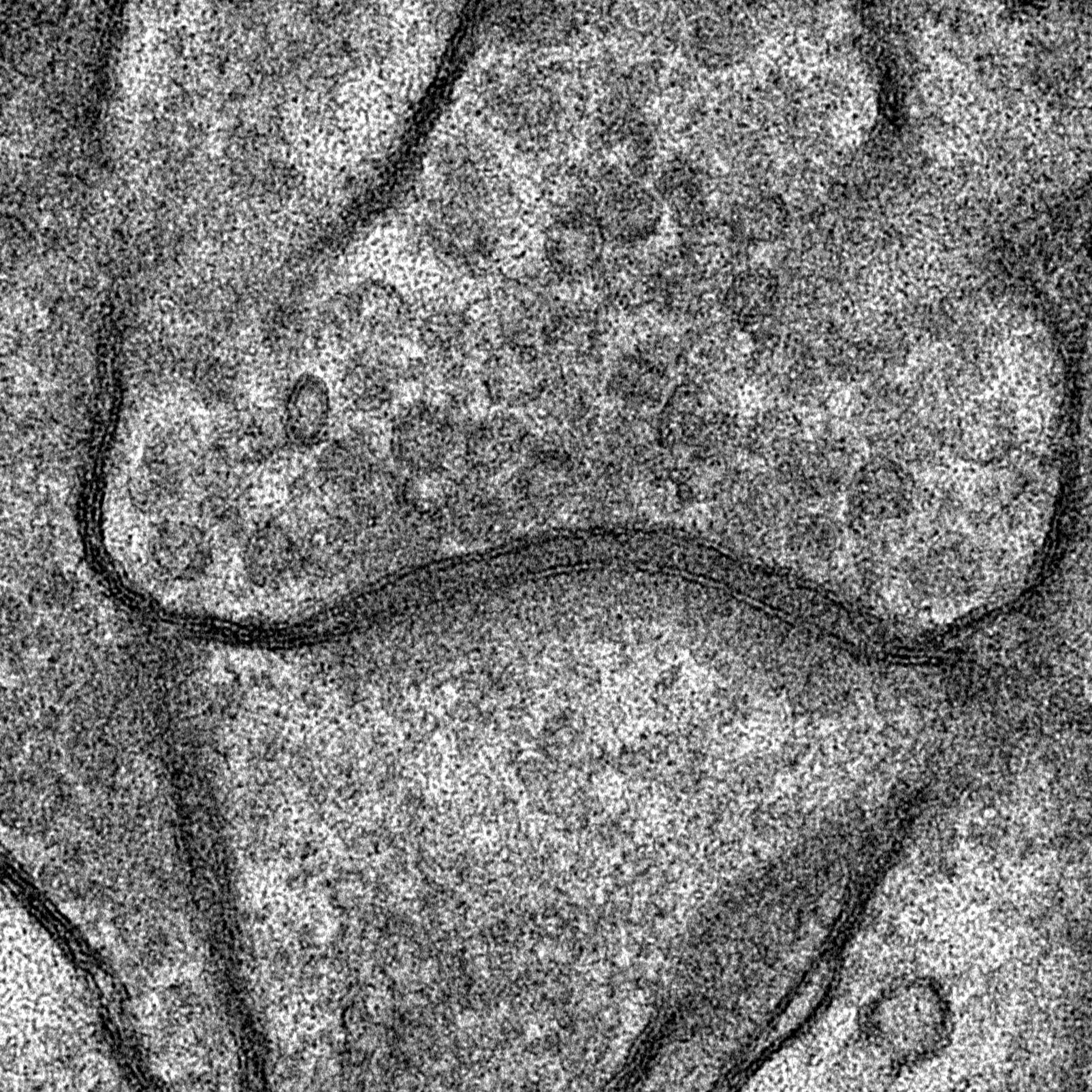

Supplement: Supplementary file 9 — Source data Fig. 5 [file 44318_2026_818_MOESM9_ESM.zip › Figure 5/Figure 5K/5XFAD+FAM134BmutLIR.tif]

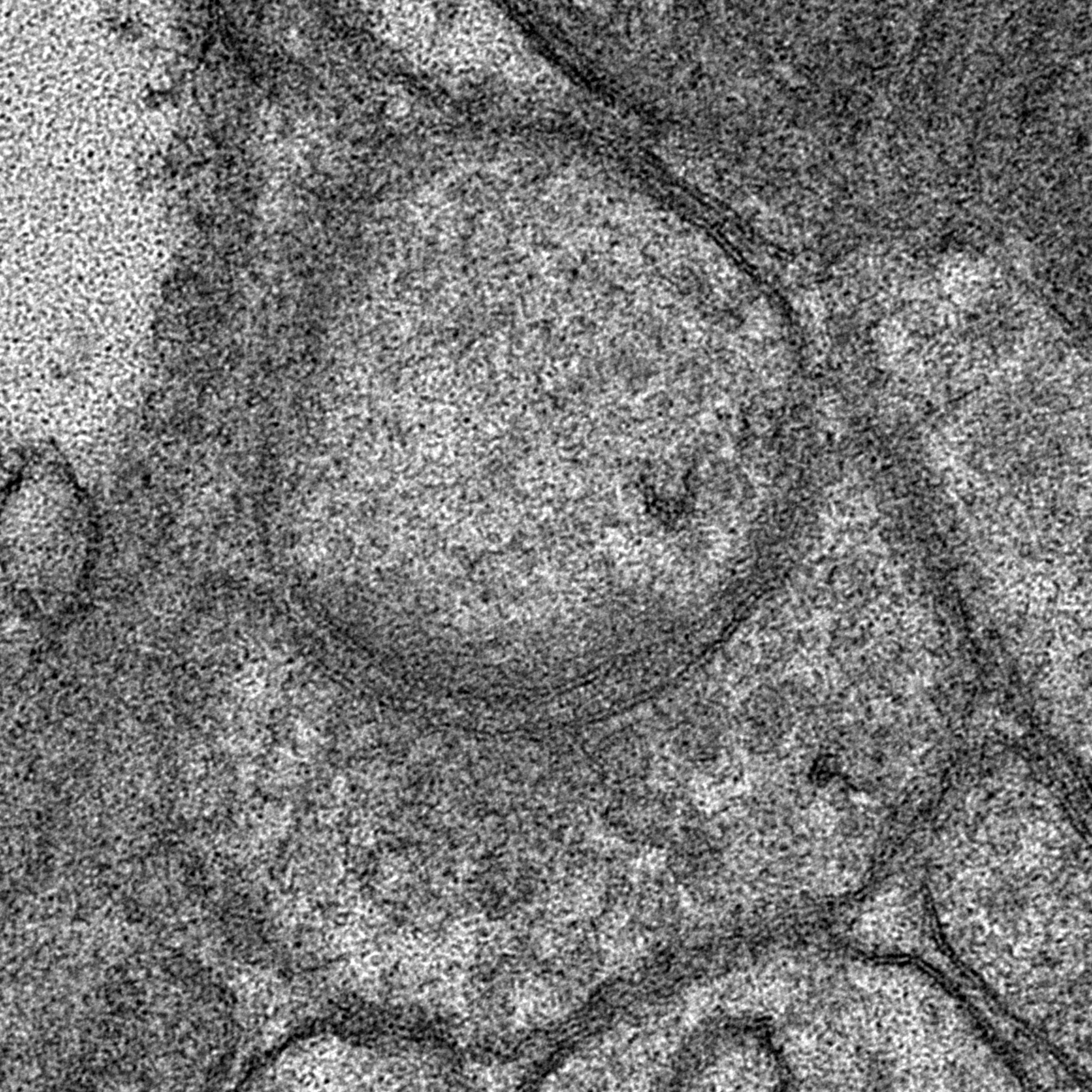

Supplement: Supplementary file 9 — Source data Fig. 5 [file 44318_2026_818_MOESM9_ESM.zip › Figure 5/Figure 5K/5XFAD+FAM134BWT.tif]

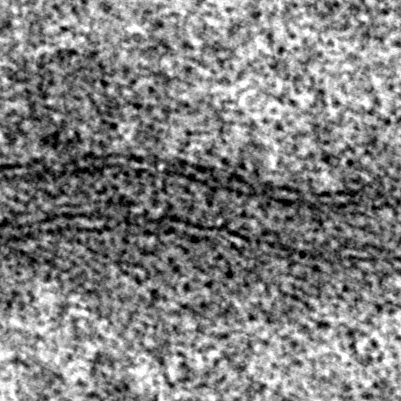

Supplement: Supplementary file 9 — Source data Fig. 5 [file 44318_2026_818_MOESM9_ESM.zip › Figure 5/Figure 5K/Inset-5XFAD+AAV.tif]

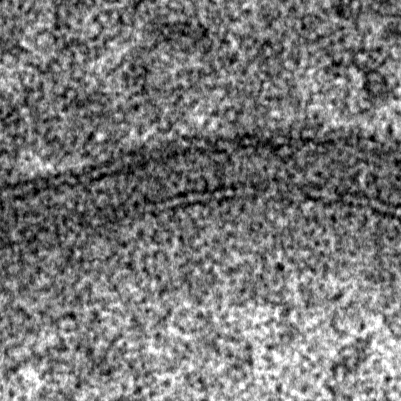

Supplement: Supplementary file 9 — Source data Fig. 5 [file 44318_2026_818_MOESM9_ESM.zip › Figure 5/Figure 5K/Inset-5XFAD+FAM134BmutLIR.tif]

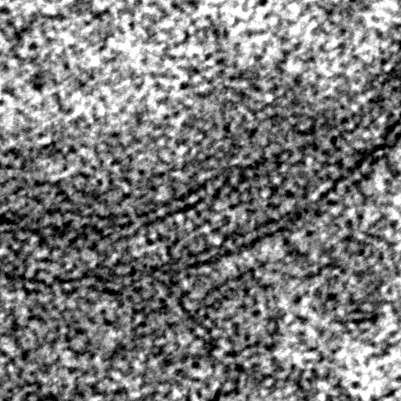

Supplement: Supplementary file 9 — Source data Fig. 5 [file 44318_2026_818_MOESM9_ESM.zip › Figure 5/Figure 5K/Inset-5XFAD+FAM134BWT.tif]

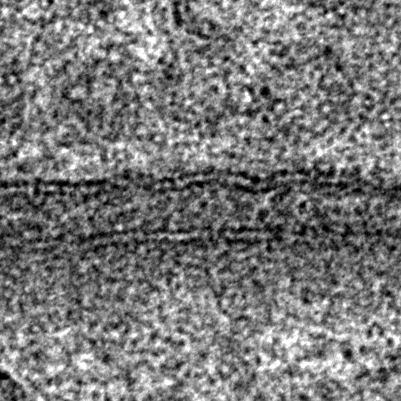

Supplement: Supplementary file 9 — Source data Fig. 5 [file 44318_2026_818_MOESM9_ESM.zip › Figure 5/Figure 5K/Inset-WT+AAV.tif]

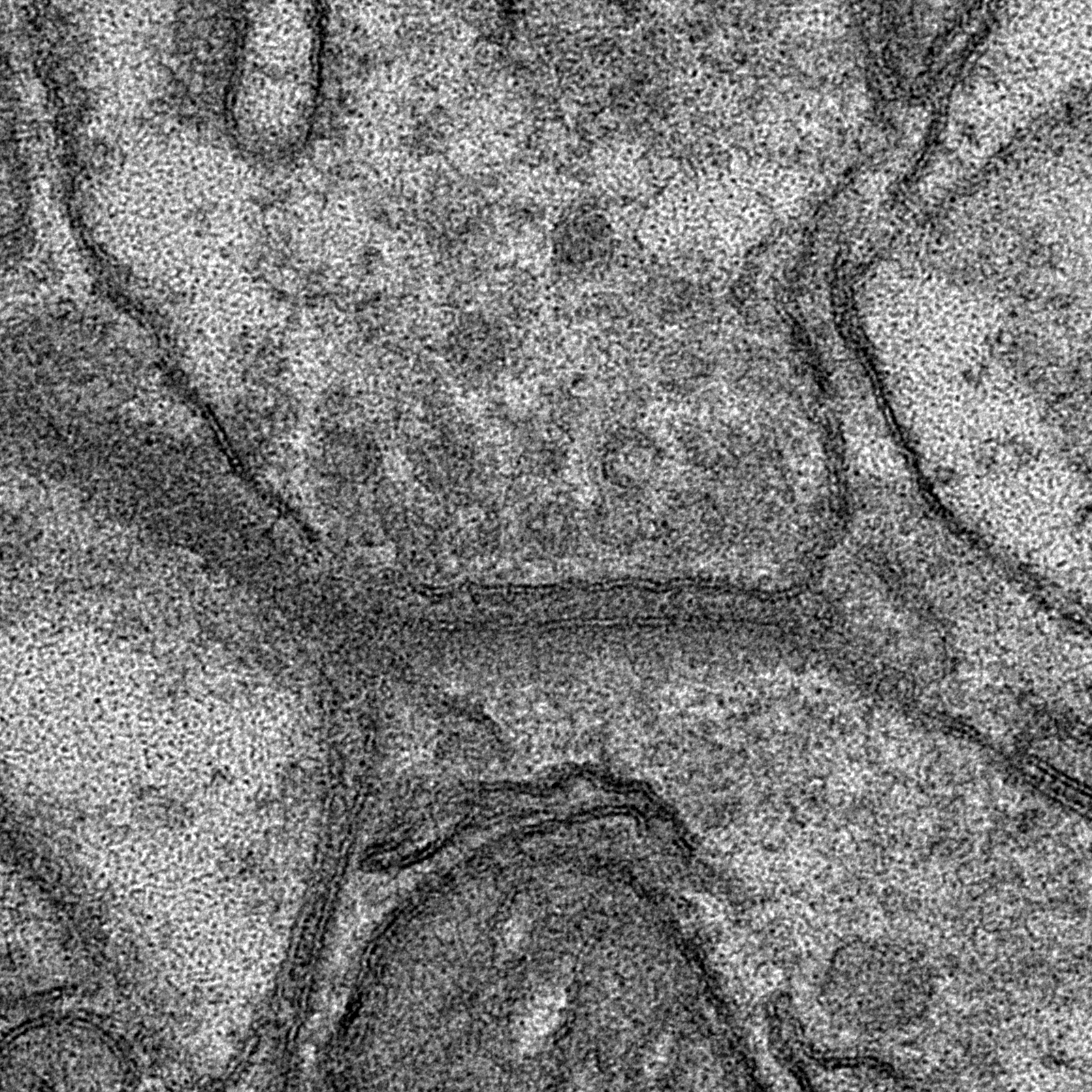

Supplement: Supplementary file 9 — Source data Fig. 5 [file 44318_2026_818_MOESM9_ESM.zip › Figure 5/Figure 5K/WT+AAV.tif]

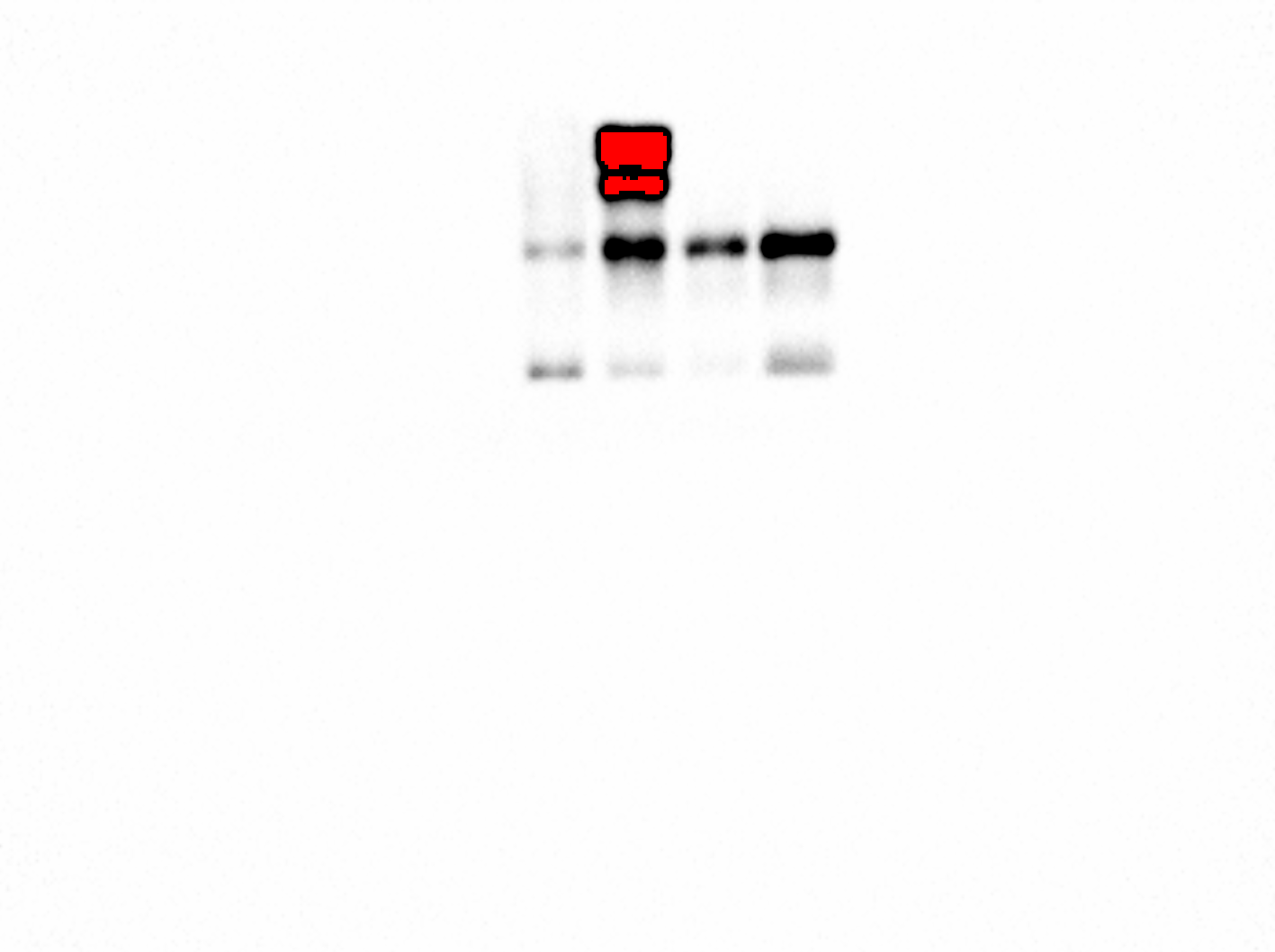

Supplement: Supplementary file 10 — Source data Fig. 6 [file 44318_2026_818_MOESM10_ESM.zip › Figure 6/Figure 6A/APP.tif]

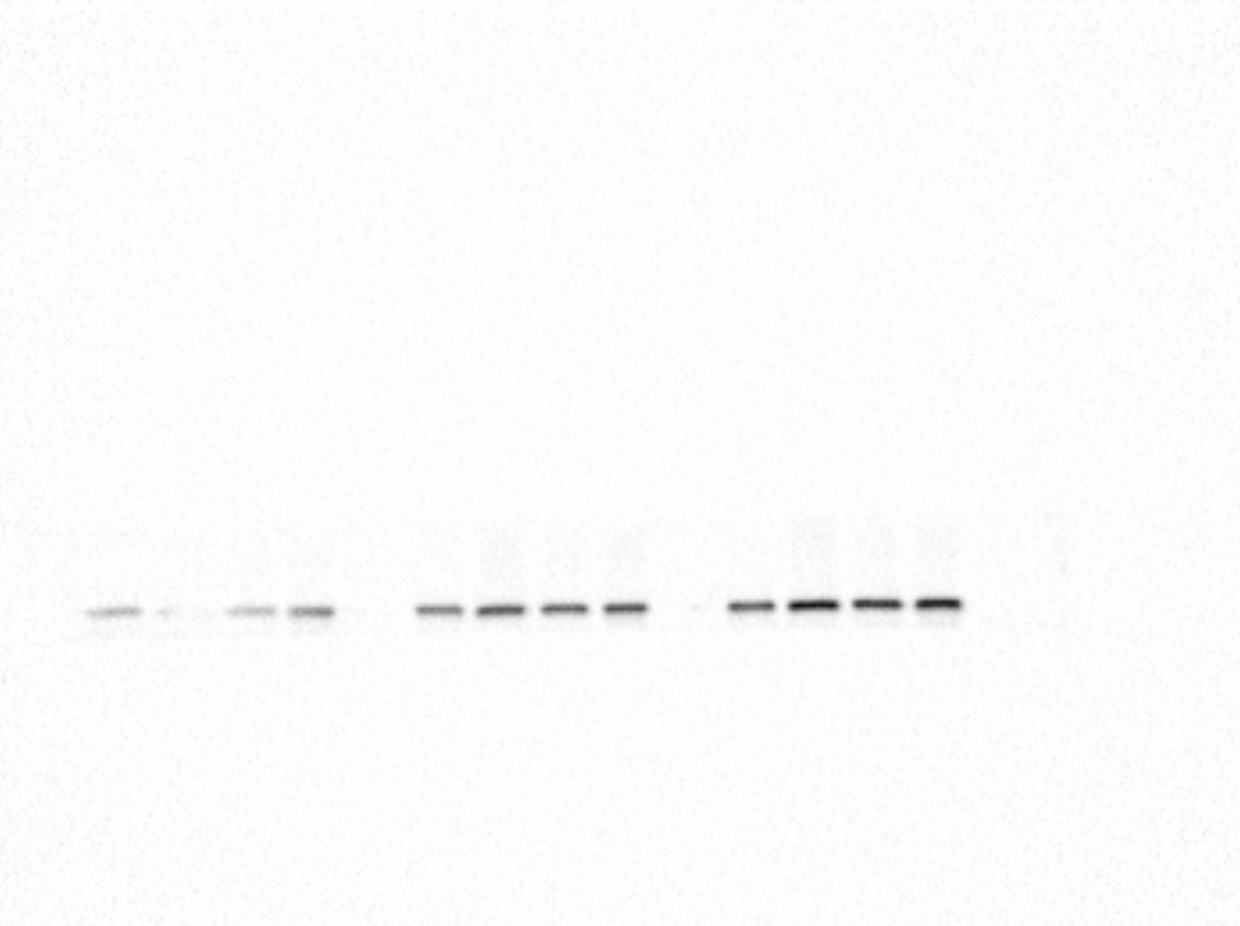

Supplement: Supplementary file 10 — Source data Fig. 6 [file 44318_2026_818_MOESM10_ESM.zip › Figure 6/Figure 6A/Calnexin (lane 9-12).tif]

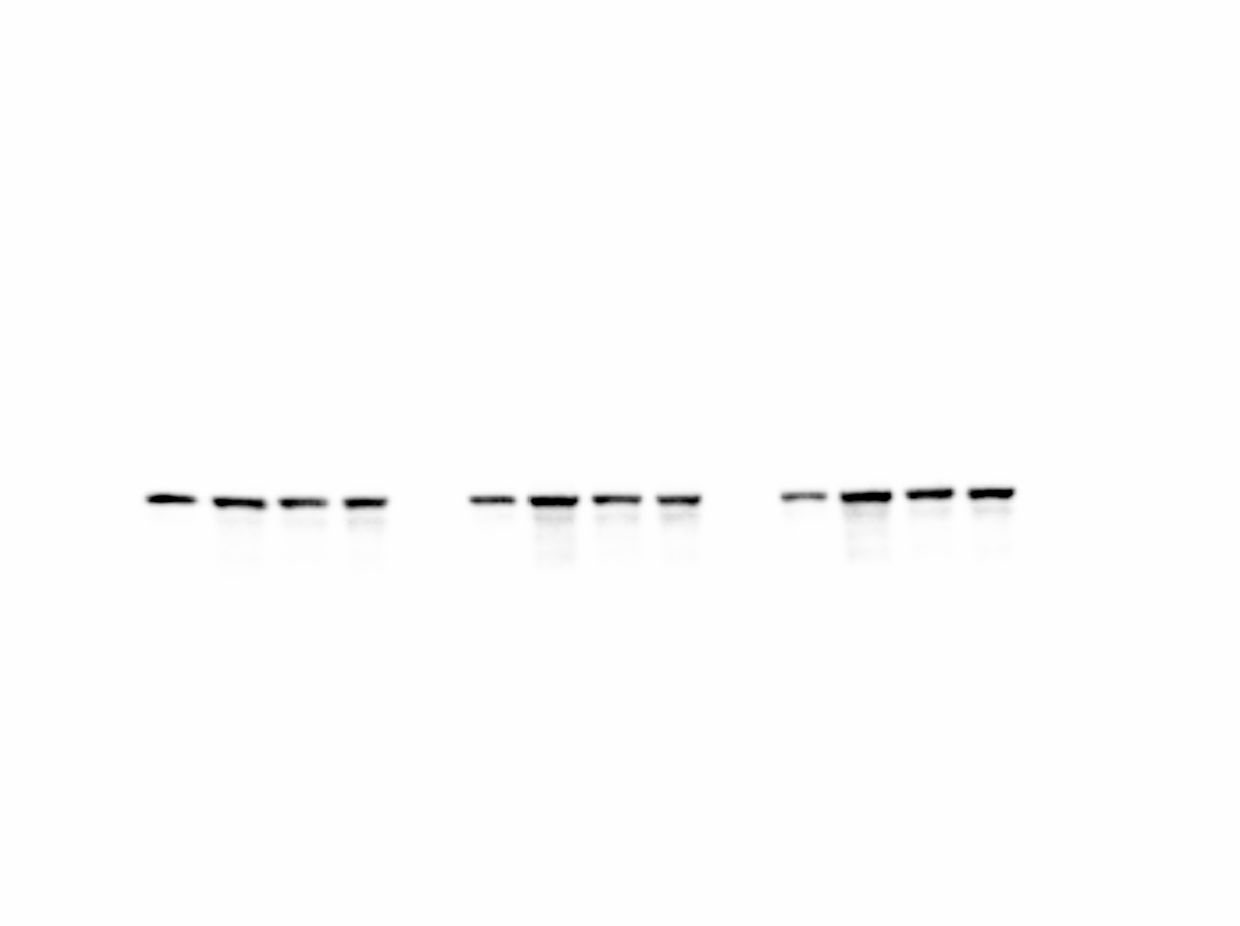

Supplement: Supplementary file 10 — Source data Fig. 6 [file 44318_2026_818_MOESM10_ESM.zip › Figure 6/Figure 6A/Climp63 (lane 5-8).tif]

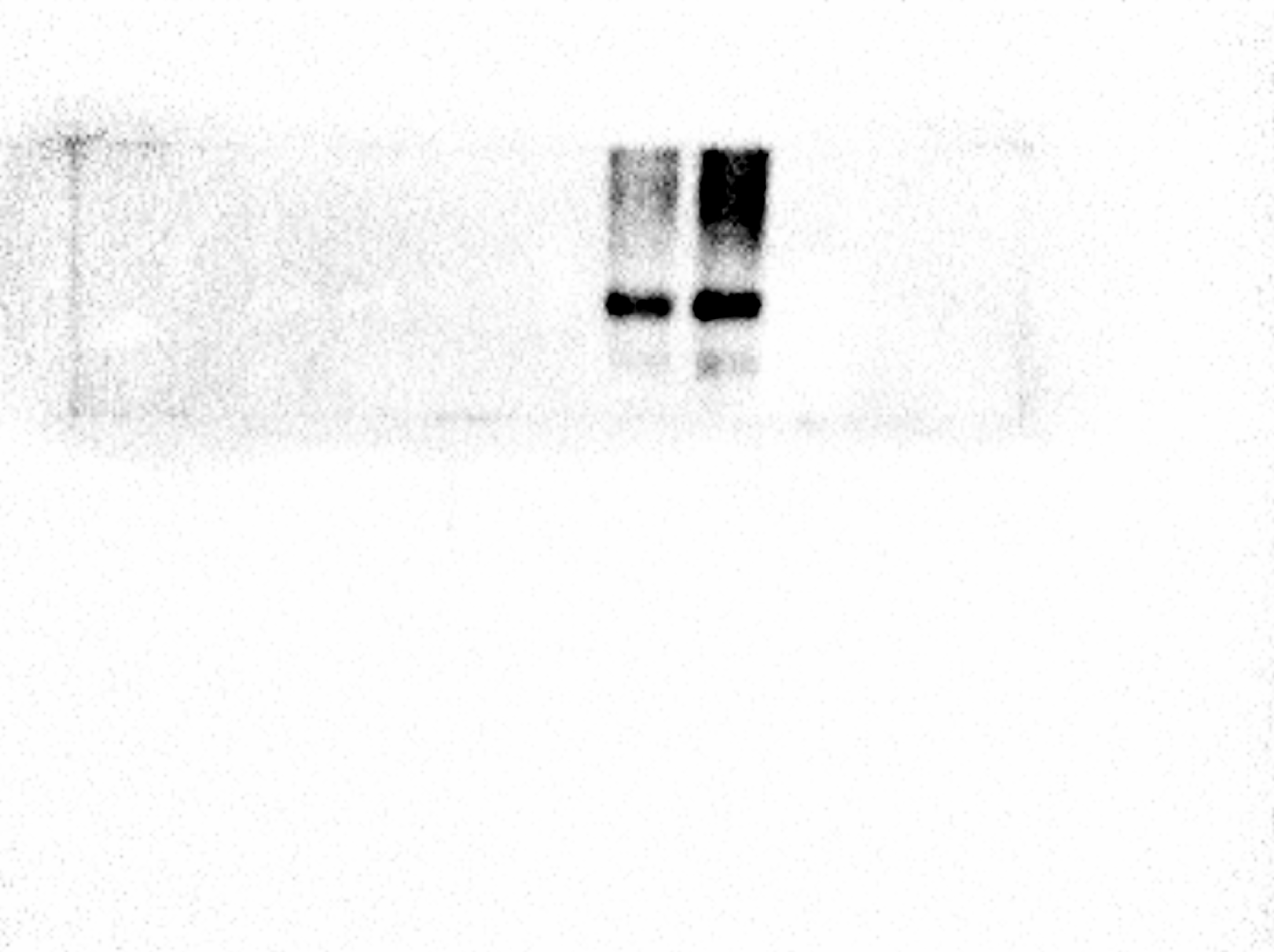

Supplement: Supplementary file 10 — Source data Fig. 6 [file 44318_2026_818_MOESM10_ESM.zip › Figure 6/Figure 6A/Fam134b.tif]

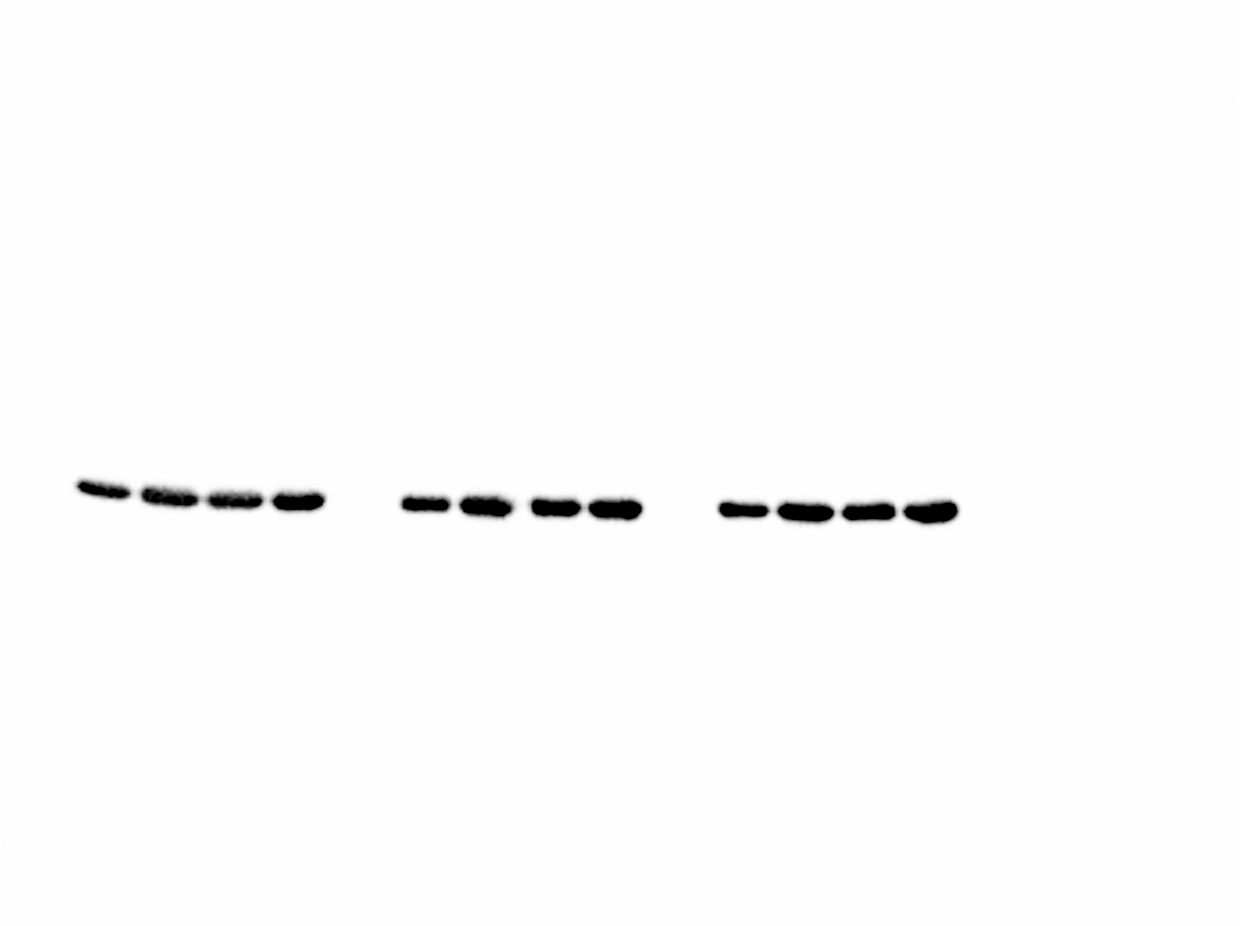

Supplement: Supplementary file 10 — Source data Fig. 6 [file 44318_2026_818_MOESM10_ESM.zip › Figure 6/Figure 6A/Gapdh (lane 5-8).tif]

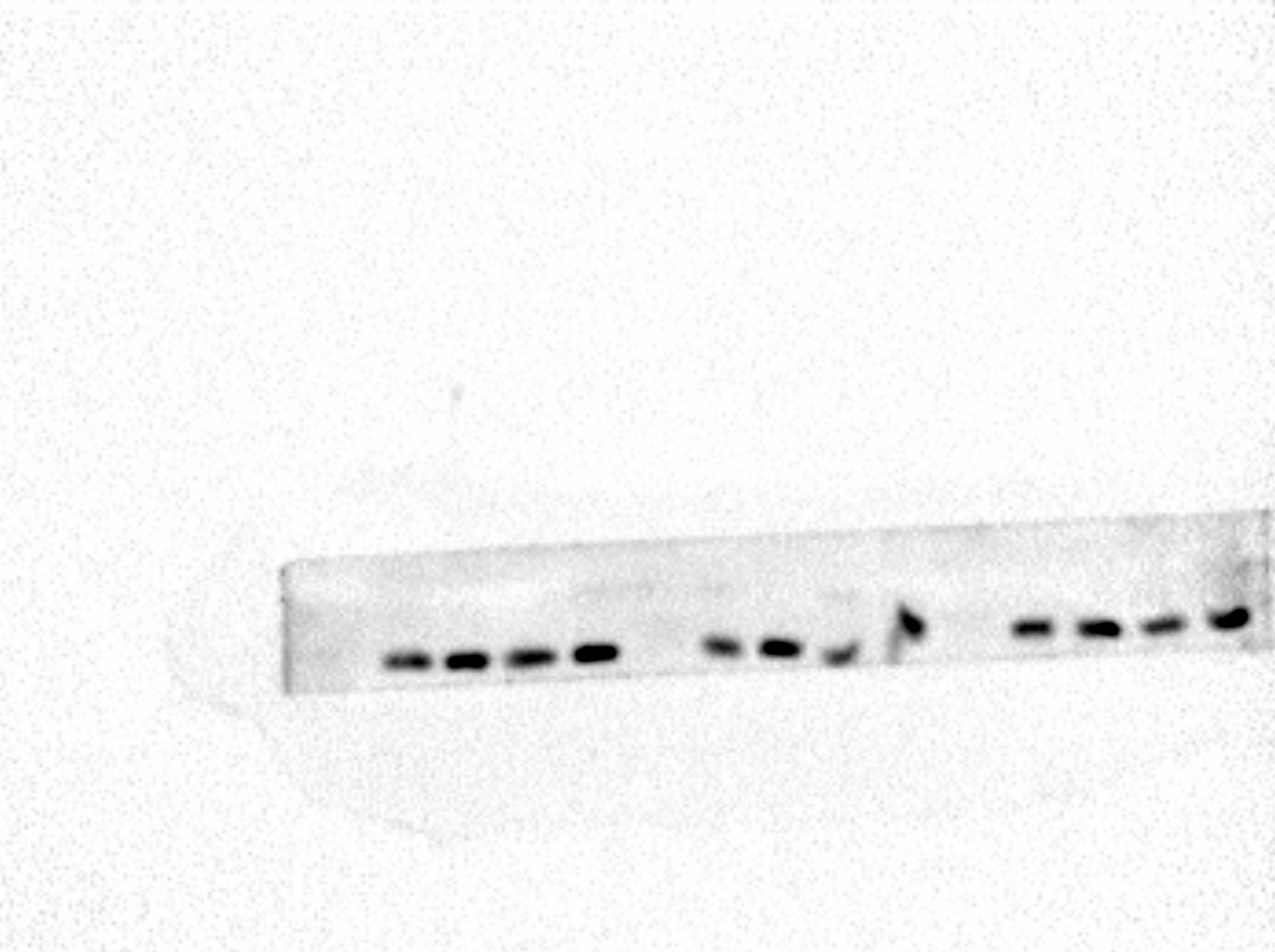

Supplement: Supplementary file 10 — Source data Fig. 6 [file 44318_2026_818_MOESM10_ESM.zip › Figure 6/Figure 6A/Reep5 (lane 1-4).tif]

Figure 6A

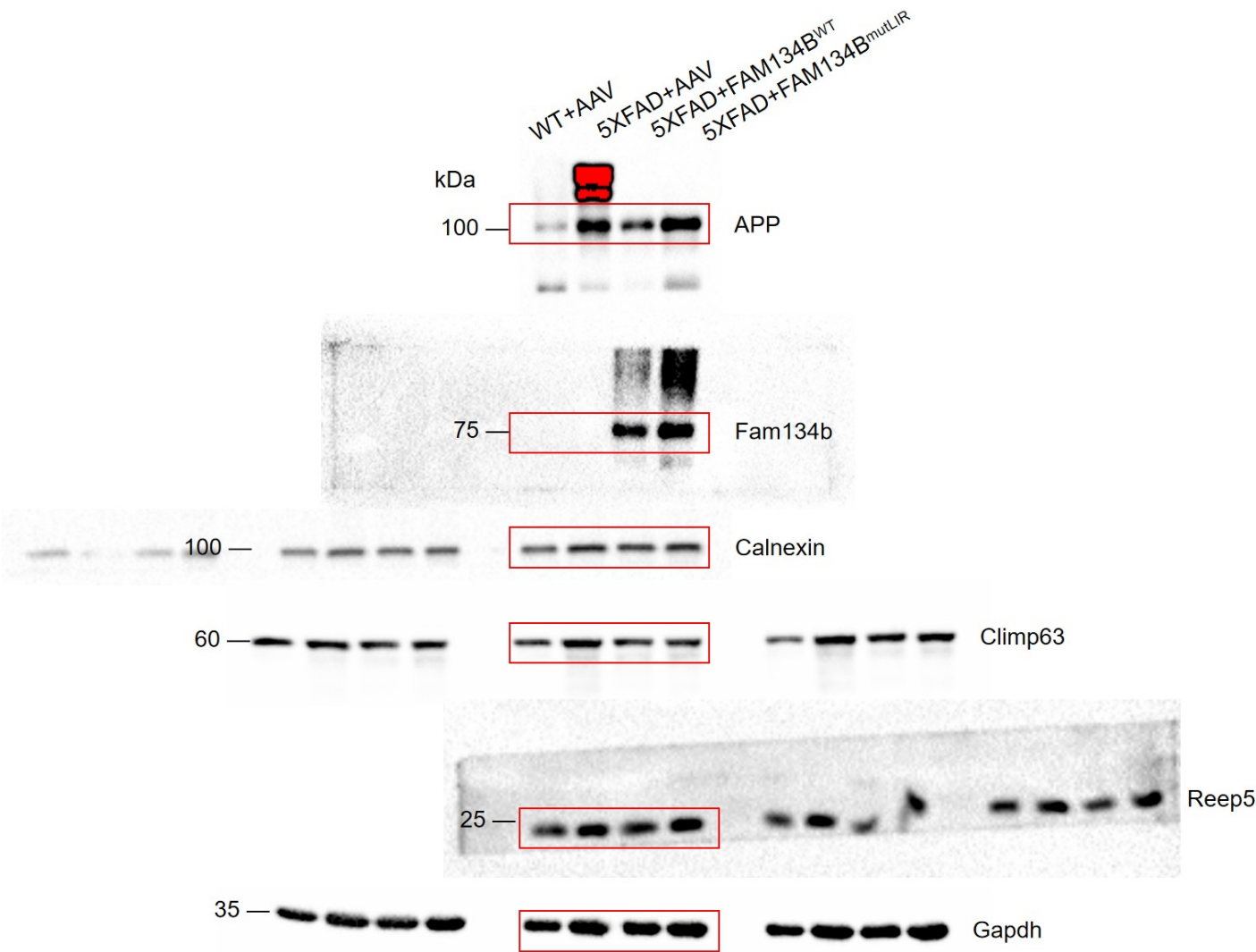

Supplement: Supplementary file 10 — Source data Fig. 6 [file 44318_2026_818_MOESM10_ESM.zip › Figure 6/Figure 6A/WB for Figure 6A.pdf]

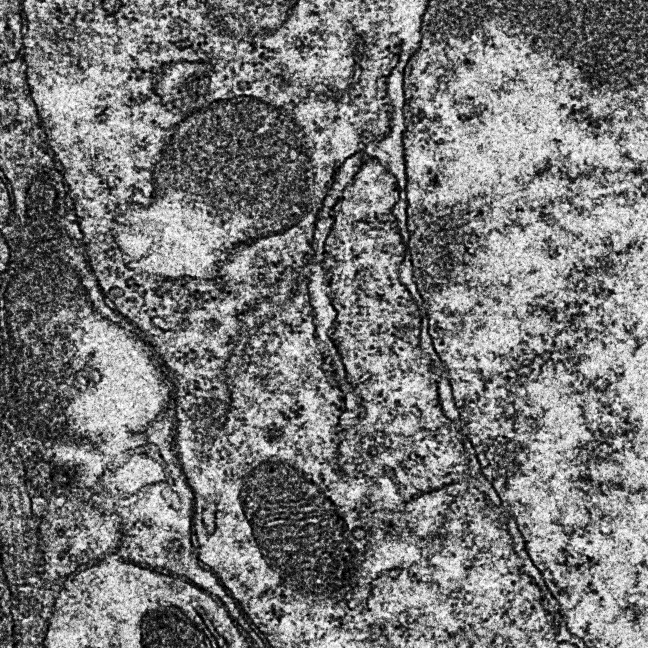

Supplement: Supplementary file 10 — Source data Fig. 6 [file 44318_2026_818_MOESM10_ESM.zip › Figure 6/Figure 6B/Inset-WT+AAV.tif]

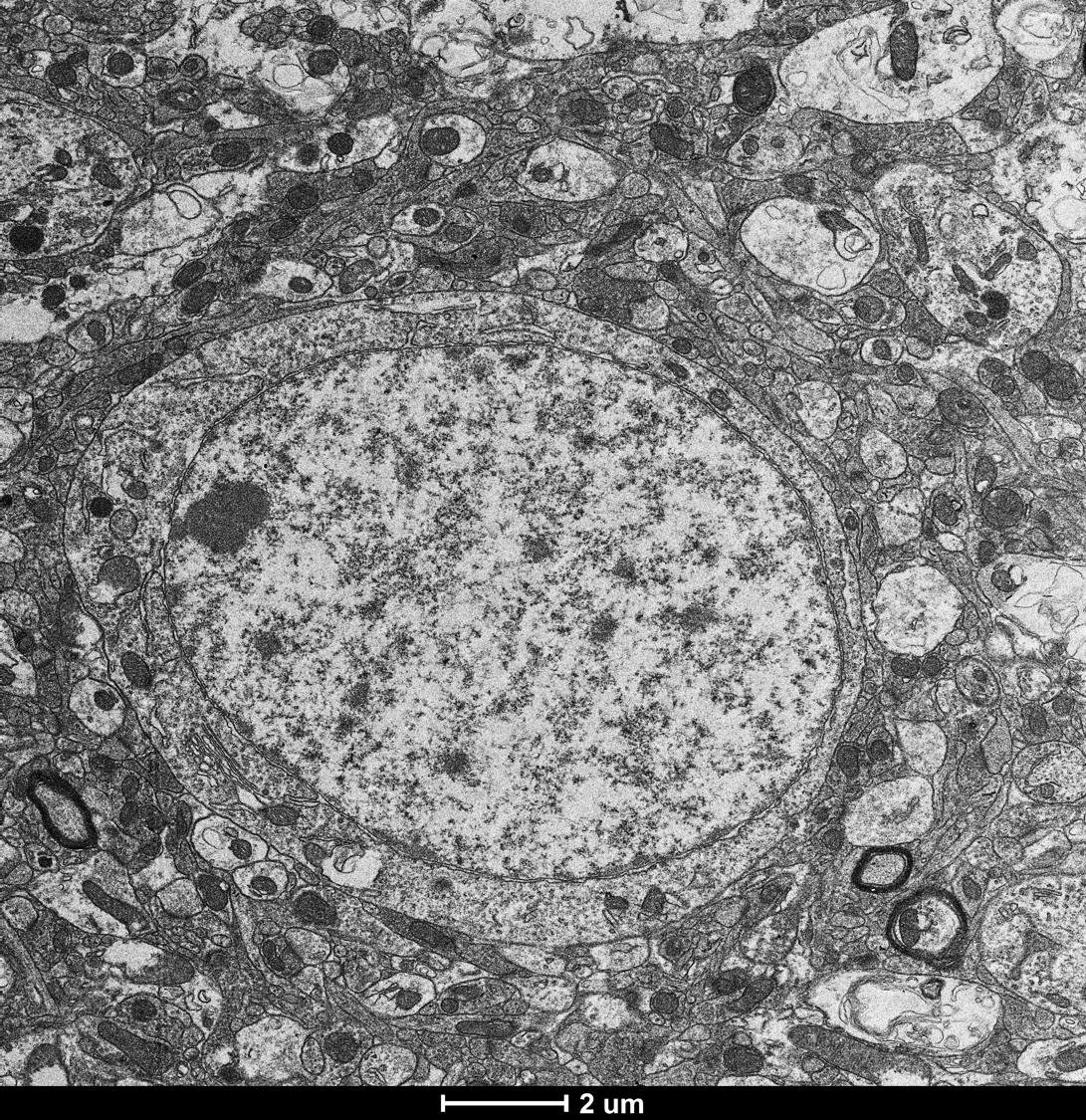

Supplement: Supplementary file 10 — Source data Fig. 6 [file 44318_2026_818_MOESM10_ESM.zip › Figure 6/Figure 6B/WT+AAV.tif]

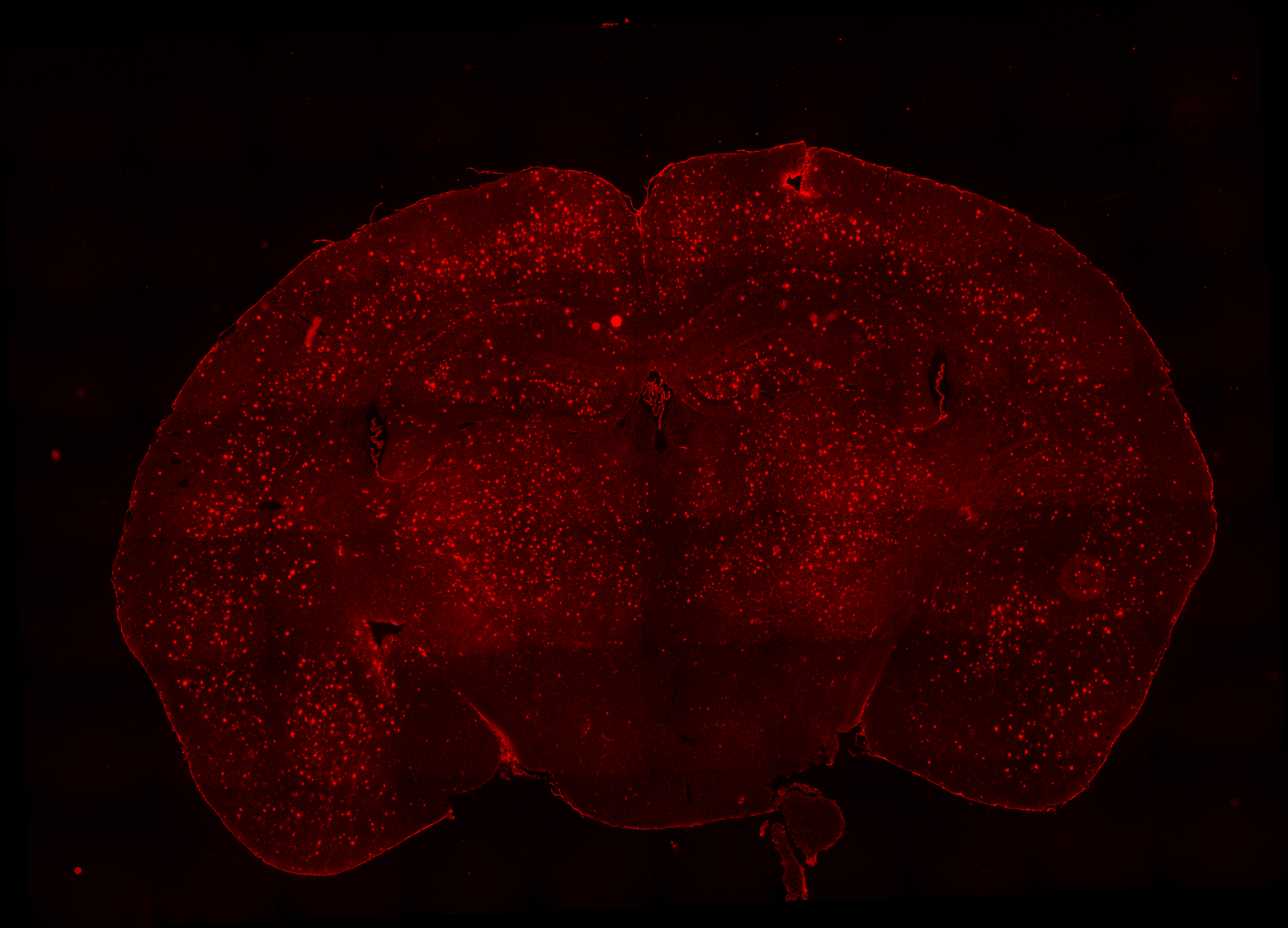

Supplement: Supplementary file 10 — Source data Fig. 6 [file 44318_2026_818_MOESM10_ESM.zip › Figure 6/Figure 6C/5XFAD+AAV/Aβ.tif]

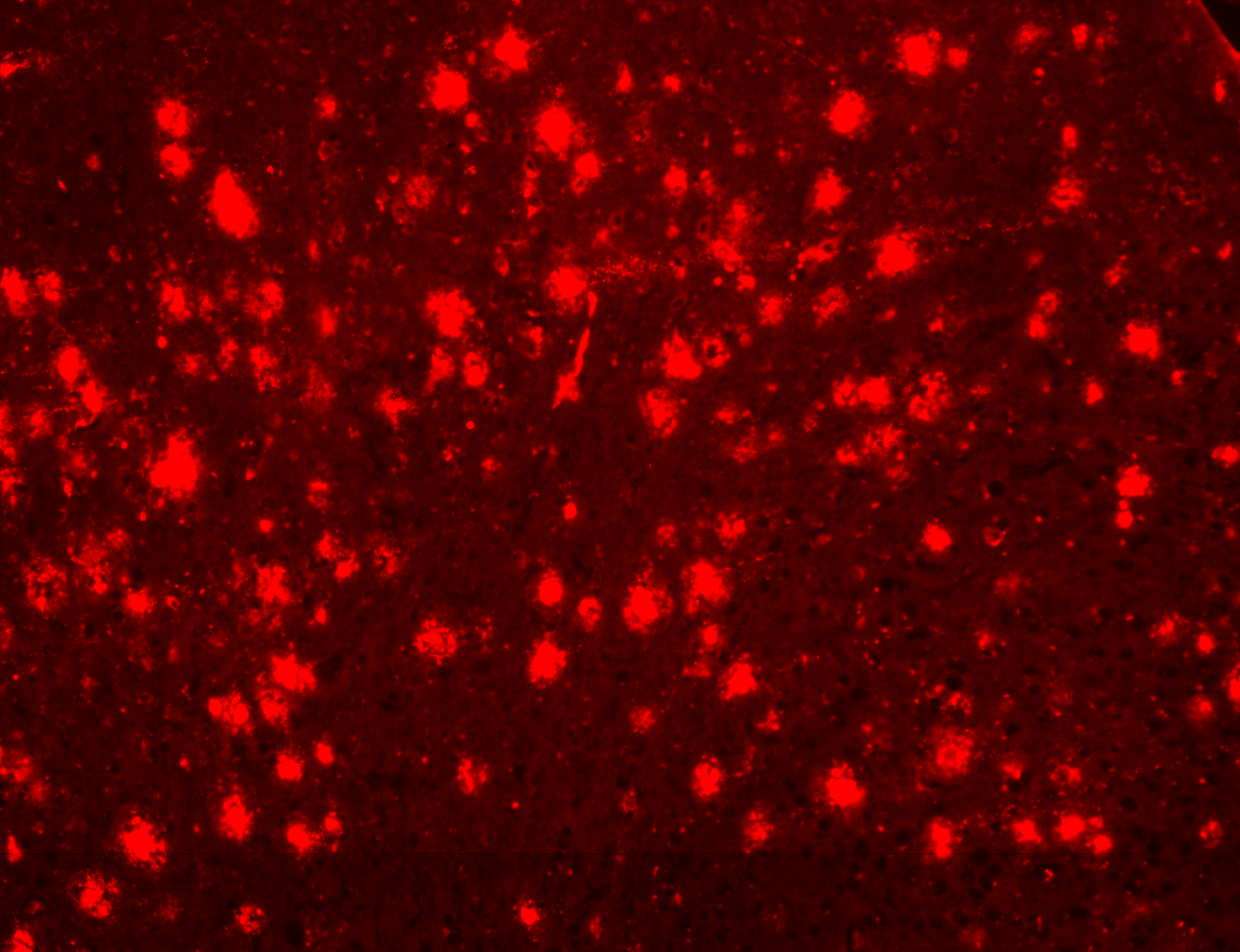

Supplement: Supplementary file 10 — Source data Fig. 6 [file 44318_2026_818_MOESM10_ESM.zip › Figure 6/Figure 6C/5XFAD+AAV/Cortex-Aβ.tif]

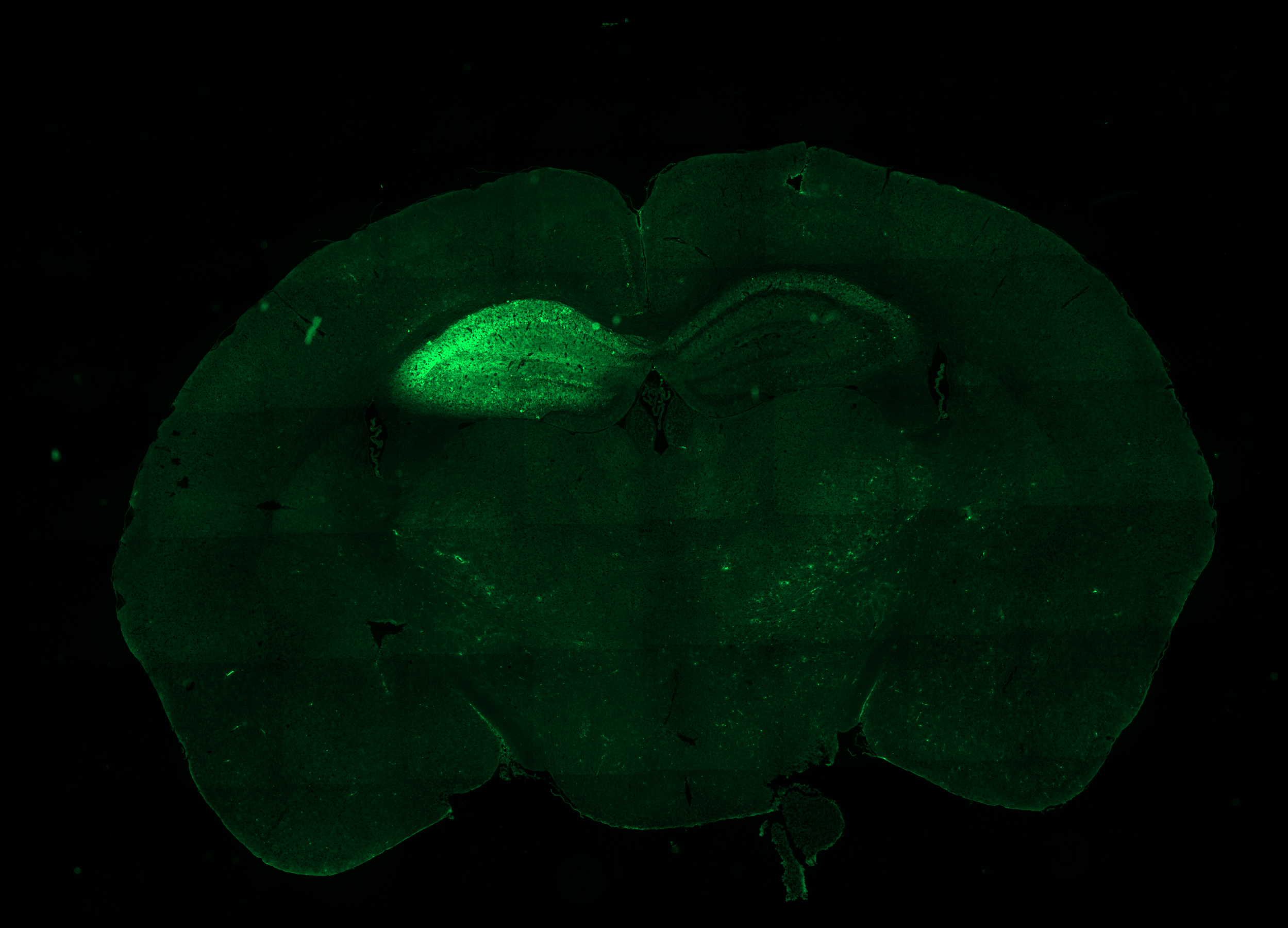

Supplement: Supplementary file 10 — Source data Fig. 6 [file 44318_2026_818_MOESM10_ESM.zip › Figure 6/Figure 6C/5XFAD+AAV/EGFP-FAM134B.tiff]

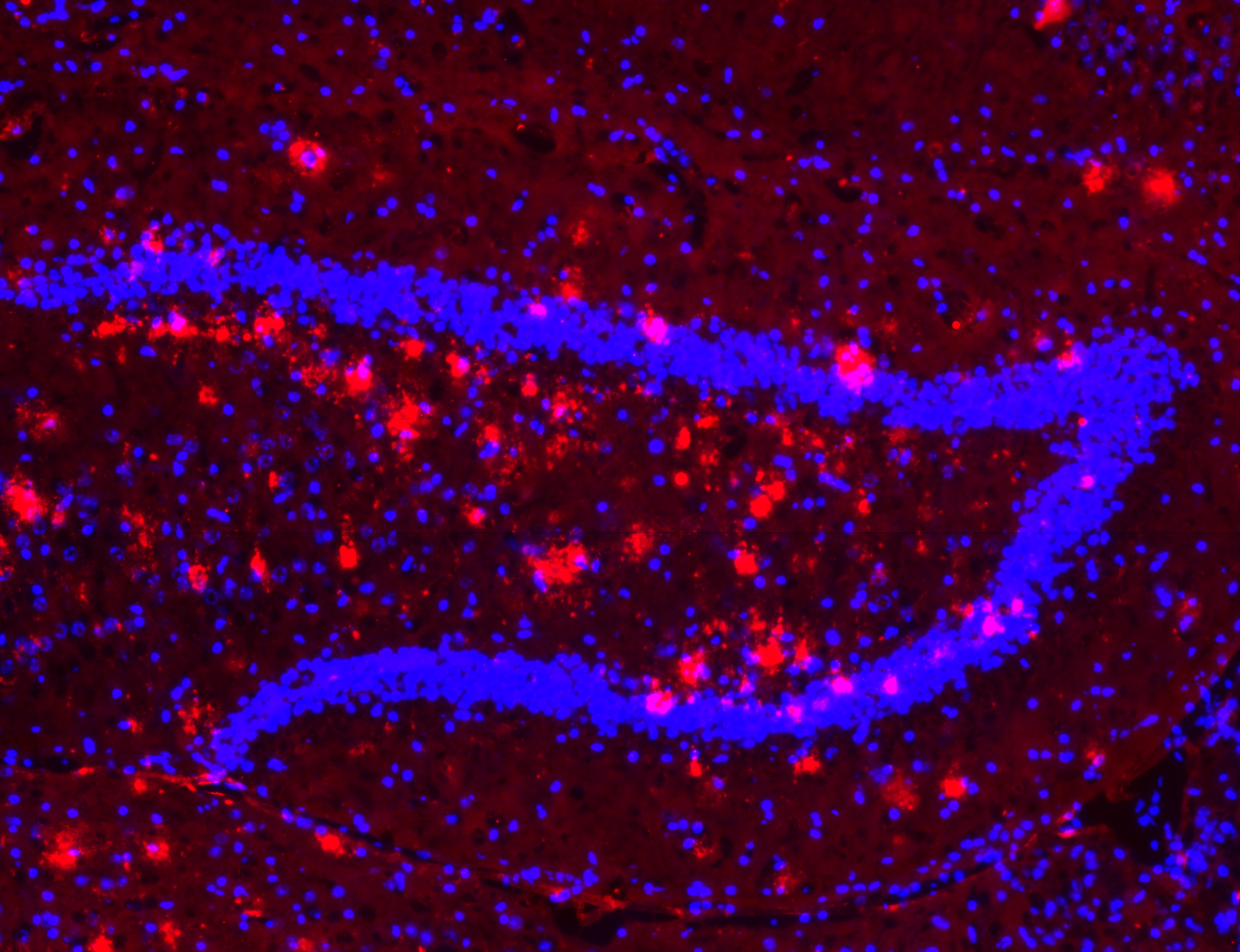

Supplement: Supplementary file 10 — Source data Fig. 6 [file 44318_2026_818_MOESM10_ESM.zip › Figure 6/Figure 6C/5XFAD+AAV/Hippocampus-Aβ.tif]

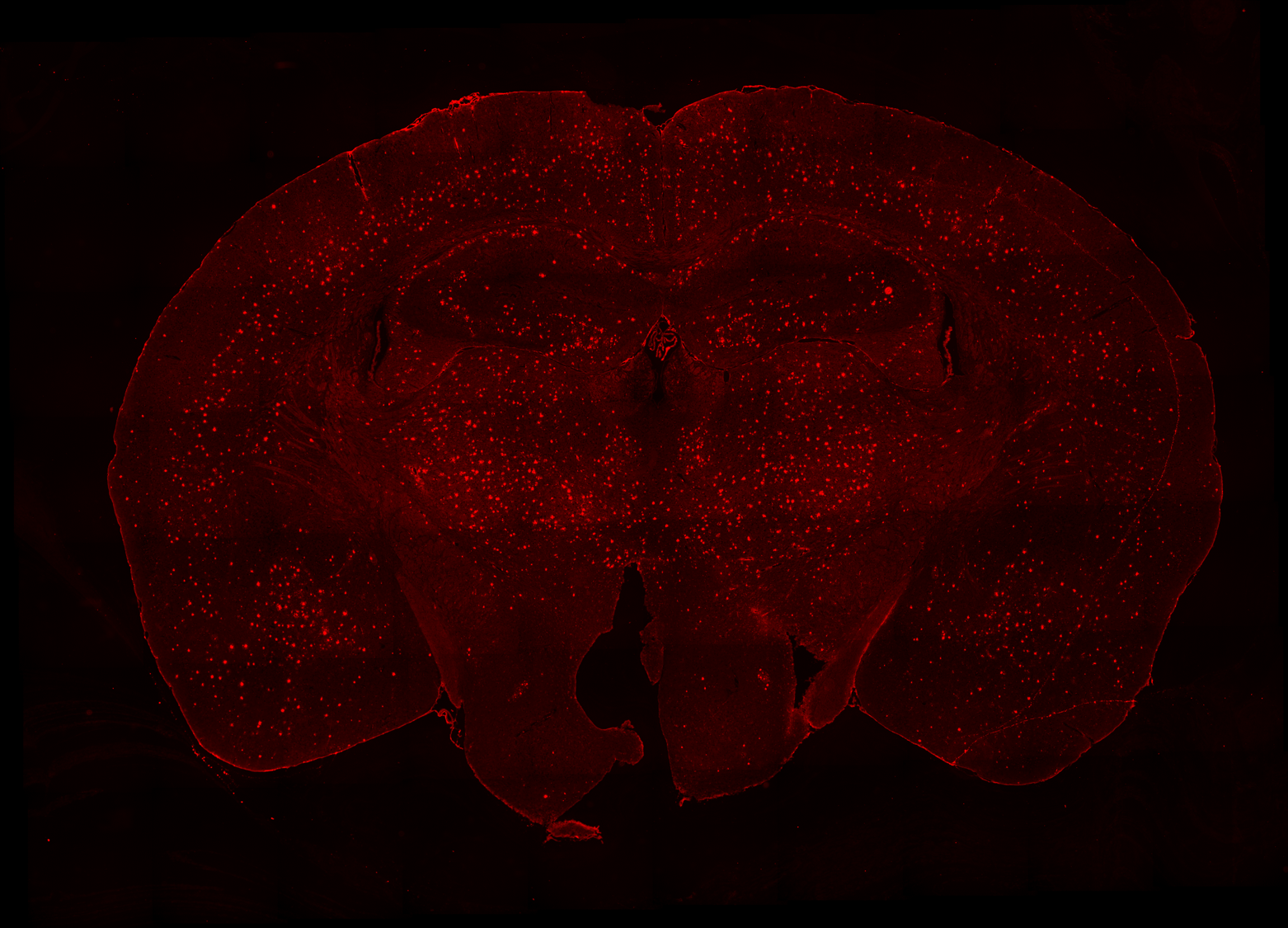

Supplement: Supplementary file 10 — Source data Fig. 6 [file 44318_2026_818_MOESM10_ESM.zip › Figure 6/Figure 6C/5XFAD+FAM134BmutLIR/Aβ.tif]

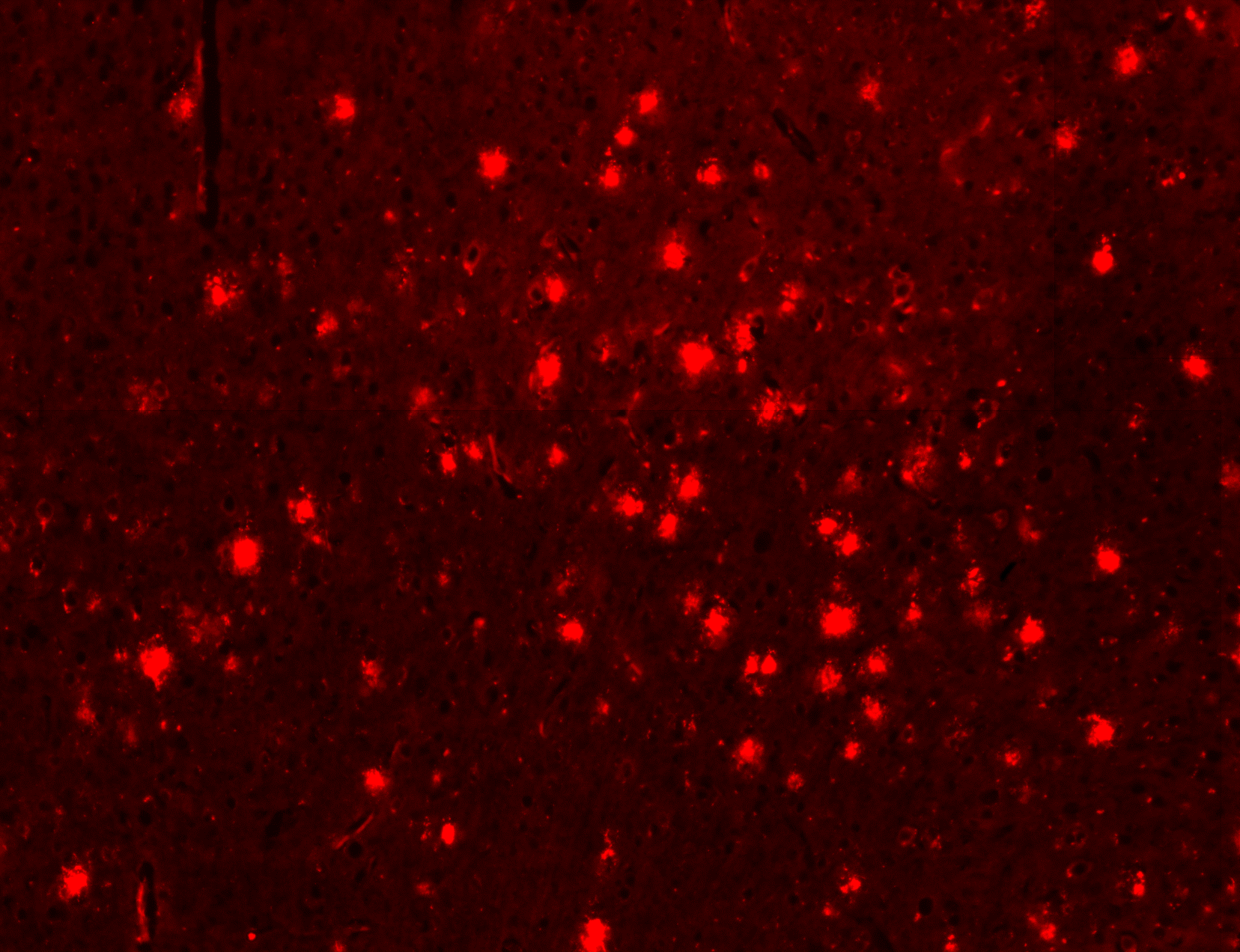

Supplement: Supplementary file 10 — Source data Fig. 6 [file 44318_2026_818_MOESM10_ESM.zip › Figure 6/Figure 6C/5XFAD+FAM134BmutLIR/Cortex-Aβ.tif]

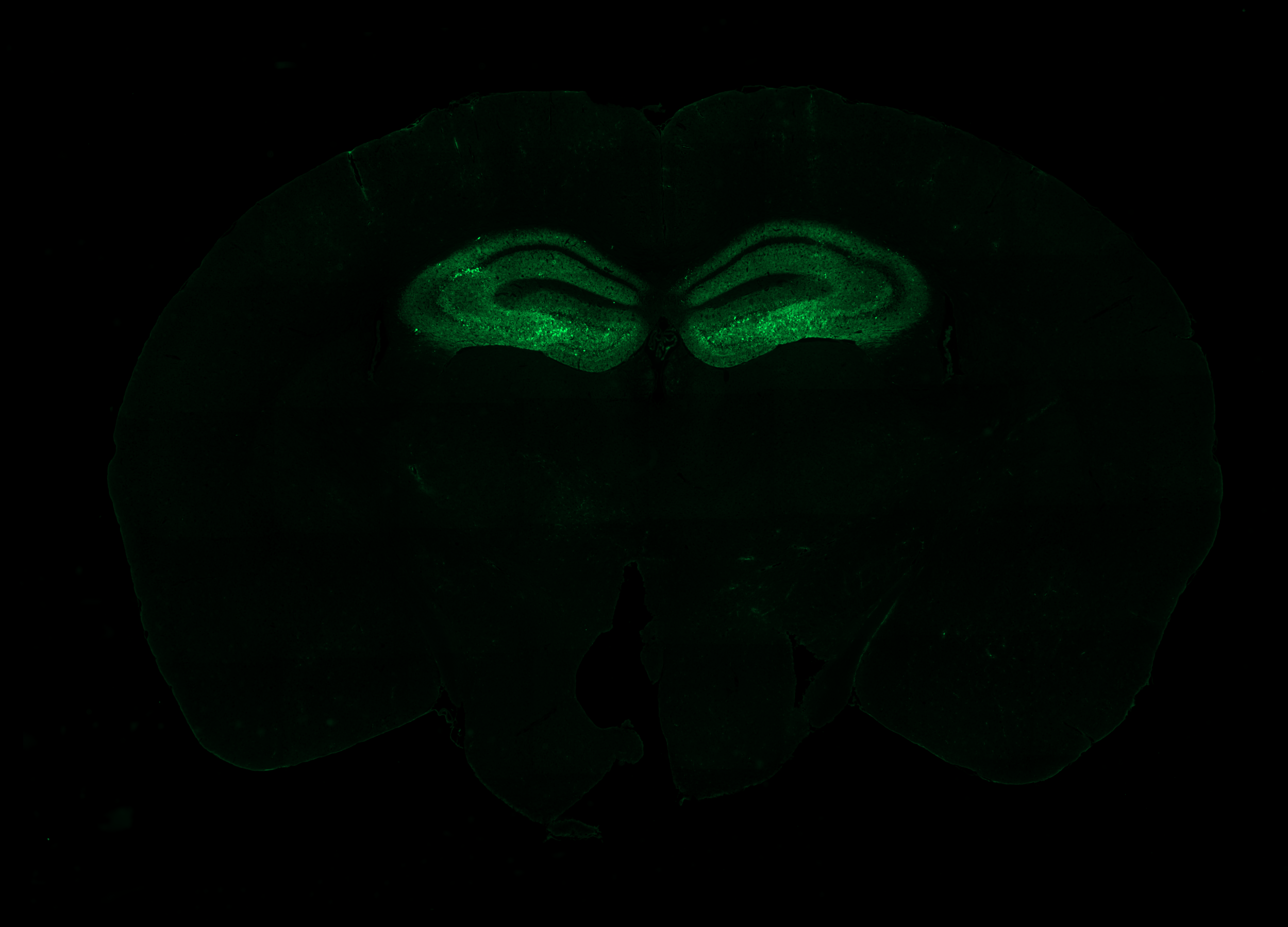

Supplement: Supplementary file 10 — Source data Fig. 6 [file 44318_2026_818_MOESM10_ESM.zip › Figure 6/Figure 6C/5XFAD+FAM134BmutLIR/EGFP-FAM134B.tiff]

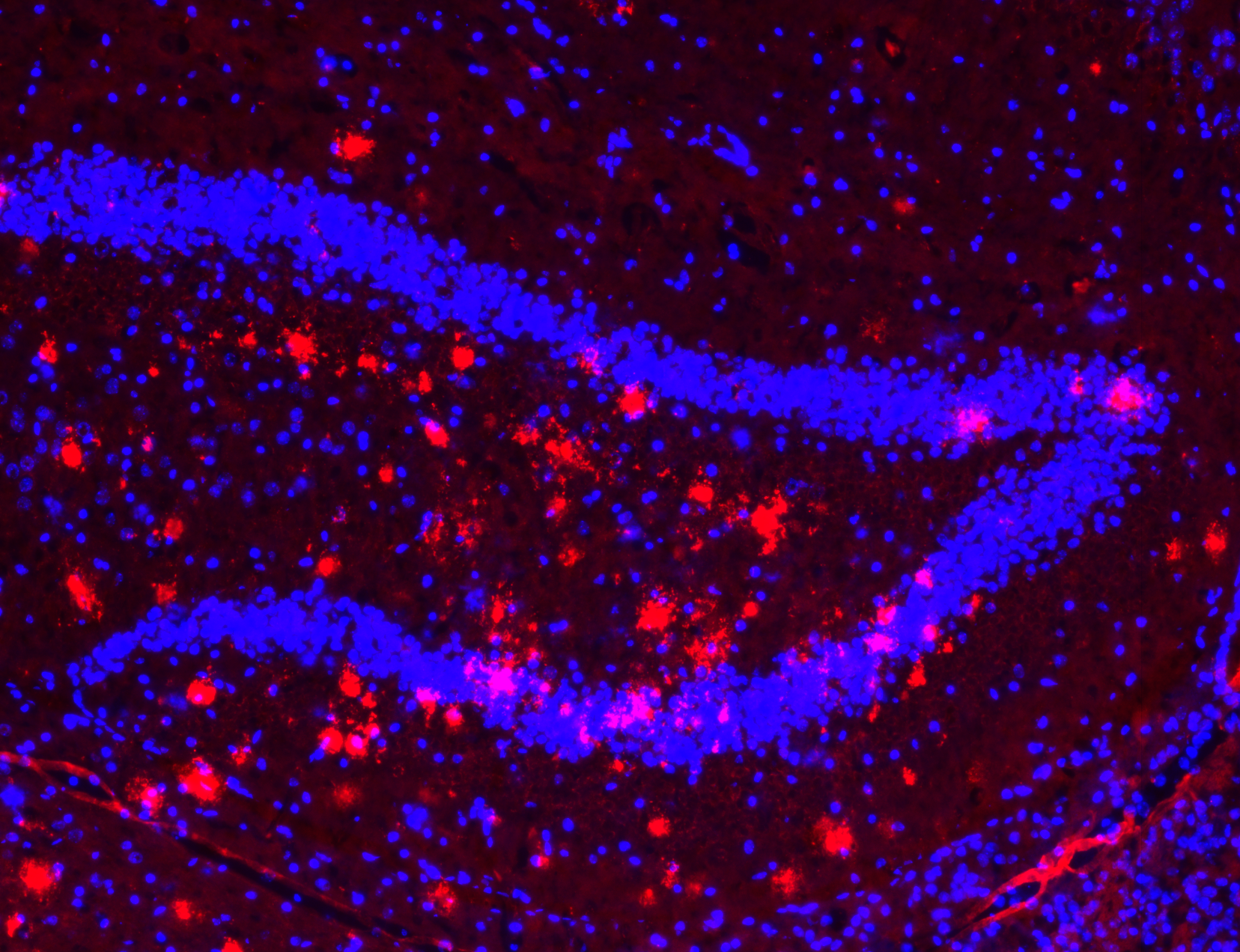

Supplement: Supplementary file 10 — Source data Fig. 6 [file 44318_2026_818_MOESM10_ESM.zip › Figure 6/Figure 6C/5XFAD+FAM134BmutLIR/Hippocampus-Aβ.tif]

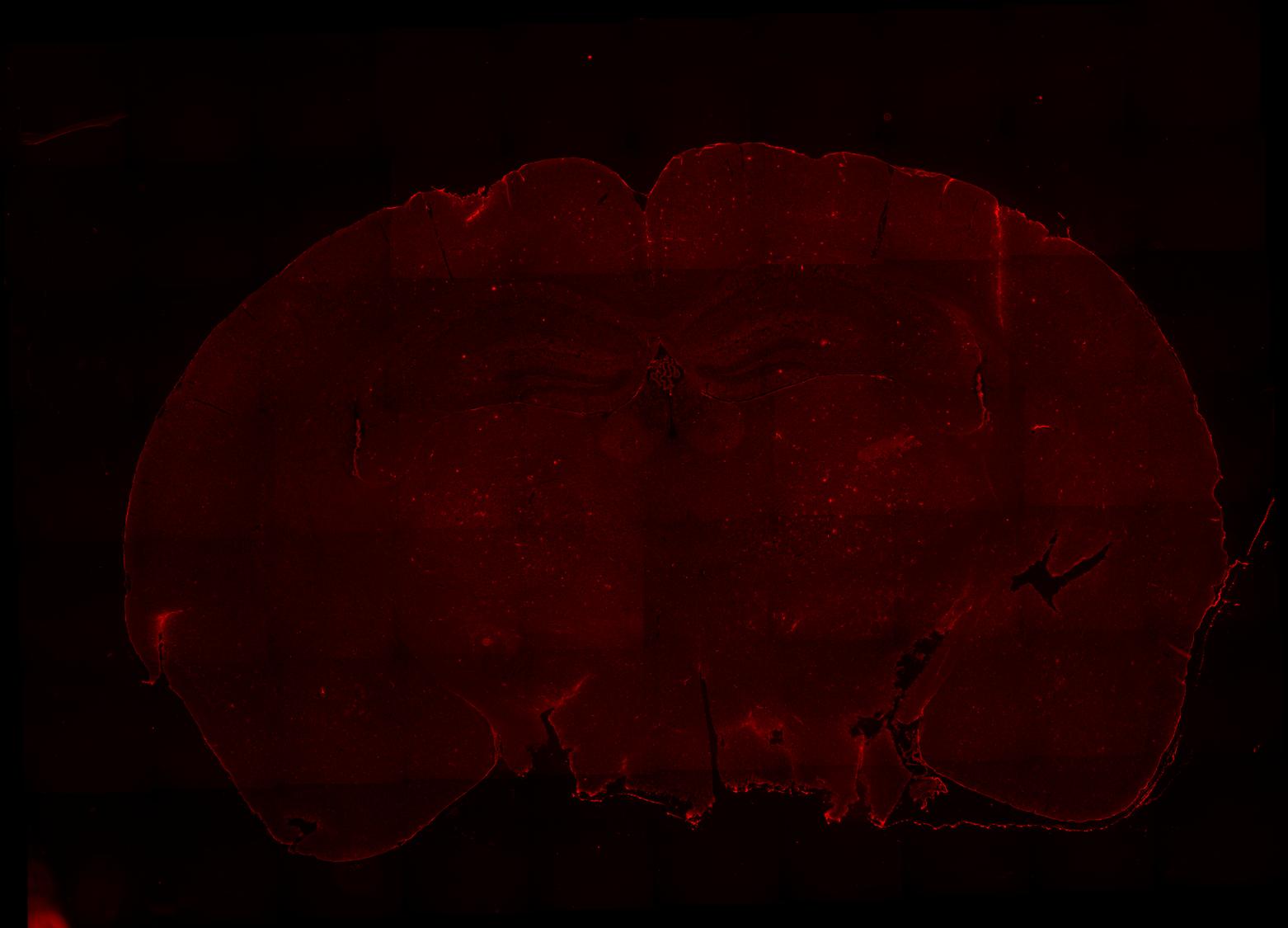

Supplement: Supplementary file 10 — Source data Fig. 6 [file 44318_2026_818_MOESM10_ESM.zip › Figure 6/Figure 6C/5XFAD+FAM134BWT/Aβ.tif]

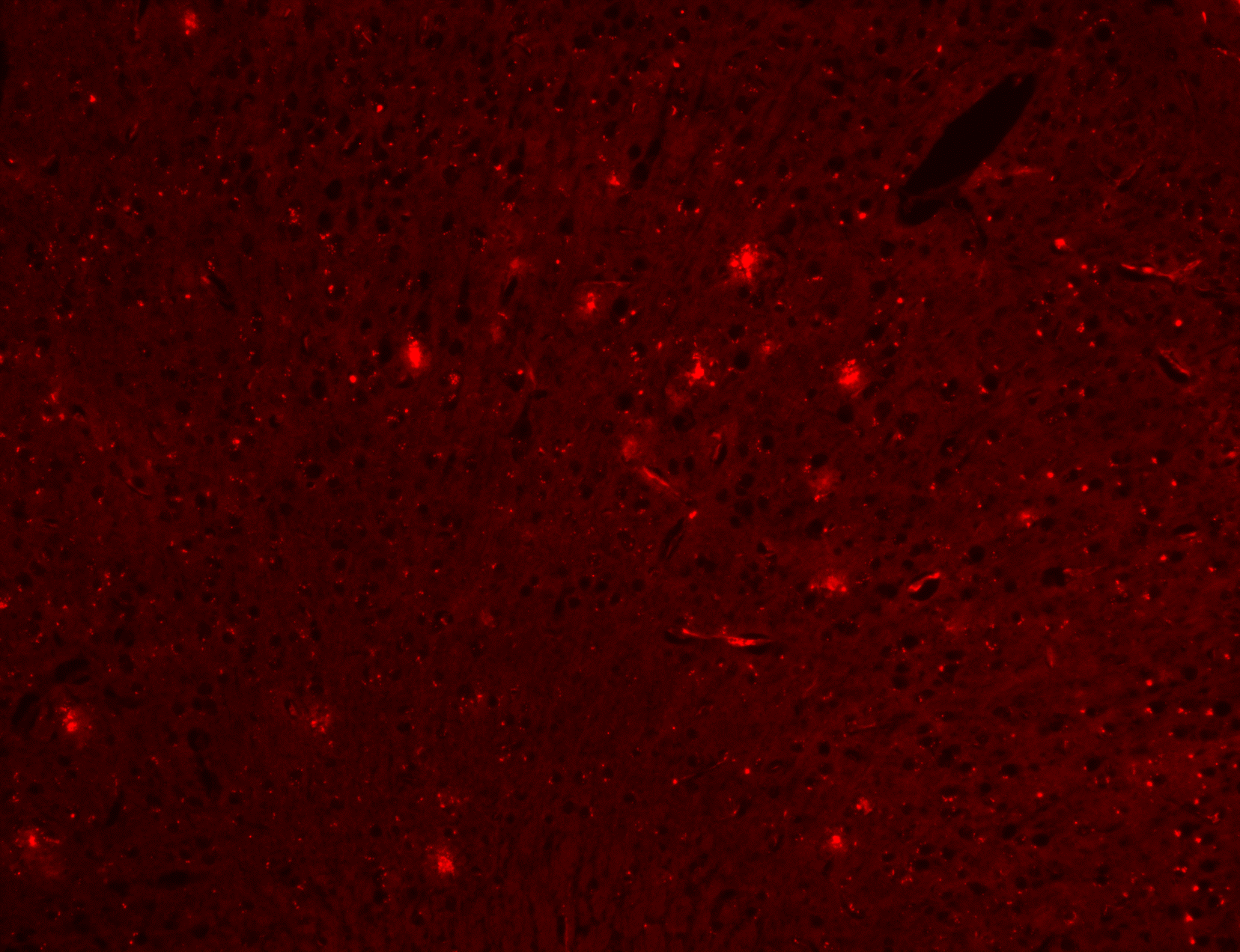

Supplement: Supplementary file 10 — Source data Fig. 6 [file 44318_2026_818_MOESM10_ESM.zip › Figure 6/Figure 6C/5XFAD+FAM134BWT/Cortex-Aβ.tif]

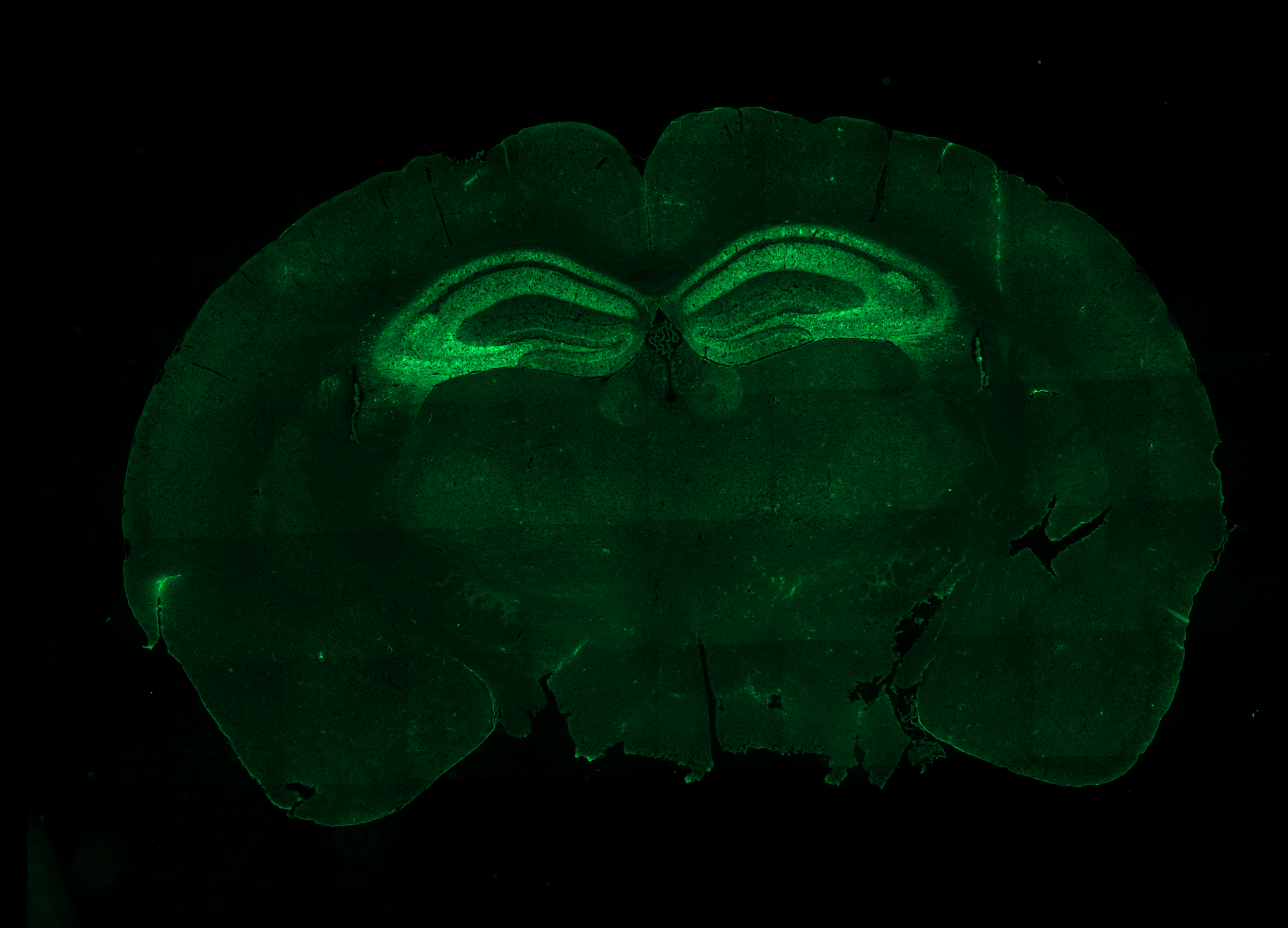

Supplement: Supplementary file 10 — Source data Fig. 6 [file 44318_2026_818_MOESM10_ESM.zip › Figure 6/Figure 6C/5XFAD+FAM134BWT/EGFP-FAM134B.tiff]

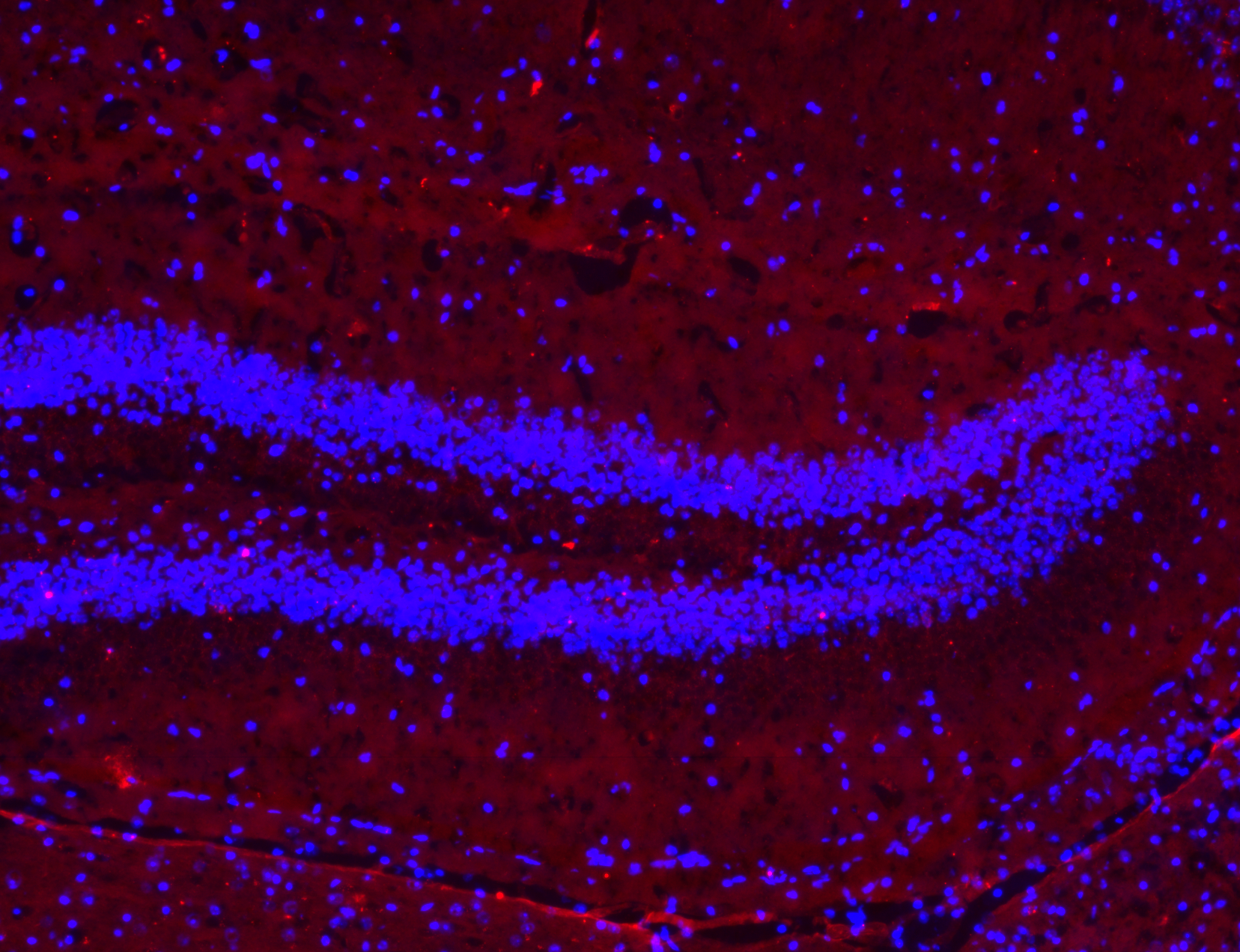

Supplement: Supplementary file 10 — Source data Fig. 6 [file 44318_2026_818_MOESM10_ESM.zip › Figure 6/Figure 6C/5XFAD+FAM134BWT/Hippocampus-Aβ.tif]

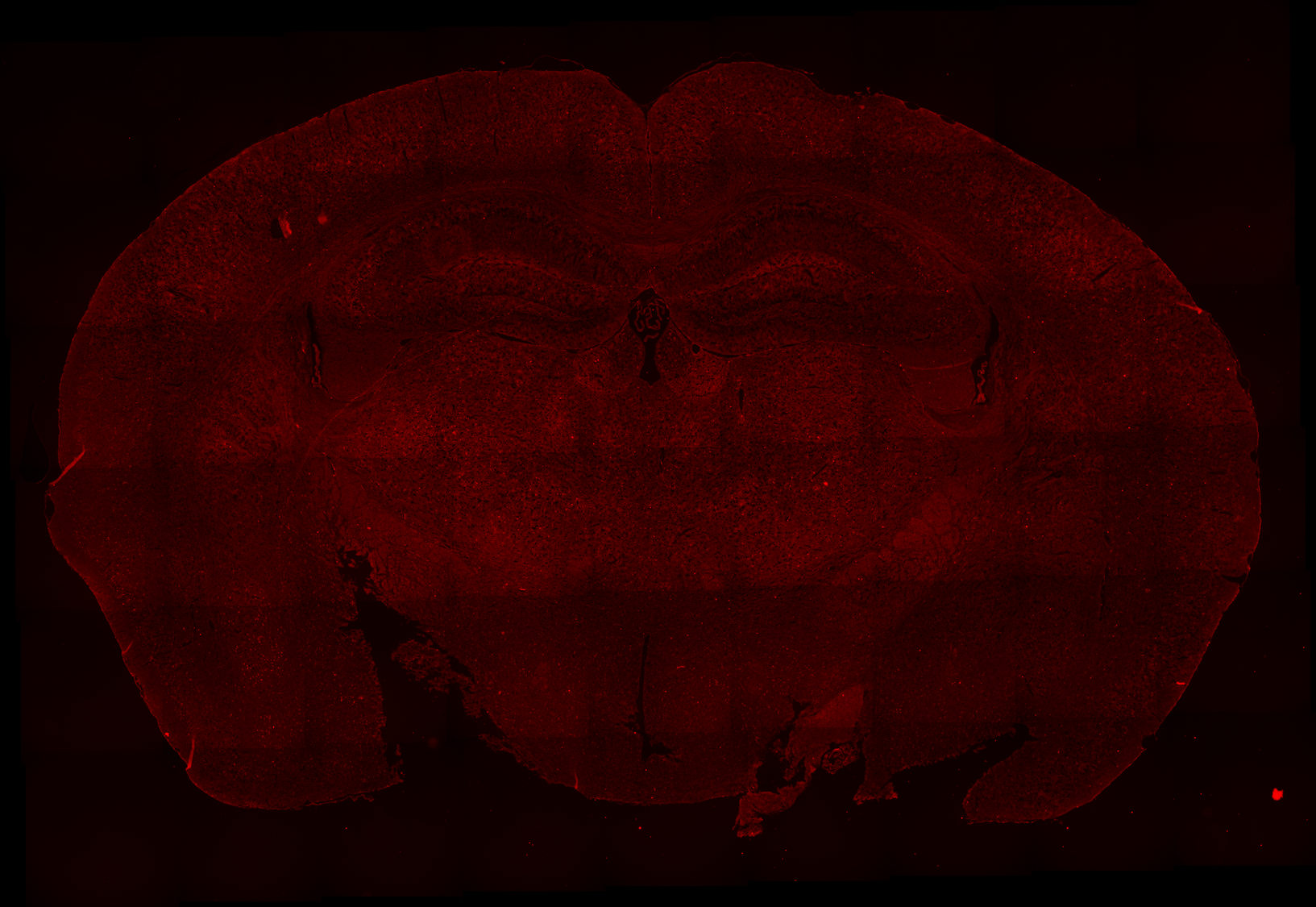

Supplement: Supplementary file 10 — Source data Fig. 6 [file 44318_2026_818_MOESM10_ESM.zip › Figure 6/Figure 6C/WT+AAV/Aβ.tif]

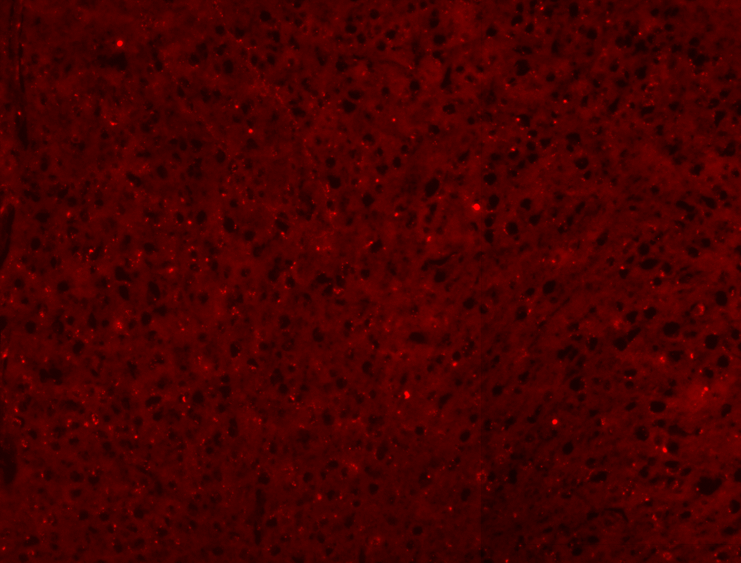

Supplement: Supplementary file 10 — Source data Fig. 6 [file 44318_2026_818_MOESM10_ESM.zip › Figure 6/Figure 6C/WT+AAV/Cortex-Aβ.tif]

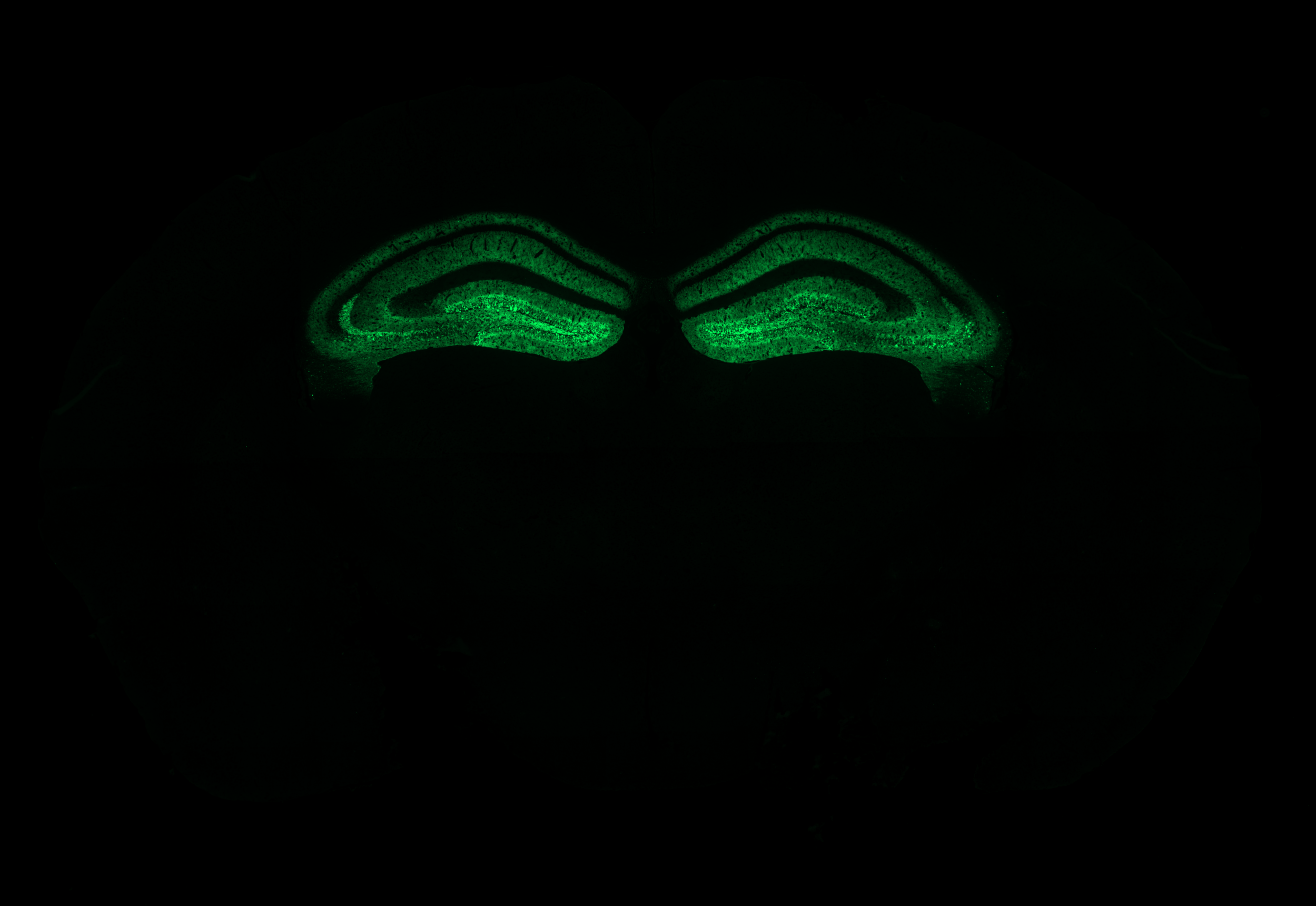

Supplement: Supplementary file 10 — Source data Fig. 6 [file 44318_2026_818_MOESM10_ESM.zip › Figure 6/Figure 6C/WT+AAV/EGFP-FAM134B.tiff]

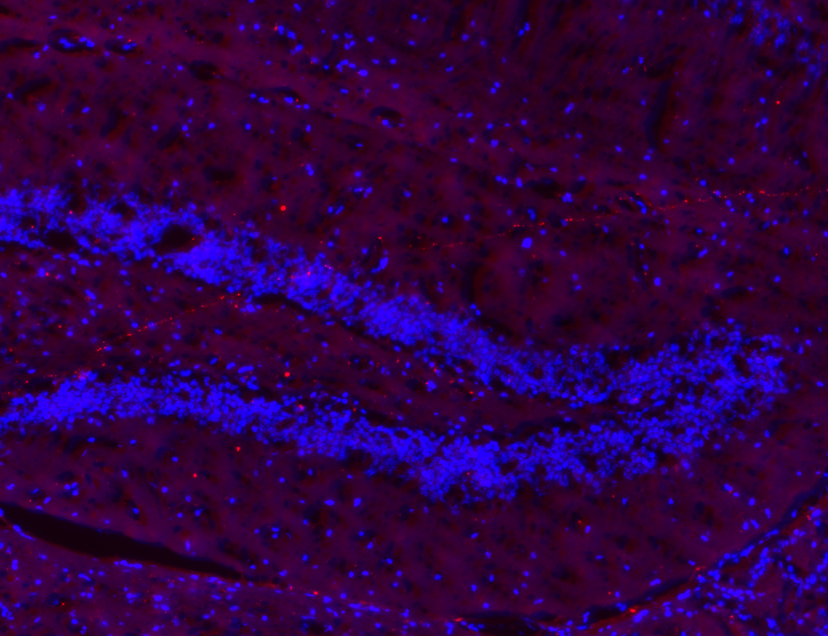

Supplement: Supplementary file 10 — Source data Fig. 6 [file 44318_2026_818_MOESM10_ESM.zip › Figure 6/Figure 6C/WT+AAV/Hippocampus-Aβ.tif]

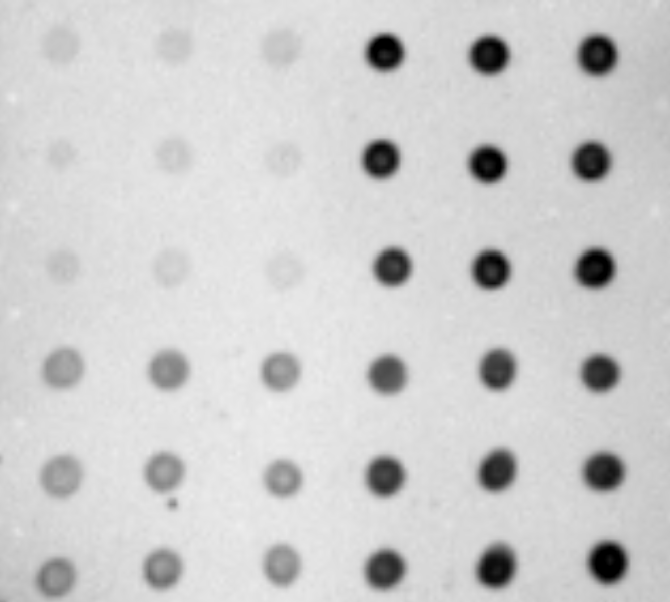

Supplement: Supplementary file 10 — Source data Fig. 6 [file 44318_2026_818_MOESM10_ESM.zip › Figure 6/Figure 6F/Cortex-Aβ.tif]

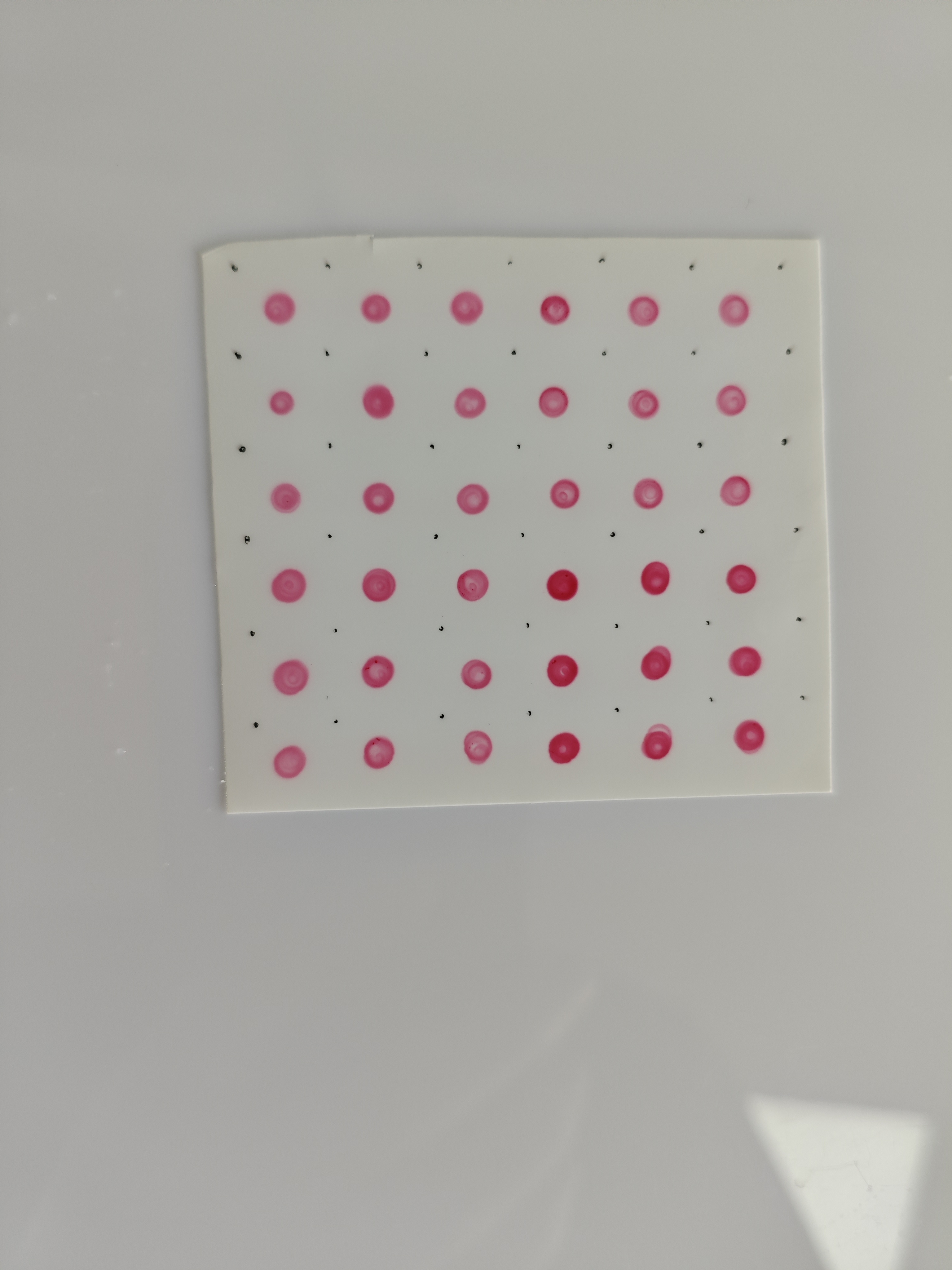

Supplement: Supplementary file 10 — Source data Fig. 6 [file 44318_2026_818_MOESM10_ESM.zip › Figure 6/Figure 6F/Cortex-ponceau S.jpg]

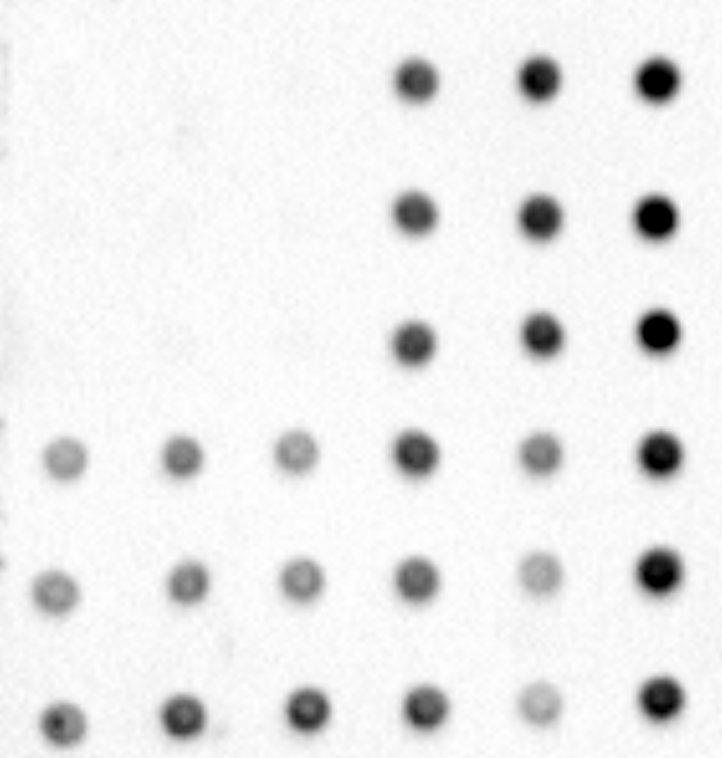

Supplement: Supplementary file 10 — Source data Fig. 6 [file 44318_2026_818_MOESM10_ESM.zip › Figure 6/Figure 6F/Hippocampus-Aβ.tif]

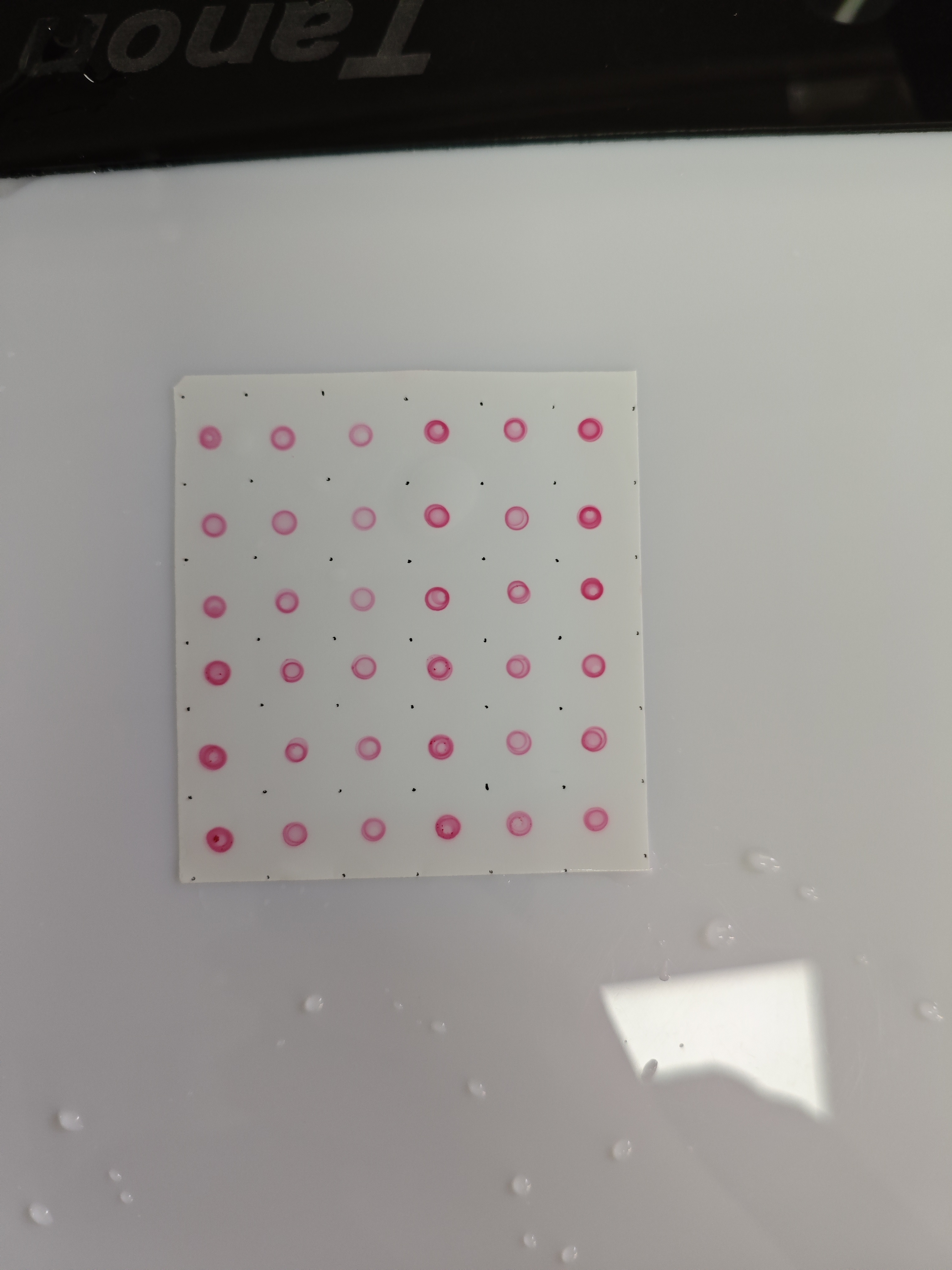

Supplement: Supplementary file 10 — Source data Fig. 6 [file 44318_2026_818_MOESM10_ESM.zip › Figure 6/Figure 6F/Hippocampus-ponceau S.jpg]

Figure 6F

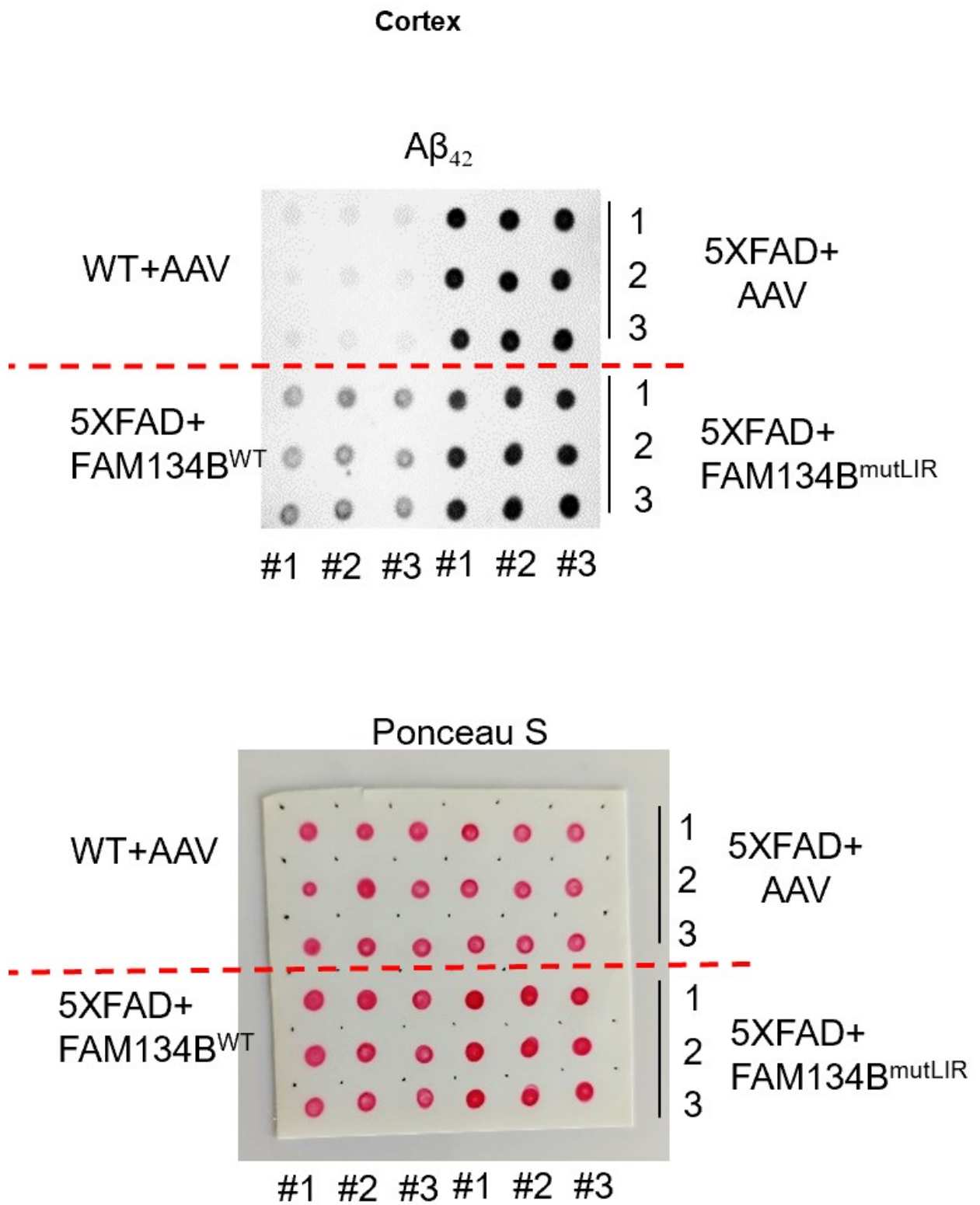

### Hippocampus

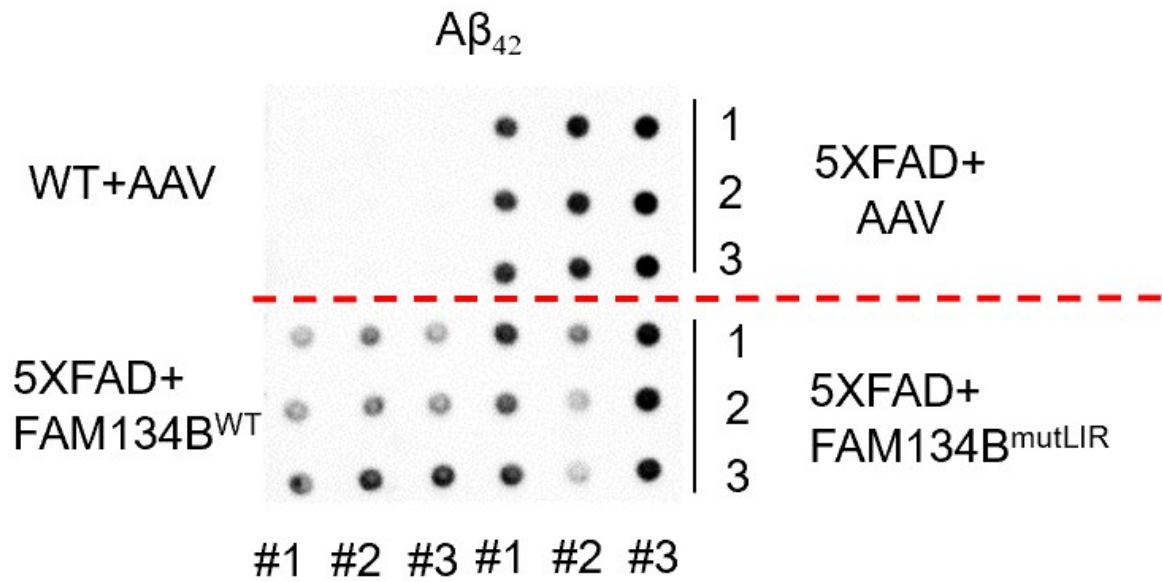

### Ponceau S

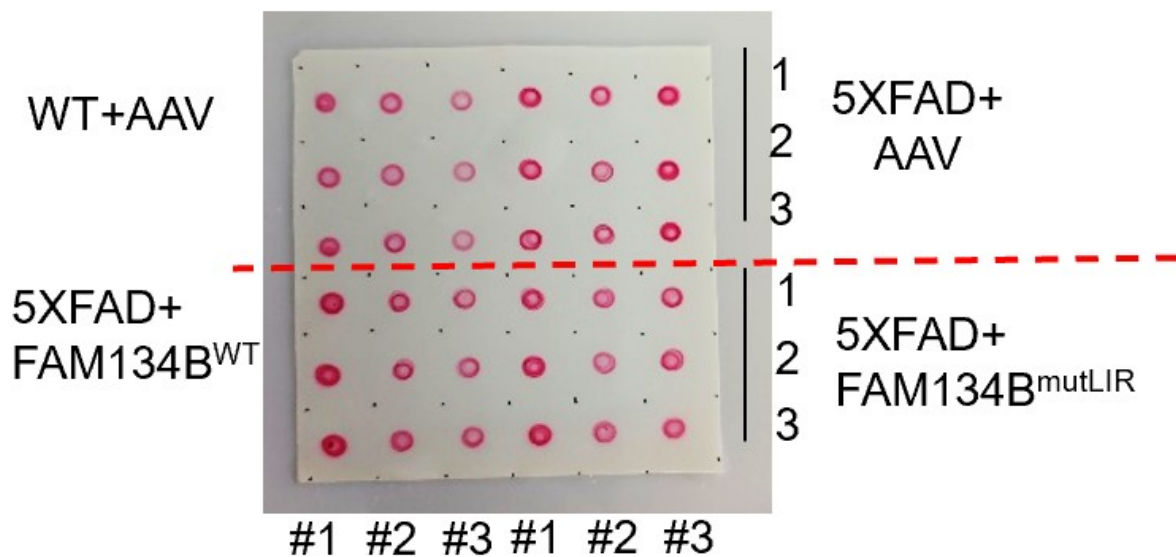

Supplement: Supplementary file 10 — Source data Fig. 6 [file 44318_2026_818_MOESM10_ESM.zip › Figure 6/Figure 6F/WB for Figure 6F.pdf]

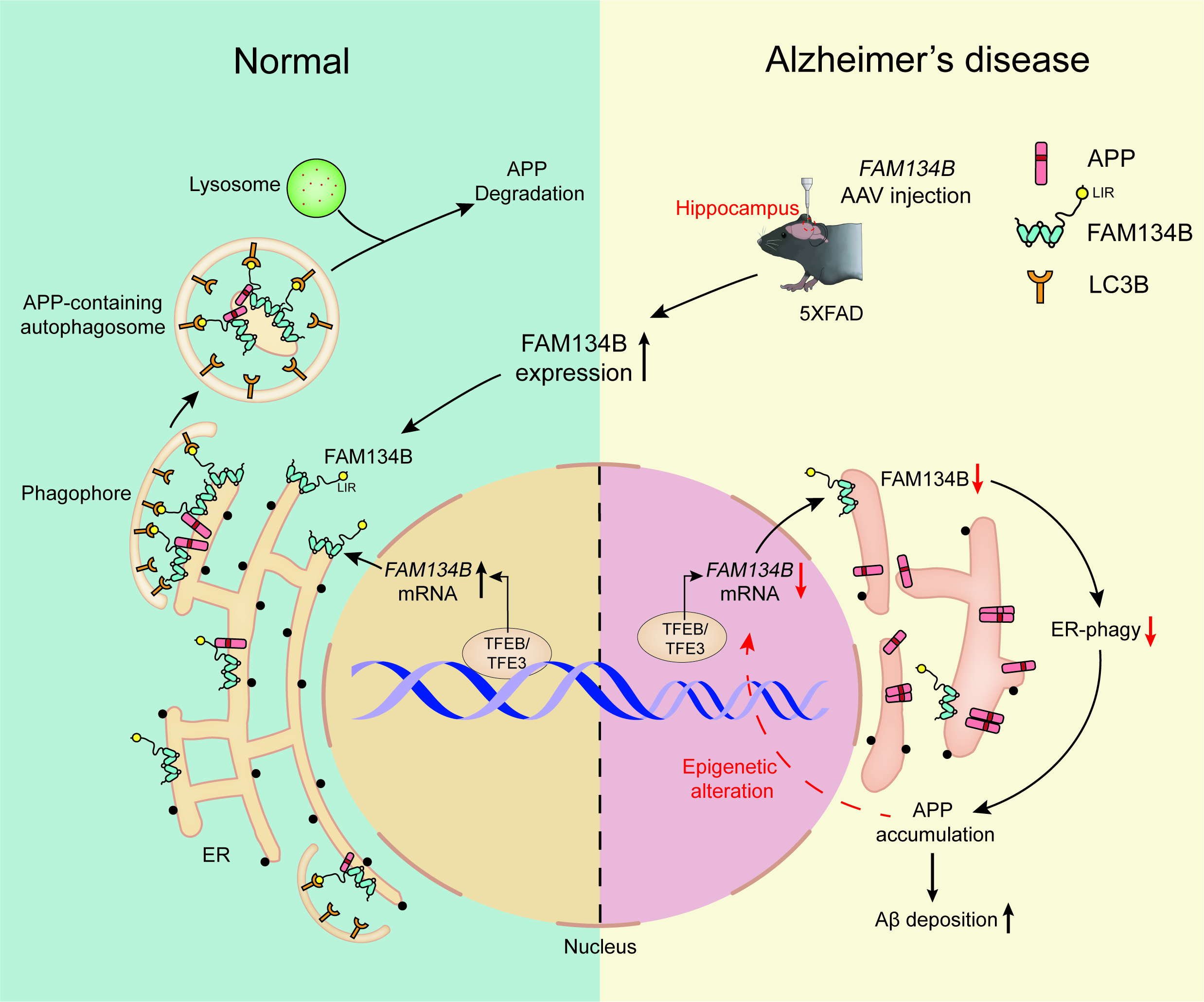

Supplement: Supplementary file 10 — Source data Fig. 6 [file 44318_2026_818_MOESM10_ESM.zip › Figure 6/Figure 6H/Figure 6H.tif]
