# Supplementary material for: FAM134B-mediated ER-phagy degrades APP and suppresses Alzheimer’s disease pathology
Source: EMBO J. 2026 May 26;45(13):4492–530. doi: 10.1038/s44318-026-00818-9 (PMC13324857; doi:10.1038/s44318-026-00818-9)
Supplement: Supplementary file 11 — Expanded View Figures [file 44318_2026_818_MOESM11_ESM.pdf]

Expanded View Figures

Figure EV1. ER-phagy receptors are downregulated and UPR is upregulated in AD, related to Fig. 1.

(**A**) Cross-database normalized microarray and RNA-seq analysis workflow. (**B**, **C**) Cross-database normalized microarray and RNA-seq analysis of ER-phagy receptor mRNA levels in the cortex of AD patients ($N = 89$) and non-AD controls ($N = 112$). P values were adjusted according to the Benjamini–Hochberg false discovery rate (FDR) correction. (**D**) qRT-PCR analysis of ER-phagy receptor mRNA levels in the cortex of 6-month-old 5XFAD mice and WT littermates ($N = 3$ per group). (**E**) qRT-PCR analysis of *Fam134b* mRNA levels in the cortex of 1-, 3-, and 6-month-old 5XFAD female mice and WT littermates ($N = 3$ per group). (**F**, **G**) qRT-PCR analysis of mRNA levels of UPR-related genes in the hippocampus and cortex of 1-, 3-, and 6-month-old 5XFAD female mice and WT littermates ($N = 4$ per group). (**H**, **I**) Immunoblotting of UPR-related protein levels in the hippocampus and cortex of 1-, 3-, and 6-month-old 5XFAD female mice and WT littermates ($N = 3$ per group). p-Ire1 and p-Perk represent phosphorylated Ire1 and Perk, indicative of activated Ire1 and Perk; Atf6-N represents the cleaved, transcriptionally active N-terminal fragment of Atf6. LE long exposure, SE short exposure. (**J**, **K**) Quantification of protein levels in (**H**, **I**) ($N = 3$ per group). Error bars represent SEM; ns, no significance, $P > 0.05$, $^{*}P < 0.05$, $^{**}P < 0.01$, $^{***}P < 0.001$, $^{****}P < 0.0001$; (**C–E**) were analyzed by unpaired Student's t test; (**F**, **G**, **J**, **K**) were analyzed by two-way ANOVA.

 © The Author(s)

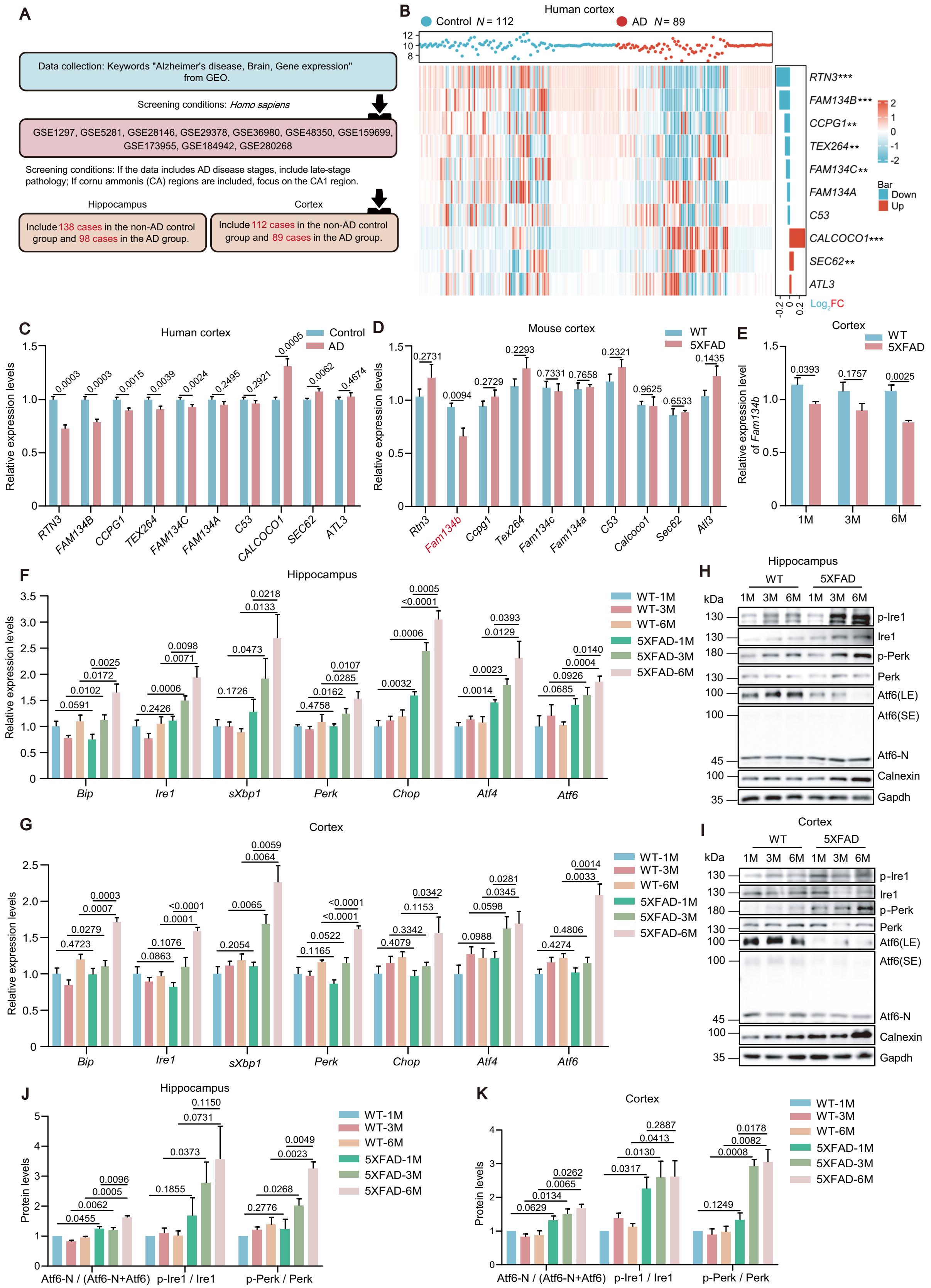
A
Data collection: Keywords "Alzheimer's disease, Brain, Gene expression" from GEO.
Screening conditions: Homo sapiens
GSE1297, GSE5281, GSE28146, GSE29378, GSE36980, GSE48350, GSE159699, GSE173955, GSE184942, GSE280268
Screening conditions: If the data includes AD disease stages, include late-stage pathology; If cornu ammonis (CA) regions are included, focus on the CA1 region.
Hippocampus
Cortex
Include 138 cases in the non-AD control group and 98 cases in the AD group.
Include 112 cases in the non-AD control group and 89 cases in the AD group.
B
Human cortex
Control N = 112
AD N = 89
RTN3***
FAM134B***
CCPG1**
TEX264**
FAM134C**
FAM134A
C53
CALCOCO1***
SEC62**
ATL3
Bar
Down
Up
Log2FC
C
Human cortex
Control
AD
Relative expression levels
RTN3
FAM134B
CCPG1
TEX264
FAM134C
FAM134A
C53
CALCOCO1
SEC62
ATL3
D
Mouse cortex
WT
5XFAD
Relative expression levels
Rtn3
Fam134b
Ccpg1
Tex264
Fam134c
Fam134a
C53
Calcoco1
Sec62
Atl3
E
Cortex
WT
5XFAD
Relative expression level of Fam134b
1M
3M
6M
F
Hippocampus
Relative expression levels
WT-1M
WT-3M
WT-6M
5XFAD-1M
5XFAD-3M
5XFAD-6M
Bip
Ire1
sXbp1
Perk
Chop
Atf4
Atf6
G
Cortex
Relative expression levels
Bip
Ire1
sXbp1
Perk
Chop
Atf4
Atf6
H
Hippocampus
WT
5XFAD
kDa
1M 3M 6M 1M 3M 6M
p-Ire1
Ire1
p-Perk
Perk
Atf6(LE)
Atf6(SE)
Atf6-N
Calnexin
Gapdh
I
Cortex
WT
5XFAD
kDa
1M 3M 6M 1M 3M 6M
p-Ire1
Ire1
p-Perk
Perk
Atf6(LE)
Atf6(SE)
Atf6-N
Calnexin
Gapdh
J
Hippocampus
Protein levels
Atf6-N / (Atf6-N+Atf6)
p-Ire1 / Ire1
p-Perk / Perk
K
Cortex
Protein levels
Atf6-N / (Atf6-N+Atf6)
p-Ire1 / Ire1
p-Perk / Perk

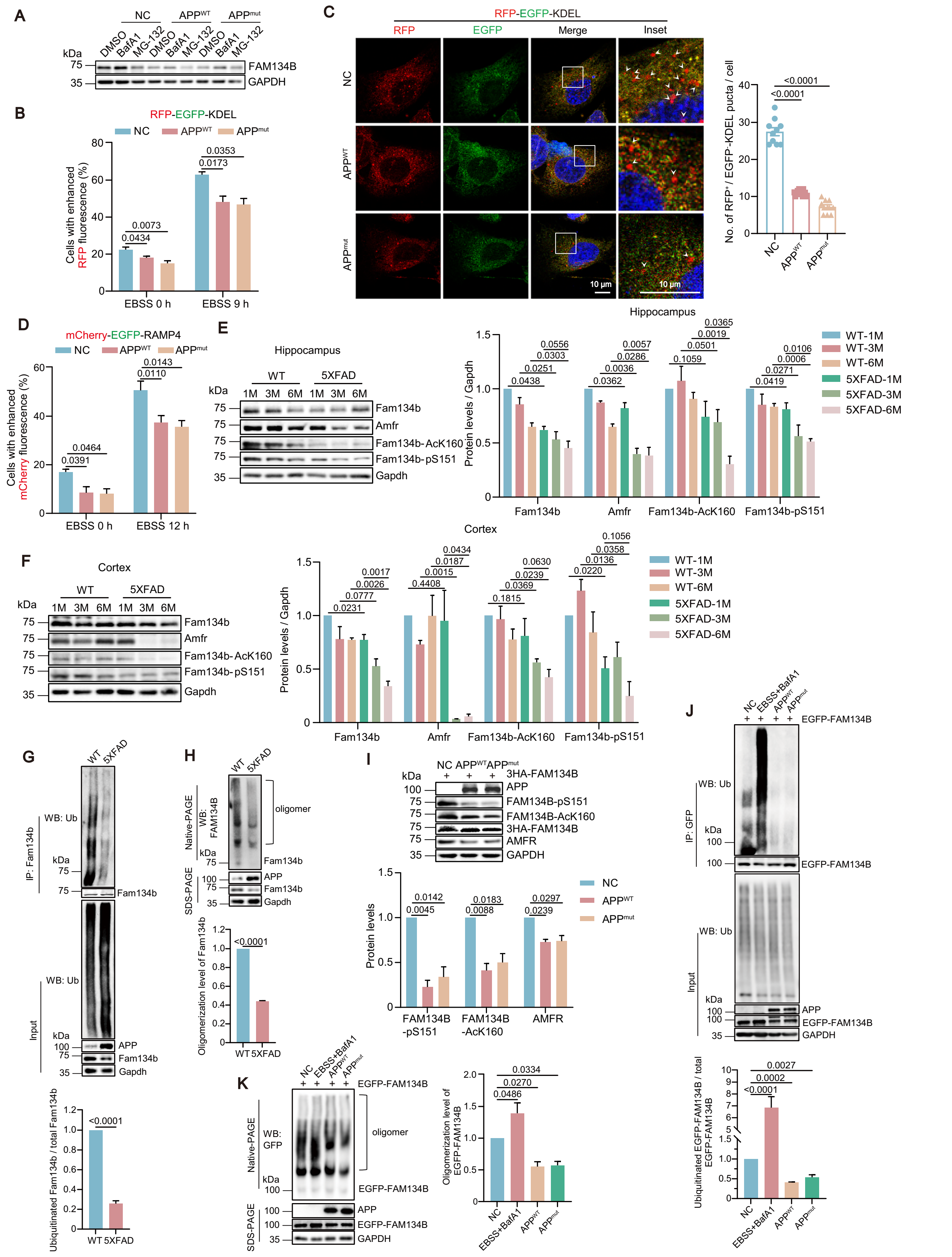

 © The Author(s)

Figure EV2. Impaired ubiquitination and oligomerization of the ER-phagy receptor FAM134B in AD, related to Fig. 1.

(**A**) Immunoblotting of protein levels in control and HEK293T cells expressing DOX-inducible $APP^{WT/mut}$. Cells were either untreated or treated with BafA1 or MG-132 for 6 h. (**B**) Quantification of cells with an increased RFP/EGFP (-KDEL) ratio by flow cytometry to assess ER-phagy flux. U2OS cells stably expressing RFP-EGFP-KDEL reporter and DOX-inducible $APP^{WT/mut}$ were treated with DOX for 24 h to induce APP expression, followed by EBSS treatment or left untreated. $n = 3$. (**C**) Confocal images of U2OS cells stably expressing RFP-EGFP-KDEL reporter and DOX-inducible $APP^{WT/mut}$. Cells were induced with DOX for 24 h, followed by EBSS treatment for 6 h. Arrowheads indicate $RFP^{+}/EGFP^{-}$ puncta. Right: Quantification of $RFP^{+}/EGFP^{-}$ puncta. $N = 10$ cells. (**D**) Quantification of cells with an increased mCherry/EGFP (-RAMP4) ratio by flow cytometry to assess ER-phagy flux. U2OS cells stably expressing mCherry-EGFP-RAMP4 reporter and DOX-inducible $APP^{WT/mut}$ were treated with DOX for 24 h to induce APP expression, followed by EBSS treatment or left untreated. $n = 3$. (**E**, **F**) Immunoblotting of protein levels in the hippocampus (**E**) and cortex (**F**) of 1-, 3-, and 6-month-old 5XFAD female mice and WT littermates ($N = 3$ per group). Fam134b-pS151 and Fam134b-AcK160 indicate Fam134b phosphorylated at Ser151 and acetylated at Lys160, respectively. (**G**) Fam134b ubiquitination levels in the hippocampus of 6-month-old WT and 5XFAD female mice ($N = 3$ per group). Fam134b was immunoprecipitated with anti-Fam134b antibodies and analyzed by immunoblotting with anti-ubiquitin antibodies. Bottom: Quantification of ubiquitinated Fam134b normalized to total Fam134b. (**H**) Fam134b oligomerization levels in the hippocampus of 6-month-old WT and 5XFAD female mice ($N = 3$ per group). Top: Native PAGE immunoblotting of Fam134b using anti-Fam134b antibodies. Input protein levels were assessed by SDS-PAGE and immunoblotting. Bottom: Quantification of oligomerized Fam134b normalized to total Fam134b. (**I**) Immunoblotting of protein levels in control and HEK293T cells expressing DOX-inducible $APP^{WT/mut}$. Cells were cultured in nutrient-rich medium and transiently transfected with 3HA–FAM134B. Bottom: Quantification of protein levels shown above. FAM134B-pS151 and FAM134B-AcK160 were normalized to total 3HA–FAM134B; AMFR was normalized to GAPDH. $n = 3$. (**J**) Effect of $APP^{WT/mut}$ overexpression on FAM134B ubiquitination. Top: EGFP-FAM134B was immunoprecipitated with anti-GFP antibodies and analyzed by immunoblotting with anti-ubiquitin antibodies. Lysates were prepared from untreated U2OS cells (NC), EBSS + BafA1-treated U2OS cells (6 h; positive control), and DOX-induced APPWT/mut-expressing U2OS cells cultured in nutrient-rich medium. All groups were transiently transfected with EGFP-FAM134B. Bottom: Quantification of ubiquitinated EGFP-FAM134B normalized to total EGFP-FAM134B. $n = 3$. (**K**) Effect of $APP^{WT/mut}$ overexpression on FAM134B oligomerization. Left: Native PAGE immunoblotting of EGFP-FAM134B using anti-GFP antibodies. Lysates were prepared from NC, EBSS + BafA1, and DOX-induced $APP^{WT/mut}$ U2OS cells. Input protein levels were assessed by SDS-PAGE and immunoblotting. Right: Quantification of oligomerized EGFP-FAM134B normalized to total EGFP-FAM134B. $n = 3$. Error bars represent SEM; ns, no significance, $P > 0.05$, $^{*}P < 0.05$, $^{**}P < 0.01$, $^{***}P < 0.001$, $^{****}P < 0.0001$; (**B**–**D**, **I**–**K**) were analyzed by one-way ANOVA; (**E**, **F**) were analyzed by two-way ANOVA; (**G**, **H**) were analyzed by unpaired Student's t test.

© The Author(s)

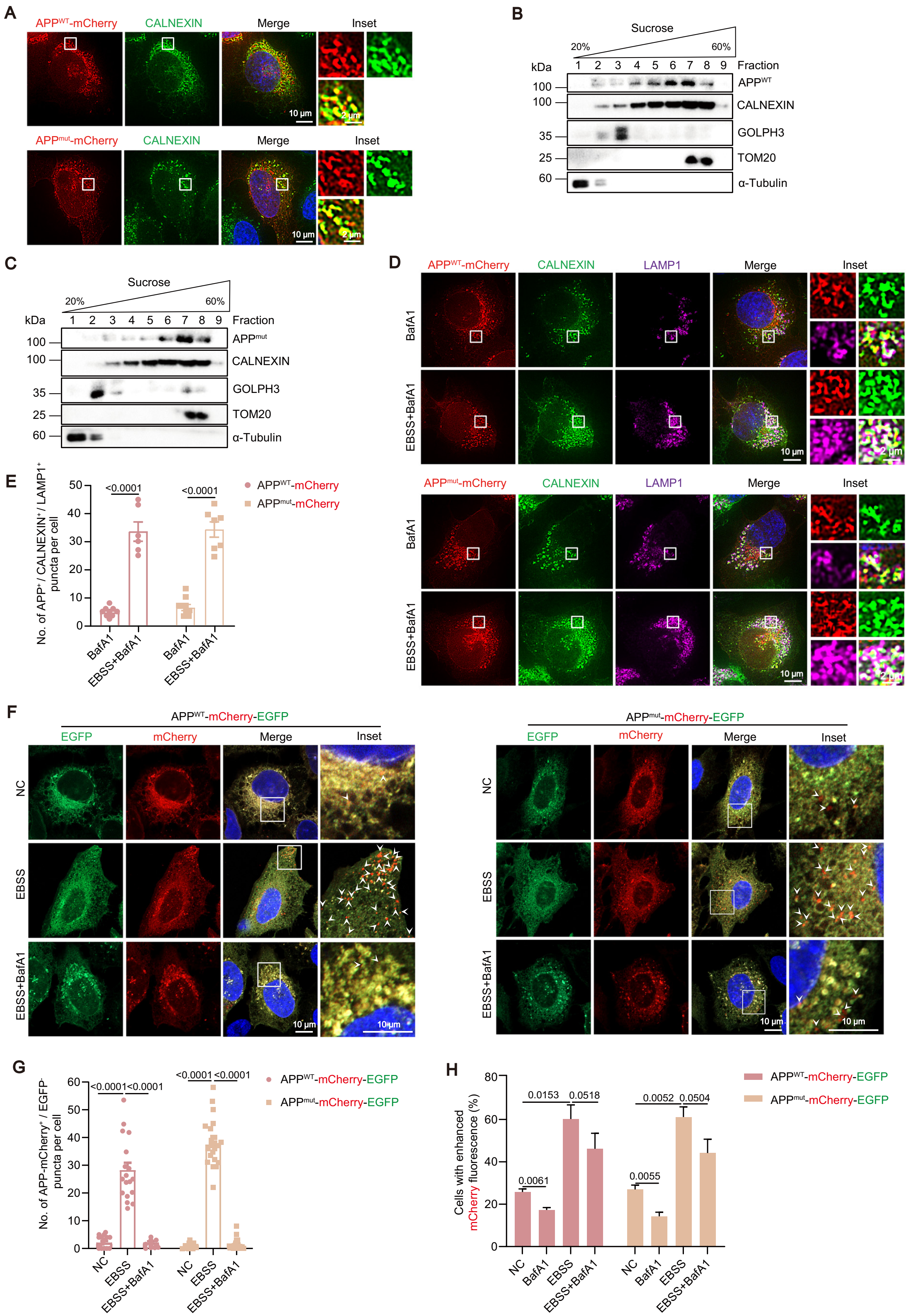
A
APPWT-mCherry
CALNEXIN
Merge
Inset
10 μm
2 μm
APPmut-mCherry
B
Sucrose
20%
60%
kDa
Fraction
APPWT
GOLPH3
TOM20
α-Tubulin
C
APPmut
D
LAMP1
BafA1
EBSS+BafA1
E
No. of APP+ / CALNEXIN+ / LAMP1+ puncta per cell
<0.0001
F
APPWT-mCherry-EGFP
APPmut-mCherry-EGFP
EGFP
mCherry
NC
EBSS
G
No. of APP-mCherry+ / EGFP- puncta per cell
H
Cells with enhanced mCherry fluorescence (%)
0.0153
0.0518
0.0061
0.0052
0.0504
0.0055

 © The Author(s)

Figure EV3. ER-localized APP is degraded through the autophagosome-lysosome pathway, related to Fig. 2.

(**A**) Multi-SIM images showing colocalization of $APP^{WT/mut}$-mCherry with CALNEXIN. U2OS cells expressing DOX-inducible $APP^{WT/mut}$-mCherry were cultured in nutrient-rich medium. CALNEXIN was detected by immunostaining. (**B**, **C**) Subcellular fractionation of U2OS cells expressing DOX-inducible APP^{WT} (**B**) or APP^{mut} (**C**). Cell lysates were fractionated on a sucrose gradient. CALNEXIN, ER marker; GOLPH3, Golgi marker; TOM20, mitochondrial marker; α-Tubulin, cytosolic marker. (**D**) Multi-SIM images showing colocalization of APP with CALNEXIN and LAMP1. U2OS cells expressing DOX-inducible $APP^{WT/mut}$-mCherry were treated with BafA1 or EBSS + BafA1 for 6 h. CALNEXIN and LAMP1 were detected by immunostaining. (**E**) Quantification of APP^{+}/$CALNEXIN^{+}$/$LAMP1^{+}$ puncta in (**D**). $N = 10$ cells (each BafA1 group), $N = 6$ cells (APP^{WT}-mCherry, EBSS + BafA1), $N = 7$ cells (APP^{mut}-mCherry, EBSS + BafA1). (**F**) Live-cell confocal images of U2OS cells expressing DOX-inducible $APP^{WT/mut}$-mCherry-EGFP, either untreated or treated with EBSS, or EBSS + BafA1 for 6 h. Arrowheads indicate $mCherry^{+}$/$EGFP^{-}$ puncta. (**G**) Quantification of $mCherry^{+}$/$EGFP^{-}$ puncta in (**F**). $N = 17$ cells (APP^{WT}-mCherry-EGFP, NC); $N = 25$ cells (APP^{mut}-mCherry-EGFP, NC); $N = 18$ cells (APP^{WT}-mCherry-EGFP, EBSS); $N = 25$ cells (APP^{mut}-mCherry-EGFP, EBSS); $N = 20$ cells (APP^{WT}-mCherry-EGFP, EBSS + BafA1); $N = 30$ cells (APP^{mut}-mCherry-EGFP, EBSS + BafA1). (**H**) Quantification of cells with an increased mCherry/EGFP (− APP) ratio by flow cytometry to assess lysosomal delivery of APP. U2OS cells expressing DOX-inducible $APP^{WT/mut}$-mCherry-EGFP were either untreated or treated with BafA1, EBSS, or EBSS + BafA1 for 6 h. $n = 3$. Error bars represent SEM; *$P < 0.05$, ****$P < 0.0001$; (**E**) was analyzed by unpaired Student's *t* test; (**G**) was analyzed by one-way ANOVA; (**H**) was analyzed by two-way ANOVA.

© The Author(s)

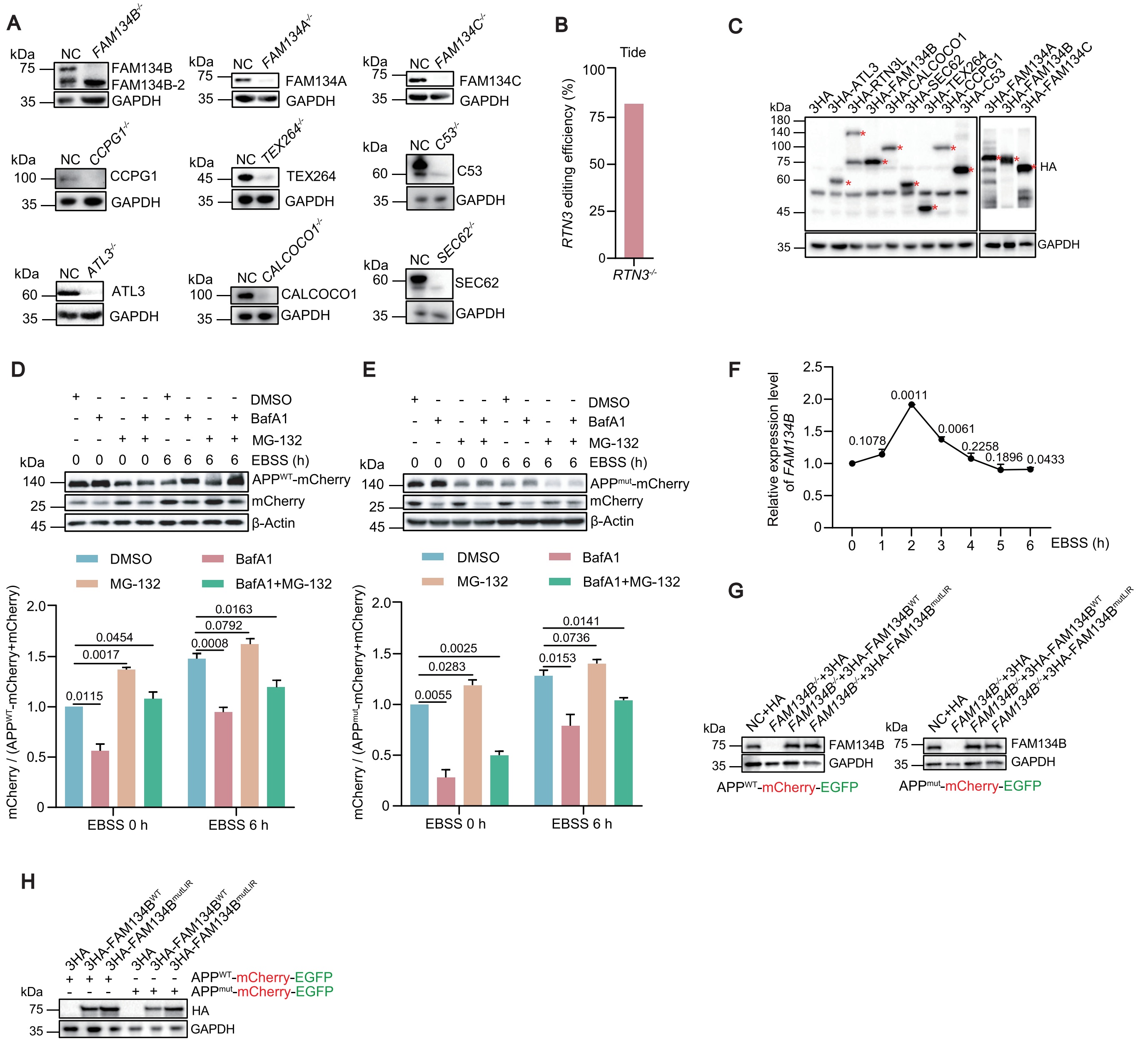

Figure EV4. APP is degraded through the lysosomal pathway, related to Fig. 2.

(**A**) Immunoblotting to validate the CRISPR-Cas9 knockout efficiency of ER-phagy receptors. (**B**) TIDE (Tracking of Indels by DEcomposition) analysis showing an 82.3% total editing efficiency for *RTN3* knockout. TIDE was used in place of immunoblotting because an RTN3L-specific antibody is unavailable. (**C**) Immunoblotting to validate the overexpression efficiency of HA-tagged ER-phagy receptors. The asterisks mark the correct band position of the target protein. (**D**, **E**) Immunoblotting of $APP^{WT/mut}$-mCherry cleavage into free mCherry. HEK293T cells expressing DOX-inducible APP^{WT}-mCherry (**D**) or APP^{mut}-mCherry (**E**) were treated as indicated. $n = 4$. (**F**) qRT-PCR analysis of *FAM134B* mRNA levels in U2OS cells, either untreated or treated with EBSS for the indicated times. $n = 3$. (**G**, **H**) Immunoblotting to validate the expression levels of 3HA-tagged $FAM134B^{WT}$ and $FAM134B^{mutLIR}$. Error bars represent SEM; ns, no significance, $P > 0.05$, $^*P < 0.05$, $^{**}P < 0.01$, $^{***}P < 0.001$; (**D**, **E**) were analyzed by two-way ANOVA; (**F**) was analyzed by unpaired Student's *t* test.

 © The Author(s)

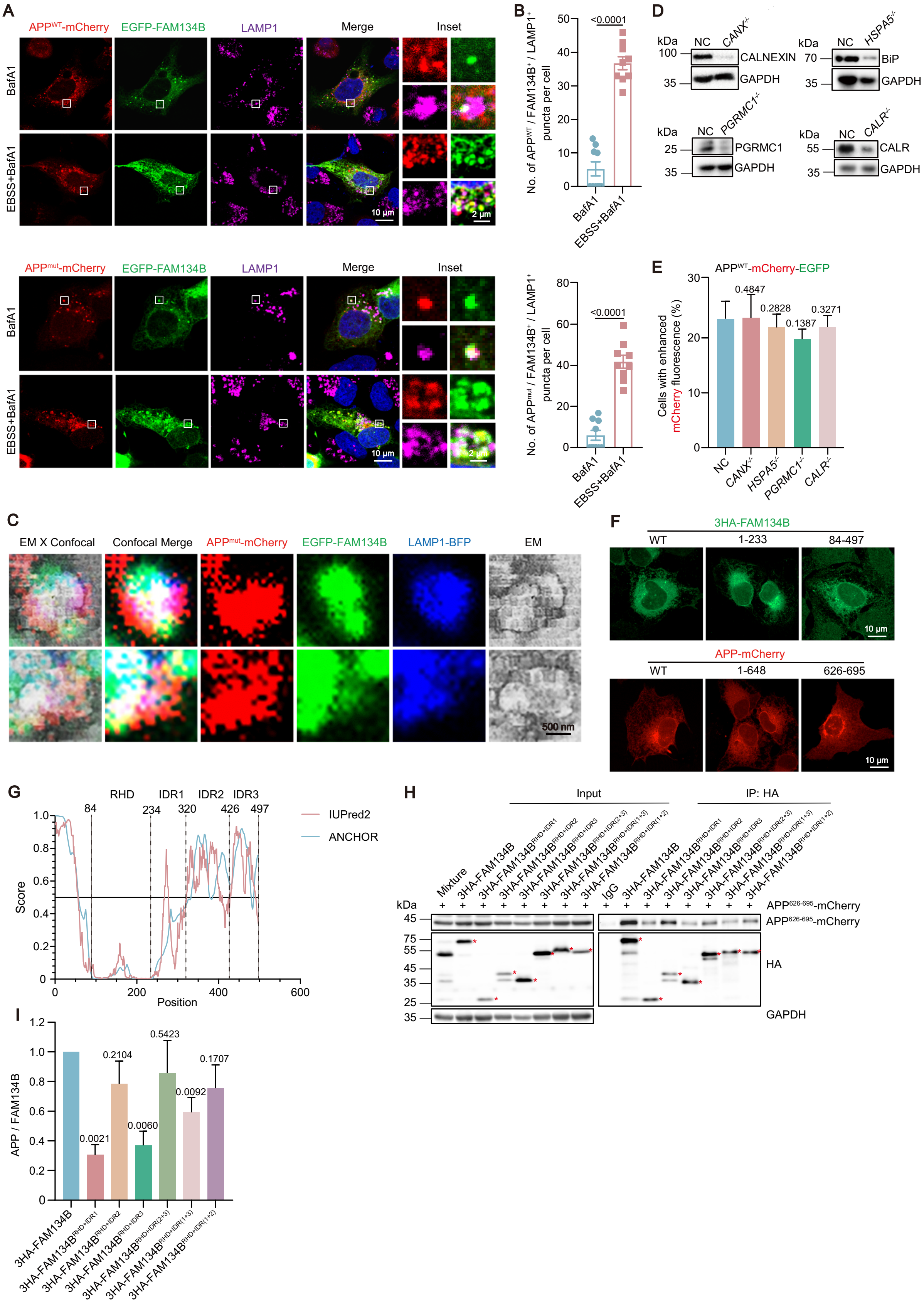
A
APPWT-mCherry
EGFP-FAM134B
LAMP1
Merge
Inset
BafA1
EBSS+BafA1
10 µm
2 µm
APPmut-mCherry
B
No. of APPWT / FAM134B+ / LAMP1+ puncta per cell
<0.0001
No. of APPmut / FAM134B+ / LAMP1+ puncta per cell
D
kDa
NC
CANX-/-
CALNEXIN
GAPDH
HSPA5-/-
BiP
PGRMC1-/-
PGRMC1
CALR-/-
CALR
E
APPWT-mCherry-EGFP
Cells with enhanced mCherry fluorescence (%)
0.4847
0.2828
0.1387
0.3271
C
EM X Confocal
Confocal Merge
EGFP-FAM134B
LAMP1-BFP
EM
500 nm
F
3HA-FAM134B
WT
1-233
84-497
APP-mCherry
1-648
626-695
G
RHD
IDR1
IDR2
IDR3
Score
Position
IUPred2
ANCHOR
H
Input
IP: HA
Mixture
3HA-FAM134B
IgG
APP626-695-mCherry
HA
GAPDH
I
APP / FAM134B
0.0021
0.2104
0.0060
0.5423
0.0092
0.1707
3HA-FAM134BRHD+IDR1
3HA-FAM134BRHD+IDR2
3HA-FAM134BRHD+IDR3
3HA-FAM134BRHD+IDR(2+3)
3HA-FAM134BRHD+IDR(1+3)
3HA-FAM134BRHD+IDR(1+2)

Figure EV5. ER-localized APP is degraded by FAM134B-mediated ER-phagy, related to Fig. 3.

(**A**) Confocal images showing colocalization of $APP^{WT/mut}$-mCherry with EGFP-FAM134B and LAMP1. U2OS cells expressing DOX-inducible $APP^{WT/mut}$-mCherry were transiently transfected with EGFP-FAM134B and treated with BafA1 or EBSS + BafA1 for 6 h. LAMP1 was detected by immunostaining. (**B**) Quantification of $APP^{WT/mut}$-$mCherry^{+}$/EGFP-$FAM134B^{+}$/$LAMP1^{+}$ puncta in (**A**). $N = 10$ cells. (**C**) CLEM images showing colocalization of EGFP-FAM134B, APP^{mut}-mCherry, and LAMP1-BFP. U2OS cells expressing DOX-inducible APP^{mut}-mCherry were transiently transfected with EGFP-FAM134B and LAMP1-BFP, then treated with EBSS + BafA1 for 6 h. Confocal and EM overlay images reveal triple-positive puncta colocalizing with autolysosomal structures. (**D**) Immunoblotting to validate the CRISPR-Cas9 knockout efficiency of ER chaperones. (**E**) Quantification of cells with an increased mCherry/EGFP (-APP) ratio by flow cytometry to assess lysosomal delivery of APP^{WT}. WT, *CANX* (CALNEXIN)-, *HSPA5* (BiP)-, *PGRMC1*-, and *CALR* (Calreticulin)-knockout U2OS cells expressing DOX-inducible APP^{WT}-mCherry-EGFP were cultured in nutrient-rich medium. $n = 3$. (**F**) Confocal images showing the subcellular localization of full-length and truncated 3HA-FAM134B and APP-mCherry. FAM134B was detected using anti-HA antibodies. (**G**) Predicted IDR structure of the C-terminus of FAM134B using IUPred2A. Three major IDRs were identified: IDR1 (aa 234–320), IDR2 (aa 321–426), and IDR3 (aa 427–497). IUPred2 (red) predicts IDRs (score > 0.5), while ANCHOR (blue) identifies binding-competent subregions within IDRs that fold upon protein interaction. (**H**) Co-IP of $APP^{626\text{–}695}$–mCherry with IDR-truncated FAM134B to determine which IDR mediates binding to the APP C-terminus. Lysates were prepared from HEK293T cells co-transfected with $APP^{626\text{–}695}$–mCherry and the indicated FAM134B constructs. The asterisks mark the correct band position of the target protein. (**I**) Quantification of APP co-immunoprecipitated with FAM134B in (**H**). $n = 3$. Error bars represent SEM; ns, no significance, $P > 0.05$, $^{*}P < 0.05$, $^{**}P < 0.01$, $^{****}P < 0.0001$; (**B**, **E**, **I**) were analyzed by unpaired Student's t test.

 © The Author(s)

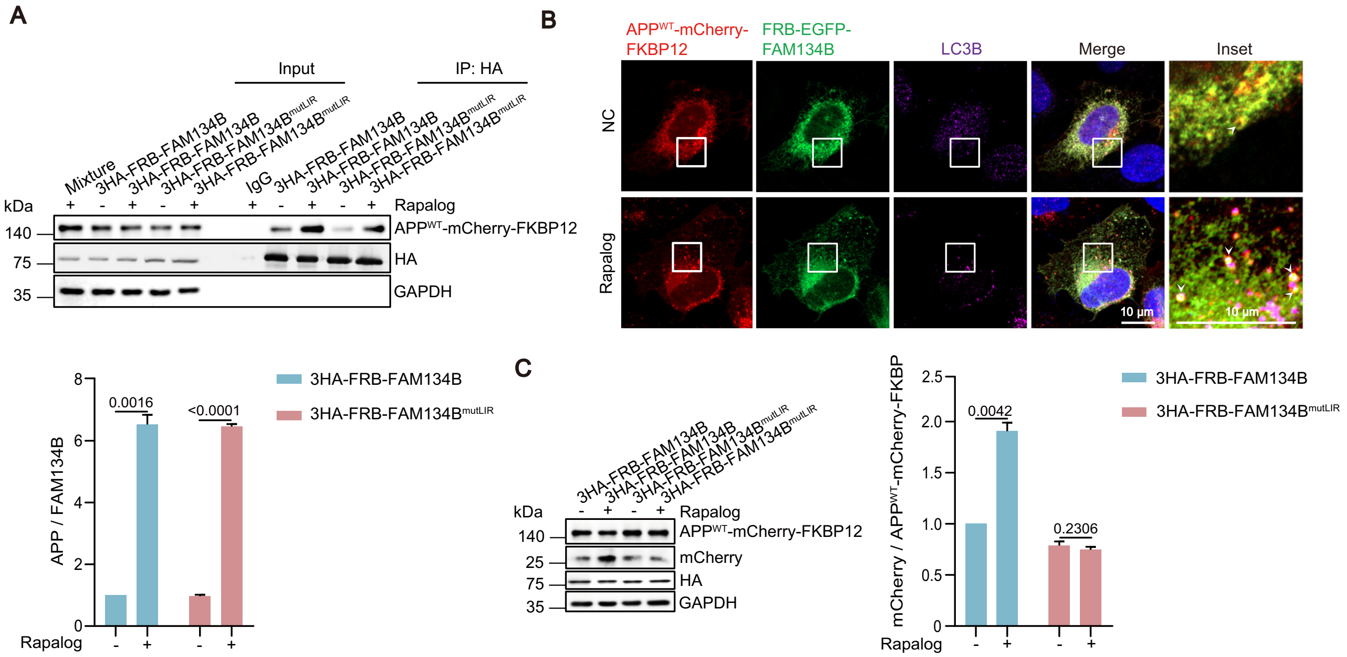

Figure EV6. Induced APP-FAM134B dimerization enhances APP degradation via ER-phagy, related to Fig. 3.

(**A**) Co-IP of APP^{WT} with FAM134B or FAM134B^{mutLIR}. Lysates were prepared from U2OS cells expressing DOX-inducible APP^{WT}-mCherry-FKBP12 and transiently transfected with 3HA-FRB-FAM134B or 3HA-FRB-FAM134B^{mutLIR}, either untreated or treated with Rapalog. Bottom: Quantification of APP co-immunoprecipitated with FAM134B. $n = 3$. (**B**) Confocal images showing colocalization of APP^{WT} with FAM134B and LC3B. U2OS cells expressing DOX-inducible APP^{WT}-mCherry-FKBP12 and transiently transfected with FRB-EGFP-FAM134B, either untreated or treated with Rapalog. LC3B was detected by immunostaining. (**C**) Immunoblotting of APP^{WT}-mCherry-FKBP12 cleavage into free mCherry. U2OS cells expressing DOX-inducible APP^{WT}-mCherry-FKBP12 transiently transfected with 3HA-FRB-FAM134B or 3HA-FRB-FAM134B^{mutLIR}, either untreated or treated with Rapalog. $n = 3$. Error bars represent SEM; ns, no significance, $P > 0.05$, $^{**}P < 0.01$, $^{***}P < 0.001$, $^{****}P < 0.0001$; (**A**, **C**) were analyzed by one-way ANOVA.

© The Author(s)

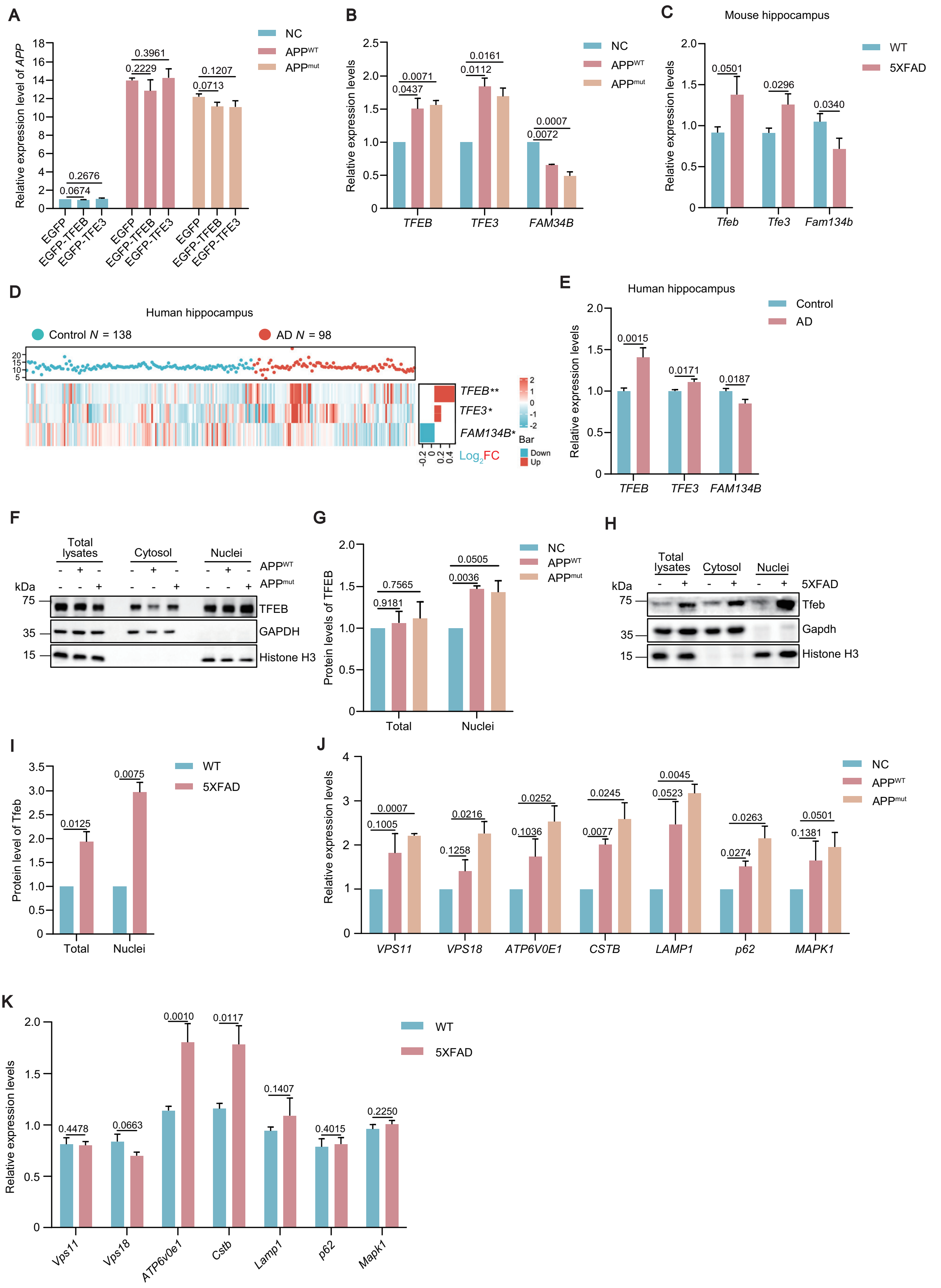
A
Relative expression level of APP
NC
APP^WT
APP^mut
0.2676
0.0674
0.3961
0.2229
0.1207
0.0713
EGFP
EGFP-TFEB
EGFP-TFE3
B
Relative expression levels
0.0071
0.0437
0.0161
0.0112
0.0007
0.0072
TFEB
TFE3
FAM34B
C
Mouse hippocampus
WT
5XFAD
0.0501
0.0296
0.0340
Tfeb
Tfe3
Fam134b
D
Human hippocampus
Control N = 138
AD N = 98
TFEB**
TFE3*
FAM134B*
Log2FC
Bar
Down
Up
E
Human hippocampus
Control
AD
0.0015
0.0171
0.0187
TFEB
TFE3
FAM134B
F
Total lysates
Cytosol
Nuclei
APP^WT
APP^mut
kDa
TFEB
GAPDH
Histone H3
G
Protein levels of TFEB
0.7565
0.9181
0.0505
0.0036
Total
Nuclei
H
Total lysates
Cytosol
Nuclei
5XFAD
Tfeb
Gapdh
Histone H3
I
Protein level of Tfeb
0.0125
0.0075
Total
Nuclei
J
Relative expression levels
0.0007
0.1005
0.0216
0.1258
0.0252
0.1036
0.0245
0.0077
0.0045
0.0523
0.0263
0.0274
0.0501
0.1381
VPS11
VPS18
ATP6V0E1
CSTB
LAMP1
p62
MAPK1
K
Relative expression levels
0.4478
0.0663
0.0010
0.0117
0.1407
0.4015
0.2250
Vps11
Vps18
ATP6v0e1
Cstb
Lamp1
p62
Mapk1

 © The Author(s)

Figure EV7. Nuclear translocation of TFEB/TFE3 is increased in AD, related to Fig. 4.

(**A**) qRT-PCR analysis of *APP* mRNA levels in control and U2OS cells expressing DOX-inducible $APP^{WT/mut}$ and transiently transfected with EGFP, EGFP-TFEB, or EGFP-TFE3 plasmids. $n = 3$. (**B**) qRT-PCR analysis of *TFEB*, *TFE3*, and *FAM134B* mRNA levels in control and U2OS cells expressing DOX-inducible $APP^{WT/mut}$. $n = 3$. (**C**) qRT-PCR analysis of *Tfeb*, *Tfe3*, and *Fam134b* mRNA levels in the hippocampus of 6-month-old WT and 5XFAD mice ($N = 3$ per group). (**D**, **E**) Cross-database normalized microarray and RNA-seq analysis of *TFEB*, *TFE3*, and *FAM134B* mRNA levels in the hippocampus of AD patients ($N = 98$) and non-AD controls ($N = 138$). P values were adjusted according to the Benjamini–Hochberg false discovery rate (FDR) correction. (**F**) Nuclear–cytosolic fractionation analysis of TFEB localization in control and U2OS cells expressing DOX-inducible $APP^{WT/mut}$. GAPDH: cytosolic marker; histone H3: nuclear marker. (**G**) Quantification of relative levels of total and nuclear TFEB in (**F**). $n = 3$. (**H**) Nuclear–cytosolic fractionation analysis of Tfeb localization in the hippocampus of WT and 5XFAD mice. (**I**) Quantification of relative levels of total and nuclear Tfeb in (**H**). $n = 3$. (**J**) qRT-PCR analysis of mRNA levels of TFEB/TFE3 downstream target genes in control and U2OS cells expressing DOX-inducible $APP^{WT/mut}$. $n = 3$. (**K**) qRT-PCR analysis of mRNA levels of Tfeb/Tfe3 downstream target genes in the hippocampus of 6-month-old WT and 5XFAD mice ($N = 3$ per group). Error bars represent SEM; ns, no significance, $P > 0.05$, $^*P < 0.05$, $^{**}P < 0.01$, $^{***}P < 0.001$; (**A**, **B**, **G**, **J**) were analyzed by one-way ANOVA; (**C**, **E**, **I**, **K**) were analyzed by unpaired Student's *t* test.

© The Author(s)

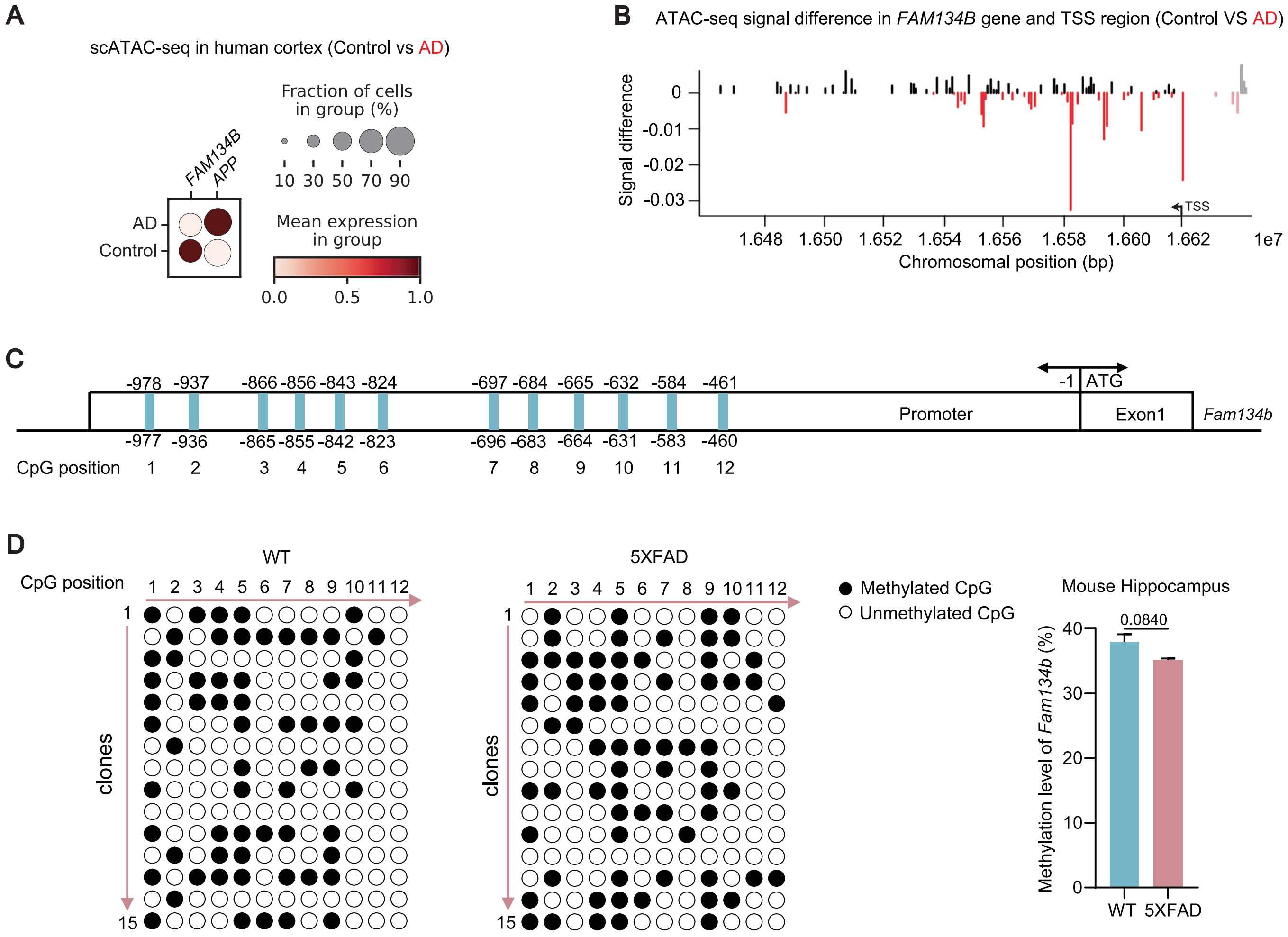

Figure EV8. Chromatin accessibility at the FAM134B promoter is reduced, while DNA methylation remains unchanged in AD, related to Fig. 4.

(**A**) Analysis of a published single-cell ATAC-seq (scATAC-seq) dataset (syn52293424) from cortex samples revealed a significant decrease in both the proportion of cells with accessible chromatin and the average accessibility at the *FAM134B* locus in AD patients ($N = 15$) compared to controls ($N = 48$). By contrast, chromatin accessibility at the *APP* locus was increased in AD samples. (**B**) scATAC-seq signal tracks showing chromatin accessibility at the *FAM134B* gene body and TSS region (±10 kb) in cortex samples from controls and AD patients. All signals are normalized to reads per million (RPM). (**C**) Schematic of the 5′ region of the mouse *Fam134b* gene, showing the promoter, first exon, and translation start site (ATG). The nucleotide upstream of the ATG is designated as position −1. Twelve analyzed CpG sites are mapped relative to the ATG (e.g., −978/−977, −937/−936) and numbered 1–12. (**D**) Bisulfite sequencing PCR (BSP) analysis of CpG methylation patterns in the *Fam134b* promoter region in the hippocampus of WT and 5XFAD mice ($N = 3$ per group). In total, 15 randomly selected clones were analyzed for each sample. Error bars represent SEM; ns, no significance; unpaired Student's t test.

 © The Author(s)

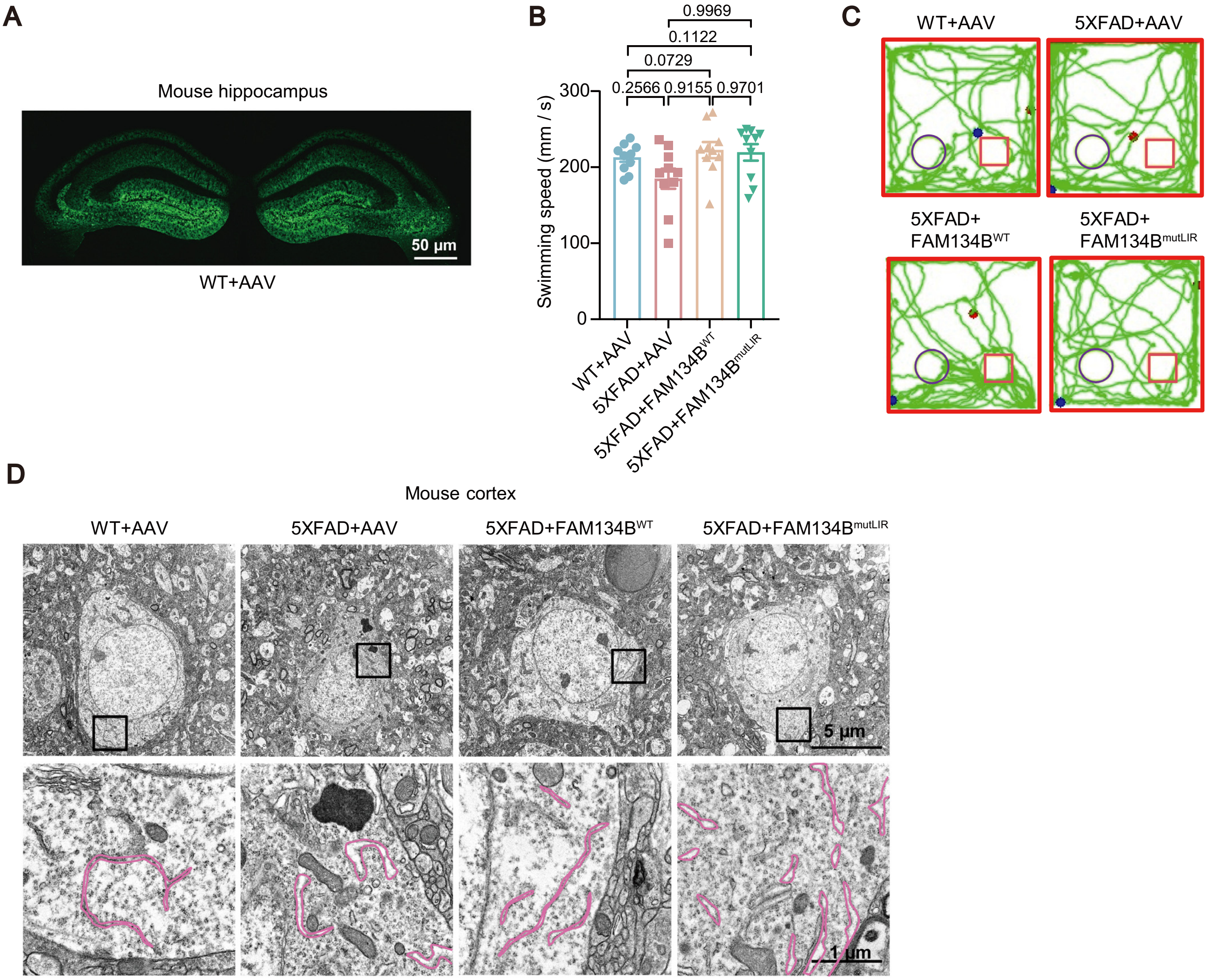

Figure EV9. Upregulation of FAM134B protects against AD-related pathology in the 5XFAD mouse model, related to Figs. 5 and 6.

(**A**) Representative images showing hippocampus-specific expression of AAV-delivered EGFP or EGFP-tagged fusion proteins following stereotactic brain injection. (**B**) Swimming speed during the 4-day Morris water maze test ($N = 10$ per group). WT + AAV; 5XFAD + AAV; 5XFAD + FAM134B^{WT}; 5XFAD + FAM134B^{mutLIR}. Error bars represent SEM; ns, no significance, $P > 0.05$; two-way ANOVA test. (**C**) Representative paths in the novel object recognition test. Blue circle indicates the novel object. (**D**) Representative TEM images of cortical neurons. ER membranes are outlined in magenta.

© The Author(s)
