# Supplementary material for: FAM134B-mediated ER-phagy degrades APP and suppresses Alzheimer’s disease pathology
Source: EMBO J. 2026 May 26;45(13):4492–530. doi: 10.1038/s44318-026-00818-9 (PMC13324857; doi:10.1038/s44318-026-00818-9)
Supplement: Supplementary file 4 — Peer Review File [file 44318_2026_818_MOESM4_ESM.pdf]

THE EMBO JOURNAL

FAM134B-mediated ER-phagy degrades APP and suppresses Alzheimer's disease pathology

Yuting Zhang, Jun Sun, Yang Cai, Ziyan Xu, Xiang Li, Wei Wei, Pai Liu, Qiming Sun, Zhihao Wang, and Yixian Cui

Corresponding author(s): Yixian Cui (yixian.cui@whu.edu.cn)

Review Timeline:

Submission Date:	7th Oct 25
Editorial Decision:	18th Dec 25
Revision Received:	27th Feb 26
Editorial Decision:	16th Apr 26
Revision Received:	27th Apr 26
Accepted:	8th May 26

Editor: William Teale

Transaction Report:

(Note: With the exception of the correction of typographical or spelling errors that could be a source of ambiguity, letters and reports are not edited. Depending on transfer agreements, referee reports obtained elsewhere may or may not be included in this compilation. Referee reports are anonymous unless the Referee chooses to sign their reports.)

1st Editorial Decision — 18th Dec 2025

Dear Dr Cui,

Thank you again for the submission of your manuscript entitled "FAM134B-mediated ER-phagy degrades APP and protects against Alzheimer's disease pathology" and for your patience during the review process. We have now received reports from three referees, which I copy below.

As you can see from their comments, all thought that your manuscript could represent an important contribution to our understanding the progression of Alzheimer's disease. That said, before proceeding you will need to bring out some mechanistic clarity on the pathways that mediate the effect of FAM134B on APP. This will require your attention before your manuscript can be published in The EMBO Journal. It may also be that, if the requested clarifications on mechanism are too dense to be practically made here, the manuscript could remain a good candidate for publication in EMBO Reports.

Based on the overall interest expressed in the reports, however, I would like to invite you to address the comments of all referees in a revised version of the manuscript. I should add that it is The EMBO Journal policy to allow only a single major round of revision and that it is therefore important to resolve the main concerns at this stage. I believe the concerns of the referees are reasonable and addressable; however, the reviews are long. I would be happy to go through your revision plan with you if you think that this might help streamline this process. Please contact me if you have any questions, need further input on the referee comments or if you anticipate any problems in addressing any of their points. Please, follow the instructions below when preparing your manuscript for resubmission.

I would also like to point out that as a matter of policy, competing manuscripts published during this period will not be taken into consideration in our assessment of the novelty presented by your study ("scooping" protection). We have extended this 'scooping protection policy' beyond the usual 3 month revision timeline to cover the period required for a full revision to address the essential experimental issues. Please contact me if you see a paper with related content published elsewhere to discuss the appropriate course of action.

When preparing your letter of response to the referees' comments, please bear in mind that this will form part of the Review Process File, and will therefore be available online to the community. For more details on our Transparent Editorial Process, please visit our website: https://link.springer.com/journal/44318/submission-guidelines#cms-Revised-submissions

Again, please contact me at any time during revision if you need any help or have further questions.

Thank you very much again for the opportunity to consider your work for publication. I look forward to your revision.

Best regards,

William Teale

William Teale, Ph.D.
Editor
The EMBO Journal

When submitting your revised manuscript, please carefully review the instructions below and include the following items:

1) a .docx formatted version of the manuscript text (including legends for main figures, EV figures and tables). Please make sure that the changes are highlighted to be clearly visible.

2) individual production quality figure files as .eps, .tif, .jpg (one file per figure).

3) a .docx formatted letter INCLUDING the reviewers' reports and your detailed point-by-point response to their comments. As part of the EMBO Press transparent editorial process, the point-by-point response is part of the Review Process File (RPF), which will be published alongside your paper.

4) a complete author checklist, which you can download from our author guidelines (https://link.springer.com/journal/44318/submission-guidelines#cms-Revised-submissions). Please insert information in the checklist that is also reflected in the manuscript. The completed author checklist will also be part of the RPF.

5) Please note that all corresponding authors are required to supply an ORCID ID for their name upon submission of a revised manuscript.

6) We require a 'Data Availability' section after the Materials and Methods. Before submitting your revision, primary datasets produced in this study need to be deposited in an appropriate public database, and the accession numbers and database listed under 'Data Availability'. Please remember to provide a reviewer password if the datasets are not yet public (see https://link.springer.com/partners/embo-press/editorial-policies#Data%20deposition). If no data deposition in external databases is needed for this paper, please then state in this section: This study includes no data deposited in external repositories. Note that the Data Availability Section is restricted to new primary data that are part of this study.

Note - All links should resolve to a page where the data can be accessed.

7) When assembling figures, please refer to our figure preparation guideline in order to ensure proper formatting and readability in print as well as on screen:
https://link.springer.com/journal/44318/submission-guidelines#cms-Figure-and-data-presentation

Please remember: Digital image enhancement is acceptable practice, as long as it accurately represents the original data and conforms to community standards. If a figure has been subjected to significant electronic manipulation, this must be noted in the figure legend or in the 'Materials and Methods' section. The editors reserve the right to request original versions of figures and the original images that were used to assemble the figure.

8) For data quantification: please specify the name of the statistical test used to generate error bars and P values, the number (n) of independent experiments (specify technical or biological replicates) underlying each data point and the test used to calculate p-values in each figure legend. The figure legends should contain a basic description of n, P and the test applied. Graphs must include a description of the bars and the error bars (s.d., s.e.m.).

9) We would also encourage you to include the source data for figure panels that show essential data. Numerical data can be provided as individual .xls or .csv files (including a tab describing the data). For 'blots' or microscopy, uncropped images should be submitted (using a zip archive or a single pdf per main figure if multiple images need to be supplied for one panel). Additional information on source data and instruction on how to label the files are available at
https://link.springer.com/journal/44318/submission-guidelines#cms-Source-data

10) We replaced Supplementary Information with Expanded View (EV) Figures and Tables that are collapsible/expandable online (see examples in https://www.embopress.org/doi/10.15252/embj.201695874). A maximum of 5 EV Figures can be typeset. EV Figures should be cited as 'Figure EV1, Figure EV2" etc. in the text and their respective legends should be included in the main text after the legends of regular figures.

- For the figures that you do NOT wish to display as Expanded View figures, they should be bundled together with their legends in a single PDF file called *Appendix*, which should start with a short Table of Content. Appendix figures should be referred to in the main text as: "Appendix Figure S1, Appendix Figure S2" etc. See detailed instructions regarding expanded view here: < https://link.springer.com/journal/44318/submission-guidelines#cms-Expanded-View-data >.

- Additional Tables/Datasets should be labelled and referred to as Table EV1, Dataset EV1, etc. Legends have to be provided in a separate tab in case of .xls files. Alternatively, the legend can be supplied as a separate text file (README) and zipped together with the Table/Dataset file.

11) At EMBO Press we ask authors to provide source data for the main manuscript figures. Our source data coordinator will contact you to discuss which figure panels we would need source data for and will also provide you with helpful tips on how to upload and organize the files.

12) Our journal encourages inclusion of *data citations in the reference list* to directly cite datasets that were re-used and obtained from public databases. Data citations in the article text are distinct from normal bibliographical citations and should directly link to the database records from which the data can be accessed. In the main text, data citations are formatted as follows: "Data ref: Smith et al, 2001" or "Data ref: NCBI Sequence Read Archive PRJNA342805, 2017". In the Reference list, data citations must be labeled with "[DATASET]". A data reference must provide the database name, accession number/identifiers and a resolvable link to the landing page from which the data can be accessed at the end of the reference. Further instructions are available at < https://link.springer.com/journal/44318/submission-guidelines#cms-Reference-guidelines >.

13) In order to increase the reproducibility and reach of your work, The EMBO Journal includes a table of reagents that were used in the study. Please provide this along with your revisions.

Further instructions for preparing your revised manuscript:

Read our guidance for manuscript revisions and related editorial policies: https://link.springer.com/journal/44318/submission-guidelines#cms-Revised-submissions

Please make sure you upload a letter of response to the referees' comments together with the revised manuscript.

Please also check that the title and abstract of the manuscript are brief, yet explicit, even to non-specialists.

When assembling figures, please refer to our figure preparation guideline in order to ensure proper formatting and readability in print as well as on screen:
https://media.springernature.com/original/springer-cms/rest/v1/content/27825798/data/v1

At EMBO Press we ask authors to provide source data for the main manuscript figures. You will receive a separate email with instructions for providing source data with your revised manuscript, including how to upload and organize the files.

IMPORTANT: When you send the revision we will require
- a point-by-point response to the referees' comments, with a detailed description of the changes made (as a word file).
- a word file of the manuscript text.
- individual production quality figure files (one file per figure)
- a complete author checklist
- Expanded View files (replacing Supplementary Information)
- a Reagents and Tools Table as part of the Methods section

Please remember: Digital image enhancement is acceptable practice, as long as it accurately represents the original data and conforms to community standards. If a figure has been subjected to significant electronic manipulation, this must be noted in the figure legend or in the 'Materials and Methods' section. The editors reserve the right to request original versions of figures and the original images that were used to assemble the figure.

We realize that it is difficult to revise to a specific deadline. In the interest of protecting the conceptual advance provided by the work, we recommend a revision within 3 months (18th Mar 2026). Please discuss the revision progress ahead of this time with the editor if you require more time to complete the revisions. Use the link below to submit your revision:

https://emboj.msubmit.net/cgi-bin/main.plex

Referee #1:

Zhang and co-workers studied the role of ER-phagy in regulating the levels of APP. They found that the ER-phagy receptor FAM134B is down-regulated in the 5xFAD mouse model for Alzheimer's disease (AD) and confirmed this downregulation in post-mortem material from AD patients. They show that the reduced expression is due to epigenetic repression of the FAM134B gene. Not only expression levels but also ubiquitylation and oligomerization of FAM134B was reduced suggesting that it its function in ER-phagy is compromised. They found that APP is itself also a substrate for FAM134B mediated ER-phagy. The result is a feedback loop where APP reduces FAM134B levels, which on its turn causes an increase in APP levels due to impaired clearance by ER-phagy. There is no involvement of chaperones in the targeting of APP by FAM134B but this is instead the consequence of a direct interaction between the cytosolic domains of APP and FAM134B. Overexpression of FAM134B in 5xFAD mice decreases amyloid plaque formation and reduces pathology.

The authors put a considerable effort in approaching this question from different angles. The findings are surprising and likely to be of interest to the broad audience of EMBO Journal. The data are of high quality and overall strongly supportive of the model. As any good study it also raises new questions (such as the mechanism by which APP causes the epigenetic repression of FAM134B and regulation of the APP-FAM134B interaction). I would, however, consider those questions interesting for follow-up investigations but outside the scope of the current study. I have a few minor comments for the authors to consider.

Comments

The authors show that expression, ubiquitylation and oligomerization of FAM134B are reduced in cells expressing APP and in brains of 5xFAD mice. Can they conclude from their data that these effects are independent or could be that the reduction in ubiquitylation and oligomerization are mere a downstream consequence of the reduced levels of FAM134B?

The model suggests that under physiological conditions wild-type APP is constitutively and selectively targeted for degradation by ER-phagy. Since no chaperones are involved and a direct interaction between APP and FAM134B is responsible for the targeting, it seems unlikely that this is a protein quality process. What could be the functional significance of this process? Are there any indications that this process is regulated? Constitutive degradation of a constitutive produced protein and the high energy expenditure that comes with it seems to be hard to explain in physiological terms.

It may be helpful if the schematic drawing of the model shown in Figure S7E is moved to the main figures.

Referee #2:

In their manuscript 'FAM134B-mediated ER-phagy degrades APP and protects against Alzheimer's disease pathology' Cui and colleagues identified FAM134B as a key endoplasmic reticulum (ER)-phagy receptor responsible for degrading amyloid precursor protein (APP). They showed FAM134B to directly interacting with APP through its C-terminal domain and facilitate its lysosomal degradation in a LIR dependent manner. In sample of Alzheimer's disease (AD) patients, FAM134B expression is low, both on protein as well as RNA level. The authors showed epigenetic silencing due to reduced chromatin accessibility and loss of activating histone marks at its promoter. Therefore TFEB, despite being upregulated, is prevented from binding effectively, leading to decreased FAM134B transcription. In a 5XFAD mouse model, restoring wild-type FAM134B expression reactivates ER-phagy, the manuscript shows increased APP and Aβ clearance, and improved cognitive function. In contrast, a LIR-mutant FAM134B fails to confer these benefits. Overall, the authors demonstrate that FAM134B downregulation represents an upstream pathogenic mechanism in AD, highlighting ER-phagy enhancement as a potential therapeutic strategy to suppress pathology.

Overall, the study presents a high-quality data set that supports the main claims. The study is timely and of high interest for the readership of EMBO J. I recommend it for publication after a carful revision on several points indicated below.

Some general major comments
1) The data is extremely dense. From what I can see, there is little difference in the cell model data between APPwt and APPmut. And the authors also do not discuss the small existing differences. The data on APPmut is of course interesting as such, but at the moment does not have real impact on the main conclusion of the paper. It would probably be easier for the reader to get this information somewhere early on, like in the abstract or at the summary paragraph at the end of the introduction. Like this, they can focus on the other aspects.
There are few not as obvious differences - Fig. S2E (NC), Fig. 1J (p62 levels), Fig. S3 (APP levels) - that in my opinion hint to a different localization and/or accessibility of wt vs. mut form by the cytosolic quality control machinery (including p62 and potentially ERAD) and may explain the increased pathological impact of the mutant. Also to note in this context - the mutations (K670N, M671L, I716V, V717I) are all localized in the C-terminus of APP that the authors identified as the interaction site with FAM134B and which could as well be a target site for other cytosolic quality control factors. Unfortunately, the interaction studies are one of the few experiments not performed with APPmut. In case the authors can follow my argumentation, I suggest to include a respective paragraph in the discussion section (or repeat the interaction study with the mutant form to gain certainty).
2) It is unclear to me, why the authors after Fig. S1 suddenly treated their cellular model with Baf or EBSS+Baf before IF. Based on their currently provided data set, it is even unclear, how e.g. FAM134B levels react to EBSS treatment. Are their changes in expression on RNA in the cellular model? If the EBSS/Baf treatment is needed to push the system and get clearer images, this is perfectly ok and needs to be stated/explained clearly in the text.
3) Please provide in the figure legends of all figures/panels: the exact cell line (U2OS, HeLa, ...) used (in case modified, stable or transient), number of n/N, definition of error bar. I would suggest to use SD instead of SEM, as it is more appropriately showing the variance of biological samples.

Detailed points:

- Fig. S1E/S1G/M&M
It is not clear what the authors define as enhanced fluorescence (cut off?), what they exactly normalized to, as well as which minimum number of cells were tested (since they used FACS probably many). Instead of a rather undefined percentage, the authors should just plot the received average RFP/GFP intensity ratio as an arbitrary unit [au] together with the standard deviation.
The M&M section states specifically/only mCherry as the red fluorophore. Please correct.

- Fig. S1F/S2F:
I could not find how the mCherry+/EGFP- puncta were obtained: manually or semi-automatic? Please define in the M&M.

- Fig. S2A/D:
Overexpressed APP localization should be (also) shown in IF under basal conditions (no Baf, no EBSS), as corresponding info to 1H and I. And under these basal conditions analyzed for colocalization with Calnexin and LAMP1.

- Fig. S2E / 183f:

I disagree with the statement in lane 184 that APPWT/mut-mCherry-EGFP displayed a typical ER distribution. Based on their data, the signal assigned to overexpressed APPwt and (less extent) mut seems to be also cytosolic.
The remaining green signal after EBSS treatment in the APPmut seems to be more ER localized than the corresponding APPwt signal. However, this is difficult to judge from a single cell. In this context - are the gradients (Fig. S2B/C) indeed done with non-treated cells (the figure legends would imply that, but do not explicitly state that the cells were non-treated) or like the IF with EBSS+Baf treated cells? Are these from the same cell type as for IF? If not, please repeat the gradient (Fig. S2B) with the very same cells as in S2E and also blotted against GFP or mCherry.

- Fig. 2B, E-H: as above for Fig. S1E.

- Fig. 2C / lane 199ff:
as for Fig. 2B. Are these stable cell lines? This may impact the statistics depending on how the normalization has been done and if non-expressing cells are included in the calculation. Statistics are probably t-test with n=3. Would the authors agree that by increasing the sample size, RTN3L or CALCOCO1 overexpression (quite big SEM compared to other tested samples) may also show significant effects? If so, please reflect this in the text.

- Lane 204ff / Fig. S3D/E:
Contradiction between text and actual data. Cleavage (based on mCherry signal alone) was affected by MG132 in WT+EBSS as well as mut- basal. Also unclear why mut+MG+Baf has no effect.
Also not clear, why are APP levels vary this much. Both patterns - APP and mCherry - individually and in relation to each other don't follow any logic mentioned in the text. It is e.g. notable that EBSS treatment dramatically changed the full-length levels of WT but not mut APP, even though the signal for cleaved mCherry did increase in both cases. And APPwt levels already drop drastically upon MG132 treatment and MG+Baf under nutrient rich conditions, while mCherry levels do not increase upon MG treatment. Might this correlate with the different distribution indicated by Fig. S2E under basal conditions? If the authors do not want to discuss this, they may want to remove all cleavage assays from the manuscript.

- I suggest to mention RETREG1 as an alternative name for FAM134B in the abstract to improve future findability.

Referee #3:

In this manuscript, Zhang et al. identify FAM134B as a mediator of amylod precursor protein (APP) degradation, via FAM134B-APP direct binding and lysosomal delivery via ER-phagy. They found that FAM134B downregulation in cellular and mouse models of Alzheimer's disease (AD) impairs ER-phagy, leading to APP accumulation. The further show that AAV-mediated expression of FAM134B in the hippocampus of an AD mouse model promotes APP clearance and improves cognitive phenotypes.

While the manuscript is potentially interesting (particularly for the in vivo findings), it raises conceptual concerns and the main message is not clearly articulated. Specifically, the authors report that: i) FAM134B-mediated ER-phagy of APP is impaired in AD models due to different mechanisms, including reduced TFEB/TFE3-driven transcription of FAM134B, reduced FAM134B oligomerization, and reduced ubiquitination; ii) APP is degraded via FAM134B-mediated ER-phagy, but this occurs during starvation, and it is unclear why both mutant and wild-type newly synthesized APP should be degraded as part of a quality-control mechanism; iii) FAM134B oligomerization and ubiquitination are impaired, but the underlying reason is not addressed; and iv) FAM134B and APP interact, but the functional significance of this interaction remains unclear. As a result, the manuscript presents several partially supported claims that do not converge into a coherent and robust conceptual flow, leaving the reader uncertain about the central conclusion of the study.

Additional major concerns:

1- Whether mutant APP is a true ER-phagy substrate remains unclear, as most experiments have been performed in APP-overexpressing cells, which may lead to confounding effects due to ER overload. Furthermore, during nutrient starvation, many ER-resident proteins are delivered to lysosomes for degradation, although they are not considered "canonical" ER-phagy substrates. It is also unclear why both wild-type and mutant APP are degraded via ER-phagy to a similar extent. Because APP overexpression can overload the ER, the observation that both WT and mutant APP follow similar degradation routes raises the possibility that the phenotype may simply reflect ER overload rather than selective ER-phagy of APP.

2- The mechanism underlying defective ubiquitination and impaired oligomerization is not clarified. While these defects could be a secondary consequence of reduced FAM134B abundance in the mouse brain, it remains unclear why overexpressed FAM134B in cells still fails to undergo proper ubiquitination and oligomerization.

3- FAM134B-APP interaction. The C-terminal domain of FAM134B is an intrinsically disordered region lacking known structured domains, making a direct interaction with APP difficult to envision. The authors should perform pull-down assays using truncated FAM134B constructs to narrow the region(s) required for interaction with APP.

4- The mechanism linking APP overexpression to epigenetic silencing of FAM134B has not been investigated.

5- Does FAM134B silencing in the mouse model occur pre- or post-symptomatically? Do the authors detect activation of the ER-stress response in these tissues?

6- It would be important to test whether BafA treatment rescues the reduction of FAM134B levels, which could be explained by increased FAM134B degradation. Indeed, the APP-induced transcriptional downregulation of FAM134B, albeit statistically significant, appears minimal.

7- It is unclear which FAM134B isoform is regulated by TFEB/TFE3 in cultured cells or in vivo. The authors should clarify this point, particularly in relation to the reported epigenetic changes.

8- The in vivo results are compelling, but the authors examined neuronal cell bodies, synapses, and myelin without demonstrating that these cells were indeed transduced by AAV-FAM134B. Since AAV does not efficiently transduce most cells, the authors should include co-staining to confirm FAM134B expression in the analysed regions.

9- In the introduction there is a misconception that ER-phagy is associated with neurodegeneration. This is not supported by current evidence, at least in humans. Instead, ER-phagy alterations appear to be linked primarily to neuropathy and possibly to defects in neurogenesis, but not directly to neurodegeneration.

Dear Dr. Teale,

Thank you for your letter and the valuable feedback provided by the reviewers regarding our manuscript entitled "FAM134B-mediated ER-phagy degrades APP and protects against Alzheimer's disease pathology" (EMBOJ-2025-122653). We greatly appreciate the reviewers' time and effort in evaluating our work and have carefully considered each of their comments to conduct additional experiments to address their concerns. Below, we present a detailed response to each point raised by the reviewers. We are grateful for the opportunity to address these issues and improve the quality of our manuscript.

Our point-by-point responses are provided below:

Referee #1:
Zhang and co-workers studied the role of ER-phagy in regulating the levels of APP. They found that the ER-phagy receptor FAM134B is down-regulated in the 5xFAD mouse model for Alzheimer's disease (AD) and confirmed this downregulation in post-mortem material from AD patients. They show that the reduced expression is due to epigenetic repression of the FAM134B gene. Not only expression levels but also ubiquitylation and oligomerization of FAM134B was reduced suggesting that it its function in ER-phagy is compromised. They found that APP is itself also a substrate for FAM134B mediated ER-phagy. The result is a feedback loop where APP reduces FAM134B levels, which on its turn causes an increase in APP levels due to impaired clearance by ER-phagy. There is no involvement of chaperones in the targeting of APP by FAM134B but this is instead the consequence of a direct interaction between the cytosolic domains of APP and FAM134B. Overexpression of FAM134B in 5xFAD mice decreases amyloid plaque formation and reduces pathology.

The authors put a considerable effort in approaching this question from different angles. The findings are surprising and likely to be of interest to the broad audience of EMBO Journal. The data are of high quality and overall strongly supportive of the model. As any good study it also raises new questions (such as the mechanism by which APP causes the epigenetic repression of FAM134B and regulation of the APP-FAM134B interaction). I would, however, consider those questions interesting for follow-up investigations but outside the scope of the current study. I have a few minor comments for the authors to consider.

Comments

The authors show that expression, ubiquitylation and oligomerization of FAM134B are

reduced in cells expressing APP and in brains of 5xFAD mice. Can they conclude from their data that these effects are independent or could be that the reduction in ubiquitylation and oligomerization are mere a downstream consequence of the reduced levels of FAM134B?

We appreciate your insightful comment regarding the causal relationship between FAM134B abundance and its ubiquitylation and oligomerization. Notably, our original dataset (original Fig. S1H,J; now Fig. EV2J,K) already indicates that these defects cannot be explained solely by reduced FAM134B levels. In these experiments, EGFP-tagged FAM134B was transiently expressed at comparable levels in control and APP-expressing cells, yet its ubiquitylation and oligomerization were markedly reduced. This observation indicates that APP expression compromises FAM134B ER-phagy receptor activity by impairing its ubiquitylation and oligomerization through a mechanism at least partly independent of protein abundance.

To investigate the underlying mechanism, we examined post-translational modifications of FAM134B known to regulate its oligomerization. Phosphorylation of FAM134B at Ser151 promotes acetylation at Lys160, enhancing its oligomerization (Jiang *et al*, 2020; Wang *et al*, 2023). AMFR-mediated ubiquitination also facilitates oligomerization, driving ER fragmentation and ER-phagy (Gonzalez *et al*, 2023). Immunoblotting revealed that Ser151 phosphorylation and Lys160 acetylation were both significantly reduced in 5XFAD mouse brains, accompanied by decreased AMFR levels (Fig. EV2E,F). Consistently, FAM134B ubiquitylation and oligomerization were also reduced (original Fig. S1I,K; now Fig. EV2G,H), suggesting impaired post-translational regulation. Together, these data indicate that FAM134B receptor activity is compromised in 5XFAD mice.

Although FAM134B protein levels are reduced in 5XFAD mice (Fig. 1G,H; EV2E,F), these defects could still theoretically arise as a secondary consequence of decreased abundance. To address this possibility, we expressed 3HA-FAM134B in APP-overexpressing cells to equalize FAM134B levels. Consistent with our original observations (Fig. S1H,J; now Fig. EV2J,K), Ser151 phosphorylation and Lys160 acetylation, as well as AMFR levels, remained reduced in both wild-type and mutant APP-expressing cells despite comparable FAM134B expression (Fig. EV2I). These results demonstrate that the impaired ubiquitylation and oligomerization of FAM134B are not merely a downstream consequence of reduced protein levels but instead reflect altered post-translational regulation.

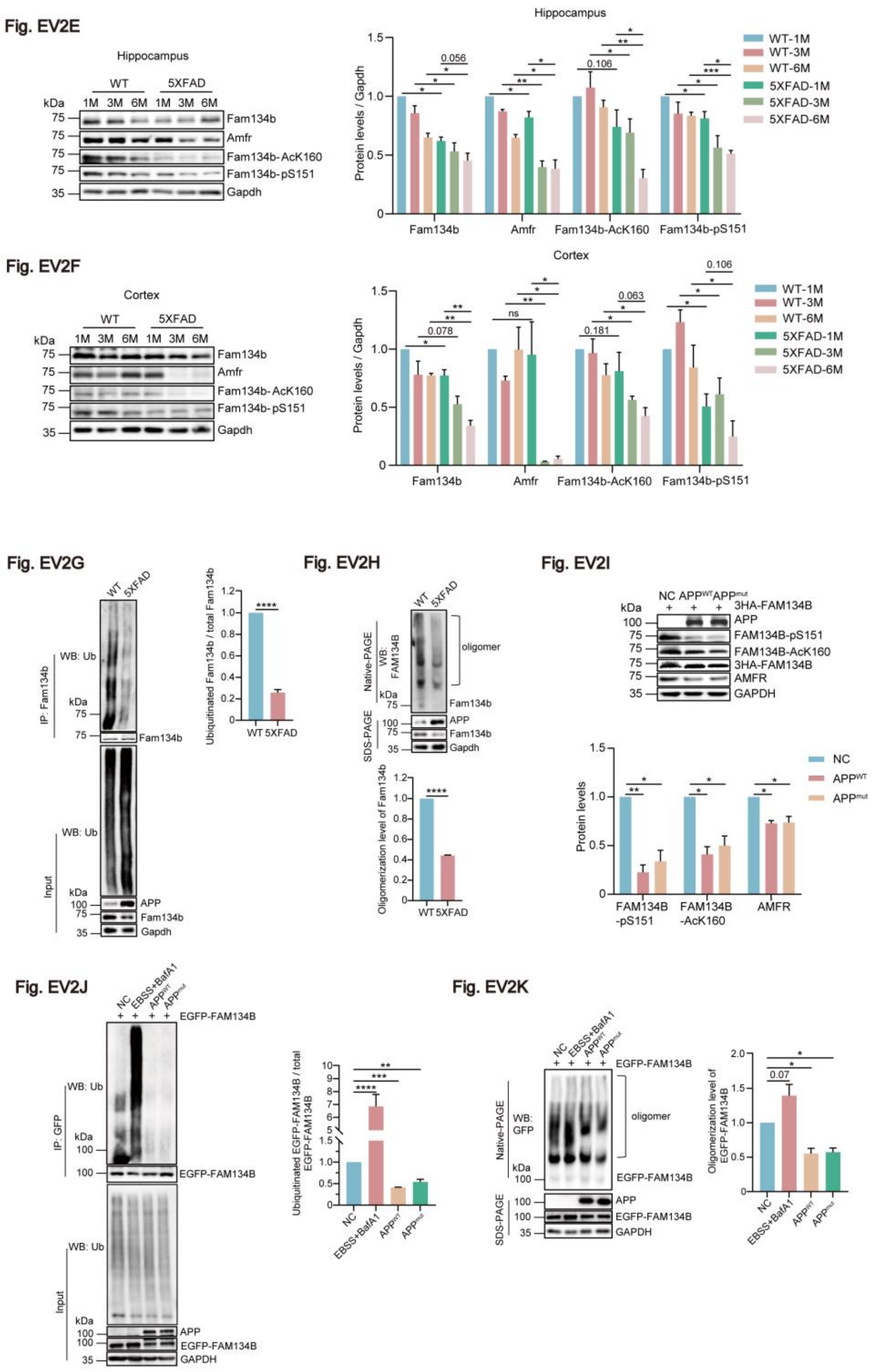

(**EV2E,F**) Immunoblotting of protein levels in the hippocampus (**E**) and cortex (**F**) of 1-, 3-, and 6-month-old 5XFAD female mice and WT littermates (*N* = 3 per group). Fam134b-pS151 and Fam134b-AcK160 indicate Fam134b phosphorylated at Ser151 and acetylated at Lys160, respectively.

(**EV2G**) Fam134b ubiquitination levels in the hippocampus of 6-month-old WT and 5XFAD female mice (*N* = 3 per group). Fam134b was immunoprecipitated with anti-Fam134b

antibodies and analyzed by immunoblotting with anti-ubiquitin antibodies. Right: Quantification of ubiquitinated Fam134b normalized to total Fam134b.

(**EV2H**) Fam134b oligomerization levels in the hippocampus of 6-month-old WT and 5XFAD female mice (*N* = 3 per group). Top: Native PAGE immunoblotting of Fam134b using anti-Fam134b antibodies. Input protein levels were assessed by SDS-PAGE and immunoblotting. Bottom: Quantification of oligomerized Fam134b normalized to total Fam134b.

(**EV2I**) Immunoblotting of protein levels in control and HEK293T cells expressing DOX-inducible $APP^{WT/mut}$. Cells were cultured in nutrient-rich medium and transiently transfected with 3HA–FAM134B. Bottom: Quantification of protein levels shown above. FAM134B-pS151 and FAM134B-AcK160 were normalized to total 3HA–FAM134B; AMFR was normalized to GAPDH. *n* = 3.

(**EV2J**) Effect of $APP^{WT/mut}$ overexpression on FAM134B ubiquitination. Left: EGFP-FAM134B was immunoprecipitated with anti-GFP antibodies and analyzed by immunoblotting with anti-ubiquitin antibodies. Lysates were prepared from untreated U2OS cells (NC), EBSS + BafA1–treated U2OS cells (6 h; positive control), and DOX-induced APPWT/mut-expressing U2OS cells cultured in nutrient-rich medium. All groups were transiently transfected with EGFP-FAM134B. Right: Quantification of ubiquitinated EGFP-FAM134B normalized to total EGFP-FAM134B. *n* = 3.

(**EV2K**) Effect of $APP^{WT/mut}$ overexpression on FAM134B oligomerization. Left: Native PAGE immunoblotting of EGFP-FAM134B using anti-GFP antibodies. Lysates were prepared from NC, EBSS + BafA1, and DOX-induced $APP^{WT/mut}$ U2OS cells. Input protein levels were assessed by SDS-PAGE and immunoblotting. Right: Quantification of oligomerized EGFP-FAM134B normalized to total EGFP-FAM134B. *n* = 3.

The model suggests that under physiological conditions wild-type APP is constitutively and selectively targeted for degradation by ER-phagy. Since no chaperones are involved and a direct interaction between APP and FAM134B is responsible for the targeting, it seems unlikely that this is a protein quality process. What could be the functional significance of this process? Are there any indications that this process is regulated? Constitutive degradation of a constitutive produced protein and the high energy expenditure that comes with it seems to be hard to explain in physiological terms.

We appreciate your thoughtful comments. Although FAM134B-mediated ER-phagy has often been associated with chaperone-assisted substrate recognition, chaperone dependence is not a defining feature of protein quality control. Notably, ER-phagy receptors such as CCPG1 can directly recognize both aggregation-prone substrates (e.g.,

six-repeated islet amyloid polypeptide, 6xIAPP) and endogenous ER luminal proteins (e.g., prolyl 3-hydroxylase family member 4, P3H4), indicating that direct receptor–substrate interactions contribute to selective ER-phagy and ER quality control (Ishii *et al*, 2023). Thus, the absence of chaperone involvement does not preclude APP degradation from representing a quality-control process.

Regarding the functional significance, we propose that FAM134B may preferentially target APP molecules that are retained in the ER, fail to enter trafficking pathways, or adopt non-productive conformations, thereby extending the concept of ER quality control beyond the clearance of overtly misfolded proteins. Although constitutive degradation of a physiologically expressed protein may appear energetically costly, such turnover could serve as a preventive mechanism to limit the generation of toxic APP-derived intermediates or to dynamically regulate the available APP pool in response to cellular demands. We have revised the Discussion accordingly.

With respect to regulation, we examined whether the APP–FAM134B interaction depends on post-translational modifications of FAM134B. Mutants that prevent phosphorylation (S151A) or acetylation (K160R) showed a tendency toward reduced APP binding, whereas phospho-mimetic (S151D) or acetylation-mimetic (K160Q) substitutions did not significantly enhance the interaction between APP and FAM134B. Notably, APP binding is mediated primarily by the C-terminal region (aa 233–497) of FAM134B (Fig. 3G,H,J), which is spatially distinct from these modification sites. These observations therefore suggest that these modifications regulate FAM134B receptor activity rather than substrate recognition, likely modulating ER-phagy efficiency and thereby indirectly affecting APP degradation rather than controlling APP binding *per se*. The precise regulatory mechanisms remain to be investigated in future studies.

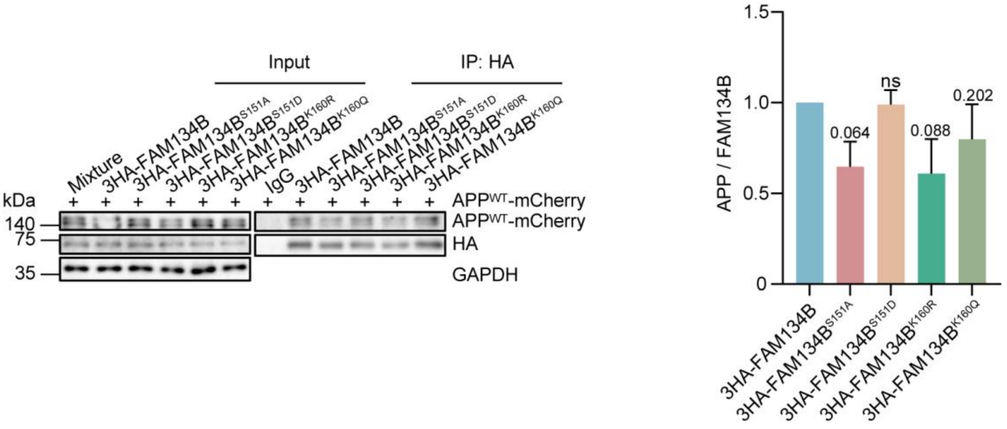

Left: Co-IP of APP with wild-type and mutant FAM134B. HEK293T cells were transiently co-transfected with APP^{WT}-mCherry and the indicated FAM134B constructs, followed by immunoprecipitation with anti-HA antibodies. IgG pulldown of mixed FAM134B lysates

served as a negative control. **Right:** Quantification of APP co-immunoprecipitated with wild-type and mutant FAM134B. *n* = 3.

It may be helpful if the schematic drawing of the model shown in Figure S7E is moved to the main figures.

We thank you for the suggestion. The schematic model shown in Figure S7E has been moved to the main figures (now Fig. 6H).

Referee #2:

In their manuscript 'FAM134B-mediated ER-phagy degrades APP and protects against Alzheimer's disease pathology' Cui and colleagues identified FAM134B as a key endoplasmic reticulum (ER)-phagy receptor responsible for degrading amyloid precursor protein (APP). They showed FAM134B to directly interacting with APP through its C-terminal domain and facilitate its lysosomal degradation in a LIR dependent manner. In sample of Alzheimer's disease (AD) patients, FAM134B expression is low, both on protein as well as RNA level. The authors showed epigenetic silencing due to reduced chromatin accessibility and loss of activating histone marks at its promoter. Therefore TFEB, despite being upregulated, is prevented from binding effectively, leading to decreased FAM134B transcription. In a 5XFAD mouse model, restoring wild-type FAM134B expression reactivates ER-phagy, the manuscript shows increased APP and Aβ clearance, and improved cognitive function. In contrast, a LIR-mutant FAM134B fails to confer these benefits. Overall, the authors demonstrate that FAM134B downregulation represents an upstream pathogenic mechanism in AD, highlighting ER-phagy enhancement as a potential therapeutic strategy to suppress pathology.

Overall, the study presents a high-quality data set that supports the main claims. The study is timely and of high interest for the readership of EMBO J. I recommend it for publication after a carful revision on several points indicated below.

Some general major comments

1) The data is extremely dense. From what I can see, there is little difference in the cell model data between APPwt and APPmut. And the authors also do not discuss the small existing differences. The data on APPmut is of course interesting as such, but at the moment does not have real impact on the main conclusion of the paper. It would probably be easier for the reader to get this information somewhere early on, like in the abstract or at the summary paragraph at the end of the introduction. Like this, they can focus on the

other aspects.

We thank you for this helpful suggestion. To improve clarity, we have added a statement at the end of the Introduction noting that **familial APP mutants behave similarly to wild-type APP**.

There are few not as obvious differences - Fig. S2E (NC), Fig. 1J (p62 levels), Fig. S3 (APP levels) - that in my opinion hint to a different localization and/or accessibility of wt vs. mut form by the cytosolic quality control machinery (including p62 and potentially ERAD) and may explain the increased pathological impact of the mutant. Also to note in this context - the mutations (K670N, M671L, I716V, V717I) are all localized in the C-terminus of APP that the authors identified as the interaction site with FAM134B and which could as well be a target site for other cytosolic quality control factors. Unfortunately, the interaction studies are one of the few experiments not performed with APPmut. In case the authors can follow my argumentation, I suggest to include a respective paragraph in the discussion section (or repeat the interaction study with the mutant form to gain certainty).

We agree that the subtle differences observed between APP^{WT} and APP^{mut} may reflect altered localization or accessibility to cytosolic quality-control machinery, potentially contributing to the increased pathological impact of the mutant APP. Accordingly, we have added the following contents to the Discussion:

"Despite their shared dependence on FAM134B-dependent ER-phagy, wild-type and familial mutant APP display subtle differences that suggest distinct engagement of cytosolic quality-control pathways. Wild-type APP overexpression reduces p62 levels, whereas mutant APP shows little effect, and the two forms respond differently to proteasome inhibition. These observations indicate differential accessibility to p62-mediated autophagy and potentially ER-associated degradation. Such variation may influence the relative pathogenicity of mutant APP despite their similar regulation by ER-phagy."

Importantly, the Swedish (K670N/M671L), Florida (I716V), and London (V717I) mutations are numbered according to the APP770 sequence and correspond to **K595N/M596L, I641V, and V642I in APP695**. Our mapping analysis shows that FAM134B interacts with the cytosolic C-terminal region of APP695 (aa 649–695, Fig. 3J), whereas these mutations lie outside this interaction domain, explaining why FAM134B can mediate degradation of both wild-type and mutant APP. To clarify this point, we have annotated the mutation positions in Fig. 3F.

Fig. 3F

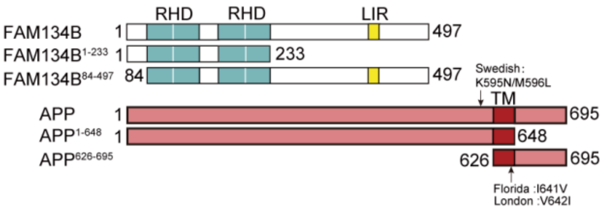

Fig. 3J

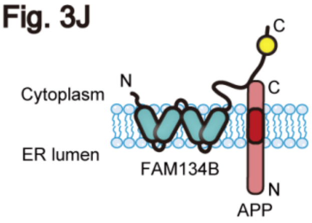

(**3F**) Schematic of full-length and truncated FAM134B and APP constructs. Domains shown include RHD (reticulon homology domain), LIR (LC3-interacting region), and TM (transmembrane domain); APP695 with the Swedish (K670N/M671L), Florida (I716V), and London (V717I) mutations (APP770 numbering; corresponding to APP695 K595N/M596L, I641V, and V642I).

(**3J**) Schematic of FAM134B and APP binding *via* their C termini on the ER membrane.

2) It is unclear to me, why the authors after Fig. S1 suddenly treated their cellular model with Baf or EBSS+Baf before IF. Based on their currently provided data set, it is even unclear, how e.g. FAM134B levels react to EBSS treatment. Are their changes in expression on RNA in the cellular model? If the EBSS/Baf treatment is needed to push the system and get clearer images, this is perfectly ok and needs to be stated/explained clearly in the text.

We thank the reviewer for this insightful comment and apologize for not clearly explaining the rationale for the EBSS and BafA1 treatments in the original manuscript. In response, we examined *FAM134B* transcription under starvation conditions. qRT-PCR analysis showed that EBSS significantly increased *FAM134B* mRNA levels at an early time point (Fig. EV4F), consistent with the induction pattern observed for other selective autophagy regulators, such as UVRAG and TM9SF3 (Qian *et al*, 2023; Yang *et al*, 2025), thereby supporting transcriptional activation. Moreover, EBSS enhanced FAM134B oligomerization and ubiquitination (now Fig. EV2J,K; original Fig. S1H,J), modifications that promote its receptor activity. Therefore, EBSS was used to activate FAM134B-dependent ER-phagy, while BafA1 was applied to block lysosomal degradation, allowing clearer visualization of APP colocalization with autophagosomes and lysosomes. These points have now been clarified in the revised manuscript.

Fig. EV4F

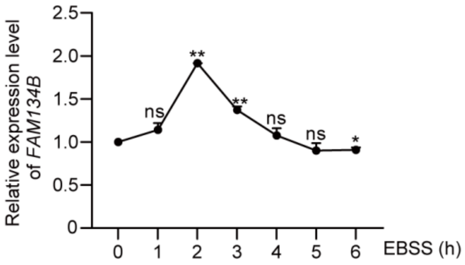

(**EV4F**) qRT-PCR analysis of *FAM134B* mRNA levels in U2OS cells, either untreated or treated with EBSS for the indicated times. $n = 3$.

3) Please provide in the figure legends of all figures/panels: the exact cell line (U2OS, HeLa, ...) used (in case modified, stable or transient), number of n/N, definition of error bar. I would suggest to use SD instead of SEM, as it is more appropriately showing the variance of biological samples.

We thank you for this suggestion. We have now updated the figure legends for all figures/panels to include the exact cell line used, specifying whether the cells are modified, stable, or transient, as well as the number of replicates and the definition of the error bars. Regarding the use of SEM instead of SD, we would like to clarify our rationale for maintaining the current presentation. In this study, statistical inference was based on formal hypothesis testing rather than visual inspection of error bars. Therefore, the representation of variability as SEM versus SD does not affect statistical significance or the conclusions of the study. We used SEM to reflect the precision of the estimated mean and have applied this consistently across all figures. Changing the error bar format at this stage would affect consistency across the manuscript without altering the interpretation of the data. We hope this clarifies our reasoning, and we would appreciate your understanding.

Detailed points:

- Fig. S1E/S1G/M&M

It is not clear what the authors define as enhanced fluorescence (cut off?), what they exactly normalized to, as well as which minimum number of cells were tested (since they used FACS probably many). Instead of a rather undefined percentage, the authors should just plot the received average RFP/GFP intensity ratio as an arbitrary unit [au] together with the standard deviation.

We are grateful to you for the insightful comments. Accordingly, we have refined the

Methods section as described below.

Autophagy flux analysis by flow cytometry

U2OS cells stably expressing mCherry-EGFP-RAMP4 or RFP-EGFP-KDEL, as well as cells expressing DOX-inducible APP$^{WT/mut}$-mCherry-EGFP, were pelleted at 2000 ×g for 1 min and resuspended in PBS. Samples were analyzed on a CytoFlex LX flow cytometer (Beckman Coulter) equipped with 488-nm and 561-nm lasers. EGFP fluorescence was collected using a 530/30-nm filter, and mCherry/RFP fluorescence using a 610/20-nm filter. Data were processed in FlowJo. Live single cells were gated based on FSC-A/SSC-A and FSC-H/FSC-A parameters. Cells positive for both EGFP and mCherry were selected, and at least 10,000 events were acquired per sample. Autophagic flux was quantified as the percentage of cells exhibiting an increased red-to-green fluorescence ratio (mCherry/EGFP or RFP/EGFP), reflecting quenching of EGFP in acidic lysosomes. The gate defining an "enhanced ratio" was established based on control cells cultured in nutrient-rich medium and was then applied uniformly to all samples. Data are presented as mean ± SEM from at least three independent experiments, as indicated in the corresponding figure legends.

- Fig. S1F/S2F:

I could not find how the mCherry+/EGFP- puncta were obtained: manually or semi-automatic? Please define in the M&M.

We thank the reviewer for pointing out this important methodological omission. We have now added a subsection entitled "**Quantification of microscopy images to evaluate autophagic flux**" to the Methods. U2OS cells stably expressing RFP-EGFP-KDEL or DOX-inducible APP$^{WT/mut}$-mCherry-EGFP were randomly selected from three independent experiments, and mCherry^{+}/EGFP^{-} puncta were manually quantified per cell. Image analysis was performed in a blinded manner using identical criteria across all samples. Data are presented as mean ± SEM, with the number of analyzed cells indicated in the corresponding figure legends (*N* = xx cells).

- Fig. S2A/D:

Overexpressed APP localization should be (also) shown in IF under basal conditions (no Baf, no EBSS), as corresponding info to 1H and I. And under these basal conditions analyzed for colocalization with Calnexin and LAMP1.

We thank the reviewer for this helpful suggestion. We have now added immunofluorescence images showing the localization of overexpressed APP under basal (nutrient-rich) conditions without BafA1 or EBSS (new Fig. EV3A). Previously, for

lysosomal colocalization analysis, only EBSS + BafA1 conditions were shown; we now additionally include BafA1 treatment under nutrient-rich conditions to visualize the basal level of lysosomal delivery of APP (new Fig. EV3D,E). BafA1 was applied under both nutrient-rich and starvation conditions to inhibit lysosomal degradation and enable visualization of ER-localized APP overlap with LAMP1-positive compartments.

Fig. EV3A

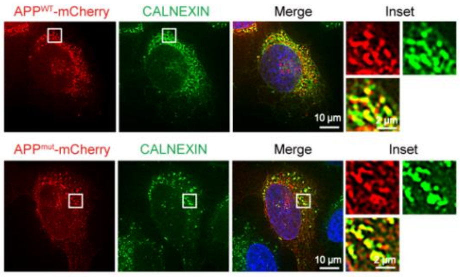

Fig. EV3D

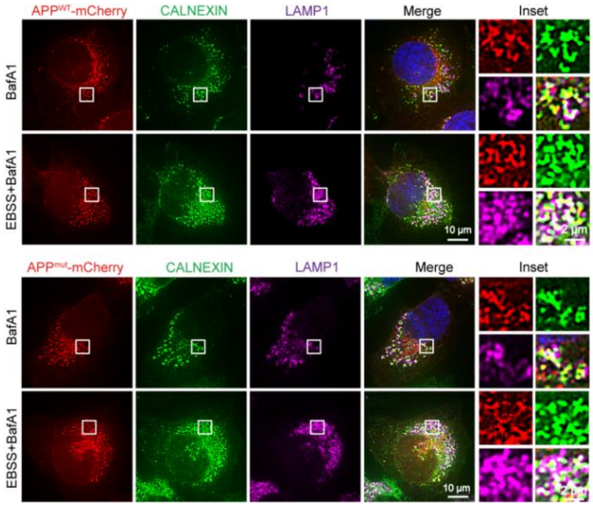

Fig. EV3E

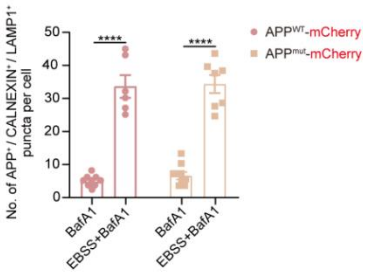

(**EV3A**) Multi-SIM images showing colocalization of $APP^{WT/mut}$-mCherry with CALNEXIN. U2OS cells expressing DOX-inducible $APP^{WT/mut}$-mCherry were cultured in nutrient-rich medium. CALNEXIN was detected by immunostaining.

(**EV3D**) Multi-SIM images showing colocalization of APP with CALNEXIN and LAMP1. U2OS cells expressing DOX-inducible $APP^{WT/mut}$-mCherry were treated with BafA1 or EBSS + BafA1 for 6 h. CALNEXIN and LAMP1 were detected by immunostaining.

(**EV3E**) Quantification of APP^{+}/$CALNEXIN^{+}$/$LAMP1^{+}$ puncta in (**D**). *N* = 10 cells (each BafA1 group), *N* = 6 cells (APP^{WT}-mCherry, EBSS + BafA1), *N* = 7 cells (APP^{mut}-mCherry, EBSS + BafA1).

- Fig. S2E / 183f:

I disagree with the statement in lane 184 that APPWT/mut-mCherry-EGFP displayed a typical ER distribution. Based on their data, the signal assigned to overexpressed APPwt

and (less extent) mut seems to be also cytosolic.

The remaining green signal after EBSS treatment in the APPmut seems to be more ER localized than the corresponding APPwt signal. However, this is difficult to judge from a single cell. In this context - are the gradients (Fig. S2B/C) indeed done with non-treated cells (the figure legends would imply that, but do not explicitly state that the cells were non-treated) or like the IF with EBSS+Baf treated cells? Are these from the same cell type as for IF? If not, please repeat the gradient (Fig. S2B) with the very same cells as in S2E and also blotted against GFP or mCherry.

We thank the reviewer for the comment. The statement in the original manuscript (line 184) regarding the "typical ER distribution" of $APP^{WT/mut}$-mCherry-EGFP was made based on fixed cells imaged by confocal microscopy. However, we acknowledge that fixation could disrupt the ER network. To address this, we have now switched to live-cell imaging, which provides a clearer view of the ER network. We observed that both APP^{WT} and APP^{mut} exhibited a predominantly ER distribution under both nutrient-rich and EBSS conditions (new Fig. EV3F,G). Moreover, our colocalization analysis using ER marker CALNEXIN supports the colocalization between APP and the ER (Fig. EV3A,D). In addition, our fractionation assay revealed co-fractionation of APP and ER markers (Fig. EV3B,C). While we have not excluded the possibility of APP also being localized to other cytosolic cellular compartments involved in trafficking, our data indicate a predominant ER localization. To avoid potential overinterpretation, we have removed the statement “APPWT/mut-mCherry-EGFP displayed a typical ER distribution” from the revised manuscript.

Fig. EV3F

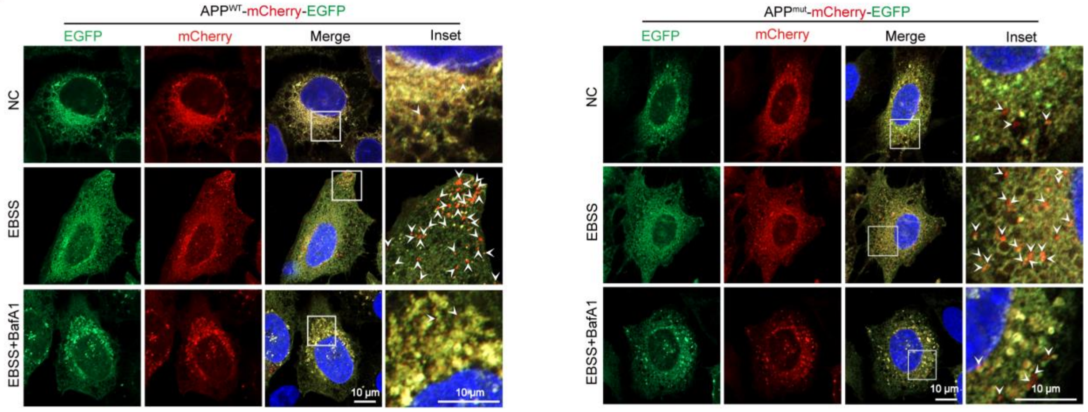

Fig. EV3G

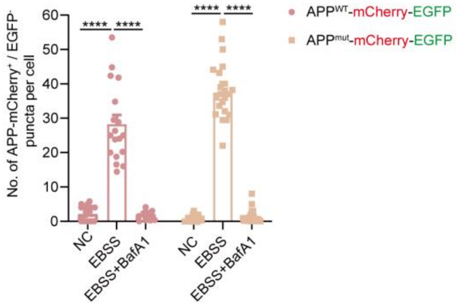

(**EV3F**) Live-cell confocal images of U2OS cells expressing DOX-inducible $APP^{WT/mut}$-mCherry-EGFP, either untreated or treated with EBSS, or EBSS + BafA1 for 6 h. Arrowheads indicate $mCherry^{+}/EGFP^{-}$ puncta.

(**EV3G**) Quantification of $mCherry^{+}/EGFP^{-}$ puncta in (**F**). *N* = 17 cells (APP^{WT}-mCherry-EGFP, NC); *N* = 25 cells (APP^{mut}-mCherry-EGFP, NC); *N* = 18 cells (APP^{WT}-mCherry-EGFP, EBSS); *N* = 25 cells (APP^{mut}-mCherry-EGFP, EBSS); *N* = 20 cells (APP^{WT}-mCherry-EGFP, EBSS + BafA1); *N* = 30 cells (APP^{mut}-mCherry-EGFP, EBSS + BafA1).

To clarify, the gradients in original Fig. S2B/C were conducted using untreated U2OS cells expressing DOX-inducible $APP^{WT/mut}$. We have updated the legend accordingly. Following your suggestion, we also performed gradient analysis on U2OS cells expressing DOX-inducible $APP^{WT/mut}$-mCherry-EGFP and blotted against mCherry for clearer data. We found that, similar to $APP^{WT/mut}$ without the tandem fluorescence tag, $APP^{WT/mut}$-mCherry-EGFP was enriched in the ER gradient (figures shown below). To avoid redundancy, this data has not been included in the manuscript.

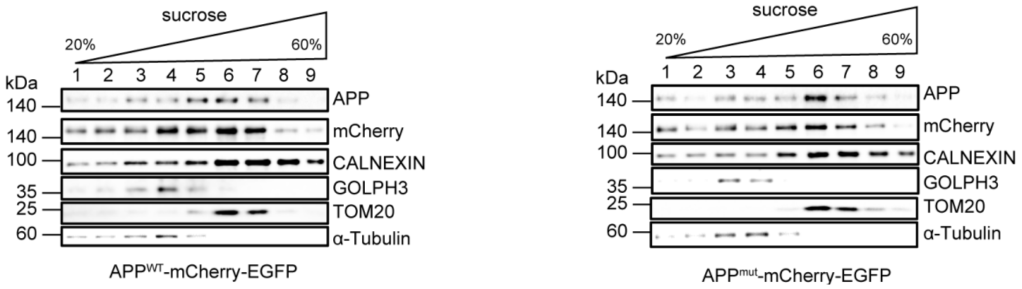

Subcellular fractionation of U2OS cells expressing DOX-inducible APP^{WT}-mCherry-EGFP (**left**) or APP^{mut}-mCherry-EGFP (**right**) cultured under nutrient-rich conditions. Cell lysates were fractionated on a sucrose gradient. CALNEXIN, ER marker; GOLPH3, Golgi marker; TOM20, mitochondrial marker; α-Tubulin, cytosolic marker.

- Fig. 2B, E-H: as above for Fig. S1E.

See response to Fig. S1E.

- Fig. 2C / lane 199ff:

as for Fig. 2B. Are these stable cell lines? This may impact the statistics depending on how the normalization has been done and if non-expressing cells are included in the calculation. Statistics are probably t-test with n=3. Would the authors agree that by increasing the sample size, RTN3L or CALCOCO1 overexpression (quite big SEM compared to other tested samples) may also show significant effects? If so, please reflect this in the text.

We thank the reviewer for this comment. The experiments shown in Fig. 2B/C were performed using stable U2OS cell lines expressing DOX-inducible APP^{WT}–mCherry–EGFP, ensuring consistent expression and excluding non-expressing cells from the analysis. This information has now been clarified in the figure legends and Methods.

In response to the suggestion to increase the sample size, we repeated the experiments with additional independent replicates. Although the larger sample size reduced the SEM, we still did not observe a significant effect of *RTN3L* or *CALCOCO1* overexpression on the lysosomal delivery of APP (new Fig. 2C). This is consistent with Fig. 2B, where knockout of *RTN3L* or *CALCOCO1* did not inhibit APP lysosomal delivery. The updated results have been incorporated into the revised manuscript.

Fig. 2C

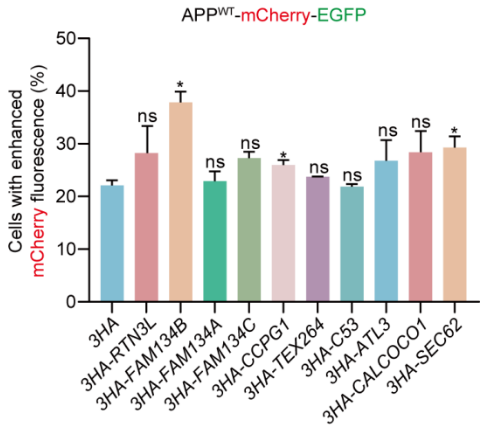

(**2C**) Quantification of lysosomal delivery of APP^{WT}-mCherry-EGFP in control and ER-phagy receptor–overexpressing U2OS cells cultured in nutrient-rich medium. U2OS cells stably expressing DOX-inducible APP^{WT}-mCherry-EGFP were transiently transfected with plasmids encoding the indicated receptors. n = 3 for all conditions except 3HA, 3HA–RTN3L, and 3HA–CALCOCO1 (n = 5).

- Lane 204ff / Fig. S3D/E:

Contradiction between text and actual data. Cleavage (based on mCherry signal alone) was affected by MG132 in WT+EBSS as well as mut- basal. Also unclear why mut+MG+Baf has no effect.

Also not clear, why are APP levels vary this much. Both patterns - APP and mCherry - individually and in relation to each other don't follow any logic mentioned in the text. It is e.g. notable that EBSS treatment dramatically changed the full-length levels of WT but not mut APP, even though the signal for cleaved mCherry did increase in both cases. And APPwt levels already drop drastically upon MG132 treatment and MG+Baf under nutrient rich conditions, while mCherry levels do not increase upon MG treatment. Might this correlate with the different distribution indicated by Fig. S2E under basal conditions? If the authors do not want to discuss this, they may want to remove all cleavage assays from the manuscript.

We thank the reviewer for the careful analysis and apologize for the confusion caused by the original presentation of Fig. S3D/E. We repeated the cleavage assays multiple times and confirmed that the quantitative trends are consistent with our conclusions. The originally shown blot was not fully representative of the quantified data; therefore, we have replaced it with a more representative immunoblot and now provide the corresponding quantification (now Fig. EV4D,E). We also note that APP protein levels indeed vary substantially across conditions. For this reason, quantification was performed using ratios (mCherry / (APP–mCherry + mCherry)) rather than relying on the mCherry signal alone, which would be misleading. In addition, MG-132 has been reported to stimulate

autophagy (Lan *et al*, 2015). Consistent with this, under nutrient-rich conditions MG-132 alone promotes lysosomal cleavage of APP$^{WT/mut}$–mCherry, which is now discussed in the revised manuscript. If the reviewer still considers the blot and quantification insufficiently convincing, we are willing to remove these supplementary data as suggested.

Fig. EV4D

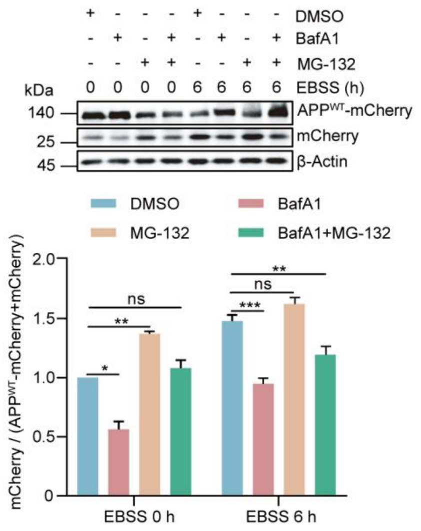

Fig. EV4E

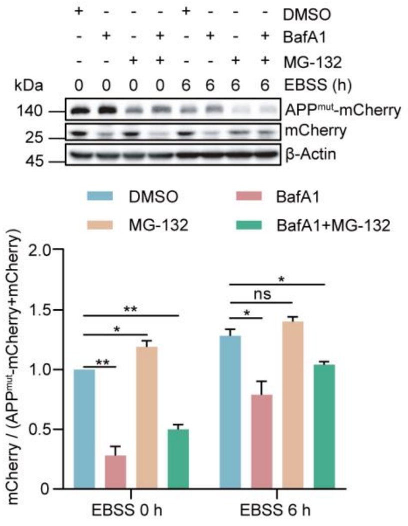

(**EV4D,E**) Immunoblotting of APP$^{WT/mut}$-mCherry cleavage into free mCherry. HEK293T cells expressing DOX-inducible APP^{WT}-mCherry (**D**) or APP^{mut}-mCherry (**E**) were treated as indicated. $n = 4$.

- I suggest to mention RETREG1 as an alternative name for FAM134B in the abstract to improve future findability.

We thank the reviewer for this helpful suggestion. We have now included RETREG1 as an alternative name for FAM134B in the Abstract and keywords to improve clarity and future findability.

Referee #3:

In this manuscript, Zhang et al. identify FAM134B as a mediator of amylod precursor protein (APP) degradation, via FAM134B-APP direct binding and lysosomal delivery via ER-phagy. They found that FAM134B downregulation in cellular and mouse models of Alzheimer's disease (AD) impairs ER-phagy, leading to APP accumulation. The further show that AAV-mediated expression of FAM134B in the hippocampus of an AD mouse model promotes APP clearance and improves cognitive phenotypes.

While the manuscript is potentially interesting (particularly for the in vivo findings), it raises conceptual concerns and the main message is not clearly articulated. Specifically, the authors report that: i) FAM134B-mediated ER-phagy of APP is impaired in AD models due to different mechanisms, including reduced TFEB/TFE3-driven transcription of FAM134B, reduced FAM134B oligomerization, and reduced ubiquitination; ii) APP is degraded via FAM134B-mediated ER-phagy, but this occurs during starvation, and it is unclear why both mutant and wild-type newly synthesized APP should be degraded as part of a quality-control mechanism; iii) FAM134B oligomerization and ubiquitination are impaired, but the underlying reason is not addressed; and iv) FAM134B and APP interact, but the functional significance of this interaction remains unclear. As a result, the manuscript presents several partially supported claims that do not converge into a coherent and robust conceptual flow, leaving the reader uncertain about the central conclusion of the study.

We thank the reviewer for the summary and for raising these conceptual concerns. For point i), as clarified in our response to your Major Comment 2, transcriptional repression of FAM134B, altered post-translational modifications leading to reduced ubiquitylation and impaired oligomerization represent distinct regulatory defects that converge on a single outcome: compromised FAM134B receptor activity. For point ii), as detailed in our response to your Major Comment 1, lysosomal degradation of ER-localized APP—both endogenous and overexpressed—occurs under basal conditions and therefore does not reflect starvation-induced bulk ER turnover. We have revised the manuscript to better emphasize the physiological relevance of this constitutive ER quality-control pathway. Point iii) is also addressed in our response to your Major Comment 2, where we provide mechanistic evidence linking altered post-translational regulation to defects in FAM134B ubiquitylation and oligomerization. For point iv), regarding the functional relevance of the APP–FAM134B interaction, FAM134B lacks a large luminal ER domain and instead recognizes substrates through cytosolic interactions *via* its C-terminal region, independently of chaperone assistance. This mode of recognition supports direct receptor–substrate engagement in selective ER-phagy. Together, these clarifications establish a coherent conceptual framework in which multiple AD-associated regulatory defects converge on impaired FAM134B receptor function, resulting in defective ER-phagy-mediated APP clearance.

Additional major concerns:

1- Whether mutant APP is a true ER-phagy substrate remains unclear, as most experiments have been performed in APP-overexpressing cells, which may lead to

confounding effects due to ER overload. Furthermore, during nutrient starvation, many ER-resident proteins are delivered to lysosomes for degradation, although they are not considered "canonical" ER-phagy substrates. It is also unclear why both wild-type and mutant APP are degraded via ER-phagy to a similar extent. Because APP overexpression can overload the ER, the observation that both WT and mutant APP follow similar degradation routes raises the possibility that the phenotype may simply reflect ER overload rather than selective ER-phagy of APP.

We thank the reviewer for this thoughtful comment and agree that the potential contribution of ER overload should be carefully considered. However, several lines of evidence support the conclusion that APP degradation in our system reflects selective, receptor-dependent ER-phagy rather than non-specific degradation caused by APP overexpression or starvation.

To address whether only overexpressed APP is targeted by ER-phagy, we examined endogenous APP. In Fig. 2A, a subset of endogenous APP colocalized with CALNEXIN and LAMP1 in SH-SY5Y cells under nutrient-rich conditions, consistent with lysosomal targeting of ER-localized endogenous APP. We further found that *FAM134B* knockout in SH-SY5Y cells resulted in increased APP protein levels, consistent with reduced endogenous APP degradation under nutrient-rich conditions (new Fig. 2E). Together, these data support a role for FAM134B in the degradation of endogenous ER-localized APP.

Importantly, FAM134B-dependent lysosomal delivery of APP occurs under nutrient-rich conditions and is not restricted to starvation (Fig. 2B,C; new Fig. 2E). Moreover, the specific requirement for FAM134B—without involvement of other tested ER-phagy receptors—supports the idea that APP is a bona fide ER-phagy substrate rather than a protein degraded non-specifically during starvation.

The similar degradation of wild-type and familial mutant APP can be explained by the location of the FAM134B binding site. The familial AD mutations used in this study lie outside the identified FAM134B-binding region (see also response to Reviewer 2, point 1). Consequently, both wild-type and mutant APP can engage FAM134B and undergo ER-phagy-mediated degradation.

Taken together, these results argue against ER overload as the primary explanation and support that APP is a bona fide substrate of FAM134B-dependent selective ER-phagy.

Fig. 2E

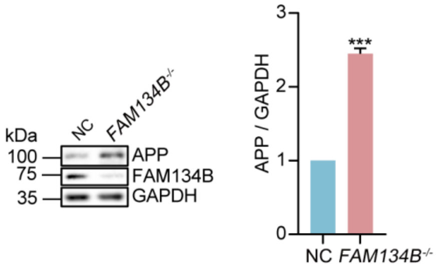

(**2E**) Immunoblotting of protein levels in WT and *FAM134B*-knockout SH-SY5Y cells cultured in nutrient-rich medium. Right: Quantification of protein levels on the left. *n* = 4.

2- The mechanism underlying defective ubiquitination and impaired oligomerization is not clarified. While these defects could be a secondary consequence of reduced FAM134B abundance in the mouse brain, it remains unclear why overexpressed FAM134B in cells still fails to undergo proper ubiquitination and oligomerization.

We thank the reviewer for raising this important question, which was also noted by Reviewer 1. To investigate the underlying mechanism, we examined post-translational modifications of FAM134B that regulate its oligomerization and receptor activity. Phosphorylation at Ser151 and acetylation at Lys160 have been reported to promote FAM134B oligomerization (Jiang *et al.*, 2020; Wang *et al.*, 2023). In 5XFAD mouse brains, both Ser151 phosphorylation and Lys160 acetylation were markedly decreased, indicating impaired post-translational regulation (new Fig. EV2E,F). We further analyzed AMFR, the E3 ubiquitin ligase responsible for FAM134B ubiquitylation during ER-phagy (Gonzalez *et al.*, 2023), and found its expression was also reduced in 5XFAD mouse brains (new Fig. EV2E,F), consistent with the decreased FAM134B ubiquitylation observed (now Fig. EV2G).

Because FAM134B protein abundance is also reduced in 5XFAD mice, these defects could theoretically be secondary to decreased levels. To address this, we expressed 3HA-FAM134B in APP-overexpressing cells to equalize FAM134B abundance. Nevertheless, Ser151 phosphorylation, Lys160 acetylation, and AMFR levels remained decreased in both wild-type and mutant APP-expressing cells despite comparable FAM134B expression (new Fig. EV2I), consistent with our original observations of decreased FAM134B ubiquitylation and oligomerization (original Fig. S1H,J; now Fig. EV2J,K). Together, these findings indicate that defective ubiquitylation and impaired oligomerization of FAM134B arise from altered post-translational regulation rather than simply reduced protein abundance in these AD models. The upstream mechanisms controlling these modifications remain to be determined.

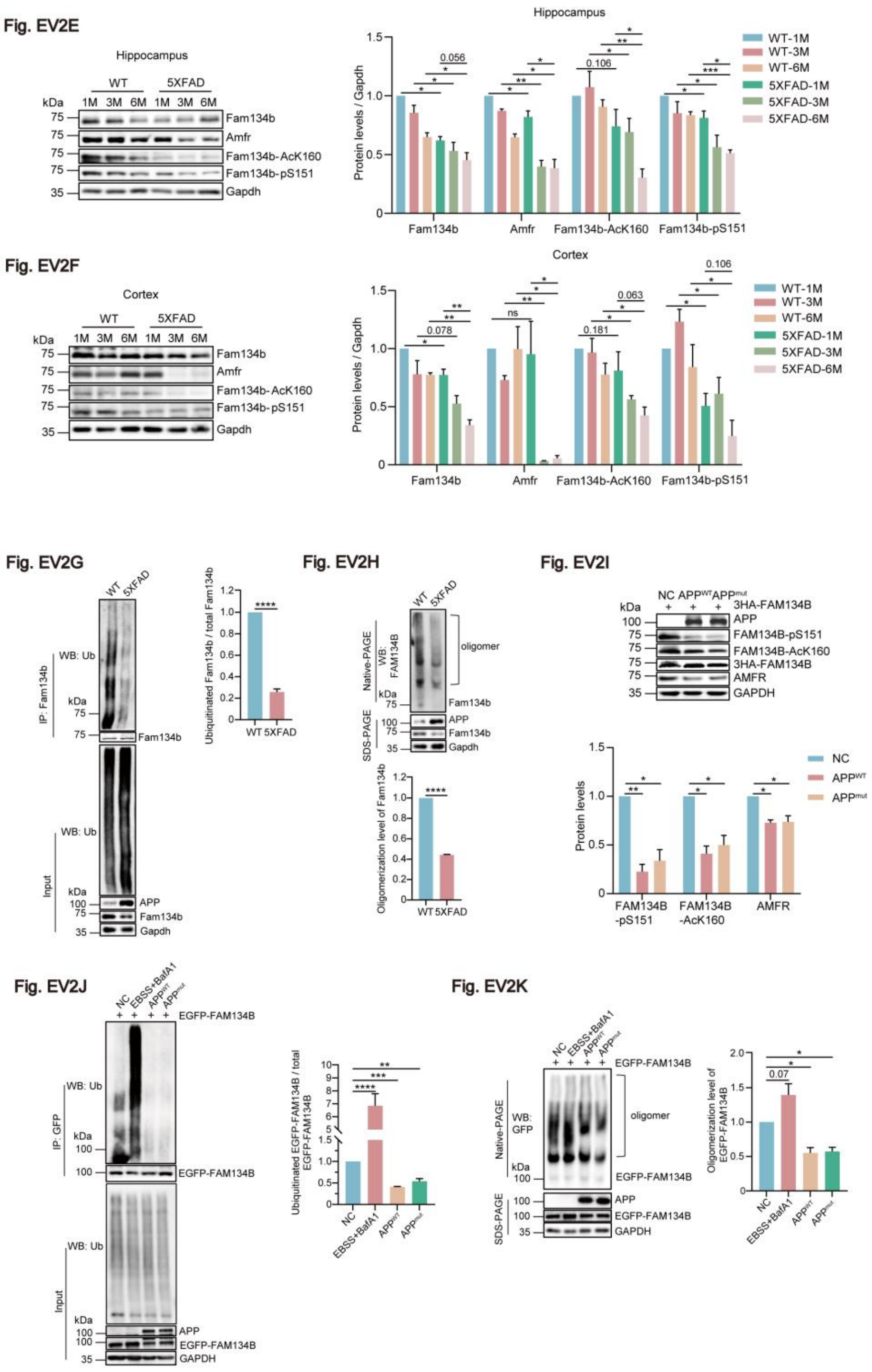

(**EV2E,F**) Immunoblotting of protein levels in the hippocampus (**E**) and cortex (**F**) of 1-, 3-, and 6-month-old 5XFAD female mice and WT littermates (*N* = 3 per group). Fam134b-pS151 and Fam134b-AcK160 indicate Fam134b phosphorylated at Ser151 and acetylated at Lys160, respectively.

(**EV2G**) Fam134b ubiquitination levels in the hippocampus of 6-month-old WT and 5XFAD female mice (*N* = 3 per group). Fam134b was immunoprecipitated with anti-Fam134b antibodies and analyzed by immunoblotting with anti-ubiquitin antibodies. Right:

Quantification of ubiquitinated Fam134b normalized to total Fam134b.

(**EV2H**) Fam134b oligomerization levels in the hippocampus of 6-month-old WT and 5XFAD female mice (*N* = 3 per group). Top: Native PAGE immunoblotting of Fam134b using anti-Fam134b antibodies. Input protein levels were assessed by SDS-PAGE and immunoblotting. Bottom: Quantification of oligomerized Fam134b normalized to total Fam134b.

(**EV2I**) Immunoblotting of protein levels in control and HEK293T cells expressing DOX-inducible $APP^{WT/mut}$. Cells were cultured in nutrient-rich medium and transiently transfected with 3HA–FAM134B. Bottom: Quantification of protein levels shown above. FAM134B-pS151 and FAM134B-AcK160 were normalized to total 3HA–FAM134B; AMFR was normalized to GAPDH. *n* = 3.

(**EV2J**) Effect of $APP^{WT/mut}$ overexpression on FAM134B ubiquitination. Left: EGFP-FAM134B was immunoprecipitated with anti-GFP antibodies and analyzed by immunoblotting with anti-ubiquitin antibodies. Lysates were prepared from untreated U2OS cells (NC), EBSS + BafA1–treated U2OS cells (6 h; positive control), and DOX-induced APPWT/mut-expressing U2OS cells cultured in nutrient-rich medium. All groups were transiently transfected with EGFP-FAM134B. Right: Quantification of ubiquitinated EGFP-FAM134B normalized to total EGFP-FAM134B. *n* = 3.

(**EV2K**) Effect of $APP^{WT/mut}$ overexpression on FAM134B oligomerization. Left: Native PAGE immunoblotting of EGFP-FAM134B using anti-GFP antibodies. Lysates were prepared from NC, EBSS + BafA1, and DOX-induced $APP^{WT/mut}$ U2OS cells. Input protein levels were assessed by SDS-PAGE and immunoblotting. Right: Quantification of oligomerized EGFP-FAM134B normalized to total EGFP-FAM134B. *n* = 3.

3- FAM134B-APP interaction. The C-terminal domain of FAM134B is an intrinsically disordered region lacking known structured domains, making a direct interaction with APP difficult to envision. The authors should perform pull-down assays using truncated FAM134B constructs to narrow the region(s) required for interaction with APP.

We thank the reviewer for this suggestion. To investigate the interaction between FAM134B and APP, we predicted the intrinsically disordered region (IDR) structure of the C-terminal of FAM134B using IUPred2A (Meszaros *et al*, 2018). We identified three major IDR regions: IDR1 (aa 234-320), IDR2 (aa 321-426), and IDR3 (aa 427-497) (Fig. EV5G). We therefore generated truncation mutants retaining only one IDR or lacking individual IDRs while preserving the RHD domains. Co-IP assays showed that APP binding was largely maintained when only IDR2 was present but markedly reduced upon deletion of

IDR2, indicating that the three IDRs act cooperatively with IDR2 providing the primary contribution (Fig. EV5H,I).

Fig. EV5G

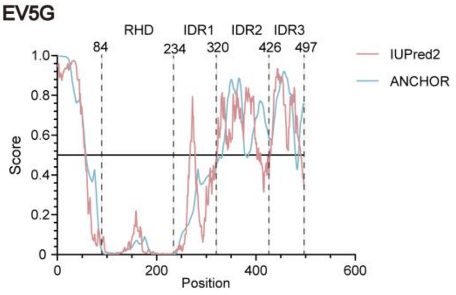

Fig. EV5H

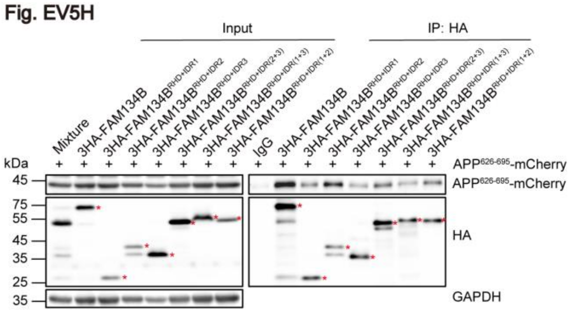

Fig. EV5I

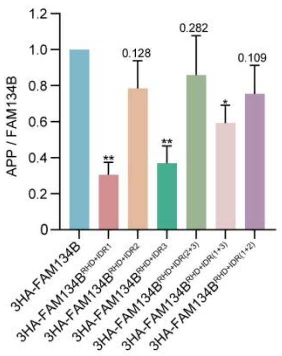

(**EV5G**) Predicted IDR structure of the C-terminus of FAM134B using IUPred2A. Three major IDRs were identified: IDR1 (aa 234–320), IDR2 (aa 321–426), and IDR3 (aa 427–497). IUPred2 (red) predicts IDRs (score > 0.5), while ANCHOR (blue) identifies binding-competent subregions within IDRs that fold upon protein interaction.

(**EV5H**) Co-IP of $APP^{626–695}$–mCherry with IDR-truncated FAM134B to determine which IDR mediates binding to the APP C-terminus. Lysates were prepared from HEK293T cells co-transfected with $APP^{626–695}$–mCherry and the indicated FAM134B constructs.

(**EV5I**) Quantification of APP co-immunoprecipitated with FAM134B in (**H**). *n* = 3.

4- The mechanism linking APP overexpression to epigenetic silencing of FAM134B has not been investigated.

We thank the reviewer for this important question, which was also raised by Reviewer 1 and considered beyond the scope of the current study. Our ChIP–qPCR analyses revealed reduced TFEB/TFE3 binding and decreased H3K4me3 and H3K27ac at the FAM134B locus in both 5XFAD mice and APP-overexpressing cells, whereas DNA methylation at CpG islands remained unchanged. These findings suggest that FAM134B repression is primarily associated with altered histone modifications rather than DNA methylation. However, the upstream mechanism linking APP overexpression to these

epigenetic changes remains to be elucidated and represents an important direction for future investigation.

5- Does FAM134B silencing in the mouse model occur pre- or post-symptomatically? Do the authors detect activation of the ER-stress response in these tissues?

We thank the reviewer for this important question. In the 5XFAD model, intraneuronal Aβ42 accumulation is detectable at around 1.5 months of age. Extracellular amyloid deposition begins at approximately 2 months, followed by synaptic marker loss, neuronal degeneration, and cognitive impairment (Oakley *et al*, 2006). To address whether *FAM134B* silencing occurs pre- or post-symptomatically, we performed qRT-PCR and found that *FAM134B* transcription is downregulated at 1 month of age in the hippocampus and cortex of 5XFAD mice, with a progressive decline as the mice age (new Fig. 1D; EV1E). Thus, *FAM134B* silencing precedes pathological phenotypes. Importantly, APP induction in cultured cells reduces *FAM134B* transcription within 6 hours (now Fig. 1I), indicating a rapid response likely linked directly to APP overexpression/accumulation. Together, these data support that *FAM134B* silencing in the mouse model occurs pre-symptomatically.

Regarding the ER-stress response, we observed activation of all three unfolded protein response (UPR) pathways at both the transcriptional and protein levels in 5XFAD mouse brains, with progressive enhancement during aging (new Fig. EV1F–K). This is consistent with previous reports demonstrating enhanced UPR activation in 5XFAD mice (He *et al*, 2024).

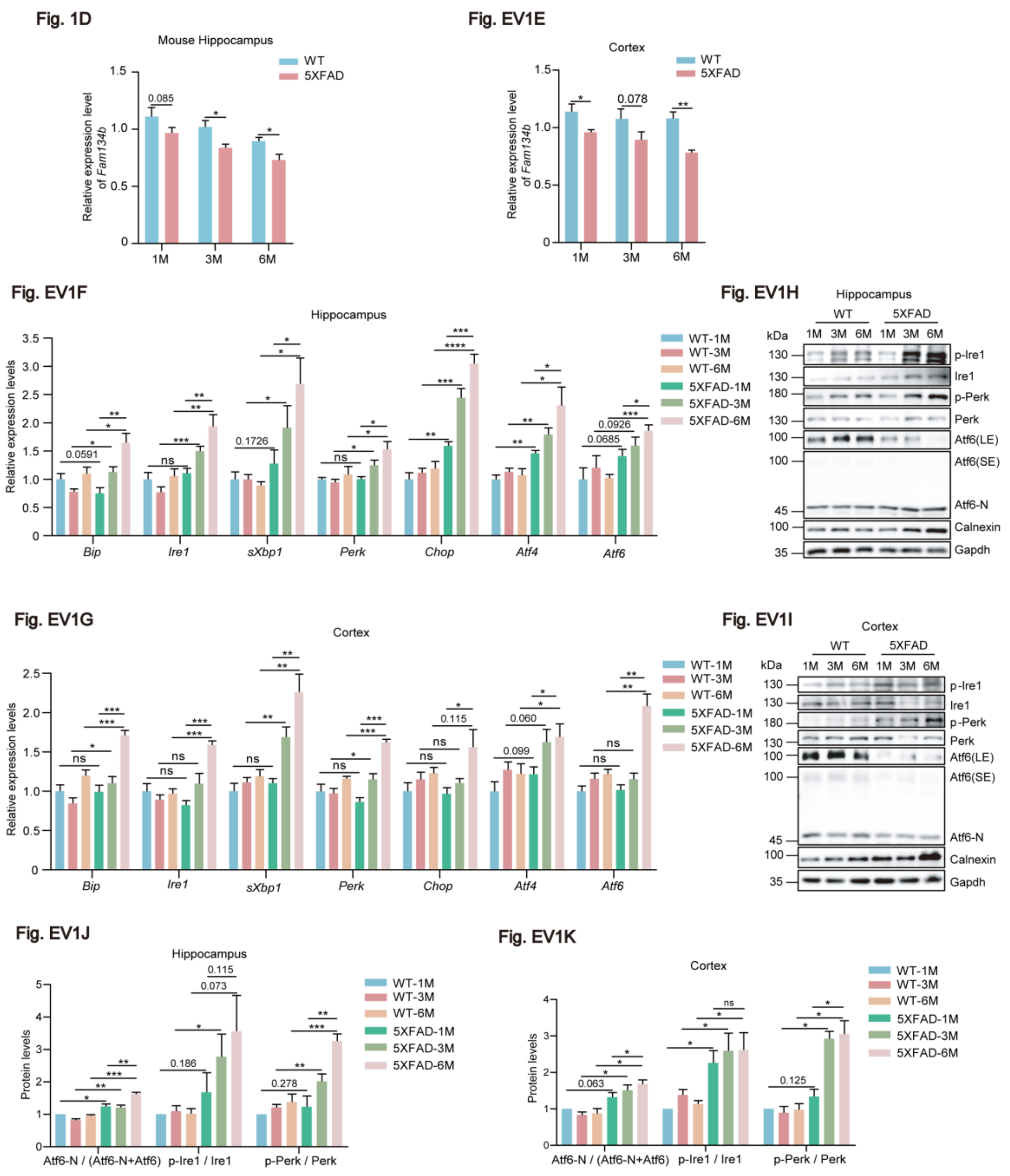

(**1D,EV1E**) qRT-PCR analysis of *Fam134b* mRNA levels in the hippocampus (**1D**) and cortex (**EV1E**) of 1-, 3-, and 6-month-old 5XFAD female mice and WT littermates (*N* = 3 per group).

(**EV1F,G**) qRT-PCR analysis of mRNA levels of UPR-related genes in the hippocampus and cortex of 1-, 3-, and 6-month-old 5XFAD female mice and WT littermates (*N* = 4 per group).

(**EV1H,I**) Immunoblotting of UPR-related protein levels in the hippocampus and cortex of 1-, 3-, and 6-month-old 5XFAD female mice and WT littermates (*N* = 3 per group). p-Ire1 and p-Perk represent phosphorylated Ire1 and Perk, indicative of activated Ire1 and Perk; Atf6-N represents the cleaved, transcriptionally active N-terminal fragment of Atf6. LE, long exposure; SE, short exposure.

(**EV1J**,**K**) Quantification of protein levels in (**H**,**I**) (*N* = 3 per group).

6- It would be important to test whether BafA treatment rescues the reduction of FAM134B levels, which could be explained by increased FAM134B degradation. Indeed, the APP-induced transcriptional downregulation of FAM134B, albeit statistically significant, appears minimal.

Following your suggestion, we treated cells expressing wild-type or mutant APP with BafA1 or MG-132. Neither treatment restored FAM134B protein levels, indicating that the decrease is not attributable to enhanced protein degradation (Fig. EV2A).

Fig. EV2A

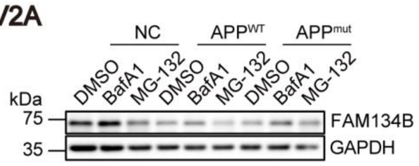

(**EV2A**) Immunoblotting of protein levels in control and HEK293T cells expressing DOX-inducible APP$^{WT/mut}$. Cells were either untreated or treated with BafA1 or MG-132 for 6 h.

7- It is unclear which FAM134B isoform is regulated by TFEB/TFE3 in cultured cells or in vivo. The authors should clarify this point, particularly in relation to the reported epigenetic changes.

We thank the reviewer for this important comment. Throughout the manuscript, we specifically focus on the longest FAM134B isoform, FAM134B-1 (referred to as FAM134B in our study), rather than the N-terminally truncated isoform FAM134B-2. Previous studies have shown that FAM134B-1 is highly expressed in the brain, whereas FAM134B-2 is more abundant in other tissues, such as the liver (Kohno *et al*, 2019). To determine the effects of TFEB/TFE3 overexpression on *FAM134B* transcription, we designed isoform-specific primers targeting the unique N-terminal region of human and mouse *FAM134B-1*. This strategy ensured selective detection of *FAM134B-1* in both cultured cells and *in vivo* experiments. We have now clarified this point explicitly in the revised manuscript.

8- The in vivo results are compelling, but the authors examined neuronal cell bodies, synapses, and myelin without demonstrating that these cells were indeed transduced by AAV-FAM134B. Since AAV does not efficiently transduce most cells, the authors should include co-staining to confirm FAM134B expression in the analysed regions.

We thank the reviewer for this valuable comment. As correctly noted, our immunofluorescence analysis shows that AAV delivery of EGFP or EGFP-FAM134B$^{WT/mutLIR}$ predominantly transduced cells in the hippocampus, with limited

spread to other brain regions (new EGFP panel added to Fig. 6C). Nevertheless, we observed broader phenotypic improvements, which we interpret as a secondary consequence of enhanced APP clearance in the hippocampus—likely reducing APP processing and downstream Aβ production and thereby influencing connected neural circuits.

For synapse and myelin analyses, we used conventional transmission electron microscopy rather than immuno-electron microscopy, and therefore cannot directly verify FAM134B expression within each ultrastructurally analyzed region. We attempted immuno-electron microscopy to assess FAM134B expression, but the signal quality was insufficient for reliable interpretation. Importantly, the observed global phenotypic improvements do not necessarily imply direct FAM134B expression in all analyzed regions.

Fig. 6C

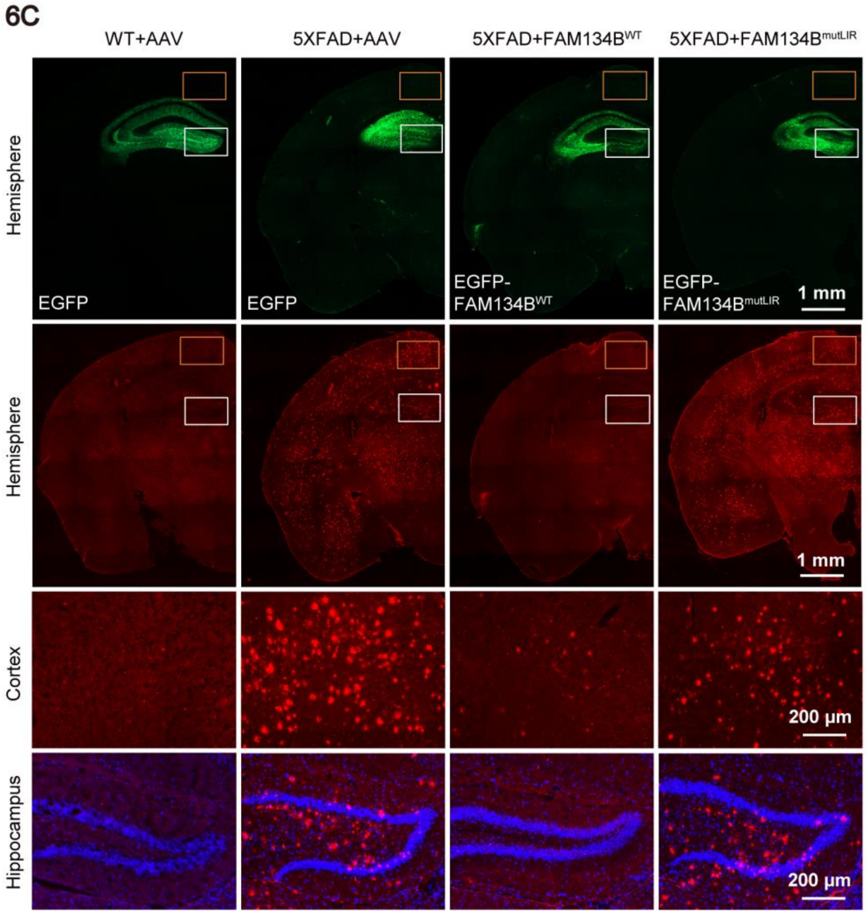

(**6C**) Confocal images of mouse brains stereotactically injected into the hippocampus with AAVs expressing EGFP-FAM134B^{WT}, EGFP-FAM134B^{mutLIR}, or EGFP control. Immunostaining for Aβ (red) and nuclei (DAPI, blue) was performed. EGFP signals indicate predominant transduction in the hippocampus. WT + AAV (*N* = 3); 5XFAD + AAV (*N* = 4); 5XFAD + FAM134B^{WT} (*N* = 4); 5XFAD + FAM134B^{mutLIR} (*N* = 4).

9- In the introduction there is a misconception that ER-phagy is associated with

neurodegeneration. This is not supported by current evidence, at least in humans. Instead, ER-phagy alterations appear to be linked primarily to neuropathy and possibly to defects in neurogenesis, but not directly to neurodegeneration.

We thank the reviewer for this comment. As suggested, we have revised the introduction to state that "ER-phagy regulates neurogenesis (Hoyer et al., 2024), and its dysfunction is implicated in neuropathy."

We express our sincere gratitude to the reviewers for their insightful and constructive comments, which have greatly contributed to enhancing the rigor of our study and providing clarity to the conclusions presented in this manuscript. By diligently following their guidance, we have made substantial improvements to our paper, aiming to meet the rigorous standards required for publication in the esteemed *EMBO Journal*.

Best wishes,

Yixian

Yixian Cui, Ph.D.

Medical Research Institute

Wuhan University, China

E-mail: yixian.cui@whu.edu.cn

References

Gonzalez A, Covarrubias-Pinto A, Bhaskara RM, Glogger M, Kuncha SK, Xavier A, Seemann E, Misra M, Hoffmann ME, Brauning B *et al* (2023) Ubiquitination regulates ER-phagy and remodelling of endoplasmic reticulum. *Nature* 618: 394–401

He X, Peng Y, Huang S, Xiao Z, Li G, Zuo Z, Zhang L, Shuai X, Zheng H, Hu X (2024) Blood Brain Barrier-Crossing Delivery of Felodipine Nanodrug Ameliorates Anxiety-Like Behavior and Cognitive Impairment in Alzheimer's Disease. *Adv Sci (Weinh)* 11: e2401731

Ishii S, Chino H, Ode KL, Kurikawa Y, Ueda HR, Matsuura A, Mizushima N, Itakura E (2023) CCPG1 recognizes endoplasmic reticulum luminal proteins for selective ER-phagy. *Mol Biol Cell* 34: ar29

Jiang X, Wang X, Ding X, Du M, Li B, Weng X, Zhang J, Li L, Tian R, Zhu Q *et al* (2020) FAM134B oligomerization drives endoplasmic reticulum membrane scission for ER-phagy. *EMBO J* 39: e102608

Kohno S, Shiozaki Y, Keenan AL, Miyazaki-Anzai S, Miyazaki M (2019) An N-terminal-truncated isoform of FAM134B (FAM134B-2) regulates starvation-induced hepatic selective ER-phagy. *Life Sci Alliance* 2

Lan D, Wang W, Zhuang J, Zhao Z (2015) Proteasome inhibitor-induced autophagy in PC12 cells overexpressing A53T mutant alpha-synuclein. *Mol Med Rep* 11: 1655–1660

Meszaros B, Erdos G, Dosztanyi Z (2018) IUPred2A: context-dependent prediction of protein disorder as a function of redox state and protein binding. *Nucleic Acids Res* 46: W329–W337

Oakley H, Cole SL, Logan S, Maus E, Shao P, Craft J, Guillozet-Bongaarts A, Ohno M, Disterhoft J, Van Eldik L *et al* (2006) Intraneuronal beta-amyloid aggregates, neurodegeneration, and neuron loss in transgenic mice with five familial Alzheimer's disease mutations: potential factors in amyloid plaque formation. *J Neurosci* 26: 10129–10140

Qian X, He L, Yang J, Sun J, Peng X, Zhang Y, Mao Y, Zhang Y, Cui Y (2023) UVRAG cooperates with cargo receptors to assemble the ER-phagy site. *EMBO J* 42: e113625

Wang X, Jiang X, Li B, Zheng J, Guo J, Gao L, Du M, Weng X, Li L, Chen S *et al* (2023) A regulatory circuit comprising the CBP and SIRT7 regulates FAM134B-mediated ER-phagy. *J Cell Biol* 222

Yang J, Dong Y, Xu J, Qian X, Cai Y, Chen Y, Zhang P, Gao D, Cui Z, Cui Y (2025) TM9SF3 is a Golgi-resident ATG8-binding protein essential for Golgi-selective autophagy. *Dev Cell* 60: 2862–2879 e2868

1st Revision - Editorial Decision 16th Apr 2026

Dear Prof. Cui,

We have now received re-review reports from two referees, which I have included below. As you will see, you have addressed their concerns satisfactorily. There are some remaining editorial points which need to be addressed. In this regard would you please:

- declare employment in a biotech company in the disclosure and competing interests statement,
- add all funders' information to the "More Funders" list without duplication in the comments box (as the information cannot currently be extracted by our production team),
- remove the AC/CrediT section from the text,
- include text callouts for Fig. 4H-J, 5C-E, J-M, O-P, 6D-E,
- upload figures in individual, high-resolution files,
- provide exact p values in the legends of figures 1B, C, D, F, H, I, K; 2A-K; 3B, H, K, L; 4A, C, D, F, H, J, K, L, O, 5C, D, E, F, 6D, E, G; EV1 C, D, E, F, G, J, K; EV2 B-K; EV3 E, G, H; EV4 D-F; EV5 B, EV6 A, C; EV7 B, C, E, G, I, J, K,
- define n in the legends of figures 1B, 5L-Q; EV1 C, EV7 E,
- define the red and orange lines in the legend of figure 5J, K,
- define arrows in the legend of figure EV4 C, EV5 H, and
- correct the names and order of sections as follows: Title page - Abstract - Keywords - Introduction - Results - Discussion - Methods - Data Availability - Acknowledgements - Disclosure and Competing Interests Statement - References - Figure Legends - Table(s) - Expanded View Figure Legends.

During a standard image analysis, we detected potential aberrations in the figure set, and we would like to clarify these issues before proceeding further. Please check the composition of:
*Figure 1E - Cell reuse / Partial cell reuse detected.
The provided source data confirms partial cell reuse between panels. Please provide us with the unprocessed original captured images. Please provide any captured information (metadata) regarding date, time and settings.

We include a synopsis of the paper (see http://emboj.embopress.org/). Please provide me with a general summary image, a two sentence statement and 3-5 bullet points that capture the key findings of the paper.

I am looking forward to receiving your revised manuscript.

EMBO Press is an editorially independent publishing platform for the development of EMBO scientific publications.

Best wishes,

William

William Teale, PhD
Editor
The EMBO Journal
w.teale@embojournal.org

Instructions for preparing your revised manuscript:

Read our guidance for manuscript revisions and related editorial policies: https://link.springer.com/journal/44318/submission-guidelines#cms-Revised-submissions

Please also check that the title and abstract of the manuscript are brief, yet explicit, even to non-specialists.

When assembling figures, please refer to our figure preparation guideline in order to ensure proper formatting and readability in print as well as on screen:
https://media.springernature.com/original/springer-cms/rest/v1/content/27825798/data/v1

IMPORTANT: When you send the revision we will require
- a point-by-point response to the referees' comments, with a detailed description of the changes made (as a word file).
- a word file of the manuscript text.
- individual production quality figure files (one file per figure)
- a complete author checklist

- Expanded View files (replacing Supplementary Information)
- a Reagents and Tools Table as part of the Methods section

Please remember: Digital image enhancement is acceptable practice, as long as it accurately represents the original data and conforms to community standards. If a figure has been subjected to significant electronic manipulation, this must be noted in the figure legend or in the 'Methods' section. The editors reserve the right to request original versions of figures and the original images that were used to assemble the figure.

We realize that it is difficult to revise to a specific deadline. In the interest of protecting the conceptual advance provided by the work, we recommend a revision within 3 months (15th Jul 2026). Please discuss the revision progress ahead of this time with the editor if you require more time to complete the revisions. Use the link below to submit your revision:

https://emboj.msubmit.net/cgi-bin/main.plex

Referee #2:

The authors have sufficiently addressed my concerns. I recommend publication of the manuscript.

Referee #3:

The authors addressed all my previous concerns, resulting in a greatly improved manuscript.

Dear Dr. Teale,

Thank you for your letter and the valuable feedback provided by the reviewers regarding our manuscript entitled "FAM134B-mediated ER-phagy degrades APP and protects against Alzheimer's disease pathology" (EMBOJ-2025-122653R). We greatly appreciate the reviewers' time and effort in evaluating our work. Below, we present a detailed response to each point raised by you and the reviewers. We are grateful for the opportunity to address these issues and improve the quality of our manuscript.

Our point-by-point responses are provided below:

Dear Prof. Cui,

We have now received re-review reports from two referees, which I have included below. As you will see, you have addressed their concerns satisfactorily. There are some remaining editorial points which need to be addressed. In this regard would you please:

- declare employment in a biotech company in the disclosure and competing interests statement,

- add all funders' information to the "More Funders" list without duplication in the comments box (as the information cannot currently be extracted by our production team),

- remove the AC/CrediT section from the text,

All done.

- include text callouts for Fig. 4H-J, 5C-E, J-M, O-P, 6D-E,

We would like to clarify that the figures you mentioned (Fig. 4H–J, 5C–E, 5J–M, 5O–P, 6D–E) have already been cited in the original manuscript. Specifically:

Fig. 4H–J are included in the citation “Fig. 4G–J” on line 385.

Fig. 5C–E are included in the citation “Fig. 5B–E” on line 424.

Fig. 5J–M and 5O–P are included in the citation “Fig. 5J–M, K–P” on line 447.

Fig. 6D–E are included in the citation “Fig. 6C–E” on line 475.

- upload figures in individual, high-resolution files,

- provide exact p values in the legends of figures 1B, C, D, F, H, I, K; 2A-K; 3B, H, K, L; 4A, C, D, F, H, J, K, L, O, 5C, D, E, F, 6D, E, G; EV1 C, D, E, F, G, J, K; EV2 B-K; EV3 E, G, H; EV4 D-F; EV5 B, EV6 A, C; EV7 B, C, E, G, I, J, K,

Providing exact *p* values individually in the figure legends would be lengthy and less intuitive. Following the format used in recent articles published in *The EMBO Journal*, we have instead indicated the exact *p* values directly on all the figures.

- define n in the legends of figures 1B, 5L-Q; EV1 C, EV7 E,

- define the red and orange lines in the legend of figure 5J, K,
- define arrows in the legend of figure EV4 C, EV5 H, and
- correct the names and order of sections as follows: Title page - Abstract - Keywords - Introduction - Results - Discussion - Methods - Data Availability - Acknowledgements - Disclosure and Competing Interests Statement - References - Figure Legends - Table(s) - Expanded View Figure Legends.

All done.

During a standard image analysis, we detected potential aberrations in the figure set, and we would like to clarify these issues before proceeding further. Please check the composition of:
*Figure 1E - Cell reuse / Partial cell reuse detected.
The provided source data confirms partial cell reuse between panels. Please provide us with the unprocessed original captured images. Please provide any captured information (metadata) regarding date, time and settings.
We sincerely apologize for this error. During figure assembly, we mistakenly uploaded the quantification images for Control #1 and AD #3 as Control #2 and AD #4, respectively. These images were acquired from a larger field of view, from which smaller regions were cropped and enlarged for the main figure. This led to partial overlap of cells between Control #1 and Control #2, as well as between AD #3 and AD #4. We have now corrected this by re-uploading the appropriate original images for Control #2 and AD #4.

In addition, we have provided the unprocessed original Zeiss confocal (.czi) files for all panels in Figure 1E, along with the corresponding metadata (including acquisition date, time, and imaging settings). For accessibility, we have also uploaded PNG files showing the metadata extracted from the original files, in case the .czi format cannot be opened. All original data are fully consistent with the images presented in Figure 1E.

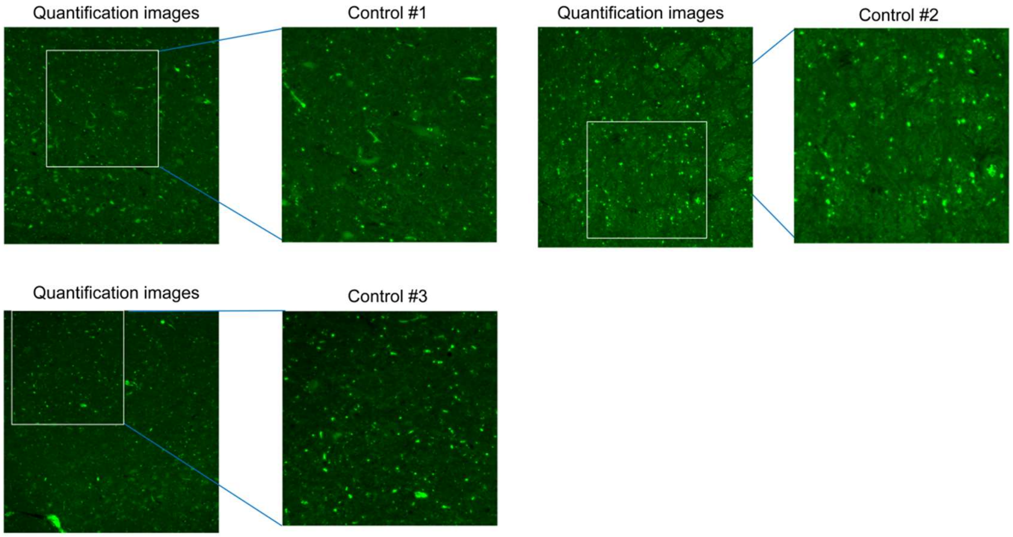

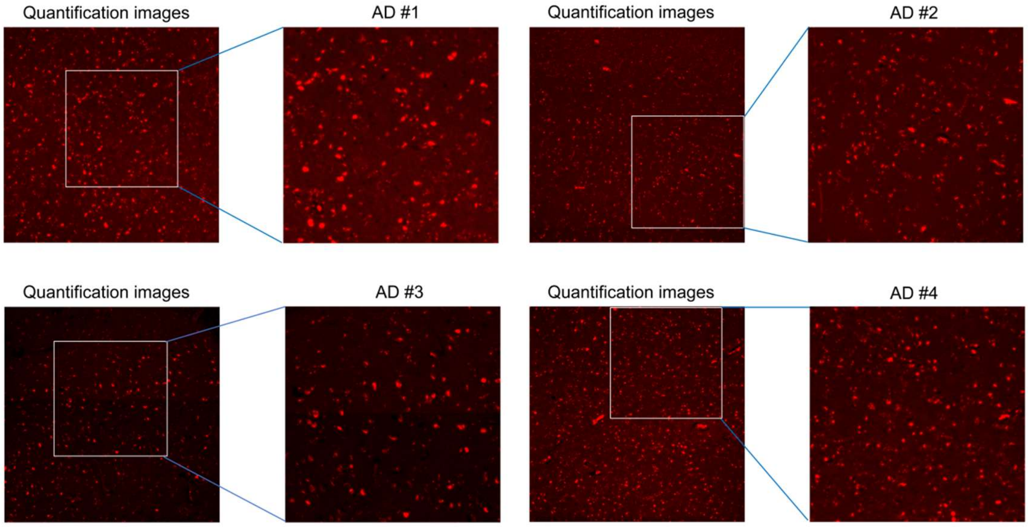

We include a synopsis of the paper (see http://emboj.embopress.org/). Please provide me with a general summary image, a two sentence statement and 3-5 bullet points that capture the key findings of the paper.
Uploaded.

We have transferred the content of the original Table EV3, EV4, and EV5 into the Reagents and Tools Table. The original Table EV6 has been renumbered as Table EV3. Table EV1, EV2 and EV3 will be uploaded in a folder named "Tables”.

I am looking forward to receiving your revised manuscript.

EMBO Press is an editorially independent publishing platform for the development of

EMBO scientific publications.

Best wishes,

William

William Teale, PhD
Editor
The EMBO Journal
w.teale@embojournal.org

Referee #2:

The authors have sufficiently addressed my concerns. I recommend publication of the manuscript.

We thank the reviewer for the positive evaluation and recommendation for publication.

Referee #3:

The authors addressed all my previous concerns, resulting in a greatly improved manuscript.

We thank the reviewer for the constructive comments, which have improved the manuscript.

We sincerely thank the Editor, the *EMBO Journal* team, and the reviewers for their time and consideration. We look forward to hearing from you.

Best wishes,

Yixian

Yixian Cui, Ph.D.

Medical Research Institute

Wuhan University, China

E-mail: yixian.cui@whu.edu.cn

Dear Prof. Cui,

I am pleased to inform you that your manuscript has been accepted for publication in the EMBO Journal.

Congratulations to you and all involved!

Your manuscript will be processed for publication by EMBO Press. It will be copy edited and you will receive page proofs prior to publication. Please note that you will be contacted by Springer Nature Author Services to complete licensing and payment information.

You may qualify for financial assistance for your publication charges - either via a Springer Nature fully open access agreement or an EMBO initiative. Check your eligibility: https://link.springer.com/journal/44318/how-to-publish-with-us

Should you be planning a Press Release on your article, please get in contact with embo_production@springernature.com as early as possible in order to coordinate publication and release dates.

If you have any questions, please do not hesitate to contact the Editorial Office. Thank you for your contribution to The EMBO Journal.

Yours sincerely,

William Teale

William Teale, PhD
Editor
The EMBO Journal
w.teale@embojournal.org

*****
Please note that it is The EMBO Journal policy for the transcript of the editorial process (containing referee reports and your response letters) to be published as an online supplement to each paper. If you should prefer removal of any referee-only figures included in the point-by-point response(s), e.g. because they may still be used for future publication or because they have been reproduced from published work by others, please do let us know immediately via response email.
More information is available here: https://link.springer.com/partners/embo-press/editorial-policies#Peer%20review

** Click here to be directed to your login page: https://emboj.msubmit.net

EMBO Press Author Checklist

Corresponding Author Name: Yixian Cui	
Journal Submitted to: The EMBO Journal	
Manuscript Number: 2025-122653R	

USEFUL LINKS FOR COMPLETING THIS FORM
The EMBO Journal - Author Guidelines
EMBO Reports - Author Guidelines
Molecular Systems Biology - Author Guidelines
EMBO Molecular Medicine - Author Guidelines

Reporting Checklist for Life Science Articles (updated January
This checklist is adapted from Materials Design Analysis Reporting (MDAR) Checklist for Authors. MDAR establishes a minimum set of requirements in transparent reporting in the life sciences (see Statement of Task: 10.31222/osf.io/9sm4x). Please follow the journal's guidelines in preparing your
Please note that a copy of this checklist will be published alongside your article.

Abridged guidelines for figures
1. Data
The data shown in figures should satisfy the following conditions:
- the data were obtained and processed according to the field's best practice and are presented to reflect the results of the experiments in an accurate and unbiased manner.
- ideally, figure panels should include only measurements that are directly comparable to each other and obtained with the same assay.
- plots include clearly labeled error bars for independent experiments and sample sizes. Unless justified, error bars should not be shown for technical
- if n<5, the individual data points from each experiment should be plotted. Any statistical test employed should be justified.
- Source Data should be included to report the data underlying figures according to the guidelines set out in the authorship guidelines on Data

2. Captions
Each figure caption should contain the following information, for each panel where they are relevant:
- a specification of the experimental system investigated (eg cell line, species name).
- the assay(s) and method(s) used to carry out the reported observations and measurements.
- an explicit mention of the biological and chemical entity(ies) that are being measured.
- an explicit mention of the biological and chemical entity(ies) that are altered/varied/perturbed in a controlled manner.
- the exact sample size (n) for each experimental group/condition, given as a number, not a range;
- a description of the sample collection allowing the reader to understand whether the samples represent technical or biological replicates (including how many animals, litters, cultures, etc.).
- a statement of how many times the experiment shown was independently replicated in the laboratory.
- definitions of statistical methods and measures:
  - common tests, such as t-test (please specify whether paired vs. unpaired), simple χ2 tests, Wilcoxon and Mann-Whitney tests, can be unambiguously identified by name only, but more complex techniques should be described in the methods section;
  - are tests one-sided or two-sided?
  - are there adjustments for multiple comparisons?
  - exact statistical test results, e.g., P values = x but not P values < x;
  - definition of 'center values' as median or average;
  - definition of error bars as s.d. or s.e.m.

Please complete ALL of the questions below.
Select "Not Applicable" only when the requested information is not relevant for your study.

Materials

Newly Created Materials	Information included in the manuscript?	In which section is the information available? (Reagents and Tools Table, Materials and Methods, Figures, Data Availability Section)
New materials and reagents need to be available; do any restrictions apply?	Not Applicable	

Antibodies	Information included in the manuscript?	In which section is the information available? (Reagents and Tools Table, Materials and Methods, Figures, Data Availability Section)
For **antibodies** provide the following information:<br>- Commercial antibodies: RRID (if possible) or supplier name, catalogue number and or/clone number<br>- Non-commercial: RRID or citation	Yes	Reagents and Tool Tables

DNA and RNA sequences	Information included in the manuscript?	In which section is the information available? (Reagents and Tools Table, Materials and Methods, Figures, Data Availability Section)
**Short novel DNA or RNA including primers, probes**: provide the sequences.	Yes	Reagents and Tool Tables

Cell materials	Information included in the manuscript?	In which section is the information available? (Reagents and Tools Table, Materials and Methods, Figures, Data Availability Section)
**Cell lines:** Provide species information, strain. Provide accession number in repository **OR** supplier name, catalog number, clone number, and/**OR** RRID.	Yes	Materials and Methods
**Primary cultures:** Provide species, strain, sex of origin, genetic modification status.	Not Applicable	
Report if the cell lines were recently **authenticated** (e.g., by STR profiling) and tested for mycoplasma contamination.	Not Applicable	

Experimental animals	Information included in the manuscript?	In which section is the information available? (Reagents and Tools Table, Materials and Methods, Figures, Data Availability Section)
**Laboratory animals or Model organisms:** Provide species, strain, sex, age, genetic modification status. Provide accession number in repository **OR** supplier name, catalog number, clone number, **OR** RRID.	Yes	Materials and Methods
**Animal observed in or captured from the field:** Provide species, sex, and age where possible.	Not Applicable	
Please detail **housing and husbandry conditions**.	Yes	Materials and Methods

Plants and microbes	Information included in the manuscript?	In which section is the information available? (Reagents and Tools Table, Materials and Methods, Figures, Data Availability Section)
**Plants:** provide species and strain, ecotype and cultivar where relevant, unique accession number if available, and source (including location for collected wild specimens).	Not Applicable	
**Microbes:** provide species and strain, unique accession number if available, and source.	Not Applicable	

Human research participants	Information included in the manuscript?	In which section is the information available? (Reagents and Tools Table, Materials and Methods, Figures, Data Availability Section)
If collected and within the bounds of privacy constraints report on age, sex and gender or ethnicity for all study participants.	Not Applicable	

Core facilities	Information included in the manuscript?	In which section is the information available? (Reagents and Tools Table, Materials and Methods, Figures, Data Availability Section)
If your work benefited from core facilities, was their service mentioned in the acknowledgments section?	Yes	Acknowledgements

Design

Study protocol	Information included in the manuscript?	In which section is the information available? (Reagents and Tools Table, Materials and Methods, Figures, Data Availability Section)
If study protocol has been **pre-registered, provide DOI in the manuscript**. For clinical trials, provide the trial registration number **OR** cite DOI.	Not Applicable	
Report the **clinical trial registration number** (at ClinicalTrials.gov or equivalent), where applicable.	Not Applicable	

Laboratory protocol	Information included in the manuscript?	In which section is the information available? (Reagents and Tools Table, Materials and Methods, Figures, Data Availability Section)
Provide DOI OR other citation details if **external detailed step-by-step protocols** are available.	Not Applicable	

Experimental study design and statistics	Information included in the manuscript?	In which section is the information available? (Reagents and Tools Table, Materials and Methods, Figures, Data Availability Section)
Include a statement about **sample size** estimate even if no statistical methods were used.	Not Applicable	
Were any steps taken to minimize the effects of subjective bias when allocating animals/samples to treatment (e.g. **randomization procedure**)? If yes, have they been described?	Yes	Materials and Methods
Include a statement about **blinding** even if no blinding was done.	Not Applicable	
Describe **inclusion/exclusion criteria** if samples or animals were excluded from the analysis. Were the criteria pre-established?<br><br>If sample or data points were omitted from analysis, report if this was due to attrition or intentional exclusion and provide justification.	Not Applicable	
For every figure, are **statistical tests** justified as appropriate? Do the data meet the assumptions of the tests (e.g., normal distribution)? Describe any methods used to assess it. Is there an estimate of variation within each group of data? Is the variance similar between the groups that are being statistically compared?	Yes	Figures

Sample definition and in-laboratory replication	Information included in the manuscript?	In which section is the information available? (Reagents and Tools Table, Materials and Methods, Figures, Data Availability Section)
In the figure legends: state number of times the experiment was **replicated** in laboratory.	Yes	Figures
In the figure legends: define whether data describe **technical or biological replicates**.	Yes	Figures

Ethics

Ethics	Information included in the manuscript?	In which section is the information available? (Reagents and Tools Table, Materials and Methods, Figures, Data Availability Section)
Studies involving **human participants**: State details of **authority granting ethics approval** (IRB or equivalent committee(s), provide reference number for approval.	Not Applicable	
Studies involving **human participants**: Include a statement confirming that **informed consent** was obtained from all subjects and that the experiments conformed to the principles set out in the WMA Declaration of Helsinki and the Department of Health and Human Services Belmont Report.	Not Applicable	
Studies involving **human participants:** For publication of **patient photos**, include a statement confirming that consent to publish was obtained.	Not Applicable	
Studies involving experimental **animals**: State details of **authority granting ethics approval** (IRB or equivalent committee(s), provide reference number for approval. Include a statement of compliance with ethical regulations.	Yes	Materials and Methods
Studies involving **specimen and field samples:** State if relevant **permits** obtained, provide details of authority approving study; if none were required, explain why.	Yes	Materials and Methods

Dual Use Research of Concern (DURC)	Information included in the manuscript?	In which section is the information available? (Reagents and Tools Table, Materials and Methods, Figures, Data Availability Section)
Could your study fall under dual use research restrictions? Please check biosecurity documents and list of **select agents and toxins** (CDC): https://www.selectagents.gov/sat/list.htm	Not Applicable	
If you used a select agent, is the security level of the lab appropriate and reported in the manuscript?	Not Applicable	
If a study is subject to dual use research of concern regulations, is the name of the **authority granting approval and reference number** for the regulatory approval provided in the manuscript?	Not Applicable	

Reporting

The MDAR framework recommends adoption of discipline-specific guidelines, established and endorsed through community initiatives. Journals have their own policy about requiring specific guidelines and recommendations to complement MDAR.

Adherence to community standards	Information included in the manuscript?	In which section is the information available? (Reagents and Tools Table, Materials and Methods, Figures, Data Availability Section)
State if relevant guidelines or checklists (e.g., **ICMJE, MIBBI, ARRIVE, PRISMA**) have been followed or provided.	Not Applicable	
For **tumor marker prognostic studies,** we recommend that you follow the **REMARK** reporting guidelines (see link list at top right). See author guidelines, under 'Reporting Guidelines'. Please confirm you have followed these guidelines.	Not Applicable	
For **phase II and III randomized controlled trials**, please refer to the **CONSORT** flow diagram (see link list at top right) and submit the CONSORT checklist (see link list at top right) with your submission. See author guidelines, under 'Reporting Guidelines'. Please confirm you have submitted this list.	Not Applicable	

Data Availability

Data availability	Information included in the manuscript?	In which section is the information available? (Reagents and Tools Table, Materials and Methods, Figures, Data Availability Section)
Have **primary datasets** been deposited according to the journal's guidelines (see 'Data Deposition' section) and the respective accession numbers provided in the Data Availability Section?	Not Applicable	
Were **human clinical and genomic datasets** deposited in a public access-controlled repository in accordance to ethical obligations to the patients and to the applicable consent agreement?	Not Applicable	
Are **computational models** that are central and integral to a study available without restrictions in a machine-readable form? Were the relevant accession numbers or links provided?	Not Applicable	
If publicly available data were reused, provide the respective **data citations in the reference list**.	Yes	Materials and Methods
